# A cadmium stress-responsive gene *AtFC1* confers plant tolerance to cadmium toxicity

**DOI:** 10.1186/s12870-017-1141-0

**Published:** 2017-10-30

**Authors:** Jun Song, Sheng Jun Feng, Jian Chen, Wen Ting Zhao, Zhi Min Yang

**Affiliations:** 1 Department of Biochemistry and Molecular Biology, College of Life Sciences, Nanjing Agricultural University, Nanjing, 210095 China; 2 Institute of Food Quality and Safety, Jiangsu Academy of Agricultural Sciences, Nanjing, 210014 China; 3 Institute of Plant Nutrition (IFZ), Justus Liebig University, Heinrich-Buff-Ring 26-32, 35392 Giessen, Germany

**Keywords:** Ferrochelatase-1, Arabidopsis, Cadmium, Glutathione, Phytochelatins

## Abstract

**Background:**

Non-essential trance metal such as cadmium (Cd) is toxic to plants. Although some plants have developed elaborate strategies to deal with absorbed Cd through multiple pathways, the regulatory mechanisms behind the Cd tolerance are not fully understood. Ferrochelatase-1 (FC1, EC4.99.1.1) is the terminal enzyme of heme biosynthesis, catalyzing insertion of ferrous ion into protoporphyrin IX. Recent studies have shown that *FC1* is involved in several physiological processes. However, its biological function associated with plant abiotic stress response is poorly understood.

**Results:**

In this study, we showed that *AtFC1* was transcriptionally activated by Cd exposure. *AtFC1* overexpression (*35S::FC1*) lines accumulated more Cd and non-protein thiol compounds than wild-type, and conferred plant tolerance to Cd stress, with improved primary root elongation, biomass and chlorophyll (Chl) content, and low degree of oxidation associated with reduced H_2_O_2_, O^·2-^ and peroxides. In contrast, the *AtFC1* loss of functional mutant *fc1* showed sensitivity to Cd stress. Exogenous provision of heme, the product of AtFC1, partially rescued the Cd-induced toxic phenotype of *fc1* mutants by improving the growth of seedlings, generation of glutathione (GSH) and phytochelatins (PCs), and GSH/PCs-synthesized gene expression (e.g. *GSH1*, *GSH2*, *PCS1*, and *PCS2*). To investigate the mechanism leading to the *AtFC1* regulating Cd stress response in Arabidopsis, a transcriptome of *fc1* mutant plants under Cd stress was profiled. Our data showed that disfunction of *AtFC1* led to 913 genes specifically up-regulated and 522 genes down-regulated in *fc1* mutants exposed to Cd. Some of the genes are involved in metal transporters, Cd-induced oxidative stress response, and detoxification.

**Conclusion:**

These results indicate that *AtFC1* would act as a positive regulator of plant tolerance to Cd stress. Our study will broaden our understanding of the role of *FC1* in mediating plant response to Cd stress and provide a basis for further exploration of its downstream genes.

**Electronic supplementary material:**

The online version of this article (10.1186/s12870-017-1141-0) contains supplementary material, which is available to authorized users.

## Background

Soil contamination with toxic metals such as cadmium (Cd), lead (Pb) and mercury (Hg) is a global environmental problem [1]. Cd is one of the naturally occurring toxic heavy metal present in the Earth’s crust and negatively affects the plant growth and development. Cd is readily absorbed by plants. Cd excess in plants can disrupt nutrient homeostasis, change physiological processes, and induce massively toxic reactive oxygen species (ROS) [2–4], all of which are associated with the damage of proteins, nucleic acids and lipids of plasma membrane [5–7]. More seriously, Cd accumulation in crops, particularly in edible parts, risks crop production and food safety [8].

Many plant species have developed diverse mechanisms to deal with the issues by Cd efflux, chelation, sequestration or detoxification [9, 10]. For example, most of brassicaceae species like India mustard, *Brasisca napus* or Arabidopsis generate phytochelatins, when challenged to environmental Cd [5, 11]. PCs are small cysteine-rich peptides with general structures of (γ-Glu-Cys)_n_-Gly (*n* = 2–7). The PCs biosynthesis is catalyzed by phytochelatin synthase (PCS, EC 2.3.2.15) with glutathione as a substrate, and has been long considered as metal detoxified chelatins because Cd can be compounded by the free thiol groups [12]. The PCs-Cd complex (2500–3600 Da) is across the tonoplasts and sequestered in vacuoles for Cd detoxification [13]. In Arabidopsis, PCS1 and PCS2 are two important components for the metal-dependent transpeptidation and mainly contribute plant tolerance to Cd toxicity [14, 15]. To date, the PCs biosynthetic pathway is thought of one of the most important mechanisms for plant tolerance to Cd stress. Some other Cd-inducible genes were also found to be involved in PCs-dependent pathway, such as *HsfA4a* [16], *HMT1* [17], *PAD2–1* and *VTC2–1* [18], ZAT6 [19]. For example, Chen et al. [20] reported a Cd-inducible gene *MAN3* (also called *XCD1* or XVE system-induced cadmium-tolerance 1), which is coding for an endo-β-mannanase. The Cd-induced *MAN3* increased the mannanase activity and the cell wall mannose content as well. Interestingly, *MAN3* overexpression enhanced Cd accumulation and tolerance through the GSH-dependent PCs synthetic pathway [20]. In addition, an ABC-type transporter *AtABCC3* was reported involving the Cd-responsive phytochelatin-mediated tolerant pathway [21].

Ferrochelatase-1 (FC1, EC4.99.1.1) is the terminal enzyme of heme biosynthesis, catalyzing insertion of ferrous iron into protoporphyrin IX (Proto IX) to generate protoheme [22–24]. Proto IX is the branch point of the tetrapyrrole pathway of heme and chlorophyll biosynthesis. While chlorophyll is known as an important pigment for light absorption, transfer and photosynthesis in plants, heme serves as a cofactor of many important proteins for diverse biological processes such as electron transfer, oxygen metabolism, and secondary metabolism [25]. FC1-produced heme is required for up-regulation of ROS scavenger genes for plant tolerance to abiotic stress [26–30]. FC-1 is highly conserved in green algae, plants and mammals, although the different number of the FC gene isoforms exists [31–33]. Soybean (*Glycine max*), poplar (*Populus trichocarpa*), and apple (*Malus domestica*) contain five or more FC gene members, whereas Arabidopsis have only two (*FC1*, AT5G26030) and (*FC2*, AT2G30390). In Arabidopsis, *AtFC1* and *AtFC2* have a distinct gene expression model. While *AtFC1* is expressed at all plant tissues including stems, flowers, leaves, and roots, *AtFC2* mainly expresses in photosynthetic tissues like shoots [22–24, 33, 34]. This suggests that *AtFC1* and *AtFC2* have different roles in various tissues. The differentially expressed *AtFC1* and *AtFC2* can be associated with the distinct genetic phenotypes in their functionally defective mutants. For example, *fc2* mutants display reduced chlorophyll and carotenoid abnormal phenotypes, while *fc1* mutants are hardly detected [33]. Promoter analyses show that *AtFC1* can be activated by norflurazon (an inhibitor of carotenoid biosynthesis) and viral infection, whereas *AtFC2* promoter activity was repressed under the same condition [26]. Furthermore, *AtFC1* is induced by a variety of biotic and environmental stresses such as drought, wounding and oxidative stress; in contrast, no such observations were found in *AtFC2* [33]. These findings indicate that *AtFC1* and *AtFC2* differently contribute to the biological functions.

To date, the majority of studies are focusing on identification of the essential role of *FC* in regulating plant growth, development and metabolism [33–35], and only a few of them concern *FC* in plant responses to abiotic stress [27, 36]. No reports are available for *FC* functional regulation of plant adaption to metal stress such as Cd-induced toxicity in plants. In this study, we reported a previously unknown function of *AtFC1* in response to Cd stress. We showed that *AtFC1* could be induced by Cd. Arabidopsis plants overexpressing *AtFC1* manifested enhanced Cd tolerance, while mutation of *AtFC1* led to more sensitivity of plants to Cd. *AtFC1* was found to activate genes involved in PCs production, suggesting that Cd-induced *AtFC1* was able to positively regulate Cd tolerance through the PCs synthesis-mediated pathway.

## Results

### Cd stress induced expression of *AtFC1* in Arabidopsis

**Fig. 1:**
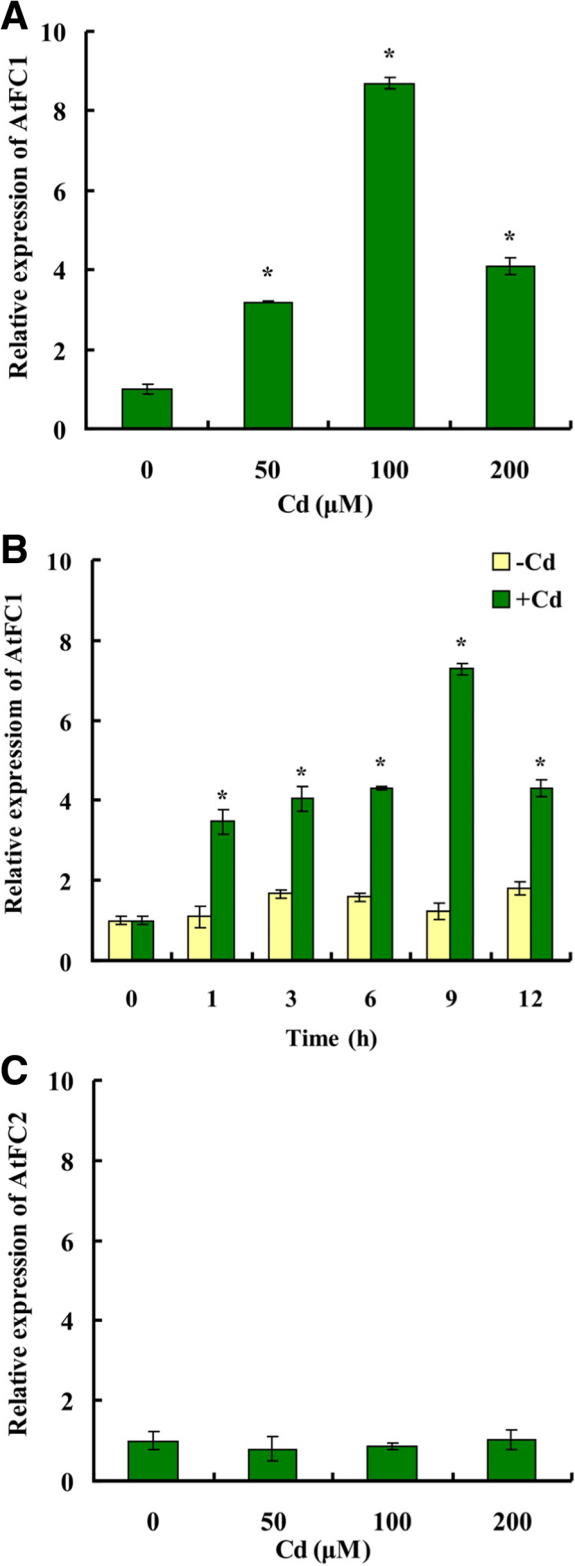
Expression pattern of *FC-1* and *FC-2* in Arabidopsis seedlings exposed to Cd. Three weed-old seedlings (wide-type) cultured in half-strength Hoagland solution were exposed to 0, 50, 100 and 200 μM Cd for 9 h (**a**, **c**) or to 100 μM Cd for 0, 1, 3, 6, 9 and 12 h (**b**). Following treatment, total RNA was extracted. Transcript levels were analyzed by qRT-PCR. Vertical bars represent standard deviation. Asterisks indicate that the mean values are significantly different between the Cd treatment (+Cd) and control (−Cd) (*p* < 0.05)

To ascertain whether *AtFC1* responses to Cd stress, the expression pattern of *AtFC1* in Arabidopsis wild-type (WT) was monitored under Cd stress by qRT-PCR. Expression of *AtFC1* was significantly induced by 50–200 μM Cd, while the maximal enhancement of *AtFC1* expression was found at 100 μM Cd (Fig. 1a). Cd-induced *AtFC1* expression was also found in a time-dependent manner, in which treatment with Cd at 100 μM enhanced 5.9-fold higher expression at 9 h than the control (Fig. 1b). In contrast, *AtFC2* failed to be induced by Cd exposure, even though the Cd concentration was up to 200 μM (Fig. 1c). This result is consistent with the conclusion that *FC2* appears not to respond to environmental stress [33, 34].

### Overexpression of *AtFC1* enhanced Cd tolerance in Arabidopsis

To identify the role of *AtFC1* in regulating plant response to Cd stress, we generated *AtFC1*-overexpressing transgenic lines of Arabidopsis. Full length of *AtFC1* sequence was fused to the cauliflower mosaic virus 35S promoter and transformed into the Col-0 ecotype background. The *35S::FC1* transgenic lines used in this study were screened and identified. The transgenic plants carrying *35S::AtFC1* showed 13.5 to 22.8-fold higher transcripts of *AtFC1* than the wild-type plants (Additional file 1: Data S1). We further used a T-DNA insertion mutant of *AtFC1* (SALK-150001.42.45×, *fc1*) from the Arabidopsis Biological Resource Center. The *fc1* mutant was verified by diagnostic PCR using gene-specific primers and was found with a T-DNA insertion in the 5′-untranslated regions; qRT-PCR analysis showed that the *AtFC1* transcript levels in *fc1* mutant plants was only 29% of those in wild type (Additional file 1: Data S2).

**Fig. 2:**
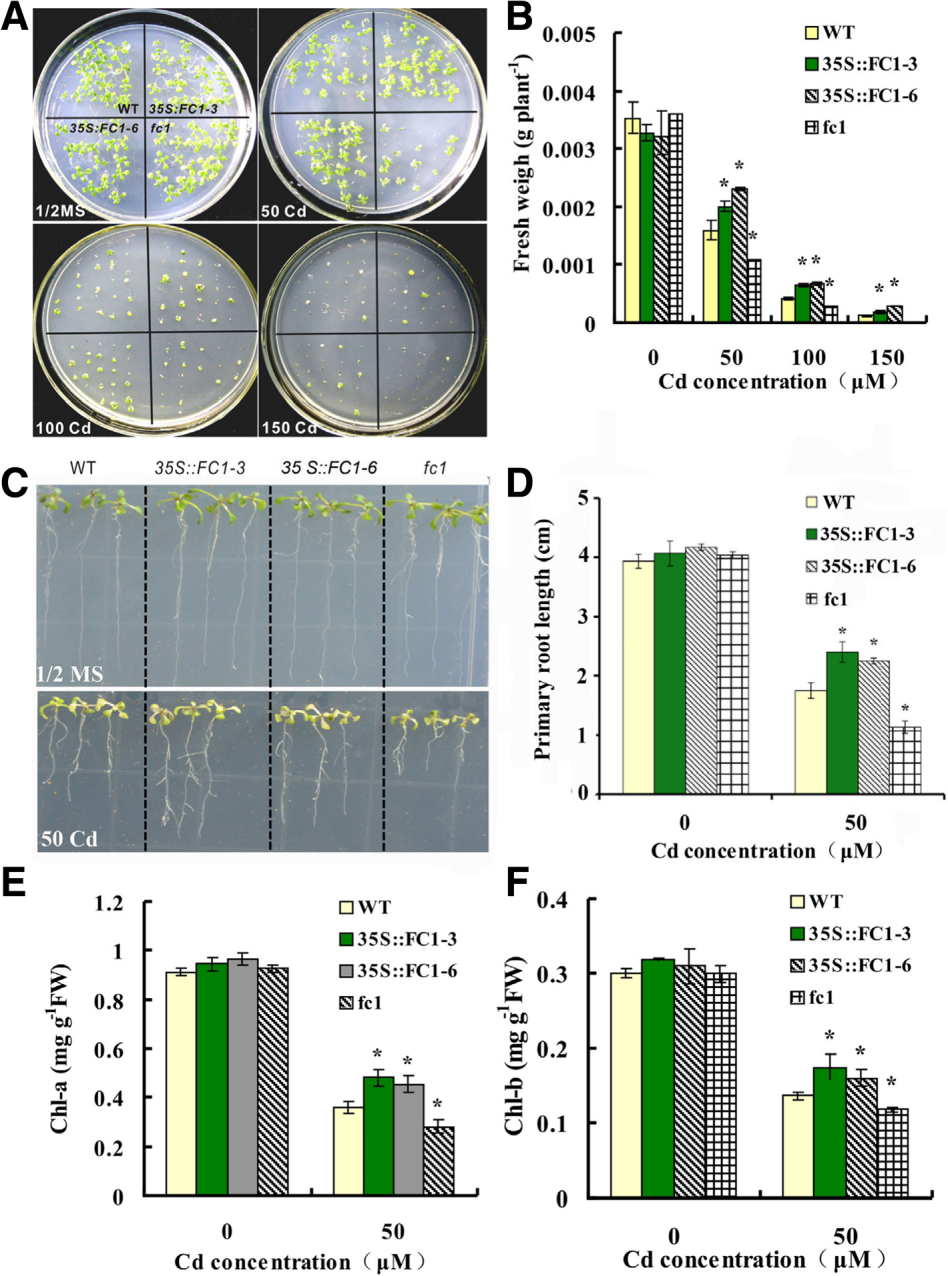
Analysis of cadmium (Cd) tolerance in the wild-type (WT), *AtFC1* transgenic lines (*35S::FC1*) and *fc1* mutants of Arabidopsis. **a**, **b**: Phenotype and fresh weigh of seedlings grown on 1/2 MS medium with or without 50, 100 or 150 μM Cd for 12 d. **c**, **d**: Root length of *AtFC1* transgenic seedlings and *fc1* mutants grown on 1/2 MS medium with or without 50 μM Cd for 12 d. E and F: The chlorophyll-a and chlorophyll-b contents of *AtFC1* transgenic seedlings and *fc1* mutants. Seedlings were grown hydroponically for 21 d and transferred to the same culture solution with and without 50 μM Cd for 5 d. Vertical bars represent standard deviation. Asterisks indicate that the mean values are significantly different between the transgenic plants/mutants and wild-type plants (*p* < 0.05)

Seeds of WT, *35S::FC1*, and *fc1* were germinated and grown on the half strength MS agar plates with Cd (0, 50, 100 and 150 μM) for 12 d. In non-Cd medium, no significant differences of biomass within the WT, *35S::FC1*, and *fc1* mutant plants were observed (Fig. 2a). When seedlings were exposed to 50–150 μM Cd, *35S::FC1* plants had increased fresh biomass, whereas *fc1* mutants had reduced biomass, as compared to WT (Fig. 2b). A similar result was observed for dried biomass (Additional file 1: Data S3). We then examined the primary root elongation of seedlings under Cd stress. *35S::FC1* and WT seeds were placed on the solid 1/2 MS medium supplemented with 50 μM Cd for 12 d. *35S::FC1* plants showed strong root growth over the wild-type (Fig. 2c). Under Cd exposure, the elongation of *35S::FC1* roots was 1.2–1.4 fold higher than that of wild-type; in contrast, the root elongation of *fc1* mutants was weak compared to the wild-type (Fig. 2d). Chlorophyll is sensitive to heavy metals and often used as a biomarker of metal stress [37]. Compared to WT, the levels of Chl-a and Chl-b were not affected in *35S::FC1* or *fc1* mutant in non-Cd medium (Fig. 2e, F). Under 50 μM Cd stress, the concentrations of Chl-a and Chl-b increased in *35S::FC1* plants, but decreased in *fc1* mutants. These results showed that *AtFC1* was able to improve the plant tolerance to Cd stress.

### Overexpression of *AtFC1* improved antioxidation capacity in plants

**Fig. 3:**
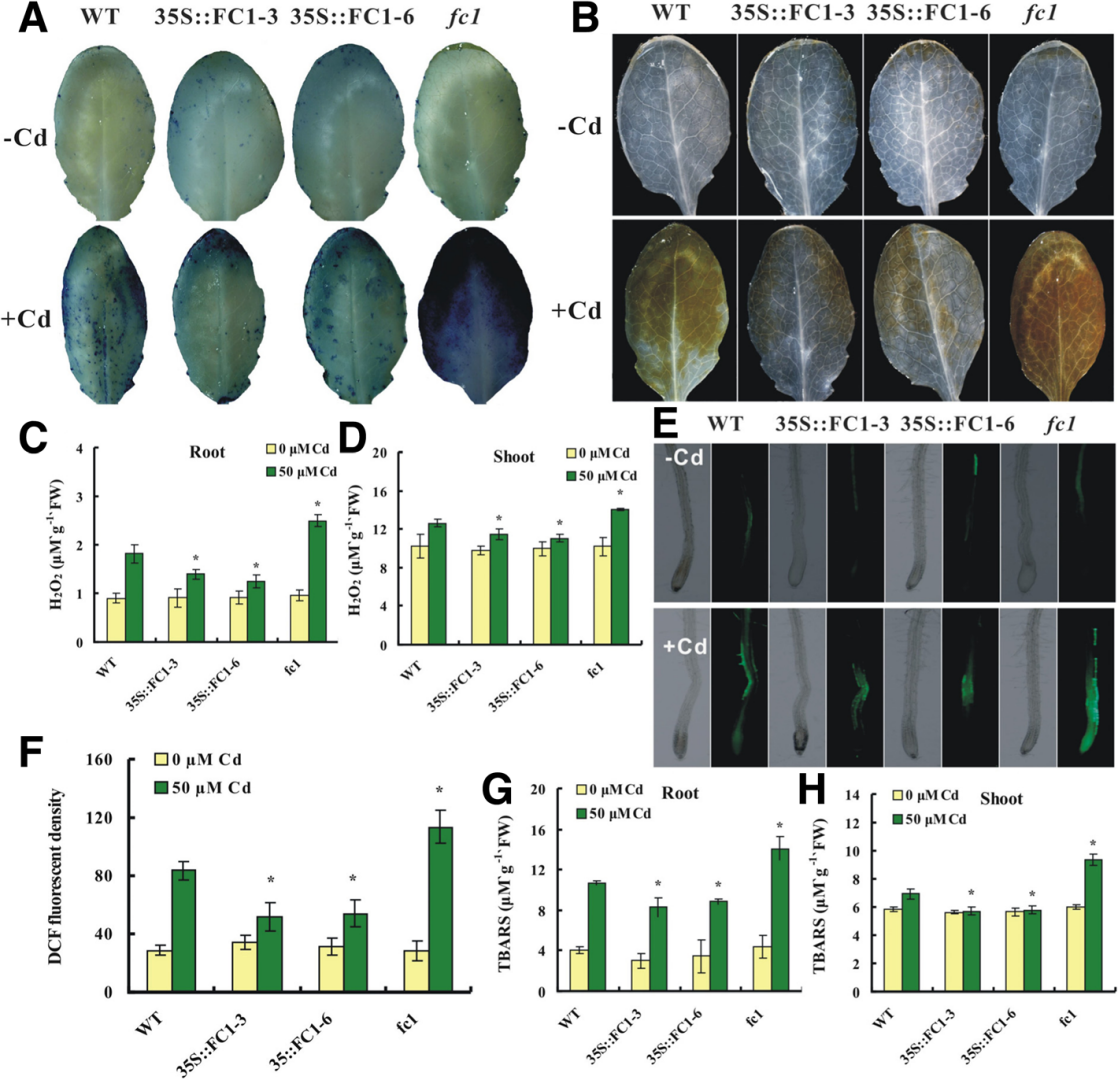
Accumulation of O_2_^−^, H_2_O_2_ and other oxidants in the wild-type (WT), *AtFC1* transgenic lines (*35S::FC1*) and *fc1* mutants of Arabidopsis under Cd stress. Seedlings were grown in the half-strength Hoagland solution for 21 d and transferred to the same culture solution with 50 μM Cd for 1 d. **a**: Histochemical detection of O_2_^•−^ on leaves by NBT staining. **b**: Histochemical detection of H_2_O_2_ on leaves by DAB staining. **c**, **d**: H_2_O_2_ contents in roots and shoots. **e**: DCFH-DA fluorescent imaging for endogenous ROS. **f**: Quantitative analysis of fluorescent density of image of (**e**). **g**, **h**: TBARS contents in roots and shoots. Vertical bars represent standard deviation. Asterisks indicate that the mean values are significantly different between the transgenic plants/mutants and WT (*p* < 0.05)

It is well known that Cd can induce oxidative stress in plants [6, 38]. To examine whether the transgenic plants have an antioxidative capability, we performed the histochemical staining with nitroblue tetrazolium (NBT), by which O_2_^-.^ And H_2_O_2_ were visualized in a dark blue by insoluble formazan compound and in a brown by 3,3-diaminobenzidine (DAB) reagent, respectively. Compared to WT, plant exposure to 50 μM Cd led to dark staining with NBT (Fig. 3a) and DAB (Fig. 3b) in the leaves of *fc1* mutant, while in *35S::FC1* leaves, the staining was relatively light. The *AtFC1*-regulated decrease of H_2_O_2_ in plant tissues was proved by the quantitative analysis (Fig. 3c, d).

Both O_2_^-.^ and H_2_O_2_ as ROS can damage the plasma membrane and other biomolecules, An in situ detection of endogenous ROS in roots was further performed by the specific fluorescent probe DCFH-DA (2′, 7′-dichlorofluoresce indiacetate) [39]. Treatment with 50 μM Cd for 30 min increased the DCF (dichlorofluoresce) fluorescent density in WT (Fig. 3e, f). A relatively lower level of DCF fluorescence was detected in the Cd-exposed *35S::FC1* plants. Conversely, the *fc1* mutant generated much more DCF fluorescence under the same condition. TBARS (thiobarbituric acid reactive substances) as a biomarker represents a degree of damage of cellular plasma membrane [39]. All these oxidative products were quantified in plants under Cd stress. Addition of 50 μM Cd into media led to a significant increase in TBARS content in both shoot and root of WT plants (Fig. 3g, h). Transgenic plants overexpressing *AtFC1* showed a lower level of TBARS than WT plants. In *fc1* mutant plants the TBARS content was always higher. These results suggest that overexpression of *AtFC1* contributed to the antioxidation capacity by repressing the generation of O_2_^-.^ and H_2_O_2_ in plants.

### Overexpression of *FC1* improved non-protein thoils production and Cd accumulation in plants

**Fig. 4:**
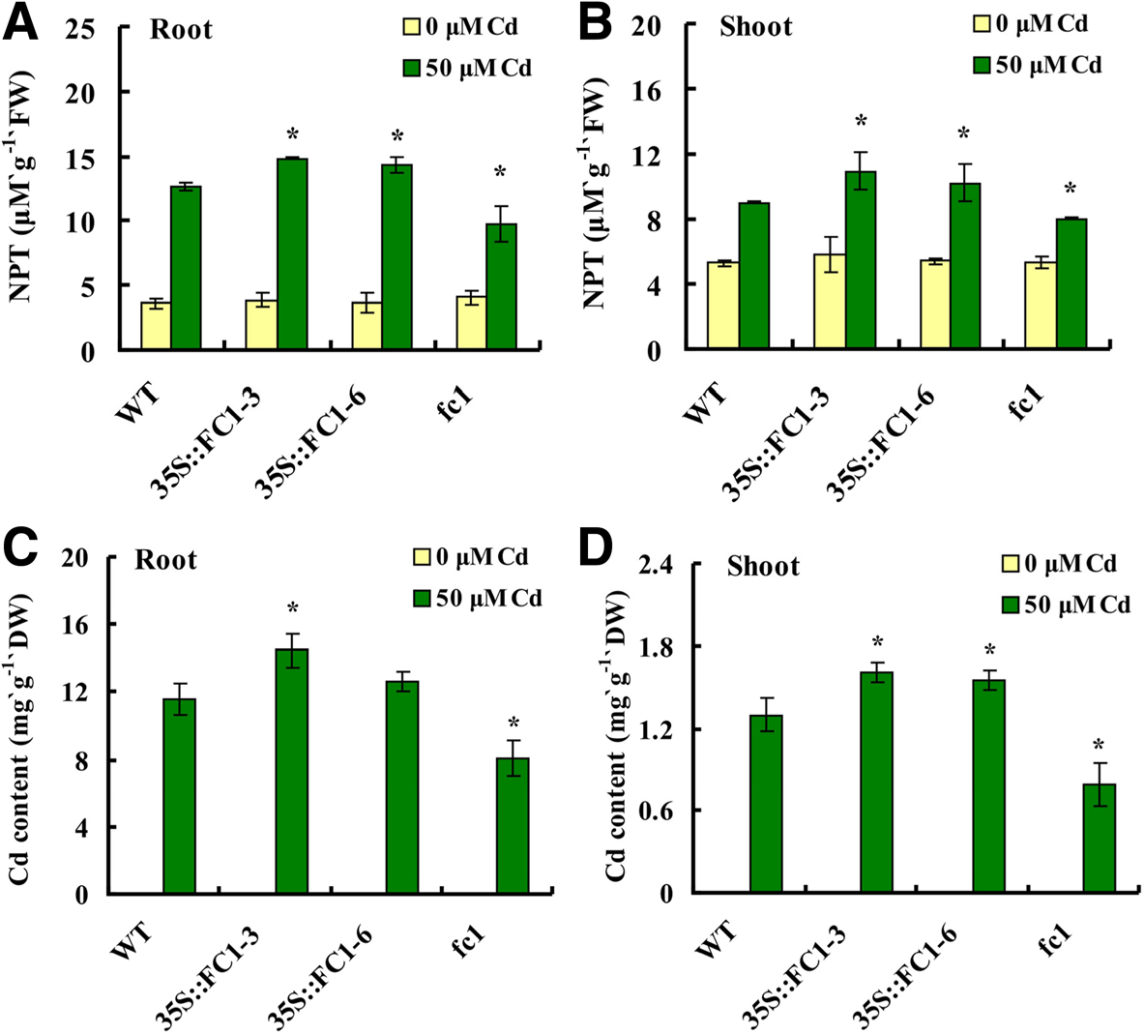
NPT and Cd concentrations in shoots and roots of WT, (*35S::FC1*) and *fc1* mutant plants. Seedlings were grown in the half-strength Hoagland solution for 21 d and transferred to the same culture solution with 50 μM Cd for 5 d. **a**, **b**: NPT contents. **c**, **d**: Cd contents. Vertical bars represent standard deviation. Asterisks indicate that the mean values are significantly different between the transgenic plants/mutants and WT (*p* < 0.05)

The non-protein thoils (NPT) compounds, consisting of several acid-soluble sulfhydryl components such as cysteine, γ-glutamyl-cysteine, GSH and PC, are able to chelate toxic metals for plant detoxification [9]. Under the normal condition, there were no differences of NPT concentrations in WT, *35S::FC1* or *fc1* mutant plants; when challenged to 50 μM Cd, *35S::FC1* plants accumulated more NPT in roots and shoots, whereas a lower level of NPT in *fc1* mutants was detected (Fig. 4a, b). Similarly, *35S::FC1* plants accumulated more Cd than WT, while *fc1* mutant plants accumulated less Cd, compared to WT (Fig. 4c, d).

### *AtFC1* improved Cd tolerance through PCs-dependent pathway

**Fig. 5:**
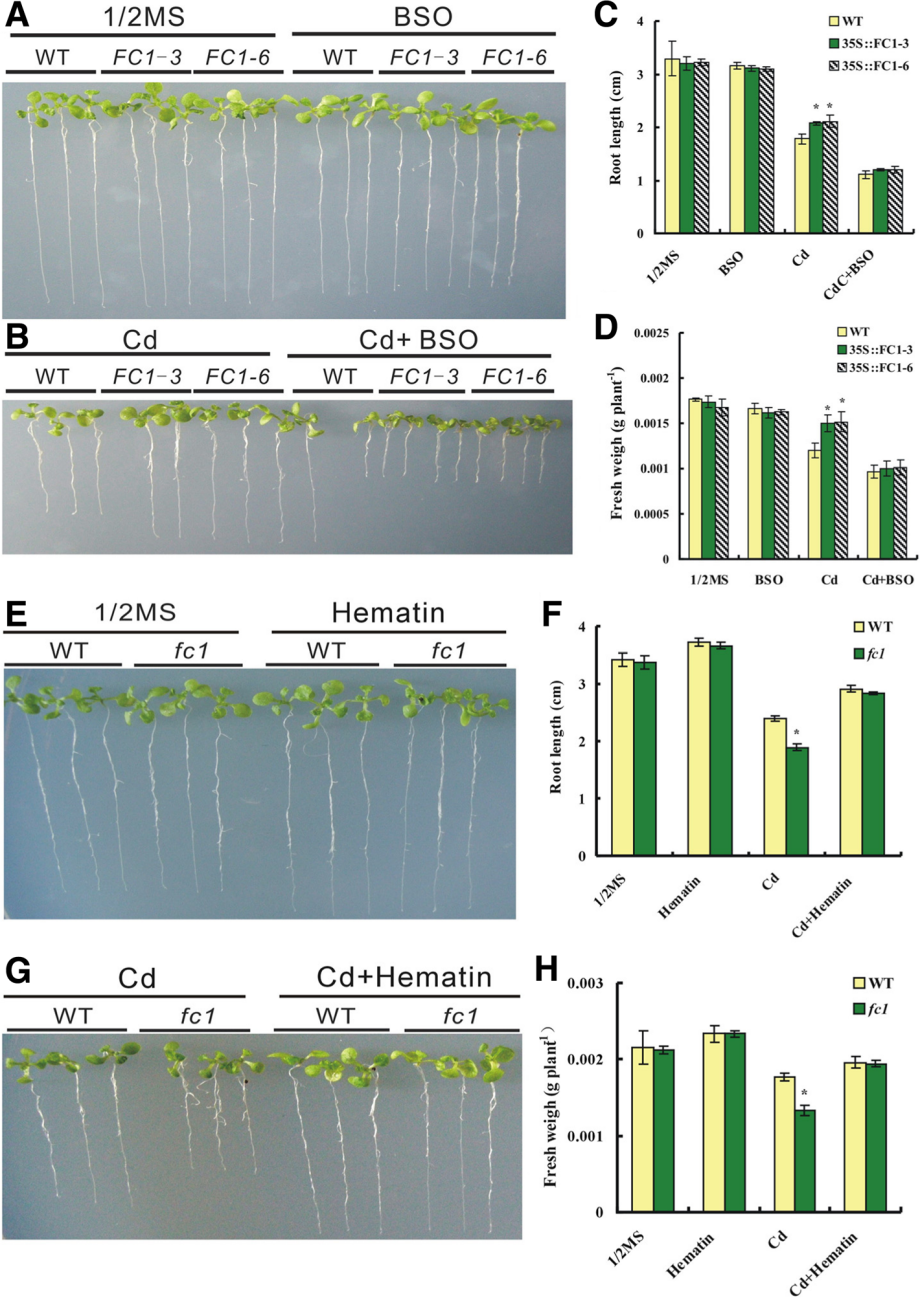
Effects of buthionine sulfoximine (BSO) with combination of hematin on the growth of seedlings of the wild-type (WT), *35S::FC1* lines and *fc1* mutants under Cd stress. Seedlings were grown on 1/2 MS media with 50 μM Cd, 100 μM BSO or 10 μM hematin for 12 d. (A, B): Phenotypes of seedlings growing with BSO under -Cd (A, 1/2 MS, control) and +Cd (B) stress. (C, D): Root elongation and fresh weight of seedlings corresponding to (**a**) and (**b**), respectively. **e**, **f**, **g**: Root elongation. **h**: Fresh weight corresponding to (**e**) and (**g**), respectively. Vertical bars represent standard deviation. Asterisks indicate that the mean values are significantly different between *35S::FC1* lines/*fc1* mutants and WT (*p* < 0.05)

**Fig. 6:**
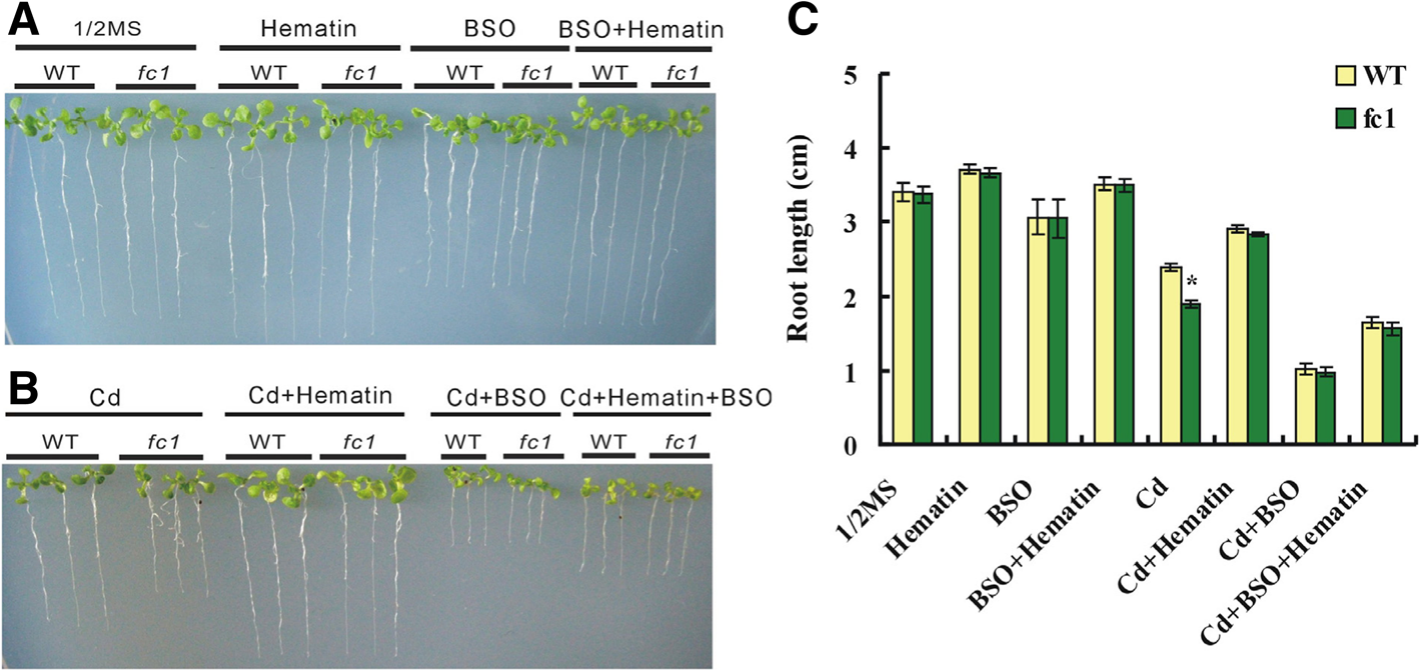
Effect of buthionine sulfoximine (BSO) with combination of hematin on growth of seedlings of the wild-type (WT) and *fc1* mutants under Cd stress. Seedlings were grown on 1/2 MS media with 50 μM Cd, 100 μM BSO or 10 μM hematin for 12 d. (A, B): Phenotypes of WT and *fc1* seedlings growing with BSO and/or Hematin under -Cd (A, 1/2 MS, control) and –Cd stress (B). (C): Quantitative analysis of root elongation corresponding to (**a**) and (**b**). Vertical bars represent standard deviation. Asterisks indicate that the mean values are significantly different between the *fc1* and WT plants (*p* < 0.05)

Buthionine sulfoximine (BSO) is a GSH synthetic inhibitor and was employed to investigate the involvement of GSH-dependent PCs pathway in *AtFC1*-regulated Cd response in Arabidopsis. The *35S::FC1* plants showed enhanced root growth and biomass under Cd stress compared to WT, but the enhanced-root growth could be blocked by BSO (Fig. 5a-d), suggesting that *AtFC1*-improved root growth was mediated by PCs-dependent detoxifying pathway. We then used a heme (the product of AtFC1) substitute, hematin (protoheme, with the same function and high stability) [28, 40], to examine the effect of hematin in *fc1* mutants. In the absence of Cd, no difference of root elongation was observed between WT and *fc1* plants; treatment with 50 μM Cd reduced the root elongation of *fc1* mutants relative to WT (Fig. 5e-g). Supplying 10 μM hematin could largely rescue the root elongation and biomass of *fc1* mutants under Cd stress (Fig. 5e-h). Supplying hematin also enhanced the GSH, PCs and NPT levels in the roots and shoots of wild-type under Cd stress (Additional file 1: Data S4). A combinational effect of BSO and hematin on the root growth was monitored. Under the normal condition, the roots of WT and *fc1* mutant seedlings showed no significant difference in the presence of BSO or in combination with hematin (Fig. 6a). Under Cd exposure, however, the positive effect of hematin on root elongation of WT and *fc1* mutants was diminished by BSO (Fig. 6b, c). A similar result was found for the fresh weight of the seedlings under the same condition (Additional file 1: Data S5).

### *AtFC1-*regulated genes were related to the GSH-dependent PC synthesis pathway

**Fig. 7:**
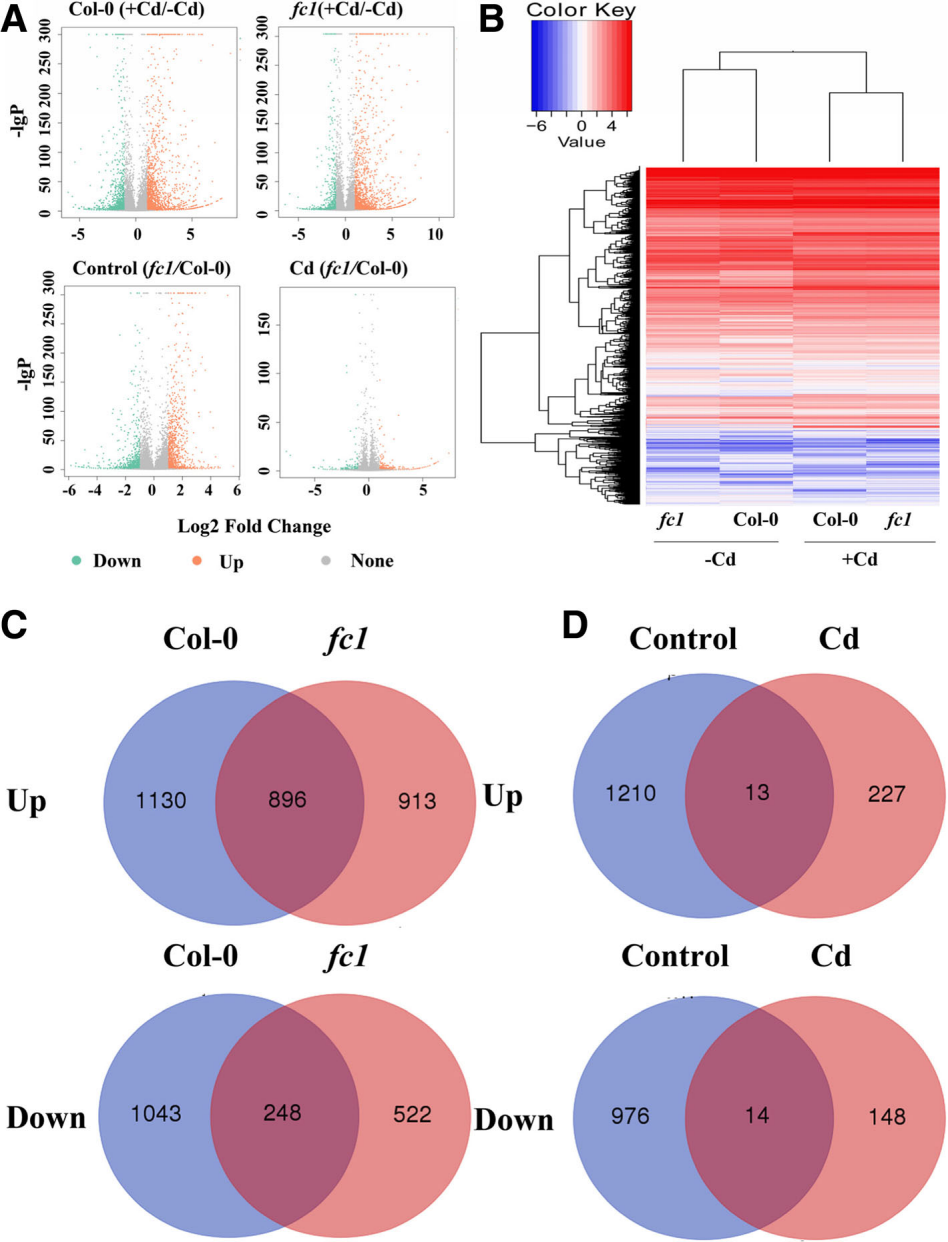
Differentially expressed genes in the wild-type (Col-0) and *fc1* mutants of Arabidopsis under Cd stress. Two week-old Arabidopsis seedlings were exposed to 0 (−Cd) and 50 μM Cd (+Cd) in the half-strength Hoagland solution. **a**: Distribution and expression levels of DEGs in WT and *fc1* mutant seedlings with or without Cd exposure. The *x* axis represents the log_2_ fold change under the mean normalized expression of all transcripts (*y* axis). Green dots indicate the down-regulated genes, and red dots show the up-regulated genes. **b**: Heatmap of a one-dimensional hierarchical clustering of differential gene expression as determined by mRNA-seq for the Cd-exposed (+Cd) seedlings (WT and *fc1*) relative to the control (−Cd). **c**: Venn diagram showing up- and down-regulated genes in Col-0 and *fc1* mutant seedlings under Cd stress. **d** Venn diagram showing up- and down-regulated genes in *fc1* seedlings relative to Col-0 grown under the control (–Cd) and Cd-exposed (+Cd) condition.

Because overexpression or knock-down of *AtFC1* led to contrasting phenotypes of plants under Cd stress, we assumed that many Cd-responsive genes including Cd tolerance and detoxifying genes were likely involved in the process. To address the question, we created four libraries with *fc1* mutant and wild-type plants under –Cd and +Cd stresses, and employed the Illumina RNA-sequencing platform (HiSeq 2000), which allows to sequence transcripts in a high-throughput manner, to identify the global transcriptome of *fc1* mutant and wild-type plants. A total of 29.9–31.6 million clean reads were generated from four libraries (Additional file 1: Data S6). Mapping the reads to Arabidopsis genome led to identification of 3317 (2026 up and 1291 down) and 2579 (1809 up and 770 down) unique genes in wild-type and *fc1* mutant plants (> two fold change, *p*< 0.05) under Cd stress, respectively (Additional file 1: Data S7–10). The distribution of differentially expressed genes was shown by color dots (blue, down, and red, up) (Fig. 7a). The *fc1* mutants under Cd stress displayed more red dots (differentially expressed genes, DEGs up-regulated) and less blue dots (DEGs down-regulated) than the wild-type (Col-0) plants. Also, under the control (−Cd) and Cd stress (+Cd), more red dots than blue dots were found in the *fcl*/Col-0 group, indicating that more genes were induced in the *fc1* mutants than those in the wide-type (Fig. 7a). These results were also presented by the heat-map graph (Fig. 7b). The number of the DEGs was further detailed by plotting Venn diagrams. There were 1130 genes that were specifically upregulated and 1043 downregulated in wild-type (Col-0) under Cd stress; also, 913 specifically upregulated genes and 522 repressed genes were found in the *fc1* mutant plants (Fig. 7c). Comparative analysis of DEGs between *fc1* mutant and wild-type plants revealed that 1210 genes were specifically induced and 976 genes were repressed under the normal condition, whereas 227 genes were specifically induced and 148 genes were repressed under Cd stress (Fig. 7d).

**Fig. 8:**
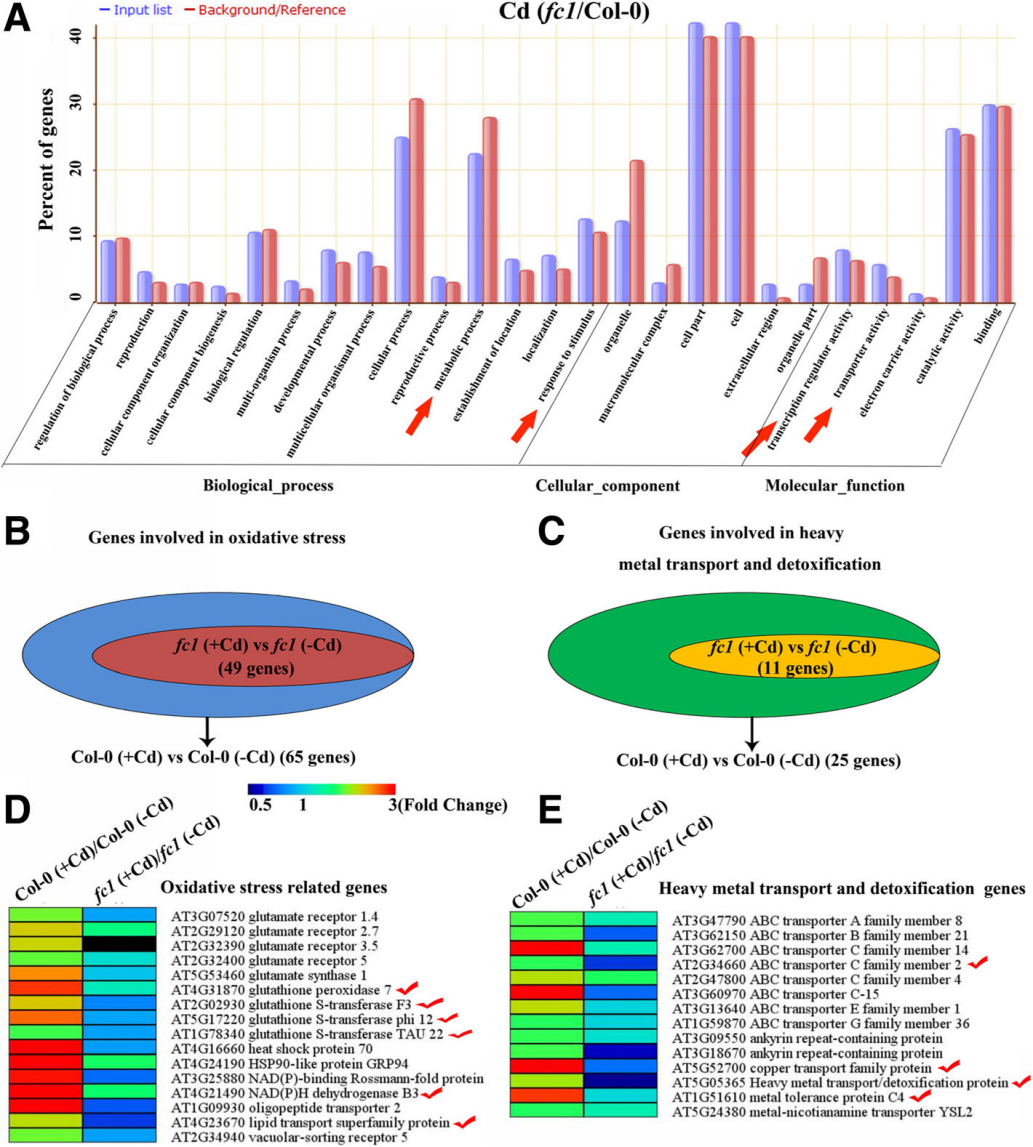
The mRNA-seq based differentially expressed genes for the Cd-exposed *fc1* mutants relative to the Cd-exposed Col-0 wild-type. Two week-old Arabidopsis seedlings were exposed to 0 (−Cd) and 50 μM Cd (+Cd) in the half-strength Hoagland solution. **a**: GO analysis of specific genes from the 227 up-regulated and 148 down-regulated genes (Figure 8d) in *fc1* mutants relative to the wild-type under Cd stress. Genes most related to Cd tolerance are noted with red arrows. **b**, **c**: Venn diagrams displaying the genes differentially expressed in Col-0 (+Cd)/Col-0 (−Cd) and *fcl* (+Cd)/*fcl* (−Cd) under Cd stress. The presented genes are functionally involved in Cd-induced oxidative stress (**b**) and Cd transport or detoxification (**c**). The small ellipses in the big ellipses represent the overlap of the genes between Col-0 and *fc1* mutant plants. **d**, **e**: Heatmap represents the gene expression levels of individual reactive oxygen species-related genes (**d**) and heavy metal transport and detoxification genes (**e**). Some of the genes most related to Cd tolerance were highlighted by red tick off

The differentially expressed genes from *fc1*/Col-0 category following Cd exposure could be functionally classified using Gene Ontology. Based on the functional annotation, all genes can be divided into three major groups including biological process, cellular component and molecular function (Fig. 8a). Several pathways such as metabolic process, response to stimulus, transcription regulator activity and transporter activity were examined. As an example, genes involved in oxidative stress and heavy metal transport/detoxification were presented. The differentially expressed genes from the two combined library groups including Col-0 (+Cd)/Col-0 (−Cd) and *fc1* (+Cd)/*fc1* (−Cd) were selected for analysis (Fig. 8b, c). For oxidative stress-responsive DEGs, there were 65 genes from each library group. Of these, 49 genes overlapping in the two library groups showed a similar expression pattern. The remaining 16 genes expressed differentially between the two library groups. As shown in Fig. 8d, genes in the Col-0 (+Cd)/Col-0 (−Cd) group were upregulated, whereas those in the *fc1* (+Cd)/*fc1* (−Cd) group were downregulated under Cd exposure, indicating that the expression of the genes was impaired in *fc1* mutant plants. Several genes encoding glutathione peroxidase, glutathione S-transferases, NAD(P)H dehydrogenase and lipid transport superfamily protein were examined. Some of the genes such as glutamate synthase, glutathione receptor and glutathione peroxidase are related to GSH synthesis, while others like glutathione S-transferases are responsible for glutathione transport and PCs synthesis 12 [12]. Similarly, 14 genes encoding metal transporters were found to be repressed in the *fc1* (+Cd)/*fc1* (−Cd) group under Cd exposure (Fig. 8c). Most of the genes such as those encoding ABC transporter C, heavy metal transport/detoxification protein, metal tolerance protein and copper transporter are responsible for functional metal transport and detoxification (Fig. 8e). These results indicated that disruption of *AtFC1* expression altered the transcription of the genes responsible for Cd detoxification.

**Fig. 9:**
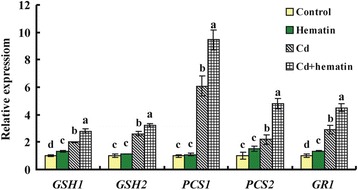
Transcript levels of genes related to PC synthesis in the wild-type under Cd exposure in the absence or presence of hematin. Seedlings were grown in the half-strength Hoagland solution for 21 d and transferred to the same culture solution without or with 50 μM Cd in the presence of 10 μM Hematin for 1 d. Vertical bars represent standard deviation. Asterisks indicate that mean values are significantly different between the treatments and control (*p* < 0.05)

Because AtFC1-produced heme contributed to the Cd detoxification in plants, we further examined and confirmed the expression of genes involved in GSH and PCs synthesis in wild-type plants under Cd stress with or without Hematin by qRT-PCR. *GSH1* encodes a γ-glutamyl-Cys synthetase (γ-ECS), which is the key enzyme limiting the synthesis of GSH in plants, and *GSH2* encodes a glutathione synthetase [12, 41]. Both *PCS1* and *PCS2* encode a phytochelatin synthase in Arabidopsis [9]. Cd exposure significantly induced the expression of *GSH1*, *GSH2*, *PCS1*, *PCS2* and *GR1* (Fig. 9). Compared to the Cd treatment alone, supplying hematin enhanced the expression of the four genes. Glutathione reductase-1 (GR1) catalyzes the reduction of oxidized GSH and is one of the key enzymes involved in modulation of redox homeostasis in Cd-tress plants [42, 43]. In this study, we also found that expression *GR1* was induced by Cd + hematin over the Cd treatment alone (Fig. 9).

## Discussion

Although several genes that mediate Cd accumulation and detoxification through glutathione and phytochelatins pathway have been identified [4, 20, 44, 45], the mechanisms for the molecular regulation and genetic basis for the physiological process are not fully understood. The present study characterized a role of *FC1* in Arabidopsis by exploring the effects of gain- and loss-of-function of the gene on the plant response to Cd exposure. The transcription of *AtFC1* was sufficiently induced by Cd. This observation is consistent to the previous results that *AtFC1* could be induced by other abiotic stresses [26, 27, 29]. Our result also supports the observation that *AtFC1* is responsible for heme and hemeproteins involved in the defense responses [26, 27, 29].

To illustrate that *AtFC1* was able to regulate plant response to Cd stress, transgenic Arabidopsis overexpressing *AtFC1* was generated. The *AtFC1* loss of function mutant *fc1* plants was also identified in the study. The *35S::FC1* plants displayed high tolerance to Cd, with improved root elongation, biomass of seedlings, chlorophyll accumulation, and non-protein thiol compounds, but showed a low degree of oxidation with reduced ROS (e.g. hydrogen peroxide and superoxide anion) and lipid peroxides. Conversely, *fc1* mutants displayed a phenotype sensitive to Cd stress. These data indicated that *AtFC1* was able to mediate the Cd stress response in Arabidopsis. To get an insight into the mechanism leading to *AtFC1*-regulated detoxification of Cd in plants, we profiled transcriptome data from *fc1* mutants under Cd stress. A large number of specific genes (522 genes) were found to be repressed in the *fc1* mutants, suggesting that the requirement for *AtFC1* is at least part of the Cd stress response. The Gene Ontology analysis revealed several pathways closely related to Cd stress response. Of these, genes involved in oxidative stress response and metal transport and detoxification should be paid more attention. The Cd-induced oxidative stress responsive genes contained several genes involved in GSH and PCs synthesis [22].

The GSH-dependent PCs synthetic pathway has been so far one of the most important mechanisms for metal tolerance in higher plants because PCs are prone to chelating and detoxifying toxic heavy metals like cadmium [4, 9]. Under Cd stress, some plant species can form Cd-GSH and Cd-PCs complexes to sequester Cd within vacuoles [3, 46] or disperse Cd through xylem and phloem vessels effectively [11]. Overexpression of *GSH* in Indian mustard and poplar enhanced Cd tolerance owning to the higher capacity of GSH and PCs synthesis [47–49]. Coexpression of *PCS1* and *GSH1* in Arabidopsis showed coordinated regulation of Cd tolerance and PCs production [44]. Thus, PCs play a vital role in heavy metal detoxification in plants [5]. The present study showed that *AtFC1* overexpression increased accumulation of total NPTs (including GSH and PCs), and the increased accumulation of NPTs was associated with enhanced Cd accumulation and tolerance in the transgenic plants. This process was correlated with the activation of genes involved in PCs synthesis, suggesting that *AtFC1* was one of the important regulators in the GSH-dependent PCs synthetic pathway contributing to Cd accumulation and tolerance. While the increased Cd accumulation was displayed in *AtFC1* transgenic plants, a lower level of Cd in *fc1* mutant plants was observed. This could be the result of weak generation of heme in *fc1* mutants. AtFC1-produced heme is one of the most important tetrapyrroles in plant cells because it is a cofactor essential for many important enzymes, proteins and transporters involved in many biological activities including oxygen metabolism, metabolite transport, electron transfer, biotic and abiotic stress responses [25]. In this regard, a shortage of heme due to the loss of function of *AtFC1* might directly or indirectly to affect some metal transporters in a negative way, which most likely impairs the uptake of Cd into plants.

Our analysis showed that pre-treatment with heme conferred plant tolerance to Cd stress. This observation can be implicated in the mechanisms for heme regulating Cd detoxification through GSH-dependent PCs synthetic pathway. Firstly, application of hematin increased the concentrations of GSH and PCs and as a consequence, the Cd tolerance in WT and *fc1* mutant plants was enhanced. Secondly, the hematin-promoted accumulation of GSH and PCs coincided with the upregulation of the expression of genes (e.g. *GSH1*, *PCS1*, and *PCS2*) involved in GSH and PCs synthesis. Finally, the positive effect of hematin was cancelled by the GSH synthesis inhibitor BSO. The site-directed mutagenesis study suggests that Thr 49 in PCS1 can be phosphorylated by casein kinase 2, which in turn increases the activity of PCS1 [50]. Casein kinase 2 was suggested to involve heme-regulated protein synthesis in mammalian cells [51]. In this case, heme may be directly or indirectly involved in promoting PC synthesis by enhance PCS1 activity through modulating its phosphorylation state. Whether heme is involved in past-modification of PCS1 activity needs further investigation.

Oxidative stress is triggered by over-generation of ROS, an observation that occurs in plants under Cd stress [45]. Plant cells possess two intrinsic systems to remove excessive ROS as a result of toxic metal exposure. The first one is anti-oxidative enzymatic system including superoxide dismutase, ascorbate peroxidase, glutathione reductase and catalase; and the second antioxidant system includes GSH and ascorbate [37]. The protective role of FC1 against photodynamically induced oxidative stress has been proposed in Arabidopsis [27]. Therefore, the anti-oxidative role of *AtFC1* in detoxifying Cd would at least partially attribute to the regulation of both anti-oxidants and anti-oxidative enzymes, which could be supported by several lines of evidence from this study and others: (1): *35S::FC1* plants accumulated less ROS, reflecting a high capacity of *AtFC1* transgenic plants, in which the Cd-induced ROS generation was effectively scavenged by anti-oxidants and anti-oxidative enzymes; (2) *AtFC1-*generated heme positively regulated the GSH synthesis by coordinately activating the expression of *GSH1*. GR is an important enzyme involved in the regulation of GSH-AsA cycle for maintaining redox homeostasis in plants upon metal exposure [52]. Hematin was shown to induce the expression of *GR1* in Cd-treated Arabidopsis, suggesting that heme may maintain the redox homeostasis by regulating *GR1* in plants in response to Cd stress; and (3) heme can be utilized as a redox cofactor to enhance the activity of many so-called heme-proteins. Several heme-proteins such as ascorbate peroxidase and catalase are involved in response to oxidative stress [53]. Ectopic expression of a *Bradyrhizobium japonicum FC* in rice protected rice from photodynamically induced oxidative stress by enhancing the activity of heme-CAT and heme-APX [36]. These results suggest that FC1 would contribute to plant tolerance to Cd through antioxidative mechanism.

## Conclusion

Our physiological and genetic evidence showed that *AtFC1* was transcriptionally induced by Cd exposure. *AtFC1* overexpression conferred plant tolerance to Cd stress by enhancing primary root growth, biomass and chlorophyll concentration, but reducing accumulation of reactive oxygen species such as H_2_O_2_, O^·2-^, and TBARS in tissues. The Cd-induced *AtFC1* and *AtFC1*-generated heme were able to contribute to the plant tolerance to Cd by activating the genes responsible for the GSH-dependent phytochelatin synthetic pathway. Thus, our study would broaden our understanding of the role of *FC1* in mediating plant response to Cd stress and provide a basis for further exploration of its downstream genes.

## Methods

### Plant growth condition and treatments

Wild-type seeds of *Arabidopsis thaliana* (ecotype Col) and the *fc1* mutant (SALK_15000.142.45.X) were ordered from the Arabidopsis Biological Resource Center, Ohio, USA. Agrobacterium (EHa105) were ordered from BaiSi Biotechnology company (Hangzhou, China). Wild-type seeds, the *fc1* mutant (AT5G26030) and transgenic seeds (T4, homozygote, Col back-ground) were surface-sterilized and plated on half-strength Murashige and Skoog (MS) solid media supplemented with 1% sucrose and 0.8% agar (pH 5.8). The plates were stored for 3 d in darkness at 4 °C and transferred to growth chamber with the conditions of 100 μE m^−2^ s^−1^, 16/8 h light/dark cycle, and 22 °C. For Cd treatment experiments, seeds of WT, the *fc1* mutants or transgenic seed were germinated and grown on half-strength Hoagland nutrient solution with Cd (pH 6.0), buthionine sulfoximine (BSO; Sigma) or other reagents. Concentrations of Cd were set as 0, 50, 100 and 200 μM for dose-response experiment or 100 μM for a time-dependent (0, 1, 3, 6, 9 and 12 h) experiment. The seedlings were grown in a growth chamber with the same condition described above. The growth solution was changed every two days. After treatments plants were sampled and immediately frozen by liquid nitrogen for following experiments.

### Transcript analysis by RT-PCR

Total RNA was extracted from tissues using TRIzol (Invitrogen) and 1.0 μg of RNA was used as a template for cDNA synthesis. A 1% agarose gel, stained by ethidium bromide, was run to check the integrity of the RNA. All RNA samples were quantified and examined for protein contamination (A260 nm/A280 nm ratios) and reagent contamination (A260 nm/A230 nm ratios) by a Nanodrop ND 1000 spectrophotometer.

The first strand cDNA was synthesized from 1.0 μg total RNA by Moloney Murine Leukemia Virus Reverse Transcriptase (Promega) using oligo(dT) primers. The quantitative RT-PCR (qRT-PCR) was performed with a MyiQ Single Color Real-time PCR system (Bio-Rad) in a final volume of 20 μL containing 2 μL of a 1/10 dilution of cDNA in water, 10 μL of the 2 × SYBR Premix Ex Taq (TaKaRa) and 200 nM of forward and reverse primers. qRT-PCR was conducted using a CFX96 Real Time PCR Detection System (Bio-Rad). The thermal cycling conditions were 40 cycles of 95 °C for 5 s for denaturation and 60 °C for 30 s for annealing and extension. All reactions were run in triplicate by monitoring the dissociation curve to control the dimers. PCR efficiency was determined by a series of 2-fold dilutions of cDNAs. The calculated efficiency of all primer pairs was 0.9 to 1.0. Gene Actin 2 was used as a reference and relative expression levels of genes were presented by 2^-△CT^. All primers used for qRT-PCR are presented in Additional file 1: Data S11.

### Histochemical detection of H_2_O_2_, O_2_^•−^ and intracellular ROS

Hydrogen peroxide (H_2_O_2_) and superoxide radicals (O_2_^•−^) were stained using diaminobenzidine (DAB) and nitroblue tetrazolium (NBT), respectively, according to the method described by Romero-Puertas et al. [38]. Seedlings were grown hydroponically for 21 d and transferred to the same culture solution containing 50 μM Cd for 24 h. When H_2_O_2_ was detected in tissue, Arabidopsis seedlings were incubated in DAB solution (pH 5.8, 1 mg mL^−1^) at room temperature for 24 h in the absence of light until the appearance of brown spots. For O_2_^•−^ detection, the treated plants were immersed in NBT solution (1 mg mL^−1^) at room temperature, and illuminated until the appearance of blue spots characteristic of blue formaz anprecipitate. After staining, leaves were transferred to 95% (*v*/v) ethanol to remove chlorophyll, and images were captured with a Nikon SMZ 1000 stereomicroscope.

To perform in situ detection of endogenous ROS, specific fluorescent probe DCFH-DA (2′, 7′ - dichlorofluoresce indiacetate) was used according to Foreman et al. [39]. Wild-type, *fc1* mutants and transgenic seeds were germinated and vertically grown on the half-strength MS media supplemented with 1% sucrose and 1% agar (pH 5.8). The seedlings were then transferred to 1/4 Hoagland nutrient solution moist filter paper with 50 μM Cd for 30 min. Roots of seedlings were incubated in 10 mM of DCFH-DA at 25 °C for 10 min and rinsed with distilled water for three times. After that, the samples were visualized with a fluorescence microscope (excitation 488 nm and emission 525 nm, ECLIPSE, TE2000-S, Nikon). Image-Pro Plus 6.0 (Media Cybernetics, Inc) was used to analyze The relative fluorescent density of the fluorescent images.

### Determination of O_2_^−^ and H_2_O_2_ and lipid peroxidation

Seedlings were grown hydroponically for 21 d and transferred to the same culture solution containing 50 μM Cd for 1 d. The H_2_O_2_ content was quantified as described previously with minor modification [54]. One gram of Arabidopsis shoots and roots was extracted in 1 mL of 80% ethanol. One hundred μL plant extracts were incubated for 30 min with 1 mL solution containing 90% methanol (v/v), 25 mM H_2_SO_4_ (v/v), 250 μM ferrous ammonium sulfate hexahydrate and 100 μM xylenol orange. The absorbance of the homogenate was measured at 560 nm. To calculate H_2_O_2_ concentrations, standard-curves ranging from 0 to 200 μM. Lipid peroxide, in terms of thiobarbituric acid reactive substances (TBARS), was determined by the method described previously [37].

### Analysis of non-protein thiol compounds

Seedlings were grown hydroponically for 21 d and transferred to the same culture solution without or with 50 μM Cd for 1 d. The treated seedlings were harvested and used for analysis of total GSH and total NPT. Normally, 100 mg samples were ground in liquid nitrogen with a mortar and pestle. The ground samples were mixed with 300 μL solution containing 1 M NaOH and 1 mg L^−1^ NaBH_4_. The homogenates were centrifuged at 13,000 g at 4 °C for 5 min. A 300 μL supernatant was acidified by adding 50 μL of 37% (*w*/*v*) HCl. Part of the solution was collected for measurement of GSH and NPT. Total NPT was quantified as described previously [55]. Ten μL extract indicated above was mixed with 500 μL of 6 mM 5, 5-dithiobis (2-nitrobenzoic acid) in stock buffer (143 mM sodium phosphate and 6.3 mM Na_2_-EDTA, pH 7.5), and was incubated at 30 °C for 2 min. The content of NPT was determined at 412 nm. Analysis of total GSH content was conducted using the glutathione reductase recycling assay as described by Anderson [56]. The concentration of PCs was calculated by subtracting the amount of GSH from the amount of total acid-soluble sulfhydryl compounds [20].

### Dry weight and measurement of cadmium content

Wild-type, *fc1* mutant and *AtFC1* transgenic seedlings were grown hydroponically for 21 d and transferred to the same culture solution containing 50 μM Cd for 5 d. When harvested, plants were thoroughly washed with 5 mM CaCl_2_ at 4 °C for 1 h and rinsed with sterile water. Shoots and roots from treated plants were separately sampled and dried at 75 °C for 2 d. After that, the samples were weighted. The dried samples were weighted and digested with nitric acid and hydrogen peroxide (HNO_3_: H_2_O_2_ = 1: 1, *v*/v). Total Cd contents from dried samples were determined using inductively coupled plasma-atomic emission spectrometry (ICP-AES) (Optimal 2100DV, Perkin Elmer Instruments, Waltham, MA, USA) [57].

### Chlorophyll quantification

Seedlings were grown hydroponically for 21 d and transferred to the same culture solution containing 50 μM Cd for 5 d. Chl of plant leaves (0.1 g FW) was extracted with 1 mL of 80% (v/v) acetone until complete bleaching was achieved. Chlorophyll a, Chlorophyll b and total Chlorophyll were quantified by reading the absorption at A_663_ and A_645_, and its concentration was calculated by the method described previously [58].

### Transformation of *AtFC1* in Arabidopsis

pCAMBIA1304 was employed as an expression vector with CaMV35S as a promoter and NOS terminator as a transcriptional termination sequence [59]. The *AtFC1* genomic sequences were PCR-amplified using primers with restriction enzyme sites at the 5′-end of forward and reverse primers, respectively. PCR amplified component sequences were first cloned to a T/A vector (pMD19, Takara), sequenced and digested with enzymes. The digested segments were cloned into pCAMBIA1304 and the cloning was confirmed by sequencing and restriction analysis. The confirmed clones were transformed into *Agrobacterium tumefaciens* strain LBA4404 by the floral dip method [30]. Positive transgenic lines were selected on the 1/2 MS medium with 50 mg L^−1^ kanamycin. Twenty independent *35S::AtFC1* transgenic lines (T4 homozygous lines) were obtained. Four lines were randomly selected for transcript analysis by qRT-PCR, and two of them were used for functional characterization.

### Preparation of total RNA libraries and mRNA sequencing

Two week-old Arabidopsis seedlings were exposed to 0 and 50 μM Cd and sampled at 6, 12 and 24 h, respectively. Total RNA from Cd-exposed and Cd-unexposed seedlings was isolated using the TRIzol Reagent (Invitrogen, USA) and pooled for RNA sequencing. The extracted RNA was treated with DNaseI (Qiagen, USA) at 25 °C for 30 min and confirmed in quality. mRNA was purified with oligo (dT)-rich magnetic beads and broken into short fragments. The first and second strand cDNAs were synthesized. The cDNAs were end-repaired and phosphorylated using T4 DNA polymerase and Klenow DNA polymerase. The Illumina paired-end solexa adaptors were ligated to these cDNA fragments. The ligated products were purified on a 2% agarose gel. Four libraries (Col-0-Cd, *fc1*-Cd, Col-0 + Cd and *fc1* + Cd) were sequenced using an Illumina hiseq2500.

The original image data generated by the sequence providers were transferred into nucleotide sequences data by base calling, defined as raw reads and saved as ‘fastq’ files. All subsequent analyses were performed on the high-quality clean read datasets according to the bioinformatics analysis approach summarized in Supplementary Data S6. A rigorous algorithm was used to identify differentially expressed genes (DEGs) between the samples. The expression level for each transcript was calculated as FPKM (fragments per kilobase of exon per million fragments mapped)-derived read counts based on the number of uniquely mapped reads that overlapped with exonic regions. The false discovery rate (FDR) was used to determine the threshold of the *p*-value in multiple tests, which corresponded to the differential gene expression test. In this study, FDR ≤ 0.001 and the absolute value of Log_2_Ratio > 1 were used as a threshold to judge the significant differences of gene expression. The resulting RNA-Seq data were deposited in the NCBI SRA database (Accession No. SRR5829541).

### Gene ontology analysis

The Gene Ontology (GO) category of the DEGs with functional significance was subject to the ultra-geometric test with Benjamini-Hochberg correction (http://www.geneontology.org/). GO terms with corrected *p*-value (*p* ≤ 0.05) were regarded as significant enrichment for the DEGs compared to the genome background.

### Statistical analyses

Each result shown in the figures was the mean of three biological replicates and each treatment contained at least 15–20 seedlings. The significant differences between treatments were statistically evaluated by standard deviation and analysis of variance (ANOVA). The data between differently treated groups were compared statistically by one-way ANOVA followed by the least significant difference test if the ANOVA result is significant at *p* < 0.05. The statistical analyses were performed with SPSS 12.0.

## Additional files

Additional file 1: **Data S1.** Identification of transgenic lines overexpressing *AtFC1* (*35S::AtFC1*). **Data S2.** Identification of *fc1* mutant from Arabidopsis. **Data S3.** Analysis of growth of Arabidopsis wild-type, *AtFC1* transgenic seedlings and *fc1* mutants. **Data S4.** Effect of Hematin on GSH, PCs and NPT accumulation in wide-type seedlings under Cd stress. **Data S5.** Effect of Buthionine sulfoximine (BSO) with combination of Hematin on the growth of wild-type (WT) and *fc1* mutant seedlings under Cd stress. **Data S6.** Output data of RNA-seq from four libraries exposed –Cd and +Cd. **Data S7.** Up regulated genes in *fc1*/Col-0 without Cd treatment. **Data S8.** Down regulated genes in *fc1*/Col-0 without Cd treatment. **Data S9.** Up regulated genes in *fc1*/Col-0 with Cd treatment. **Data S10.** Down regulated genes in *fc1*/Col-0 with Cd treatment. **Data S11.** Primer and probe sequences used for this study (PDF 6044 kb)

## Abbreviations

APX: Ascorbate peroxidase.
BSO: Buthionine sulfoximine
CAT: Catalase
Cd: Cadmium
Chl: Chlorophyll
DAB: 3,3-diaminobenzidine
DCF: Dichlorofluoresce
DCFH-DA: 2′, 7′ - Dichlorofluoresce indiacetate
DEGs: Differentially expressed genes
FC1: Ferrochelatase-1
GO: Gene Ontology
GR: Glutathione reductase
GSH: Glutathione
Hg: Mercury
NBT: Nitroblue tetrazolium
NPT: Non-protein thoils
Pb: Lead
PCS: Phytochelatin synthase
PCs: Phytochelatins
Proto IX: Protoporphyrin IX
ROS: Reactive oxygen species
TBARS: Thiobarbituric acid reactive substances
γ-ECS: γ-glutamyl-Cys synthetase

## References

[1] Alloway BJ, Steinnes, E. Anthropogenic additions of cadmium to soils. In Cadmium in soils and plants (eds M.J. McLaughlin & B.R. Singh). 1999; pp. 97–123. Kluwer Academic Publishers, Dordrecht, The Netherlands.

[2] Hall JL (2002). Cellular mechanisms for heavy metal detoxification and tolerance. J Exp Bot.

[3] DalCorso G, Farinati S, Furin A (2010). Regulatory networks of cadmium stress in plants. Plant Signal Behavior.

[4] Lin YF, Aaers M (2012). The molecular mechanism of zinc and cadmium stress response in plants. Cellular Mol Life Sci.

[5] Clemens S (2006). Toxic metal accumulation, responses to exposure and mechanisms of tolerance in plants. Biochimie.

[6] Garnier L, Simon-Plas F, Thuleau P, Agnel JP, Blein JP, Ranjeva RJ, Montillet L (2006). Cadmium affects tobacco cells by a series of three waves of reactive oxygen species that contribute to cytotoxicity. Plant Cell Environ.

[7] Feng SJ, Liu XS, Tao H, Tan SK, Chu SS, Oono Y, Zhang XD, Chen J, Yang ZM (2016). Variation of DNA methylation patterns associated with gene expression in rice (*Oryza sativa*) exposed to cadmium. Plant Cell Environ.

[8] Clemens S, Aarts MGM, Thomine S, Verbruggen N (2013). Plant science: the key to preventing slow cadmium poisoning. Trend Plant Sci.

[9] Cobbett C, Goldsbrough P (2002). Phytochelatins and metallothioneins: roles in heavy metal detoxification and homeostasis. Plant Biol.

[10] Schat H, Llugany M, Vooijs R, Hartley-Whitaker J, Bleeker PM (2002). The role of phytochelatins in constitutive and adaptive heavy metal tolerance in hyperaccumulator and non-hyperaccumulator metallophytes. J Exp Bot.

[11] Mendoza-Cozatl D, Butko E, Springer F, Torpey J, Komives E, Kehr J, Schroeder J (2008). Identification of high levels of phytochelatins, glutathione and cadmium in the phloem sap of *Brassica napus*. A role for thiol-peptides in the long-distance transport of cadmium and the effect of cadmium on iron translocation. Plant J.

[12] Grill E, Winnacker EL, Zenk MH (1985). Phytochelatins: the principal heavy-metal complexing peptides of higher plants. Science.

[13] Salt DE, Prince RC, Pickering IJ, Raskin I (1995). Mechanisms of cadmium mobility and accumulation in Indian mustard. Plant Physiol.

[14] Brunetti P, Zanella L, Proia A, De Paolis A, Falasca G, Altamura MM (2011). Sanita di Toppi L, Costantino P, Cardarelli M. Cadmium tolerance and phytochelatin content of *Arabidopsis* seedlings overexpressing the phytochelatin synthase gene *AtPCS1*. J Exp Bot.

[15] Kuhnlenz T, Schmidt H, Uraguchi S, Clemens S (2014). *Arabidopsis thaliana* phytochelatin synthase 2 is constitutively active *in vivo* and can rescue the growth defect of the *PCS1*-deficient *cad1-3* mutant on Cd-contaminated soil. J Exp Bot.

[16] Shim D, Hwang JU, Lee J, Lee S, Choi Y, An G, Martinoia E, Lee Y (2009). Orthologs of the class A4 heat shock transcription factor HsfA4a confer cadmium tolerance in wheat and rice. Plant Cell.

[17] Huang J, Zhang Y, Peng JS, Zhong C, Yi HY, Ow DW, Gong JM (2012). Fission yeast HMT1 lowers seed cadmium through phytochelatin-dependent vacuolar sequestration in Arabidopsis. Plant Physol.

[18] Koffler BE, Polanschütz L, Zechmann B (2014). Higher sensitivity of pad2-1 and vtc2-1 mutants to cadmium is related to lower subcellular glutathione rather than ascorbate contents. Protoplasma.

[19] Chen J, Yang L, Yan X, Liu Y, Wang R, Fan T, Ren Y, Tang X, Xiao F, Liu YS, Cao S (2016). Zinc-finger transcription factor ZAT6 positively regulates cadmium tolerance through the glutathione-dependent pathway in Arabidopsis. Plant Physiol.

[20] Chen J, Yang L, Gu J, Bai X, Ren Y, Fan T, Han Y, Jiang L, Xiao F, Liu Y, Cao S (2015). *MAN3* gene regulates cadmium tolerance through the glutathione-dependent pathway in *Arabidopsis thaliana*. New Phytol.

[21] Brunetti P, Zanella L, De Paolis A, Litta DD, Cecchetti V, Falasca G, Barbieri M, Altamura MM, Costantino P, Cardarelli M. Cadmium-inducible expression of the ABC-type transporter *AtABCC3* increases phytochelatin-mediated cadmium tolerance in *Arabidopsis*. J Exp Bot 2015; 66: 3815−3829. 10.1093/jxb/erv185 PMC4473984 25900618

[22] Smith AG, Santana MA, Wallace-Cook AD, Roper JM, Labbe-Bois R (1994). Isolation of a cDNA encoding chloroplast ferrochelatase from Arabidopsis Thaliana by functional complementation of a yeast mutant. J Biol Chem.

[23] Chow KS, Singh DP, Walker AR, Smith AG (1998). Two different genes encode ferrochelatase in Arabidopsis: mapping, expression and subcellular targeting of the precursor proteins. Plant J.

[24] Suzuki T, Masuda T, Singh DP, Tan FC, Tsuchiya T, Shimada H, Takamiya K (2002). Two types of ferrochelatase in photosynthetic and nonphotosynthetic tissues of cucumber: their difference in phylogeny, gene expression, and localization. J Biol Chem.

[25] Heinemann IU, Jahn M, Jahn D (2008). The biochemistry of heme biosynthesis. Arch. Biochem. Biophy.

[26] Singh DP, Cornah JE, Hadingham S, Smith AG (2002). Expression analysis of the two ferrochelatase genes in Arabidopsis in different tissues and under stress conditions reveals their different roles in haem biosynthesis. Plant Mol Biol.

[27] Nagai S, Koide M, Takahashi S, Kikuta A, Aono M, Sasaki-Sekimoto Y, Masuda T (2007). Induction of isoforms of tetrapyrrole biosynthetic enzymes, *AtHEMA2* and *AtFC1*, under stress conditions and their physiological functions in Arabidopsis. Plant Physiol.

[28] Cao ZY, Geng BB, Xu S, Xuan W, Nie L, Shen WB, Liang YC, Guan RZ (2011). *BnHO1*, a haem oxyenase-1 gene from *Brassica napus*, is required for salinity and osmotic stress-induced lateral root formation. J Exp Bot.

[29] Phung TH, Jung HI, Park JH, Kim JG, Back K, Jung S (2011). Porphyrin biosynthesis control under water stress: sustained porphyrin status correlates with drought tolerance in transgenic rice. Plant Physiol.

[30] Shen Q, Jiang M, Li H, Che LL, Yang ZM (2011). Expression of a *Brassica napus* heme oxygenase confers plant tolerance to mercury toxicity. Plant Cell Environ.

[31] Ajioka RS, Phillips JD, Kushner JP (2006). Biosynthesis of heme in mammals. Biochim Biophy Acta.

[32] Watanabe S, Hanaoka M, Ohba Y, Ono T, Ohnuma M, Yoshikawa H, Taketani S, Tanaka K (2013). Mitochondrial localization of ferrochelatase in a red alga *Cyanidioschyzon merolae*. Plant Cell Physiol..

[33] Scharfenberg M Mittermayr L, von Roepenack-Lahaye E, Schlicke H, Grimm B, Leister D, Kleine T. Functional characterization of the two ferrochelatases in *Arabidopsis thaliana*. Plant Cell Environ 2015; 38: 280−298. 10.1111/pce.12248 24329537

[34] Espinas NA, Kobayashi K, Sato Y, Mochizuki N, Takahashi K, Tanaka R, Masuda T (2016). Allocation of heme is differentially regulated by ferrochelatase isoforms in *Arabidopsis* cells. Front Plant Sci.

[35] Woodson JD, Perez-Ruiz JM, Chory J (2011). Heme synthesis by plastid ferrochelatase I regulates nuclear gene expression in plants. Curr Biol.

[36] Kim JG, Back K, Lee HY, Lee HJ, Phung TH, Grimm B, Jung S (2014). Increased expression of Fe-chelatase leads to increased metabolic flux into heme and confers protection against photodynamically induced oxidative stress. Plant Mol Biol.

[37] Zhou ZS, Wang SJ, Yang ZM (2008). Biological detection and analysis of mercury toxicity to alfalfa (*Medicago sativa*) plants. Chemosphere.

[38] Romero-Puertas MC, Rodriguez-Serrano M, Corpas FJ, GomezM del Rio LA, Sandalio LM (2004). Cadmium-induced subcellular accumulation of O_2_^-.^ And H_2_O_2_ in pea leaves. Plant Cell Environ.

[39] Foreman J, Demidchik V, Bothwell JH, Mylona P, Miedema H (2003). Reactive oxygen species produced by NADPH oxidase regulate plant cell growth. Nature.

[40] Li H, Song JB, Zhao WT, Yang ZM (2013). AtHO1 is involved in iron homeostasis in a NO-dependent manner. Plant Cell Physiol..

[41] Clemens S (2006). Toxic metal accumulation, responses to exposure and mechanisms of tolerance in plants. Boichimie.

[42] Xiang C, Oliver DJ (1998). Glutathione metabolic genes coordinately respond to heavy metals and jasmonic acid in Arabidopsis. Plant Cell.

[43] Anjum NA, Ahmad I, Mohmood I, Pacheco M, Duarte AC, Pereira E, Umar S, Ahmad A, Khan NA, Iqbal M, Prasad MNV (2012). Modulation of glutathione and its related enzymes in plants responses to toxic metals and metalloids—a review environ. Experi Bot.

[44] Guo JB, Dai X, Xu W, Ma M (2008). Overexpressing GSH1 and AsPCS1 simultaneously increases the tolerance and accumulation of cadmium and arsenic in *Arabidopsis thaliana*. Chemosphere.

[45] Chmielowska-Bak J, Gzyl J, Rucinska-Sobkowiak R, Arasimowicz-Jelonek M, Deckert J (2014). The new insights into cadmium sensing. Front Plant Sci.

[46] Jozefczak M, Bohler S, Schat H, Horemans N, Guisez Y, Remans T, Vangronsveld J, Cuypers A (2015). Both the concentration and redox state of glutathione and ascorbate influence the sensitivity of Arabidopsis to cadmium. Ann Bot (Lond).

[47] Zhu YL, Pilon-Smits EA, Jouanin L, Terry N (1999). Overexpression of glutathione synthetase in *Indian mustard* enhances cadmium accumulation and tolerance. Plant Physiol.

[48] Ivanova LA, Ronzhina DA, Ivanov LA, Stroukova LV, Peuke AD, Rennenberg H (2011). Over-expression of gsh1 in the cytosol affects the photosynthetic apparatus and improves the performance of transgenic poplars on heavy metal-contaminated soil. Plant Biol (Stuttgart).

[49] Hernández LE, Sobrino-Plata J, Montero-Palmero MB, Carrasco-Gil S, Flores-Cáceres ML, Ortega-Villasante C, Escobar C (2015). Contribution of glutathione to the control of cellular redox homeostasis under toxic metal and metalloid stress. J Exp Bot.

[50] Wang HC, Wu JS, Chia JC, Yang CC, Wu YJ, Juang RH (2009). Phytochelatin synthase is regulated by protein phosphorylation at a threonine residue near its catalytic site. J Agri Food Chem.

[51] Méndez R, de Haro C. Casein kinase II is implicated in the regulation of heme-controlled translational inhibitor of reticulocyte lysates. J Biol Chem 1994; 269: 6170−6176. 7907089

[52] Hossain MA, Piyatida P, da Silva JAT, Fujita M (2012). Molecular mechanism of heavy metal toxicity and tolerance in plants: central role of glutathione in detoxification of reactive oxygen species and methylglyoxal and in heavy metal chelation. J Bot.

[53] Zámocký M, Hofbauer S, Schaffner I, Gasselhuber B, Nicolussi A, Soudi M, Pirker KF, Furtmüller PG, Obinger C (2015). Independent evolution of four heme peroxidase superfamilies. Arch Biochem Biophy.

[54] DeLong JM, Prange RK, Hodges DM, Forney CF, Bishop MC, Quilliam M (2002). Using a modified ferrous oxidation-xylenol orange (FOX) assay for detection of lipid hydroperoxides in plant tissue. J Agric Food Chem.

[55] Lee S, Moon JS, Ko TS, Petros D, Goldsbrough PB, Korban SS (2003). Overexpression of Arabidopsis phytochelatin synthase paradoxically leads to hypersensitivity to cadmium stress. Plant Physiol.

[56] Anderson ME (1985). Determination of glutathione and glutathione disulfide in biological samples. Methods Enzymol.

[57] Zheng Q, Cheng ZZ, Yang ZM (2013). *HISN3* mediates adaptive response of *Chlamydomonas reinhardtii* to excess nickel. Plant Cell Physiol.

[58] Porra RJ, Thompson RA, Kriedemann PE (1989). Determination of accurate extinction coefficients and simultaneous equations for assaying chlorophylls a and b extracted with four different solvent verifications of the concentration of chlorophyll standards by atomic absorption spectroscopy. Biochem Biophys Acta.

[59] Gao S, Zhang YL, Yang L, Song JB, Yang ZM (2014). *AtMYB20* is negatively involved in plant adaptive response to drought stress. Plant Soil.

