# Supplementary material for: A cadmium stress-responsive gene AtFC1 confers plant tolerance to cadmium toxicity
Source: BMC Plant Biol. 2017 Oct 30;17:187. doi: 10.1186/s12870-017-1141-0 (PMC5663144; doi:10.1186/s12870-017-1141-0)
Supplement: Supplementary file 1 — Data S1. Identification of transgenic lines overexpressing AtFC1 (35S::AtFC1). Data S2. Identification of fc1 mutant from Arabidopsis. Data S3. Analysis of growth of Arabidopsis wild-type, AtFC1 transgenic seedlings and fc1 mutants. Data S4. Effect of Hematin on GSH, PCs and NPT accumulation in wide-type seedlings under Cd stress. Data S5. Effect of Buthionine sulfoximine (BSO) with combination of Hematin on the growth of wild-type (WT) and fc1 mutant seedlings under Cd stress. Data S6. Output data of RNA-seq from four libraries exposed –Cd and +Cd. Data S7. Up regulated genes in fc1/Col-0 without Cd treatment. Data S8. Down regulated genes in fc1/Col-0 without Cd treatment. Data S9. Up regulated genes in fc1/Col-0 with Cd treatment. Data S10. Down regulated genes in fc1/Col-0 with Cd treatment. Data S11. Primer and probe sequences used for this study (PDF 6044 kb) [file 12870_2017_1141_MOESM1_ESM.pdf]

Additional file

Title: A cadmium stress-responsive gene *AtFC1* confers plant tolerance to cadmium toxicity

Running head: *AtFC1* confers plant Cd tolerance

Full names of all authors: [1*]Jun Song, [1*]Sheng Jun Feng, [2]Jian Chen, [3] Wen Ting Zhao, [1]Zhi Min Yang

Name of Institution: [1]Department of Biochemistry and Molecular Biology, College of Life Sciences, Nanjing Agricultural University, Nanjing, China; [2]Institute of Food Quality and Safety, Jiangsu Academy of Agricultural Sciences, Nanjing 210014, China; [3]Institute of Plant Nutrition (IFZ), Justus Liebig University, Heinrich-Buff-Ring 26-32, 35392 Giessen, Germany

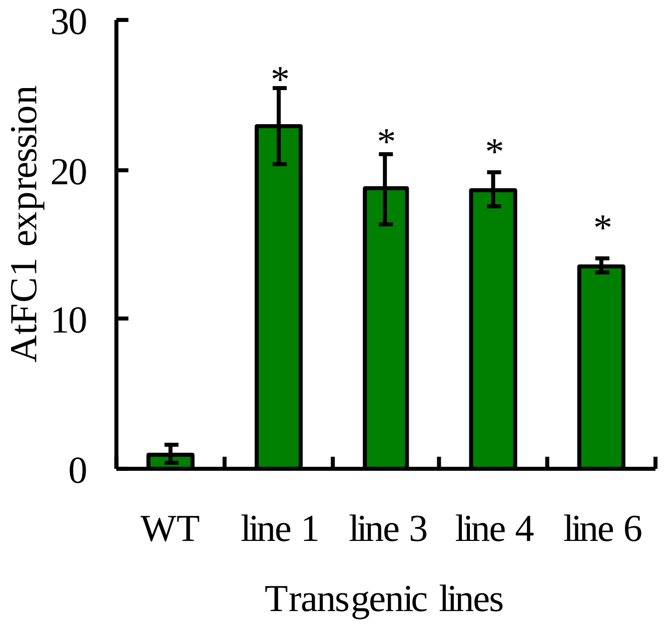

Supplementary Data S1. Identification of transgenic lines overexpressing *AtFC1* (*35S::AtFC1*). Total RNA from 14 d old seedlings was extracted and its expression was analyzed by qRT-PCR. Vertical bars represent standard deviation of the mean. Asterisk indicates the significant difference in expression between the *35S::AtFC1* lines and wild type ($p<0.05$).

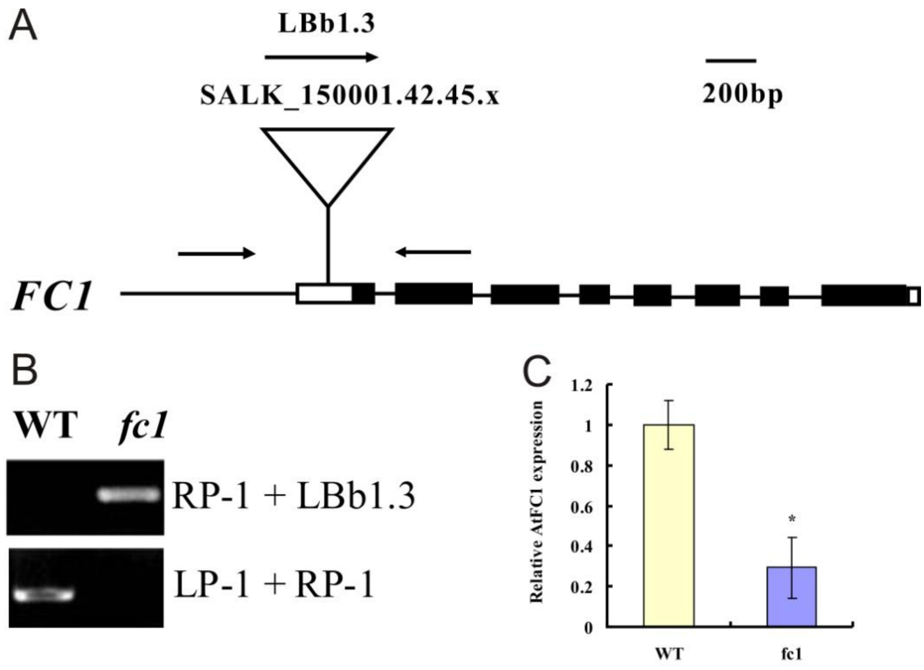

Supplementary Data S2. Identification of *fc1* mutant from Arabidopsis. Total RNA from 14 d old seedlings was extracted and its expression was analyzed by qRT-PCR. Vertical bars represent standard deviation of the mean. Asterisk indicates the significant difference in expression between the *35S::AtFC1* lines and wild type (p<0.05). (A): Schematic diagram of *AtFC1* structure and T-DNA diagnostic PCR and RT-PCR. The coding region and 5' and 3' untranslated regions are identified by the black and white boxes, respectively; introns are indicated by lines. T-DNA insertion position is indicated by triangle. The locations of the primer pairs used to analyze the mutation by RT-PCR are indicated by the arrows. (B): RT-PCR analysis of the *fc1* insertion mutant. The reverse transcription products were PCR-amplified using primer pairs LP-1 + RP-1, LP-1 + LBb1.3. (C): *AtFC1* transcript levels in *fc1* mutant.

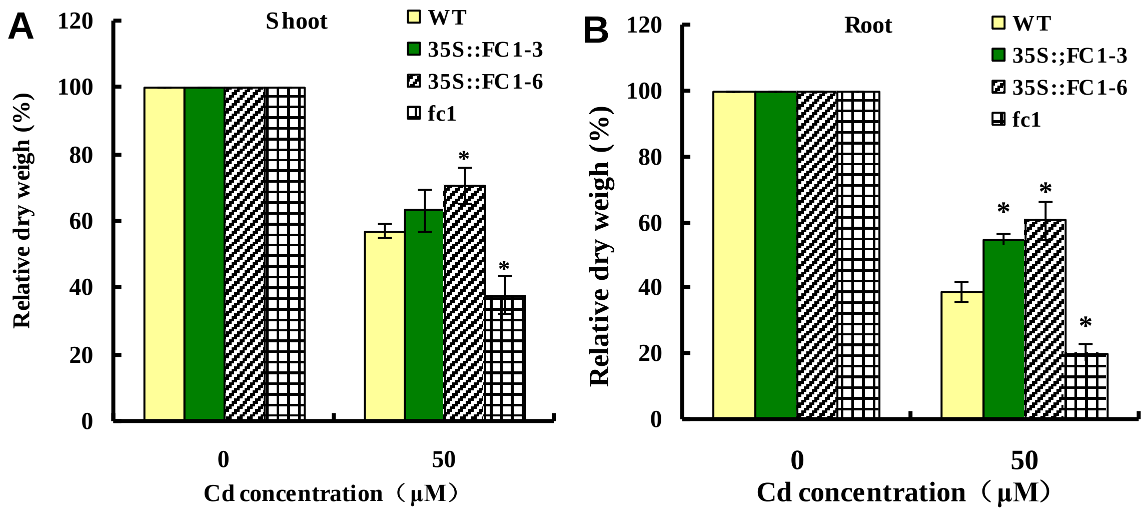

Supplementary Data S3. Analysis of growth of Arabidopsis wild-type, *AtFC1* transgenic seedlings and *fc1* mutants. Seedlings were grown hydroponically for 21 d and transferred to the same culture solution with and without 50 μM $CdCl_2$ for 5 d. Asterisks indicate that the mean values are significantly different between the transgenic plants/mutants and WT($P<0.05$).

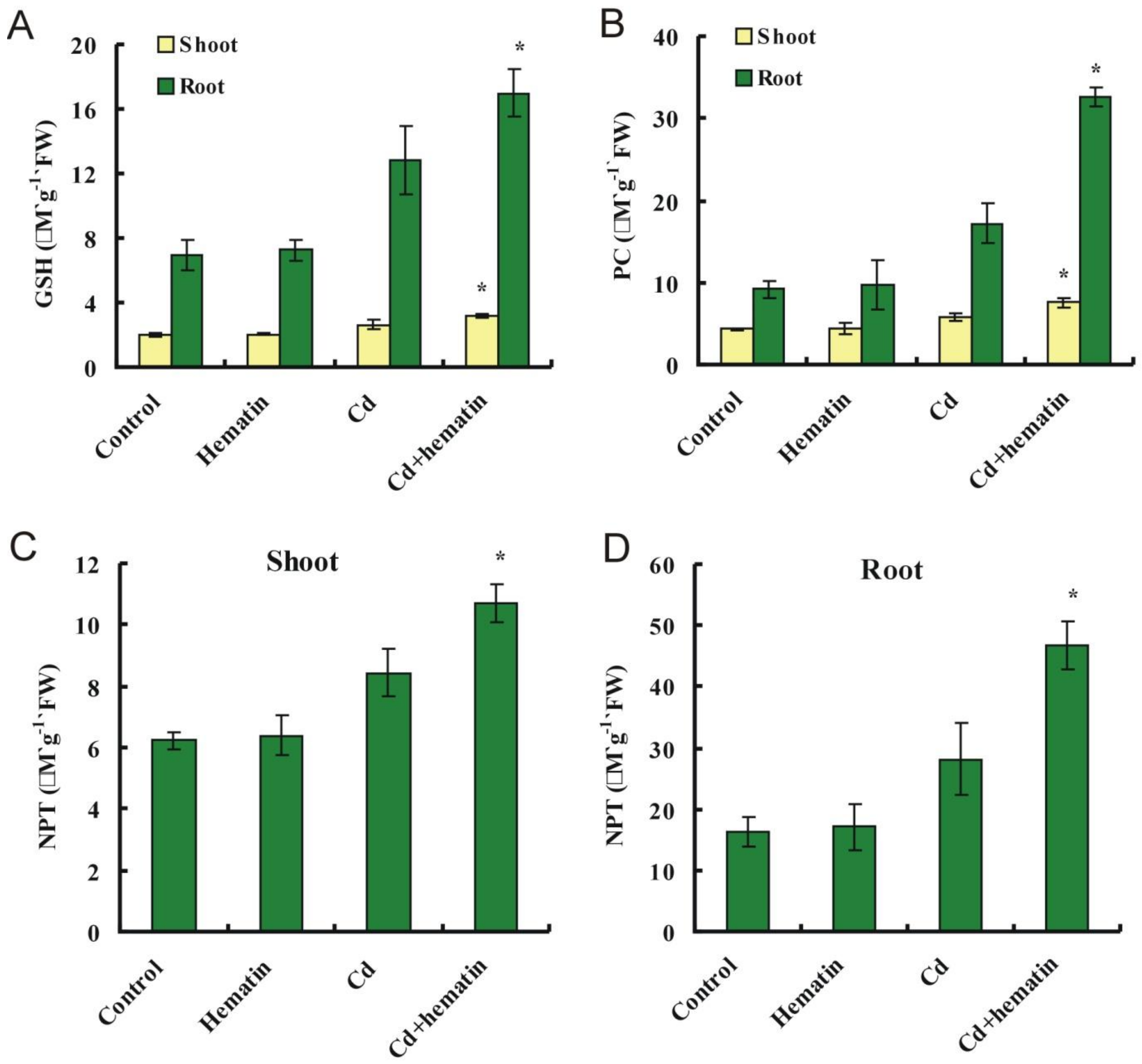

Supplementary Data S4. Effect of Hematin on GSH, PCs and NPT accumulation in wide-type seedlings under Cd stress. Wild-type seedlings were grown on 1/2 MS medium with 50 μM Cd, 10 μM Hematin or 50 μM Cd+10 μM Hematin for 12 d. (A) GSH contents. (B) PC contents. (C) NPT in shoots. (D) NPT in roots. Vertical bars represent standard deviation. Asterisks indicate that the mean values are significantly different between Cd and Cd+Hematin treatments (p<0.05).

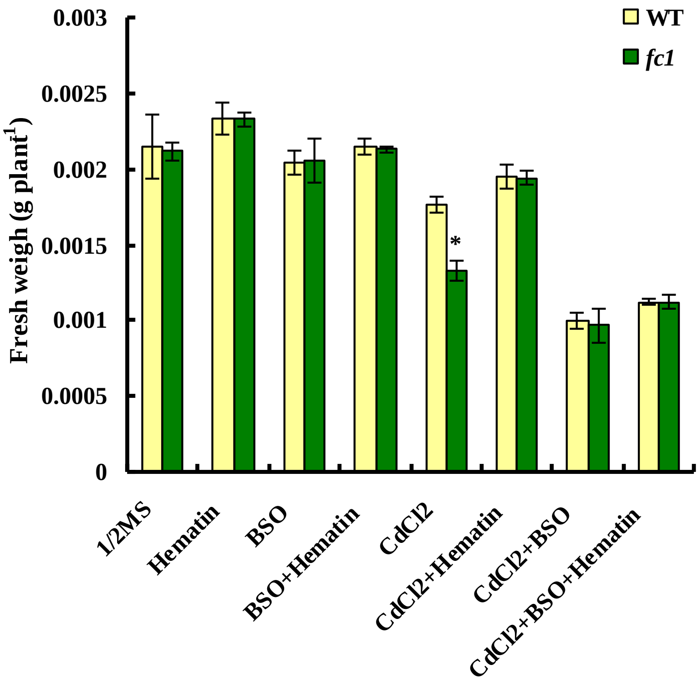

Supplementary Data S5. Effect of Buthionine sulfoximine (BSO) with combination of Hematin on the growth of wild-type (WT) and *fc1* mutant seedlings under Cd stress. Seedlings were grown on 1/2 MS media with 50 µM Cd, 100 µM BSO or 10 µM Hematin for 12 d. Vertical bars represent standard deviation. Asterisks indicate that the mean values are significantly different between *fc1* and WT plants (p<0.05).

Sample	Col-0 (-Cd)	*fc1* (-Cd)	Col-0 (+Cd)	*fc1* (+Cd)
Raw Reads Number	35084424	33540540	32801764	33784758
Raw Reads Length (bp)	150	150	150	150
Clean Reads Number	31643626	30471310	29907118	30854898
Clean Reads Length (bp)	150	150	150	150
Clean Reads Rate (%)	90.19	90.85	91.18	91.33
Low-quality Reads	3021236	2733958	2555128	2617036
Low-quality Reads Rate	8.61	8.15	7.79	7.75

Supplementary Data S6 Output data of RNA-seq from four libraries exposed –Cd and +Cd.

Supplementary Data S7 Up regulated genes in *fc1*/Col-o without Cd treatment.

Gene	Log2FoldChange	pval	NT:Description
AT5G04140	1.389344288	0	Arabidopsis thaliana ferredoxin-dependent glutamate synthase 1 mRNA, complete cds
AT4G28080	2.607621419	0	Arabidopsis thaliana tetratricopeptide repeat domain protein mRNA, complete cds
AT1G01320	2.351735702	0	Arabidopsis thaliana tetratricopeptide repeat-containing protein mRNA, complete cds
AT5G40450	3.202087392	0	Arabidopsis thaliana uncharacterized protein mRNA, complete cds
AT5G53460	1.677740747	0	Arabidopsis thaliana glutamate synthase 1 [NADH] mRNA, complete cds
AT1G70320	2.654025541	0	Arabidopsis thaliana ubiquitin-protein ligase 2 mRNA, complete cds
AT2G28290	3.603087395	0	Arabidopsis thaliana chromatin structure-remodeling complex protein SYD mRNA, complete cds
AT3G02260	2.891386553	0	Arabidopsis thaliana auxin transport protein BIG mRNA, complete cds
AT1G15290	1.886368092	0	Arabidopsis thaliana tetratricopeptide repeat-containing protein mRNA, complete cds
AT1G55490	1.036839121	0	Arabidopsis thaliana chaperonin 60 subunit beta 1 mRNA, complete cds
AT1G28290	1.997791239	0	Arabidopsis thaliana arabinogalactan protein 31 mRNA, complete cds
AT4G29060	1.187031079	0	Arabidopsis thaliana elongation factor Ts family protein mRNA, complete cds
AT1G80070	2.084031631	0	Arabidopsis thaliana putative splicing factor Prp8 mRNA, complete cds
AT1G55860	2.937866497	0	Arabidopsis thaliana ubiquitin-protein ligase 1 mRNA, complete cds
AT5G41790	2.663942024	0	Arabidopsis thaliana COP1-interactive protein 1 mRNA, complete cds
AT4G25100	1.75884258	0	Arabidopsis thaliana superoxide dismutase [Fe] mRNA, complete cds
AT1G79280	2.719902076	0	Arabidopsis thaliana nuclear pore anchor mRNA, complete cds
AT1G36160	1.862679922	0	Arabidopsis thaliana acetyl-CoA carboxylase 1 mRNA, complete cds

AT4G17330	2.089028687	0	Arabidopsis thaliana G2484-1 protein mRNA, complete cds
AT1G48090	2.479172821	0	Arabidopsis thaliana calcium-dependent lipid-binding family protein mRNA, complete cds
AT3G22380	1.423424077	0	Arabidopsis thaliana clock regulator protein TIME FOR COFFEE mRNA, complete cds
AT2G46020	2.335527479	0	Arabidopsis thaliana ATP-dependent helicase BRAHMA mRNA, complete cds
AT3G41979	5.193100928	7.42E-302	Erysimum belvederense internal transcribed spacer 1, partial sequence; 5.8S ribosomal RNA gene, complete sequence; and internal transcribed spacer 2, partial sequence
AT4G02510	1.228947199	1.49E-296	Arabidopsis thaliana translocase of chloroplast 159 mRNA, complete cds
AT3G50370	1.77331863	1.54E-288	Arabidopsis thaliana uncharacterized protein mRNA, complete cds
AT4G01800	1.291762257	2.57E-288	Arabidopsis thaliana protein translocase subunit secA mRNA, complete cds
AT2G17930	2.428778749	4.16E-285	Arabidopsis thaliana phosphatidylinositol 3- and 4-kinase family protein with FAT domain mRNA, complete cds
AT5G44800	2.708577084	9.57E-281	Arabidopsis thaliana chromatin remodeling 4 protein mRNA, complete cds
AT1G64790	1.985985446	5.18E-271	Arabidopsis thaliana protein ILITHYIA mRNA, complete cds
AT1G20960	2.029056128	4.88E-270	Arabidopsis thaliana putative U5 small nuclear ribonucleoprotein helicase mRNA, complete cds
AT1G02080	1.736004464	3.71E-269	Arabidopsis thaliana CCR4-NOT transcription complex subunit 1 domain protein mRNA, complete cds
AT4G24190	1.046901471	1.10E-266	Arabidopsis thaliana HSP90-like protein GRP94 mRNA, complete cds
AT4G35800	2.018523924	9.72E-254	Arabidopsis thaliana DNA-directed RNA polymerase II subunit RPB1 mRNA, complete cds
AT5G35210	1.570448969	2.65E-252	Arabidopsis thaliana PHD type transcription factor with transmembrane domain protein mRNA, complete cds
AT4G10120	1.503637078	1.52E-249	Arabidopsis thaliana probable

			sucrose-phosphate synthase 4 mRNA, complete cds
AT2G35630	1.833857639	4.24E-238	Arabidopsis thaliana protein MICROTUBULE ORGANIZATION 1 mRNA, complete cds
AT1G03080	2.394693862	2.19E-234	Arabidopsis thaliana kinase interacting (KIP1-like) protein mRNA, complete cds
AT1G03060	2.309363504	4.17E-219	Arabidopsis thaliana WD/BEACH domain protein SPIRRIG mRNA, complete cds
AT2G22125	1.569800055	1.94E-218	Arabidopsis thaliana cellulose synthase-interactive protein 1 mRNA, complete cds
AT5G47690	1.464955189	3.43E-216	Arabidopsis thaliana uncharacterized binding protein mRNA, complete cds
AT3G08530	1.029556825	7.73E-214	Arabidopsis thaliana Clathrin, heavy chain mRNA, complete cds
AT4G17140	2.230990331	2.05E-212	Arabidopsis thaliana pleckstrin homology (PH) domain-containing protein mRNA, complete cds
AT5G04560	2.42681465	5.90E-212	Arabidopsis thaliana transcriptional activator DEMETER mRNA, complete cds
AT1G70620	1.528900354	1.14E-207	Arabidopsis thaliana cyclin-related protein mRNA, complete cds
AT4G15180	2.567930988	4.87E-207	Arabidopsis thaliana putative histone-lysine N-methyltransferase ATXR3 mRNA, complete cds
AT1G65010	2.11222719	3.18E-206	Arabidopsis thaliana flower development-related protein mRNA, complete cds
AT4G38950	1.820737397	2.28E-198	Arabidopsis thaliana ATP binding microtubule motor family protein mRNA, complete cds
AT3G60240	1.713033932	8.77E-196	Arabidopsis thaliana eukaryotic translation initiation factor 4G mRNA, complete cds
AT1G21580	1.804929681	1.78E-195	Arabidopsis thaliana zinc finger CCCH domain-containing protein mRNA, complete cds
AT1G67120	3.40809547	2.72E-195	Arabidopsis thaliana protein MIDASIN1 mRNA, complete cds
AT5G23110	2.398233585	3.28E-192	Arabidopsis thaliana uncharacterized protein mRNA, complete cds
AT1G65440	1.979316768	3.68E-189	Arabidopsis thaliana transcription elongation factor SPT6-like protein

			mRNA, complete cds
AT1G55350	1.579907971	9.61E-187	Arabidopsis thaliana calpain-type cysteine protease DEK1 mRNA, complete cds
AT1G35115	3.314774245	2.39E-184	Arabidopsis thaliana chromosome 1 sequence
AT4G11420	1.164426717	3.94E-182	Arabidopsis thaliana eukaryotic translation initiation factor 3A mRNA, complete cds
AT4G36520	2.483016725	1.88E-181	Arabidopsis thaliana chaperone DnaJ-domain containing protein mRNA, complete cds
AT1G76810	1.424865367	8.98E-181	Arabidopsis thaliana eukaryotic translation initiation factor 2 (eIF-2) family protein mRNA, complete cds
AT4G38600	1.2550525	8.51E-175	Arabidopsis thaliana HECT ubiquitin protein ligase family protein mRNA, complete cds
AT2G35050	1.261779182	1.53E-174	Arabidopsis thaliana protein kinase family protein mRNA, complete cds
AT5G13000	1.239403148	6.05E-174	Arabidopsis thaliana callose synthase 3 mRNA, complete cds
AT3G19050	3.61775215	5.47E-173	Arabidopsis thaliana phragmoplast orienting kinesin 2 mRNA, complete cds
AT3G22790	2.943427203	2.22E-171	Arabidopsis thaliana protein NETWORKED 1A mRNA, complete cds
AT3G48870	1.219402132	1.55E-170	Arabidopsis thaliana Clp ATPase mRNA, complete cds
AT1G26150	1.524127012	4.88E-170	Arabidopsis thaliana proline-rich extensin-like receptor kinase 10 mRNA, complete cds
AT1G67230	1.599328649	7.49E-170	Arabidopsis thaliana protein little nuclei1 mRNA, complete cds
AT4G31570	2.745254663	1.83E-169	Arabidopsis thaliana AUCSIA-1 interacting protein mRNA, complete cds
AT4G14760	2.717204393	1.65E-163	Arabidopsis thaliana kinase interacting-like protein mRNA, complete cds
AT1G79000	1.9424249	5.80E-163	Arabidopsis thaliana histone acetyltransferase HAC1 mRNA, complete cds
AT2G36910	1.431160939	8.00E-163	Arabidopsis thaliana ABC transporter B family member 1 mRNA, complete cds
AT3G52140	1.364891735	2.69E-161	Arabidopsis thaliana tetratricopeptide

			repeat (TPR)-containing protein mRNA, complete cds
AT3G16000	1.357564103	3.43E-159	Arabidopsis thaliana MAR-binding filament-like protein 1 mRNA, complete cds
AT5G20490	1.470773634	7.41E-153	Arabidopsis thaliana Myosin XI K mRNA, complete cds
AT1G50030	1.731194165	8.44E-152	Arabidopsis thaliana serine/threonine-protein kinase TOR mRNA, complete cds
AT4G00450	1.973509116	4.94E-151	Arabidopsis thaliana transcriptional regulator MED12-like protein mRNA, complete cds
AT1G21630	1.578626618	9.51E-149	Arabidopsis thaliana calcium-binding EF-hand-containing protein mRNA, complete cds
AT2G45540	1.377877952	1.36E-144	Arabidopsis thaliana beige-related and WD-40 repeat-containing protein mRNA, complete cds
AT3G23890	2.119925558	1.14E-143	Arabidopsis thaliana DNA topoisomerase 2 mRNA, complete cds
AT2G32240	1.231695789	1.27E-142	Arabidopsis thaliana uncharacterized protein mRNA, complete cds
AT1G58250	1.974939239	4.45E-139	Arabidopsis thaliana protein SABRE mRNA, complete cds
AT2G13370	1.810665861	9.18E-139	Arabidopsis thaliana chromatin remodeling 5 mRNA, complete cds
AT1G32750	2.115521917	1.38E-138	Arabidopsis thaliana histone acetyltransferase of the CBP family 13 mRNA, complete cds
AT4G36080	2.861492003	1.95E-138	Arabidopsis thaliana phosphotransferases/inositol or phosphatidylinositol kinases mRNA, complete cds
AT3G04340	1.301535468	1.32E-134	Arabidopsis thaliana protein EMBRYO DEFECTIVE 2458 mRNA, complete cds
AT1G23720	1.452675283	2.46E-134	Arabidopsis thaliana chromosome 1 sequence
AT4G18240	1.443881313	9.55E-134	Arabidopsis thaliana starch synthase 4 mRNA, complete cds
AT1G20970	1.69666033	3.37E-131	Arabidopsis thaliana uncharacterized protein mRNA, complete cds
AT2G31960	1.336596432	2.35E-130	Arabidopsis thaliana glucan synthase-like 3 mRNA, complete cds

AT2G43150	1.086239536	3.78E-130	Arabidopsis thaliana Proline-rich extensin-like family protein mRNA, complete cds
AT4G26630	1.122401617	1.73E-128	Arabidopsis thaliana DEK domain-containing chromatin associated protein mRNA, complete cds
AT2G42270	1.768886631	5.41E-128	Arabidopsis thaliana U5 small nuclear ribonucleoprotein helicase mRNA, complete cds
AT2G25170	1.569784179	2.32E-127	Arabidopsis thaliana CHD3-type chromatin-remodeling factor PICKLE mRNA, complete cds
AT1G77300	2.218581148	2.66E-127	Arabidopsis thaliana histone-lysine N-methyltransferase ASHH2 mRNA, complete cds
AT1G22060	2.194335433	3.94E-125	Arabidopsis thaliana uncharacterized protein mRNA, complete cds
AT2G34680	1.229832243	1.37E-124	Arabidopsis thaliana protein AUXIN-INDUCED IN ROOT CULTURES 9 mRNA, complete cds
AT3G22120	1.854190633	3.21E-124	Arabidopsis thaliana chromosome 3, complete sequence
AT1G10760	1.501611325	4.13E-124	Arabidopsis thaliana alpha-glucan water dikinase 1 mRNA, complete cds
AT3G43300	1.218319491	4.24E-123	Arabidopsis thaliana brefeldin A-inhibited guanine nucleotide-exchange protein 5 mRNA, complete cds
AT5G48570	1.355698928	2.94E-121	Arabidopsis thaliana peptidyl-prolyl cis-trans isomerase FKBP65 mRNA, complete cds
AT1G68890	1.523542687	8.06E-121	Arabidopsis thaliana protein PHYLLO mRNA, complete cds
AT2G26890	1.552456343	4.45E-120	Arabidopsis thaliana gravitropism defective 2 mRNA, complete cds
AT2G36850	1.159337144	1.35E-119	Arabidopsis thaliana glucan synthase-like 8 mRNA, complete cds
AT5G13010	1.452358799	2.74E-119	Arabidopsis thaliana RNA helicase family protein mRNA, complete cds
AT1G68790	1.980179544	5.73E-119	Arabidopsis thaliana little nuclei3 protein mRNA, complete cds
AT1G79830	1.173213996	4.27E-117	Arabidopsis thaliana golgin candidate 5 mRNA, complete cds
AT5G61140	1.647986244	6.12E-117	Arabidopsis thaliana U5 small nuclear ribonucleoprotein helicase mRNA,

			complete cds
AT4G01290	1.221179119	8.30E-117	Arabidopsis thaliana uncharacterized protein mRNA, complete cds
AT1G76930	2.153572564	6.19E-116	Arabidopsis thaliana extensin 4 mRNA, complete cds
AT1G24706	1.959194519	1.14E-115	Arabidopsis thaliana THO complex subunit 2 mRNA, complete cds
AT3G54670	1.965685417	4.01E-115	Arabidopsis thaliana structural maintenance of chromosomes 1 mRNA, complete cds
AT2G39580	1.29068529	5.27E-115	Arabidopsis thaliana uncharacterized protein mRNA, complete cds
AT2G16485	2.027340153	6.59E-114	Arabidopsis thaliana GW repeat- and PHD finger-containing protein NERD mRNA, complete cds
AT3G57300	1.426180763	2.39E-111	Arabidopsis thaliana DNA helicase INO80 complex-like 1 mRNA, complete cds
AT1G17360	1.337104518	3.95E-109	Arabidopsis thaliana uncharacterized protein mRNA, complete cds
AT2G03150	1.579049292	5.41E-109	Arabidopsis thaliana protein EMBRYO DEFECTIVE 1579 mRNA, complete cds
AT1G24460	1.8903701	1.72E-108	Arabidopsis thaliana TGN-localized SYP41 interacting protein mRNA, complete cds
AT4G39420	1.901236341	6.50E-107	Arabidopsis thaliana uncharacterized protein mRNA, complete cds
AT5G23150	2.125271795	1.31E-105	Arabidopsis thaliana ENHANCER OF AG-4 protein 2 mRNA, complete cds
AT3G11964	1.766549441	1.69E-105	Arabidopsis thaliana protein ribosomal RNA processing 5 mRNA, complete cds
AT3G01370	1.332661061	3.14E-105	Arabidopsis thaliana CRM family member 2 mRNA, complete cds
AT1G28420	2.025636662	3.43E-105	Arabidopsis thaliana homeobox-1 mRNA, complete cds
AT3G54580	1.434790337	7.77E-104	Arabidopsis thaliana chromosome 3, complete sequence
AT5G52640	1.032017725	1.31E-103	Arabidopsis thaliana heat shock protein 90.1 mRNA, complete cds
AT3G47910	1.723700024	1.69E-103	Arabidopsis thaliana ubiquitin carboxyl-terminal hydrolase-related protein mRNA, complete cds
AT4G24680	1.485171479	3.12E-103	Arabidopsis thaliana protein MODIFIER OF SNC1 1 mRNA, complete cds
AT5G55660	1.412830279	8.00E-102	Arabidopsis thaliana DEK domain-containing chromatin associated

			protein mRNA, complete cds
AT1G20390	3.976099557	2.65E-101	Arabidopsis thaliana chromosome 1 sequence
AT3G14172	1.920031463	6.67E-101	Arabidopsis thaliana uncharacterized protein mRNA, complete cds
AT2G46560	1.669544884	1.58E-100	Arabidopsis thaliana transducin family protein / WD-40 repeat family protein mRNA, complete cds
AT4G18600	3.152266367	2.29E-100	Arabidopsis thaliana SCAR family protein WAVE5 mRNA, complete cds
AT4G16870	2.290374559	8.81E-100	Arabidopsis thaliana chromosome 4 sequence
AT1G09770	1.021610263	1.55E-99	Arabidopsis thaliana cell division cycle 5-like protein mRNA, complete cds
AT4G16660	1.116397334	1.85E-98	Arabidopsis thaliana heat shock protein 70 mRNA, complete cds
AT4G39050	1.343368768	2.53E-98	Arabidopsis thaliana Kinesin motor family protein mRNA, complete cds
AT1G09750	1.004470657	5.38E-98	Arabidopsis thaliana aspartyl protease-like protein mRNA, complete cds
AT3G01320	2.036430378	8.22E-97	Arabidopsis thaliana paired amphipathic helix protein Sin3-like 1 mRNA, complete cds
AT5G58040	1.50104678	4.25E-96	Arabidopsis thaliana FIP1 [V]-like protein mRNA, complete cds
AT1G19835	1.26045509	3.37E-95	Arabidopsis thaliana uncharacterized protein mRNA, complete cds
AT1G70060	1.902610991	1.36E-94	Arabidopsis thaliana paired amphipathic helix protein Sin3-like 4 mRNA, complete cds
AT2G38440	1.488519211	1.64E-94	Arabidopsis thaliana WAVE complex SCAR2 mRNA, complete cds
AT5G47820	1.342042356	2.01E-94	Arabidopsis thaliana kinesin-like protein FRA1 mRNA, complete cds
AT5G10470	1.166950412	3.89E-94	Arabidopsis thaliana kinesin CDKA;1 associated 1 mRNA, complete cds
AT3G17360	3.180646154	8.55E-94	Arabidopsis thaliana phragmoplast orienting kinesin 1 mRNA, complete cds
AT3G28550	1.559900023	9.21E-94	Arabidopsis thaliana chromosome 3, complete sequence
AT5G67470	1.948752772	1.83E-93	Arabidopsis thaliana formin-like protein 6 mRNA, complete cds
AT1G72410	1.266688982	9.17E-93	Arabidopsis thaliana COP1-interacting

			protein-related protein mRNA, complete cds
AT2G25660	1.60392924	2.94E-92	Arabidopsis thaliana embryo defective 2410 protein mRNA, complete cds
AT3G06400	1.043531875	5.35E-92	Arabidopsis thaliana chromatin-remodeling protein 11 mRNA, complete cds
AT3G50240	1.834102213	1.54E-91	Arabidopsis thaliana kinesin-related protein mRNA, complete cds
AT3G54590	1.74446876	2.46E-91	Arabidopsis thaliana hydroxyproline-rich glycoprotein mRNA, complete cds
AT5G46070	1.788575747	4.57E-90	Arabidopsis thaliana Guanylate-binding protein mRNA, complete cds
AT1G15940	2.106055051	6.79E-90	Arabidopsis thaliana aspartyl beta-hydroxylase N-terminal region domain-containing protein mRNA, complete cds
AT3G48190	1.724996952	1.36E-89	Arabidopsis thaliana serine/threonine-protein kinase ATM mRNA, complete cds
AT1G15780	1.809806008	4.47E-89	Arabidopsis thaliana uncharacterized protein mRNA, complete cds
AT1G67140	1.522800022	1.58E-88	Arabidopsis thaliana protein SWEETIE mRNA, complete cds
AT3G01460	2.166493791	2.41E-88	Arabidopsis thaliana methyl-CPG-binding domain 9 mRNA, complete cds
AT3G50380	1.571386326	2.61E-88	Arabidopsis thaliana uncharacterized protein mRNA, complete cds
AT2G25730	1.314895655	3.25E-88	Arabidopsis thaliana uncharacterized protein mRNA, complete cds
AT5G07740	1.598087768	5.17E-88	Arabidopsis thaliana chromosome 5 sequence
AT4G00800	1.41583627	5.87E-88	Arabidopsis thaliana transducin family protein / WD-40 repeat family protein mRNA, complete cds
AT3G51740	1.246500623	8.26E-88	Arabidopsis thaliana probably inactive leucine-rich repeat receptor-like protein kinase IMK2 mRNA, complete cds
AT4G26190	1.60389543	1.94E-87	Arabidopsis thaliana Haloacid dehalogenase-like hydrolase (HAD) superfamily protein mRNA, complete cds
AT5G42950	1.124809096	5.06E-87	Arabidopsis thaliana GYF domain-containing protein mRNA, complete cds

AT3G13300	1.035169622	6.24E-87	Arabidopsis thaliana mRNA decapping complex VCS mRNA, complete cds
AT3G62010	1.193929533	8.48E-86	Arabidopsis thaliana uncharacterized protein mRNA, complete cds
AT1G13220	2.30631342	2.27E-85	Arabidopsis thaliana protein CROWDED NUCLEI 2 mRNA, complete cds
AT5G44180	1.466839385	2.62E-85	Arabidopsis thaliana protein RINGLET2 mRNA, complete cds
AT5G43900	1.226949713	2.79E-85	Arabidopsis thaliana myosin 2 mRNA, complete cds
AT5G63420	1.353119402	1.69E-84	Arabidopsis thaliana RNase J mRNA, complete cds
AT1G77800	1.267646632	2.17E-84	Arabidopsis thaliana PHD finger-containing protein mRNA, complete cds
AT1G58230	1.636773841	1.51E-83	Arabidopsis thaliana WD40 and Beach domain-containing protein mRNA, complete cds
AT1G02730	1.308897598	1.58E-83	Arabidopsis thaliana cellulose synthase-like protein D5 mRNA, complete cds
AT3G54280	1.600349929	2.62E-83	Arabidopsis thaliana TATA-binding protein-associated factor BTAF1 mRNA, complete cds
AT2G41960	1.747669847	3.53E-83	Arabidopsis thaliana uncharacterized protein mRNA, complete cds
AT5G60930	2.40251314	1.18E-82	Arabidopsis thaliana P-loop containing nucleoside triphosphate hydrolases superfamily protein mRNA, complete cds
AT4G30100	1.248424705	1.32E-82	Arabidopsis thaliana P-loop containing nucleoside triphosphate hydrolases superfamily protein mRNA, complete cds
AT2G26330	1.080851021	2.89E-82	Arabidopsis thaliana LRR receptor-like serine/threonine-protein kinase ERECTA mRNA, complete cds
AT2G47800	1.142515762	3.94E-82	Arabidopsis thaliana ABC transporter C family member 4 mRNA, complete cds
AT1G24300	1.658685408	1.23E-80	Arabidopsis thaliana GYF domain-containing protein mRNA, complete cds
AT3G12810	2.143395724	2.10E-80	Arabidopsis thaliana photoperiod-independent early flowering 1 protein mRNA, complete cds
AT3G33530	1.20816135	2.72E-80	Arabidopsis thaliana transducin/WD40

			domain-containing protein mRNA, complete cds
AT3G06290	1.684239088	3.65E-80	Arabidopsis thaliana SAC3/GANP/Nin1/mts3/eIF-3 p25 family protein mRNA, complete cds
AT1G06670	1.644991308	1.82E-79	Arabidopsis thaliana nuclear DEIH-boxhelicase mRNA, complete cds
AT1G61210	1.65883284	3.73E-79	Arabidopsis thaliana protein DWD hypersensitive to ABA 3 mRNA, complete cds
AT2G20190	1.054478149	1.97E-78	Arabidopsis thaliana CLIP-associated protein mRNA, complete cds
AT5G43310	1.683421001	3.01E-78	Arabidopsis thaliana COP1-interacting protein-like protein mRNA, complete cds
AT3G12980	1.85427108	8.77E-78	Arabidopsis thaliana histone acetyltransferase HAC5 mRNA, complete cds
AT3G60860	1.441513763	5.10E-77	Arabidopsis thaliana SEC7-like guanine nucleotide exchange family protein mRNA, complete cds
AT5G51200	1.293654632	8.54E-77	Arabidopsis thaliana uncharacterized protein mRNA, complete cds
AT4G27430	1.155794885	8.07E-76	Arabidopsis thaliana COP1-interacting protein 7 mRNA, complete cds
AT4G30990	1.788755646	8.53E-76	Arabidopsis thaliana down-regulated in metastasis (DRIM) domain-containing protein mRNA, complete cds
AT5G24740	1.553613865	2.84E-75	Arabidopsis thaliana uncharacterized protein mRNA, complete cds
AT1G49340	1.168386861	2.94E-75	Arabidopsis thaliana phosphatidylinositol 4-kinase alpha mRNA, complete cds
AT4G16340	1.077113024	4.68E-75	Arabidopsis thaliana DOCK family guanine nucleotide exchange factor SPIKE1 mRNA, complete cds
AT2G48160	1.292640795	4.79E-75	Arabidopsis thaliana Tudor/PWWP/MBT domain-containing protein mRNA, complete cds
AT3G07770	1.043815978	6.19E-75	Arabidopsis thaliana HEAT SHOCK PROTEIN 89.1 mRNA, complete cds
AT1G32490	1.543839	1.59E-74	Arabidopsis thaliana DEAH RNA helicase homolog PRP2 mRNA, complete cds
AT3G57470	1.018078979	1.86E-74	Arabidopsis thaliana insulinase family protein mRNA, complete cds
AT5G41140	1.878386844	3.39E-74	Arabidopsis thaliana Myosin heavy

			chain-related protein mRNA, complete cds
AT4G22505	4.175734214	6.56E-74	Arabidopsis thaliana chromosome 4 sequence
AT2G03140	1.749202197	6.77E-74	Arabidopsis thaliana alpha/beta-Hydrolases superfamily protein mRNA, complete cds
AT2G47410	1.56504848	7.49E-74	Arabidopsis thaliana WD40 domain-containing protein mRNA, complete cds
AT2G36490	1.516090547	7.53E-74	Arabidopsis thaliana protein ROS1 mRNA, complete cds
AT1G19720	1.186447608	1.13E-73	Arabidopsis thaliana pentatricopeptide repeat-containing protein mRNA, complete cds
AT2G43800	1.931624391	4.55E-73	Arabidopsis thaliana formin-like protein 2 mRNA, complete cds
AT1G19715	1.136308734	1.43E-72	Arabidopsis thaliana Mannose-binding lectin superfamily protein mRNA, complete cds
AT5G56890	1.473099354	1.61E-72	Arabidopsis thaliana protein kinase family protein mRNA, complete cds
AT4G33240	1.313887463	1.84E-72	Arabidopsis thaliana 1-phosphatidylinositol-3-phosphate 5-kinase FAB1A mRNA, complete cds
AT1G56660	1.877500158	2.24E-72	Arabidopsis thaliana uncharacterized protein mRNA, complete cds
AT5G18620	1.457718727	9.15E-72	Arabidopsis thaliana chromatin remodeling factor17 mRNA, complete cds
AT1G71220	1.017092903	9.90E-72	Arabidopsis thaliana UDP-glucose:glycoprotein glucosyltransferase mRNA, complete cds
AT5G40480	1.50176828	5.56E-71	Arabidopsis thaliana protein EMBRYO DEFECTIVE 3012 mRNA, complete cds
AT2G31970	1.979694971	2.39E-70	Arabidopsis thaliana DNA repair protein RAD50 mRNA, complete cds
AT5G48600	2.180733022	3.34E-69	Arabidopsis thaliana structural maintenance of chromosomes protein 4 mRNA, complete cds
AT4G28710	1.419589066	5.69E-69	Arabidopsis thaliana myosin XI H mRNA, complete cds
AT3G14270	1.401379945	1.20E-68	Arabidopsis thaliana phosphatidylinositol-3P 5-kinase-like

			mRNA, complete cds
AT1G75310	2.31684677	4.66E-68	Arabidopsis thaliana auxin-like 1 protein mRNA, complete cds
AT4G31880	1.236621782	1.97E-67	Arabidopsis thaliana uncharacterized protein mRNA, complete cds
AT1G24190	1.506228922	2.16E-67	Arabidopsis thaliana paired amphipathic helix protein Sin3-like 3 mRNA, complete cds
AT5G40340	1.567485593	3.27E-67	Arabidopsis thaliana PWWP domain-containing protein mRNA, complete cds
AT3G63070	1.476515773	3.65E-67	Arabidopsis thaliana Tudor/PWWP/MBT domain-containing protein mRNA, complete cds
AT3G06880	1.604612913	4.26E-67	Arabidopsis thaliana transducin/WD-40 repeat-containing protein mRNA, complete cds
AT4G31160	1.607796782	5.76E-67	Arabidopsis thaliana DDB1- and CUL4-associated factor-1 mRNA, complete cds
AT3G13330	1.243818531	6.68E-67	Arabidopsis thaliana proteasome activating protein 200 mRNA, complete cds
AT4G25290	1.117456603	8.68E-67	Arabidopsis thaliana DNA photolyase mRNA, complete cds
AT1G78580	1.155469243	1.12E-65	Arabidopsis thaliana alpha,alpha-trehalose-phosphate synthase [UDP-forming] 1 mRNA, complete cds
AT5G55820	2.786424076	1.69E-65	Arabidopsis thaliana uncharacterized protein mRNA, complete cds
AT3G27260	1.166750487	2.08E-65	Arabidopsis thaliana global transcription factor group E8 mRNA, complete cds
AT3G10650	1.098576804	2.23E-65	Arabidopsis thaliana nucleoporin NUP1 mRNA, complete cds
AT3G24870	2.068683667	1.23E-64	Arabidopsis thaliana Helicase/SANT-associated, DNA binding protein mRNA, complete cds
AT2G34640	1.117108672	1.41E-64	Arabidopsis thaliana plastid transcriptionally active 12 mRNA, complete cds
AT3G29320	1.29830431	3.34E-64	Arabidopsis thaliana alpha-glucan phosphorylase 1 mRNA, complete cds
AT3G05680	1.118772595	4.81E-64	Arabidopsis thaliana embryo defective

			2016 protein mRNA, complete cds
AT5G42540	1.012228715	8.46E-64	Arabidopsis thaliana 5'-3' exoribonuclease 2 mRNA, complete cds
AT5G55300	1.020306033	9.38E-64	Arabidopsis thaliana DNA topoisomerase 1 mRNA, complete cds
AT2G19110	1.165969743	1.22E-63	Arabidopsis thaliana putative cadmium/zinc-transporting ATPase HMA4 mRNA, complete cds
AT5G18700	2.323120341	1.28E-63	Arabidopsis thaliana protein kinase family protein with ARM repeat domain mRNA, complete cds
AT1G08600	1.743350343	1.35E-63	Arabidopsis thaliana protein ATRX mRNA, complete cds
AT3G59930	1.778352888	1.38E-63	Arabidopsis thaliana defensin-like protein 206 mRNA, complete cds
AT1G17580	1.389532064	1.42E-63	Arabidopsis thaliana myosin 1 mRNA, complete cds
AT2G37080	1.295684213	1.72E-63	Arabidopsis thaliana protein ROP interactive partner 2 mRNA, complete cds
AT3G62900	1.536939174	3.33E-63	Arabidopsis thaliana CW-type zinc-finger protein mRNA, complete cds
AT2G21300	1.261958069	3.62E-62	Arabidopsis thaliana ATP binding microtubule motor family protein mRNA, complete cds
AT1G74160	1.495479697	5.43E-62	Arabidopsis thaliana uncharacterized protein mRNA, complete cds
AT5G16210	1.273385941	5.83E-62	Arabidopsis thaliana HEAT repeat-containing protein mRNA, complete cds
AT5G02310	1.144746972	1.80E-61	Arabidopsis thaliana proteolysis 6 mRNA, complete cds
AT5G16780	1.253663263	2.49E-61	Arabidopsis thaliana SART-1 family protein DOT2 mRNA, complete cds
AT1G18370	1.918552534	3.60E-61	Arabidopsis thaliana kinesin HINKEL mRNA, complete cds
AT5G24350	1.427521104	5.12E-61	Arabidopsis thaliana uncharacterized protein mRNA, complete cds
AT3G47890	1.485174688	5.93E-61	Arabidopsis thaliana ubiquitin carboxyl-terminal hydrolase-related protein mRNA, complete cds
AT3G57660	1.697958611	7.78E-61	Arabidopsis thaliana nuclear RNA polymerase A1 mRNA, complete cds
AT1G17110	1.300325641	8.97E-61	Arabidopsis thaliana ubiquitin

			carboxyl-terminal hydrolase 15 mRNA, complete cds
AT1G08060	1.982781339	1.23E-60	Arabidopsis thaliana helicase protein MOM1 mRNA, complete cds
AT4G33200	1.314760211	1.94E-60	Arabidopsis thaliana myosin-15 mRNA, complete cds
AT5G15680	1.386468818	2.26E-60	Arabidopsis thaliana cell morphogenesis domain-containing protein mRNA, complete cds
AT1G79560	1.009233603	3.45E-60	Arabidopsis thaliana AT1G79560 mRNA, complete cds, clone: RAFL07-08-E14
AT4G32420	1.299986344	4.67E-60	Arabidopsis thaliana cyclophilin-like peptidyl-prolyl cis-trans isomerase family protein mRNA, complete cds
AT1G79350	1.300997249	6.76E-60	Arabidopsis thaliana protein EMBRYO DEFECTIVE 1135 mRNA, complete cds
AT1G03910	1.26485833	7.70E-60	Arabidopsis thaliana uncharacterized protein mRNA, complete cds
AT1G31810	2.100598099	7.97E-60	Arabidopsis thaliana formin-like protein 14 mRNA, complete cds
AT4G38760	1.128360191	9.75E-60	Arabidopsis thaliana uncharacterized protein mRNA, complete cds
AT3G54760	1.107267048	4.03E-59	Arabidopsis thaliana dentin sialophosphoprotein-related protein mRNA, complete cds
AT4G04350	1.114861901	4.94E-59	Arabidopsis thaliana tRNA synthetase class I (I, L, M and V) family protein mRNA, complete cds
AT4G23800	1.104888051	5.19E-59	Arabidopsis thaliana HMG (high mobility group) box protein mRNA, complete cds
AT3G45850	1.239441326	1.29E-58	Arabidopsis thaliana P-loop containing nucleoside triphosphate hydrolases superfamily protein mRNA, complete cds
AT1G13980	1.17138676	3.22E-58	Arabidopsis thaliana ARF guanine-nucleotide exchange factor GNOM mRNA, complete cds
AT5G12400	1.448829502	6.36E-58	Arabidopsis thaliana PHD-finger and DNA binding domain-containing protein mRNA, complete cds
AT2G39340	1.079468106	9.43E-58	Arabidopsis thaliana SAC3/GANP/Nin1/mts3/eIF-3 p25-family protein mRNA, complete cds
AT5G61190	1.483590398	2.10E-57	Arabidopsis thaliana putative endonuclease or glycosyl hydrolase with

			C2H2-type zinc finger domain mRNA, complete cds
AT5G64170	1.199120101	2.98E-57	Arabidopsis thaliana dentin sialophosphoprotein-like protein mRNA, complete cds
AT1G06720	1.545691728	3.38E-57	Arabidopsis thaliana BMS1 domain-containing protein mRNA, complete cds
AT4G27595	2.344265416	5.68E-57	Arabidopsis thaliana uncharacterized protein mRNA, complete cds
AT1G45160	1.266872128	7.03E-57	Arabidopsis thaliana protein kinase mRNA, complete cds
AT5G47480	1.015976289	7.96E-57	Arabidopsis thaliana RGPR-related protein mRNA, complete cds
AT5G15540	1.52217449	1.07E-56	Arabidopsis thaliana sister-chromatid cohesion protein 2 mRNA, complete cds
AT5G01400	1.441426793	1.11E-56	Arabidopsis thaliana Symplekin/Pta1-like protein mRNA, complete cds
AT4G18670	1.354354692	1.79E-56	Arabidopsis thaliana chromosome 4 sequence
AT1G48650	1.090771777	5.93E-56	Arabidopsis thaliana DEA(D/H)-box RNA helicase family protein mRNA, complete cds
AT2G27170	1.353252905	6.50E-56	Arabidopsis thaliana structural maintenance of chromosome 3 mRNA, complete cds
AT4G12780	1.374632889	1.03E-55	Arabidopsis thaliana auxilin-related protein 1 mRNA, complete cds
AT3G19190	1.341572773	1.68E-55	Arabidopsis thaliana protein autophagy 2 mRNA, complete cds
AT4G19020	1.524554883	1.68E-55	Arabidopsis thaliana chromomethylase 2 mRNA, complete cds
AT4G32620	1.468742201	1.82E-55	Arabidopsis thaliana Enhancer of polycomb-like transcription factor protein mRNA, complete cds
AT1G72250	2.034016093	2.15E-55	Arabidopsis thaliana Di-glucose binding protein with Kinesin motor domain mRNA, complete cds
AT3G10010	2.29193305	2.25E-55	Arabidopsis thaliana putative DNA glycosylase mRNA, complete cds
AT5G16730	1.109437155	6.04E-55	Arabidopsis thaliana uncharacterized protein mRNA, complete cds
AT3G52250	1.255323895	2.72E-54	Arabidopsis thaliana protein POWERDRESS mRNA, complete cds

AT2G39260	1.264855	2.98E-54	Arabidopsis thaliana regulator of nonsense transcripts UPF2 mRNA, complete cds
AT2G24120	1.022144599	7.04E-54	Arabidopsis thaliana DNA-directed RNA polymerase 3 mRNA, complete cds
AT4G19530	1.034158699	1.10E-53	Arabidopsis thaliana TIR-NBS-LRR class disease resistance protein mRNA, complete cds
AT1G47900	1.55153655	1.65E-53	Arabidopsis thaliana uncharacterized protein mRNA, complete cds
AT1G77460	1.846796694	1.78E-53	Arabidopsis thaliana armadillo/beta-catenin-like repeat and C2 domain-containing protein mRNA, complete cds
AT3G18110	1.506970998	2.31E-53	Arabidopsis thaliana pentatricopeptide repeat-containing protein mRNA, complete cds
AT3G12020	1.340125685	7.16E-53	Arabidopsis thaliana kinesin motor protein-like protein mRNA, complete cds
AT1G11720	1.139552094	7.85E-53	Arabidopsis thaliana starch synthase 3 mRNA, complete cds
AT5G65440	1.211675519	6.46E-52	Arabidopsis thaliana uncharacterized protein mRNA, complete cds
AT3G26560	1.193757546	6.55E-52	Arabidopsis thaliana probable pre-mRNA-splicing factor ATP-dependent RNA helicase mRNA, complete cds
AT2G35110	1.297940629	7.48E-52	Arabidopsis thaliana protein NAP1 mRNA, complete cds
AT3G58110	1.223183806	1.13E-51	Arabidopsis thaliana uncharacterized protein mRNA, complete cds
AT5G16680	1.466486915	1.35E-51	Arabidopsis thaliana RING/FYVE/PHD zinc finger-containing protein mRNA, complete cds
AT5G07940	1.444227759	1.73E-51	Arabidopsis thaliana uncharacterized protein mRNA, complete cds
AT5G02880	1.098766052	3.35E-51	Arabidopsis thaliana E3 ubiquitin-protein ligase UPL4 mRNA, complete cds
AT4G12400	1.359254917	6.70E-51	Arabidopsis thaliana carboxylate clamp-tetratricopeptide repeat protein mRNA, complete cds
AT2G28620	1.740005952	8.03E-51	Arabidopsis thaliana kinesin motor protein-related protein mRNA, complete cds
AT2G22610	2.324571399	1.00E-50	Arabidopsis thaliana Di-glucose binding

			protein with Kinesin motor domain mRNA, complete cds
AT5G43810	1.085475048	1.39E-50	Arabidopsis thaliana eIF2C family protein Argonaute10 mRNA, complete cds
AT1G08260	1.874925602	2.07E-50	Arabidopsis thaliana DNA polymerase epsilon catalytic subunit A mRNA, complete cds
AT2G48060	1.196055895	4.91E-50	Arabidopsis thaliana uncharacterized protein mRNA, complete cds
AT1G80020	1.289071367	5.92E-50	Arabidopsis thaliana chromosome 1 sequence
AT5G04020	1.92832658	1.01E-49	Arabidopsis thaliana calmodulin-binding protein mRNA, complete cds
AT5G55040	1.081819806	1.39E-49	Arabidopsis thaliana DNA-binding bromodomain-containing protein mRNA, complete cds
AT5G22450	1.176363834	2.02E-49	Arabidopsis thaliana uncharacterized protein mRNA, complete cds
AT3G24880	2.179172387	2.07E-49	Arabidopsis thaliana Helicase/SANT-associated, DNA binding protein mRNA, complete cds
AT4G31210	1.344371583	2.74E-49	Arabidopsis thaliana DNA topoisomerase, type IA, core mRNA, complete cds
AT1G55325	1.250232127	5.85E-49	Arabidopsis thaliana RNA polymerase II transcription mediator mRNA, complete cds
AT1G74260	1.047332516	1.23E-48	Arabidopsis thaliana phosphoribosylformylglycinamidine synthase mRNA, complete cds
AT1G44910	1.415694161	1.43E-48	Arabidopsis thaliana pre-mRNA-processing protein 40A mRNA, complete cds
AT1G16270	1.184709658	5.80E-48	Arabidopsis thaliana octicosapeptide/Phox/Bem1p domain-containing protein kinase mRNA, complete cds
AT3G13290	1.688827146	5.92E-48	Arabidopsis thaliana varicose-related protein mRNA, complete cds
AT3G24480	1.083795032	5.97E-48	Arabidopsis thaliana chromosome 3, complete sequence
AT5G17910	1.284362414	8.24E-48	Arabidopsis thaliana uncharacterized protein mRNA, complete cds
AT3G20150	1.709286838	8.59E-48	Arabidopsis thaliana Kinesin motor family protein mRNA, complete cds

AT2G22795	1.062133528	2.70E-47	Arabidopsis thaliana uncharacterized protein mRNA, complete cds
AT2G23740	1.725366006	2.93E-47	Arabidopsis thaliana histone-lysine N-methyltransferase SUVR5 mRNA, complete cds
AT1G27430	1.127446588	3.27E-47	Arabidopsis thaliana GYF domain-containing protein mRNA, complete cds
AT3G54460	1.187724374	4.78E-47	Arabidopsis thaliana SNF2, helicase and F-box domain-containing protein mRNA, complete cds
AT3G23670	2.18158694	5.85E-47	Arabidopsis thaliana kinesin-like protein KIN12B mRNA, complete cds
AT5G54280	1.201649505	9.09E-47	Arabidopsis thaliana myosin 2 mRNA, complete cds
AT1G23230	1.401613767	9.13E-47	Arabidopsis thaliana mediator of RNA polymerase II transcription subunit 23 mRNA, complete cds
AT5G39500	1.460641567	9.50E-47	Arabidopsis thaliana ARF guanine-nucleotide exchange factor GNL1 mRNA, complete cds
AT1G16710	1.314751147	2.47E-46	Arabidopsis thaliana histone acetyltransferase HAC12 mRNA, complete cds
AT4G00440	1.059326941	5.15E-46	Arabidopsis thaliana uncharacterized protein mRNA, complete cds
AT5G27970	1.053946414	9.90E-46	Arabidopsis thaliana ARM repeat superfamily protein mRNA, complete cds
AT5G25060	1.061981837	1.31E-45	Arabidopsis thaliana RNA recognition motif-containing protein RRC1 mRNA, complete cds
AT5G49160	1.15594998	1.63E-45	Arabidopsis thaliana DNA (cytosine-5)-methyltransferase 1 mRNA, complete cds
AT5G16270	1.067504305	1.78E-45	Arabidopsis thaliana sister chromatid cohesion 1 protein 4 mRNA, complete cds
AT5G65930	1.138059565	4.32E-45	Arabidopsis thaliana kinesin-like calmodulin-binding protein ZWICHEL mRNA, complete cds
AT2G17820	1.073248258	6.38E-45	Arabidopsis thaliana histidine kinase 1 mRNA, complete cds
AT4G32820	1.561804886	1.01E-44	Arabidopsis thaliana tetratricopeptide repeat domain-containing protein mRNA,

			complete cds
AT4G16630	1.462531544	2.40E-44	Arabidopsis thaliana DEAD-box ATP-dependent RNA helicase 28 mRNA, complete cds
AT1G35660	2.05479441	4.19E-44	Arabidopsis thaliana uncharacterized protein mRNA, complete cds
AT4G03550	1.087040381	4.82E-44	Arabidopsis thaliana callose synthase 12 mRNA, complete cds
AT1G72390	1.370964304	6.71E-44	Arabidopsis thaliana protein PHYTOCHROME-DEPENDENT LATE-FLOWERING mRNA, complete cds
AT2G38770	1.277613828	7.79E-44	Arabidopsis thaliana embryo defective 2765 mRNA, complete cds
AT5G53440	1.090357269	9.96E-44	Arabidopsis thaliana uncharacterized protein mRNA, complete cds
AT3G59100	1.101887282	1.63E-43	Arabidopsis thaliana putative callose synthase 6 mRNA, complete cds
AT5G15580	1.468379926	1.73E-43	Arabidopsis thaliana protein longifolia1 mRNA, complete cds
AT5G01890	1.057496593	1.95E-43	Arabidopsis thaliana leucine-rich receptor-like protein kinase mRNA, complete cds
AT3G53540	1.372885593	2.01E-43	Arabidopsis thaliana uncharacterized protein mRNA, complete cds
AT5G38840	1.683964105	3.50E-43	Arabidopsis thaliana SMAD/FHA domain-containing protein mRNA, complete cds
AT1G55970	1.973000318	7.16E-43	Arabidopsis thaliana histone acetyltransferase of the CBP family 4 mRNA, complete cds
AT3G15120	1.681192212	9.54E-43	Arabidopsis thaliana P-loop containing nucleoside triphosphate hydrolases superfamily protein mRNA, complete cds
AT2G33435	1.956574771	9.84E-43	Arabidopsis thaliana RNA recognition motif-containing protein mRNA, complete cds
AT5G11530	1.599102233	1.36E-42	Arabidopsis thaliana embryonic flower 1 mRNA, complete cds
AT2G21440	1.155703262	1.99E-42	Arabidopsis thaliana RNA recognition motif-containing protein mRNA, complete cds
AT3G06530	1.271945903	4.32E-42	Arabidopsis thaliana U3snoRNP10 and NUC211 domain-containing protein mRNA, complete cds

AT1G55540	1.59819995	7.52E-42	Arabidopsis thaliana nuclear pore complex protein LNO1 mRNA, complete cds
AT5G67100	1.553715848	7.96E-42	Arabidopsis thaliana DNA polymerase alpha catalytic subunit mRNA, complete cds
AT4G36180	1.035917693	1.09E-41	Arabidopsis thaliana putative LRR receptor-like serine/threonine-protein kinase mRNA, complete cds
AT2G21380	1.227925655	1.29E-41	Arabidopsis thaliana kinesin motor family protein mRNA, complete cds
AT1G66980	1.019771008	2.23E-41	Arabidopsis thaliana suppressor of npr1-1 constitutive 4 mRNA, complete cds
AT5G42400	1.432892054	3.50E-41	Arabidopsis thaliana histone methyltransferase SDG25 mRNA, complete cds
AT3G21250	1.039691375	3.69E-41	Arabidopsis thaliana multidrug resistance-associated protein 6 mRNA, complete cds
AT5G45060	1.201369552	3.92E-41	Arabidopsis thaliana TIR-NBS-LRR class disease resistance protein mRNA, complete cds
AT2G36200	1.613371514	5.80E-41	Arabidopsis thaliana kinesin family protein mRNA, complete cds
AT5G13530	1.265494325	7.88E-41	Arabidopsis thaliana E3 ubiquitin-protein ligase KEG mRNA, complete cds
AT3G61780	1.520066211	2.89E-40	Arabidopsis thaliana protein embryo defective 1703 mRNA, complete cds
AT2G26570	1.061387313	4.81E-40	Arabidopsis thaliana coiled-coil protein WEB1 mRNA, complete cds
AT4G30790	1.268372803	5.06E-40	Arabidopsis thaliana uncharacterized protein mRNA, complete cds
AT4G29940	1.822343651	7.55E-40	Arabidopsis thaliana Pathogenesis-related homeodomain protein mRNA, complete cds
AT4G02400	1.443079181	8.98E-40	Arabidopsis thaliana U3 ribonucleoprotein family protein mRNA, complete cds
AT5G08230	1.660594998	1.05E-39	Arabidopsis thaliana Tudor/PWWP/MBT domain-containing protein mRNA, complete cds
AT2G25050	1.722965142	1.25E-39	Arabidopsis thaliana actin-binding FH2 protein mRNA, complete cds

AT3G22142	2.966574049	1.37E-39	Arabidopsis thaliana chromosome 3, complete sequence
AT4G12020	1.269449402	1.85E-39	Arabidopsis thaliana mitogen-activated protein kinase kinase kinase 11 mRNA, complete cds
AT4G02560	1.218112816	2.43E-39	Arabidopsis thaliana homeobox protein LUMINIDEPENDENS mRNA, complete cds
AT5G13590	1.059711952	2.55E-39	Arabidopsis thaliana uncharacterized protein mRNA, complete cds
AT2G34357	1.031271299	2.55E-39	Arabidopsis thaliana NUC173 domain-containing protein mRNA, complete cds
AT5G52280	1.318296746	2.59E-39	Arabidopsis thaliana Myosin heavy chain-related protein mRNA, complete cds
AT1G63300	1.964962999	2.71E-39	Arabidopsis thaliana Myosin heavy chain-related protein mRNA, complete cds
AT5G62410	1.744181628	7.41E-39	Arabidopsis thaliana structural maintenance of chromosome 2 mRNA, complete cds
AT3G10310	2.117390228	3.91E-38	Arabidopsis thaliana myosin and kinesin motor and CH domain-containing protein mRNA, complete cds
AT5G48360	1.066109723	4.36E-38	Arabidopsis thaliana actin-binding FH2 (formin homology 2) family protein mRNA, complete cds
AT5G65460	1.197161833	5.12E-38	Arabidopsis thaliana kinesin like protein for actin based chloroplast movement 2 mRNA, complete cds
AT1G17440	1.059476631	5.71E-38	Arabidopsis thaliana transcription initiation factor TFIID subunit 12B mRNA, complete cds
AT4G32730	1.169275522	6.60E-38	Arabidopsis thaliana myb-related protein 3R-1 mRNA, complete cds
AT1G73960	1.141803083	1.09E-37	Arabidopsis thaliana TBP-associated factor 2 mRNA, complete cds
AT5G55540	1.33892515	1.47E-37	Arabidopsis thaliana protein TORNADO 1 mRNA, complete cds
AT5G07980	1.380357081	2.29E-37	Arabidopsis thaliana dentin sialophosphoprotein-like protein mRNA, complete cds
AT1G14840	1.266840771	3.15E-37	Arabidopsis thaliana microtubule-associated protein 70-4

			mRNA, complete cds
AT1G01040	1.038411734	3.67E-37	Arabidopsis thaliana endoribonuclease Dicer-like 1 mRNA, complete cds
AT3G04740	1.080358679	8.20E-37	Arabidopsis thaliana mediator of RNA polymerase II transcription subunit 14 mRNA, complete cds
AT4G10930	1.946332368	1.93E-36	Arabidopsis thaliana uncharacterized protein mRNA, complete cds
AT2G40770	1.968621878	2.46E-36	Arabidopsis thaliana RING-finger, DEAD-like helicase, PHD and SNF2 domain-containing protein mRNA, complete cds
AT3G01810	1.002048851	3.93E-36	Arabidopsis thaliana uncharacterized protein mRNA, complete cds
AT3G58160	2.043099745	4.58E-36	Arabidopsis thaliana Class XI myosin mRNA, complete cds
AT1G27850	1.148571883	5.53E-36	Arabidopsis thaliana uncharacterized protein mRNA, complete cds
AT1G02990	1.665251159	6.33E-36	Arabidopsis thaliana uncharacterized protein mRNA, complete cds
AT1G15240	1.023433038	6.85E-36	Arabidopsis thaliana phox (PX) domain-containing protein mRNA, complete cds
AT5G45650	1.057328837	8.26E-36	Arabidopsis thaliana subtilase family protein mRNA, complete cds
AT5G62640	1.348439452	8.27E-36	Arabidopsis thaliana protein EARLY FLOWERING 5 mRNA, complete cds
AT3G44050	1.9362004	1.39E-35	Arabidopsis thaliana kinesin motor protein-like protein mRNA, complete cds
AT1G61850	1.060756488	1.73E-35	Arabidopsis thaliana galactolipase/ phospholipase mRNA, complete cds
AT4G02710	1.750333855	1.89E-35	Arabidopsis thaliana Kinase interacting (KIP1-like) family protein mRNA, complete cds
ATCG00190	1.755023188	2.14E-35	Arabidopsis thaliana chloroplast DNA, complete genome, ecotype: Columbia
AT1G02110	1.073470428	2.97E-35	Arabidopsis thaliana uncharacterized protein mRNA, complete cds
AT4G16310	1.308038496	5.23E-35	Arabidopsis thaliana protein LSD1-like 3 mRNA, complete cds
AT4G12770	1.05708721	7.00E-35	Arabidopsis thaliana auxilin-related protein 2 mRNA, complete cds
AT3G49500	1.097220255	7.09E-35	Arabidopsis thaliana RNA-dependent RNA polymerase 6 mRNA, complete cds

AT5G04290	1.609849561	1.05E-34	Arabidopsis thaliana kow domain-containing transcription factor 1 mRNA, complete cds
AT1G70070	1.095775505	3.13E-34	Arabidopsis thaliana DEAD-box ATP-dependent RNA helicase ISE2 mRNA, complete cds
AT3G17900	1.035960112	4.00E-34	Arabidopsis thaliana uncharacterized protein mRNA, complete cds
AT2G24650	1.184945952	5.59E-34	Arabidopsis thaliana B3 domain-containing protein REM13 mRNA, complete cds
AT4G00060	1.013019405	8.33E-34	Arabidopsis thaliana protein MATERNAL EFFECT EMBRYO ARREST 44 mRNA, complete cds
AT5G35750	1.09635601	1.22E-33	Arabidopsis thaliana histidine kinase 2 mRNA, complete cds
AT2G40030	2.185220935	1.37E-33	Arabidopsis thaliana nuclear RNA polymerase D1B mRNA, complete cds
AT3G28030	1.743369071	2.17E-33	Arabidopsis thaliana DNA repair protein UVH3 mRNA, complete cds
AT1G27595	1.043970746	2.36E-33	Arabidopsis thaliana symplekin tight junction protein domain-containing protein mRNA, complete cds
AT3G21430	1.349416524	2.47E-33	Arabidopsis thaliana protein ALWAYS EARLY 3 mRNA, complete cds
AT2G46240	2.227573146	4.03E-33	Arabidopsis thaliana BCL-2-associated athanogene 6 mRNA, complete cds
AT4G05190	1.337507602	4.36E-33	Arabidopsis thaliana kinesin 5 mRNA, complete cds
AT5G38150	1.010346451	4.37E-33	Arabidopsis thaliana plastid movement impaired protein 15 mRNA, complete cds
AT3G57060	1.643538551	4.51E-33	Arabidopsis thaliana putative condensin complex protein Cap-D2 mRNA, complete cds
AT5G66310	1.507162812	9.21E-33	Arabidopsis thaliana ATP binding microtubule motor family protein mRNA, complete cds
AT3G19670	1.003525186	9.75E-33	Arabidopsis thaliana pre-mRNA-processing protein 40B mRNA, complete cds
AT1G19220	1.509581234	1.09E-32	Arabidopsis thaliana auxin response factor 19 mRNA, complete cds
AT5G49430	1.305878713	1.56E-32	Arabidopsis thaliana WD40/YVTN repeat and Bromo-WDR9-I-like

			domain-containing protein mRNA, complete cds
AT4G00990	1.241196429	7.44E-32	Arabidopsis thaliana transcription factor jumonji (jmjC) domain-containing protein mRNA, complete cds
AT3G46960	1.237964751	8.74E-32	Arabidopsis thaliana DExD/H box RNA helicase mRNA, complete cds
AT1G76740	3.360730472	1.97E-31	Arabidopsis thaliana uncharacterized protein mRNA, complete cds
AT5G28400	2.270201327	2.47E-31	Arabidopsis thaliana uncharacterized protein mRNA, complete cds
AT2G46630	1.139024256	2.99E-31	Arabidopsis thaliana chromosome 2, complete sequence
AT1G79730	1.064053137	3.38E-31	Arabidopsis thaliana protein early flowering 7 mRNA, complete cds
AT5G23080	1.126327246	4.79E-31	Arabidopsis thaliana TATA-box binding protein-interacting protein TOUGH mRNA, complete cds
AT1G63640	1.184895875	8.01E-31	Arabidopsis thaliana microtubule motor protein KinG mRNA, complete cds
AT2G20290	1.753170411	1.15E-30	Arabidopsis thaliana myosin-like protein XIG mRNA, complete cds
AT3G51150	1.458427146	1.17E-30	Arabidopsis thaliana ATP binding microtubule motor family protein mRNA, complete cds
AT3G43690	1.243455664	1.21E-30	Arabidopsis thaliana chromosome 3, complete sequence
AT3G14570	1.244162605	1.46E-30	Arabidopsis thaliana glucan synthase-like 4 mRNA, complete cds
AT5G04240	1.145453154	4.76E-30	Arabidopsis thaliana probable lysine-specific demethylase ELF6 mRNA, complete cds
AT3G14980	1.388037818	7.38E-30	Arabidopsis thaliana histone H3 acetyltransferase IDM1 mRNA, complete cds
AT1G64570	1.079488753	1.06E-29	Arabidopsis thaliana DUO pollen 3 protein mRNA, complete cds
AT3G16840	1.007693037	1.22E-29	Arabidopsis thaliana P-loop containing nucleoside triphosphate hydrolases superfamily protein mRNA, complete cds
AT1G34355	2.381151432	1.34E-29	Arabidopsis thaliana parallel Spindle 1 protein mRNA, complete cds
AT3G43920	1.798258301	1.47E-29	Arabidopsis thaliana endoribonuclease Dicer-like 3 mRNA, complete cds

AT1G06490	1.726034281	1.63E-29	Arabidopsis thaliana callose synthase 7 mRNA, complete cds
AT5G27240	1.75834896	1.98E-29	Arabidopsis thaliana chromosome 5 sequence
AT1G63100	1.101270562	2.03E-29	Arabidopsis thaliana chromosome 1 sequence
AT5G44870	1.06174453	2.26E-29	Arabidopsis thaliana TIR-NBS-LRR class disease resistance protein LAZ5 mRNA, complete cds
AT1G19485	1.127825507	2.64E-29	Arabidopsis thaliana transducin/WD-40 repeat-containing protein mRNA, complete cds
AT2G48110	1.125406907	3.66E-29	Arabidopsis thaliana protein REDUCED EPIDERMAL FLUORESCENCE 4 mRNA, complete cds
AT1G72440	1.017611205	3.74E-29	Arabidopsis thaliana protein SLOW WALKER2 mRNA, complete cds
AT1G77030	1.155160911	5.43E-29	Arabidopsis thaliana putative DEAD-box ATP-dependent RNA helicase 29 mRNA, complete cds
AT4G02020	1.024768927	6.43E-29	Arabidopsis thaliana histone-lysine N-methyltransferase EZA1 mRNA, complete cds
AT3G20010	1.361865761	7.25E-29	Arabidopsis thaliana RING finger-related, SNF2 and helicase domain-containing protein mRNA, complete cds
AT3G06480	1.186054875	1.04E-28	Arabidopsis thaliana DEAD-box ATP-dependent RNA helicase 40 mRNA, complete cds
AT4G29790	1.05941507	1.20E-28	Arabidopsis thaliana uncharacterized protein mRNA, complete cds
AT1G77580	1.296763842	1.27E-28	Arabidopsis thaliana filament-like plant protein 1 mRNA, complete cds
AT1G62310	1.190654821	1.59E-28	Arabidopsis thaliana transcription factor jumonji domain-containing protein mRNA, complete cds
AT1G50240	1.356727388	1.84E-28	Arabidopsis thaliana serine/threonine-protein kinase FUSED mRNA, complete cds
AT5G58160	1.538698245	2.02E-28	Arabidopsis thaliana formin-like protein 13 mRNA, complete cds
AT3G51120	1.046056496	5.21E-28	Arabidopsis thaliana zinc finger CCCH domain-containing protein 44 mRNA, complete cds

AT2G27980	1.002435978	6.59E-28	Arabidopsis thaliana acyl-CoA N-acyltransferase with RING/FYVE/PHD-type zinc finger domain-containing protein mRNA, complete cds
AT5G44660	1.000146461	1.08E-27	Arabidopsis thaliana uncharacterized protein mRNA, complete cds
AT5G42920	1.061448346	1.35E-27	Arabidopsis thaliana THO complex, subunit 5 mRNA, complete cds
AT5G19310	1.419746677	1.37E-27	Arabidopsis thaliana homeotic protein regulator mRNA, complete cds
AT3G56410	1.669993085	1.73E-27	Arabidopsis thaliana uncharacterized protein mRNA, complete cds
AT1G61690	1.047847516	1.82E-27	Arabidopsis thaliana phosphoinositide binding protein mRNA, complete cds
AT5G48310	1.586941886	1.83E-27	Arabidopsis thaliana uncharacterized protein mRNA, complete cds
AT5G22010	1.121151086	2.17E-27	Arabidopsis thaliana replication factor C1 mRNA, complete cds
AT4G22485	2.731992219	2.27E-27	Arabidopsis thaliana chromosome 4 sequence
AT1G76720	1.485149153	2.86E-27	Arabidopsis thaliana eukaryotic translation initiation factor 2 (eIF-2) family protein mRNA, complete cds
AT1G07400	1.17647017	3.18E-27	Arabidopsis thaliana chromosome 1 sequence
AT4G04970	1.007657968	8.00E-27	Arabidopsis thaliana glucan synthase-like 1 mRNA, complete cds
AT2G30800	1.235267537	8.20E-27	Arabidopsis thaliana protein helicase in vascular tissue and tapetum mRNA, complete cds
AT3G07540	1.193757546	8.26E-27	Arabidopsis thaliana Actin-binding FH2 (formin homology 2) family protein mRNA, complete cds
AT5G51600	1.384380794	8.27E-27	Arabidopsis thaliana microtubule associated protein MAP65-3 mRNA, complete cds
AT1G58060	1.04737316	8.63E-27	Arabidopsis thaliana RNA helicase family protein mRNA, complete cds
AT4G27370	1.675650246	1.11E-26	Arabidopsis thaliana myosin heavy chain-like protein mRNA, complete cds
AT2G02480	1.380714477	1.21E-26	Arabidopsis thaliana protein STICHEL mRNA, complete cds
AT2G36350	1.535263771	1.28E-26	Arabidopsis thaliana protein kinase

			mRNA, complete cds
AT1G55250	1.148409628	1.37E-26	Arabidopsis thaliana E3 ubiquitin-protein ligase HUB2 mRNA, complete cds
AT5G28320	2.17463418	1.88E-26	Arabidopsis thaliana uncharacterized protein mRNA, complete cds
AT1G30410	1.046873504	4.10E-26	Arabidopsis thaliana multidrug resistance-associated protein 13 mRNA, complete cds
AT5G26860	1.042262981	4.23E-26	Arabidopsis thaliana lon protease 1 mRNA, complete cds
AT3G44730	1.563343374	4.59E-26	Arabidopsis thaliana kinesin-like protein 1 mRNA, complete cds
AT1G77600	1.278494971	5.33E-26	Arabidopsis thaliana ARM repeat superfamily protein mRNA, complete cds
AT1G21160	1.005849198	5.58E-26	Arabidopsis thaliana eukaryotic translation initiation factor 2 (eIF-2) family protein mRNA, complete cds
AT3G48430	1.071180712	6.11E-26	Arabidopsis thaliana lysine-specific demethylase REF6 mRNA, complete cds
AT1G19850	1.100939707	6.24E-26	Arabidopsis thaliana auxin response factor 5 mRNA, complete cds
AT2G43900	1.015158535	1.56E-25	Arabidopsis thaliana 5-inositol-phosphate phosphatase mRNA, complete cds
AT2G19950	1.010968169	1.59E-25	Arabidopsis thaliana golgin candidate 1 mRNA, complete cds
AT4G01020	1.671609155	1.68E-25	Arabidopsis thaliana zinc finger-related and helicase and IBR domain-containing protein mRNA, complete cds
AT1G63020	1.996031287	2.21E-25	Arabidopsis thaliana DNA-directed RNA polymerase IV subunit 1 mRNA, complete cds
AT2G34780	1.388037818	2.27E-25	Arabidopsis thaliana protein MATERNAL EFFECT EMBRYO ARREST 22 mRNA, complete cds
AT3G45830	1.2781602	3.37E-25	Arabidopsis thaliana uncharacterized protein mRNA, complete cds
AT1G21170	1.074043904	4.63E-25	Arabidopsis thaliana exocyst complex component SEC5B mRNA, complete cds
AT3G12915	1.602655095	6.76E-25	Arabidopsis thaliana ribosomal protein S5/Elongation factor G/III/V family protein mRNA, complete cds
AT5G60040	1.688827146	8.01E-25	Arabidopsis thaliana nuclear RNA polymerase C1 mRNA, complete cds

AT4G24610	1.272291917	8.33E-25	Arabidopsis thaliana uncharacterized protein mRNA, complete cds
AT3G02890	1.236034724	8.45E-25	Arabidopsis thaliana RING/FYVE/PHD zinc finger-related protein mRNA, complete cds
AT1G17450	1.640803888	1.25E-24	Arabidopsis thaliana B-block binding subunit of TFIIIC mRNA, complete cds
AT1G03830	1.823253199	1.76E-24	Arabidopsis thaliana guanylate-binding protein mRNA, complete cds
AT1G16800	1.851769574	4.30E-24	Arabidopsis thaliana P-loop containing nucleoside triphosphate hydrolases superfamily protein mRNA, complete cds
AT3G23780	1.074473196	7.09E-24	Arabidopsis thaliana nuclear RNA polymerase D2A mRNA, complete cds
AT2G13970	1.146191047	1.17E-23	Arabidopsis thaliana chromosome 2, complete sequence
AT1G10450	1.063343289	1.38E-23	Arabidopsis thaliana paired amphipathic helix protein Sin3-like 6 mRNA, complete cds
AT1G34047	4.34409447	1.63E-23	Arabidopsis thaliana defensin-like protein 208 mRNA, complete cds
AT3G55160	1.414429317	1.75E-23	Arabidopsis thaliana uncharacterized protein mRNA, complete cds
AT2G33440	1.959194519	1.92E-23	Arabidopsis thaliana RNA recognition motif-containing protein mRNA, complete cds
AT1G79150	1.269982056	2.76E-23	Arabidopsis thaliana nucleolar complex-associated protein domain-containing protein mRNA, complete cds
AT3G02930	1.651072224	2.93E-23	Arabidopsis thaliana uncharacterized protein mRNA, complete cds
AT1G13940	1.138755025	1.01E-22	Arabidopsis thaliana uncharacterized protein mRNA, complete cds
AT1G49870	1.645403474	1.41E-22	Arabidopsis thaliana uncharacterized protein mRNA, complete cds
AT5G54650	1.425391232	1.55E-22	Arabidopsis thaliana formin-like protein 5 mRNA, complete cds
AT1G03780	1.097136618	1.81E-22	Arabidopsis thaliana protein TPX2 mRNA, complete cds
AT4G27010	1.687141478	2.14E-22	Arabidopsis thaliana uncharacterized protein mRNA, complete cds
AT1G12040	1.591738084	3.15E-22	Arabidopsis thaliana chromosome 1 sequence

AT4G02660	2.351511942	3.35E-22	Arabidopsis thaliana Beige/BEACH and WD40 domain-containing protein mRNA, complete cds
AT3G42670	1.449438362	3.55E-22	Arabidopsis thaliana chromatin remodeling 38 mRNA, complete cds
AT1G07910	1.29391564	3.89E-22	Arabidopsis thaliana tRNA ligase mRNA, complete cds
AT3G44200	1.171145194	5.76E-22	Arabidopsis thaliana serine/threonine-protein kinase Nek5 mRNA, complete cds
AT3G21290	1.115805625	8.18E-22	Arabidopsis thaliana dentin sialophosphoprotein-like protein mRNA, complete cds
AT5G17880	1.063100973	8.32E-22	Arabidopsis thaliana TIR-NBS-LRR class disease resistance protein mRNA, complete cds
AT5G42140	1.245895973	1.47E-21	Arabidopsis thaliana Regulator of chromosome condensation (RCC1) family with FYVE zinc finger domain mRNA, complete cds
AT3G57980	1.407146641	1.53E-21	Arabidopsis thaliana DNA-binding bromodomain-containing protein mRNA, complete cds
AT4G32710	1.489791317	1.55E-21	Arabidopsis thaliana proline-rich receptor-like protein kinase PERK14 mRNA, complete cds
AT4G14150	2.004709178	1.63E-21	Arabidopsis thaliana phragmoplast-associated kinesin-related protein 1 mRNA, complete cds
AT2G37420	1.419746677	1.77E-21	Arabidopsis thaliana ATP binding microtubule motor family protein mRNA, complete cds
AT5G48610	1.412285364	2.18E-21	Arabidopsis thaliana uncharacterized protein mRNA, complete cds
AT4G13750	1.838699227	2.27E-21	Arabidopsis thaliana nuclear factor NO VEIN mRNA, complete cds
AT4G38070	1.848802591	2.52E-21	Arabidopsis thaliana transcription factor bHLH131 mRNA, complete cds
AT4G11130	1.803075317	2.56E-21	Arabidopsis thaliana RNA-dependent RNA polymerase 2 mRNA, complete cds
AT5G23480	2.057889216	3.40E-21	Arabidopsis thaliana SWIB/MDM2, Plus-3 and GYF domain-containing protein mRNA, complete cds
AT1G20720	2.074473196	3.59E-21	Arabidopsis thaliana RAD3-like

			DNA-binding helicase protein mRNA, complete cds
AT5G37190	1.222451752	3.96E-21	Arabidopsis thaliana COP1-interacting protein 4 mRNA, complete cds
AT2G34730	1.160437105	8.55E-21	Arabidopsis thaliana myosin heavy chain-related mRNA, complete cds
AT5G46400	1.33646747	9.21E-21	Arabidopsis thaliana pre-mRNA-processing factor 39 mRNA, complete cds
AT5G47490	1.089514327	1.07E-20	Arabidopsis thaliana RGPR-related protein mRNA, complete cds
AT1G10930	1.383139018	2.18E-20	Arabidopsis thaliana ATP-dependent DNA helicase Q-like 4A mRNA, complete cds
AT4G25280	1.032378866	2.70E-20	Arabidopsis thaliana probably adenylate kinase mRNA, complete cds
AT1G20670	1.004459253	4.38E-20	Arabidopsis thaliana DNA-binding bromodomain-containing protein mRNA, complete cds
AT3G60160	1.321669008	5.85E-20	Arabidopsis thaliana multidrug resistance-associated protein 9 mRNA, complete cds
AT4G26660	1.175734214	6.57E-20	Arabidopsis thaliana uncharacterized protein mRNA, complete cds
AT3G47460	1.380491009	8.00E-20	Arabidopsis thaliana structural maintenance of chromosomes protein 2-2 mRNA, complete cds
AT1G66730	1.01143263	1.03E-19	Arabidopsis thaliana DNA ligase 6 mRNA, complete cds
AT2G36480	1.198717218	1.16E-19	Arabidopsis thaliana ENTH/VHS-like protein mRNA, complete cds
AT1G33390	1.167893461	1.29E-19	Arabidopsis thaliana protein FASCIATED STEM 4 mRNA, complete cds
AT1G67530	1.05610255	1.39E-19	Arabidopsis thaliana ARM repeat superfamily protein mRNA, complete cds
AT5G37630	1.377877952	1.40E-19	Arabidopsis thaliana protein EMBRYO DEFECTIVE 2656 mRNA, complete cds
AT5G10370	1.708216131	1.54E-19	Arabidopsis thaliana helicase , IBR and zinc finger protein domain-containing protein mRNA, complete cds
AT1G04160	1.375213777	2.45E-19	Arabidopsis thaliana myosin XI B mRNA, complete cds
AT5G49680	1.504993964	2.69E-19	Arabidopsis thaliana SABRE-like protein mRNA, complete cds
AT5G53020	1.155544806	2.90E-19	Arabidopsis thaliana ribonuclease P

			protein subunit P38-like protein mRNA, complete cds
AT3G22400	1.323267562	3.72E-19	Arabidopsis thaliana lipoxygenase 5 mRNA, complete cds
AT4G12010	1.241628741	4.55E-19	Arabidopsis thaliana TIR-NBS-LRR class disease resistance protein mRNA, complete cds
AT2G33240	2.525541342	5.97E-19	Arabidopsis thaliana myosin XI D mRNA, complete cds
AT4G21270	1.286915893	6.26E-19	Arabidopsis thaliana kinesin-like motor protein heavy chain mRNA, complete cds
AT1G23935	2.703539643	6.67E-19	Arabidopsis thaliana uncharacterized protein mRNA, complete cds
AT2G36255	2.5135687	1.03E-18	Arabidopsis thaliana putative defensin-like protein 203 mRNA, complete cds
AT5G61460	1.489321154	1.88E-18	Arabidopsis thaliana protein SMC6B mRNA, complete cds
AT5G55520	1.237187497	1.97E-18	Arabidopsis thaliana uncharacterized protein mRNA, complete cds
AT5G40820	2.236034724	2.25E-18	Arabidopsis thaliana serine/threonine-protein kinase ATR mRNA, complete cds
AT1G36180	1.680819567	2.79E-18	Arabidopsis thaliana acetyl-CoA carboxylase 2 mRNA, complete cds
AT2G28240	1.517618779	2.89E-18	Arabidopsis thaliana ATP-dependent helicase-like protein mRNA, complete cds
AT2G31900	1.285200781	3.31E-18	Arabidopsis thaliana myosin-like protein XIF mRNA, complete cds
AT4G25120	1.730361133	3.52E-18	Arabidopsis thaliana helicase SRS2-like protein mRNA, complete cds
AT3G10180	2.400010459	5.80E-18	Arabidopsis thaliana kinesin motor protein-related protein mRNA, complete cds
AT3G03340	1.037681315	6.78E-18	Arabidopsis thaliana protein UNFERTILIZED EMBRYO SAC 6 mRNA, complete cds
AT4G00930	1.472734982	1.31E-17	Arabidopsis thaliana COP1-interacting protein 4.1 mRNA, complete cds
AT5G15070	1.528900354	1.48E-17	Arabidopsis thaliana phosphoglycerate mutase-like protein mRNA, complete cds
AT2G31320	1.260196775	1.99E-17	Arabidopsis thaliana poly [ADP-ribose] polymerase 2 mRNA, complete cds
AT1G14460	1.074014921	2.72E-17	Arabidopsis thaliana AAA-type ATPase

			family protein mRNA, complete cds
AT4G32350	1.259493697	5.03E-17	Arabidopsis thaliana regulator of Vps4 activity protein mRNA, complete cds
AT1G28010	1.032600461	5.36E-17	Arabidopsis thaliana ABC transporter B family member 14 mRNA, complete cds
AT5G26160	1.29391564	5.39E-17	Arabidopsis thaliana uncharacterized protein mRNA, complete cds
AT5G63950	1.018923037	5.60E-17	Arabidopsis thaliana protein chromatin remodeling 24 mRNA, complete cds
AT3G15550	1.177701414	5.76E-17	Arabidopsis thaliana uncharacterized protein mRNA, complete cds
AT4G32700	1.403467858	6.02E-17	Arabidopsis thaliana MUS308 and mammalian DNA polymerase-like protein mRNA, complete cds
AT4G29440	1.190342634	6.32E-17	Arabidopsis thaliana regulator of Vps4 activity protein mRNA, complete cds
AT4G08115	3.153572564	7.01E-17	Arabidopsis thaliana chromosome 4 sequence
AT3G49160	1.161058127	7.29E-17	Arabidopsis thaliana pyruvate kinase-like protein mRNA, complete cds
AT4G34900	1.185573389	8.89E-17	Arabidopsis thaliana xanthine dehydrogenase 2 mRNA, complete cds
AT4G14330	1.173750446	9.81E-17	Arabidopsis thaliana phragmoplast-associated kinesin-related protein 2 mRNA, complete cds
AT1G26130	1.19570263	2.02E-16	Arabidopsis thaliana putative phospholipid-transporting ATPase 12 mRNA, complete cds
AT3G05470	1.427747528	2.08E-16	Arabidopsis thaliana Actin-binding protein FH2 mRNA, complete cds
AT3G18100	1.414033026	2.15E-16	Arabidopsis thaliana myb domain protein 4r1 mRNA, complete cds
AT1G09470	1.485823709	2.99E-16	Arabidopsis thaliana uncharacterized protein mRNA, complete cds
AT1G80810	1.357664169	3.45E-16	Arabidopsis thaliana Tudor/PWWP/MBT superfamily protein mRNA, complete cds
AT2G23360	1.234709327	3.57E-16	Arabidopsis thaliana filament-like plant protein 7 mRNA, complete cds
AT4G32970	1.252522847	4.58E-16	Arabidopsis thaliana uncharacterized protein mRNA, complete cds
AT1G62970	1.261125705	4.69E-16	Arabidopsis thaliana chromosome 1 sequence
AT4G16680	1.167749985	5.09E-16	Arabidopsis thaliana putative RNA helicase mRNA, complete cds

AT4G08100	2.698377938	6.28E-16	Arabidopsis thaliana chromosome 4 sequence
AT4G36120	1.493280978	7.40E-16	Arabidopsis thaliana uncharacterized protein mRNA, complete cds
AT4G02110	1.560874415	8.03E-16	Arabidopsis thaliana transcription coactivator protein mRNA, complete cds
AT1G60860	1.061809585	1.08E-15	Arabidopsis thaliana ADP-ribosylation factor GTPase-activating protein AGD2 mRNA, complete cds
AT5G38140	1.094124099	2.23E-15	Arabidopsis thaliana nuclear factor Y, subunit C12 mRNA, complete cds
AT1G76780	2.216669399	3.11E-15	Arabidopsis thaliana chromosome 1 sequence
AT5G07180	1.196068317	3.36E-15	Arabidopsis thaliana LRR receptor-like serine/threonine-protein kinase ERL2 mRNA, complete cds
AT1G68725	1.868170345	5.49E-15	Arabidopsis thaliana arabinogalactan protein 19 mRNA, complete cds
AT5G52310	1.121702049	5.64E-15	Arabidopsis thaliana protein LOW-TEMPERATURE-INDUCED 78 mRNA, complete cds
AT3G22760	1.0085587	6.81E-15	Arabidopsis thaliana CXC domain containing TSO1-like protein 1 mRNA, complete cds
AT2G15820	1.001258579	6.90E-15	Arabidopsis thaliana organelle transcript processing 51 mRNA, complete cds
AT4G17610	1.127948977	7.30E-15	Arabidopsis thaliana tRNA/rRNA methyltransferase (SpoU) family protein mRNA, complete cds
AT5G45520	3.236034724	7.78E-15	Arabidopsis thaliana chromosome 5 sequence
AT3G13440	1.507815173	8.04E-15	Arabidopsis thaliana S-adenosyl-L-methionine-dependent methyltransferase-like protein mRNA, complete cds
AT1G20060	1.730689392	8.44E-15	Arabidopsis thaliana ATP binding microtubule motor family protein mRNA, complete cds
AT5G10800	1.789592088	8.86E-15	Arabidopsis thaliana RNA recognition motif (RRM)-containing protein mRNA, complete cds
AT5G52410	1.227264515	1.20E-14	Arabidopsis thaliana uncharacterized protein mRNA, complete cds
AT4G04223	3.525541342	1.26E-14	Arabidopsis thaliana ARM repeat

			superfamily protein mRNA, complete cds
AT3G18090	1.305936738	1.42E-14	Arabidopsis thaliana DNA-directed RNA polymerase D subunit 2b mRNA, complete cds
AT3G14460	2.177141035	1.44E-14	Arabidopsis thaliana chromosome 3, complete sequence
AT1G77270	1.214208362	1.99E-14	Arabidopsis thaliana uncharacterized protein mRNA, complete cds
AT2G24700	1.170446383	2.73E-14	Arabidopsis thaliana transcriptional factor B3 family protein mRNA, complete cds
AT3G55060	1.690600588	2.78E-14	Arabidopsis thaliana uncharacterized protein mRNA, complete cds
AT4G22970	1.691429961	4.94E-14	Arabidopsis thaliana separase-like protein mRNA, complete cds
AT4G16970	1.492374478	6.72E-14	Arabidopsis thaliana protein kinase superfamily protein mRNA, complete cds
AT5G07660	1.788575747	7.80E-14	Arabidopsis thaliana structural maintenance of chromosomes 6A mRNA, complete cds
AT4G19430	1.436672593	7.91E-14	Arabidopsis thaliana chromosome 4 sequence
AT3G07210	1.147246486	8.60E-14	Arabidopsis thaliana uncharacterized protein mRNA, complete cds
AT5G02810	1.262506936	8.61E-14	Arabidopsis thaliana pseudo-response regulator 7 mRNA, complete cds
AT5G28390	1.703539643	1.01E-13	Arabidopsis thaliana RNA recognition motif-containing protein mRNA, complete cds
AT1G75150	1.661056312	1.03E-13	Arabidopsis thaliana uncharacterized protein mRNA, complete cds
AT3G49650	1.297735424	1.09E-13	Arabidopsis thaliana P-loop containing nucleoside triphosphate hydrolase-like protein mRNA, complete cds
AT1G15160	1.850381032	1.22E-13	Arabidopsis thaliana MATE efflux family protein mRNA, complete cds
AT5G63960	1.003178467	1.28E-13	Arabidopsis thaliana DNA polymerase delta subunit 1 mRNA, complete cds
AT4G01490	1.867564043	1.36E-13	Arabidopsis thaliana chromosome 4 sequence
AT1G58561	1.171644137	1.82E-13	Arabidopsis thaliana chromosome 1 sequence
AT3G43210	1.055985506	2.01E-13	Arabidopsis thaliana kinesin TETRASPORE mRNA, complete cds

AT5G60150	1.594488695	2.05E-13	Arabidopsis thaliana uncharacterized protein mRNA, complete cds
AT1G08840	1.486952844	2.11E-13	Arabidopsis thaliana embryo defective protein 2411 mRNA, complete cds
AT5G15920	1.188828881	2.43E-13	Arabidopsis thaliana structural maintenance of chromosomes 5 mRNA, complete cds
AT2G45900	1.641018559	2.44E-13	Arabidopsis thaliana phosphatidylinositol N-acetyglucosaminlytransferase subunit P-like protein mRNA, complete cds
AT4G21820	1.49292439	2.50E-13	Arabidopsis thaliana binding / calmodulin binding protein mRNA, complete cds
AT2G19930	1.216669399	3.34E-13	Arabidopsis thaliana probable RNA-dependent RNA polymerase 5 mRNA, complete cds
AT4G18490	2.632456545	3.92E-13	Arabidopsis thaliana uncharacterized protein mRNA, complete cds
AT5G52230	1.392096034	4.64E-13	Arabidopsis thaliana methyl-CPG-binding domain protein 13 mRNA, complete cds
AT5G12100	1.169203216	5.56E-13	Arabidopsis thaliana chromosome 5 sequence
AT5G24280	1.58350894	5.57E-13	Arabidopsis thaliana gamma-irradiation and mitomycin c induced 1 mRNA, complete cds
AT4G02070	1.082788464	6.49E-13	Arabidopsis thaliana DNA mismatch repair protein MSH6 mRNA, complete cds
AT2G46980	1.388037818	8.17E-13	Arabidopsis thaliana ASYNAPTIC 3 mRNA, complete cds
AT3G51290	1.451763415	1.05E-12	Arabidopsis thaliana protein ALTERED PHOSPHATE STARVATION RESPONSE 1 mRNA, complete cds
AT2G45460	1.348509454	1.23E-12	Arabidopsis thaliana SMAD/FHA domain-containing protein mRNA, complete cds
AT2G12490	1.520675586	1.81E-12	Arabidopsis thaliana chromosome 2, complete sequence
AT3G42640	1.009926887	2.63E-12	Arabidopsis thaliana H(+)-ATPase 8 mRNA, complete cds
AT4G14970	1.5434315	2.96E-12	Arabidopsis thaliana uncharacterized protein mRNA, complete cds
AT2G16390	1.038939522	3.00E-12	Arabidopsis thaliana putative chromatin remodeling protein mRNA, complete cds
AT2G35350	1.066109723	3.03E-12	Arabidopsis thaliana protein phosphatase

			2C 29 mRNA, complete cds
AT2G35340	1.170446383	3.72E-12	Arabidopsis thaliana protein MATERNAL EFFECT EMBRYO ARREST 29 mRNA, complete cds
AT5G07810	1.549431962	6.20E-12	Arabidopsis thaliana SNF2 and helicase domain-containing protein mRNA, complete cds
AT3G58650	1.078182555	6.39E-12	Arabidopsis thaliana uncharacterized protein mRNA, complete cds
AT4G00380	1.353686313	7.93E-12	Arabidopsis thaliana XH/XS domain-containing protein mRNA, complete cds
AT5G57250	1.01682882	1.02E-11	Arabidopsis thaliana pentatricopeptide repeat-containing protein mRNA, complete cds
AT5G39580	1.28578776	1.14E-11	Arabidopsis thaliana peroxidase 62 mRNA, complete cds
AT1G10270	1.054235623	1.25E-11	Arabidopsis thaliana chromosome 1 sequence
AT2G30480	1.135151367	1.35E-11	Arabidopsis thaliana uncharacterized protein mRNA, complete cds
AT1G50890	1.066109723	1.79E-11	Arabidopsis thaliana microtubule-associated protein SPIRAL2-like mRNA, complete cds
AT2G47230	1.025792268	2.20E-11	Arabidopsis thaliana uncharacterized protein mRNA, complete cds
AT3G48770	2.803075317	3.50E-11	Arabidopsis thaliana ATP/DNA binding protein mRNA, complete cds
AT2G18760	1.019567137	3.64E-11	Arabidopsis thaliana chromatin remodeling 8 mRNA, complete cds
AT1G29560	1.945815489	3.96E-11	Arabidopsis thaliana zinc finger C-x8-C-x5-C-x3-H type family protein mRNA, complete cds
AT1G16330	1.342234128	5.16E-11	Arabidopsis thaliana cyclin B3-1 mRNA, complete cds
AT4G24450	1.176533713	5.45E-11	Arabidopsis thaliana phosphoglucan, water dikinase mRNA, complete cds
AT4G19890	1.231918616	6.70E-11	Arabidopsis thaliana Pentatricopeptide repeat domain-containing protein mRNA, complete cds
AT2G24350	1.677544435	6.88E-11	Arabidopsis thaliana RNA recognition motif-containing protein mRNA, complete cds
AT3G53800	1.018015435	8.01E-11	Arabidopsis thaliana hsp70-interacting

			protein FES1B-like protein mRNA, complete cds
AT2G31340	1.1602319	8.84E-11	Arabidopsis thaliana protein embryo defective 1381 mRNA, complete cds
AT1G56120	1.24897378	1.01E-10	Arabidopsis thaliana Leucine-rich repeat transmembrane protein kinase mRNA, complete cds
AT1G65470	1.000656661	1.34E-10	Arabidopsis thaliana chromatin assembly factor 1 subunit FAS1 mRNA, complete cds
AT5G61940	2.103584428	1.57E-10	Arabidopsis thaliana ubiquitin carboxyl-terminal hydrolase-related protein mRNA, complete cds
AT4G22860	1.256212606	1.80E-10	Arabidopsis thaliana cell cycle regulated microtubule associated protein mRNA, complete cds
AT1G59540	1.021909919	1.93E-10	Arabidopsis thaliana kinesin-like protein mRNA, complete cds
AT2G01750	1.039878181	2.05E-10	Arabidopsis thaliana microtubule-associated protein 70-3 mRNA, complete cds
AT1G22770	1.5135687	2.54E-10	Arabidopsis thaliana protein GIGANTEA mRNA, complete cds
AT1G76820	1.198560019	2.72E-10	Arabidopsis thaliana translation initiation factor 2 (eIF-2) family protein mRNA, complete cds
AT4G17000	1.103977908	3.37E-10	Arabidopsis thaliana uncharacterized protein mRNA, complete cds
AT3G28153	3.838699227	3.69E-10	Arabidopsis thaliana chromosome 3, complete sequence
AT4G16960	1.282146649	3.96E-10	Arabidopsis thaliana TIR-NBS-LRR class disease resistance protein mRNA, complete cds
AT3G13100	1.0455484	4.03E-10	Arabidopsis thaliana ABC transporter C family member 7 mRNA, complete cds
AT3G62455	2.5135687	4.23E-10	Arabidopsis thaliana chromosome 3, complete sequence
AT3G21480	1.088477536	4.34E-10	Arabidopsis thaliana BRCT domain-containing DNA repair protein mRNA, complete cds
AT1G55200	1.399093006	4.64E-10	Arabidopsis thaliana protein kinase protein with adenine nucleotide alpha hydrolases-like domain mRNA, complete cds

AT3G20200	1.55153655	4.90E-10	Arabidopsis thaliana Protein kinase protein with adenine nucleotide alpha hydrolases-like domain mRNA, complete cds
AT1G69830	1.058949931	5.78E-10	Arabidopsis thaliana alpha-amylase-like 3 mRNA, complete cds
AT3G20440	1.025467738	6.88E-10	Arabidopsis thaliana putative glycoside hydrolase mRNA, complete cds
AT4G15810	1.012670464	8.55E-10	Arabidopsis thaliana P-loop containing nucleoside triphosphate hydrolases superfamily protein mRNA, complete cds
AT5G15700	1.226574395	1.34E-09	Arabidopsis thaliana DNA-directed RNA polymerase 2 mRNA, complete cds
ATCG00180	1.734488232	1.89E-09	Arabidopsis lyrata subsp. lyrata RNA polymerase beta subunit-1, mRNA
AT4G34060	1.734488232	1.89E-09	Arabidopsis thaliana DEMETER-like protein 3 mRNA, complete cds
AT3G18730	1.400010459	2.08E-09	Arabidopsis thaliana protein BRUSHY 1 mRNA, complete cds
AT1G48120	1.099276587	2.27E-09	Arabidopsis thaliana serine/threonine-protein phosphatase 7 long form homolog mRNA, complete cds
AT2G15810	2.18158694	2.29E-09	Arabidopsis thaliana chromosome 2, complete sequence
AT5G27890	1.058332372	2.82E-09	Arabidopsis thaliana uncharacterized protein mRNA, complete cds
AT5G52910	1.170905913	2.87E-09	Arabidopsis thaliana protein TIMELESS mRNA, complete cds
AT1G53160	1.066109723	3.06E-09	Arabidopsis thaliana squamosa promoter binding protein-like 4 mRNA, complete cds
AT5G46880	1.0157362	3.06E-09	Arabidopsis thaliana homeobox-leucine zipper protein HDG5 mRNA, complete cds
AT5G28330	1.988107211	3.09E-09	Arabidopsis thaliana chromosome 5 sequence
AT1G14090	1.223651	3.42E-09	Arabidopsis thaliana chromosome 1 sequence
AT5G22750	1.213666911	4.86E-09	Arabidopsis thaliana mRNA for hypothetical protein, complete cds, clone: RAFL16-05-A16
AT2G42920	1.074377339	4.91E-09	Arabidopsis thaliana pentatricopeptide repeat-containing protein mRNA, complete cds

AT1G15830	1.131065165	6.13E-09	Arabidopsis thaliana chromosome 1 sequence
AT1G02065	1.319866315	6.72E-09	Arabidopsis thaliana squamosa promoter binding protein-like 8 mRNA, complete cds
AT1G65920	1.178319226	7.67E-09	Arabidopsis thaliana Regulator of chromosome condensation (RCC1) family with FYVE zinc finger domain mRNA, complete cds
AT3G66652	1.288502144	9.67E-09	Arabidopsis thaliana fip1 motif-containing protein mRNA, complete cds
AT3G20280	1.112206137	9.76E-09	Arabidopsis thaliana PHD finger protein mRNA, complete cds
AT5G02430	1.334042928	1.18E-08	Arabidopsis thaliana transducin/WD40 domain-containing protein mRNA, complete cds
AT1G11160	1.185050796	1.68E-08	Arabidopsis thaliana WD40 domain-containing protein mRNA, complete cds
AT4G21070	1.093943931	1.88E-08	Arabidopsis thaliana protein BREAST CANCER SUSCEPTIBILITY 1-like protein mRNA, complete cds
AT1G52030	1.075098506	1.97E-08	Arabidopsis thaliana myrosinase-binding protein 2 mRNA, complete cds
AT1G21810	1.233219709	2.31E-08	Arabidopsis thaliana uncharacterized protein mRNA, complete cds
AT4G37490	1.002684343	2.47E-08	Arabidopsis thaliana cyclin-B1-1 mRNA, complete cds
AT1G13790	1.331699834	2.51E-08	Arabidopsis thaliana SGS3-like protein FDM4 mRNA, complete cds
AT4G16910	2.589671679	2.70E-08	Arabidopsis thaliana chromosome 4 sequence
AT4G36515	1.585483882	3.04E-08	Arabidopsis thaliana uncharacterized protein mRNA, complete cds
AT2G22560	1.057176598	3.06E-08	Arabidopsis thaliana Kinase interacting (KIP1-like) family protein mRNA, complete cds
AT3G05130	1.257052506	3.50E-08	Arabidopsis thaliana chromosome 3, complete sequence
AT4G05631	1.301326185	4.39E-08	Arabidopsis thaliana uncharacterized protein mRNA, complete cds
AT5G44620	1.245079864	5.03E-08	Arabidopsis thaliana cytochrome P450, family 706, subfamily A, polypeptide 3

			mRNA, complete cds
AT1G54575	1.651072224	5.60E-08	Arabidopsis thaliana chromosome 1 sequence
AT1G45120	1.107332386	6.65E-08	Arabidopsis thaliana chromosome 1 sequence
AT3G11000	1.973000318	7.31E-08	Arabidopsis thaliana DCD (Development and Cell Death) domain protein mRNA, complete cds
AT3G06630	2.120557507	8.77E-08	Arabidopsis thaliana protein kinase family protein mRNA, complete cds
AT5G27895	2.120557507	8.77E-08	Arabidopsis thaliana chromosome 5 sequence
AT3G05415	2.18158694	9.05E-08	Arabidopsis thaliana chromosome 3, complete sequence
AT3G04690	1.220832318	1.03E-07	Arabidopsis thaliana receptor-like protein kinase ANXUR1 mRNA, complete cds
AT3G44690	1.504230835	1.38E-07	Arabidopsis thaliana ecotype Bla-1 DM2A, DM2B, DM2C, DM2D, DM2E, DM2F, DM2G, and DM2H genes, complete cds
AT5G05940	1.046744398	1.39E-07	Arabidopsis thaliana ROP guanine nucleotide exchange factor 5 mRNA, complete cds
AT2G05440	1.249331547	1.90E-07	Arabidopsis thaliana glycine-rich protein 9 mRNA, complete cds
AT2G19920	1.372771061	1.97E-07	Arabidopsis thaliana RNA-dependent RNA polymerase family protein mRNA, complete cds
AT4G34400	1.179567773	1.98E-07	Arabidopsis thaliana AP2/B3-like transcriptional factor family protein mRNA, complete cds
AT2G15880	1.066109723	2.00E-07	Arabidopsis thaliana pollen-specific leucine-rich repeat extensin-like protein 3 mRNA, complete cds
AT3G49142	2.066109723	2.41E-07	Arabidopsis thaliana putative pentatricopeptide repeat-containing protein mRNA, complete cds
AT1G52450	2.125003412	2.53E-07	Arabidopsis thaliana ubiquitin carboxyl-terminal hydrolase-related protein mRNA, complete cds
AT1G15165	1.055767779	2.75E-07	Arabidopsis thaliana U-box domain protein mRNA, complete cds
AT4G38180	1.088136029	2.84E-07	Arabidopsis thaliana protein FAR1-related sequence 5 mRNA,

			complete cds
AT4G29990	1.30314892	2.90E-07	Arabidopsis thaliana leucine-rich repeat transmembrane protein kinase mRNA, complete cds
AT5G35914	2.873464645	3.01E-07	Arabidopsis thaliana chromosome 5 sequence
AT4G19050	1.201039303	3.08E-07	Arabidopsis thaliana NB-ARC domain-containing disease resistance protein mRNA, complete cds
AT1G09050	1.469465417	3.14E-07	Arabidopsis thaliana uncharacterized protein mRNA, complete cds
AT1G30290	1.112652309	3.28E-07	Arabidopsis thaliana chromosome 1 sequence
AT1G26760	1.335296356	3.52E-07	Arabidopsis thaliana chromosome 1 sequence
AT3G24982	1.203613247	3.76E-07	Arabidopsis thaliana receptor like protein 40 mRNA, complete cds
AT2G17490	3.820997225	3.83E-07	Arabidopsis thaliana chromosome 2, complete sequence
AT2G36026	3.820997225	3.83E-07	Arabidopsis thaliana chromosome 2, complete sequence
AT1G25175	5.589671679	4.52E-07	Arabidopsis thaliana At1g25170/F4F7_2 mRNA, complete cds
AT2G12440	5.589671679	4.52E-07	Arabidopsis thaliana chromosome 2, complete sequence
AT4G13650	1.414033026	4.92E-07	Arabidopsis thaliana pentatricopeptide repeat-containing protein mRNA, complete cds
AT2G05510	3.339128217	5.76E-07	Arabidopsis thaliana glycine-rich protein mRNA, complete cds
AT1G11100	1.229048294	5.78E-07	Arabidopsis thaliana SNF2 , helicase and zinc-finger domain-containing protein mRNA, complete cds
AT1G23940	1.651072224	5.85E-07	Arabidopsis thaliana ARM repeat superfamily protein mRNA, complete cds
AT3G26050	1.273705142	6.09E-07	Arabidopsis thaliana TPX2 (targeting protein for Xklp2) family protein mRNA, complete cds
AT5G61300	1.102329911	6.80E-07	Arabidopsis thaliana uncharacterized protein mRNA, complete cds
AT4G15890	1.014394686	7.88E-07	Arabidopsis thaliana condensation complex subunit 1 domain-containing protein mRNA, complete cds
ATCG00170	1.887139582	9.01E-07	Arabidopsis thaliana chloroplast DNA,

			complete genome, ecotype: Columbia
AT5G12030	1.651072224	1.05E-06	Arabidopsis thaliana chromosome 5 sequence
AT1G60930	1.012867702	1.18E-06	Arabidopsis thaliana RECQ helicase L4B mRNA, complete cds
AT1G09040	1.468208166	1.20E-06	Arabidopsis thaliana uncharacterized protein mRNA, complete cds
AT5G27230	2.351511942	1.27E-06	Arabidopsis thaliana Frigida-like protein mRNA, complete cds
AT1G35530	1.53077799	1.33E-06	Arabidopsis thaliana helicase FANCM mRNA, complete cds
AT3G24715	1.118577143	1.33E-06	Arabidopsis thaliana octicosapeptide/Phox/Bem1p domain-containing protein kinase mRNA, complete cds
AT3G04980	1.436947418	1.51E-06	Arabidopsis thaliana chromosome 3, complete sequence
AT5G38910	4.525541342	1.55E-06	Arabidopsis thaliana putative germin-like protein subfamily 1 member 9 mRNA, complete cds
AT4G30130	1.091644815	1.72E-06	Arabidopsis thaliana uncharacterized protein mRNA, complete cds
AT5G05510	1.105638087	1.85E-06	Arabidopsis thaliana Mad3/BUB1 homology region 1 mRNA, complete cds
AT4G13880	1.407146641	1.88E-06	Arabidopsis thaliana receptor like protein 48 mRNA, complete cds
AT5G01550	1.794030177	2.08E-06	Arabidopsis thaliana chromosome 5 sequence
AT3G44765	2.136499051	2.13E-06	Arabidopsis thaliana Full-length cDNA Complete sequence from clone GSLTPGH55ZA10 of Hormone Treated Callus of strain col-0 of Arabidopsis thaliana (thale cress)
AT5G26170	1.46096934	2.15E-06	Arabidopsis thaliana putative WRKY transcription factor 50 mRNA, complete cds
AT1G26330	1.261125705	2.76E-06	Arabidopsis thaliana DNA binding protein mRNA, complete cds
AT5G48060	1.157257611	3.35E-06	Arabidopsis thaliana C2 calcium/lipid-binding plant phosphoribosyltransferase family protein mRNA, complete cds
AT3G07273	2.032942859	3.36E-06	Arabidopsis thaliana chromosome 3, complete sequence

AT3G28770	1.359840926	3.42E-06	Arabidopsis thaliana uncharacterized protein mRNA, complete cds
AT5G11470	1.325496352	3.44E-06	Arabidopsis thaliana protein ANTI-SILENCING 1 mRNA, complete cds
AT5G02950	1.197354256	3.70E-06	Arabidopsis thaliana chromosome 5 sequence
AT1G05950	1.10875406	4.14E-06	Arabidopsis thaliana uncharacterized protein mRNA, complete cds
AT1G59218	1.200410815	4.51E-06	Arabidopsis thaliana putative disease resistance protein RDL6/RF9 mRNA, complete cds
AT3G51490	1.998995527	5.55E-06	Arabidopsis thaliana tonoplast monosaccharide transporter3 mRNA, complete cds
AT1G49940	1.431759195	5.78E-06	Arabidopsis thaliana uncharacterized protein mRNA, complete cds
AT5G45720	1.066109723	6.06E-06	Arabidopsis thaliana AAA-type ATPase family protein mRNA, complete cds
AT5G12020	1.813343652	6.73E-06	Arabidopsis thaliana chromosome 5 sequence
AT5G38870	1.052689207	6.83E-06	Arabidopsis thaliana chromosome 5 sequence
AT4G09750	1.027635575	7.03E-06	Arabidopsis thaliana mRNA for hypothetical protein, complete cds, clone: RAFL14-08-C18
AT1G77310	1.288502144	7.31E-06	Arabidopsis thaliana uncharacterized protein mRNA, complete cds
AT2G30750	1.128845478	8.10E-06	Arabidopsis thaliana cytochrome P450 71A12 mRNA, complete cds
AT1G48580	1.907411977	8.34E-06	Arabidopsis thaliana uncharacterized protein mRNA, complete cds
AT3G12970	1.329144129	8.92E-06	Arabidopsis thaliana chromosome 3, complete sequence
AT3G23370	1.531773295	9.17E-06	Arabidopsis thaliana RNA recognition motif-containing protein mRNA, complete cds
AT5G61980	1.080756499	9.81E-06	Arabidopsis thaliana ADP-ribosylation factor GTPase-activating protein AGD1 mRNA, complete cds
AT5G25045	4.314037236	1.01E-05	Arabidopsis thaliana chromosome 5 sequence
AT3G07274	1.301737971	1.08E-05	Arabidopsis thaliana chromosome 3, complete sequence
AT4G30700	1.489321154	1.18E-05	Arabidopsis thaliana chromosome 4

			sequence
AT2G35160	1.097136618	1.29E-05	Arabidopsis thaliana histone-lysine N-methyltransferase, H3 lysine-9 specific SUVH5 mRNA, complete cds
AT1G26540	1.038095347	1.41E-05	Arabidopsis thaliana agenet domain-containing protein mRNA, complete cds
AT1G15825	1.19650636	1.43E-05	Arabidopsis thaliana chromosome 1 sequence
AT5G08250	2.766549441	1.49E-05	Arabidopsis thaliana cytochrome P450 superfamily protein mRNA, complete cds
AT3G29590	1.222228925	1.49E-05	Arabidopsis thaliana chromosome 3, complete sequence
AT5G34825	3.458427146	1.59E-05	Arabidopsis thaliana chromosome 5 sequence
AT1G35112	2.55153655	1.69E-05	Arabidopsis thaliana chromosome 1 sequence
AT5G21150	1.750607897	1.71E-05	Arabidopsis thaliana argonaute 9 mRNA, complete cds
AT3G09480	2.153572564	1.81E-05	Arabidopsis thaliana chromosome 3, complete sequence
AT2G24680	1.425190816	2.23E-05	Arabidopsis thaliana B3 domain-containing protein REM12 mRNA, complete cds
AT5G66940	1.49906913	2.52E-05	Arabidopsis thaliana chromosome 5 sequence
AT3G58770	1.953634994	2.67E-05	Arabidopsis thaliana uncharacterized protein mRNA, complete cds
AT4G31610	1.757987428	3.10E-05	Arabidopsis thaliana B3 domain-containing protein REM1 mRNA, complete cds
AT5G40100	1.035736074	3.17E-05	Arabidopsis thaliana TIR-NBS-LRR class disease resistance protein mRNA, complete cds
AT5G27250	1.455152014	3.21E-05	Arabidopsis thaliana chromosome 5 sequence
AT5G57320	1.241196429	3.29E-05	Arabidopsis thaliana putative villin mRNA, complete cds
AT1G07390	1.188100247	3.71E-05	Arabidopsis thaliana receptor like protein 1 mRNA, complete cds
AT1G65120	1.472102083	3.83E-05	Arabidopsis thaliana ubiquitin carboxyl-terminal hydrolase-related protein mRNA, complete cds
AT3G27730	1.854605618	3.95E-05	Arabidopsis thaliana DNA helicase mRNA,

			complete cds
AT3G01970	1.428679802	4.82E-05	Arabidopsis thaliana WRKY DNA-binding protein 45 mRNA, complete cds
AT1G64625	1.00598873	4.86E-05	Arabidopsis thaliana transcription factor bHLH157 mRNA, complete cds
AT3G13090	1.168471441	5.28E-05	Arabidopsis thaliana multidrug resistance-associated protein 8 mRNA, complete cds
AT2G01510	1.510894566	5.43E-05	Arabidopsis thaliana chromosome 2, complete sequence
AT1G09080	3.314037236	5.50E-05	Arabidopsis thaliana protein BIP3 mRNA, complete cds
AT1G12210	1.123442898	5.74E-05	Arabidopsis thaliana chromosome 1 sequence
AT3G02030	1.004709178	5.95E-05	Arabidopsis thaliana transferase mRNA, complete cds
AT4G00890	1.258754801	5.95E-05	Arabidopsis thaliana chromosome 4 sequence
AT4G13410	1.388037818	6.00E-05	Arabidopsis thaliana putative mannan synthase 15 mRNA, complete cds
AT5G27220	4.066109723	6.71E-05	Arabidopsis thaliana Frigida-like protein mRNA, complete cds
AT3G48900	1.199376254	6.78E-05	Arabidopsis thaliana single-stranded DNA endonuclease family protein mRNA, complete cds
AT2G34390	1.873464645	7.12E-05	Arabidopsis thaliana aquaporin NIP2-1 mRNA, complete cds
AT5G62230	1.003373968	7.29E-05	Arabidopsis thaliana LRR receptor-like serine/threonine-protein kinase ERL1 mRNA, complete cds
AT1G74150	1.203613247	8.30E-05	Arabidopsis thaliana kelch motif-containing protein mRNA, complete cds
AT5G49390	1.126651265	8.62E-05	Arabidopsis thaliana uncharacterized protein mRNA, complete cds
AT2G38720	1.361565606	8.91E-05	Arabidopsis thaliana microtubule-associated protein 65-5 mRNA, complete cds
AT5G40010	1.314037236	8.95E-05	Arabidopsis thaliana chromosome 5 sequence
AT3G02330	1.776603106	0.000102364	Arabidopsis thaliana pentatricopeptide repeat-containing protein mRNA, complete cds
AT4G24580	1.066109723	0.000103965	Arabidopsis thaliana Rho GTPase

			activating protein REN1 mRNA, complete cds
AT5G25970	1.651072224	0.000122792	Arabidopsis thaliana core-2/I-branching beta-1,6-N-acetylglucosaminyltransferase family protein mRNA, complete cds
AT3G57965	3.973000318	0.000126726	Arabidopsis thaliana chromosome 3, complete sequence
AT5G23155	3.973000318	0.000126726	Arabidopsis thaliana chromosome 5 sequence
AT2G33760	2.803075317	0.000131199	Arabidopsis thaliana chromosome 2, complete sequence
AT1G04930	1.08600928	0.000138066	Arabidopsis thaliana hydroxyproline-rich glycoprotein family protein mRNA, complete cds
AT2G17770	1.55153655	0.00013877	Arabidopsis thaliana bZIP transcription factor FD-like protein mRNA, complete cds
AT3G30720	1.690600588	0.000142308	Arabidopsis thaliana qua-quine starch mRNA, complete cds
AT3G18020	1.185408651	0.000145273	Arabidopsis thaliana chromosome 3, complete sequence
AT1G64250	2.066109723	0.000150934	Arabidopsis thaliana chromosome 1 sequence
AT3G28945	1.218112816	0.000151735	Arabidopsis thaliana chromosome 3, complete sequence
AT1G69710	2.18158694	0.000157215	Arabidopsis thaliana regulator of chromosome condensation and FYVE zinc finger domain-containing protein mRNA, complete cds
AT3G18550	2.18158694	0.000157215	Arabidopsis thaliana transcription factor TCP18 mRNA, complete cds
AT4G11070	2.329144129	0.000157556	Arabidopsis thaliana putative WRKY transcription factor 41 mRNA, complete cds
AT1G05490	1.735961121	0.000163637	Arabidopsis thaliana chromatin remodeling 31 mRNA, complete cds
AT1G64572	1.735961121	0.000163637	Arabidopsis thaliana chromosome 1 sequence
AT4G35640	1.580682896	0.000163713	Arabidopsis thaliana serine acetyltransferase 3;2 mRNA, complete cds
AT2G19120	1.0470009	0.000176864	Arabidopsis thaliana P-loop containing nucleoside triphosphate hydrolases superfamily protein mRNA, complete cds

AT1G62440	1.189492138	0.000177932	Arabidopsis thaliana leucine-rich repeat extensin-like protein 2 mRNA, complete cds
AT2G26700	1.189492138	0.000177932	Arabidopsis thaliana protein kinase PINOID 2 mRNA, complete cds
AT3G14395	1.788575747	0.000186354	Arabidopsis thaliana uncharacterized protein mRNA, complete cds
AT3G27640	1.134281226	0.000195107	Arabidopsis thaliana transducin/WD40 repeat-like superfamily protein mRNA, complete cds
AT4G01270	1.028635017	0.000198844	Arabidopsis thaliana RING/U-box superfamily protein mRNA, complete cds
AT4G11480	1.850381032	0.000209677	Arabidopsis thaliana putative cysteine-rich receptor-like protein kinase 32 mRNA, complete cds
AT3G24780	1.046480916	0.000217205	Arabidopsis thaliana chromosome 3, complete sequence
AT5G03790	1.651072224	0.000224416	Arabidopsis thaliana homeobox 51 mRNA, complete cds
AT3G25013	1.066109723	0.000236244	Arabidopsis thaliana Synaptobrevin family protein mRNA, complete cds
AT1G20400	2.729074736	0.000237107	Arabidopsis thaliana uncharacterized protein mRNA, complete cds
AT1G48660	2.458427146	0.000263945	Arabidopsis thaliana auxin-responsive GH3 family protein mRNA, complete cds
AT5G28415	2.125003412	0.000266934	Arabidopsis thaliana chromosome 5 sequence
AT5G64060	2.125003412	0.000266934	Arabidopsis thaliana NAC domain containing protein 103 mRNA, complete cds
AT5G01335	2.125003412	0.000266934	Arabidopsis thaliana chromosome 5 sequence
AT3G57970	2.267743584	0.000272331	Arabidopsis thaliana Emsy N Terminus and plant Tudor-like domain-containing protein mRNA, complete cds
AT1G02670	1.279103446	0.000284661	Arabidopsis thaliana P-loop containing nucleoside triphosphate hydrolases superfamily protein mRNA, complete cds
AT5G59720	1.279103446	0.000284661	Arabidopsis thaliana chromosome 5 sequence
AT5G23000	1.57512337	0.000299285	Arabidopsis thaliana transcription factor RAX1 mRNA, complete cds
AT2G13130	1.008006768	0.00033581	Arabidopsis thaliana chromosome 2, complete sequence

AT5G07570	3.066109723	0.000350595	Arabidopsis thaliana glycine/proline-rich protein mRNA, complete cds
AT3G01319	3.066109723	0.000350595	Arabidopsis thaliana chromosome 3, complete sequence
ATCG00760	3.066109723	0.000350595	Olimarabidopsis pumila chloroplast DNA, complete sequence
AT5G60630	4.651072224	0.000359338	Arabidopsis thaliana chromosome 5 sequence
AT1G69400	1.089193336	0.000386186	Arabidopsis thaliana transducin/WD-40 repeat-containing protein mRNA, complete cds
AT1G04700	1.209067677	0.000400905	Arabidopsis thaliana PB1 domain-containing protein tyrosine kinase mRNA, complete cds
AT4G31070	1.651072224	0.000411292	Arabidopsis thaliana chromosome 4 sequence
AT3G46230	1.651072224	0.000411292	Arabidopsis thaliana chromosome 3, complete sequence
AT4G31020	1.066109723	0.000438721	Arabidopsis thaliana alpha/beta-Hydrolases superfamily protein mRNA, complete cds
AT3G11385	2.066109723	0.000450776	Arabidopsis thaliana chromosome 3, complete sequence
ATCG00160	3.766549441	0.000455101	Arabidopsis thaliana chloroplast DNA, complete genome, ecotype: Columbia
AT5G19810	2.203613247	0.000468318	Arabidopsis thaliana chromosome 5 sequence
AT1G10417	1.698377938	0.000476995	Arabidopsis thaliana uncharacterized protein mRNA, complete cds
AT1G22275	1.698377938	0.000476995	Arabidopsis thaliana synaptonemal complex protein 2 mRNA, complete cds
AT5G08600	1.147029718	0.000544979	Arabidopsis thaliana U3 ribonucleoprotein (Utp) family protein mRNA, complete cds
AT1G61080	1.043389646	0.000609878	Arabidopsis thaliana hydroxyproline-rich glycoprotein-like protein mRNA, complete cds
AT3G03480	1.267743584	0.00062043	Arabidopsis thaliana acetyl CoA:(Z)-3-hexen-1-ol acetyltransferase mRNA, complete cds
AT2G15800	1.820997225	0.000621436	Arabidopsis thaliana chromosome 2, complete sequence
AT1G04600	1.606678104	0.000646309	Arabidopsis thaliana myosin XI A mRNA, complete cds

ATCG00065	2.973000318	0.000647977	Arabidopsis thaliana ecotype XJalt rpl20-rps12 intergenic spacer, complete sequence; ribosomal protein S12 (rps12) gene, partial cds; and rps12-clpP intergenic spacer, complete sequence; chloroplast
AT1G02530	2.973000318	0.000647977	Arabidopsis thaliana P-glycoprotein 12 mRNA, complete cds
AT5G66840	1.150173988	0.000669883	Arabidopsis thaliana SAP domain-containing protein mRNA, complete cds
AT5G66960	1.150173988	0.000669883	Arabidopsis thaliana Prolyl oligopeptidase family protein mRNA, complete cds
AT4G08109	4.525541342	0.000680644	Arabidopsis thaliana chromosome 4 sequence
AT1G26410	4.525541342	0.000680644	Arabidopsis thaliana chromosome 1 sequence
AT5G09950	1.494953022	0.000713006	Arabidopsis thaliana chromosome 5 sequence
AT5G46460	1.04265075	0.000750625	Arabidopsis thaliana chromosome 5 sequence
AT3G54870	1.277613828	0.000758286	Arabidopsis thaliana armadillo repeat-containing kinesin-like protein 1 mRNA, complete cds
AT5G54570	2.568610063	0.000766013	Arabidopsis thaliana beta glucosidase 41 mRNA, complete cds
ATCG00150	1.120557507	0.000772799	Arabidopsis thaliana chloroplast DNA, complete genome, ecotype: Columbia
AT5G40990	2.314037236	0.000810447	Arabidopsis thaliana GDSL lipase 1 mRNA, complete cds
AT1G51880	1.236034724	0.000904593	Arabidopsis thaliana protein ROOT HAIR SPECIFIC 6 mRNA, complete cds
AT3G09080	1.157257611	0.001013173	Arabidopsis thaliana transducin/WD40 domain-containing protein mRNA, complete cds
AT5G49110	1.018804008	0.001039096	Arabidopsis thaliana uncharacterized protein mRNA, complete cds
AT5G60880	1.018804008	0.001039096	Arabidopsis thaliana protein BREAKING OF ASYMMETRY IN THE STOMATAL LINEAGE mRNA, complete cds
AT1G08730	1.453132846	0.001090421	Arabidopsis thaliana myosin class XI mRNA, complete cds
AT4G21300	1.244446964	0.001108761	Arabidopsis thaliana chromosome 4

			sequence
AT3G44970	1.244446964	0.001108761	Arabidopsis thaliana cytochrome P450 family protein mRNA, complete cds
AT2G44630	1.300574977	0.001131548	Arabidopsis thaliana chromosome 2, complete sequence
AT5G13230	1.843717302	0.001138771	Arabidopsis thaliana chromosome 5 sequence
AT1G15040	1.843717302	0.001138771	Arabidopsis thaliana chromosome 1 sequence
AT1G64310	2.873464645	0.001194738	Arabidopsis thaliana chromosome 1 sequence
AT4G11393	2.873464645	0.001194738	Arabidopsis thaliana Defensin-like (DEFL) family protein mRNA, complete cds
AT2G46840	2.873464645	0.001194738	Arabidopsis thaliana uncharacterized protein mRNA, complete cds
AT1G52120	2.873464645	0.001194738	Arabidopsis thaliana jacalin-like lectin domain-containing protein mRNA, complete cds
AT5G10880	1.940578841	0.001262583	Arabidopsis thaliana tRNA synthetase/ligase-related protein mRNA, complete cds
AT5G18633	2.481147222	0.001367949	Arabidopsis thaliana chromosome 5 sequence
AT4G38780	1.314037236	0.001381268	Arabidopsis thaliana Pre-mRNA-processing-splicing factor mRNA, complete cds
AT3G27980	1.040114514	0.001403359	Arabidopsis thaliana plant invertase/pectin methylesterase inhibitor superfamily mRNA, complete cds
AT2G35420	1.127510268	0.001443097	Arabidopsis thaliana chromosome 2, complete sequence
AT3G06120	1.210499632	0.001611631	Arabidopsis thaliana transcription factor MUTE mRNA, complete cds
AT5G46915	1.709965913	0.001620499	Arabidopsis thaliana putative B3 domain-containing protein mRNA, complete cds
AT4G29550	3.525541342	0.001647721	Arabidopsis thaliana uncharacterized protein mRNA, complete cds
AT1G08860	3.525541342	0.001647721	Arabidopsis thaliana protein BONZAI 3 mRNA, complete cds
AT3G59580	1.095857066	0.001656636	Arabidopsis thaliana nodule inception protein-like protein 9 mRNA, complete cds
AT5G01730	1.410064124	0.001656749	Arabidopsis thaliana protein SCAR4

			mRNA, complete cds
AT1G08070	1.2640491	0.001665416	Arabidopsis thaliana chromosome 1 sequence
AT1G33615	1.329144129	0.001684952	Arabidopsis thaliana chromosome 1 sequence
ATCG00780	1.039142675	0.001730605	Arabidopsis thaliana chloroplast DNA, complete genome, ecotype: Columbia
AT2G31830	1.13024006	0.001778759	Arabidopsis thaliana Type II inositol-1,4,5-trisphosphate 5-phosphatase 14 mRNA, complete cds
AT5G65350	1.55153655	0.001863681	Arabidopsis thaliana chromosome 5 sequence
AT5G51920	1.218112816	0.001981034	Arabidopsis thaliana chromosome 5 sequence
AT1G02710	1.218112816	0.001981034	Arabidopsis thaliana chromosome 1 sequence
AT1G74350	1.218112816	0.001981034	Arabidopsis thaliana chromosome 1 sequence
AT4G22070	1.435343533	0.002001524	Arabidopsis thaliana WRKY DNA-binding protein 31 mRNA, complete cds
AT2G31751	1.435343533	0.002001524	Arabidopsis thaliana clone asmbl_6700 unknown mRNA sequence
AT1G15310	1.097136618	0.002043805	Arabidopsis thaliana signal recognition particle protein SRP54A mRNA, complete cds
AT5G06400	2.766549441	0.002196351	Arabidopsis thaliana chromosome 5 sequence
AT3G24929	1.59662444	0.002205415	Arabidopsis thaliana uncharacterized protein mRNA, complete cds
AT3G20395	1.992109141	0.002298503	Arabidopsis thaliana RING-finger domain-containing protein mRNA, complete cds
AT5G20420	1.066109723	0.002329751	Arabidopsis thaliana chromatin remodeling 42 mRNA, complete cds
AT5G65570	1.011661939	0.00240054	Arabidopsis thaliana chromosome 5 sequence
AT5G50260	1.464659099	0.002411313	Arabidopsis thaliana KDEL-tailed cysteine endopeptidase CEP1 mRNA, complete cds
AT5G61850	2.388037818	0.002430365	Arabidopsis thaliana protein LEAFY mRNA, complete cds
AT1G22260	2.388037818	0.002430365	Arabidopsis thaliana synaptonemal complex protein ZYP1a mRNA, complete cds

AT2G30670	2.388037818	0.002430365	Arabidopsis thaliana NAD(P)-binding Rossmann-fold superfamily protein mRNA, complete cds
AT4G35210	2.153572564	0.002433603	Arabidopsis thaliana chromosome 4 sequence
AT3G26614	4.236034724	0.00249179	Arabidopsis thaliana chromosome 3, complete sequence
AT1G31258	4.236034724	0.00249179	Arabidopsis thaliana uncharacterized protein mRNA, complete cds
AT3G28510	1.365670005	0.002500019	Arabidopsis thaliana chromosome 3, complete sequence
AT4G20730	1.365670005	0.002500019	Arabidopsis thaliana mRNA for putative protein, complete cds, clone: RAFL24-04-L03
AT3G47875	1.651072224	0.002589161	Arabidopsis thaliana chromosome 3, complete sequence
AT3G49950	1.136499051	0.002706813	Arabidopsis thaliana chromosome 3, complete sequence
AT1G11990	1.49906913	0.002894512	Arabidopsis thaliana O-fucosyltransferase family protein mRNA, complete cds
AT4G10940	1.49906913	0.002894512	Arabidopsis thaliana RING/U-box protein mRNA, complete cds
AT5G43530	1.718186419	0.003007173	Arabidopsis thaliana Helicase protein with RING/U-box domain mRNA, complete cds
AT2G24750	1.388037818	0.003038943	Arabidopsis thaliana chromosome 2, complete sequence
AT4G15165	1.388037818	0.003038943	Arabidopsis thaliana N-terminal nucleophile aminohydrolases (Ntn hydrolases) superfamily protein mRNA, complete cds
AT5G45470	1.30314892	0.003060728	Arabidopsis thaliana chromosome 5 sequence
AT5G03620	1.30314892	0.003060728	Arabidopsis thaliana Subtilisin-like serine endopeptidase family protein mRNA, complete cds
AT5G17430	1.100057055	0.003116529	Arabidopsis thaliana AP2-like ethylene-responsive transcription factor BBM mRNA, complete cds
AT1G29110	3.388037818	0.003143396	Arabidopsis thaliana cysteine proteinases superfamily protein mRNA, complete cds
AT2G18720	3.388037818	0.003143396	Arabidopsis thaliana translation elongation factor EF1A/initiation factor IF2gamma family protein mRNA,

			complete cds
AT2G30660	3.388037818	0.003143396	Arabidopsis thaliana probable 3-hydroxyisobutyryl-CoA hydrolase 3 mRNA, complete cds
AT5G67411	3.388037818	0.003143396	Arabidopsis thaliana clone asmbl_17543 unknown mRNA sequence
AT1G33430	3.388037818	0.003143396	Arabidopsis thaliana putative beta-1,3-galactosyltransferase 8 mRNA, complete cds
AT5G03250	1.035736074	0.003257316	Arabidopsis thaliana phototropic-responsive NPH3 family protein mRNA, complete cds
AT4G14170	1.140110304	0.003342018	Arabidopsis thaliana pentatricopeptide repeat-containing protein mRNA, complete cds
AT1G02190	1.140110304	0.003342018	Arabidopsis thaliana protein CER1-like 1 mRNA, complete cds
ATCG00360	1.803075317	0.003440767	Arabidopsis lyrata subsp. lyrata predicted protein, mRNA
ATCG00770	1.540040911	0.003458025	Arabidopsis thaliana chloroplast DNA, complete genome, ecotype: Columbia
AT3G16610	1.540040911	0.003458025	Arabidopsis thaliana chromosome 3, complete sequence
AT1G78340	1.188100247	0.003538182	Arabidopsis thaliana glutathione S-transferase TAU 22 mRNA, complete cds
AT3G01580	1.188100247	0.003538182	Arabidopsis thaliana pentatricopeptide repeat-containing protein mRNA, complete cds
AT5G52330	1.414033026	0.003686774	Arabidopsis thaliana meprin and TRAF homology domain-containing protein mRNA, complete cds
AT2G29500	1.101733633	0.00385241	Arabidopsis thaliana chromosome 2, complete sequence
AT4G25980	1.914106629	0.003852703	Arabidopsis thaliana peroxidase 43 mRNA, complete cds
ATMG00630	2.651072224	0.00402302	Arabidopsis thaliana ecotype Landsberg erecta mitochondrion, complete genome
AT5G38260	2.651072224	0.00402302	Arabidopsis thaliana protein kinase family protein mRNA, complete cds
AT2G07706	2.651072224	0.00402302	Arabidopsis thaliana uncharacterized protein mRNA, complete cds
AT1G35240	2.651072224	0.00402302	Arabidopsis thaliana auxin response factor 20 mRNA, complete cds

AT5G65550	2.066109723	0.004175193	Arabidopsis thaliana chromosome 5 sequence
AT5G48390	2.288502144	0.004292552	Arabidopsis thaliana ZIP4-like protein mRNA, complete cds
AT5G59330	2.288502144	0.004292552	Arabidopsis thaliana Bifunctional inhibitor/lipid-transfer protein/seed storage 2S albumin superfamily protein mRNA, complete cds
AT5G47330	1.19539274	0.004364661	Arabidopsis thaliana palmitoyl protein thioesterase family protein mRNA, complete cds
AT2G21800	1.066109723	0.004399768	Arabidopsis thaliana essential meiotic endonuclease 1A mRNA, complete cds
AT4G19080	1.444621346	0.004461017	Arabidopsis thaliana chromosome 4 sequence
AT2G34850	1.258754801	0.004528945	Arabidopsis thaliana putative UDP-arabinose 4-epimerase 2 mRNA, complete cds
AT4G16160	4.066109723	0.004820616	Arabidopsis thaliana outer envelope pore protein 16-2 mRNA, complete cds
AT5G28310	4.066109723	0.004820616	Arabidopsis thaliana NAD(P)-binding Rossmann-fold superfamily protein mRNA, complete cds
AT3G54490	4.066109723	0.004820616	Arabidopsis thaliana RNA polymerase II fifth largest subunit, E mRNA, complete cds
AT1G55010	4.066109723	0.004820616	Arabidopsis thaliana plant defensin 1.5 mRNA, complete cds
AT5G01570	1.651072224	0.004830228	Arabidopsis thaliana uncharacterized protein mRNA, complete cds
AT4G17760	1.032942859	0.004983033	Arabidopsis thaliana PCNA domain-containing protein mRNA, complete cds
AT2G12460	1.148571883	0.005104565	Arabidopsis thaliana chromosome 2, complete sequence
AT2G07711	1.148571883	0.005104565	Arabidopsis thaliana chromosome 2, complete sequence
AT4G00870	1.066109723	0.005446884	Arabidopsis thaliana transcription factor bHLH14 mRNA, complete cds
AT1G35513	1.2725606	0.005569213	Arabidopsis lyrata subsp. lyrata hypothetical protein, mRNA
AT2G45350	1.2725606	0.005569213	Arabidopsis thaliana chlororespiratory reduction 4 protein mRNA, complete cds
AT1G76530	1.361565606	0.005592948	Arabidopsis thaliana auxin efflux carrier

			family protein mRNA, complete cds
AT1G76065	1.361565606	0.005592948	Arabidopsis thaliana LYR family of Fe/S cluster biogenesis protein mRNA, complete cds
AT1G69120	1.001979385	0.005601013	Arabidopsis thaliana Floral homeotic protein APETALA 1 mRNA, complete cds
AT1G58889	1.729074736	0.005613557	Arabidopsis thaliana DNA, retrotransposon:AtRE1, complete sequence, ecotype: Niederzenz
AT3G04184	1.729074736	0.005613557	Arabidopsis thaliana chromosome 3, complete sequence
AT3G19663	3.236034724	0.006004734	Arabidopsis thaliana chromosome 3, complete sequence
AT5G35932	3.236034724	0.006004734	Arabidopsis thaliana chromosome 5 sequence
AT3G03660	3.236034724	0.006004734	Arabidopsis thaliana WUSCHEL related homeobox 11 mRNA, complete cds
AT5G34820	3.236034724	0.006004734	Arabidopsis thaliana chromosome 5 sequence
AT2G16005	3.236034724	0.006004734	Arabidopsis thaliana MD-2-related lipid recognition domain-containing protein mRNA, complete cds
AT1G32172	3.236034724	0.006004734	Arabidopsis thaliana chromosome 1 sequence
AT1G69920	3.236034724	0.006004734	Arabidopsis thaliana glutathione S-transferase TAU 12 mRNA, complete cds
AT1G53860	1.031344305	0.006170879	Arabidopsis thaliana remorin-like protein mRNA, complete cds
ATCG00740	1.153572564	0.006315363	Arabidopsis thaliana chloroplast DNA, complete genome, ecotype: Columbia
AT3G14640	1.831644469	0.006406111	Arabidopsis thaliana cytochrome P450, family 72, subfamily A, polypeptide 10 mRNA, complete cds
AT1G34460	1.525541342	0.006454221	Arabidopsis thaliana cyclin-B1-5 mRNA, complete cds
AT5G47600	1.066109723	0.006749262	Arabidopsis thaliana heat shock protein 14.7 mRNA, complete cds
AT3G09450	1.388037818	0.00681934	Arabidopsis thaliana uncharacterized protein mRNA, complete cds
AT5G61070	1.288502144	0.00684705	Arabidopsis thaliana histone deacetylase 18 mRNA, complete cds
AT2G28040	1.288502144	0.00684705	Arabidopsis thaliana chromosome 2, complete sequence

AT2G19190	1.288502144	0.00684705	Arabidopsis thaliana FLG22-induced receptor-like kinase 1 mRNA, complete cds
AT2G37370	1.973000318	0.007108109	Arabidopsis thaliana uncharacterized protein mRNA, complete cds
AT2G07776	1.973000318	0.007108109	Arabidopsis thaliana uncharacterized protein mRNA, complete cds
AT4G28700	2.525541342	0.007336074	Arabidopsis thaliana chromosome 4 sequence
AT3G60970	2.525541342	0.007336074	Arabidopsis thaliana putative ABC transporter C-15 mRNA, complete cds
AT2G10735	2.525541342	0.007336074	Arabidopsis thaliana chromosome 2, complete sequence
AT5G36740	2.525541342	0.007336074	Arabidopsis thaliana Acyl-CoA N-acyltransferase domain-containing protein mRNA, complete cds
AT3G32925	2.525541342	0.007336074	Arabidopsis thaliana chromosome 3, complete sequence
AT1G65810	2.525541342	0.007336074	Arabidopsis thaliana P-loop containing nucleoside triphosphate hydrolases superfamily protein mRNA, complete cds
AT5G03130	2.18158694	0.007530424	Arabidopsis thaliana uncharacterized protein mRNA, complete cds
AT4G27190	2.18158694	0.007530424	Arabidopsis thaliana chromosome 4 sequence
AT3G51060	1.029583847	0.007648942	Arabidopsis thaliana protein SHI RELATED SEQUENCE 1 mRNA, complete cds
AT4G18470	1.029583847	0.007648942	Arabidopsis thaliana negative regulator of systemic acquired resistance (SNI1) mRNA, complete cds
ATCG00640	1.580682896	0.007692207	Arabidopsis thaliana chloroplast DNA, complete genome, ecotype: Columbia
AT3G60670	1.159219127	0.007819468	Arabidopsis thaliana PLATZ transcription factor family protein mRNA, complete cds
AT1G79890	1.159219127	0.007819468	Arabidopsis thaliana RAD3-like DNA-binding helicase protein mRNA, complete cds
AT1G03540	1.307117822	0.008414732	Arabidopsis thaliana chromosome 1 sequence
AT5G46830	1.651072224	0.009078247	Arabidopsis thaliana chromosome 5 sequence
AT3G05790	1.651072224	0.009078247	Arabidopsis thaliana lon protease 4 mRNA, complete cds

AT3G25011	3.873464645	0.009402205	Arabidopsis thaliana rbpg1-1 (rbpg1-1) and rbpg1-2 (rbpg1-2) genes, complete cds
AT1G06750	3.873464645	0.009402205	Arabidopsis thaliana P-loop containing nucleoside triphosphate hydrolases superfamily protein mRNA, complete cds
AT5G20260	3.873464645	0.009402205	Arabidopsis thaliana Exostosin family protein mRNA, complete cds
AT3G10100	3.873464645	0.009402205	Arabidopsis thaliana At4g20730 mRNA, complete cds
AT2G33070	3.873464645	0.009402205	Arabidopsis thaliana Nitrile-specifier protein 2 mRNA, complete cds
AT3G46614	3.873464645	0.009402205	Arabidopsis thaliana chromosome 3, complete sequence
AT5G04630	3.873464645	0.009402205	Arabidopsis thaliana chromosome 5 sequence
AT3G47870	3.873464645	0.009402205	Arabidopsis thaliana LOB domain-containing protein 27 mRNA, complete cds
AT5G66700	1.458427146	0.010057994	Arabidopsis thaliana homeobox-leucine zipper protein ATHB-53 mRNA, complete cds
AT5G18350	1.458427146	0.010057994	Arabidopsis thaliana TIR-NBS-LRR class disease resistance protein mRNA, complete cds
AT5G61920	1.458427146	0.010057994	Arabidopsis thaliana protein FLX-like 4 mRNA, complete cds
AT5G19097	1.236034724	0.010152227	Arabidopsis thaliana chromosome 5 sequence
AT4G13992	1.329144129	0.01033424	Arabidopsis thaliana chromosome 4 sequence
AT2G01905	1.066109723	0.010394313	Arabidopsis thaliana cyclin J18 mRNA, complete cds
AT5G28340	1.066109723	0.010394313	Arabidopsis thaliana pentatricopeptide repeat-containing protein mRNA, complete cds
AT1G19394	1.744181628	0.010557732	Arabidopsis thaliana uncharacterized protein mRNA, complete cds
AT4G17085	1.744181628	0.010557732	Arabidopsis thaliana putative membrane lipoprotein mRNA, complete cds
AT4G02960	3.066109723	0.011480831	Arabidopsis thaliana copia-like retrotransposon AtRE2 gene for polyprotein, complete cds, ecotype: Isenburg, chromosome: 4

AT5G60470	3.066109723	0.011480831	Arabidopsis thaliana C2H2 and C2HC zinc finger-containing protein mRNA, complete cds
AT5G42120	3.066109723	0.011480831	Arabidopsis thaliana chromosome 5 sequence
AT3G12981	1.873464645	0.011996954	Arabidopsis lyrata subsp. lyrata hypothetical protein, mRNA
AT5G44630	1.873464645	0.011996954	Arabidopsis thaliana sesquiterpene synthase mRNA, complete cds
AT1G72720	1.873464645	0.011996954	Arabidopsis thaliana chromosome 1 sequence
AT3G25880	1.873464645	0.011996954	Arabidopsis thaliana NAD(P)-binding Rossmann-fold superfamily protein mRNA, complete cds
AT4G15350	1.173024927	0.012018553	Arabidopsis thaliana cytochrome P450, family 705, subfamily A, polypeptide 2 mRNA, complete cds
AT2G02061	1.506682314	0.012136768	Arabidopsis thaliana nucleotide-diphospho-sugar transferase domain-containing protein mRNA, complete cds
AT1G58460	1.506682314	0.012136768	Arabidopsis thaliana uncharacterized protein mRNA, complete cds
AT4G20420	1.250534294	0.012549702	Arabidopsis thaliana chromosome 4 sequence
AT5G33382	1.35561634	0.012677439	Arabidopsis thaliana chromosome 5 sequence
AT3G20730	1.35561634	0.012677439	Arabidopsis thaliana pentatricopeptide repeat-containing protein mRNA, complete cds
AT4G35190	1.066109723	0.012921694	Arabidopsis thaliana cytokinin riboside 5'-monophosphate phosphoribohydrolase LOG5 mRNA, complete cds
AT1G51670	1.066109723	0.012921694	Arabidopsis thaliana uncharacterized protein mRNA, complete cds
AT1G03800	2.066109723	0.013107783	Arabidopsis thaliana chromosome 1 sequence
AT1G52430	2.066109723	0.013107783	Arabidopsis thaliana ubiquitin carboxyl-terminal hydrolase-related protein mRNA, complete cds
AT3G04050	2.066109723	0.013107783	Arabidopsis thaliana pyruvate kinase mRNA, complete cds
AT3G05900	2.066109723	0.013107783	Arabidopsis thaliana neurofilament

			protein-related protein mRNA, complete cds
AT2G46455	2.388037818	0.013304278	Arabidopsis thaliana OxaA/YidC-like membrane insertion protein mRNA, complete cds
AT2G39240	1.023041001	0.014661756	Arabidopsis thaliana RNA polymerase I specific transcription initiation factor RRN3 protein mRNA, complete cds
AT5G59305	1.18158694	0.014921097	Arabidopsis thaliana uncharacterized protein mRNA, complete cds
AT5G46140	1.18158694	0.014921097	Arabidopsis thaliana uncharacterized protein mRNA, complete cds
ATMG00660	1.267743584	0.015516775	Arabidopsis thaliana ecotype Landsberg erecta mitochondrion, complete genome
AT4G12740	1.066109723	0.016084973	Arabidopsis thaliana HhH-GPD base excision DNA repair family protein mRNA, complete cds
AT3G56550	1.120557507	0.017428339	Arabidopsis thaliana pentatricopeptide repeat-containing protein mRNA, complete cds
AT5G06650	3.651072224	0.018501818	Arabidopsis thaliana chromosome 5 sequence
AT4G25200	3.651072224	0.018501818	Arabidopsis thaliana small heat shock protein 23.6 mRNA, complete cds
AT5G46665	3.651072224	0.018501818	Arabidopsis thaliana chromosome 5 sequence
AT1G13290	3.651072224	0.018501818	Arabidopsis thaliana protein DOT5 mRNA, complete cds
AT1G65630	3.651072224	0.018501818	Arabidopsis thaliana putativeDegP protease 3 mRNA, complete cds
AT5G28715	3.651072224	0.018501818	Arabidopsis thaliana chromosome 5 sequence
AT1G16705	3.651072224	0.018501818	Arabidopsis thaliana p300/CBP acetyltransferase-related protein mRNA, complete cds
AT2G04515	3.651072224	0.018501818	Arabidopsis thaliana chromosome 2, complete sequence
AT2G15070	3.651072224	0.018501818	Arabidopsis thaliana chromosome 2, complete sequence
AT3G24900	3.651072224	0.018501818	Arabidopsis thaliana chromosome 3, complete sequence
AT3G24340	1.428679802	0.018957645	Arabidopsis thaliana chromatin remodeling 40 mRNA, complete cds
AT5G64685	1.428679802	0.018957645	Arabidopsis thaliana chromosome 5

			sequence
AT3G11680	1.288502144	0.01918588	Arabidopsis thaliana Aluminum activated malate transporter family protein mRNA, complete cds
AT5G56970	1.288502144	0.01918588	Arabidopsis thaliana cytokinin dehydrogenase 3 mRNA, complete cds
AT5G58280	1.288502144	0.01918588	Arabidopsis thaliana AP2/B3-like transcriptional factor family protein mRNA, complete cds
AT4G26260	1.766549441	0.020051575	Arabidopsis thaliana myo-inositol oxygenase 4 mRNA, complete cds
AT5G25230	1.766549441	0.020051575	Arabidopsis thaliana ribosomal protein S5/Elongation factor G/III/V family protein mRNA, complete cds
AT1G69880	1.766549441	0.020051575	Arabidopsis thaliana thioredoxin H8 mRNA, complete cds
AT2G47190	1.766549441	0.020051575	Arabidopsis thaliana mRNA for MYB transcription factor, complete cds, clone: RAFL16-42-L19
AT1G67792	1.066109723	0.020052441	Arabidopsis thaliana mRNA for hypothetical protein, complete cds, clone: RAFL07-30-D10
AT2G39510	1.066109723	0.020052441	Arabidopsis thaliana nodulin MtN21-like transporter family protein mRNA, complete cds
AT4G16015	1.125003412	0.021727318	Arabidopsis thaliana chromosome 4 sequence
AT2G20480	1.125003412	0.021727318	Arabidopsis thaliana uncharacterized protein mRNA, complete cds
AT2G15060	2.873464645	0.021956467	Arabidopsis thaliana chromosome 2, complete sequence
AT3G48523	2.873464645	0.021956467	Arabidopsis thaliana chromosome 3, complete sequence
AT1G32510	2.873464645	0.021956467	Arabidopsis thaliana NAC domain containing protein 11 mRNA, complete cds
AT4G15320	2.873464645	0.021956467	Arabidopsis thaliana cellulose synthase-like protein B6 mRNA, complete cds
AT3G62610	2.873464645	0.021956467	Arabidopsis thaliana myb domain protein 11 mRNA, complete cds
AT5G33395	2.873464645	0.021956467	Arabidopsis thaliana chromosome 5 sequence
AT5G28510	2.873464645	0.021956467	Arabidopsis thaliana beta glucosidase 24

			mRNA, complete cds
AT2G03020	1.940578841	0.022610034	Arabidopsis thaliana Heat shock protein HSP20/alpha crystallin family protein mRNA, complete cds
AT5G55020	1.940578841	0.022610034	Arabidopsis thaliana myb domain protein 120 mRNA, complete cds
AT1G04880	1.940578841	0.022610034	Arabidopsis thaliana high mobility group B protein 15 mRNA, complete cds
AT5G64490	1.940578841	0.022610034	Arabidopsis thaliana chromosome 5 sequence
AT5G07640	1.940578841	0.022610034	Arabidopsis thaliana RING/U-box superfamily protein mRNA, complete cds
AT3G15930	1.017200122	0.022775755	Arabidopsis thaliana pentatricopeptide repeat-containing protein mRNA, complete cds
AT1G44120	1.481147222	0.02304694	Arabidopsis thaliana armadillo/beta-catenin-like repeat and C2 calcium/lipid-binding domain-containing protein mRNA, complete cds
AT5G12280	1.481147222	0.02304694	Arabidopsis thaliana SWAP (Suppressor-of-White-APricot)/surp RNA-binding domain-containing protein mRNA, complete cds
AT4G26200	1.203613247	0.023070092	Arabidopsis thaliana 1-aminocyclopropane-1-carboxylate synthase 7 mRNA, complete cds
AT2G27610	1.203613247	0.023070092	Arabidopsis thaliana chromosome 2, complete sequence
AT5G40320	1.314037236	0.023715556	Arabidopsis thaliana chromosome 5 sequence
AT2G29370	2.236034724	0.023965033	Arabidopsis thaliana tropinone reductase-like protein mRNA, complete cds
AT1G62240	2.236034724	0.023965033	Arabidopsis thaliana chromosome 1 sequence
AT4G16820	2.236034724	0.023965033	Arabidopsis thaliana chromosome 4 sequence
AT4G19760	2.236034724	0.023965033	Arabidopsis thaliana Glycosyl hydrolase family protein with chitinase insertion domain mRNA, complete cds
AT1G08080	2.236034724	0.023965033	Arabidopsis thaliana alpha carbonic anhydrase 7 mRNA, complete cds
AT2G27880	2.236034724	0.023965033	Arabidopsis thaliana argonaute 5 mRNA,

			complete cds
AT4G12890	2.236034724	0.023965033	Arabidopsis thaliana Gamma interferon responsive lysosomal thiol (GILT) reductase family protein mRNA, complete cds
AT4G14140	1.55153655	0.027813574	Arabidopsis thaliana DNA methyltransferase 2 mRNA, complete cds
AT1G58320	1.55153655	0.027813574	Arabidopsis thaliana PLAC8 family protein mRNA, complete cds
AT5G09930	1.55153655	0.027813574	Arabidopsis thaliana ABC transporter F family member 2 mRNA, complete cds
AT3G27120	1.013642303	0.028457984	Arabidopsis thaliana P-loop containing nucleoside triphosphate hydrolases superfamily protein mRNA, complete cds
AT1G13620	1.218112816	0.028733515	Arabidopsis thaliana root meristem growth factor 2 mRNA, complete cds
AT1G64260	1.218112816	0.028733515	Arabidopsis thaliana chromosome 1 sequence
AT5G24205	1.218112816	0.028733515	Arabidopsis thaliana chromosome 5 sequence
AT5G24900	1.218112816	0.028733515	Arabidopsis thaliana mRNA for cytochrome P450 like protein, complete cds, clone: RAFL09-95-E07
AT4G24170	1.346217642	0.029289793	Arabidopsis thaliana ATP binding microtubule motor family protein mRNA, complete cds
AT1G47760	1.346217642	0.029289793	Arabidopsis thaliana protein agamous-like 102 mRNA, complete cds
AT1G53540	1.651072224	0.033145571	Arabidopsis thaliana chromosome 1 sequence
AT3G05152	1.651072224	0.033145571	Arabidopsis thaliana Full-length cDNA Complete sequence from clone GSLTSIL76ZB06 of Silique of strain col-0 of Arabidopsis thaliana (thale cress)
AT5G64395	1.651072224	0.033145571	Arabidopsis thaliana chromosome 5 sequence
AT1G67270	1.651072224	0.033145571	Arabidopsis thaliana zinc-finger domain of monoamine-oxidase A repressor R1 protein mRNA, complete cds
AT4G16590	1.651072224	0.033145571	Arabidopsis thaliana cellulose synthase-like A01 mRNA, complete cds
AT4G37780	1.136499051	0.033941986	Arabidopsis thaliana myb domain protein 87 mRNA, complete cds
AT5G16850	1.236034724	0.035826919	Arabidopsis thaliana telomerase reverse

			transcriptase mRNA, complete cds
AT1G61440	1.236034724	0.035826919	Arabidopsis thaliana G-type lectin S-receptor-like serine/threonine-protein kinase mRNA, complete cds
AT4G39366	1.236034724	0.035826919	Arabidopsis thaliana chromosome 4 sequence
AT1G27820	3.388037818	0.036767383	Arabidopsis thaliana chromosome 1 sequence
AT2G22821	3.388037818	0.036767383	Arabidopsis thaliana chromosome 2, complete sequence
AT4G25510	3.388037818	0.036767383	Arabidopsis thaliana uncharacterized protein mRNA, complete cds
AT3G58420	3.388037818	0.036767383	Arabidopsis thaliana TRAF-like family protein mRNA, complete cds
AT5G66340	3.388037818	0.036767383	Arabidopsis thaliana uncharacterized protein mRNA, complete cds
AT1G33830	3.388037818	0.036767383	Arabidopsis thaliana P-loop containing nucleoside triphosphate hydrolases superfamily protein mRNA, complete cds
AT3G03702	3.388037818	0.036767383	Arabidopsis thaliana mRNA for unknown protein, complete cds, clone: RAFL19-12-M17
AT3G60170	3.388037818	0.036767383	Arabidopsis thaliana chromosome 3, complete sequence
AT1G50830	3.388037818	0.036767383	Arabidopsis thaliana chromosome 1 sequence
AT4G16560	3.388037818	0.036767383	Arabidopsis thaliana HSP20-like chaperone mRNA, complete cds
AT1G28220	3.388037818	0.036767383	Arabidopsis thaliana purine permease 3 mRNA, complete cds
AT4G16550	3.388037818	0.036767383	Arabidopsis thaliana HSP20-like chaperone mRNA, complete cds
AT5G52740	3.388037818	0.036767383	Arabidopsis thaliana copper transport family protein mRNA, complete cds
AT4G01380	3.388037818	0.036767383	Arabidopsis thaliana plastocyanin-like domain-containing protein mRNA, complete cds
AT1G64240	3.388037818	0.036767383	Arabidopsis thaliana chromosome 1 sequence
AT5G24355	3.388037818	0.036767383	Arabidopsis thaliana chromosome 5 sequence
AT3G23960	3.388037818	0.036767383	Arabidopsis thaliana chromosome 3, complete sequence
AT4G14368	1.803075317	0.03859026	Arabidopsis thaliana regulator of

			chromosome condensation repeat-containing protein mRNA, complete cds
AT1G58390	1.803075317	0.03859026	Arabidopsis thaliana CC-NBS-LRR class disease resistance protein mRNA, complete cds
AT2G45410	1.803075317	0.03859026	Arabidopsis thaliana LOB domain-containing protein 19 mRNA, complete cds
AT4G36740	1.803075317	0.03859026	Arabidopsis thaliana homeobox protein 40 mRNA, complete cds
AT5G51330	1.803075317	0.03859026	Arabidopsis thaliana protein DYAD mRNA, complete cds
AT5G55270	1.803075317	0.03859026	Arabidopsis thaliana chromosome 5 sequence
AT3G04420	1.066109723	0.039282472	Arabidopsis thaliana NAC domain containing protein 48 mRNA, complete cds
AT1G79360	1.066109723	0.039282472	Arabidopsis thaliana chromosome 1 sequence
AT2G45940	2.651072224	0.04196297	Arabidopsis thaliana uncharacterized protein mRNA, complete cds
AT2G21187	2.651072224	0.04196297	Arabidopsis thaliana chromosome 2, complete sequence
AT2G04852	2.651072224	0.04196297	Arabidopsis thaliana chromosome 2, complete sequence
AT2G22905	2.651072224	0.04196297	Arabidopsis thaliana chromosome 2, complete sequence
AT5G13940	2.651072224	0.04196297	Arabidopsis thaliana aminopeptidase mRNA, complete cds
AT4G04510	2.651072224	0.04196297	Arabidopsis thaliana cysteine-rich receptor-like protein kinase 38 mRNA, complete cds
AT4G10265	2.651072224	0.04196297	Arabidopsis thaliana chromosome 4 sequence
AT3G49580	2.651072224	0.04196297	Arabidopsis thaliana protein RESPONSE TO LOW SULFUR 1 mRNA, complete cds
AT5G38750	2.651072224	0.04196297	Arabidopsis thaliana asparaginyl-tRNA synthetase-like protein mRNA, complete cds
ATMG01030	2.651072224	0.04196297	Arabidopsis thaliana ecotype Landsberg erecta mitochondrion, complete genome
AT1G57780	2.651072224	0.04196297	Arabidopsis thaliana heavy-metal-associated

			domain-containing protein mRNA, complete cds
AT5G47170	2.651072224	0.04196297	Arabidopsis thaliana chromosome 5 sequence
AT4G03930	2.651072224	0.04196297	Arabidopsis thaliana putative pectinesterase/pectinesterase inhibitor 42 mRNA, complete cds
AT4G32205	2.651072224	0.04196297	Arabidopsis thaliana clone asmbl_13272 unknown mRNA sequence
AT1G53260	2.651072224	0.04196297	Arabidopsis thaliana uncharacterized protein mRNA, complete cds
AT2G04038	2.651072224	0.04196297	Arabidopsis thaliana chromosome 2, complete sequence
AT5G57690	2.651072224	0.04196297	Arabidopsis thaliana diacylglycerol kinase 4 mRNA, complete cds
AT5G28360	1.144112235	0.042551978	Arabidopsis thaliana 1-aminocyclopropane-1-carboxylate synthase mRNA, complete cds
AT5G50940	1.144112235	0.042551978	Arabidopsis thaliana RNA-binding KH domain-containing protein mRNA, complete cds
AT1G49220	1.144112235	0.042551978	Arabidopsis thaliana chromosome 1 sequence
ATCG00200	1.144112235	0.042551978	Aster spathulifolius chloroplast, complete genome
AT2G13810	2.066109723	0.04280713	Arabidopsis thaliana AGD2-like defense response protein 1 mRNA, complete cds
AT2G35200	2.066109723	0.04280713	Arabidopsis thaliana chromosome 2, complete sequence
AT3G48010	2.066109723	0.04280713	Arabidopsis thaliana cyclic nucleotide-gated channel 16 mRNA, complete cds
AT2G33000	2.066109723	0.04280713	Arabidopsis thaliana ubiquitin-associated (UBA)/TS-N domain-containing protein-like protein mRNA, complete cds
AT2G44590	2.066109723	0.04280713	Arabidopsis thaliana dynamin-related protein 1d mRNA, complete cds
AT1G59265	2.066109723	0.04280713	Arabidopsis thaliana chromosome 1 sequence
AT5G44990	2.066109723	0.04280713	Arabidopsis thaliana Glutathione S-transferase family protein mRNA, complete cds
AT5G22570	2.066109723	0.04280713	Arabidopsis thaliana putative WRKY transcription factor 38 mRNA, complete

			cds
AT4G04540	2.066109723	0.04280713	Arabidopsis thaliana putative cysteine-rich receptor-like protein kinase 39 mRNA, complete cds
AT3G44705	1.444621346	0.044355851	Arabidopsis thaliana chromosome 3, complete sequence
AT2G23348	1.444621346	0.044355851	Arabidopsis thaliana uncharacterized protein mRNA, complete cds
AT2G46567	1.444621346	0.044355851	Arabidopsis thaliana chromosome 2, complete sequence
AT4G03816	1.444621346	0.044355851	Arabidopsis thaliana chromosome 4 sequence
AT1G18100	1.444621346	0.044355851	Arabidopsis thaliana protein MOTHER of FT and TF 1 mRNA, complete cds
AT5G49370	1.004709178	0.044706391	Arabidopsis thaliana Pleckstrin homology (PH) domain-containing protein-like protein mRNA, complete cds
AT5G49380	1.258754801	0.044717251	Arabidopsis thaliana uncharacterized protein mRNA, complete cds
AT1G28680	1.258754801	0.044717251	Arabidopsis thaliana HXXXD-type acyl-transferase family protein mRNA, complete cds
AT1G14490	1.066109723	0.049380462	Arabidopsis thaliana chromosome 1 sequence
AT3G04440	1.066109723	0.049380462	Arabidopsis thaliana plasma-membrane choline transporter family protein mRNA, complete cds
AT4G36770	1.066109723	0.049380462	Arabidopsis thaliana chromosome 4 sequence
AT2G37880	1.153572564	0.053474806	Arabidopsis thaliana chromosome 2, complete sequence
AT3G43110	1.153572564	0.053474806	Arabidopsis thaliana chromosome 3, complete sequence
AT4G33800	1.153572564	0.053474806	Arabidopsis thaliana uncharacterized protein mRNA, complete cds
AT1G74400	1.153572564	0.053474806	Arabidopsis thaliana chromosome 1 sequence
AT3G51570	1.153572564	0.053474806	Arabidopsis thaliana TIR-NBS-LRR class disease resistance protein mRNA, complete cds
AT1G05894	1.525541342	0.054105249	Arabidopsis thaliana chromosome 1 sequence
AT2G12450	1.525541342	0.054105249	Arabidopsis thaliana chromosome 2, complete sequence

AT5G53100	1.525541342	0.054105249	Arabidopsis thaliana Rossmann-fold NAD(P)-binding domain-containing protein mRNA, complete cds
AT4G35180	1.525541342	0.054105249	Arabidopsis thaliana LYS/HIS transporter 7 mRNA, complete cds
AT1G23465	1.288502144	0.055856619	Arabidopsis thaliana peptidase-S24/S26 domain-containing protein mRNA, complete cds
AT1G52920	1.066109723	0.062260007	Arabidopsis thaliana G-protein coupled receptor 2 mRNA, complete cds
AT3G28750	1.066109723	0.062260007	Arabidopsis thaliana chromosome 3, complete sequence
AT1G04150	1.066109723	0.062260007	Arabidopsis thaliana chromosome 1 sequence
AT1G64320	1.066109723	0.062260007	Arabidopsis thaliana myosin heavy chain-related protein mRNA, complete cds

Supplementary Data S8 Down regulated genes in *fc1*/Col-o without Cd treatment.

Gene	Log2FoldChange	pval	NT:Description
AT4G12550	-1.641314025	0	Arabidopsis thaliana chromosome 4 sequence
AT3G16240	-1.039583315	0	Arabidopsis thaliana aquaporin TIP2-1 mRNA, complete cds
AT2G37170	-1.164119236	0	Arabidopsis thaliana aquaporin PIP2-2 mRNA, complete cds
AT1G73330	-1.187880266	0	Arabidopsis thaliana chromosome 1 sequence
AT4G02380	-1.3124019	3.34E-250	Arabidopsis thaliana senescence-associated protein SAG21 mRNA, complete cds
AT3G01190	-1.155732262	1.42E-241	Arabidopsis thaliana peroxidase 27 mRNA, complete cds
AT1G78850	-1.013200923	2.52E-237	Arabidopsis thaliana chromosome 1 sequence
AT4G19690	-2.395313743	1.69E-233	Arabidopsis thaliana Fe(2+) transport protein 1 mRNA, complete cds
AT4G23400	-1.014697285	4.57E-220	Arabidopsis thaliana putative aquaporin PIP1-5 mRNA, complete cds
AT5G63160	-1.294242531	4.36E-215	Arabidopsis thaliana BTB and TAZ domain protein 1 mRNA, complete cds
AT5G43350	-1.278448485	2.18E-179	Arabidopsis thaliana inorganic phosphate transporter 1-1 mRNA, complete cds
AT2G33830	-1.397369828	5.41E-172	Arabidopsis thaliana dormancy/auxin associated protein mRNA, complete cds
AT2G40000	-1.538877889	6.78E-172	Arabidopsis thaliana chromosome 2, complete sequence
AT4G12520	-1.589878074	7.31E-171	Arabidopsis thaliana chromosome 4 sequence
AT3G48360	-1.002162298	7.36E-170	Arabidopsis thaliana TAC1-mediated telomerase activation pathway protein BT2 mRNA, complete cds
AT1G70850	-1.43563941	7.01E-166	Arabidopsis thaliana MLP-like protein 34 mRNA, complete cds
AT1G32640	-1.658422049	3.22E-151	Arabidopsis thaliana chromosome 1 sequence
AT5G53450	-1.542839458	6.86E-149	Arabidopsis thaliana OBP3-responsive protein 1 mRNA, complete cds
AT5G19240	-1.112723151	5.33E-147	Arabidopsis thaliana GPI-anchored glycoprotein membrane precursor mRNA, complete cds
AT2G28630	-1.310945368	1.81E-145	Arabidopsis thaliana chromosome 2, complete sequence
AT5G06320	-1.124356057	2.85E-134	Arabidopsis thaliana chromosome 5 sequence
AT1G55450	-1.166105341	1.43E-129	Arabidopsis thaliana S-adenosyl-L-methionine-dependent methyltransferases superfamily protein mRNA, complete cds
AT5G47450	-1.118224958	1.63E-128	Arabidopsis thaliana aquaporin TIP2-3 mRNA,

			complete cds
AT3G05950	-1.44376759	1.79E-126	Arabidopsis thaliana mRNA for germin-like protein, complete cds, clone: RAFL16-44-D15
AT1G01580	-1.809066252	1.51E-123	Arabidopsis thaliana ferric reduction oxidase 2 mRNA, complete cds
AT4G12545	-1.192665717	2.64E-122	Arabidopsis thaliana chromosome 4 sequence
AT1G19770	-1.153849349	4.81E-119	Arabidopsis thaliana purine permease 14 mRNA, complete cds
AT5G60660	-1.376567457	7.44E-114	Arabidopsis thaliana putative aquaporin PIP2-4 mRNA, complete cds
AT2G22500	-1.585386529	3.05E-107	Arabidopsis thaliana chromosome 2, complete sequence
AT1G47600	-2.864627615	3.25E-106	Arabidopsis thaliana myrosinase 4 mRNA, complete cds
AT5G17820	-1.122551841	2.00E-103	Arabidopsis thaliana peroxidase 57 mRNA, complete cds
AT5G26030	-2.36149645	2.12E-103	Arabidopsis thaliana ferrochelatase 1 mRNA, complete cds
AT1G51470	-2.912930658	4.03E-99	Arabidopsis thaliana myrosinase 5 mRNA, complete cds
AT3G44300	-2.279665114	1.60E-97	Arabidopsis thaliana nitrilase 2 mRNA, complete cds
AT1G66100	-1.407507802	3.06E-97	Arabidopsis thaliana probable thionin-2.4 mRNA, complete cds
AT5G46890	-1.456074361	4.44E-96	Arabidopsis thaliana chromosome 5 sequence
AT2G23810	-1.191990279	1.09E-91	Arabidopsis thaliana tetraspanin8 mRNA, complete cds
AT4G12720	-1.073293334	2.60E-88	Arabidopsis thaliana nudix hydrolase 7 mRNA, complete cds
AT3G44260	-2.544305957	4.22E-88	Arabidopsis thaliana chromosome 3, complete sequence
AT4G24570	-2.448149786	4.37E-88	Arabidopsis thaliana chromosome 4 sequence
AT5G48430	-1.06182119	5.19E-87	Arabidopsis thaliana chromosome 5 sequence
AT3G55980	-1.242304586	2.11E-86	Arabidopsis thaliana salt-inducible zinc finger 1 mRNA, complete cds
AT2G01530	-1.424525395	2.94E-86	Arabidopsis thaliana MLP-like protein 329 mRNA, complete cds
AT5G63600	-1.041009122	4.08E-86	Arabidopsis thaliana flavonol synthase 5 mRNA, complete cds
AT5G46900	-1.53776124	1.20E-83	Arabidopsis thaliana chromosome 5 sequence
AT4G00970	-1.285565715	1.84E-81	Arabidopsis thaliana cysteine-rich receptor-like protein kinase 41 mRNA, complete cds
AT3G56880	-1.10361712	3.20E-79	Arabidopsis thaliana chromosome 3, complete sequence
AT2G30930	-1.224651412	7.49E-79	Arabidopsis thaliana uncharacterized protein mRNA,

			complete cds
AT5G54370	-2.031187478	1.08E-78	Arabidopsis thaliana late embryogenesis abundant protein-like protein mRNA, complete cds
AT1G27730	-2.604967936	3.71E-77	Arabidopsis thaliana chromosome 1 sequence
AT4G18205	-1.147943398	8.95E-75	Arabidopsis thaliana nucleotide-sugar transporter family protein mRNA, complete cds
AT1G52070	-2.320725635	1.04E-74	Arabidopsis thaliana jacalin-like lectin domain-containing protein mRNA, complete cds
AT2G37130	-1.127178353	2.32E-72	Arabidopsis thaliana peroxidase mRNA, complete cds
AT5G61600	-1.679706789	3.52E-72	Arabidopsis thaliana chromosome 5 sequence
AT4G29780	-2.302697064	1.29E-70	Arabidopsis thaliana chromosome 4 sequence
AT1G52060	-2.07046333	3.33E-68	Arabidopsis thaliana jacalin-like lectin domain-containing protein mRNA, complete cds
AT1G74670	-1.032574552	1.82E-66	Arabidopsis thaliana gibberellin-regulated protein 6 mRNA, complete cds
AT3G19030	-1.357281055	1.88E-66	Arabidopsis thaliana chromosome 3, complete sequence
AT4G27280	-1.819985822	1.58E-65	Arabidopsis thaliana chromosome 4 sequence
AT4G33120	-1.782684473	2.57E-65	Arabidopsis thaliana S-adenosyl-L-methionine-dependent methyltransferases superfamily protein mRNA, complete cds
AT1G52050	-2.91163747	6.93E-63	Arabidopsis thaliana jacalin-like lectin domain-containing protein mRNA, complete cds
AT5G05250	-1.838030262	9.97E-62	Arabidopsis thaliana chromosome 5 sequence
AT2G24600	-1.207614961	2.24E-60	Arabidopsis thaliana Ankyrin repeat family protein mRNA, complete cds
AT2G32150	-1.283648215	7.93E-60	Arabidopsis thaliana haloacid dehalogenase-like hydrolase domain-containing protein mRNA, complete cds
AT3G22060	-1.401833396	1.10E-59	Arabidopsis thaliana Receptor-like protein kinase-related family protein mRNA, complete cds
AT5G45340	-1.214720266	1.92E-58	Arabidopsis thaliana abscisic acid 8'-hydroxylase 3 mRNA, complete cds
AT3G29410	-1.100980349	1.14E-55	Arabidopsis thaliana terpenoid synthase 25 mRNA, complete cds
AT2G45220	-1.327554126	3.08E-55	Arabidopsis thaliana putative pectinesterase/pectinesterase inhibitor 17 mRNA, complete cds
AT5G26130	-1.840160225	4.82E-55	Arabidopsis thaliana chromosome 5 sequence
AT4G37610	-1.014614214	2.78E-54	Arabidopsis thaliana BTB and TAZ domain protein 5 mRNA, complete cds
AT3G16440	-2.466924986	1.98E-52	Arabidopsis thaliana myrosinase-binding protein-like protein-300B mRNA, complete cds

AT3G51330	-1.030330037	2.16E-52	Arabidopsis thaliana aspartyl protease family protein mRNA, complete cds
AT4G11290	-1.057150986	3.46E-52	Arabidopsis thaliana peroxidase 39 mRNA, complete cds
AT1G06090	-2.563749316	1.71E-51	Arabidopsis thaliana delta-9 desaturase-like 1 protein mRNA, complete cds
AT5G13740	-1.026483076	4.62E-51	Arabidopsis thaliana zinc induced facilitator 1 proetin mRNA, complete cds
AT1G56430	-1.087115476	7.74E-51	Arabidopsis thaliana chromosome 1 sequence
AT3G12900	-2.015278604	7.88E-51	Arabidopsis thaliana oxidoreductase, 2OG-Fe(II) oxygenase family protein mRNA, complete cds
AT3G52400	-1.122029276	1.08E-49	Arabidopsis thaliana syntaxin-122 mRNA, complete cds
AT4G36500	-1.004619103	2.54E-48	Arabidopsis thaliana chromosome 4 sequence
AT5G57560	-1.248606885	2.62E-48	Arabidopsis thaliana xyloglucan endotransglucosylase/hydrolase protein 22 mRNA, complete cds
AT3G25930	-1.158234925	3.86E-47	Arabidopsis thaliana Adenine nucleotide alpha hydrolases-like superfamily protein mRNA, complete cds
AT5G60530	-1.618919733	2.28E-46	Arabidopsis thaliana late embryogenesis abundant protein-like protein mRNA, complete cds
AT5G44610	-1.010163019	3.94E-46	Arabidopsis thaliana microtubule-associated protein 18 mRNA, complete cds
AT5G37770	-1.164886764	5.04E-46	Arabidopsis thaliana chromosome 5 sequence
AT3G24300	-1.37056369	5.26E-46	Arabidopsis thaliana chromosome 3, complete sequence
AT1G23020	-1.177733863	1.58E-45	Arabidopsis thaliana ferric reduction oxidase 3 mRNA, complete cds
AT4G11280	-1.459959089	1.93E-44	Arabidopsis thaliana 1-aminocyclopropane-1-carboxylate synthase 6 mRNA, complete cds
AT3G22600	-1.023461018	1.14E-43	Arabidopsis thaliana protease inhibitor/seed storage/lipid transfer protein (LTP) family protein mRNA, complete cds
AT2G14878	-1.184952041	5.37E-43	Arabidopsis thaliana chromosome 2, complete sequence
AT3G07720	-1.126980563	1.51E-42	Arabidopsis thaliana galactose oxidase/kelch repeat-containing protein mRNA, complete cds
AT3G50740	-1.219627734	4.48E-42	Arabidopsis thaliana chromosome 3, complete sequence
AT2G33790	-1.135362047	1.97E-41	Arabidopsis thaliana arabinogalactan protein 30 mRNA, complete cds
AT1G73260	-2.024210884	3.20E-41	Arabidopsis thaliana chromosome 1 sequence

AT1G13609	-3.692335599	3.29E-41	Arabidopsis thaliana defensin-like protein 287 mRNA, complete cds
AT1G57990	-1.007251728	5.75E-41	Arabidopsis thaliana chromosome 1 sequence
AT5G12940	-1.407306125	1.59E-40	Arabidopsis thaliana chromosome 5 sequence
AT1G21100	-1.026421563	1.62E-39	Arabidopsis thaliana indole glucosinolate o-methyltransferase 1 mRNA, complete cds
AT2G28160	-1.404655924	1.75E-39	Arabidopsis thaliana FER-LIKE IRON DEFICIENCY-INDUCED transcription factor mRNA, complete cds
AT1G65845	-1.280646509	4.18E-38	Arabidopsis thaliana uncharacterized protein mRNA, complete cds
AT1G80840	-2.401016288	5.19E-38	Arabidopsis thaliana putative WRKY transcription factor 40 mRNA, complete cds
AT3G29250	-1.321815931	1.38E-36	Arabidopsis thaliana short-chain dehydrogenase reductase 4 mRNA, complete cds
AT1G73540	-1.347309498	2.12E-36	Arabidopsis thaliana nudix hydrolase 21 mRNA, complete cds
AT1G64980	-1.043173927	2.26E-36	Arabidopsis thaliana putative nucleotide-diphospho-sugar transferase mRNA, complete cds
AT1G07750	-1.295474572	3.53E-36	Arabidopsis thaliana cupin domain-containing protein mRNA, complete cds
AT2G14247	-4.859889696	6.73E-36	Arabidopsis thaliana chromosome 2, complete sequence
AT1G29025	-1.024345228	7.01E-36	Arabidopsis thaliana Calcium-binding EF-hand family protein mRNA, complete cds
AT5G51190	-2.184265111	1.37E-35	Arabidopsis thaliana chromosome 5 sequence
AT2G29350	-2.524705504	2.16E-35	Arabidopsis thaliana senescence-associated protein 13 mRNA, complete cds
AT1G53830	-1.551642713	8.02E-34	Arabidopsis thaliana pectin methylesterase 2 mRNA, complete cds
AT2G22122	-1.089081648	3.22E-33	Arabidopsis thaliana chromosome 2, complete sequence
AT1G33610	-1.224441569	1.30E-32	Arabidopsis thaliana leucine-rich repeat (LRR) family protein mRNA, complete cds
AT5G10580	-1.025812767	2.30E-32	Arabidopsis thaliana uncharacterized protein mRNA, complete cds
AT2G39200	-1.077254452	4.77E-32	Arabidopsis thaliana protein MILDEW RESISTANCE LOCUS O 12 mRNA, complete cds
AT2G36100	-1.120230778	1.25E-31	Arabidopsis thaliana casparian strip membrane protein 1 mRNA, complete cds
AT1G19180	-1.331156916	5.29E-31	Arabidopsis thaliana chromosome 1 sequence
AT1G22500	-1.323342366	2.77E-30	Arabidopsis thaliana chromosome 1 sequence
AT3G56980	-3.548600121	1.78E-29	Arabidopsis thaliana transcription factor ORG3

			mRNA, complete cds
AT1G49500	-1.136927766	2.64E-29	Arabidopsis thaliana uncharacterized protein mRNA, complete cds
AT4G13620	-1.554476688	2.10E-28	Arabidopsis thaliana chromosome 4 sequence
AT3G18560	-1.200098931	2.50E-28	Arabidopsis thaliana chromosome 3, complete sequence
AT1G72910	-1.162159265	2.68E-28	Arabidopsis thaliana Toll-Interleukin-Resistance domain-containing protein mRNA, complete cds
AT5G04200	-1.507625522	2.88E-28	Arabidopsis thaliana chromosome 5 sequence
AT1G61890	-1.065239699	4.51E-28	Arabidopsis thaliana MATE efflux family protein mRNA, complete cds
AT1G29280	-1.010061958	1.58E-27	Arabidopsis thaliana putative WRKY transcription factor 65 mRNA, complete cds
AT3G46620	-1.252900889	1.76E-27	Arabidopsis thaliana chromosome 3, complete sequence
AT1G26820	-1.741245199	2.57E-27	Arabidopsis thaliana ribonuclease 3 mRNA, complete cds
AT3G32030	-1.233008489	3.01E-27	Arabidopsis thaliana terpenoid synthase 30 mRNA, complete cds
AT2G26530	-1.331545698	6.15E-27	Arabidopsis thaliana chromosome 2, complete sequence
AT4G21830	-1.029347436	1.14E-26	Arabidopsis thaliana peptide methionine sulfoxide reductase B7 mRNA, complete cds
AT3G61060	-1.232069769	3.37E-26	Arabidopsis thaliana phloem protein 2-A13 mRNA, complete cds
AT2G27830	-1.30450031	5.55E-26	Arabidopsis thaliana chromosome 2, complete sequence
AT2G41240	-4.009178404	5.91E-26	Arabidopsis thaliana transcription factor bHLH100 mRNA, complete cds
AT5G57480	-1.33525284	6.84E-26	Arabidopsis thaliana chromosome 5 sequence
AT3G45710	-1.191188313	6.87E-26	Arabidopsis thaliana putative nitrate excretion transporter 6 mRNA, complete cds
AT1G18300	-1.630821209	1.38E-25	Arabidopsis thaliana nudix hydrolase 4 mRNA, complete cds
AT5G05600	-1.790526102	2.10E-25	Arabidopsis thaliana oxidoreductase, 2OG-Fe(II) oxygenase family protein mRNA, complete cds
AT1G74450	-1.313104113	9.10E-25	Arabidopsis thaliana chromosome 1 sequence
AT4G15760	-1.149525332	2.53E-24	Arabidopsis thaliana monooxygenase 1 mRNA, complete cds
AT2G42060	-1.456925029	3.53E-24	Arabidopsis thaliana chromosome 2, complete sequence
AT1G56010	-1.021051078	4.08E-24	Arabidopsis thaliana transcription factor NAC1 mRNA, complete cds
AT3G25780	-1.038101272	7.61E-24	Arabidopsis thaliana allene oxide cyclase 3 mRNA,

			complete cds
AT5G66390	-1.055880801	1.03E-23	Arabidopsis thaliana peroxidase 72 mRNA, complete cds
AT1G78460	-1.159310391	1.05E-23	Arabidopsis thaliana SOUL heme-binding protein mRNA, complete cds
AT5G24410	-1.183783583	1.36E-23	Arabidopsis thaliana 6-phosphogluconolactonase 4 mRNA, complete cds
AT4G23220	-1.162421836	1.60E-23	Arabidopsis thaliana cysteine-rich receptor-like protein kinase 14 mRNA, complete cds
AT4G17490	-1.518852778	1.64E-23	Arabidopsis thaliana chromosome 4 sequence
AT3G51350	-1.080731665	2.07E-23	Arabidopsis thaliana aspartyl protease family protein mRNA, complete cds
AT3G15210	-1.188631476	2.38E-23	Arabidopsis thaliana chromosome 3, complete sequence
AT2G41660	-1.289155556	3.47E-23	Arabidopsis thaliana chromosome 2, complete sequence
AT3G52450	-1.354222076	4.08E-23	Arabidopsis thaliana chromosome 3, complete sequence
AT3G62020	-1.220771425	4.42E-23	Arabidopsis thaliana chromosome 3, complete sequence
AT1G44350	-2.126535355	5.17E-23	Arabidopsis thaliana IAA-amino acid hydrolase ILR1-like 6 mRNA, complete cds
AT5G17350	-2.119001682	8.33E-23	Arabidopsis thaliana chromosome 5 sequence
AT5G64120	-1.528836866	3.65E-22	Arabidopsis thaliana peroxidase 71 mRNA, complete cds
AT4G15093	-1.039326552	5.50E-22	Arabidopsis thaliana extradiol ring-cleavage dioxygenase mRNA, complete cds
AT4G10310	-1.029542516	7.29E-22	Arabidopsis thaliana sodium transporter HKT1 mRNA, complete cds
AT5G02090	-2.198593503	1.03E-21	Arabidopsis thaliana chromosome 5 sequence
AT3G25760	-1.873629752	1.53E-21	Arabidopsis thaliana allene oxide cyclase 1 mRNA, complete cds
AT1G05675	-1.44846345	3.10E-21	Arabidopsis thaliana UDP-Glycosyltransferase superfamily protein mRNA, complete cds
AT2G35710	-1.204192683	3.40E-21	Arabidopsis thaliana putative glucuronosyltransferase PGSIP8 mRNA, complete cds
AT3G56970	-3.606315619	3.44E-21	Arabidopsis thaliana transcription factor ORG2 mRNA, complete cds
AT1G11540	-1.064850955	3.73E-21	Arabidopsis thaliana Sulfite exporter TauE/SafE family protein mRNA, complete cds
AT5G04340	-1.738070951	5.99E-21	Arabidopsis thaliana chromosome 5 sequence
AT1G14200	-1.052363747	1.06E-20	Arabidopsis thaliana chromosome 1 sequence
AT1G62280	-1.933890277	1.57E-20	Arabidopsis thaliana S-type anion channel SLAH1 mRNA, complete cds

AT5G52750	-1.249836214	1.91E-20	Arabidopsis thaliana heavy metal transport/detoxification domain-containing protein mRNA, complete cds
AT4G11210	-1.652708525	2.05E-20	Arabidopsis thaliana chromosome 4 sequence
AT5G42180	-1.188788468	2.30E-20	Arabidopsis thaliana peroxidase mRNA, complete cds
AT1G65310	-1.253799204	2.50E-20	Arabidopsis thaliana probable xyloglucan endotransglucosylase/hydrolase protein 17 mRNA, complete cds
AT3G19580	-1.266160316	3.03E-20	Arabidopsis thaliana chromosome 3, complete sequence
AT2G39430	-1.345867977	5.52E-20	Arabidopsis thaliana chromosome 2, complete sequence
AT4G29740	-1.031258114	8.27E-20	Arabidopsis thaliana cytokinin dehydrogenase 4 mRNA, complete cds
AT3G05200	-1.097928435	8.99E-20	Arabidopsis thaliana chromosome 3, complete sequence
AT5G15180	-1.131620644	1.06E-19	Arabidopsis thaliana peroxidase 56 mRNA, complete cds
AT4G33790	-1.895822236	1.49E-19	Arabidopsis thaliana fatty acyl-CoA reductase CER4 mRNA, complete cds
AT1G72940	-1.06672596	4.21E-19	Arabidopsis thaliana Toll-Interleukin-Resistance domain-containing protein mRNA, complete cds
AT3G21550	-1.16926834	4.48E-19	Arabidopsis thaliana chromosome 3, complete sequence
AT3G16720	-1.020961985	5.55E-19	Arabidopsis thaliana chromosome 3, complete sequence
AT5G27420	-1.174898377	7.87E-19	Arabidopsis thaliana chromosome 5 sequence
AT4G21250	-1.011196003	1.14E-18	Arabidopsis thaliana Sulfite exporter TauE/SafE family protein mRNA, complete cds
AT3G49190	-3.118314848	1.33E-18	Arabidopsis thaliana O-acyltransferase (WSD1-like) family protein mRNA, complete cds
AT4G23170	-1.046000643	2.16E-18	Arabidopsis thaliana chromosome 4 sequence
AT5G47220	-2.143343643	2.88E-18	Arabidopsis thaliana chromosome 5 sequence
AT3G06460	-2.410328321	3.23E-18	Arabidopsis thaliana chromosome 3, complete sequence
AT5G47950	-1.900723413	3.62E-18	Arabidopsis thaliana chromosome 5 sequence
AT4G22666	-1.098277095	3.65E-18	Arabidopsis thaliana Bifunctional inhibitor/lipid-transfer protein/seed storage 2S albumin superfamily protein mRNA, complete cds
AT1G66090	-1.150919514	5.38E-18	Arabidopsis thaliana TIR-NBS class of disease resistance protein mRNA, complete cds
AT1G30870	-1.023441862	5.96E-18	Arabidopsis thaliana peroxidase 7 mRNA, complete cds
AT2G20670	-1.023441862	5.96E-18	Arabidopsis thaliana uncharacterized protein mRNA,

			complete cds
AT5G19970	-1.154917156	8.25E-18	Arabidopsis thaliana chromosome 5 sequence
AT5G65080	-3.166551034	8.34E-18	Arabidopsis thaliana protein MADS AFFECTING FLOWERING 5 mRNA, complete cds
AT1G23710	-1.163228764	9.17E-18	Arabidopsis thaliana chromosome 1 sequence
AT1G72520	-1.425030755	9.41E-18	Arabidopsis thaliana lipoxygenase 4 mRNA, complete cds
AT1G53940	-1.233098296	1.08E-17	Arabidopsis thaliana GDSL-motif lipase 2 mRNA, complete cds
AT3G14680	-1.044637343	1.72E-17	Arabidopsis thaliana cytochrome P450, family 72, subfamily A, polypeptide 14 mRNA, complete cds
AT1G72920	-1.605009422	2.38E-17	Arabidopsis thaliana Toll-Interleukin-Resistance domain-containing protein mRNA, complete cds
AT5G14650	-1.334852591	2.55E-17	Arabidopsis thaliana pectin lyase-like superfamily protein mRNA, complete cds
AT1G05650	-1.44846345	2.66E-17	Arabidopsis thaliana pectin lyase-like protein mRNA, complete cds
AT2G24710	-1.082920054	3.01E-17	Arabidopsis thaliana glutamate receptor 2.3 mRNA, complete cds
AT5G06200	-1.658577542	3.76E-17	Arabidopsis thaliana uncharacterized protein mRNA, complete cds
AT4G23870	-1.206143213	4.31E-17	Arabidopsis thaliana chromosome 4 sequence
AT5G03570	-1.481378072	5.04E-17	Arabidopsis thaliana nickel transport protein FPN2 mRNA, complete cds
AT3G58810	-1.229780511	5.81E-17	Arabidopsis thaliana chromosome 3, complete sequence
AT5G59820	-1.745280712	6.14E-17	Arabidopsis thaliana chromosome 5 sequence
AT1G47400	-2.506141726	6.14E-17	Arabidopsis thaliana chromosome 1 sequence
AT4G25250	-1.295711099	8.89E-17	Arabidopsis thaliana chromosome 4 sequence
AT3G28340	-1.65333811	9.20E-17	Arabidopsis thaliana chromosome 3, complete sequence
AT3G29035	-1.847475525	1.30E-16	Arabidopsis thaliana NAC domain-containing protein 3 mRNA, complete cds
AT1G73500	-1.243434648	1.31E-16	Arabidopsis thaliana chromosome 1 sequence
AT4G12510	-1.127770011	2.12E-16	Arabidopsis thaliana chromosome 4 sequence
AT4G37370	-1.40714239	2.23E-16	Arabidopsis thaliana cytochrome P450, family 81, subfamily D, polypeptide 8 mRNA, complete cds
AT3G04010	-1.630498134	3.15E-16	Arabidopsis thaliana O-glycosyl hydrolases family 17 protein mRNA, complete cds
AT2G27370	-1.60442851	4.04E-16	Arabidopsis thaliana uncharacterized protein mRNA, complete cds
AT1G73300	-1.44846345	5.48E-16	Arabidopsis thaliana serine carboxypeptidase-like 2 mRNA, complete cds

AT5G42590	-1.44846345	5.48E-16	Arabidopsis thaliana cytochrome P450 71A16 mRNA, complete cds
AT1G08320	-1.077192755	5.89E-16	Arabidopsis thaliana bZIP transcription factor family protein mRNA, complete cds
AT4G26050	-1.065715489	5.97E-16	Arabidopsis thaliana plant intracellular ras group-related LRR 8 mRNA, complete cds
AT4G36850	-1.634329995	8.31E-16	Arabidopsis thaliana PQ-loop repeat family protein / transmembrane family protein mRNA, complete cds
AT2G36220	-1.156282698	8.38E-16	Arabidopsis thaliana chromosome 2, complete sequence
AT1G70690	-1.035674015	9.32E-16	Arabidopsis thaliana plasmodesmata-located protein 5 mRNA, complete cds
AT5G39020	-1.269757563	1.69E-15	Arabidopsis thaliana chromosome 5 sequence
AT5G23840	-1.060562081	1.96E-15	Arabidopsis thaliana MD-2-related lipid recognition domain-containing protein mRNA, complete cds
AT3G44860	-3.538752335	2.09E-15	Arabidopsis thaliana farnesoic acid carboxyl-O-methyltransferase mRNA, complete cds
AT1G63295	-1.355132926	2.26E-15	Arabidopsis thaliana Remorin family protein mRNA, complete cds
AT3G16690	-1.323217657	2.47E-15	Arabidopsis thaliana bidirectional sugar transporter SWEET16 mRNA, complete cds
AT1G69890	-1.034584277	2.63E-15	Arabidopsis thaliana uncharacterized protein mRNA, complete cds
AT1G50560	-1.100448427	2.88E-15	Arabidopsis thaliana cytochrome P450, family 705, subfamily A, polypeptide 25 mRNA, complete cds
AT2G27080	-1.057122043	3.07E-15	Arabidopsis thaliana chromosome 2, complete sequence
AT1G66800	-1.504206002	3.73E-15	Arabidopsis thaliana alcohol dehydrogenase-like protein mRNA, complete cds
AT5G40730	-1.351047828	5.93E-15	Arabidopsis thaliana chromosome 5 sequence
AT1G08500	-1.550561638	6.64E-15	Arabidopsis thaliana early nodulin-like protein 18 mRNA, complete cds
AT4G15290	-1.407061952	8.38E-15	Arabidopsis thaliana cellulose synthase-like protein B5 mRNA, complete cds
AT3G48340	-1.119635796	1.21E-14	Arabidopsis thaliana KDEL-tailed cysteine endopeptidase CEP2 mRNA, complete cds
AT1G73280	-1.543100324	1.61E-14	Arabidopsis thaliana serine carboxypeptidase-like 3 mRNA, complete cds
AT4G17500	-1.317495269	1.92E-14	Arabidopsis thaliana chromosome 4 sequence
AT5G06839	-1.355591507	1.99E-14	Arabidopsis thaliana bZIP transcription factor TGA10 mRNA, complete cds
AT5G37450	-1.087460666	2.04E-14	Arabidopsis thaliana probable LRR receptor-like serine/threonine-protein kinase mRNA, complete cds
AT3G26500	-1.165215823	3.40E-14	Arabidopsis thaliana plant intracellular ras

			group-related LRR 2 mRNA, complete cds
AT1G49000	-1.487991814	3.49E-14	Arabidopsis thaliana chromosome 1 sequence
AT1G17190	-1.02964926	4.40E-14	Arabidopsis thaliana glutathione S-transferase tau 26 mRNA, complete cds
AT5G59260	-1.788039411	4.43E-14	Arabidopsis thaliana chromosome 5 sequence
AT3G55970	-3.864627615	7.03E-14	Arabidopsis thaliana jasmonate-regulated protein JRG21 mRNA, complete cds
AT5G45080	-1.112764235	7.39E-14	Arabidopsis thaliana protein PHLOEM PROTEIN 2-LIKE A6 mRNA, complete cds
AT4G35770	-1.830054466	7.65E-14	Arabidopsis thaliana senescence-associated protein DIN1 mRNA, complete cds
AT5G15150	-1.13028749	8.16E-14	Arabidopsis thaliana mRNA for homeobox protein, complete cds, clone: RAFL16-61-C06
AT5G22250	-1.707297867	8.50E-14	Arabidopsis thaliana chromosome 5 sequence
AT5G08240	-1.158105434	8.60E-14	Arabidopsis thaliana uncharacterized protein mRNA, complete cds
AT1G65390	-1.281813581	9.68E-14	Arabidopsis thaliana protein PHLOEM protein 2-LIKE A5 mRNA, complete cds
AT3G26610	-1.150848812	1.03E-13	Arabidopsis thaliana putative polygalacturonase / pectinase mRNA, complete cds
AT2G25810	-1.071393801	1.07E-13	Arabidopsis thaliana aquaporin TIP4-1 mRNA, complete cds
AT1G53680	-1.143682662	1.22E-13	Arabidopsis thaliana glutathione S-transferase TAU 28 mRNA, complete cds
AT4G15330	-1.017413633	1.42E-13	Arabidopsis thaliana cytochrome P450, family 705, subfamily A, polypeptide 1 mRNA, complete cds
AT3G05920	-1.64873306	1.60E-13	Arabidopsis thaliana heavy-metal-associated domain-containing protein mRNA, complete cds
AT1G30750	-1.237282421	1.70E-13	Arabidopsis thaliana chromosome 1 sequence
AT2G39040	-1.339529078	1.97E-13	Arabidopsis thaliana peroxidase 24 mRNA, complete cds
AT2G23030	-1.493761797	2.73E-13	Arabidopsis thaliana serine/threonine-protein kinase SNRK2.9 mRNA, complete cds
AT4G27400	-2.354222076	4.57E-13	Arabidopsis thaliana late embryogenesis abundant protein-like protein mRNA, complete cds
AT5G04150	-4.606315619	7.19E-13	Arabidopsis thaliana transcription factor bHLH101 mRNA, complete cds
AT5G43370	-1.832010663	7.61E-13	Arabidopsis thaliana phosphate transporter Pht1;2 mRNA, complete cds
AT1G73270	-1.416041972	1.06E-12	Arabidopsis thaliana serine carboxypeptidase-like 6 mRNA, complete cds
AT5G38100	-1.884980677	1.28E-12	Arabidopsis thaliana putative S-adenosylmethionine-dependent methyltransferase mRNA, complete cds

AT3G09020	-1.273520374	1.37E-12	Arabidopsis thaliana chromosome 3, complete sequence
AT5G38820	-2.999979468	1.49E-12	Arabidopsis thaliana putative amino acid transporter mRNA, complete cds
AT1G66160	-1.528251476	1.76E-12	Arabidopsis thaliana chromosome 1 sequence
AT4G34410	-5.488479129	1.86E-12	Arabidopsis thaliana chromosome 4 sequence
AT5G06570	-1.028354961	2.10E-12	Arabidopsis thaliana probable carboxylesterase 15 mRNA, complete cds
AT2G32300	-1.070309475	2.10E-12	Arabidopsis thaliana uclacyanin 1 mRNA, complete cds
AT4G15400	-1.164670484	2.65E-12	Arabidopsis thaliana chromosome 4 sequence
AT5G24070	-2.089168503	2.99E-12	Arabidopsis thaliana probable peroxidase 61 mRNA, complete cds
AT2G21020	-1.160525922	4.21E-12	Arabidopsis thaliana chromosome 2, complete sequence
AT1G28370	-2.741245199	4.79E-12	Arabidopsis thaliana chromosome 1 sequence
AT3G52520	-1.362733576	5.25E-12	Arabidopsis thaliana chromosome 3, complete sequence
AT1G74930	-2.272692191	5.63E-12	Arabidopsis thaliana chromosome 1 sequence
AT3G11550	-1.002227407	7.72E-12	Arabidopsis thaliana uncharacterized protein mRNA, complete cds
AT2G25735	-1.109967506	1.02E-11	Arabidopsis thaliana chromosome 2, complete sequence
AT1G22550	-1.113213977	1.37E-11	Arabidopsis thaliana putative peptide/nitrate transporter mRNA, complete cds
AT2G40095	-1.103815279	1.40E-11	Arabidopsis thaliana alpha/beta hydrolase related protein mRNA, complete cds
AT5G47240	-1.550561638	1.51E-11	Arabidopsis thaliana nudix hydrolase 8 mRNA, complete cds
AT4G31380	-1.550561638	1.51E-11	Arabidopsis thaliana chromosome 4 sequence
AT1G29395	-1.585966974	1.97E-11	Arabidopsis thaliana cold regulated 314 inner membrane 1 mRNA, complete cds
AT5G47230	-1.189835258	2.31E-11	Arabidopsis thaliana chromosome 5 sequence
AT4G32280	-1.0654199	2.66E-11	Arabidopsis thaliana auxin-responsive protein IAA29 mRNA, complete cds
AT3G49845	-1.222566566	2.75E-11	Arabidopsis thaliana uncharacterized protein mRNA, complete cds
AT1G27140	-2.374462869	2.98E-11	Arabidopsis thaliana glutathione S-transferase tau 14 mRNA, complete cds
AT1G55152	-1.07506634	3.07E-11	Arabidopsis thaliana chromosome 1 sequence
AT1G47603	-1.68725031	3.35E-11	Arabidopsis thaliana purine permease 19 mRNA, complete cds
AT3G06390	-1.158596564	3.48E-11	Arabidopsis thaliana uncharacterized protein mRNA, complete cds

AT1G23830	-1.451289494	3.88E-11	Arabidopsis thaliana chromosome 1 sequence
AT5G47990	-1.563940667	4.40E-11	Arabidopsis thaliana cytochrome P450 705A5 mRNA, complete cds
AT1G14250	-1.20441922	4.66E-11	Arabidopsis thaliana probable apyrase 5 mRNA, complete cds
AT5G26340	-1.210730482	6.17E-11	Arabidopsis thaliana sugar transport protein 13 mRNA, complete cds
AT1G59590	-1.228633543	6.68E-11	Arabidopsis thaliana chromosome 1 sequence
AT3G19430	-1.646608325	7.20E-11	Arabidopsis thaliana late embryogenesis abundant protein-like protein mRNA, complete cds
AT3G61190	-2.017306285	7.93E-11	Arabidopsis thaliana BON association protein 1 mRNA, complete cds
AT4G19370	-1.367786804	8.59E-11	Arabidopsis thaliana uncharacterized protein mRNA, complete cds
AT2G45550	-1.374462869	9.70E-11	Arabidopsis thaliana cytochrome P450 76C4 mRNA, complete cds
AT2G39330	-1.013889599	1.57E-10	Arabidopsis thaliana jacalin-related lectin 23 mRNA, complete cds
AT3G16390	-1.14655116	1.62E-10	Arabidopsis thaliana Nitrile-specifier protein 3 mRNA, complete cds
AT2G39650	-1.355354046	2.01E-10	Arabidopsis thaliana uncharacterized protein mRNA, complete cds
AT3G05155	-1.208252689	2.10E-10	Arabidopsis thaliana mRNA for hypothetical protein, complete cds, clone: RAFL14-21-B09
AT2G44220	-1.707614421	2.33E-10	Arabidopsis thaliana uncharacterized protein mRNA, complete cds
AT5G42250	-1.023686947	2.48E-10	Arabidopsis thaliana alcohol dehydrogenase-like 7 mRNA, complete cds
AT1G07135	-1.770391545	2.74E-10	Arabidopsis thaliana chromosome 1 sequence
AT1G23850	-1.001867071	2.80E-10	Arabidopsis thaliana chromosome 1 sequence
AT4G22460	-1.844622939	3.06E-10	Arabidopsis thaliana chromosome 4 sequence
AT3G15300	-1.131829655	3.07E-10	Arabidopsis thaliana chromosome 3, complete sequence
AT1G14185	-1.568605813	3.13E-10	Arabidopsis thaliana glucose-methanol-choline oxidoreductase-like protein mRNA, complete cds
AT3G03500	-1.390747952	3.69E-10	Arabidopsis thaliana TatD related DNase mRNA, complete cds
AT1G47395	-1.778915617	4.43E-10	Arabidopsis thaliana chromosome 1 sequence
AT1G21910	-1.473409807	6.24E-10	Arabidopsis thaliana chromosome 1 sequence
AT5G62420	-3.196924683	6.91E-10	Arabidopsis thaliana aldo/keto reductase family protein mRNA, complete cds
AT4G15380	-1.005551262	6.92E-10	Arabidopsis thaliana cytochrome P450, family 705, subfamily A, polypeptide 4 mRNA, complete cds
AT2G44600	-1.021353118	7.07E-10	Arabidopsis thaliana chromosome 2, complete

			sequence
AT5G24100	-1.075664238	7.09E-10	Arabidopsis thaliana Leucine-rich repeat protein kinase family protein mRNA, complete cds
AT1G66440	-2.143343643	7.13E-10	Arabidopsis thaliana chromosome 1 sequence
AT1G76590	-1.19023003	7.52E-10	Arabidopsis thaliana PLATZ transcription factor family protein mRNA, complete cds
AT2G32620	-2.371295589	8.10E-10	Arabidopsis thaliana cellulose synthase-like protein B2 mRNA, complete cds
AT3G45730	-1.10008256	9.31E-10	Arabidopsis thaliana chromosome 3, complete sequence
AT4G31320	-1.058781525	9.66E-10	Arabidopsis thaliana chromosome 4 sequence
AT1G65970	-1.328749894	1.09E-09	Arabidopsis thaliana thioredoxin-dependent peroxidase 2 mRNA, complete cds
AT1G74660	-1.255818372	1.09E-09	Arabidopsis thaliana chromosome 1 sequence
AT1G58420	-2.123714836	1.16E-09	Arabidopsis thaliana chromosome 1 sequence
AT5G10280	-1.127662021	1.20E-09	Arabidopsis thaliana myb domain protein 92 mRNA, complete cds
AT3G32040	-1.670855871	1.35E-09	Arabidopsis thaliana geranylgeranyl pyrophosphate synthase 12 mRNA, complete cds
AT4G29050	-1.318554127	1.56E-09	Arabidopsis thaliana chromosome 4 sequence
AT5G10625	-1.348927776	1.60E-09	Arabidopsis thaliana chromosome 5 sequence
AT1G54790	-1.191688035	1.60E-09	Arabidopsis thaliana GDSL esterase/lipase mRNA, complete cds
AT4G15740	-1.399013008	2.03E-09	Arabidopsis thaliana chromosome 4 sequence
AT4G37850	-2.571320198	2.34E-09	Arabidopsis thaliana transcription factor bHLH25 mRNA, complete cds
AT1G22250	-1.066915696	2.37E-09	Arabidopsis thaliana uncharacterized protein mRNA, complete cds
AT3G52480	-1.153456654	2.40E-09	Arabidopsis thaliana chromosome 3, complete sequence
AT1G31885	-1.246168202	2.85E-09	Arabidopsis thaliana aquaporin NIP3-1 mRNA, complete cds
AT1G50050	-1.189729182	2.95E-09	Arabidopsis thaliana putative pathogenesis-related protein mRNA, complete cds
AT3G23630	-1.992783966	3.20E-09	Arabidopsis thaliana chromosome 3, complete sequence
AT1G65570	-3.103815279	3.80E-09	Arabidopsis thaliana polygalacturonase family protein mRNA, complete cds
AT1G30370	-1.775192531	4.48E-09	Arabidopsis thaliana chromosome 1 sequence
AT2G40113	-1.424215904	4.69E-09	Arabidopsis thaliana pollen Ole e 1 allergen and extensin family protein mRNA, complete cds
AT2G45360	-1.184116907	4.73E-09	Arabidopsis thaliana uncharacterized protein mRNA, complete cds

AT5G35490	-1.48037863	4.97E-09	Arabidopsis thaliana chromosome 5 sequence
AT1G60190	-1.492621236	5.52E-09	Arabidopsis thaliana chromosome 1 sequence
AT4G31875	-1.007139259	5.68E-09	Arabidopsis thaliana chromosome 4 sequence
AT1G71050	-1.054519982	5.84E-09	Arabidopsis thaliana heavy-metal-associated domain-containing protein mRNA, complete cds
AT1G61750	-1.402443286	6.08E-09	Arabidopsis thaliana Receptor-like protein kinase-related protein mRNA, complete cds
AT5G16170	-1.127770011	6.29E-09	Arabidopsis thaliana core-2/I-branching beta-1,6-N-acetylglucosaminyltransferase family protein mRNA, complete cds
AT4G13580	-1.231156318	6.47E-09	Arabidopsis thaliana chromosome 4 sequence
AT1G19900	-1.077856052	6.72E-09	Arabidopsis thaliana chromosome 1 sequence
AT4G37990	-1.28278542	7.33E-09	Arabidopsis thaliana cinnamyl alcohol dehydrogenase 8 mRNA, complete cds
AT4G00955	-1.103815279	7.60E-09	Arabidopsis thaliana chromosome 4 sequence
AT5G43540	-1.630498134	7.68E-09	Arabidopsis thaliana chromosome 5 sequence
AT5G35870	-4.933890277	8.80E-09	Arabidopsis thaliana chromosome 5 sequence
AT1G08890	-1.00944131	8.95E-09	Arabidopsis thaliana sugar transporter ERD6-like 1 mRNA, complete cds
AT1G14540	-1.069773705	9.12E-09	Arabidopsis thaliana peroxidase 4 mRNA, complete cds
AT2G30770	-1.294887937	9.54E-09	Arabidopsis thaliana cytochrome P450, family 71, subfamily A, polypeptide 13 mRNA, complete cds
AT2G23630	-1.442903925	9.62E-09	Arabidopsis thaliana protein SKU5 similar 16 mRNA, complete cds
AT4G04745	-1.716298842	1.01E-08	Arabidopsis thaliana chromosome 4 sequence
AT4G18360	-1.0953537	1.04E-08	Arabidopsis thaliana glycolate oxidase mRNA, complete cds
AT1G33817	-1.826975073	1.22E-08	Arabidopsis thaliana chromosome 1 sequence
AT3G29034	-1.02039927	1.22E-08	Arabidopsis thaliana chromosome 3, complete sequence
AT4G15270	-2.021353118	1.27E-08	Arabidopsis thaliana glucosyltransferase-related protein mRNA, complete cds
AT1G05310	-1.031187478	1.43E-08	Arabidopsis thaliana putative pectinesterase 8 mRNA, complete cds
AT3G23180	-1.179366312	1.62E-08	Arabidopsis thaliana HR-like lesion-inducing protein-like protein mRNA, complete cds
AT1G11190	-1.179366312	1.62E-08	Arabidopsis thaliana endonuclease 1 mRNA, complete cds
AT2G34600	-5.840780873	1.75E-08	Arabidopsis thaliana jasmonate-zim-domain protein 7 mRNA, complete cds
AT5G48290	-1.245091965	1.81E-08	Arabidopsis thaliana heavy metal transport/detoxification domain-containing protein mRNA, complete cds

AT1G34040	-1.054905678	1.94E-08	Arabidopsis thaliana Pyridoxal phosphate-dependent transferases superfamily protein mRNA, complete cds
AT2G05380	-1.549549575	2.20E-08	Arabidopsis thaliana glycine-rich protein 3 short isoform mRNA, complete cds
AT5G35480	-1.659715314	2.21E-08	Arabidopsis thaliana chromosome 5 sequence
AT1G02400	-1.014508737	2.22E-08	Arabidopsis thaliana gibberellin 2-oxidase 6 mRNA, complete cds
AT3G14260	-1.025388632	2.61E-08	Arabidopsis thaliana LURP-one-related 11 protein mRNA, complete cds
AT2G22570	-1.069667056	2.61E-08	Arabidopsis thaliana nicotinamidase 1 mRNA, complete cds
AT3G60490	-1.213998196	2.71E-08	Arabidopsis thaliana chromosome 3, complete sequence
AT3G20160	-3.978284396	2.72E-08	Arabidopsis thaliana chromosome 3, complete sequence
AT1G45015	-1.239392824	2.93E-08	Arabidopsis thaliana MD-2-related lipid recognition domain-containing protein mRNA, complete cds
AT3G59880	-1.00747141	3.46E-08	Arabidopsis thaliana chromosome 3, complete sequence
AT1G24147	-1.073165066	3.53E-08	Arabidopsis thaliana uncharacterized protein mRNA, complete cds
AT2G20880	-1.550561638	3.61E-08	Arabidopsis thaliana chromosome 2, complete sequence
AT2G47540	-1.380146507	3.85E-08	Arabidopsis thaliana pollen Ole e 1 allergen and extensin family protein mRNA, complete cds
AT2G01880	-1.149618968	4.30E-08	Arabidopsis thaliana purple acid phosphatase 7 mRNA, complete cds
AT1G27670	-2.181817791	4.51E-08	Arabidopsis thaliana chromosome 1 sequence
AT4G34790	-1.801786741	4.77E-08	Arabidopsis thaliana chromosome 4 sequence
AT4G20820	-1.353429169	8.04E-08	Arabidopsis thaliana chromosome 4 sequence
AT1G32350	-3.888086588	8.89E-08	Arabidopsis thaliana alternative oxidase 1D mRNA, complete cds
AT4G03450	-1.215661246	1.07E-07	Arabidopsis thaliana ankyrin repeat-containing protein mRNA, complete cds
AT3G47710	-1.302041389	1.13E-07	Arabidopsis thaliana atypical non-DNA binding bHLH protein BNQ3 mRNA, complete cds
AT1G34180	-1.634329995	1.37E-07	Arabidopsis thaliana NAC domain containing protein 16 mRNA, complete cds
AT4G05170	-3.840780873	1.61E-07	Arabidopsis thaliana transcription factor bHLH114 mRNA, complete cds
AT5G38120	-1.416283044	1.66E-07	Arabidopsis thaliana 4-coumarate--CoA ligase-like 8 mRNA, complete cds
AT2G47670	-1.741245199	1.78E-07	Arabidopsis thaliana chromosome 2, complete

			sequence
AT1G21326	-2.859889696	1.94E-07	Arabidopsis thaliana chromosome 1 sequence
AT3G62160	-1.043012	2.17E-07	Arabidopsis thaliana HXXXD-type acyl-transferase-like protein mRNA, complete cds
AT5G55110	-4.634329995	2.90E-07	Arabidopsis thaliana chromosome 5 sequence
AT4G11521	-1.29058379	2.98E-07	Arabidopsis thaliana chromosome 4 sequence
AT5G55090	-1.236453047	3.48E-07	Arabidopsis thaliana chromosome 5 sequence
AT3G49620	-2.287527232	4.24E-07	Arabidopsis thaliana 2-oxoacid-dependent dioxygenase-like protein DIN11 mRNA, complete cds
AT1G23840	-1.249092509	4.59E-07	Arabidopsis thaliana chromosome 1 sequence
AT3G26830	-1.051073816	4.63E-07	Arabidopsis thaliana protein PHYTOALEXIN DEFICIENT 3 mRNA, complete cds
AT5G53380	-5.518852778	4.77E-07	Arabidopsis thaliana O-acyltransferase (WSD1-like) family protein mRNA, complete cds
AT5G55250	-1.223396894	4.90E-07	Arabidopsis thaliana IAA carboxylmethyltransferase 1 mRNA, complete cds
AT4G31940	-4.577746467	5.23E-07	Arabidopsis thaliana cytochrome P450, family 82, subfamily C, polypeptide 4 mRNA, complete cds
AT1G76070	-1.098277095	5.25E-07	Arabidopsis thaliana chromosome 1 sequence
AT5G45660	-1.026336526	5.35E-07	Arabidopsis thaliana uncharacterized protein mRNA, complete cds
AT5G42580	-1.026336526	5.35E-07	Arabidopsis thaliana cytochrome P450, family 705, subfamily A, polypeptide 12 mRNA, complete cds
AT4G18425	-1.300672608	6.34E-07	Arabidopsis thaliana chromosome 4 sequence
AT2G41970	-1.537961601	6.44E-07	Arabidopsis thaliana putative protein kinase mRNA, complete cds
AT3G04060	-1.12151728	6.94E-07	Arabidopsis thaliana NAC domain containing protein 46 mRNA, complete cds
AT2G27690	-1.741245199	7.30E-07	Arabidopsis thaliana chromosome 2, complete sequence
AT4G10350	-2.59685529	7.56E-07	Arabidopsis thaliana protein BEARSKIN 2 mRNA, complete cds
AT5G59070	-1.419317104	8.25E-07	Arabidopsis thaliana glycosyl transferase family protein mRNA, complete cds
AT5G52050	-1.044073195	8.48E-07	Arabidopsis thaliana chromosome 5 sequence
AT2G28860	-1.5002371	8.69E-07	Arabidopsis thaliana chromosome 2, complete sequence
AT3G29000	-1.5002371	8.69E-07	Arabidopsis thaliana chromosome 3, complete sequence
AT1G13130	-2.44846345	9.12E-07	Arabidopsis thaliana Cellulase (glycosyl hydrolase family 5) protein mRNA, complete cds
AT1G67105	-2.44846345	9.12E-07	Arabidopsis thaliana chromosome 1 sequence
AT1G07550	-1.656356302	9.88E-07	Arabidopsis thaliana putative LRR receptor-like

			serine/threonine-protein kinase mRNA, complete cds
AT3G50120	-2.3262077	1.05E-06	Arabidopsis thaliana uncharacterized protein mRNA, complete cds
AT1G30757	-1.685962764	1.06E-06	Arabidopsis thaliana chromosome 1 sequence
AT1G75250	-1.263197902	1.12E-06	Arabidopsis thaliana protein RADIALIS-like 6 mRNA, complete cds
AT2G15370	-1.080731665	1.32E-06	Arabidopsis thaliana probable fucosyltransferase 5 mRNA, complete cds
AT5G51580	-1.607662045	1.39E-06	Arabidopsis thaliana chromosome 5 sequence
AT3G57157	-1.430316103	1.52E-06	Arabidopsis thaliana chromosome 3, complete sequence
AT4G27657	-1.384551686	1.75E-06	Arabidopsis thaliana chromosome 4 sequence
AT1G70420	-1.103815279	1.76E-06	Arabidopsis thaliana chromosome 1 sequence
AT4G29800	-1.085893371	1.79E-06	Arabidopsis thaliana PATATIN-like protein 8 mRNA, complete cds
AT3G03280	-1.963637621	2.14E-06	Arabidopsis thaliana chromosome 3, complete sequence
AT3G21500	-5.3262077	2.60E-06	Arabidopsis thaliana 1-deoxy-D-xylulose 5-phosphate synthase 1 mRNA, complete cds
AT1G33760	-5.3262077	2.60E-06	Arabidopsis thaliana chromosome 1 sequence
AT4G14780	-1.134188928	2.69E-06	Arabidopsis thaliana protein kinase family protein mRNA, complete cds
AT1G52830	-2.656356302	3.06E-06	Arabidopsis thaliana indole-3-acetic acid 6 mRNA, complete cds
AT4G21340	-2.156282698	3.15E-06	Arabidopsis thaliana transcription factor bHLH103 mRNA, complete cds
AT5G42440	-1.00206178	3.25E-06	Arabidopsis thaliana chromosome 5 sequence
AT5G45120	-2.071393801	3.31E-06	Arabidopsis thaliana chromosome 5 sequence
AT2G44840	-1.998020615	3.40E-06	Arabidopsis thaliana chromosome 2, complete sequence
AT1G61340	-1.407821465	3.67E-06	Arabidopsis thaliana F-box stress induced 1 mRNA, complete cds
AT5G19870	-1.407821465	3.67E-06	Arabidopsis thaliana chromosome 5 sequence
AT1G75030	-1.423275118	4.12E-06	Arabidopsis thaliana thaumatin-like protein 3 mRNA, complete cds
AT2G47360	-1.084449954	4.47E-06	Arabidopsis thaliana chromosome 2, complete sequence
AT3G13950	-5.255818372	4.62E-06	Arabidopsis thaliana uncharacterized protein mRNA, complete cds
AT4G08040	-1.239698707	4.83E-06	Arabidopsis thaliana 1-aminocyclopropane-1-carboxylate synthase 11 mRNA, complete cds
AT5G01700	-1.151481712	4.86E-06	Arabidopsis thaliana putative protein phosphatase 2C 65 mRNA, complete cds

AT5G44350	-1.566158493	5.22E-06	Arabidopsis thaliana chromosome 5 sequence
AT2G21900	-2.611962182	5.27E-06	Arabidopsis thaliana putative WRKY transcription factor 59 mRNA, complete cds
AT2G16970	-1.132670141	5.81E-06	Arabidopsis thaliana tetracycline transporter-like protein 1 mRNA, complete cds
AT3G49330	-1.187646869	6.24E-06	Arabidopsis thaliana chromosome 3, complete sequence
AT1G25430	-1.223396894	6.82E-06	Arabidopsis thaliana chromosome 1 sequence
AT5G22460	-1.114462523	6.90E-06	Arabidopsis thaliana esterase/lipase/thioesterase family protein mRNA, complete cds
AT4G12090	-1.093546943	7.03E-06	Arabidopsis thaliana Cornichon family protein mRNA, complete cds
AT5G02780	-1.518852778	7.11E-06	Arabidopsis thaliana glutathione transferase lambda 1 mRNA, complete cds
AT2G01300	-1.038859837	7.13E-06	Arabidopsis thaliana chromosome 2, complete sequence
AT3G07000	-1.038859837	7.13E-06	Arabidopsis thaliana chromosome 3, complete sequence
AT4G01970	-1.055880801	7.14E-06	Arabidopsis thaliana stachyose synthase mRNA, complete cds
ATCG00080	-2.296460357	7.29E-06	Cardamine resedifolia plastid, complete genome
AT2G28710	-1.264535589	7.31E-06	Arabidopsis thaliana C2H2-type zinc finger-containing protein mRNA, complete cds
AT3G10930	-1.7274394	7.64E-06	Arabidopsis thaliana chromosome 3, complete sequence
AT4G15417	-1.54269952	7.85E-06	Arabidopsis thaliana protein RNAse II-like 1 mRNA, complete cds
AT4G24000	-1.770391545	8.05E-06	Arabidopsis thaliana cellulose synthase-like protein G2 mRNA, complete cds
AT4G01140	-1.076848231	8.26E-06	Arabidopsis thaliana chromosome 4 sequence
AT1G33813	-2.085893371	8.43E-06	Arabidopsis thaliana chromosome 1 sequence
AT2G38790	-1.146193881	9.05E-06	Arabidopsis thaliana chromosome 2, complete sequence
AT4G13860	-1.126535355	1.08E-05	Arabidopsis thaliana RNA recognition motif-containing protein mRNA, complete cds
AT5G23990	-1.661810732	1.10E-05	Arabidopsis thaliana ferric reduction oxidase 5 mRNA, complete cds
AT4G06534	-1.103815279	1.10E-05	Arabidopsis thaliana chromosome 4 sequence
AT4G10540	-1.213998196	1.11E-05	Arabidopsis thaliana Subtilase family protein mRNA, complete cds
AT3G49070	-1.415016967	1.12E-05	Arabidopsis thaliana chromosome 3, complete sequence
AT1G16370	-1.255818372	1.19E-05	Arabidopsis thaliana chromosome 1 sequence
AT3G50460	-1.107538365	1.28E-05	Arabidopsis thaliana RPW8-like protein 2 mRNA,

			complete cds
AT5G63130	-2.143343643	1.31E-05	Arabidopsis thaliana octicosapeptide/Phox/Bem1p domain-containing protein mRNA, complete cds
AT5G46040	-1.265096186	1.37E-05	Arabidopsis thaliana peptide transporter PTR3-B mRNA, complete cds
AT4G01890	-1.969514187	1.40E-05	Arabidopsis thaliana glycoside hydrolase family 28 protein / polygalacturonase (pectinase) family protein mRNA, complete cds
AT1G74290	-1.451738582	1.41E-05	Arabidopsis thaliana esterase/lipase/thioesterase family protein mRNA, complete cds
AT3G22275	-5.103815279	1.47E-05	Arabidopsis thaliana uncharacterized protein mRNA, complete cds
AT2G32610	-1.328749894	1.63E-05	Arabidopsis thaliana cellulose synthase-like protein B1 mRNA, complete cds
AT1G15580	-1.393321896	1.64E-05	Arabidopsis thaliana auxin-responsive protein IAA5 mRNA, complete cds
AT5G61360	-1.634329995	1.69E-05	Arabidopsis thaliana uncharacterized protein mRNA, complete cds
AT5G14020	-1.016352437	1.77E-05	Arabidopsis thaliana Endosomal targeting BRO1-like domain-containing protein mRNA, complete cds
AT3G10320	-1.285362648	1.80E-05	Arabidopsis thaliana Glycosyltransferase family 61 protein mRNA, complete cds
AT4G27652	-1.670855871	1.82E-05	Arabidopsis thaliana chromosome 4 sequence
AT1G71740	-1.808359395	2.14E-05	Arabidopsis thaliana chromosome 1 sequence
AT5G42785	-1.866776081	2.21E-05	Arabidopsis thaliana uncharacterized protein mRNA, complete cds
AT3G22240	-1.099900229	2.39E-05	Arabidopsis thaliana uncharacterized protein mRNA, complete cds
AT1G43160	-1.371295589	2.39E-05	Arabidopsis thaliana ethylene-responsive transcription factor RAP2-6 mRNA, complete cds
AT2G39030	-5.021353118	2.64E-05	Arabidopsis thaliana chromosome 2, complete sequence
AT1G33280	-2.469943177	2.65E-05	Arabidopsis thaliana protein BEARSKIN1 mRNA, complete cds
AT1G23965	-2.469943177	2.65E-05	Arabidopsis thaliana chromosome 1 sequence
AT4G14380	-1.493317686	2.91E-05	Arabidopsis thaliana chromosome 4 sequence
AT2G02610	-1.493317686	2.91E-05	Arabidopsis thaliana chromosome 2, complete sequence
AT1G19320	-2.303124087	3.06E-05	Arabidopsis thaliana pathogenesis-related thaumatin-like protein mRNA, complete cds
AT4G37235	-1.725303655	3.19E-05	Arabidopsis thaliana CASP-like protein mRNA, complete cds
AT1G35625	-1.424215904	3.44E-05	Arabidopsis thaliana RING/U-box domain-containing protein mRNA, complete cds

AT1G47590	-1.897364401	3.58E-05	Arabidopsis thaliana chromosome 1 sequence
AT1G33320	-1.973418641	3.59E-05	Arabidopsis thaliana Pyridoxal phosphate (PLP)-dependent transferases superfamily protein mRNA, complete cds
AT2G31310	-2.634329995	3.73E-05	Arabidopsis thaliana LOB domain-containing protein 14 mRNA, complete cds
AT1G13430	-1.007139259	3.80E-05	Arabidopsis thaliana chromosome 1 sequence
AT1G53700	-1.087695613	3.81E-05	Arabidopsis thaliana chromosome 1 sequence
AT2G24850	-1.444852196	3.86E-05	Arabidopsis thaliana tyrosine aminotransferase 3 mRNA, complete cds
AT2G29740	-1.026999682	4.48E-05	Arabidopsis thaliana chromosome 2, complete sequence
AT4G13130	-1.21682424	4.50E-05	Arabidopsis thaliana chromosome 4 sequence
AT2G32200	-2.419317104	4.52E-05	Arabidopsis thaliana uncharacterized protein mRNA, complete cds
AT4G13395	-4.933890277	4.75E-05	Arabidopsis thaliana chromosome 4 sequence
AT2G23960	-1.225656401	5.18E-05	Arabidopsis thaliana class I glutamine amidotransferase-like superfamily protein mRNA, complete cds
AT4G40010	-1.071393801	5.23E-05	Arabidopsis thaliana SNF1-related protein kinase 2.7 mRNA, complete cds
AT3G46700	-1.071393801	5.23E-05	Arabidopsis thaliana UDP-glycosyltransferase 76E3 mRNA, complete cds
AT5G07780	-1.049367495	5.24E-05	Arabidopsis thaliana formin-like protein 19 mRNA, complete cds
AT1G73290	-1.796386753	5.51E-05	Arabidopsis thaliana serine carboxypeptidase-like 5 mRNA, complete cds
AT5G43890	-2.021353118	5.72E-05	Arabidopsis thaliana chromosome 5 sequence
AT1G61480	-1.933890277	5.77E-05	Arabidopsis thaliana G-type lectin S-receptor-like serine/threonine-protein kinase mRNA, complete cds
AT3G14440	-1.235059812	5.97E-05	Arabidopsis thaliana chromosome 3, complete sequence
AT2G47485	-1.031187478	6.10E-05	Arabidopsis thaliana chromosome 2, complete sequence
AT5G12340	-1.052071703	6.12E-05	Arabidopsis thaliana chromosome 5 sequence
AT5G28237	-2.577746467	6.45E-05	Arabidopsis thaliana tryptophan synthase beta chain-like protein mRNA, complete cds
AT3G27884	-1.581588533	6.47E-05	Arabidopsis thaliana clone 102688 mRNA sequence
AT3G20935	-1.357101708	6.52E-05	Arabidopsis thaliana cytochrome P450, family 705, subfamily A, polypeptide 28 mRNA, complete cds
AT3G18777	-1.103815279	7.00E-05	Arabidopsis thaliana chromosome 3, complete sequence
AT2G01275	-1.618388451	7.05E-05	Arabidopsis thaliana RING/FYVE/PHD zinc finger-containing protein mRNA, complete cds

AT5G50760	-1.659715314	7.62E-05	Arabidopsis thaliana chromosome 5 sequence
AT1G16820	-1.706479781	8.16E-05	Arabidopsis thaliana V-ATPase-related protein mRNA, complete cds
AT3G47050	-1.176746801	8.90E-05	Arabidopsis thaliana glycosyl hydrolase family protein mRNA, complete cds
AT3G32047	-1.821415548	9.01E-05	Arabidopsis thaliana cytochrome P450 superfamily protein mRNA, complete cds
AT1G64910	-1.267314011	9.09E-05	Arabidopsis thaliana chromosome 1 sequence
AT4G25470	-1.978284396	9.27E-05	Arabidopsis thaliana chromosome 4 sequence
AT5G43620	-1.413883218	9.45E-05	Arabidopsis thaliana chromosome 5 sequence
AT5G59930	-1.085893371	9.64E-05	Arabidopsis thaliana chromosome 5 sequence
AT5G59845	-1.550561638	9.80E-05	Arabidopsis thaliana gibberellin-regulated protein mRNA, complete cds
AT5G66400	-1.550561638	9.80E-05	Arabidopsis thaliana dehydrin Rab18 mRNA, complete cds
AT5G51870	-1.585966974	0.000107466	Arabidopsis thaliana MADS-box transcription factor AGL71 mRNA, complete cds
AT3G01760	-1.348927776	0.000107855	Arabidopsis thaliana Lysine histidine transporter-like 4 mRNA, complete cds
AT2G20562	-1.348927776	0.000107855	Arabidopsis thaliana uncharacterized protein mRNA, complete cds
AT2G22790	-1.348927776	0.000107855	Arabidopsis thaliana chromosome 2, complete sequence
AT1G79320	-1.118314848	0.000110376	Arabidopsis thaliana metacaspase 6 mRNA, complete cds
AT3G08870	-1.019281768	0.000112766	Arabidopsis thaliana chromosome 3, complete sequence
AT5G42930	-1.46113728	0.00011915	Arabidopsis thaliana lipase class 3-like protein mRNA, complete cds
AT4G30420	-1.094354949	0.000130921	Arabidopsis thaliana nodulin MtN21-like transporter family protein mRNA, complete cds
AT1G23120	-1.067746024	0.000132242	Arabidopsis thaliana polyketide cyclase/dehydrase and lipid transport superfamily protein mRNA, complete cds
AT2G44010	-1.067746024	0.000132242	Arabidopsis thaliana chromosome 2, complete sequence
AT2G31980	-1.933890277	0.000149485	Arabidopsis thaliana cysteine proteinase inhibitor 2 mRNA, complete cds
AT5G24220	-2.741245199	0.000153253	Arabidopsis thaliana lipase class 3-related protein mRNA, complete cds
AT5G51470	-2.741245199	0.000153253	Arabidopsis thaliana auxin-responsive GH3 family protein mRNA, complete cds
AT4G27654	-4.741245199	0.000156595	Arabidopsis thaliana chromosome 4 sequence
AT1G72260	-4.741245199	0.000156595	Arabidopsis thaliana thionin 2.1 mRNA, complete cds

AT5G22410	-1.407821465	0.000156914	Arabidopsis thaliana peroxidase 60 mRNA, complete cds
AT3G46810	-1.210730482	0.000159578	Arabidopsis thaliana chromosome 3, complete sequence
AT1G77640	-1.55280011	0.000163014	Arabidopsis thaliana chromosome 1 sequence
AT1G32928	-1.431389937	0.00017673	Arabidopsis thaliana chromosome 1 sequence
AT1G70860	-1.431389937	0.00017673	Arabidopsis thaliana SRPBCC ligand-binding domain-containing protein mRNA, complete cds
AT2G35910	-1.103815279	0.000177726	Arabidopsis thaliana chromosome 2, complete sequence
AT5G64230	-1.004279605	0.0001782	Arabidopsis thaliana uncharacterized protein mRNA, complete cds
AT3G28007	-1.591002564	0.000178736	Arabidopsis thaliana bidirectional sugar transporter SWEET4 mRNA, complete cds
AT1G62262	-3.103815279	0.000193907	Arabidopsis thaliana SLAC1 homologue 4 mRNA, complete cds
AT3G25597	-1.634329995	0.000194369	Arabidopsis thaliana chromosome 3, complete sequence
AT1G73810	-1.683912024	0.000209283	Arabidopsis thaliana core-2/I-branching beta-1,6-N-acetylglucosaminyltransferase-like protein mRNA, complete cds
AT1G17610	-1.052534774	0.000211129	Arabidopsis thaliana chromosome 1 sequence
AT1G79250	-3.840780873	0.000211237	Arabidopsis thaliana AGC kinase 1.7 mRNA, complete cds
AT2G23410	-1.4864313	0.000221481	Arabidopsis thaliana cis-prenyltransferase mRNA, complete cds
AT3G52072	-2.103815279	0.000233962	Arabidopsis thaliana mRNA for hypothetical protein, complete cds, clone: RAFL21-36-M10
AT5G45580	-1.148903168	0.000234621	Arabidopsis thaliana homeodomain-like superfamily protein mRNA, complete cds
AT1G05100	-1.888086588	0.000239866	Arabidopsis thaliana chromosome 1 sequence
AT1G30560	-1.196924683	0.00026083	Arabidopsis thaliana chromosome 1 sequence
AT3G28890	-2.670855871	0.000268631	Arabidopsis thaliana receptor like protein 43 mRNA, complete cds
AT3G52970	-2.670855871	0.000268631	Arabidopsis thaliana cytochrome P450, family 76, subfamily G, polypeptide 1 mRNA, complete cds
AT5G67080	-2.670855871	0.000268631	Arabidopsis thaliana chromosome 5 sequence
AT5G14180	-1.555378654	0.000271722	Arabidopsis thaliana Myzus persicae-induced lipase 1 mRNA, complete cds
AT5G42460	-1.059421159	0.000288119	Arabidopsis thaliana chromosome 5 sequence
AT3G13437	-1.206908772	0.000301857	Arabidopsis thaliana uncharacterized protein mRNA, complete cds
AT1G71030	-1.164187897	0.000316553	Arabidopsis thaliana putative myb family transcription factor mRNA, complete cds

AT1G76430	-1.64438366	0.000323405	Arabidopsis thaliana putative inorganic phosphate transporter 1-9 mRNA, complete cds
AT5G46130	-2.393321896	0.000326559	Arabidopsis thaliana uncharacterized protein mRNA, complete cds
AT3G55515	-2.393321896	0.000326559	Arabidopsis thaliana chromosome 3, complete sequence
AT5G15900	-1.126535355	0.000326994	Arabidopsis thaliana protein trichome birefringence-like 19 mRNA, complete cds
AT5G15890	-1.453264436	0.000330595	Arabidopsis thaliana protein trichome birefringence-like 21 mRNA, complete cds
AT1G62510	-1.063173294	0.000336598	Arabidopsis thaliana chromosome 1 sequence
AT5G59940	-1.063173294	0.000336598	Arabidopsis thaliana chromosome 5 sequence
AT2G31230	-1.063173294	0.000336598	Arabidopsis thaliana chromosome 2, complete sequence
AT3G25950	-1.699425023	0.000347336	Arabidopsis thaliana chromosome 3, complete sequence
AT3G46760	-3.021353118	0.000348439	Arabidopsis thaliana chromosome 3, complete sequence
AT1G28040	-1.284387524	0.000373595	Arabidopsis thaliana RING-H2 finger protein ATL20 mRNA, complete cds
AT1G33870	-1.284387524	0.000373595	Arabidopsis thaliana P-loop containing nucleoside triphosphate hydrolases superfamily protein mRNA, complete cds
AT1G10070	-1.369989392	0.00038334	Arabidopsis thaliana branched-chain-amino-acid aminotransferase 2 mRNA, complete cds
AT4G19920	-1.369989392	0.00038334	Arabidopsis thaliana Toll-Interleukin-Resistance domain-containing protein mRNA, complete cds
AT4G25490	-3.741245199	0.000388987	Arabidopsis thaliana chromosome 4 sequence
AT2G30760	-3.741245199	0.000388987	Arabidopsis thaliana uncharacterized protein mRNA, complete cds
AT2G24040	-1.098277095	0.000389081	Arabidopsis thaliana Low temperature and salt responsive protein mRNA, complete cds
AT1G05575	-1.933890277	0.000389617	Arabidopsis thaliana chromosome 1 sequence
AT2G14210	-1.014060626	0.000390904	Arabidopsis thaliana protein agamous-like 44 mRNA, complete cds
AT1G33900	-1.014060626	0.000390904	Arabidopsis thaliana P-loop containing nucleoside triphosphate hydrolases superfamily protein mRNA, complete cds
AT1G68620	-1.016352437	0.000457614	Arabidopsis thaliana chromosome 1 sequence
AT4G14060	-1.016352437	0.000457614	Arabidopsis thaliana major latex protein-like protein mRNA, complete cds
AT3G15800	-1.042414734	0.000460491	Arabidopsis thaliana glycosyl hydrolase superfamily protein mRNA, complete cds
AT1G55390	-1.318554127	0.00049058	Arabidopsis thaliana chromosome 1 sequence

AT2G45570	-1.318554127	0.00049058	Arabidopsis thaliana cytochrome P450 76C2 mRNA, complete cds
AT1G17300	-1.148015082	0.000515399	Arabidopsis thaliana uncharacterized protein mRNA, complete cds
AT5G21960	-4.518852778	0.00052662	Arabidopsis thaliana chromosome 5 sequence
AT5G44260	-1.109740113	0.000529296	Arabidopsis thaliana chromosome 5 sequence
AT3G51540	-1.109740113	0.000529296	Arabidopsis thaliana uncharacterized protein mRNA, complete cds
AT4G17470	-1.255818372	0.00053669	Arabidopsis thaliana putative palmitoyl-protein thioesterase mRNA, complete cds
AT3G53840	-1.075909282	0.000536794	Arabidopsis thaliana wall-associated receptor kinase-like 15 mRNA, complete cds
AT5G63090	-1.075909282	0.000536794	Arabidopsis thaliana protein LATERAL ORGAN BOUNDARIES mRNA, complete cds
AT5G52270	-2.3262077	0.0005562	Arabidopsis thaliana SNARE-like superfamily protein mRNA, complete cds
AT5G47980	-1.791871272	0.000607945	Arabidopsis thaliana chromosome 5 sequence
AT4G37700	-1.270925264	0.00061791	Arabidopsis thaliana chromosome 4 sequence
AT2G43310	-1.881422857	0.000627238	Arabidopsis thaliana chromosome 2, complete sequence
AT3G50800	-1.021353118	0.000627433	Arabidopsis thaliana chromosome 3, complete sequence
AT3G44700	-1.992783966	0.000629398	Arabidopsis thaliana uncharacterized protein mRNA, complete cds
AT5G50560	-1.049367495	0.000630242	Arabidopsis thaliana chromosome 5 sequence
AT1G11185	-1.213998196	0.000663233	Arabidopsis thaliana chromosome 1 sequence
AT2G34340	-1.518852778	0.000687961	Arabidopsis thaliana chromosome 2, complete sequence
AT5G11610	-1.287527232	0.000710304	Arabidopsis thaliana Exostosin family protein mRNA, complete cds
AT4G28530	-1.384551686	0.000725288	Arabidopsis thaliana NAC domain containing protein 74 mRNA, complete cds
AT4G00960	-1.384551686	0.000725288	Arabidopsis thaliana protein kinase family protein mRNA, complete cds
AT5G39610	-1.085893371	0.00073275	Arabidopsis thaliana NAC-domain transcription factor mRNA, complete cds
AT1G56630	-1.053189205	0.000737421	Arabidopsis thaliana alpha/beta-Hydrolases superfamily protein mRNA, complete cds
AT1G23160	-1.174898377	0.000809693	Arabidopsis thaliana auxin-responsive GH3 family protein mRNA, complete cds
AT3G48520	-1.611962182	0.000831687	Arabidopsis thaliana chromosome 3, complete sequence
AT2G03600	-1.670855871	0.000899939	Arabidopsis thaliana ureide permease 3 mRNA, complete cds

AT5G58784	-1.670855871	0.000899939	Arabidopsis thaliana dehydrodolichyl diphosphate synthase 5 mRNA, complete cds
AT4G30640	-1.3262077	0.00093312	Arabidopsis thaliana RNI-like superfamily protein mRNA, complete cds
AT1G05530	-1.185429044	0.000940279	Arabidopsis thaliana chromosome 1 sequence
AT5G44920	-1.185429044	0.000940279	Arabidopsis thaliana Toll-Interleukin-Resistance domain-containing protein mRNA, complete cds
AT5G20810	-2.255818372	0.000942752	Arabidopsis thaliana SAUR-like auxin-responsive protein mRNA, complete cds
AT5G64810	-1.741245199	0.000959654	Arabidopsis thaliana putative WRKY transcription factor 51 mRNA, complete cds
AT4G11310	-4.393321896	0.000973747	Arabidopsis thaliana putative cysteine proteinase mRNA, complete cds
AT5G66650	-1.00206178	0.000998225	Arabidopsis thaliana uncharacterized protein mRNA, complete cds
AT3G09960	-1.097389009	0.001000129	Arabidopsis thaliana calcineurin-like metallo-phosphoesterase-like protein mRNA, complete cds
AT1G73580	-1.826975073	0.001003974	Arabidopsis thaliana C2 domain-containing protein mRNA, complete cds
AT5G46845	-1.826975073	0.001003974	Arabidopsis thaliana chromosome 5 sequence
AT2G41850	-2.071393801	0.001007753	Arabidopsis thaliana polygalacturonase ADPG2 mRNA, complete cds
AT4G14450	-2.071393801	0.001007753	Arabidopsis thaliana chromosome 4 sequence
AT2G19660	-1.030105592	0.0010086	Arabidopsis thaliana chromosome 2, complete sequence
AT5G09670	-1.933890277	0.001023808	Arabidopsis thaliana chromosome 5 sequence
AT1G16515	-1.933890277	0.001023808	Arabidopsis thaliana chromosome 1 sequence
AT5G65070	-1.348927776	0.001065592	Arabidopsis thaliana protein MADS AFFECTING FLOWERING 4 mRNA, complete cds
AT3G13403	-2.840780873	0.00111802	Arabidopsis thaliana defensin-like protein 302 mRNA, complete cds
AT1G19968	-2.840780873	0.00111802	Arabidopsis thaliana chromosome 1 sequence
AT3G45638	-2.840780873	0.00111802	Arabidopsis thaliana clone asmbl_10177 unknown mRNA sequence
AT2G35210	-1.103815279	0.001168327	Arabidopsis thaliana root and pollen arfgap mRNA, complete cds
AT1G62045	-1.272692191	0.001178955	Arabidopsis thaliana uncharacterized protein mRNA, complete cds
AT5G56230	-1.566158493	0.001275686	Arabidopsis thaliana chromosome 5 sequence
AT3G22250	-3.518852778	0.001325341	Arabidopsis thaliana UDP-glycosyltransferase 82A1 mRNA, complete cds
AT3G53010	-1.156282698	0.0013263	Arabidopsis thaliana uncharacterized protein mRNA, complete cds

AT2G14095	-1.40337556	0.001375922	Arabidopsis thaliana uncharacterized protein mRNA, complete cds
AT1G30845	-1.40337556	0.001375922	Arabidopsis thaliana uncharacterized protein mRNA, complete cds
AT1G70985	-1.071393801	0.001383336	Arabidopsis thaliana chromosome 1 sequence
AT1G51260	-1.621946271	0.001394982	Arabidopsis thaliana 1-acyl-sn-glycerol-3-phosphate acyltransferase 3 mRNA, complete cds
AT5G54030	-1.621946271	0.001394982	Arabidopsis thaliana chromosome 5 sequence
AT3G13275	-1.223396894	0.001465716	Arabidopsis thaliana chromosome 3, complete sequence
AT1G14860	-1.166551034	0.001543855	Arabidopsis thaliana nudix hydrolase 18 mRNA, complete cds
AT4G14305	-1.166551034	0.001543855	Arabidopsis thaliana Mpv17/PMP22 domain-containing protein mRNA, complete cds
AT1G32690	-1.436390618	0.001553487	Arabidopsis thaliana chromosome 1 sequence
AT1G63820	-1.3124019	0.001556668	Arabidopsis thaliana CCT motif family protein mRNA, complete cds
AT5G65500	-2.181817791	0.001589454	Arabidopsis thaliana U-box domain-containing protein kinase family protein mRNA, complete cds
AT4G25400	-1.770391545	0.001597119	Arabidopsis thaliana transcription factor bHLH118 mRNA, complete cds
AT3G45330	-1.009178404	0.00161142	Arabidopsis thaliana chromosome 3, complete sequence
AT5G38240	-1.040805481	0.001623913	Arabidopsis thaliana Protein kinase family protein mRNA, complete cds
AT1G07430	-1.872489732	0.001655277	Arabidopsis thaliana protein phosphatase 2C 3 mRNA, complete cds
AT5G25910	-2.004279605	0.001660762	Arabidopsis thaliana receptor like protein 52 mRNA, complete cds
AT3G04530	-1.238744859	0.001695968	Arabidopsis thaliana phosphoenolpyruvate carboxylase kinase 2 mRNA, complete cds
AT4G11020	-1.474458658	0.001744136	Arabidopsis thaliana uncharacterized protein mRNA, complete cds
AT1G46552	-1.474458658	0.001744136	Arabidopsis thaliana chromosome 1 sequence
AT5G02170	-1.335988721	0.001782274	Arabidopsis thaliana transmembrane amino acid transporter family protein mRNA, complete cds
AT1G69720	-1.17781586	0.001796098	Arabidopsis thaliana heme oxygenase 3 mRNA, complete cds
AT5G65300	-1.17781586	0.001796098	Arabidopsis thaliana chromosome 5 sequence
AT5G45105	-4.255818372	0.001811226	Arabidopsis thaliana zinc transporter 8 precursor mRNA, complete cds
AT1G50930	-4.255818372	0.001811226	Arabidopsis thaliana uncharacterized protein mRNA, complete cds
AT1G47630	-4.255818372	0.001811226	Arabidopsis thaliana chromosome 1 sequence

AT2G22750	-4.255818372	0.001811226	Arabidopsis thaliana transcription factor bHLH18 mRNA, complete cds
AT3G57950	-1.04492159	0.001904008	Arabidopsis thaliana chromosome 3, complete sequence
AT3G50280	-1.04492159	0.001904008	Arabidopsis thaliana chromosome 3, complete sequence
AT1G15900	-2.741245199	0.001994553	Arabidopsis thaliana chromosome 1 sequence
ATCG00220	-1.089168503	0.002218776	Olimarabidopsis pumila chloroplast DNA, complete sequence
AT4G23030	-1.634329995	0.002346619	Arabidopsis thaliana chromosome 4 sequence
AT3G28650	-1.20397944	0.002425691	Arabidopsis thaliana chromosome 3, complete sequence
AT2G05518	-1.20397944	0.002425691	Arabidopsis thaliana mRNA, clone: RAFL17-49-H20
AT2G37780	-1.20397944	0.002425691	Arabidopsis thaliana chromosome 2, complete sequence
AT1G73120	-2.348927776	0.002439617	Arabidopsis thaliana uncharacterized protein mRNA, complete cds
AT5G22150	-3.393321896	0.002451299	Arabidopsis thaliana uncharacterized protein mRNA, complete cds
AT1G72590	-3.393321896	0.002451299	Arabidopsis thaliana 3-oxo-5-alpha-steroid 4-dehydrogenase family protein mRNA, complete cds
AT2G29470	-3.393321896	0.002451299	Arabidopsis thaliana glutathione S-transferase tau 3 mRNA, complete cds
AT1G74080	-2.103815279	0.002664046	Arabidopsis thaliana myb domain protein 122 mRNA, complete cds
AT3G22820	-1.933890277	0.002719559	Arabidopsis thaliana allergen-related protein mRNA, complete cds
AT1G51480	-1.933890277	0.002719559	Arabidopsis thaliana CC-NBS-LRR class disease resistance protein mRNA, complete cds
AT1G06160	-1.219292496	0.002814975	Arabidopsis thaliana chromosome 1 sequence
AT1G63600	-1.156282698	0.002957775	Arabidopsis thaliana chromosome 1 sequence
AT2G15760	-1.3209134	0.002988538	Arabidopsis thaliana chromosome 2, complete sequence
AT1G69150	-1.518852778	0.003286599	Arabidopsis thaliana chromosome 1 sequence
AT2G32179	-4.103815279	0.003390297	Arabidopsis thaliana uncharacterized protein mRNA, complete cds
AT1G48070	-4.103815279	0.003390297	Arabidopsis thaliana TRX domain-containing protein mRNA, complete cds
AT1G34500	-4.103815279	0.003390297	Arabidopsis thaliana chromosome 1 sequence
AT1G15010	-1.348927776	0.003421263	Arabidopsis thaliana chromosome 1 sequence
AT3G02840	-1.168355531	0.003448723	Arabidopsis thaliana chromosome 3, complete sequence
AT1G69990	-2.634329995	0.003545999	Arabidopsis thaliana chromosome 1 sequence

AT3G18880	-1.112227518	0.003558533	Arabidopsis thaliana Nucleic acid-binding, OB-fold-like protein mRNA, complete cds
AT3G01175	-1.025038165	0.003599128	Arabidopsis thaliana uncharacterized protein mRNA, complete cds
AT4G05100	-1.255818372	0.003775905	Arabidopsis thaliana myb domain protein 74 mRNA, complete cds
AT4G15248	-1.255818372	0.003775905	Arabidopsis thaliana chromosome 4 sequence
AT2G43600	-1.381349254	0.003901443	Arabidopsis thaliana chitinase family protein mRNA, complete cds
AT5G57400	-1.650097311	0.003960786	Arabidopsis thaliana uncharacterized protein mRNA, complete cds
AT3G54070	-1.650097311	0.003960786	Arabidopsis thaliana ankyrin repeat-containing protein mRNA, complete cds
AT1G70110	-1.650097311	0.003960786	Arabidopsis thaliana putative L-type lectin-domain containing receptor kinase V.1 mRNA, complete cds
AT1G65680	-2.255818372	0.004183124	Arabidopsis thaliana expansin B2 mRNA, complete cds
AT3G02430	-2.255818372	0.004183124	Arabidopsis thaliana chromosome 3, complete sequence
AT2G44700	-1.741245199	0.004240445	Arabidopsis thaliana chromosome 2, complete sequence
AT1G55290	-1.741245199	0.004240445	Arabidopsis thaliana feruloyl CoA ortho-hydroxylase 2 mRNA, complete cds
AT2G13960	-1.277844678	0.004361533	Arabidopsis thaliana homeodomain-like superfamily protein mRNA, complete cds
AT2G43870	-1.859889696	0.004422594	Arabidopsis thaliana putative polygalacturonase /pectinase mRNA, complete cds
AT3G46410	-1.859889696	0.004422594	Arabidopsis thaliana protein kinase family protein mRNA, complete cds
AT4G12350	-1.859889696	0.004422594	Arabidopsis thaliana myb domain protein 42 mRNA, complete cds
AT1G26360	-1.419317104	0.00442706	Arabidopsis thaliana methyl esterase 13 mRNA, complete cds
AT5G39680	-1.419317104	0.00442706	Arabidopsis thaliana chromosome 5 sequence
AT1G44010	-1.419317104	0.00442706	Arabidopsis thaliana uncharacterized protein mRNA, complete cds
AT4G16220	-1.419317104	0.00442706	Arabidopsis thaliana GDSL esterase/lipase mRNA, complete cds
AT1G35310	-3.255818372	0.004538298	Arabidopsis thaliana MLP-like protein 168 mRNA, complete cds
AT4G09780	-3.255818372	0.004538298	Arabidopsis thaliana TRAF-like family protein mRNA, complete cds
AT1G58430	-3.255818372	0.004538298	Arabidopsis thaliana anther-specific proline-rich protein RXF26 mRNA, complete cds

AT4G34380	-1.196924683	0.004678871	Arabidopsis thaliana chromosome 4 sequence
AT5G38005	-1.131829655	0.004872916	Arabidopsis thaliana Unknown protein mRNA, partial cds
AT2G46495	-1.078280186	0.004964726	Arabidopsis thaliana putative RING-H2 finger protein mRNA, complete cds
AT5G11180	-1.078280186	0.004964726	Arabidopsis thaliana glutamate receptor 2.6 mRNA, complete cds
AT5G43030	-1.078280186	0.004964726	Arabidopsis thaliana chromosome 5 sequence
AT3G17890	-1.033425951	0.004974211	Arabidopsis thaliana uncharacterized protein mRNA, complete cds
AT1G31835	-1.033425951	0.004974211	Arabidopsis thaliana chromosome 1 sequence
AT3G61930	-1.464404994	0.004991457	Arabidopsis thaliana chromosome 3, complete sequence
AT1G65484	-1.464404994	0.004991457	Arabidopsis thaliana uncharacterized protein mRNA, complete cds
AT1G34050	-1.518852778	0.005580549	Arabidopsis thaliana ankyrin repeats-containing protein mRNA, complete cds
AT2G07671	-1.332439654	0.005774083	Arabidopsis thaliana ecotype Col-0 mitochondrion, complete genome
AT2G09795	-1.085893371	0.005827617	Arabidopsis thaliana Full-length cDNA Complete sequence from clone GSLTFB3ZA09 of Flowers and buds of strain col-0 of Arabidopsis thaliana (thale cress)
AT5G03795	-1.085893371	0.005827617	Arabidopsis thaliana Exostosin family protein mRNA, complete cds
AT1G48670	-1.585966974	0.006168559	Arabidopsis thaliana auxin-responsive GH3 family protein mRNA, complete cds
AT2G20700	-1.585966974	0.006168559	Arabidopsis thaliana LORELEI-like glucosylphosphatidylinositol-anchored protein 2 mRNA, complete cds
AT1G75590	-1.233450559	0.006321342	Arabidopsis thaliana chromosome 1 sequence
AT2G24545	-3.933890277	0.006388922	Arabidopsis thaliana chromosome 2, complete sequence
AT4G30730	-3.933890277	0.006388922	Arabidopsis thaliana chromosome 4 sequence
AT5G09910	-3.933890277	0.006388922	Arabidopsis thaliana Ras-related small GTP-binding family protein mRNA, complete cds
AT5G61100	-3.933890277	0.006388922	Arabidopsis thaliana uncharacterized protein mRNA, complete cds
AT1G02440	-1.366849684	0.006607638	Arabidopsis thaliana ADP-ribosylation factor D1A mRNA, complete cds
AT5G35525	-1.366849684	0.006607638	Arabidopsis thaliana PLAC8 family protein mRNA, complete cds
AT1G10585	-1.670855871	0.006711549	Arabidopsis thaliana basic helix-loop-helix domain-containing protein mRNA, complete cds

AT1G03020	-1.001004473	0.006823443	Arabidopsis thaliana chromosome 1 sequence
AT5G22380	-2.156282698	0.007127542	Arabidopsis thaliana NAC domain-containing protein mRNA, complete cds
AT2G38240	-2.156282698	0.007127542	Arabidopsis thaliana 2-oxoglutarate (2OG) and Fe(II)-dependent oxygenase-like protein mRNA, complete cds
AT1G57570	-1.781887184	0.007137759	Arabidopsis thaliana jacalin-like plant lectin domain-containing protein mRNA, complete cds
AT2G37740	-1.781887184	0.007137759	Arabidopsis thaliana chromosome 2, complete sequence
AT1G71890	-1.781887184	0.007137759	Arabidopsis thaliana sucrose transport protein SUC5 mRNA, complete cds
AT4G23700	-1.255818372	0.007330321	Arabidopsis thaliana cation/H(+) antiporter 17 mRNA, complete cds
AT1G08310	-1.255818372	0.007330321	Arabidopsis thaliana alpha/beta-Hydrolases superfamily protein mRNA, complete cds
AT1G52660	-1.255818372	0.007330321	Arabidopsis thaliana probable disease resistance protein mRNA, complete cds
AT4G26380	-1.933890277	0.007333811	Arabidopsis thaliana cysteine/histidine-rich C1 domain-containing protein mRNA, complete cds
AT3G22961	-1.933890277	0.007333811	Arabidopsis thaliana chromosome 3, complete sequence
AT1G77960	-1.933890277	0.007333811	Arabidopsis thaliana uncharacterized protein mRNA, complete cds
AT1G72760	-1.407821465	0.007522878	Arabidopsis thaliana putative serine/threonine protein kinase mRNA, complete cds
AT1G73410	-1.170929474	0.007788893	Arabidopsis thaliana myb domain protein 54 mRNA, complete cds
AT2G35585	-1.103815279	0.008031173	Arabidopsis thaliana uncharacterized protein mRNA, complete cds
AT2G29710	-1.103815279	0.008031173	Arabidopsis thaliana chromosome 2, complete sequence
AT4G32785	-1.004279605	0.008042423	Arabidopsis thaliana uncharacterized protein mRNA, complete cds
AT1G35330	-1.004279605	0.008042423	Arabidopsis thaliana RING-H2 finger protein ATL34 mRNA, complete cds
AT1G74000	-1.049367495	0.00810254	Arabidopsis thaliana strictosidine synthase 3 mRNA, complete cds
ATMG01220	-3.103815279	0.008407207	Arabidopsis thaliana ecotype Landsberg erecta mitochondrion, complete genome
AT3G17050	-3.103815279	0.008407207	Arabidopsis thaliana chromosome 3, complete sequence
AT4G23215	-1.457452233	0.008506487	Arabidopsis thaliana chromosome 4 sequence
AT3G45960	-1.007890859	0.009484211	Arabidopsis thaliana expansin-like A3 mRNA,

			complete cds
AT1G66860	-1.518852778	0.00952913	Arabidopsis thaliana class I glutamine amidotransferase-like domain-containing protein mRNA, complete cds
AT5G44690	-1.3124019	0.009785506	Arabidopsis thaliana uncharacterized protein mRNA, complete cds
AT1G51830	-1.59685529	0.010534949	Arabidopsis thaliana putative leucine-rich repeat protein kinase mRNA, complete cds
AT4G33467	-2.393321896	0.011059208	Arabidopsis thaliana uncharacterized protein mRNA, complete cds
AT1G61800	-2.393321896	0.011059208	Arabidopsis thaliana glucose-6-phosphate/phosphate translocator 2 mRNA, complete cds
AT1G33920	-2.393321896	0.011059208	Arabidopsis thaliana phloem protein 2-A4 mRNA, complete cds
AT3G45280	-2.393321896	0.011059208	Arabidopsis thaliana syntaxin-72 mRNA, complete cds
AT1G21360	-2.393321896	0.011059208	Arabidopsis thaliana glycolipid transfer protein 2 mRNA, complete cds
AT1G63930	-1.126535355	0.011067021	Arabidopsis thaliana chromosome 1 sequence
AT1G17030	-1.011892789	0.011190792	Arabidopsis thaliana uncharacterized protein mRNA, complete cds
AT2G42430	-1.348927776	0.011246261	Arabidopsis thaliana LOB domain-containing protein 16 mRNA, complete cds
AT1G24420	-1.348927776	0.011246261	Arabidopsis thaliana chromosome 1 sequence
AT3G48640	-1.348927776	0.011246261	Arabidopsis thaliana chromosome 3, complete sequence
AT5G01180	-1.699425023	0.01142448	Arabidopsis thaliana peptide transporter PTR5 mRNA, complete cds
AT4G39000	-1.699425023	0.01142448	Arabidopsis thaliana glycosyl hydrolase 9B17 mRNA, complete cds
AT1G01453	-1.699425023	0.01142448	Arabidopsis thaliana chromosome 1 sequence
AT1G68350	-1.699425023	0.01142448	Arabidopsis thaliana chromosome 1 sequence
AT3G04181	-1.699425023	0.01142448	Arabidopsis thaliana uncharacterized protein mRNA, complete cds
AT5G54790	-1.840780873	0.012026993	Arabidopsis thaliana uncharacterized protein mRNA, complete cds
AT2G36270	-1.840780873	0.012026993	Arabidopsis thaliana protein abscisic acid-insensitive 5 mRNA, complete cds
AT2G22950	-2.049367495	0.012057352	Arabidopsis thaliana putative calcium-transporting ATPase 7 mRNA, complete cds
AT3G57110	-3.741245199	0.012127161	Arabidopsis thaliana chromosome 3, complete sequence
AT2G17740	-3.741245199	0.012127161	Arabidopsis thaliana chromosome 2, complete sequence

AT1G78330	-3.741245199	0.012127161	Arabidopsis thaliana chromosome 1 sequence
AT1G56165	-3.741245199	0.012127161	Arabidopsis thaliana clone 126147 mRNA sequence
AT5G53700	-3.741245199	0.012127161	Arabidopsis thaliana RNA-binding (RRM/RBD/RNP motifs) family protein mRNA, complete cds
AT2G47040	-3.741245199	0.012127161	Arabidopsis thaliana pectinesterase 5 mRNA, complete cds
AT1G29830	-1.229346161	0.012360709	Arabidopsis thaliana Magnesium transporter CorA-like family protein mRNA, complete cds
AT1G06980	-1.393321896	0.012857364	Arabidopsis thaliana chromosome 1 sequence
AT1G59725	-1.393321896	0.012857364	Arabidopsis thaliana putative DNAJ heat shock protein mRNA, complete cds
AT5G23950	-1.393321896	0.012857364	Arabidopsis thaliana chromosome 5 sequence
AT4G22610	-1.393321896	0.012857364	Arabidopsis thaliana chromosome 4 sequence
AT3G01960	-1.255818372	0.014372334	Arabidopsis thaliana uncharacterized protein mRNA, complete cds
AT4G01670	-1.255818372	0.014372334	Arabidopsis thaliana uncharacterized protein mRNA, complete cds
AT1G67980	-1.44846345	0.014592156	Arabidopsis thaliana caffeoyl-CoA 3-O-methyltransferase mRNA, complete cds
AT3G58380	-2.933890277	0.015575638	Arabidopsis thaliana TRAF-like family protein mRNA, complete cds
AT2G37000	-2.933890277	0.015575638	Arabidopsis thaliana chromosome 2, complete sequence
AT2G17890	-2.933890277	0.015575638	Arabidopsis thaliana calcium-dependent protein kinase 16 mRNA, complete cds
AT5G57550	-2.933890277	0.015575638	Arabidopsis thaliana probable xyloglucan endotransglucosylase/hydrolase protein 25 mRNA, complete cds
AT3G56660	-2.933890277	0.015575638	Arabidopsis thaliana basic region/leucine zipper motif protein 49 mRNA, complete cds
AT1G07460	-2.933890277	0.015575638	Arabidopsis thaliana chromosome 1 sequence
AT4G31250	-2.933890277	0.015575638	Arabidopsis thaliana putative LRR receptor-like serine/threonine-protein kinase mRNA, complete cds
AT1G18835	-2.933890277	0.015575638	Arabidopsis thaliana chromosome 1 sequence
AT4G33985	-1.080731665	0.015605653	Arabidopsis thaliana uncharacterized protein mRNA, complete cds
AT5G26690	-1.080731665	0.015605653	Arabidopsis thaliana heavy-metal-associated domain-containing protein mRNA, complete cds
ATMG01080	-1.080731665	0.015605653	Arabidopsis thaliana ecotype Landsberg erecta mitochondrion, complete genome
AT3G14510	-1.518852778	0.016388146	Arabidopsis thaliana putative geranylgeranyl pyrophosphate synthase 8 mRNA, complete cds
AT5G11190	-1.518852778	0.016388146	Arabidopsis thaliana ethylene-responsive transcription factor SHINE 2 mRNA, complete cds

AT5G51930	-1.518852778	0.016388146	Arabidopsis thaliana Glucose-methanol-choline (GMC) oxidoreductase family protein mRNA, complete cds
AT5G38790	-1.518852778	0.016388146	Arabidopsis thaliana chromosome 5 sequence
AT2G03370	-1.518852778	0.016388146	Arabidopsis thaliana Glycosyltransferase family 61 protein mRNA, complete cds
AT2G43390	-1.518852778	0.016388146	Arabidopsis thaliana chromosome 2, complete sequence
AT3G56275	-1.518852778	0.016388146	Arabidopsis thaliana chromosome 3, complete sequence
AT5G11290	-1.174898377	0.017864034	Arabidopsis thaliana chromosome 5 sequence
AT2G31945	-1.174898377	0.017864034	Arabidopsis thaliana chromosome 2, complete sequence
AT1G63910	-1.611962182	0.018119365	Arabidopsis thaliana myb domain protein 103 mRNA, complete cds
AT3G04330	-1.611962182	0.018119365	Arabidopsis thaliana chromosome 3, complete sequence
AT4G11390	-1.611962182	0.018119365	Arabidopsis thaliana cysteine/histidine-rich C1 domain-containing protein mRNA, complete cds
AT3G49820	-1.611962182	0.018119365	Arabidopsis thaliana chromosome 3, complete sequence
AT1G19200	-1.091431554	0.018399025	Arabidopsis thaliana uncharacterized protein mRNA, complete cds
AT1G17285	-1.091431554	0.018399025	Arabidopsis thaliana uncharacterized protein mRNA, complete cds
AT1G58225	-1.091431554	0.018399025	Arabidopsis thaliana uncharacterized protein mRNA, complete cds
AT5G59510	-1.091431554	0.018399025	Arabidopsis thaliana chromosome 5 sequence
AT2G19050	-1.3262077	0.019258532	Arabidopsis thaliana GDSL esterase/lipase mRNA, complete cds
AT5G17860	-1.3262077	0.019258532	Arabidopsis thaliana chromosome 5 sequence
AT2G45580	-1.3262077	0.019258532	Arabidopsis thaliana cytochrome P450 76C3 mRNA, complete cds
AT1G07795	-2.255818372	0.019363621	Arabidopsis thaliana chromosome 1 sequence
AT5G10230	-2.255818372	0.019363621	Arabidopsis thaliana annexin D7 mRNA, complete cds
AT1G61120	-2.255818372	0.019363621	Arabidopsis thaliana terpene synthase 04 mRNA, complete cds
AT2G46480	-2.255818372	0.019363621	Arabidopsis thaliana probable galacturonosyltransferase 2 mRNA, complete cds
AT1G03580	-2.255818372	0.019363621	Arabidopsis thaliana chromosome 1 sequence
AT5G26630	-2.255818372	0.019363621	Arabidopsis thaliana chromosome 5 sequence
AT1G79680	-2.255818372	0.019363621	Arabidopsis thaliana wall-associated receptor kinase-like 10 mRNA, complete cds

AT1G01810	-1.741245199	0.019546899	Arabidopsis thaliana chromosome 1 sequence
AT3G63060	-1.741245199	0.019546899	Arabidopsis thaliana chromosome 3, complete sequence
AT3G44840	-1.741245199	0.019546899	Arabidopsis thaliana S-adenosyl-L-methionine-dependent methyltransferases superfamily protein mRNA, complete cds
AT5G51210	-1.933890277	0.020229256	Arabidopsis thaliana oleosin3 mRNA, complete cds
AT2G43470	-1.933890277	0.020229256	Arabidopsis thaliana uncharacterized protein mRNA, complete cds
AT3G48630	-1.933890277	0.020229256	Arabidopsis thaliana uncharacterized protein mRNA, complete cds
AT1G13145	-1.196924683	0.020924887	Arabidopsis thaliana chromosome 1 sequence
AT1G09480	-1.103815279	0.021697505	Arabidopsis thaliana alcohol dehydrogenase-like protein mRNA, complete cds
AT1G25054	-1.103815279	0.021697505	Arabidopsis thaliana UDP-3-O-[3-hydroxymyristoyl] N-acetylglucosamine deacetylase mRNA, complete cds
AT5G01480	-1.103815279	0.021697505	Arabidopsis thaliana chromosome 5 sequence
AT4G22630	-1.033425951	0.021831173	Arabidopsis thaliana bifunctional inhibitor/lipid-transfer protein/seed storage 2S albumin superfamily protein mRNA, complete cds
AT1G52530	-1.033425951	0.021831173	Arabidopsis thaliana Hus1 domain-containing protein mRNA, complete cds
AT5G53980	-1.374462869	0.022135212	Arabidopsis thaliana chromosome 5 sequence
AT3G47770	-1.374462869	0.022135212	Arabidopsis thaliana ABC transporter A family member 6 mRNA, complete cds
AT1G80470	-3.518852778	0.023200279	Arabidopsis thaliana F-box/FBD/LRR-repeat protein mRNA, complete cds
ATCG01090	-3.518852778	0.023200279	Arabidopsis thaliana chloroplast DNA, complete genome, ecotype: Columbia
AT5G35407	-3.518852778	0.023200279	Arabidopsis thaliana chromosome 5 sequence
AT3G62320	-3.518852778	0.023200279	Arabidopsis thaliana putative nucleic acid binding protein mRNA, complete cds
AT1G76230	-3.518852778	0.023200279	Arabidopsis thaliana chromosome 1 sequence
AT1G34490	-3.518852778	0.023200279	Arabidopsis thaliana chromosome 1 sequence
AT5G62040	-3.518852778	0.023200279	Arabidopsis thaliana protein BROTHER of FT and TFL 1 mRNA, complete cds
AT5G50360	-3.518852778	0.023200279	Arabidopsis thaliana chromosome 5 sequence
AT5G01080	-3.518852778	0.023200279	Arabidopsis thaliana chromosome 5 sequence
AT3G43850	-3.518852778	0.023200279	Arabidopsis thaliana uncharacterized protein mRNA, complete cds
AT5G20045	-3.518852778	0.023200279	Arabidopsis thaliana chromosome 5 sequence
AT4G34810	-1.223396894	0.024475655	Arabidopsis thaliana chromosome 4 sequence

AT3G15700	-1.223396894	0.024475655	Arabidopsis thaliana P-loop containing nucleoside triphosphate hydrolases superfamily protein mRNA, complete cds
AT5G54490	-1.223396894	0.024475655	Arabidopsis thaliana chromosome 5 sequence
AT2G32210	-1.436390618	0.025244545	Arabidopsis thaliana uncharacterized protein mRNA, complete cds
AT4G01930	-1.436390618	0.025244545	Arabidopsis thaliana chromosome 4 sequence
AT1G10790	-1.436390618	0.025244545	Arabidopsis thaliana uncharacterized protein mRNA, complete cds
AT5G41685	-1.436390618	0.025244545	Arabidopsis thaliana chromosome 5 sequence
AT4G26770	-1.436390618	0.025244545	Arabidopsis thaliana phosphatidate cytidylyltransferase mRNA, complete cds
AT4G20362	-1.040805481	0.025847187	Arabidopsis thaliana Full-length cDNA Complete sequence from clone GSLTPGH40ZC03 of Hormone Treated Callus of strain col-0 of Arabidopsis thaliana (thale cress)
AT5G47590	-1.040805481	0.025847187	Arabidopsis thaliana Heat shock protein HSP20/alpha crystallin family protein mRNA, complete cds
AT1G48220	-1.040805481	0.025847187	Arabidopsis thaliana protein kinase family prtein mRNA, complete cds
AT2G32020	-1.040805481	0.025847187	Arabidopsis thaliana chromosome 2, complete sequence
AT3G23230	-1.040805481	0.025847187	Arabidopsis thaliana chromosome 3, complete sequence
AT1G73965	-1.518852778	0.028449529	Arabidopsis thaliana chromosome 1 sequence
AT3G21351	-1.518852778	0.028449529	Arabidopsis thaliana uncharacterized protein mRNA, complete cds
AT1G70660	-1.518852778	0.028449529	Arabidopsis thaliana ubiquitin-conjugating enzyme E2 variant 1B mRNA, complete cds
AT3G09330	-1.255818372	0.028568016	Arabidopsis thaliana transmembrane amino acid transporter-like protein mRNA, complete cds
AT5G24040	-1.255818372	0.028568016	Arabidopsis thaliana uncharacterized protein mRNA, complete cds
AT2G01580	-1.255818372	0.028568016	Arabidopsis thaliana chromosome 2, complete sequence
AT3G51680	-1.255818372	0.028568016	Arabidopsis thaliana short-chain dehydrogenase reductase 2a mRNA, complete cds
AT5G35495	-2.741245199	0.028837806	Arabidopsis thaliana chromosome 5 sequence
AT1G11560	-2.741245199	0.028837806	Arabidopsis thaliana chromosome 1 sequence
AT2G04490	-2.741245199	0.028837806	Arabidopsis thaliana chromosome 2, complete sequence
AT4G26790	-2.741245199	0.028837806	Arabidopsis thaliana GDSL esterase/lipase mRNA, complete cds
AT3G11050	-1.135524138	0.030182724	Arabidopsis thaliana ferritin 2 mRNA, complete cds

AT5G43240	-1.049367495	0.030626511	Arabidopsis thaliana uncharacterized protein mRNA, complete cds
AT2G07808	-1.049367495	0.030626511	Arabidopsis thaliana chromosome 2, complete sequence
AT4G01895	-1.049367495	0.030626511	Arabidopsis thaliana chromosome 4 sequence
AT1G43590	-1.634329995	0.031449726	Arabidopsis thaliana chromosome 1 sequence
AT2G34330	-1.634329995	0.031449726	Arabidopsis thaliana chromosome 2, complete sequence
AT5G16330	-1.634329995	0.031449726	Arabidopsis thaliana NC domain-containing protein-like protein mRNA, complete cds
AT1G25422	-1.634329995	0.031449726	Arabidopsis thaliana chromosome 1 sequence
AT1G49832	-1.296460357	0.033236484	Arabidopsis thaliana chromosome 1 sequence
AT3G63360	-1.296460357	0.033236484	Arabidopsis thaliana defensin-like protein mRNA, complete cds
AT3G03776	-1.296460357	0.033236484	Arabidopsis thaliana hydroxyproline-rich glycoprotein family protein mRNA, complete cds
ATCG00420	-1.808359395	0.033618536	Arabidopsis thaliana chloroplast DNA, complete genome, ecotype: Columbia
AT5G07700	-1.808359395	0.033618536	Arabidopsis thaliana myb domain protein 76 mRNA, complete cds
AT5G41570	-1.808359395	0.033618536	Arabidopsis thaliana WRKY transcription factor 24 mRNA, complete cds
AT1G19371	-1.808359395	0.033618536	Arabidopsis thaliana chromosome 1 sequence
AT1G47620	-2.103815279	0.033654305	Arabidopsis thaliana chromosome 1 sequence
AT5G14110	-2.103815279	0.033654305	Arabidopsis thaliana uncharacterized protein mRNA, complete cds
AT3G16210	-2.103815279	0.033654305	Arabidopsis thaliana chromosome 3, complete sequence
AT2G37720	-2.103815279	0.033654305	Arabidopsis thaliana trichome birefringence-like 15 protein mRNA, complete cds
AT1G51770	-2.103815279	0.033654305	Arabidopsis thaliana core-2/I-branching beta-1,6-N-acetylglucosaminyltransferase family protein mRNA, complete cds
AT3G14185	-1.156282698	0.035589571	Arabidopsis thaliana Full-length cDNA Complete sequence from clone GSLTSIL56ZC04 of Silique of strain col-0 of Arabidopsis thaliana (thale cress)
AT1G61470	-1.156282698	0.035589571	Arabidopsis thaliana chromosome 1 sequence
AT1G16225	-1.156282698	0.035589571	Arabidopsis thaliana putative syntaxin-type t-SNARE protein mRNA, complete cds
AT1G77200	-1.059421159	0.036319869	Arabidopsis thaliana chromosome 1 sequence
AT5G01060	-1.059421159	0.036319869	Arabidopsis thaliana Protein kinase protein with tetratricopeptide repeat domain mRNA, complete cds
AT5G02580	-1.059421159	0.036319869	Arabidopsis thaliana uncharacterized protein mRNA,

			complete cds
AT3G06100	-1.348927776	0.038472987	Arabidopsis thaliana putative aquaporin NIP7-1 mRNA, complete cds
AT5G07060	-1.348927776	0.038472987	Arabidopsis thaliana MOS4-associated complex subunit 5C mRNA, complete cds
AT2G35612	-1.348927776	0.038472987	Arabidopsis thaliana chromosome 2, complete sequence
AT5G38770	-1.348927776	0.038472987	Arabidopsis thaliana chromosome 5 sequence
AT1G69430	-1.181817791	0.041938324	Arabidopsis thaliana chromosome 1 sequence
AT3G16900	-1.181817791	0.041938324	Arabidopsis thaliana uncharacterized protein mRNA, complete cds
AT2G22880	-1.071393801	0.043108412	Arabidopsis thaliana chromosome 2, complete sequence
AT1G61830	-1.071393801	0.043108412	Arabidopsis thaliana chromosome 1 sequence
AT3G14630	-1.071393801	0.043108412	Arabidopsis thaliana cytochrome P450, family 72, subfamily A, polypeptide 9 mRNA, complete cds
AT1G49370	-1.419317104	0.044174267	Arabidopsis thaliana chromosome 1 sequence
AT1G50390	-1.419317104	0.044174267	Arabidopsis thaliana pfkB-like carbohydrate kinase family protein mRNA, complete cds
AT1G78440	-1.419317104	0.044174267	Arabidopsis thaliana gibberellin 2-beta-dioxygenase 1 mRNA, complete cds
AT4G37050	-1.419317104	0.044174267	Arabidopsis thaliana PATATIN-like protein 4 mRNA, complete cds
ATMG00810	-3.255818372	0.044766751	Arabidopsis thaliana ecotype Landsberg erecta mitochondrion, complete genome
AT5G37620	-3.255818372	0.044766751	Arabidopsis thaliana chromosome 5 sequence
AT3G05780	-3.255818372	0.044766751	Arabidopsis thaliana lon protease 3 mRNA, complete cds
AT5G51105	-3.255818372	0.044766751	Arabidopsis thaliana chromosome 5 sequence
AT3G29970	-3.255818372	0.044766751	Arabidopsis thaliana B12D protein mRNA, complete cds
AT3G49300	-3.255818372	0.044766751	Arabidopsis thaliana proline-rich family protein mRNA, complete cds
AT2G21610	-3.255818372	0.044766751	Arabidopsis thaliana pectinesterase 11 mRNA, complete cds
AT3G11380	-3.255818372	0.044766751	Arabidopsis thaliana pentatricopeptide repeat-containing protein mRNA, complete cds
AT1G15050	-3.255818372	0.044766751	Arabidopsis thaliana auxin-responsive protein IAA34 mRNA, complete cds
AT2G21780	-3.255818372	0.044766751	Arabidopsis thaliana chromosome 2, complete sequence
AT4G15970	-3.255818372	0.044766751	Arabidopsis thaliana Nucleotide-diphospho-sugar transferase family protein mRNA, complete cds
AT4G26930	-3.255818372	0.044766751	Arabidopsis thaliana myb domain protein 97 mRNA,

			complete cds
AT1G36675	-1.213998196	0.049356736	Arabidopsis thaliana glycine-rich protein mRNA, complete cds
AT5G41590	-1.213998196	0.049356736	Arabidopsis thaliana uncharacterized protein mRNA, complete cds
AT4G22214	-1.213998196	0.049356736	Arabidopsis thaliana defensin-like protein 99 mRNA, complete cds
AT1G06923	-1.213998196	0.049356736	Arabidopsis thaliana uncharacterized protein mRNA, complete cds
AT3G10815	-1.213998196	0.049356736	Arabidopsis thaliana RING/U-box domain-containing protein mRNA, complete cds
AT2G18550	-1.518852778	0.050028779	Arabidopsis thaliana homeobox-leucine zipper protein ATHB-21 mRNA, complete cds
AT4G36230	-1.518852778	0.050028779	Arabidopsis thaliana chromosome 4 sequence
ATCG00400	-1.518852778	0.050028779	Oltmannsiellopsis viridis chloroplast, complete genome
AT3G29252	-1.518852778	0.050028779	Arabidopsis thaliana chromosome 3, complete sequence
AT5G39720	-1.518852778	0.050028779	Arabidopsis thaliana avirulence induced protein 2 like protein mRNA, complete cds
AT4G11370	-1.085893371	0.051209119	Arabidopsis thaliana chromosome 4 sequence
AT3G06490	-2.518852778	0.053304086	Arabidopsis thaliana putative transcription factor MYB108 mRNA, complete cds
AT2G29170	-2.518852778	0.053304086	Arabidopsis thaliana NAD(P)-binding Rossmann-fold superfamily protein mRNA, complete cds
AT2G25770	-2.518852778	0.053304086	Arabidopsis thaliana chromosome 2, complete sequence
AT3G45170	-2.518852778	0.053304086	Arabidopsis thaliana GATA transcription factor 14 mRNA, complete cds
AT2G01770	-2.518852778	0.053304086	Arabidopsis thaliana vacuolar iron transporter 1 mRNA, complete cds
AT5G21030	-2.518852778	0.053304086	Arabidopsis thaliana protein argonaute 8 mRNA, complete cds
AT4G31950	-2.518852778	0.053304086	Arabidopsis thaliana cytochrome P450, family 82, subfamily C, polypeptide 3 mRNA, complete cds
AT2G27220	-2.518852778	0.053304086	Arabidopsis thaliana BEL1-like homeodomain 5 mRNA, complete cds
AT2G42440	-2.518852778	0.053304086	Arabidopsis thaliana LOB domain-containing protein 17 mRNA, complete cds
AT4G11385	-2.518852778	0.053304086	Arabidopsis thaliana uncharacterized protein mRNA, complete cds
AT2G28670	-2.518852778	0.053304086	Arabidopsis thaliana protein ENHANCED SUBERIN 1 mRNA, complete cds
AT5G39260	-2.518852778	0.053304086	Arabidopsis thaliana expansin A21 mRNA, complete

			cds
AT4G33310	-2.518852778	0.053304086	Arabidopsis thaliana chromosome 4 sequence
AT1G62190	-2.518852778	0.053304086	Arabidopsis thaliana chromosome 1 sequence
AT4G15075	-2.518852778	0.053304086	Arabidopsis thaliana FBD-like domain family protein mRNA, complete cds
AT3G04410	-2.518852778	0.053304086	Arabidopsis thaliana no apical meristem-domain containing transcriptional regulator mRNA, complete cds
AT3G49150	-2.518852778	0.053304086	Arabidopsis thaliana putative F-box/LRR-repeat protein mRNA, complete cds
AT1G35255	-2.518852778	0.053304086	Arabidopsis thaliana chromosome 1 sequence
AT1G09380	-1.670855871	0.055257715	Arabidopsis thaliana nodulin MtN21-like transporter family protein mRNA, complete cds
AT5G59370	-1.670855871	0.055257715	Arabidopsis thaliana actin 4 mRNA, complete cds
AT2G23400	-1.670855871	0.055257715	Arabidopsis thaliana undecaprenyl pyrophosphate synthetase family protein mRNA, complete cds
AT2G35890	-1.255818372	0.057950392	Arabidopsis thaliana calcium-dependent protein kinase 25 mRNA, complete cds
AT5G10570	-1.255818372	0.057950392	Arabidopsis thaliana transcription factor bHLH61 mRNA, complete cds
AT4G03600	-1.255818372	0.057950392	Arabidopsis thaliana chromosome 4 sequence
AT3G59730	-1.255818372	0.057950392	Arabidopsis thaliana chromosome 3, complete sequence
AT1G69050	-1.255818372	0.057950392	Arabidopsis thaliana chromosome 1 sequence
AT3G21780	-1.255818372	0.057950392	Arabidopsis thaliana chromosome 3, complete sequence
AT3G50290	-1.255818372	0.057950392	Arabidopsis thaliana chromosome 3, complete sequence
AT4G29610	-1.255818372	0.057950392	Arabidopsis thaliana chromosome 4 sequence
AT1G34520	-1.255818372	0.057950392	Arabidopsis thaliana MBOAT (membrane bound O-acyl transferase) family protein mRNA, complete cds
AT4G19000	-1.933890277	0.057965237	Arabidopsis thaliana INTERACTS WITH SPT6-like protein IWS2 mRNA, complete cds
AT3G12835	-1.933890277	0.057965237	Arabidopsis thaliana chromosome 3, complete sequence
AT1G65670	-1.933890277	0.057965237	Arabidopsis thaliana cytochrome P450, family 702, subfamily A, polypeptide 1 mRNA, complete cds
AT1G67265	-1.933890277	0.057965237	Arabidopsis thaliana chromosome 1 sequence
AT1G25240	-1.933890277	0.057965237	Arabidopsis thaliana chromosome 1 sequence
AT1G27921	-1.933890277	0.057965237	Arabidopsis thaliana chromosome 1 sequence
AT4G10860	-1.933890277	0.057965237	Arabidopsis thaliana chromosome 4 sequence
AT4G03470	-1.103815279	0.060879843	Arabidopsis thaliana ankyrin repeat-containing

			protein mRNA, complete cds
AT3G01830	-1.103815279	0.060879843	Arabidopsis thaliana chromosome 3, complete sequence
AT3G49510	-1.103815279	0.060879843	Arabidopsis thaliana F-box protein mRNA, complete cds
AT5G45740	-1.103815279	0.060879843	Arabidopsis thaliana ubiquitin domain-containing protein mRNA, complete cds
AT4G30370	-1.103815279	0.060879843	Arabidopsis thaliana chromosome 4 sequence
AT5G14070	-1.103815279	0.060879843	Arabidopsis thaliana chromosome 5 sequence
AT1G34540	-1.103815279	0.060879843	Arabidopsis thaliana chromosome 1 sequence
AT5G23700	-1.004279605	0.060925936	Arabidopsis thaliana uncharacterized protein mRNA, complete cds
AT1G35515	-1.004279605	0.060925936	Arabidopsis thaliana R2R3-type MYB transcription factor mRNA, complete cds
AT3G53450	-1.004279605	0.060925936	Arabidopsis thaliana cytokinin riboside 5'-monophosphate phosphoribohydrolase LOG4 mRNA, complete cds
AT4G40065	-1.004279605	0.060925936	Arabidopsis thaliana chromosome 4 sequence
AT4G15280	-1.004279605	0.060925936	Arabidopsis thaliana chromosome 4 sequence

Up regulated genes in Col-0(+Cd)/Col-0(-Cd)

Gene	Log2FoldChange	pval	NT:Description
AT3G45140	4.109111133	0	Arabidopsis thaliana lipoxygenase 2 mRNA, complete cds
AT5G24770	3.911102977	0	Arabidopsis thaliana acid phosphatase VSP2 mRNA, complete cds
AT1G52400	2.635737858	0	Arabidopsis thaliana beta glucosidase 18 mRNA, complete cds
AT3G25770	4.764181795	0	Arabidopsis thaliana allene oxide cyclase 2 mRNA, complete cds
AT4G23600	5.612916744	0	Arabidopsis thaliana cystine lyase CORI3 mRNA, complete cds
AT1G13930	2.863239806	0	Arabidopsis thaliana salt tolerance-related protein mRNA, complete cds
AT5G24780	5.162955153	0	Arabidopsis thaliana acid phosphatase VSP1 mRNA, complete cds
AT3G26740	2.65866999	0	Arabidopsis thaliana CCR-like protein mRNA, complete cds
AT1G29910	1.243626797	0	Arabidopsis thaliana chromosome 1 sequence
AT1G20620	1.210279908	0	Arabidopsis thaliana catalase 3 mRNA, complete cds
AT3G22231	3.376385722	0	Arabidopsis thaliana protein PATHOGEN AND CIRCADIAN CONTROLLED 1 mRNA, complete cds

AT2G21660	2.12946939	0	Arabidopsis thaliana glycine-rich RNA-binding protein 7 mRNA, complete cds
AT4G18440	3.255483082	0	Arabidopsis thaliana L-aspartase-like family protein mRNA, complete cds
AT1G19670	2.793047568	0	Arabidopsis thaliana chlorophyllase 1 mRNA, complete cds
AT1G15520	5.946293682	0	Arabidopsis thaliana ABC transporter G family member 40 mRNA, complete cds
AT1G31580	1.368166541	0	Arabidopsis thaliana protein ECS1 mRNA, complete cds
AT3G46970	3.518492847	0	Arabidopsis thaliana alpha-glucan phosphorylase 2 mRNA, complete cds
AT1G10760	3.115427757	0	Arabidopsis thaliana alpha-glucan water dikinase 1 mRNA, complete cds
AT4G24190	1.716116259	0	Arabidopsis thaliana HSP90-like protein GRP94 mRNA, complete cds
AT4G08870	2.733323308	0	Arabidopsis thaliana arginine amidohydrolase 2 mRNA, complete cds
AT1G02930	2.969881287	0	Arabidopsis thaliana glutathione S-transferase F6 mRNA, complete cds
AT2G24850	5.821782516	0	Arabidopsis thaliana tyrosine aminotransferase 3 mRNA, complete cds
AT5G50950	3.540064026	0	Arabidopsis thaliana fumarate hydratase 2 mRNA, complete cds
AT2G14560	3.702119914	0	Arabidopsis thaliana protein LURP1 mRNA, complete cds
AT1G19570	1.831188667	0	Arabidopsis thaliana dehydroascorbate reductase mRNA, complete cds
AT2G34430	1.059035372	0	Arabidopsis thaliana chromosome 2, complete sequence
AT5G28540	1.675877901	0	Arabidopsis thaliana Luminal-binding protein 1 mRNA, complete cds
AT2G42540	5.808707361	0	Arabidopsis thaliana cold-regulated protein 15a mRNA, complete cds
AT1G59870	1.013450026	0	Arabidopsis thaliana ABC transporter G family member 36 mRNA, complete cds
AT4G23670	1.226901744	0	Arabidopsis thaliana polyketide cyclase/dehydrase and lipid transport superfamily protein mRNA, complete cds
AT4G14400	1.405694761	0	Arabidopsis thaliana protein ACCELERATED CELL DEATH 6 mRNA, complete cds
AT3G57260	5.019726142	0	Arabidopsis thaliana beta 1,3-glucanase 2 mRNA, complete cds
AT5G15970	3.867231555	0	Arabidopsis thaliana stress-induced protein KIN2 mRNA, complete cds
AT1G69830	4.779521263	0	Arabidopsis thaliana alpha-amylase-like 3 mRNA, complete cds
AT3G29320	2.999585092	0	Arabidopsis thaliana alpha-glucan phosphorylase 1

			mRNA, complete cds
AT5G40450	2.487583928	0	Arabidopsis thaliana uncharacterized protein mRNA, complete cds
AT5G51070	1.657452699	0	Arabidopsis thaliana chaperone protein ClpD mRNA, complete cds
AT5G42020	1.558626146	0	Arabidopsis thaliana Luminal-binding protein 2 mRNA, complete cds
AT4G28080	1.617342491	0	Arabidopsis thaliana tetratricopeptide repeat domain protein mRNA, complete cds
AT5G11670	1.19317774	0	Arabidopsis thaliana NADP-dependent malic enzyme 2 mRNA, complete cds
AT2G25510	1.2116039	0	Arabidopsis thaliana uncharacterized protein mRNA, complete cds
AT1G14250	3.740524898	0	Arabidopsis thaliana probable apyrase 5 mRNA, complete cds
AT4G02520	1.198841943	0	Arabidopsis thaliana glutathione S-transferase F2 mRNA, complete cds
AT1G01320	1.602458339	0	Arabidopsis thaliana tetratricopeptide repeat-containing protein mRNA, complete cds
AT1G02920	2.131032437	0	Arabidopsis thaliana glutathione S-transferase F7 mRNA, complete cds
AT5G53460	1.378476859	0	Arabidopsis thaliana glutamate synthase 1 [NADH] mRNA, complete cds
AT1G52410	3.202455752	0	Arabidopsis thaliana TSK-associating protein 1 mRNA, complete cds
AT5G42650	1.674303076	0	Arabidopsis thaliana chromosome 5 sequence
AT5G61790	1.370496523	0	Arabidopsis thaliana calnexin 1 mRNA, complete cds
AT4G37370	3.435419466	0	Arabidopsis thaliana cytochrome P450, family 81, subfamily D, polypeptide 8 mRNA, complete cds
AT5G02940	2.155536055	0	Arabidopsis thaliana uncharacterized protein mRNA, complete cds
AT4G34950	1.915829868	0	Arabidopsis thaliana major facilitator family protein mRNA, complete cds
AT4G34710	1.6329899	0	Arabidopsis thaliana chromosome 4 sequence
AT5G20230	2.120450762	0	Arabidopsis thaliana blue-copper-binding protein mRNA, complete cds
AT1G70320	2.259665903	0	Arabidopsis thaliana ubiquitin-protein ligase 2 mRNA, complete cds
AT1G75040	3.806652031	0	Arabidopsis thaliana pathogenesis-related protein 5 mRNA, complete cds
AT2G40840	2.582441689	0	Arabidopsis thaliana 4-alpha-glucanotransferase DPE2 mRNA, complete cds
AT3G22235	2.916583081	0	Arabidopsis thaliana uncharacterized protein mRNA, complete cds

AT1G12090	1.103507623	0	Arabidopsis thaliana chromosome 1 sequence
AT3G44860	4.594253136	0	Arabidopsis thaliana farnesoic acid carboxyl-O-methyltransferase mRNA, complete cds
AT2G15970	2.263575208	0	Arabidopsis thaliana cold regulated 413 plasma membrane 1 mRNA, complete cds
AT4G16660	1.900988052	0	Arabidopsis thaliana heat shock protein 70 mRNA, complete cds
AT5G53450	1.434514427	0	Arabidopsis thaliana OBP3-responsive protein 1 mRNA, complete cds
AT2G39330	3.123907898	0	Arabidopsis thaliana jacalin-related lectin 23 mRNA, complete cds
AT3G18290	1.503275646	0	Arabidopsis thaliana putative E3 ligase BRUTUS mRNA, complete cds
AT1G52040	3.369229003	0	Arabidopsis thaliana myrosinase-binding protein 1 mRNA, complete cds
AT4G01870	3.80400272	0	Arabidopsis thaliana chromosome 4 sequence
AT3G63160	1.085078654	0	Arabidopsis thaliana chromosome 3, complete sequence
AT1G16410	2.722974808	0	Arabidopsis thaliana dihomomethionine N-hydroxylase mRNA, complete cds
AT1G43670	1.774848451	0	Arabidopsis thaliana fructose-1,6-bisphosphatase mRNA, complete cds
AT5G35735	1.763812683	0	Arabidopsis thaliana putative auxin-responsive protein mRNA, complete cds
AT4G17470	4.814161791	0	Arabidopsis thaliana putative palmitoyl-protein thioesterase mRNA, complete cds
AT3G27690	1.212078993	0	Arabidopsis thaliana photosystem II light harvesting complex protein 2.3 mRNA, complete cds
AT1G21250	1.583378829	0	Arabidopsis thaliana wall-associated receptor kinase 1 mRNA, complete cds
AT3G02260	2.362365742	0	Arabidopsis thaliana auxin transport protein BIG mRNA, complete cds
AT2G38470	1.812379901	1.07E-302	Arabidopsis thaliana putative WRKY transcription factor 33 mRNA, complete cds
AT2G47000	2.111537631	5.32E-301	Arabidopsis thaliana auxin efflux transmembrane transporter MDR4 mRNA, complete cds
AT1G70820	2.372783833	4.67E-297	Arabidopsis thaliana phosphoglucomutase-like protein mRNA, complete cds
AT4G15210	6.858030899	1.27E-284	Arabidopsis thaliana beta-amylase 5 mRNA, complete cds
AT3G18080	1.407323028	5.41E-281	Arabidopsis thaliana beta-glucosidase 44 mRNA, complete cds
AT4G21990	2.125634775	9.85E-279	Arabidopsis thaliana 5'-adenylylsulfate reductase 3 mRNA, complete cds
AT4G30650	3.594690944	1.19E-277	Arabidopsis thaliana putative low temperature and salt responsive protein mRNA, complete cds

AT5G42530	1.154338853	2.56E-274	Arabidopsis thaliana uncharacterized protein mRNA, complete cds
AT5G52310	3.42150407	4.11E-274	Arabidopsis thaliana protein LOW-TEMPERATURE-INDUCED 78 mRNA, complete cds
AT1G05680	8.43275507	3.42E-270	Arabidopsis thaliana Uridine diphosphate glycosyltransferase 74E2 mRNA, complete cds
AT1G21750	1.413516053	2.44E-269	Arabidopsis thaliana protein disulfide isomerase-like 1-1 mRNA, complete cds
AT3G09390	1.563993139	3.91E-269	Arabidopsis thaliana metallothionein 2A mRNA, complete cds
AT2G26560	3.633477126	1.42E-268	Arabidopsis thaliana phospholipase A 2A mRNA, complete cds
AT1G55860	2.458932545	4.73E-263	Arabidopsis thaliana ubiquitin-protein ligase 1 mRNA, complete cds
AT3G26830	3.360317913	8.39E-261	Arabidopsis thaliana protein PHYTOALEXIN DEFICIENT 3 mRNA, complete cds
AT1G80070	1.685082915	2.68E-260	Arabidopsis thaliana putative splicing factor Prp8 mRNA, complete cds
AT2G42530	5.805972394	8.79E-258	Arabidopsis thaliana cold-regulated protein 15b mRNA, complete cds
AT2G28290	2.801281509	2.54E-256	Arabidopsis thaliana chromatin structure-remodeling complex protein SYD mRNA, complete cds
AT3G62700	1.972176726	2.42E-251	Arabidopsis thaliana ABC transporter C family member 14 mRNA, complete cds
AT4G26530	1.350775983	1.19E-250	Arabidopsis thaliana fructose-bisphosphate aldolase 5 mRNA, complete cds
AT3G08530	1.097264347	1.20E-246	Arabidopsis thaliana Clathrin, heavy chain mRNA, complete cds
AT3G13080	1.762807858	1.21E-245	Arabidopsis thaliana ABC transporter C family member 3 mRNA, complete cds
AT5G10760	2.870184872	1.12E-242	Arabidopsis thaliana aspartyl protease family protein mRNA, complete cds
AT2G16660	1.625049712	3.58E-231	Arabidopsis thaliana Major facilitator superfamily protein mRNA, complete cds
AT3G13330	2.038169944	4.60E-230	Arabidopsis thaliana proteasome activating protein 200 mRNA, complete cds
AT5G03350	3.055497813	1.11E-228	Arabidopsis thaliana chromosome 5 sequence
AT2G03760	2.549837916	7.26E-228	Arabidopsis thaliana chromosome 2, complete sequence
AT1G35710	1.379552388	3.29E-227	Arabidopsis thaliana putative leucine-rich repeat receptor-like protein kinase mRNA, complete cds
AT2G32240	1.493796199	6.16E-227	Arabidopsis thaliana uncharacterized protein mRNA, complete cds
AT1G06460	1.687940627	2.39E-224	Arabidopsis thaliana alpha-crystallin domain 32.1 mRNA, complete cds

AT5G11740	1.071902507	3.21E-224	Arabidopsis thaliana chromosome 5 sequence
AT4G04020	1.556799997	2.17E-222	Arabidopsis thaliana fibrillin mRNA, complete cds
AT1G68600	3.253735512	6.13E-221	Arabidopsis thaliana Aluminum activated malate transporter family protein mRNA, complete cds
AT1G29395	3.247640467	1.17E-219	Arabidopsis thaliana cold regulated 314 inner membrane 1 mRNA, complete cds
AT4G24350	1.802004068	3.51E-219	Arabidopsis thaliana phosphorylase family protein mRNA, complete cds
AT5G23820	1.407726139	3.19E-215	Arabidopsis thaliana MD-2-related lipid recognition domain-containing protein mRNA, complete cds
AT5G08790	2.243133015	1.02E-214	Arabidopsis thaliana protein ATAF2 mRNA, complete cds
AT5G41790	2.065145501	8.25E-208	Arabidopsis thaliana COP1-interactive protein 1 mRNA, complete cds
AT1G79280	2.20491068	2.67E-204	Arabidopsis thaliana nuclear pore anchor mRNA, complete cds
AT2G22125	1.518343846	1.45E-199	Arabidopsis thaliana cellulose synthase-interactive protein 1 mRNA, complete cds
AT1G49570	2.386709069	6.15E-199	Arabidopsis thaliana peroxidase mRNA, complete cds
AT3G23810	1.013678132	1.37E-197	Arabidopsis thaliana adenosylhomocysteinase 2 mRNA, complete cds
AT4G16890	1.378834941	9.42E-197	Arabidopsis thaliana protein SUPPRESSOR OF npr1-1, CONSTITUTIVE 1 mRNA, complete cds
AT4G16260	1.969619243	2.31E-196	Arabidopsis thaliana putative beta-1,3-endoglucanase mRNA, complete cds
AT1G77510	1.977379871	6.48E-193	Arabidopsis thaliana protein disulfide isomerase-like 1-2 mRNA, complete cds
AT1G45201	1.097068367	1.03E-191	Arabidopsis thaliana triacylglycerol lipase-like 1 mRNA, complete cds
AT1G48090	2.011742214	1.98E-190	Arabidopsis thaliana calcium-dependent lipid-binding family protein mRNA, complete cds
AT4G17140	2.142715918	5.58E-189	Arabidopsis thaliana pleckstrin homology (PH) domain-containing protein mRNA, complete cds
AT3G56710	3.062173577	5.66E-189	Arabidopsis thaliana chromosome 3, complete sequence
AT5G53420	2.981966015	2.15E-188	Arabidopsis thaliana CCT motif family protein mRNA, complete cds
AT3G16470	1.004177079	1.92E-187	Arabidopsis thaliana JA-responsive protein 1 mRNA, complete cds
AT5G59670	2.267213831	5.96E-184	Arabidopsis thaliana Leucine-rich repeat protein kinase family protein mRNA, complete cds
AT2G47180	2.925987405	8.33E-183	Arabidopsis thaliana galactinol synthase 1 mRNA, complete cds
AT4G35800	1.785757494	1.49E-182	Arabidopsis thaliana DNA-directed RNA polymerase II subunit RPB1 mRNA, complete cds
AT3G22370	2.1641372	4.41E-181	Arabidopsis thaliana alternative oxidase 1A mRNA,

			complete cds
AT3G15840	1.034238075	8.30E-181	Arabidopsis thaliana post-illumination chlorophyll fluorescence increase protein mRNA, complete cds
AT1G76680	1.58853003	4.58E-180	Arabidopsis thaliana 12-oxophytodienoate reductase 1 mRNA, complete cds
AT2G43530	3.044013101	1.66E-177	Arabidopsis thaliana defensin-like protein 194 mRNA, complete cds
AT1G02080	1.474290321	8.19E-177	Arabidopsis thaliana CCR4-NOT transcription complex subunit 1 domain protein mRNA, complete cds
AT4G17330	1.601662904	1.85E-175	Arabidopsis thaliana G2484-1 protein mRNA, complete cds
AT1G03080	2.156432097	2.35E-174	Arabidopsis thaliana kinase interacting (KIP1-like) protein mRNA, complete cds
AT5G02810	3.33258608	1.10E-173	Arabidopsis thaliana pseudo-response regulator 7 mRNA, complete cds
AT1G58602	1.004825615	1.25E-172	Arabidopsis thaliana protein RECOGNITION OF PERONOSPORA PARASITICA 7 mRNA, complete cds
AT3G14620	1.718806893	3.10E-172	Arabidopsis thaliana cytochrome P450, family 72, subfamily A, polypeptide 8 mRNA, complete cds
AT1G01560	4.326555666	3.73E-172	Arabidopsis thaliana mitogen-activated protein kinase 11 mRNA, complete cds
AT3G56400	2.058670049	4.34E-170	Arabidopsis thaliana WRKY transcription factor 70 mRNA, complete cds
AT3G18890	1.181613209	7.79E-170	Arabidopsis thaliana protein TIC 62 mRNA, complete cds
AT4G12290	2.365032928	3.55E-168	Arabidopsis thaliana copper amine oxidase family protein mRNA, complete cds
AT2G35050	1.234712352	1.43E-164	Arabidopsis thaliana protein kinase family protein mRNA, complete cds
AT2G28900	1.493780961	2.35E-162	Arabidopsis thaliana outer plastid envelope protein 16-1 mRNA, complete cds
AT4G09020	2.449879684	3.79E-162	Arabidopsis thaliana isoamylase 3 mRNA, complete cds
AT1G60730	2.386273134	7.09E-160	Arabidopsis thaliana probable aldo-keto reductase 5 mRNA, complete cds
AT5G26340	2.58604505	3.89E-159	Arabidopsis thaliana sugar transport protein 13 mRNA, complete cds
AT4G16870	2.704337581	2.68E-158	Arabidopsis thaliana chromosome 4 sequence
AT5G06870	2.080958669	1.48E-156	Arabidopsis thaliana polygalacturonase inhibitor 2 mRNA, complete cds
AT1G17170	5.029314269	5.32E-156	Arabidopsis thaliana glutathione S-transferase TAU 24 mRNA, complete cds
AT5G67480	2.458132044	6.51E-153	Arabidopsis thaliana BTB and TAZ domain protein 4 mRNA, complete cds
AT4G39980	1.309198303	6.58E-152	Arabidopsis thaliana 3-deoxy-D-arabino-heptulosonate 7-phosphate synthase 1 mRNA, complete cds

AT3G12145	1.944097016	2.46E-151	Arabidopsis thaliana leucine-rich repeat protein FLOR1 mRNA, complete cds
AT5G02490	1.370272824	6.84E-151	Arabidopsis thaliana heat shock protein 70 mRNA, complete cds
AT3G22380	1.029687786	5.58E-150	Arabidopsis thaliana clock regulator protein TIME FOR COFFEE mRNA, complete cds
AT3G49620	3.978421149	1.18E-148	Arabidopsis thaliana 2-oxoacid-dependent dioxygenase-like protein DIN11 mRNA, complete cds
AT5G59540	1.781689811	2.45E-148	Arabidopsis thaliana oxidoreductase, 2OG-Fe(II) oxygenase family protein mRNA, complete cds
AT5G04560	2.13686741	3.06E-148	Arabidopsis thaliana transcriptional activator DEMETER mRNA, complete cds
AT2G17930	1.909286133	3.55E-147	Arabidopsis thaliana phosphatidylinositol 3- and 4-kinase family protein with FAT domain mRNA, complete cds
AT3G50930	2.009657209	3.76E-147	Arabidopsis thaliana chromosome 3, complete sequence
AT1G32350	4.438106716	3.12E-146	Arabidopsis thaliana alternative oxidase 1D mRNA, complete cds
AT3G52180	2.028893826	1.90E-145	Arabidopsis thaliana phosphoglucan phosphatase DSP4 mRNA, complete cds
AT4G16950	1.538389505	2.16E-142	Arabidopsis thaliana TIR-NBS-LRR class disease resistance protein mRNA, complete cds
AT2G06050	1.585332178	1.28E-139	Arabidopsis thaliana AT2G06050 mRNA, complete cds, clone: RAFL22-42-B17
AT2G34810	2.109520998	5.88E-139	Arabidopsis thaliana chromosome 2, complete sequence
AT3G25760	2.369362614	1.30E-138	Arabidopsis thaliana allene oxide cyclase 1 mRNA, complete cds
AT3G06500	1.784972713	1.53E-138	Arabidopsis thaliana protein alkaline/neutral invertase C mRNA, complete cds
AT5G26570	1.180511694	2.25E-138	Arabidopsis thaliana phosphoglucan, water dikinase mRNA, complete cds
AT1G20510	1.469666928	1.08E-137	Arabidopsis thaliana OPC-8:0 CoA ligase1 mRNA, complete cds
AT1G36370	1.605051503	1.20E-137	Arabidopsis thaliana putative serine hydroxymethyltransferase mRNA, complete cds
AT5G44800	2.088718067	7.56E-135	Arabidopsis thaliana chromatin remodeling 4 protein mRNA, complete cds
AT1G67120	3.019813975	9.16E-134	Arabidopsis thaliana protein MIDASIN1 mRNA, complete cds
AT1G64790	1.512256062	1.68E-133	Arabidopsis thaliana protein ILITHYIA mRNA, complete cds
AT4G23210	4.443793341	7.24E-133	Arabidopsis thaliana cysteine-rich receptor-like protein kinase 13 mRNA, complete cds
AT1G31690	1.868214108	2.89E-132	Arabidopsis thaliana copper amine oxidase family protein mRNA, complete cds

AT2G29460	4.27111668	3.87E-132	Arabidopsis thaliana glutathione S-transferase tau 4 mRNA, complete cds
AT1G56300	4.059900945	8.90E-132	Arabidopsis thaliana chaperone DnaJ-domain containing protein mRNA, complete cds
AT1G72180	1.828540375	1.75E-131	Arabidopsis thaliana leucine-rich receptor-like protein kinase mRNA, complete cds
AT2G26900	1.015872821	4.76E-131	Arabidopsis thaliana sodium/pyruvate cotransporter BASS2 mRNA, complete cds
AT3G60240	1.462886601	1.02E-130	Arabidopsis thaliana eukaryotic translation initiation factor 4G mRNA, complete cds
AT1G03060	1.909626369	2.17E-130	Arabidopsis thaliana WD/BEACH domain protein SPIRRIG mRNA, complete cds
AT2G36390	1.456190947	2.00E-129	Arabidopsis thaliana 1,4-alpha-glucan branching enzyme 2-1 mRNA, complete cds
AT4G23140	2.544847851	3.33E-128	Arabidopsis thaliana cysteine-rich receptor-like protein kinase 6 mRNA, complete cds
AT1G65010	1.764104019	2.02E-127	Arabidopsis thaliana flower development-related protein mRNA, complete cds
AT4G18240	1.416608512	9.00E-127	Arabidopsis thaliana starch synthase 4 mRNA, complete cds
AT1G32940	2.499638478	2.17E-126	Arabidopsis thaliana Subtilase 3.5 mRNA, complete cds
AT5G23110	2.059129491	7.68E-126	Arabidopsis thaliana uncharacterized protein mRNA, complete cds
AT2G43550	2.438987024	1.08E-125	Arabidopsis thaliana defensin-like protein 197 mRNA, complete cds
AT5G43900	1.44438962	1.24E-125	Arabidopsis thaliana myosin 2 mRNA, complete cds
AT2G35630	1.427851798	1.60E-125	Arabidopsis thaliana protein MICROTUBULE ORGANIZATION 1 mRNA, complete cds
AT4G16860	1.605739524	1.80E-125	Arabidopsis thaliana protein RECOGNITION OF PERONOSPORA PARASITICA 4 mRNA, complete cds
AT2G46020	1.656645742	3.95E-125	Arabidopsis thaliana ATP-dependent helicase BRAHMA mRNA, complete cds
AT4G21380	4.459943081	4.32E-125	Arabidopsis thaliana receptor kinase 3 mRNA, complete cds
AT3G51450	2.717418393	5.54E-125	Arabidopsis thaliana strictosidine synthase family protein mRNA, complete cds
AT1G14880	2.398677983	1.13E-124	Arabidopsis thaliana cadmium resistance protein 1 mRNA, complete cds
AT1G07050	4.142509556	9.67E-123	Arabidopsis thaliana CCT motif family protein mRNA, complete cds
AT1G52000	1.65928067	1.18E-122	Arabidopsis thaliana mannose-binding lectin superfamily protein mRNA, complete cds
AT3G08590	1.101803383	1.19E-122	Arabidopsis thaliana phosphoglycerate mutase, 2,3-bisphosphoglycerate-independent mRNA, complete

			cds
AT1G66980	1.60233379	1.73E-120	Arabidopsis thaliana suppressor of npr1-1 constitutive 4 mRNA, complete cds
AT3G11010	1.66610056	2.01E-120	Arabidopsis thaliana receptor like protein 34 mRNA, complete cds
AT3G20810	2.01144683	2.70E-120	Arabidopsis thaliana jumonji-C domain-containing protein 30 mRNA, complete cds
AT2G25730	1.497194358	2.77E-120	Arabidopsis thaliana uncharacterized protein mRNA, complete cds
AT4G15180	2.114810489	4.63E-120	Arabidopsis thaliana putative histone-lysine N-methyltransferase ATXR3 mRNA, complete cds
AT4G16590	7.05490813	1.81E-118	Arabidopsis thaliana cellulose synthase-like A01 mRNA, complete cds
AT2G43570	2.86734636	4.65E-118	Arabidopsis thaliana putative chitinase mRNA, complete cds
AT1G52030	3.115252423	5.97E-118	Arabidopsis thaliana myrosinase-binding protein 2 mRNA, complete cds
AT3G01310	1.539391761	8.12E-118	Arabidopsis thaliana phosphoglycerate mutase-like protein mRNA, complete cds
AT1G69870	1.72774301	6.15E-117	Arabidopsis thaliana nitrate transporter 1.7 mRNA, complete cds
AT4G12320	1.360434505	1.17E-116	Arabidopsis thaliana cytochrome P450, family 706, subfamily A, polypeptide 6 mRNA, complete cds
AT1G22770	3.737945388	6.27E-116	Arabidopsis thaliana protein GIGANTEA mRNA, complete cds
AT5G54960	1.641221995	1.92E-115	Arabidopsis thaliana chromosome 5 sequence
AT4G28710	1.741938349	4.64E-114	Arabidopsis thaliana myosin XI H mRNA, complete cds
AT5G43450	2.330790172	2.82E-113	Arabidopsis thaliana 1-aminocyclopropane-1-carboxylate oxidase-like protein mRNA, complete cds
AT2G04050	5.590861729	4.31E-112	Arabidopsis thaliana MATE efflux family protein mRNA, complete cds
AT3G28220	1.699886592	1.16E-111	Arabidopsis thaliana TRAF-like family protein mRNA, complete cds
AT5G25250	1.83985606	3.53E-111	Arabidopsis thaliana Flotillin-like protein 1 mRNA, complete cds
AT1G20960	1.438485921	4.62E-111	Arabidopsis thaliana putative U5 small nuclear ribonucleoprotein helicase mRNA, complete cds
AT5G47690	1.114184935	5.53E-111	Arabidopsis thaliana uncharacterized binding protein mRNA, complete cds
AT3G62550	2.397443025	1.97E-110	Arabidopsis thaliana drought responsive ATP-binding motif containing protein mRNA, complete cds
AT1G36160	1.160207731	1.02E-109	Arabidopsis thaliana acetyl-CoA carboxylase 1 mRNA, complete cds
AT1G76790	1.746989144	6.00E-109	Arabidopsis thaliana indole glucosinolate

			o-methyltransferase 5 mRNA, complete cds
AT2G45540	1.224894877	3.85E-108	Arabidopsis thaliana beige-related and WD-40 repeat-containing protein mRNA, complete cds
AT5G59130	2.712149388	4.41E-107	Arabidopsis thaliana Subtilase family protein mRNA, complete cds
AT1G78680	1.147927287	8.43E-107	Arabidopsis thaliana gamma-glutamyl hydrolase 2 mRNA, complete cds
AT1G53310	1.05323149	6.07E-106	Arabidopsis thaliana phosphoenolpyruvate carboxylase 1 mRNA, complete cds
AT4G01080	2.652426631	8.15E-106	Arabidopsis thaliana protein TRICHOME BIREFRINGENCE-LIKE 26 mRNA, complete cds
AT4G14760	2.3337141	1.34E-105	Arabidopsis thaliana kinase interacting-like protein mRNA, complete cds
AT1G72450	1.267696235	2.30E-105	Arabidopsis thaliana jasmonate-zim-domain protein 6 mRNA, complete cds
AT2G40080	2.995534423	7.56E-105	Arabidopsis thaliana chromosome 2, complete sequence
AT4G14365	1.127559215	9.77E-104	Arabidopsis thaliana putative E3 ubiquitin-protein ligase XBAT34 mRNA, complete cds
AT4G31570	2.312309416	1.47E-103	Arabidopsis thaliana AUCSIA-1 interacting protein mRNA, complete cds
AT5G06600	1.049744796	1.93E-103	Arabidopsis thaliana ubiquitin carboxyl-terminal hydrolase 12 mRNA, complete cds
AT2G43010	2.291153638	2.44E-103	Arabidopsis thaliana transcription factor PIF4 mRNA, complete cds
AT1G22400	1.905923962	5.82E-103	Arabidopsis thaliana UDP-glycosyltransferase 85A1 mRNA, complete cds
AT2G26440	2.471572329	1.02E-102	Arabidopsis thaliana Probable pectinesterase/pectinesterase inhibitor 12 mRNA, complete cds
AT2G36800	2.099477164	1.87E-102	Arabidopsis thaliana chromosome 2, complete sequence
AT4G21910	1.329300176	6.64E-102	Arabidopsis thaliana MATE efflux family protein mRNA, complete cds
AT5G35210	1.074935876	4.16E-100	Arabidopsis thaliana PHD type transcription factor with transmembrane domain protein mRNA, complete cds
AT1G71220	1.175166616	1.04E-99	Arabidopsis thaliana UDP-glucose:glycoprotein glucosyltransferase mRNA, complete cds
AT1G24460	1.833171317	1.65E-99	Arabidopsis thaliana TGN-localized SYP41 interacting protein mRNA, complete cds
AT3G52140	1.115678129	5.84E-99	Arabidopsis thaliana tetratricopeptide repeat (TPR)-containing protein mRNA, complete cds
AT1G80840	1.875948393	1.87E-98	Arabidopsis thaliana putative WRKY transcription factor 40 mRNA, complete cds
AT3G04550	1.037737786	6.72E-98	Arabidopsis thaliana chromosome 3, complete sequence
AT5G60100	3.29072356	1.62E-96	Arabidopsis thaliana pseudo-response regulator 3 mRNA,

			complete cds
AT2G39030	4.664114413	1.83E-96	Arabidopsis thaliana chromosome 2, complete sequence
AT3G26290	3.75236669	2.14E-96	Arabidopsis thaliana cytochrome P450 71B26 mRNA, complete cds
AT5G43440	3.250530804	2.98E-96	Arabidopsis thaliana 1-aminocyclopropane-1-carboxylate oxidase-like protein 9 mRNA, complete cds
AT3G11964	1.703625315	1.55E-95	Arabidopsis thaliana protein ribosomal RNA processing 5 mRNA, complete cds
AT3G02570	1.071807705	2.26E-95	Arabidopsis thaliana mannose-6-phosphate isomerase mRNA, complete cds
AT1G50030	1.439527825	5.66E-95	Arabidopsis thaliana serine/threonine-protein kinase TOR mRNA, complete cds
AT4G04490	3.829749777	1.39E-94	Arabidopsis thaliana cysteine-rich receptor-like protein kinase 36 mRNA, complete cds
AT4G19120	1.473096068	1.91E-94	Arabidopsis thaliana putative methyltransferase PMT21 mRNA, complete cds
AT3G14270	1.59887315	2.58E-94	Arabidopsis thaliana phosphatidylinositol-3P 5-kinase-like mRNA, complete cds
AT2G23010	3.580438374	3.23E-94	Arabidopsis thaliana serine carboxypeptidase-like 9 mRNA, complete cds
AT2G47800	1.214148004	3.73E-94	Arabidopsis thaliana ABC transporter C family member 4 mRNA, complete cds
AT4G36520	1.954711714	5.69E-94	Arabidopsis thaliana chaperone DnaJ-domain containing protein mRNA, complete cds
AT1G22060	1.970764119	2.80E-93	Arabidopsis thaliana uncharacterized protein mRNA, complete cds
AT3G50370	1.119493143	1.94E-92	Arabidopsis thaliana uncharacterized protein mRNA, complete cds
AT1G80020	1.661874599	3.22E-92	Arabidopsis thaliana chromosome 1 sequence
AT2G21380	1.702261095	2.32E-91	Arabidopsis thaliana kinesin motor family protein mRNA, complete cds
AT5G13200	2.05571647	9.87E-91	Arabidopsis thaliana GRAM domain family protein mRNA, complete cds
AT1G15780	1.82683505	1.21E-90	Arabidopsis thaliana uncharacterized protein mRNA, complete cds
AT1G77120	1.790109243	5.71E-90	Arabidopsis thaliana alcohol dehydrogenase 1 mRNA, complete cds
AT1G66880	1.137253003	7.19E-90	Arabidopsis thaliana serine/threonine protein kinase mRNA, complete cds
AT3G49110	1.524205233	2.42E-89	Arabidopsis thaliana peroxidase 33 mRNA, complete cds
AT2G26890	1.373755847	2.66E-88	Arabidopsis thaliana gravitropism defective 2 mRNA, complete cds
AT3G25010	3.010514011	3.91E-88	Arabidopsis thaliana chromosome 3, complete sequence
AT1G67230	1.225875873	4.83E-88	Arabidopsis thaliana protein little nuclei1 mRNA,

			complete cds
AT3G57660	1.964250812	7.93E-88	Arabidopsis thaliana nuclear RNA polymerase A1 mRNA, complete cds
AT1G13210	1.184933082	1.06E-87	Arabidopsis thaliana autoinhibited Ca2+/ATPase II mRNA, complete cds
AT1G79000	1.525208853	2.77E-87	Arabidopsis thaliana histone acetyltransferase HAC1 mRNA, complete cds
AT5G51440	2.202407685	5.53E-87	Arabidopsis thaliana mRNA for mitochondrial heat shock 22 kd protein-like, complete cds, clone: RAFL21-16-A12
AT1G72520	1.859804085	1.44E-86	Arabidopsis thaliana lipoxygenase 4 mRNA, complete cds
AT1G61120	5.207256738	1.35E-85	Arabidopsis thaliana terpene synthase 04 mRNA, complete cds
AT1G19960	3.240364482	1.68E-85	Arabidopsis thaliana chromosome 1 sequence
AT1G31550	2.415247263	2.26E-85	Arabidopsis thaliana GDSL esterase/lipase mRNA, complete cds
AT5G10470	1.121706017	3.13E-85	Arabidopsis thaliana kinesin CDKA;1 associated 1 mRNA, complete cds
AT1G26150	1.143821316	2.25E-84	Arabidopsis thaliana proline-rich extensin-like receptor kinase 10 mRNA, complete cds
AT5G47240	2.301897427	2.32E-84	Arabidopsis thaliana nudix hydrolase 8 mRNA, complete cds
AT1G35115	2.549487929	3.65E-84	Arabidopsis thaliana chromosome 1 sequence
AT3G25250	3.761401647	6.37E-84	Arabidopsis thaliana AGC (cAMP-dependent, cGMP-dependent and protein kinase C) kinase family protein mRNA, complete cds
AT3G43300	1.037189185	7.55E-84	Arabidopsis thaliana brefeldin A-inhibited guanine nucleotide-exchange protein 5 mRNA, complete cds
AT5G16970	1.343477898	8.08E-84	Arabidopsis thaliana 2-alkenal reductase mRNA, complete cds
AT3G05500	1.287165684	2.88E-83	Arabidopsis thaliana Rubber elongation factor protein mRNA, complete cds
AT2G29670	1.457630951	6.57E-83	Arabidopsis thaliana tetratricopeptide repeat-containing protein mRNA, complete cds
AT1G68050	5.469945629	7.95E-83	Arabidopsis thaliana flavin-binding, kelch repeat, f box 1 mRNA, complete cds
AT3G56200	1.727534837	1.03E-82	Arabidopsis thaliana putative amino acid transporter mRNA, complete cds
AT3G54670	1.731290987	2.45E-82	Arabidopsis thaliana structural maintenance of chromosomes 1 mRNA, complete cds
AT4G33070	3.897407873	2.67E-82	Arabidopsis thaliana pyruvate decarboxylase 1 mRNA, complete cds
AT1G20970	1.409351484	3.96E-82	Arabidopsis thaliana uncharacterized protein mRNA, complete cds
AT4G27560	1.755202823	8.19E-82	Arabidopsis thaliana chromosome 4 sequence

AT4G15490	2.235988456	2.95E-81	Arabidopsis thaliana chromosome 4 sequence
AT5G48880	2.250470733	5.22E-81	Arabidopsis thaliana 3-keto-acyl-CoA thiolase 2 mRNA, complete cds
AT1G65440	1.417329431	1.98E-80	Arabidopsis thaliana transcription elongation factor SPT6-like protein mRNA, complete cds
AT5G55660	1.28473549	2.10E-80	Arabidopsis thaliana DEK domain-containing chromatin associated protein mRNA, complete cds
AT1G19180	1.367511314	7.40E-80	Arabidopsis thaliana chromosome 1 sequence
AT5G64120	1.710463268	7.54E-80	Arabidopsis thaliana peroxidase 71 mRNA, complete cds
AT5G26220	4.346768887	8.29E-80	Arabidopsis thaliana ChaC-like family protein mRNA, complete cds
AT2G20960	1.230475338	9.31E-80	Arabidopsis thaliana phospholipase-like protein (PEARLI 4) domain-containing protein mRNA, complete cds
AT3G60860	1.462941017	2.56E-79	Arabidopsis thaliana SEC7-like guanine nucleotide exchange family protein mRNA, complete cds
AT4G36080	2.357202658	3.47E-79	Arabidopsis thaliana phosphotransferases/inositol or phosphatidylinositol kinases mRNA, complete cds
AT3G25610	1.669631418	3.62E-79	Arabidopsis thaliana ATPase E1-E2 type family protein / haloacid dehalogenase-like hydrolase family protein mRNA, complete cds
AT1G24706	1.695533167	3.77E-79	Arabidopsis thaliana THO complex subunit 2 mRNA, complete cds
AT2G18690	1.238356611	8.06E-79	Arabidopsis thaliana chromosome 2, complete sequence
AT2G31960	1.08216016	1.73E-78	Arabidopsis thaliana glucan synthase-like 3 mRNA, complete cds
AT4G16146	3.682791812	2.04E-78	Arabidopsis thaliana cAMP-regulated phosphoprotein 19-related protein mRNA, complete cds
AT3G04290	1.117003591	1.12E-77	Arabidopsis thaliana Li-tolerant lipase 1 mRNA, complete cds
AT5G41740	1.62105773	2.88E-77	Arabidopsis thaliana TIR-NBS-LRR class disease resistance protein mRNA, complete cds
AT5G45820	4.926017737	1.23E-76	Arabidopsis thaliana chromosome 5 sequence
AT1G21580	1.240098794	1.46E-76	Arabidopsis thaliana zinc finger CCCH domain-containing protein mRNA, complete cds
AT4G34390	1.412028627	6.71E-76	Arabidopsis thaliana extra-large GTP-binding protein 2 mRNA, complete cds
AT1G65800	1.218022306	8.61E-76	Arabidopsis thaliana receptor-like serine/threonine-protein kinase SD1-6 mRNA, complete cds
AT2G36490	1.536222161	8.68E-76	Arabidopsis thaliana protein ROS1 mRNA, complete cds
AT5G20490	1.100248195	1.03E-75	Arabidopsis thaliana Myosin XI K mRNA, complete cds
AT5G02310	1.251333185	2.85E-75	Arabidopsis thaliana proteolysis 6 mRNA, complete cds
AT2G30750	3.234633177	2.95E-75	Arabidopsis thaliana cytochrome P450 71A12 mRNA, complete cds

AT2G04070	4.204636038	3.80E-75	Arabidopsis thaliana MATE efflux family protein mRNA, complete cds
AT4G27430	1.152360227	8.52E-75	Arabidopsis thaliana COP1-interacting protein 7 mRNA, complete cds
AT5G64860	1.60920955	1.03E-74	Arabidopsis thaliana 4-alpha-glucanotransferase DPE1 mRNA, complete cds
AT2G41240	2.325131805	2.02E-74	Arabidopsis thaliana transcription factor bHLH100 mRNA, complete cds
AT3G50380	1.470054427	2.03E-74	Arabidopsis thaliana uncharacterized protein mRNA, complete cds
AT4G04840	2.121402176	2.31E-74	Arabidopsis thaliana methionine sulfoxide reductase B6 mRNA, complete cds
AT4G23260	1.267630784	3.34E-74	Arabidopsis thaliana cysteine-rich receptor-like protein kinase 18 mRNA, complete cds
AT2G13370	1.413454035	4.25E-74	Arabidopsis thaliana chromatin remodeling 5 mRNA, complete cds
AT1G03740	1.307462264	5.16E-74	Arabidopsis thaliana protein kinase mRNA, complete cds
AT1G21630	1.183468005	2.12E-73	Arabidopsis thaliana calcium-binding EF-hand-containing protein mRNA, complete cds
AT4G32340	1.987718436	5.02E-73	Arabidopsis thaliana tetratricopeptide repeat domain-containing protein-like protein mRNA, complete cds
AT5G24300	1.039201581	5.62E-73	Arabidopsis thaliana starch synthase 1 mRNA, complete cds
AT3G06530	1.598738855	1.32E-72	Arabidopsis thaliana U3snoRNP10 and NUC211 domain-containing protein mRNA, complete cds
AT1G77300	1.79099835	6.73E-72	Arabidopsis thaliana histone-lysine N-methyltransferase ASHH2 mRNA, complete cds
AT4G03400	2.181853248	2.03E-71	Arabidopsis thaliana auxin-responsive GH3 family protein mRNA, complete cds
AT5G41140	1.854615636	2.52E-71	Arabidopsis thaliana Myosin heavy chain-related protein mRNA, complete cds
AT3G01320	1.814038616	3.75E-71	Arabidopsis thaliana paired amphipathic helix protein Sin3-like 1 mRNA, complete cds
AT2G15490	5.640830189	2.22E-70	Arabidopsis thaliana mRNA for putative glucosyltransferase, complete cds, clone: RAFL14-26-J02
AT2G18193	3.011510462	6.61E-70	Arabidopsis thaliana P-loop containing nucleoside triphosphate hydrolases superfamily protein mRNA, complete cds
AT1G28420	1.731043246	1.50E-69	Arabidopsis thaliana homeobox-1 mRNA, complete cds
AT3G62750	1.160296982	7.55E-69	Arabidopsis thaliana beta glucosidase 8 mRNA, complete cds
AT2G39800	1.123558843	1.04E-68	Arabidopsis thaliana delta1-pyrroline-5-carboxylate synthase 1 mRNA, complete cds

AT1G17380	2.524162727	1.23E-68	Arabidopsis thaliana protein TIFY 11A mRNA, complete cds
AT2G34660	1.016580459	2.06E-68	Arabidopsis thaliana ABC transporter C family member 2 mRNA, complete cds
AT1G70060	1.680457846	2.14E-68	Arabidopsis thaliana paired amphipathic helix protein Sin3-like 4 mRNA, complete cds
AT5G42380	3.244093506	1.06E-67	Arabidopsis thaliana chromosome 5 sequence
AT1G13340	2.74920635	1.39E-67	Arabidopsis thaliana Regulator of Vps4 activity in the MVB pathway protein mRNA, complete cds
AT3G28210	3.8480816	1.57E-67	Arabidopsis thaliana zinc finger (AN1-like) family protein mRNA, complete cds
AT2G02010	3.585812346	1.64E-67	Arabidopsis thaliana glutamate decarboxylase 4 mRNA, complete cds
AT4G19530	1.144960672	1.95E-67	Arabidopsis thaliana TIR-NBS-LRR class disease resistance protein mRNA, complete cds
AT4G11380	1.025206708	3.70E-67	Arabidopsis thaliana beta-adaptin-like protein B mRNA, complete cds
AT4G03550	1.303893008	7.02E-67	Arabidopsis thaliana callose synthase 12 mRNA, complete cds
AT2G25110	1.018016094	9.28E-67	Arabidopsis thaliana stromal cell-derived factor 2-like protein mRNA, complete cds
AT4G39420	1.583536518	1.04E-66	Arabidopsis thaliana uncharacterized protein mRNA, complete cds
AT3G53800	2.205352549	1.32E-66	Arabidopsis thaliana hsp70-interacting protein FES1B-like protein mRNA, complete cds
AT5G24740	1.478756728	3.16E-66	Arabidopsis thaliana uncharacterized protein mRNA, complete cds
AT1G24147	1.975917922	5.40E-66	Arabidopsis thaliana uncharacterized protein mRNA, complete cds
AT5G50160	1.145493415	1.00E-65	Arabidopsis thaliana ferric reduction oxidase 8 mRNA, complete cds
AT1G58250	1.473997972	1.04E-65	Arabidopsis thaliana protein SABRE mRNA, complete cds
AT1G32750	1.586670327	1.74E-65	Arabidopsis thaliana histone acetyltransferase of the CBP family 13 mRNA, complete cds
AT2G40750	1.967978455	6.47E-65	Arabidopsis thaliana WRKY DNA-binding protein 54 mRNA, complete cds
AT2G15480	2.045599352	4.55E-64	Arabidopsis thaliana UDP-glucosyl transferase 73B5 mRNA, complete cds
AT2G30550	1.2225186	6.35E-64	Arabidopsis thaliana phospholipase A1-Igamma2 mRNA, complete cds
AT1G14360	1.44789774	1.29E-63	Arabidopsis thaliana UDP-galactose transporter 3 mRNA, complete cds
AT4G05020	1.440409769	2.43E-63	Arabidopsis thaliana NAD(P)H dehydrogenase B2 mRNA, complete cds

AT2G25170	1.179272739	2.53E-63	Arabidopsis thaliana CHD3-type chromatin-remodeling factor PICKLE mRNA, complete cds
AT5G61160	4.786681055	5.07E-63	Arabidopsis thaliana chromosome 5 sequence
AT5G07340	1.338670804	7.97E-63	Arabidopsis thaliana calnexin2 mRNA, complete cds
AT3G28930	1.263177019	5.02E-62	Arabidopsis thaliana avrRpt2-induced protein AIG2 mRNA, complete cds
AT5G52810	1.562839814	8.44E-62	Arabidopsis thaliana chromosome 5 sequence
AT5G20830	1.052434393	2.37E-61	Arabidopsis thaliana sucrose synthase 1 mRNA, complete cds
AT5G63790	1.277498541	1.32E-60	Arabidopsis thaliana NAC domain-containing protein 102 mRNA, complete cds
AT2G39340	1.105129412	1.45E-60	Arabidopsis thaliana SAC3/GANP/Nin1/mts3/eIF-3 p25-family protein mRNA, complete cds
AT3G61820	1.08303178	2.05E-60	Arabidopsis thaliana chromosome 3, complete sequence
AT5G18700	2.28473549	2.09E-60	Arabidopsis thaliana protein kinase family protein with ARM repeat domain mRNA, complete cds
AT2G27170	1.399350485	3.40E-60	Arabidopsis thaliana structural maintenance of chromosome 3 mRNA, complete cds
AT4G00450	1.364776836	2.87E-59	Arabidopsis thaliana transcriptional regulator MED12-like protein mRNA, complete cds
AT4G18600	2.637520711	3.61E-59	Arabidopsis thaliana SCAR family protein WAVE5 mRNA, complete cds
AT2G22010	1.13686741	4.03E-59	Arabidopsis thaliana E3 ubiquitin-protein ligase RKP mRNA, complete cds
AT4G39730	1.144935331	4.14E-59	Arabidopsis thaliana Lipase/lipooxygenase, PLAT/LH2 family protein mRNA, complete cds
AT4G30990	1.620860452	9.58E-59	Arabidopsis thaliana down-regulated in metastasis (DRIM) domain-containing protein mRNA, complete cds
AT5G54100	1.94323362	1.13E-58	Arabidopsis thaliana SPFH/Band 7/PHB domain-containing membrane-associated protein mRNA, complete cds
AT4G18950	1.061779279	1.81E-58	Arabidopsis thaliana Integrin-linked protein kinase family protein mRNA, complete cds
AT3G19190	1.37467252	1.90E-58	Arabidopsis thaliana protein autophagy 2 mRNA, complete cds
AT1G66700	5.365464981	3.31E-58	Arabidopsis thaliana SABATH family methyltransferase PXMT1 mRNA, complete cds
AT3G14172	1.548584551	3.98E-58	Arabidopsis thaliana uncharacterized protein mRNA, complete cds
AT4G33720	1.112067324	4.03E-58	Arabidopsis thaliana chromosome 4 sequence
AT1G24190	1.421031583	4.19E-58	Arabidopsis thaliana paired amphipathic helix protein Sin3-like 3 mRNA, complete cds
AT3G06880	1.508246861	3.20E-57	Arabidopsis thaliana transducin/WD-40 repeat-containing protein mRNA, complete cds

AT2G41960	1.508246861	3.20E-57	Arabidopsis thaliana uncharacterized protein mRNA, complete cds
AT1G19835	1.014490333	5.21E-57	Arabidopsis thaliana uncharacterized protein mRNA, complete cds
AT3G48850	5.33416885	5.98E-57	Arabidopsis thaliana phosphate transporter 3;2 mRNA, complete cds
AT1G30410	1.4662221	1.09E-56	Arabidopsis thaliana multidrug resistance-associated protein 13 mRNA, complete cds
AT2G47410	1.408169432	1.11E-56	Arabidopsis thaliana WD40 domain-containing protein mRNA, complete cds
AT1G67140	1.26691203	2.20E-56	Arabidopsis thaliana protein SWEETIE mRNA, complete cds
AT5G07010	3.588741677	2.49E-56	Arabidopsis thaliana chromosome 5 sequence
AT3G27260	1.093330762	6.90E-56	Arabidopsis thaliana global transcription factor group E8 mRNA, complete cds
AT3G19050	2.481379279	7.99E-56	Arabidopsis thaliana phragmoplast orienting kinesin 2 mRNA, complete cds
AT2G43620	2.583509777	8.00E-56	Arabidopsis thaliana chitinase family protein mRNA, complete cds
AT2G38440	1.198531283	8.13E-56	Arabidopsis thaliana WAVE complex SCAR2 mRNA, complete cds
AT5G42900	2.411817209	1.02E-55	Arabidopsis thaliana cold regulated protein 27 mRNA, complete cds
AT3G59820	1.604477724	1.10E-55	Arabidopsis thaliana LETM1-like protein mRNA, complete cds
AT3G25500	1.161623149	2.19E-55	Arabidopsis thaliana formin-like protein 1 mRNA, complete cds
AT3G03640	1.550827284	5.52E-55	Arabidopsis thaliana beta glucosidase 25 mRNA, complete cds
AT5G16210	1.213577038	5.83E-55	Arabidopsis thaliana HEAT repeat-containing protein mRNA, complete cds
AT4G11890	2.101974263	7.02E-55	Arabidopsis thaliana receptor-like cytosolic kinase ARCK1 mRNA, complete cds
AT3G01420	1.272069308	2.27E-54	Arabidopsis thaliana alpha-dioxygenase mRNA, complete cds
AT4G23170	1.248656196	4.03E-54	Arabidopsis thaliana chromosome 4 sequence
AT2G20340	1.460921098	4.94E-54	Arabidopsis thaliana tyrosine decarboxylase 1 mRNA, complete cds
AT1G68790	1.45262303	5.29E-54	Arabidopsis thaliana little nuclei3 protein mRNA, complete cds
AT1G78490	1.110297362	7.45E-54	Arabidopsis thaliana cytochrome P450, family 708, subfamily A, polypeptide 3 mRNA, complete cds
AT3G22790	1.943501165	8.52E-54	Arabidopsis thaliana protein NETWORKED 1A mRNA, complete cds

AT5G64230	2.326919111	1.29E-53	Arabidopsis thaliana uncharacterized protein mRNA, complete cds
AT5G16730	1.099999261	1.60E-53	Arabidopsis thaliana uncharacterized protein mRNA, complete cds
AT5G39050	1.471451269	2.48E-53	Arabidopsis thaliana chromosome 5 sequence
AT1G71140	2.724643097	2.99E-53	Arabidopsis thaliana MATE efflux family protein mRNA, complete cds
AT3G12810	1.834054313	3.64E-53	Arabidopsis thaliana photoperiod-independent early flowering 1 protein mRNA, complete cds
AT5G24350	1.350317776	4.88E-53	Arabidopsis thaliana uncharacterized protein mRNA, complete cds
AT5G61560	1.943116395	1.22E-52	Arabidopsis thaliana U-box domain-containing protein 51 mRNA, complete cds
AT2G46440	1.06359634	1.52E-52	Arabidopsis thaliana cyclic nucleotide-gated channel 11 mRNA, complete cds
AT5G61270	2.218153012	1.53E-52	Arabidopsis thaliana transcription factor PIF7 mRNA, complete cds
AT1G13609	1.661696752	1.64E-52	Arabidopsis thaliana defensin-like protein 287 mRNA, complete cds
AT4G29520	1.267770383	2.05E-52	Arabidopsis thaliana uncharacterized protein mRNA, complete cds
AT4G15440	1.412891842	2.47E-52	Arabidopsis thaliana hydroperoxide lyase 1 mRNA, complete cds
AT5G46070	1.441607623	2.54E-52	Arabidopsis thaliana Guanylate-binding protein mRNA, complete cds
AT1G74360	2.377875509	2.76E-52	Arabidopsis thaliana putative LRR receptor-like serine/threonine-protein kinase mRNA, complete cds
AT1G09080	6.820950957	4.02E-52	Arabidopsis thaliana protein BIP3 mRNA, complete cds
AT1G54040	1.479583765	1.24E-51	Arabidopsis thaliana epithiospecifier protein mRNA, complete cds
AT2G43820	1.090066425	1.27E-51	Arabidopsis thaliana UDP-glucosyltransferase 74F2 mRNA, complete cds
AT1G78600	1.862629908	1.33E-51	Arabidopsis thaliana light-regulated zinc finger protein 1 mRNA, complete cds
AT3G12980	1.57936497	1.38E-51	Arabidopsis thaliana histone acetyltransferase HAC5 mRNA, complete cds
AT3G63070	1.324401074	2.35E-51	Arabidopsis thaliana Tudor/PWWP/MBT domain-containing protein mRNA, complete cds
AT1G65790	2.560645462	3.27E-51	Arabidopsis thaliana receptor-like serine/threonine-protein kinase SD1-7 mRNA, complete cds
AT2G31970	1.752733723	3.89E-51	Arabidopsis thaliana DNA repair protein RAD50 mRNA, complete cds
AT5G40480	1.314411479	3.91E-51	Arabidopsis thaliana protein EMBRYO DEFECTIVE 3012

			mRNA, complete cds
AT3G54760	1.041484759	4.92E-51	Arabidopsis thaliana dentin sialophosphoprotein-related protein mRNA, complete cds
AT1G17440	1.208321289	5.11E-51	Arabidopsis thaliana transcription initiation factor TFIID subunit 12B mRNA, complete cds
AT2G46560	1.267688208	5.19E-51	Arabidopsis thaliana transducin family protein / WD-40 repeat family protein mRNA, complete cds
AT5G01400	1.383062278	5.56E-51	Arabidopsis thaliana Symplekin/Pta1-like protein mRNA, complete cds
AT1G71030	2.556383436	5.64E-51	Arabidopsis thaliana putative myb family transcription factor mRNA, complete cds
AT1G68890	1.060379274	1.30E-50	Arabidopsis thaliana protein PHYLLO mRNA, complete cds
AT2G03140	1.504866739	1.67E-50	Arabidopsis thaliana alpha/beta-Hydrolases superfamily protein mRNA, complete cds
AT1G13650	1.186845278	1.91E-50	Arabidopsis thaliana uncharacterized protein mRNA, complete cds
AT5G59820	1.716167726	2.10E-50	Arabidopsis thaliana chromosome 5 sequence
AT3G05030	1.561690353	2.15E-50	Arabidopsis thaliana K+/H+ exchanger mRNA, complete cds
AT5G15540	1.452915815	2.68E-50	Arabidopsis thaliana sister-chromatid cohesion protein 2 mRNA, complete cds
AT5G61140	1.165703328	3.00E-50	Arabidopsis thaliana U5 small nuclear ribonucleoprotein helicase mRNA, complete cds
AT5G60930	1.995554595	6.24E-50	Arabidopsis thaliana P-loop containing nucleoside triphosphate hydrolases superfamily protein mRNA, complete cds
AT5G36220	1.673746032	6.69E-50	Arabidopsis thaliana cytochrome P450 81D1 mRNA, complete cds
AT1G32490	1.308043095	1.37E-49	Arabidopsis thaliana DEAH RNA helicase homolog PRP2 mRNA, complete cds
AT3G04720	1.009781715	1.46E-49	Arabidopsis thaliana pathogenesis-related 4 mRNA, complete cds
AT5G05600	1.450604698	3.21E-49	Arabidopsis thaliana oxidoreductase, 2OG-Fe(II) oxygenase family protein mRNA, complete cds
AT2G32680	1.646199831	4.15E-49	Arabidopsis thaliana chromosome 2, complete sequence
AT3G57300	1.008925302	5.92E-49	Arabidopsis thaliana DNA helicase INO80 complex-like 1 mRNA, complete cds
AT3G48650	1.671203838	5.95E-49	Arabidopsis thaliana chromosome 3, complete sequence
AT1G70890	1.191358043	1.04E-48	Arabidopsis thaliana MLP-like protein 43 mRNA, complete cds
AT4G39670	2.498367241	1.38E-48	Arabidopsis thaliana chromosome 4 sequence
AT2G16485	1.451238154	1.46E-48	Arabidopsis thaliana GW repeat- and PHD finger-containing protein NERD mRNA, complete cds

AT5G48850	4.215524354	1.51E-48	Arabidopsis thaliana protein SULPHUR DEFICIENCY-INDUCED 1 mRNA, complete cds
AT2G46240	2.570763936	1.68E-48	Arabidopsis thaliana BCL-2-associated athanogene 6 mRNA, complete cds
AT3G51150	1.764313242	1.73E-48	Arabidopsis thaliana ATP binding microtubule motor family protein mRNA, complete cds
AT1G24070	1.764313242	1.73E-48	Arabidopsis thaliana cellulose synthase-like A10 mRNA, complete cds
AT4G08470	1.222104502	3.28E-48	Arabidopsis thaliana MAPK/ERK kinase kinase 3 mRNA, complete cds
AT3G48190	1.343956345	3.37E-48	Arabidopsis thaliana serine/threonine-protein kinase ATM mRNA, complete cds
AT3G43190	1.788855426	4.34E-48	Arabidopsis thaliana sucrose synthase 4 mRNA, complete cds
AT3G46080	5.656096945	5.13E-48	Arabidopsis thaliana chromosome 3, complete sequence
AT5G59320	2.429864503	8.32E-48	Arabidopsis thaliana non-specific lipid-transfer protein 3 mRNA, complete cds
AT1G17420	1.351996709	1.07E-47	Arabidopsis thaliana lipoxygenase 3 mRNA, complete cds
AT4G26270	1.635516982	1.33E-47	Arabidopsis thaliana 6-phosphofructokinase 3 mRNA, complete cds
AT4G15760	1.107849754	2.40E-47	Arabidopsis thaliana monooxygenase 1 mRNA, complete cds
AT2G36145	1.563420011	2.53E-47	Arabidopsis thaliana uncharacterized protein mRNA, complete cds
AT5G13080	3.645668745	3.09E-47	Arabidopsis thaliana putative WRKY transcription factor 75 mRNA, complete cds
AT1G76580	1.640176942	3.25E-47	Arabidopsis thaliana squamosa promoter-binding-like protein 16 mRNA, complete cds
AT2G22795	1.063563278	3.94E-47	Arabidopsis thaliana uncharacterized protein mRNA, complete cds
AT1G02520	1.660536912	6.22E-47	Arabidopsis thaliana P-glycoprotein 11 mRNA, complete cds
AT2G04040	3.907271196	6.49E-47	Arabidopsis thaliana MATE efflux family protein DTX1 mRNA, complete cds
AT2G46400	2.10860915	6.97E-47	Arabidopsis thaliana putative WRKY transcription factor 46 mRNA, complete cds
AT5G63780	1.154163992	7.04E-47	Arabidopsis thaliana putative E3 ligase SHA1 mRNA, complete cds
AT1G17500	1.15065105	7.50E-47	Arabidopsis thaliana ATPase E1-E2 type family protein / haloacid dehalogenase-like hydrolase family protein mRNA, complete cds
AT2G30140	1.517080692	9.39E-47	Arabidopsis thaliana UDP-glycosyltransferase 87A2 mRNA, complete cds
AT1G54020	3.472299769	9.63E-47	Arabidopsis thaliana GDSL esterase/lipase mRNA,

			complete cds
AT4G23150	4.523646649	1.01E-46	Arabidopsis thaliana cysteine-rich receptor-like protein kinase 7 mRNA, complete cds
AT2G42270	1.173107818	1.11E-46	Arabidopsis thaliana U5 small nuclear ribonucleoprotein helicase mRNA, complete cds
AT3G45260	1.652588159	1.26E-46	Arabidopsis thaliana C2H2-like zinc finger protein mRNA, complete cds
AT3G05880	1.130447813	1.29E-46	Arabidopsis thaliana Hydrophobic protein RCI2A mRNA, complete cds
AT4G10500	2.632984515	1.38E-46	Arabidopsis thaliana oxidoreductase, 2OG-Fe(II) oxygenase family protein mRNA, complete cds
AT1G23230	1.402913706	1.45E-46	Arabidopsis thaliana mediator of RNA polymerase II transcription subunit 23 mRNA, complete cds
AT1G21240	3.514102876	2.22E-46	Arabidopsis thaliana wall-associated receptor kinase 3 mRNA, complete cds
AT3G51660	1.71765276	3.71E-46	Arabidopsis thaliana Tautomerase/MIF superfamily protein mRNA, complete cds
AT4G31210	1.307993556	6.90E-46	Arabidopsis thaliana DNA topoisomerase, type IA, core mRNA, complete cds
AT3G61280	1.79202862	7.17E-46	Arabidopsis thaliana lipopolysaccharide-modifying domain-containing protein mRNA, complete cds
AT4G16880	2.944222332	7.86E-46	Arabidopsis thaliana leucine-rich repeat (LRR) family protein mRNA, complete cds
AT3G56410	2.050081674	9.84E-46	Arabidopsis thaliana uncharacterized protein mRNA, complete cds
AT3G09010	2.019454246	1.19E-45	Arabidopsis thaliana protein kinase mRNA, complete cds
AT1G03310	1.196934425	2.23E-45	Arabidopsis thaliana Isoamylase 2 mRNA, complete cds
AT1G06670	1.309283767	2.62E-45	Arabidopsis thaliana nuclear DEIH-boxhelicase mRNA, complete cds
AT2G03150	1.096593883	2.88E-45	Arabidopsis thaliana protein EMBRYO DEFECTIVE 1579 mRNA, complete cds
AT4G14230	1.297745442	3.53E-45	Arabidopsis thaliana CBS domain-containing protein with a domain of unknown function (DUF21) mRNA, complete cds
AT5G27060	4.802448371	3.58E-45	Arabidopsis thaliana chromosome 5 sequence
AT1G53440	1.007221338	4.94E-45	Arabidopsis thaliana putative LRR receptor-like serine/threonine-protein kinase mRNA, complete cds
AT4G39030	1.476187855	1.10E-44	Arabidopsis thaliana enhanced disease susceptibility 5 mRNA, complete cds
AT1G24300	1.302733157	1.67E-44	Arabidopsis thaliana GYF domain-containing protein mRNA, complete cds
AT5G44180	1.117537467	1.83E-44	Arabidopsis thaliana protein RINGLET2 mRNA, complete cds
AT4G00800	1.06218407	2.03E-44	Arabidopsis thaliana transducin family protein / WD-40

			repeat family protein mRNA, complete cds
AT5G61190	1.334940827	2.89E-44	Arabidopsis thaliana putative endonuclease or glycosyl hydrolase with C2H2-type zinc finger domain mRNA, complete cds
AT5G51830	1.358200972	3.68E-44	Arabidopsis thaliana pfkB-like carbohydrate kinase family protein mRNA, complete cds
AT3G47910	1.215524354	6.12E-44	Arabidopsis thaliana ubiquitin carboxyl-terminal hydrolase-related protein mRNA, complete cds
AT1G58230	1.257114544	8.47E-44	Arabidopsis thaliana WD40 and Beach domain-containing protein mRNA, complete cds
AT3G54280	1.228512892	9.55E-44	Arabidopsis thaliana TATA-binding protein-associated factor BTAF1 mRNA, complete cds
AT3G01830	3.616756054	9.69E-44	Arabidopsis thaliana chromosome 3, complete sequence
AT2G47890	1.642970157	1.58E-43	Arabidopsis thaliana zinc finger protein CONSTANS-LIKE 13 mRNA, complete cds
AT2G29350	1.423276324	1.79E-43	Arabidopsis thaliana senescence-associated protein 13 mRNA, complete cds
AT3G60420	1.100225566	1.91E-43	Arabidopsis thaliana phosphoglycerate mutase family protein mRNA, complete cds
AT1G15940	1.586470789	2.80E-43	Arabidopsis thaliana aspartyl beta-hydroxylase N-terminal region domain-containing protein mRNA, complete cds
AT5G04020	1.829901584	3.21E-43	Arabidopsis thaliana calmodulin-binding protein mRNA, complete cds
AT5G26860	1.303217722	3.49E-43	Arabidopsis thaliana lon protease 1 mRNA, complete cds
AT4G31160	1.343294766	5.07E-43	Arabidopsis thaliana DDB1- and CUL4-associated factor-1 mRNA, complete cds
AT1G08260	1.763673608	6.69E-43	Arabidopsis thaliana DNA polymerase epsilon catalytic subunit A mRNA, complete cds
AT5G05340	4.158191179	1.14E-42	Arabidopsis thaliana peroxidase 52 mRNA, complete cds
AT3G24982	2.566506459	1.45E-42	Arabidopsis thaliana receptor like protein 40 mRNA, complete cds
AT4G10930	2.06628673	3.10E-42	Arabidopsis thaliana uncharacterized protein mRNA, complete cds
AT3G28540	1.84416655	4.14E-42	Arabidopsis thaliana chromosome 3, complete sequence
AT1G56120	2.225516125	4.15E-42	Arabidopsis thaliana Leucine-rich repeat transmembrane protein kinase mRNA, complete cds
AT3G10010	2.070121671	5.42E-42	Arabidopsis thaliana putative DNA glycosylase mRNA, complete cds
AT5G01100	1.476974947	5.78E-42	Arabidopsis thaliana O-fucosyltransferase family protein mRNA, complete cds
AT3G44200	1.548402748	7.77E-42	Arabidopsis thaliana serine/threonine-protein kinase Nek5 mRNA, complete cds
AT1G56060	5.452563551	8.08E-42	Arabidopsis thaliana uncharacterized protein mRNA,

			complete cds
AT1G27330	1.315060027	9.41E-42	Arabidopsis thaliana ribosome associated membrane protein RAMP4 mRNA, complete cds
AT1G56510	1.029434981	1.10E-41	Arabidopsis thaliana TIR-NB-LRR disease resistance protein mRNA, complete cds
AT5G22450	1.09268195	1.95E-41	Arabidopsis thaliana uncharacterized protein mRNA, complete cds
AT5G13320	2.303942063	2.17E-41	Arabidopsis thaliana 4-substituted benzoates-glutamate ligase GH3.12 mRNA, complete cds
AT5G65440	1.100987922	2.41E-41	Arabidopsis thaliana uncharacterized protein mRNA, complete cds
AT5G48600	1.791793978	2.71E-41	Arabidopsis thaliana structural maintenance of chromosomes protein 4 mRNA, complete cds
AT1G64160	3.917183079	3.16E-41	Arabidopsis thaliana chromosome 1 sequence
AT3G44190	1.024718989	5.02E-41	Arabidopsis thaliana FAD/NAD(P)-binding oxidoreductase family protein mRNA, complete cds
AT4G24680	1.006147746	5.58E-41	Arabidopsis thaliana protein MODIFIER OF SNC1 1 mRNA, complete cds
AT3G45850	1.064592774	5.63E-41	Arabidopsis thaliana P-loop containing nucleoside triphosphate hydrolases superfamily protein mRNA, complete cds
AT4G01360	4.350944563	6.15E-41	Arabidopsis thaliana BYPASS1-related protein mRNA, complete cds
AT1G51800	1.256675136	6.65E-41	Arabidopsis thaliana putative leucine-rich repeat protein kinase mRNA, complete cds
AT1G51610	1.500390203	6.89E-41	Arabidopsis thaliana metal tolerance protein C4 mRNA, complete cds
AT1G17110	1.097829311	1.03E-40	Arabidopsis thaliana ubiquitin carboxyl-terminal hydrolase 15 mRNA, complete cds
AT4G25810	1.778119041	1.65E-40	Arabidopsis thaliana probable xyloglucan endotransglucosylase/hydrolase protein 23 mRNA, complete cds
AT3G28580	3.467670443	1.93E-40	Arabidopsis thaliana chromosome 3, complete sequence
AT1G22070	1.22414345	4.05E-40	Arabidopsis thaliana transcription factor TGA3 mRNA, complete cds
AT1G06720	1.335600806	5.48E-40	Arabidopsis thaliana BMS1 domain-containing protein mRNA, complete cds
AT2G35980	3.640830189	6.87E-40	Arabidopsis thaliana chromosome 2, complete sequence
AT1G45160	1.089577049	7.61E-40	Arabidopsis thaliana protein kinase mRNA, complete cds
AT5G38510	1.157919361	8.68E-40	Arabidopsis thaliana rhomboid-related intramembrane serine protease-like protein mRNA, complete cds
AT4G13410	3.292145635	9.54E-40	Arabidopsis thaliana putative mannan synthase 15 mRNA, complete cds
AT1G58561	1.86760105	9.55E-40	Arabidopsis thaliana chromosome 1 sequence

AT2G34357	1.035989358	1.81E-39	Arabidopsis thaliana NUC173 domain-containing protein mRNA, complete cds
AT1G02990	1.733939593	1.93E-39	Arabidopsis thaliana uncharacterized protein mRNA, complete cds
AT5G17760	1.406908886	2.48E-39	Arabidopsis thaliana chromosome 5 sequence
AT1G73470	1.368771918	2.62E-39	Arabidopsis thaliana uncharacterized protein mRNA, complete cds
AT1G04980	1.165711387	2.89E-39	Arabidopsis thaliana protein disulfide-isomerase like 2-2 mRNA, complete cds
AT1G03910	1.055934854	3.68E-39	Arabidopsis thaliana uncharacterized protein mRNA, complete cds
AT1G32960	3.944222332	4.84E-39	Arabidopsis thaliana Subtilase family protein SBT3.3 mRNA, complete cds
AT1G51940	1.268695013	5.05E-39	Arabidopsis thaliana LysM type receptor kinase-like protein mRNA, complete cds
AT3G10940	1.146835862	6.41E-39	Arabidopsis thaliana phosphoglucan phosphatase LSF2 mRNA, complete cds
AT2G31865	1.437531491	8.30E-39	Arabidopsis thaliana poly(ADP-ribose) glycohydrolase 2 mRNA, complete cds
AT1G72440	1.157954846	9.04E-39	Arabidopsis thaliana protein SLOW WALKER2 mRNA, complete cds
AT1G07160	4.27386834	1.45E-38	Arabidopsis thaliana putative protein phosphatase 2C 2 mRNA, complete cds
AT1G53540	5.33416885	1.48E-38	Arabidopsis thaliana chromosome 1 sequence
AT4G29440	1.698619295	1.54E-38	Arabidopsis thaliana regulator of Vps4 activity protein mRNA, complete cds
AT5G37990	1.69166794	2.06E-38	Arabidopsis thaliana probable S-adenosylmethionine-dependent methyltransferase mRNA, complete cds
AT5G41750	1.266666751	2.27E-38	Arabidopsis thaliana TIR-NBS-LRR class disease resistance protein mRNA, complete cds
AT4G32620	1.260551433	2.57E-38	Arabidopsis thaliana Enhancer of polycomb-like transcription factor protein mRNA, complete cds
AT5G48250	1.488137736	3.37E-38	Arabidopsis thaliana zinc finger protein CONSTANS-LIKE 10 mRNA, complete cds
AT4G27830	1.157014411	3.45E-38	Arabidopsis thaliana beta glucosidase 10 mRNA, complete cds
AT1G48540	1.141847098	3.87E-38	Arabidopsis thaliana Outer arm dynein light chain 1 protein mRNA, complete cds
AT5G56890	1.125361353	3.93E-38	Arabidopsis thaliana protein kinase family protein mRNA, complete cds
AT1G72330	1.488952086	7.18E-38	Arabidopsis thaliana alanine aminotransferase 2 mRNA, complete cds
AT5G13220	2.392049766	8.59E-38	Arabidopsis thaliana protein TIFY 9 mRNA, complete cds

AT1G75310	1.854915791	9.83E-38	Arabidopsis thaliana auxin-like 1 protein mRNA, complete cds
AT1G67560	1.004983645	1.15E-37	Arabidopsis thaliana lipoxygenase 6 mRNA, complete cds
AT4G39210	1.408169432	1.32E-37	Arabidopsis thaliana glucose-1-phosphate adenylyltransferase large subunit 3 mRNA, complete cds
AT3G23890	1.243110185	1.40E-37	Arabidopsis thaliana DNA topoisomerase 2 mRNA, complete cds
AT2G04080	1.476777874	1.67E-37	Arabidopsis thaliana MATE efflux family protein mRNA, complete cds
AT4G12780	1.163365763	1.97E-37	Arabidopsis thaliana auxilin-related protein 1 mRNA, complete cds
AT4G16630	1.365623737	2.04E-37	Arabidopsis thaliana DEAD-box ATP-dependent RNA helicase 28 mRNA, complete cds
AT5G27600	1.266964579	2.24E-37	Arabidopsis thaliana long-chain acyl-CoA synthetase 7 mRNA, complete cds
AT5G18620	1.10654832	3.03E-37	Arabidopsis thaliana chromatin remodeling factor17 mRNA, complete cds
AT3G26840	1.431673369	3.13E-37	Arabidopsis thaliana phytyl ester synthesis and diacylglycerol acyltransferase activity protein mRNA, complete cds
AT3G62740	1.812956834	3.39E-37	Arabidopsis thaliana beta glucosidase 7 mRNA, complete cds
AT1G28190	1.875474241	4.40E-37	Arabidopsis thaliana chromosome 1 sequence
AT1G33970	1.347765858	5.16E-37	Arabidopsis thaliana AIG1 domain-containing protein mRNA, complete cds
AT1G26380	2.020353401	6.54E-37	Arabidopsis thaliana chromosome 1 sequence
AT5G23150	1.404137678	7.34E-37	Arabidopsis thaliana ENHANCER OF AG-4 protein 2 mRNA, complete cds
AT1G51090	3.69673893	7.96E-37	Arabidopsis thaliana uncharacterized protein mRNA, complete cds
AT5G15680	1.124921805	9.16E-37	Arabidopsis thaliana cell morphogenesis domain-containing protein mRNA, complete cds
AT1G17450	1.932531921	1.14E-36	Arabidopsis thaliana B-block binding subunit of TFIIIC mRNA, complete cds
AT5G13210	2.23824443	1.18E-36	Arabidopsis thaliana chromosome 5 sequence
AT1G17180	3.474511927	1.38E-36	Arabidopsis thaliana glutathione S-transferase TAU 25 mRNA, complete cds
AT5G12030	3.248512766	1.56E-36	Arabidopsis thaliana chromosome 5 sequence
AT5G13490	1.142824865	2.47E-36	Arabidopsis thaliana ADP/ATP carrier protein 2 mRNA, complete cds
AT5G16780	1.000941669	2.57E-36	Arabidopsis thaliana SART-1 family protein DOT2 mRNA, complete cds
AT3G01460	1.531718784	2.80E-36	Arabidopsis thaliana methyl-CPG-binding domain 9 mRNA, complete cds

AT1G10930	1.762019001	4.22E-36	Arabidopsis thaliana ATP-dependent DNA helicase Q-like 4A mRNA, complete cds
AT2G40770	1.966659721	5.22E-36	Arabidopsis thaliana RING-finger, DEAD-like helicase, PHD and SNF2 domain-containing protein mRNA, complete cds
AT4G12120	1.667637223	8.08E-36	Arabidopsis thaliana protein transport sec1b mRNA, complete cds
AT5G17910	1.135013138	1.13E-35	Arabidopsis thaliana uncharacterized protein mRNA, complete cds
AT2G02810	1.474423344	1.64E-35	Arabidopsis thaliana UDP-galactose transporter 1 mRNA, complete cds
AT4G09760	1.231601941	3.06E-35	Arabidopsis thaliana choline synthase mRNA, complete cds
AT5G38840	1.551435511	4.16E-35	Arabidopsis thaliana SMAD/FHA domain-containing protein mRNA, complete cds
AT5G06460	1.1572608	4.78E-35	Arabidopsis thaliana ubiquitin-activating enzyme E1 2 mRNA, complete cds
AT3G55760	1.612870527	7.17E-35	Arabidopsis thaliana uncharacterized protein mRNA, complete cds
AT1G15125	1.544847851	9.51E-35	Arabidopsis thaliana S-adenosyl-L-methionine-dependent methyltransferase mRNA, complete cds
AT5G04340	1.339177633	1.02E-34	Arabidopsis thaliana chromosome 5 sequence
AT4G16250	1.513108679	1.23E-34	Arabidopsis thaliana phytochrome D mRNA, complete cds
AT5G40340	1.189911266	1.25E-34	Arabidopsis thaliana PWWP domain-containing protein mRNA, complete cds
AT1G73325	4.640830189	1.31E-34	Arabidopsis thaliana chromosome 1 sequence
AT5G17700	1.569632854	1.35E-34	Arabidopsis thaliana MATE efflux family protein mRNA, complete cds
AT5G43310	1.201718554	1.57E-34	Arabidopsis thaliana COP1-interacting protein-like protein mRNA, complete cds
AT4G17260	1.201718554	1.57E-34	Arabidopsis thaliana lactate/malate dehydrogenase family protein mRNA, complete cds
AT1G73480	1.226562625	1.94E-34	Arabidopsis thaliana alpha/beta-Hydrolases superfamily protein mRNA, complete cds
AT4G24450	1.930590466	2.37E-34	Arabidopsis thaliana phosphoglucan, water dikinase mRNA, complete cds
AT1G13990	1.25249068	4.78E-34	Arabidopsis thaliana uncharacterized protein mRNA, complete cds
AT2G48060	1.013113265	4.84E-34	Arabidopsis thaliana uncharacterized protein mRNA, complete cds
AT3G62900	1.186744937	5.50E-34	Arabidopsis thaliana CW-type zinc-finger protein mRNA, complete cds
AT4G21840	3.391095918	5.52E-34	Arabidopsis thaliana methionine sulfoxide reductase B8

			mRNA, complete cds
AT2G20320	1.31306873	7.55E-34	Arabidopsis thaliana DENN (AEX-3) domain-containing protein mRNA, complete cds
AT1G44350	1.417158215	7.89E-34	Arabidopsis thaliana IAA-amino acid hydrolase ILR1-like 6 mRNA, complete cds
AT2G39260	1.031054416	1.06E-33	Arabidopsis thaliana regulator of nonsense transcripts UPF2 mRNA, complete cds
AT4G27820	1.274152084	1.26E-33	Arabidopsis thaliana beta glucosidase 9 mRNA, complete cds
AT1G02850	2.041694898	1.30E-33	Arabidopsis thaliana beta glucosidase 11 mRNA, complete cds
AT4G33200	1.021251997	1.61E-33	Arabidopsis thaliana myosin-15 mRNA, complete cds
AT1G79150	1.48833978	1.75E-33	Arabidopsis thaliana nucleolar complex-associated protein domain-containing protein mRNA, complete cds
AT1G77460	1.528759573	2.82E-33	Arabidopsis thaliana armadillo/beta-catenin-like repeat and C2 domain-containing protein mRNA, complete cds
AT4G34131	2.141523772	3.91E-33	Arabidopsis thaliana chromosome 4 sequence
AT5G02780	2.153355533	4.09E-33	Arabidopsis thaliana glutathione transferase lambda 1 mRNA, complete cds
AT5G43745	1.125176999	4.58E-33	Arabidopsis thaliana uncharacterized protein mRNA, complete cds
AT4G12900	2.408169432	4.90E-33	Arabidopsis thaliana Gamma interferon responsive lysosomal thiol (GILT) reductase family protein mRNA, complete cds
AT5G25260	2.573023444	5.45E-33	Arabidopsis thaliana SPFH/Band 7/PHB domain-containing membrane-associated protein mRNA, complete cds
AT5G46330	1.830624403	5.87E-33	Arabidopsis thaliana LRR receptor-like serine/threonine-protein kinase FLS2 mRNA, complete cds
AT3G46090	4.215524354	6.08E-33	Arabidopsis thaliana chromosome 3, complete sequence
AT4G26190	1.064809956	6.89E-33	Arabidopsis thaliana Haloacid dehalogenase-like hydrolase (HAD) superfamily protein mRNA, complete cds
AT3G23120	1.552745004	7.31E-33	Arabidopsis thaliana chromosome 3, complete sequence
AT4G19020	1.223251716	1.04E-32	Arabidopsis thaliana chromomethylase 2 mRNA, complete cds
AT1G13220	1.592426724	1.10E-32	Arabidopsis thaliana protein CROWDED NUCLEI 2 mRNA, complete cds
AT1G08060	1.551904909	1.31E-32	Arabidopsis thaliana helicase protein MOM1 mRNA, complete cds
AT4G12770	1.027497546	1.61E-32	Arabidopsis thaliana auxilin-related protein 2 mRNA, complete cds
AT5G61810	1.556561272	3.29E-32	Arabidopsis thaliana ATP-Mg/Pi transporter mRNA,

			complete cds
AT2G38250	3.46475296	3.32E-32	Arabidopsis thaliana trihelix transcription factor GT-3b mRNA, complete cds
AT3G24880	1.8480816	4.25E-32	Arabidopsis thaliana Helicase/SANT-associated, DNA binding protein mRNA, complete cds
AT1G43910	1.18577701	5.63E-32	Arabidopsis thaliana P-loop containing nucleoside triphosphate hydrolases superfamily protein mRNA, complete cds
AT5G12400	1.126756497	6.30E-32	Arabidopsis thaliana PHD-finger and DNA binding domain-containing protein mRNA, complete cds
AT3G24870	1.567983117	6.43E-32	Arabidopsis thaliana Helicase/SANT-associated, DNA binding protein mRNA, complete cds
AT2G45560	1.235104723	6.84E-32	Arabidopsis thaliana cytochrome P450 76C1 mRNA, complete cds
AT5G59720	3.053074473	8.04E-32	Arabidopsis thaliana chromosome 5 sequence
AT2G35110	1.052711713	8.23E-32	Arabidopsis thaliana protein NAP1 mRNA, complete cds
AT2G37760	1.108133943	9.27E-32	Arabidopsis thaliana aldo-keto reductase family 4 member C8 mRNA, complete cds
AT2G30770	1.603565121	1.22E-31	Arabidopsis thaliana cytochrome P450, family 71, subfamily A, polypeptide 13 mRNA, complete cds
AT4G02660	2.686154179	1.45E-31	Arabidopsis thaliana Beige/BEACH and WD40 domain-containing protein mRNA, complete cds
AT4G21390	1.696514926	1.49E-31	Arabidopsis thaliana G-type lectin S-receptor-like serine/threonine-protein kinase B120 mRNA, complete cds
AT4G02400	1.311493412	1.53E-31	Arabidopsis thaliana U3 ribonucleoprotein family protein mRNA, complete cds
AT3G17360	2.16360022	1.66E-31	Arabidopsis thaliana phragmoplast orienting kinesin 1 mRNA, complete cds
AT2G29310	1.341593957	1.68E-31	Arabidopsis thaliana tropinone reductase-like protein mRNA, complete cds
AT2G29990	1.834142409	1.70E-31	Arabidopsis thaliana alternative NAD(P)H dehydrogenase 2 mRNA, complete cds
AT5G27030	1.108335108	1.70E-31	Arabidopsis thaliana Topless-related protein 3 mRNA, complete cds
AT2G23840	1.700951181	1.73E-31	Arabidopsis thaliana HNH endonuclease mRNA, complete cds
AT1G54575	2.869617396	3.28E-31	Arabidopsis thaliana chromosome 1 sequence
AT5G13750	1.236483972	3.90E-31	Arabidopsis thaliana zinc induced facilitator-like 1 protein mRNA, complete cds
AT3G56720	1.141048895	5.59E-31	Arabidopsis thaliana uncharacterized protein mRNA, complete cds
AT2G32290	1.496525306	7.45E-31	Arabidopsis thaliana beta-amylase 6 mRNA, complete cds
AT1G28230	2.421844369	8.97E-31	Arabidopsis thaliana purine permease 1 mRNA, complete

			cds
AT2G25660	1.007221338	1.36E-30	Arabidopsis thaliana embryo defective 2410 protein mRNA, complete cds
AT2G23740	1.446365755	1.41E-30	Arabidopsis thaliana histone-lysine N-methyltransferase SUVR5 mRNA, complete cds
AT4G38560	2.496525306	2.20E-30	Arabidopsis thaliana phospholipase like protein (PEARLI 4) mRNA, complete cds
AT5G67470	1.234633177	2.59E-30	Arabidopsis thaliana formin-like protein 6 mRNA, complete cds
AT5G55920	1.067019357	2.88E-30	Arabidopsis thaliana ribosome biogenesis protein OLI2 mRNA, complete cds
AT5G54860	1.210094806	3.34E-30	Arabidopsis thaliana probable folate-biopterin transporter 4 mRNA, complete cds
AT3G63380	2.031379402	3.36E-30	Arabidopsis thaliana chromosome 3, complete sequence
AT5G44420	4.097468592	4.95E-30	Arabidopsis thaliana ethylene- and jasmonate-responsive plant defensin mRNA, complete cds
AT4G22530	1.971598771	5.86E-30	Arabidopsis thaliana S-adenosyl-L-methionine-dependent methyltransferase domain-containing protein mRNA, complete cds
AT3G53230	1.359525839	6.29E-30	Arabidopsis thaliana cell division control protein 48-B mRNA, complete cds
AT1G28610	1.578094433	8.80E-30	Arabidopsis thaliana GDSL esterase/lipase mRNA, complete cds
AT3G15120	1.451345573	9.28E-30	Arabidopsis thaliana P-loop containing nucleoside triphosphate hydrolases superfamily protein mRNA, complete cds
AT1G30755	1.130184684	9.76E-30	Arabidopsis thaliana uncharacterized protein mRNA, complete cds
AT4G25940	1.033180093	9.96E-30	Arabidopsis thaliana ENTH/ANTH/VHS superfamily protein mRNA, complete cds
AT1G72900	1.158134255	1.09E-29	Arabidopsis thaliana Toll-Interleukin-Resistance domain-containing protein mRNA, complete cds
AT1G47900	1.212998479	1.11E-29	Arabidopsis thaliana uncharacterized protein mRNA, complete cds
AT1G61140	1.090799619	1.12E-29	Arabidopsis thaliana protein EMBRYO SAC DEVELOPMENT ARREST 16 mRNA, complete cds
AT4G17770	1.224293518	1.29E-29	Arabidopsis thaliana putative trehalose phosphatase/synthase 5 mRNA, complete cds
AT5G55820	2.086241337	1.36E-29	Arabidopsis thaliana uncharacterized protein mRNA, complete cds
AT1G76520	1.086241337	1.74E-29	Arabidopsis thaliana auxin efflux carrier family protein mRNA, complete cds
AT1G52780	1.018195949	1.86E-29	Arabidopsis thaliana chromosome 1 sequence
AT1G54820	1.054060931	1.88E-29	Arabidopsis thaliana putative serine/threonine protein

			kinase mRNA, complete cds
AT1G70070	1.025014538	2.76E-29	Arabidopsis thaliana DEAD-box ATP-dependent RNA helicase ISE2 mRNA, complete cds
AT3G23150	1.850753258	3.16E-29	Arabidopsis thaliana ethylene receptor 2 mRNA, complete cds
AT1G77600	1.347982426	3.43E-29	Arabidopsis thaliana ARM repeat superfamily protein mRNA, complete cds
AT3G47780	1.893596259	3.48E-29	Arabidopsis thaliana ABC transporter A family member 7 mRNA, complete cds
AT1G65580	1.019315864	3.76E-29	Arabidopsis thaliana Type II inositol-1,4,5-trisphosphate 5-phosphatase FRA3 mRNA, complete cds
AT5G60900	1.115486157	4.64E-29	Arabidopsis thaliana receptor-like protein kinase 1 mRNA, complete cds
AT1G61610	3.049715461	5.47E-29	Arabidopsis thaliana putative G-type lectin S-receptor-like serine/threonine-protein kinase mRNA, complete cds
AT5G62410	1.547689301	7.64E-29	Arabidopsis thaliana structural maintenance of chromosome 2 mRNA, complete cds
AT1G04770	1.675733496	8.00E-29	Arabidopsis thaliana tetratricopeptide repeat-containing protein mRNA, complete cds
AT5G41120	1.814794691	8.76E-29	Arabidopsis thaliana Esterase/lipase/thioesterase family protein mRNA, complete cds
AT2G38750	1.186935336	1.10E-28	Arabidopsis thaliana annexin D4 mRNA, complete cds
AT1G08600	1.255427442	1.22E-28	Arabidopsis thaliana protein ATRX mRNA, complete cds
AT4G27595	1.803169178	1.22E-28	Arabidopsis thaliana uncharacterized protein mRNA, complete cds
AT1G65890	2.858830841	1.24E-28	Arabidopsis thaliana acyl activating enzyme 12 mRNA, complete cds
AT2G28720	1.885543987	1.59E-28	Arabidopsis thaliana chromosome 2, complete sequence
AT1G21120	1.061463764	1.67E-28	Arabidopsis thaliana O-methyltransferase family protein mRNA, complete cds
AT3G13290	1.361519338	2.32E-28	Arabidopsis thaliana varicose-related protein mRNA, complete cds
AT3G49570	5.28263855	2.51E-28	Arabidopsis thaliana chromosome 3, complete sequence
AT1G44910	1.124511354	2.95E-28	Arabidopsis thaliana pre-mRNA-processing protein 40A mRNA, complete cds
AT2G25320	1.124511354	2.95E-28	Arabidopsis thaliana TRAF-like protein mRNA, complete cds
AT1G32870	1.306507457	2.99E-28	Arabidopsis thaliana NAC domain protein 13 mRNA, complete cds
AT1G19530	1.79304496	3.04E-28	Arabidopsis thaliana uncharacterized protein mRNA, complete cds
AT1G61210	1.074521573	4.29E-28	Arabidopsis thaliana protein DWD hypersensitive to ABA 3 mRNA, complete cds

AT1G55250	1.179533257	4.58E-28	Arabidopsis thaliana E3 ubiquitin-protein ligase HUB2 mRNA, complete cds
AT5G24660	2.229199291	4.97E-28	Arabidopsis thaliana chromosome 5 sequence
AT5G07940	1.11410813	5.93E-28	Arabidopsis thaliana uncharacterized protein mRNA, complete cds
AT1G76960	3.11598868	6.22E-28	Arabidopsis thaliana uncharacterized protein mRNA, complete cds
AT1G16710	1.056541103	7.02E-28	Arabidopsis thaliana histone acetyltransferase HAC12 mRNA, complete cds
AT5G16680	1.128061513	8.72E-28	Arabidopsis thaliana RING/FYVE/PHD zinc finger-containing protein mRNA, complete cds
AT5G52280	1.136681075	8.90E-28	Arabidopsis thaliana Myosin heavy chain-related protein mRNA, complete cds
AT4G04330	1.133894312	1.01E-27	Arabidopsis thaliana Chaperonin-like RbcX protein mRNA, complete cds
AT3G50240	1.119823937	1.04E-27	Arabidopsis thaliana kinesin-related protein mRNA, complete cds
AT2G38770	1.047767189	1.09E-27	Arabidopsis thaliana embryo defective 2765 mRNA, complete cds
AT5G49680	1.754259577	1.24E-27	Arabidopsis thaliana SABRE-like protein mRNA, complete cds
AT2G28620	1.355516683	1.27E-27	Arabidopsis thaliana kinesin motor protein-related protein mRNA, complete cds
AT4G27860	1.071980644	1.32E-27	Arabidopsis thaliana vacuolar iron transporter-like protein mRNA, complete cds
AT5G67340	1.439030395	2.00E-27	Arabidopsis thaliana ARM repeat superfamily protein mRNA, complete cds
AT3G23550	1.563055034	2.17E-27	Arabidopsis thaliana MATE efflux family protein LAL5 mRNA, complete cds
AT2G34930	1.379741023	2.26E-27	Arabidopsis thaliana chromosome 2, complete sequence
AT1G55970	1.652588159	2.26E-27	Arabidopsis thaliana histone acetyltransferase of the CBP family 4 mRNA, complete cds
AT4G02060	1.101454431	2.45E-27	Arabidopsis thaliana protein PROLIFERA mRNA, complete cds
AT4G35180	4.841128839	2.77E-27	Arabidopsis thaliana LYS/HIS transporter 7 mRNA, complete cds
AT5G39410	1.106778268	2.88E-27	Arabidopsis thaliana Saccharopine dehydrogenase mRNA, complete cds
AT1G55540	1.337616152	3.22E-27	Arabidopsis thaliana nuclear pore complex protein LNO1 mRNA, complete cds
AT3G06290	1.069367518	4.06E-27	Arabidopsis thaliana SAC3/GANP/Nin1/mts3/eIF-3 p25 family protein mRNA, complete cds
AT3G23670	1.762238914	4.58E-27	Arabidopsis thaliana kinesin-like protein KIN12B mRNA, complete cds

AT5G33290	1.112616752	7.59E-27	Arabidopsis thaliana xylogalacturonan beta-1,3-xylosyltransferase mRNA, complete cds
AT2G20290	1.661553668	9.92E-27	Arabidopsis thaliana myosin-like protein XIG mRNA, complete cds
AT1G58225	2.611964634	1.24E-26	Arabidopsis thaliana uncharacterized protein mRNA, complete cds
AT5G39500	1.148853314	1.52E-26	Arabidopsis thaliana ARF guanine-nucleotide exchange factor GNL1 mRNA, complete cds
AT1G10585	2.954137801	1.82E-26	Arabidopsis thaliana basic helix-loop-helix domain-containing protein mRNA, complete cds
AT5G52760	1.893596259	2.32E-26	Arabidopsis thaliana copper transport family protein mRNA, complete cds
AT1G13470	3.045599352	3.74E-26	Arabidopsis thaliana uncharacterized protein mRNA, complete cds
AT1G19220	1.376583089	3.90E-26	Arabidopsis thaliana auxin response factor 19 mRNA, complete cds
AT3G62150	1.056797559	4.38E-26	Arabidopsis thaliana ABC transporter B family member 21 mRNA, complete cds
AT1G26130	1.469750754	4.52E-26	Arabidopsis thaliana putative phospholipid-transporting ATPase 12 mRNA, complete cds
AT2G05510	5.145135026	8.87E-26	Arabidopsis thaliana glycine-rich protein mRNA, complete cds
AT4G03060	1.441241253	8.93E-26	Arabidopsis thaliana Col-0 2-oxoglutarate-dependent dioxygenase (AOP2) pseudogene, mRNA sequence
AT5G26170	2.64423679	9.30E-26	Arabidopsis thaliana putative WRKY transcription factor 50 mRNA, complete cds
AT5G22430	1.986705663	9.93E-26	Arabidopsis thaliana pollen Ole e 1 allergen and extensin family protein mRNA, complete cds
AT1G05910	1.102543149	1.14E-25	Arabidopsis thaliana cell division cycle protein 48-related protein mRNA, complete cds
AT2G44080	1.463911984	1.20E-25	Arabidopsis thaliana ARGOS-like protein mRNA, complete cds
AT1G57630	2.96759684	1.50E-25	Arabidopsis thaliana chromosome 1 sequence
AT1G72390	1.086241337	1.56E-25	Arabidopsis thaliana protein PHYTOCHROME-DEPENDENT LATE-FLOWERING mRNA, complete cds
AT1G14840	1.075824721	1.65E-25	Arabidopsis thaliana microtubule-associated protein 70-4 mRNA, complete cds
AT3G55160	1.469014987	1.72E-25	Arabidopsis thaliana uncharacterized protein mRNA, complete cds
AT3G47890	1.022634683	1.77E-25	Arabidopsis thaliana ubiquitin carboxyl-terminal hydrolase-related protein mRNA, complete cds
AT3G18110	1.099890398	1.93E-25	Arabidopsis thaliana pentatricopeptide repeat-containing protein mRNA, complete cds

AT1G08130	1.044349725	2.07E-25	Arabidopsis thaliana DNA ligase 1 mRNA, complete cds
AT1G05630	1.041998499	2.31E-25	Arabidopsis thaliana Type I inositol-1,4,5-trisphosphate 5-phosphatase 13 mRNA, complete cds
AT3G59140	1.3026648	3.37E-25	Arabidopsis thaliana multidrug resistance-associated protein 14 mRNA, complete cds
AT1G56650	2.69031266	3.97E-25	Arabidopsis thaliana transcription factor MYB75 mRNA, complete cds
AT5G13205	2.300685005	4.73E-25	Arabidopsis thaliana chromosome 5 sequence
AT3G49580	6.700951181	4.85E-25	Arabidopsis thaliana protein RESPONSE TO LOW SULFUR 1 mRNA, complete cds
AT1G10450	1.094508953	5.51E-25	Arabidopsis thaliana paired amphipathic helix protein Sin3-like 6 mRNA, complete cds
AT5G11610	2.162592223	6.73E-25	Arabidopsis thaliana Exostosin family protein mRNA, complete cds
AT2G39050	1.326227818	7.77E-25	Arabidopsis thaliana Euonymus lectin S3 mRNA, complete cds
AT5G62360	1.772382672	8.85E-25	Arabidopsis thaliana chromosome 5 sequence
AT5G11530	1.274850902	9.21E-25	Arabidopsis thaliana embryonic flower 1 mRNA, complete cds
AT2G29500	2.883254315	1.03E-24	Arabidopsis thaliana chromosome 2, complete sequence
AT3G46230	3.484790713	1.78E-24	Arabidopsis thaliana chromosome 3, complete sequence
AT1G36180	1.900022528	1.91E-24	Arabidopsis thaliana acetyl-CoA carboxylase 2 mRNA, complete cds
AT3G28030	1.534999454	2.18E-24	Arabidopsis thaliana DNA repair protein UVH3 mRNA, complete cds
AT4G25120	1.951791137	3.16E-24	Arabidopsis thaliana helicase SRS2-like protein mRNA, complete cds
AT1G76930	1.142094572	3.25E-24	Arabidopsis thaliana extensin 4 mRNA, complete cds
AT1G07910	1.346108464	4.45E-24	Arabidopsis thaliana tRNA ligase mRNA, complete cds
AT2G05440	2.101191678	4.55E-24	Arabidopsis thaliana glycine-rich protein 9 mRNA, complete cds
AT1G44900	1.077893994	4.59E-24	Arabidopsis thaliana minichromosome maintenance protein 2 mRNA, complete cds
AT1G52770	2.513662561	5.18E-24	Arabidopsis thaliana phototropic-responsive NPH3 family protein mRNA, complete cds
AT5G24470	1.569665811	5.59E-24	Arabidopsis thaliana pseudo-response regulator 5 mRNA, complete cds
AT5G55450	1.430195738	6.07E-24	Arabidopsis thaliana bifunctional inhibitor/lipid-transfer protein/seed storage 2S albumin-like protein mRNA, complete cds
AT4G11650	1.461139445	6.67E-24	Arabidopsis thaliana osmotin-like protein OSM34 mRNA, complete cds
AT1G32920	1.003500906	7.35E-24	Arabidopsis thaliana chromosome 1 sequence
AT3G25020	1.39760019	7.83E-24	Arabidopsis thaliana chromosome 3, complete sequence

AT3G01600	3.359259831	8.37E-24	Arabidopsis thaliana NAC domain containing protein 44 mRNA, complete cds
AT3G05380	1.116614986	1.10E-23	Arabidopsis thaliana protein ALWAYS EARLY 2 mRNA, complete cds
AT3G55240	3.800486854	1.12E-23	Arabidopsis thaliana uncharacterized protein mRNA, complete cds
AT5G24280	2.035867814	1.65E-23	Arabidopsis thaliana gamma-irradiation and mitomycin c induced 1 mRNA, complete cds
AT3G43920	1.63878236	1.97E-23	Arabidopsis thaliana endoribonuclease Dicer-like 3 mRNA, complete cds
AT1G51890	1.155345985	2.02E-23	Arabidopsis thaliana probable LRR receptor-like protein kinase mRNA, complete cds
AT3G12500	1.457396946	2.12E-23	Arabidopsis thaliana basic chitinase B mRNA, complete cds
AT3G54100	1.151829678	2.31E-23	Arabidopsis thaliana O-fucosyltransferase family protein mRNA, complete cds
AT2G43800	1.209252227	2.44E-23	Arabidopsis thaliana formin-like protein 2 mRNA, complete cds
AT2G29120	1.264578578	2.70E-23	Arabidopsis thaliana glutamate receptor 2.7 mRNA, complete cds
AT2G20870	1.97021754	3.13E-23	Arabidopsis thaliana chromosome 2, complete sequence
AT4G16680	1.380526677	3.64E-23	Arabidopsis thaliana putative RNA helicase mRNA, complete cds
AT5G42400	1.114438229	6.29E-23	Arabidopsis thaliana histone methyltransferase SDG25 mRNA, complete cds
AT5G28090	7.656096945	6.62E-23	Arabidopsis thaliana uncharacterized protein mRNA, complete cds
AT5G44635	1.281666711	7.72E-23	Arabidopsis thaliana minichromosome maintenance protein 6 mRNA, complete cds
AT4G32820	1.173704178	8.80E-23	Arabidopsis thaliana tetratricopeptide repeat domain-containing protein mRNA, complete cds
AT2G33435	1.518192219	9.15E-23	Arabidopsis thaliana RNA recognition motif-containing protein mRNA, complete cds
AT5G60040	1.632434267	9.79E-23	Arabidopsis thaliana nuclear RNA polymerase C1 mRNA, complete cds
AT4G36010	1.727057072	1.03E-22	Arabidopsis thaliana pathogenesis-related thaumatin family protein mRNA, complete cds
AT5G39790	1.509681277	1.15E-22	Arabidopsis thaliana putative starch binding scaffold protein mRNA, complete cds
AT2G19710	1.296694145	1.21E-22	Arabidopsis thaliana Vps4 regulator of MVB pathway mRNA, complete cds
AT5G38900	2.457079032	1.34E-22	Arabidopsis thaliana Thioredoxin superfamily protein mRNA, complete cds
AT5G52390	2.660711464	1.47E-22	Arabidopsis thaliana PAR1 protein mRNA, complete cds

AT3G21430	1.139680596	1.60E-22	Arabidopsis thaliana protein ALWAYS EARLY 3 mRNA, complete cds
AT2G32210	2.941293001	1.83E-22	Arabidopsis thaliana uncharacterized protein mRNA, complete cds
AT5G57550	3.730097527	2.35E-22	Arabidopsis thaliana probable xyloglucan endotransglucosylase/hydrolase protein 25 mRNA, complete cds
AT2G34390	3.47855876	2.80E-22	Arabidopsis thaliana aquaporin NIP2-1 mRNA, complete cds
AT2G44460	3.878799257	2.82E-22	Arabidopsis thaliana beta glucosidase 28 mRNA, complete cds
AT3G57240	3.588741677	3.52E-22	Arabidopsis thaliana beta-1,3-glucanase 3 mRNA, complete cds
AT3G12520	1.509922931	4.41E-22	Arabidopsis thaliana putative sulfate transporter 4.2 mRNA, complete cds
AT3G50280	1.938684148	6.11E-22	Arabidopsis thaliana chromosome 3, complete sequence
AT2G21130	1.42504325	7.19E-22	Arabidopsis thaliana chromosome 2, complete sequence
AT5G19310	1.284180714	9.36E-22	Arabidopsis thaliana homeotic protein regulator mRNA, complete cds
AT5G15960	7.561974768	1.02E-21	Arabidopsis thaliana cold and ABA inducible protein kin1 mRNA, complete cds
AT1G16090	1.563890813	1.28E-21	Arabidopsis thaliana wall associated kinase-like 7 mRNA, complete cds
AT1G77500	1.004711451	1.40E-21	Arabidopsis thaliana uncharacterized protein mRNA, complete cds
AT4G16740	7.545672955	1.61E-21	Arabidopsis thaliana tricyclene synthase mRNA, complete cds
AT3G53540	1.008836833	2.09E-21	Arabidopsis thaliana uncharacterized protein mRNA, complete cds
AT5G19110	1.155213479	2.24E-21	Arabidopsis thaliana Eukaryotic aspartyl protease family protein mRNA, complete cds
AT3G54150	2.6070735	2.37E-21	Arabidopsis thaliana S-adenosyl-L-methionine-dependent methyltransferase-like protein mRNA, complete cds
AT1G20720	2.077829097	4.00E-21	Arabidopsis thaliana RAD3-like DNA-binding helicase protein mRNA, complete cds
AT5G46740	1.423276324	5.87E-21	Arabidopsis thaliana ubiquitin-specific protease 21 mRNA, complete cds
AT2G36200	1.213560167	6.92E-21	Arabidopsis thaliana kinesin family protein mRNA, complete cds
AT1G33390	1.203664199	7.60E-21	Arabidopsis thaliana protein FASCIATED STEM 4 mRNA, complete cds
AT4G23540	1.265689112	8.45E-21	Arabidopsis thaliana ARM repeat superfamily protein mRNA, complete cds
AT2G20720	1.718509552	8.52E-21	Arabidopsis thaliana pentatricopeptide repeat-containing

			protein mRNA, complete cds
AT2G21640	1.950179787	9.84E-21	Arabidopsis thaliana uncharacterized oxidative stress response protein mRNA, complete cds
AT5G57010	1.842970186	1.07E-20	Arabidopsis thaliana calmodulin-binding family protein mRNA, complete cds
AT2G41730	3.113722073	1.40E-20	Arabidopsis thaliana chromosome 2, complete sequence
AT3G28510	3.113722073	1.40E-20	Arabidopsis thaliana chromosome 3, complete sequence
AT5G15700	1.72669895	1.77E-20	Arabidopsis thaliana DNA-directed RNA polymerase 2 mRNA, complete cds
AT3G61780	1.137903457	1.78E-20	Arabidopsis thaliana protein embryo defective 1703 mRNA, complete cds
AT4G27010	1.62795064	1.93E-20	Arabidopsis thaliana uncharacterized protein mRNA, complete cds
AT1G56660	1.107302952	2.16E-20	Arabidopsis thaliana uncharacterized protein mRNA, complete cds
AT1G30620	1.054392471	2.22E-20	Arabidopsis thaliana UDP-arabinose 4-epimerase 1 mRNA, complete cds
AT1G62310	1.025868719	2.35E-20	Arabidopsis thaliana transcription factor jumonji domain-containing protein mRNA, complete cds
AT1G24148	1.076234163	2.79E-20	Arabidopsis thaliana chromosome 1 sequence
AT4G23215	2.551364068	3.74E-20	Arabidopsis thaliana chromosome 4 sequence
AT5G10695	1.743353623	4.21E-20	Arabidopsis thaliana chromosome 5 sequence
AT5G22570	5.33416885	4.23E-20	Arabidopsis thaliana putative WRKY transcription factor 38 mRNA, complete cds
AT2G38530	1.00411457	4.32E-20	Arabidopsis thaliana non-specific lipid-transfer protein 2 mRNA, complete cds
AT4G23310	2.880657203	4.83E-20	Arabidopsis thaliana putative cysteine-rich receptor-like protein kinase 23 mRNA, complete cds
AT2G41835	1.426923392	6.95E-20	Arabidopsis thaliana zinc finger (C2H2 type, AN1-like) family protein mRNA, complete cds
AT3G60160	1.322941595	7.06E-20	Arabidopsis thaliana multidrug resistance-associated protein 9 mRNA, complete cds
AT4G02710	1.375747954	7.38E-20	Arabidopsis thaliana Kinase interacting (KIP1-like) family protein mRNA, complete cds
AT1G16800	1.713639104	7.74E-20	Arabidopsis thaliana P-loop containing nucleoside triphosphate hydrolases superfamily protein mRNA, complete cds
AT1G76955	4.768065377	8.03E-20	Arabidopsis thaliana uncharacterized protein mRNA, complete cds
AT2G46790	1.944222332	8.11E-20	Arabidopsis thaliana two-component response regulator-like APRR9 mRNA, complete cds
AT1G76600	1.19098217	8.25E-20	Arabidopsis thaliana chromosome 1 sequence
AT1G18370	1.201718554	9.25E-20	Arabidopsis thaliana kinesin HINKEL mRNA, complete cds
AT2G06925	1.144500735	9.64E-20	Arabidopsis thaliana phospholipase A2-alpha mRNA,

			complete cds
AT2G32400	1.089790404	1.33E-19	Arabidopsis thaliana glutamate receptor 5 mRNA, complete cds
AT1G34340	1.009402356	1.97E-19	Arabidopsis thaliana alpha/beta-hydrolase domain-containing protein mRNA, complete cds
AT1G28480	3.130635456	2.05E-19	Arabidopsis thaliana chromosome 1 sequence
AT1G70810	1.164243849	2.33E-19	Arabidopsis thaliana Calcium-dependent lipid-binding (CaLB domain) family protein mRNA, complete cds
AT4G22970	1.941043421	2.33E-19	Arabidopsis thaliana separase-like protein mRNA, complete cds
AT3G48350	1.29451265	2.48E-19	Arabidopsis thaliana KDEL-tailed cysteine endopeptidase CEP3 mRNA, complete cds
AT4G23280	4.730097527	2.68E-19	Arabidopsis thaliana putative cysteine-rich receptor-like protein kinase 20 mRNA, complete cds
AT5G14730	1.969427672	2.81E-19	Arabidopsis thaliana chromosome 5 sequence
AT1G13480	2.115192711	3.10E-19	Arabidopsis thaliana uncharacterized protein mRNA, complete cds
AT4G24610	1.137700936	3.44E-19	Arabidopsis thaliana uncharacterized protein mRNA, complete cds
AT1G01500	1.731957869	6.01E-19	Arabidopsis thaliana Erythronate-4-phosphate dehydrogenase-like protein mRNA, complete cds
AT4G08555	4.06809399	6.06E-19	Arabidopsis thaliana chromosome 4 sequence
AT2G40340	4.06809399	6.06E-19	Arabidopsis thaliana dehydration-responsive element-binding protein 2C mRNA, complete cds
AT5G67100	1.105199495	6.26E-19	Arabidopsis thaliana DNA polymerase alpha catalytic subunit mRNA, complete cds
AT1G22370	1.678363629	7.01E-19	Arabidopsis thaliana UDP-glucosyl transferase 85A5 mRNA, complete cds
AT5G49930	1.009957752	7.33E-19	Arabidopsis thaliana protein EMBRYO DEFECTIVE 1441 mRNA, complete cds
AT1G63860	1.329097861	8.52E-19	Arabidopsis thaliana TIR-NBS-LRR class disease resistance protein mRNA, complete cds
AT5G05410	1.408169432	8.56E-19	Arabidopsis thaliana dehydration-responsive element-binding protein 2A mRNA, complete cds
AT5G41100	1.221959347	9.32E-19	Arabidopsis thaliana uncharacterized protein mRNA, complete cds
AT1G65500	2.374486305	1.10E-18	Arabidopsis thaliana uncharacterized protein mRNA, complete cds
AT3G47480	2.256166338	1.11E-18	Arabidopsis thaliana chromosome 3, complete sequence
AT5G40820	2.256166338	1.11E-18	Arabidopsis thaliana serine/threonine-protein kinase ATR mRNA, complete cds
AT1G02450	4.049715461	1.12E-18	Arabidopsis thaliana chromosome 1 sequence
AT5G28464	7.295694702	1.13E-18	Arabidopsis thaliana chromosome 5 sequence
AT1G34420	2.086241337	1.39E-18	Arabidopsis thaliana leucine-rich repeat transmembrane

			protein kinase-like protein mRNA, complete cds
AT3G09405	1.750492747	1.43E-18	Arabidopsis thaliana pectinacetylesterase family protein mRNA, complete cds
AT1G09932	1.144455951	1.53E-18	Arabidopsis thaliana phosphoglycerate mutase family protein mRNA, complete cds
AT5G08230	1.200983423	1.68E-18	Arabidopsis thaliana Tudor/PWWP/MBT domain-containing protein mRNA, complete cds
AT4G09750	1.769051161	1.87E-18	Arabidopsis thaliana mRNA for hypothetical protein, complete cds, clone: RAFL14-08-C18
AT5G64750	1.934238243	1.92E-18	Arabidopsis thaliana ethylene-responsive transcription factor ABR1 mRNA, complete cds
AT4G13750	1.733939593	1.95E-18	Arabidopsis thaliana nuclear factor NO VEIN mRNA, complete cds
AT1G35660	1.437916775	2.55E-18	Arabidopsis thaliana uncharacterized protein mRNA, complete cds
AT2G13810	5.194765794	2.58E-18	Arabidopsis thaliana AGD2-like defense response protein 1 mRNA, complete cds
AT5G01790	1.820429846	3.58E-18	Arabidopsis thaliana chromosome 5 sequence
AT5G44430	6.173704178	3.59E-18	Arabidopsis thaliana defensin-like protein mRNA, complete cds
AT5G28400	1.83166851	4.04E-18	Arabidopsis thaliana uncharacterized protein mRNA, complete cds
AT5G57160	1.082419626	4.33E-18	Arabidopsis thaliana DNA ligase 4 mRNA, complete cds
AT4G34900	1.229395109	4.62E-18	Arabidopsis thaliana xanthine dehydrogenase 2 mRNA, complete cds
AT2G39920	2.002717981	4.91E-18	Arabidopsis thaliana acid phosphatase class IIIB protein mRNA, complete cds
AT1G74710	1.02425445	5.52E-18	Arabidopsis thaliana Isochorismate synthase 1 mRNA, complete cds
AT1G67970	1.429275065	6.75E-18	Arabidopsis thaliana heat stress transcription factor A-8 mRNA, complete cds
AT1G35230	1.34167402	7.06E-18	Arabidopsis thaliana chromosome 1 sequence
AT5G61460	1.472299769	7.36E-18	Arabidopsis thaliana protein SMC6B mRNA, complete cds
AT3G01970	2.514102876	7.81E-18	Arabidopsis thaliana WRKY DNA-binding protein 45 mRNA, complete cds
AT5G47330	2.758666679	8.27E-18	Arabidopsis thaliana palmitoyl protein thioesterase family protein mRNA, complete cds
AT3G18500	1.067332874	8.40E-18	Arabidopsis thaliana DNAse I-like superfamily protein mRNA, complete cds
AT1G21400	1.261167019	9.95E-18	Arabidopsis thaliana thiamin diphosphate-binding fold protein mRNA, complete cds
AT5G12020	2.878799257	1.06E-17	Arabidopsis thaliana chromosome 5 sequence
AT5G62480	1.254923171	1.17E-17	Arabidopsis thaliana glutathione S-transferase tau 9 mRNA, complete cds

AT2G47520	7.194765794	1.25E-17	Arabidopsis thaliana ethylene-responsive transcription factor ERF071 mRNA, complete cds
AT4G04540	5.130635456	1.52E-17	Arabidopsis thaliana putative cysteine-rich receptor-like protein kinase 39 mRNA, complete cds
AT2G27150	1.016532365	1.52E-17	Arabidopsis thaliana abscisic-aldehyde oxidase mRNA, complete cds
AT4G23130	1.492561619	1.80E-17	Arabidopsis thaliana cysteine-rich receptor-like protein kinase 5 mRNA, complete cds
AT4G23990	1.306840476	2.09E-17	Arabidopsis thaliana cellulose synthase-like protein G3 mRNA, complete cds
AT5G65900	1.015506825	2.80E-17	Arabidopsis thaliana DEAD-box ATP-dependent RNA helicase 27 mRNA, complete cds
AT3G25882	4.215524354	2.82E-17	Arabidopsis thaliana chromosome 3, complete sequence
AT2G43590	1.037836705	3.72E-17	Arabidopsis thaliana chitinase family protein mRNA, complete cds
AT2G34780	1.166806818	4.78E-17	Arabidopsis thaliana protein MATERNAL EFFECT EMBRYO ARREST 22 mRNA, complete cds
AT1G47395	1.387195303	4.80E-17	Arabidopsis thaliana chromosome 1 sequence
AT5G23990	1.759200319	4.86E-17	Arabidopsis thaliana ferric reduction oxidase 5 mRNA, complete cds
AT5G55150	5.086241337	4.99E-17	Arabidopsis thaliana uncharacterized protein mRNA, complete cds
AT3G05660	2.833475266	5.94E-17	Arabidopsis thaliana receptor like protein 33 mRNA, complete cds
AT2G38240	2.833475266	5.94E-17	Arabidopsis thaliana 2-oxoglutarate (2OG) and Fe(II)-dependent oxygenase-like protein mRNA, complete cds
AT5G22300	2.006070988	7.23E-17	Arabidopsis thaliana bifunctional nitrilase/nitrile hydratase NIT4 mRNA, complete cds
AT2G22610	1.496706106	8.27E-17	Arabidopsis thaliana Di-glucose binding protein with Kinesin motor domain mRNA, complete cds
AT5G17220	1.437713707	9.65E-17	Arabidopsis thaliana glutathione S-transferase phi 12 mRNA, complete cds
AT4G18270	1.324105167	1.21E-16	Arabidopsis thaliana phospho-N-acetylmuramoyl-pentapeptide-transferase-like protein mRNA, complete cds
AT5G43870	1.086241337	1.22E-16	Arabidopsis thaliana uncharacterized protein mRNA, complete cds
AT3G58160	1.489819721	1.24E-16	Arabidopsis thaliana Class XI myosin mRNA, complete cds
AT1G16030	1.474311789	1.30E-16	Arabidopsis thaliana chromosome 1 sequence
AT1G79190	1.025487306	1.72E-16	Arabidopsis thaliana uncharacterized protein mRNA, complete cds
AT2G16060	1.10860915	2.27E-16	Arabidopsis thaliana non-symbiotic hemoglobin 1 mRNA, complete cds

AT4G13800	2.944222332	2.31E-16	Arabidopsis thaliana uncharacterized protein mRNA, complete cds
AT5G07660	1.924184579	2.33E-16	Arabidopsis thaliana structural maintenance of chromosomes 6A mRNA, complete cds
AT4G14090	1.29356631	2.43E-16	Arabidopsis thaliana chromosome 4 sequence
AT2G02480	1.113377467	2.45E-16	Arabidopsis thaliana protein STICHEL mRNA, complete cds
AT1G48120	1.428160907	2.55E-16	Arabidopsis thaliana serine/threonine-protein phosphatase 7 long form homolog mRNA, complete cds
AT3G10180	2.308633758	3.66E-16	Arabidopsis thaliana kinesin motor protein-related protein mRNA, complete cds
AT1G29715	4.47855876	3.93E-16	Arabidopsis thaliana mRNA for hypothetical protein, complete cds, clone: RAFL21-67-K19
AT4G26600	1.162057119	5.82E-16	Arabidopsis thaliana S-adenosyl-L-methionine-dependent methyltransferase-like protein mRNA, complete cds
AT3G14460	2.262564109	6.14E-16	Arabidopsis thaliana chromosome 3, complete sequence
AT1G72250	1.213214193	6.28E-16	Arabidopsis thaliana Di-glucose binding protein with Kinesin motor domain mRNA, complete cds
AT5G66310	1.099873202	6.52E-16	Arabidopsis thaliana ATP binding microtubule motor family protein mRNA, complete cds
AT4G21940	1.936345906	7.42E-16	Arabidopsis thaliana calcium-dependent protein kinase 15 mRNA, complete cds
AT2G36480	1.086241337	9.33E-16	Arabidopsis thaliana ENTH/VHS-like protein mRNA, complete cds
AT4G01020	1.360863717	1.02E-15	Arabidopsis thaliana zinc finger-related and helicase and IBR domain-containing protein mRNA, complete cds
AT3G14980	1.044662233	1.04E-15	Arabidopsis thaliana histone H3 acetyltransferase IDM1 mRNA, complete cds
AT3G57460	1.415023103	1.20E-15	Arabidopsis thaliana catalytic/ metal ion binding / metalloendopeptidase/ zinc ion binding protein mRNA, complete cds
AT4G12735	4.432016174	1.33E-15	Arabidopsis thaliana chromosome 4 sequence
AT5G28290	1.072820821	1.99E-15	Arabidopsis thaliana serine/threonine-protein kinase Nek3 mRNA, complete cds
AT2G36350	1.211772219	1.99E-15	Arabidopsis thaliana protein kinase mRNA, complete cds
AT4G33950	1.115387682	2.30E-15	Arabidopsis thaliana calcium-independent ABA-activated protein kinase mRNA, complete cds
AT2G19190	2.876318267	2.35E-15	Arabidopsis thaliana FLG22-induced receptor-like kinase 1 mRNA, complete cds
AT1G04160	1.243342298	2.41E-15	Arabidopsis thaliana myosin XI B mRNA, complete cds
AT5G57123	2.553367347	2.49E-15	Arabidopsis thaliana chromosome 5 sequence
AT1G23730	1.612989224	2.50E-15	Arabidopsis thaliana beta carbonic anhydrase 3 mRNA, complete cds
AT3G16565	1.154083983	2.57E-15	Arabidopsis thaliana threonyl and alanyl tRNA synthetase

			second additional domain-containing protein mRNA, complete cds
AT3G29590	1.968185575	2.59E-15	Arabidopsis thaliana chromosome 3, complete sequence
AT5G06730	2.050908264	3.59E-15	Arabidopsis thaliana peroxidase 54 mRNA, complete cds
AT2G15820	1.013074924	3.62E-15	Arabidopsis thaliana organelle transcript processing 51 mRNA, complete cds
AT4G04500	4.040437647	3.83E-15	Arabidopsis thaliana cysteine-rich receptor-like protein kinase 37 mRNA, complete cds
AT4G09500	1.709171688	4.19E-15	Arabidopsis thaliana chromosome 4 sequence
AT3G21540	1.008899248	4.90E-15	Arabidopsis thaliana transducin/WD40 domain-containing protein mRNA, complete cds
AT1G31810	1.181893576	6.14E-15	Arabidopsis thaliana formin-like protein 14 mRNA, complete cds
AT2G29470	2.930590466	6.17E-15	Arabidopsis thaliana glutathione S-transferase tau 3 mRNA, complete cds
AT1G53625	4.016978674	7.07E-15	Arabidopsis thaliana chromosome 1 sequence
AT3G10530	1.127464	7.43E-15	Arabidopsis thaliana transducin/WD40 domain-containing protein mRNA, complete cds
AT2G32140	1.580230177	7.61E-15	Arabidopsis thaliana transmembrane receptor protein mRNA, complete cds
AT1G62970	1.219135607	8.13E-15	Arabidopsis thaliana chromosome 1 sequence
AT3G45830	1.003779177	9.95E-15	Arabidopsis thaliana uncharacterized protein mRNA, complete cds
AT1G80130	1.313012199	1.17E-14	Arabidopsis thaliana tetratricopeptide repeat domain-containing protein mRNA, complete cds
AT1G76740	2.561974768	1.21E-14	Arabidopsis thaliana uncharacterized protein mRNA, complete cds
AT1G75960	1.754259577	1.33E-14	Arabidopsis thaliana chromosome 1 sequence
AT2G14610	2.993131932	1.60E-14	Arabidopsis thaliana chromosome 2, complete sequence
AT1G17600	1.329097861	1.76E-14	Arabidopsis thaliana TIR-NBS-LRR class disease resistance protein mRNA, complete cds
AT1G08840	1.541435963	1.91E-14	Arabidopsis thaliana embryo defective protein 2411 mRNA, complete cds
AT2G40030	1.547821422	2.26E-14	Arabidopsis thaliana nuclear RNA polymerase D1B mRNA, complete cds
AT2G34090	1.233517599	2.33E-14	Arabidopsis thaliana maternal effect embryo arrest 18 protein mRNA, complete cds
AT2G29490	1.680984858	2.85E-14	Arabidopsis thaliana glutathione S-transferase tau 1 mRNA, complete cds
AT2G25440	4.308633758	2.86E-14	Arabidopsis thaliana receptor like protein 20 mRNA, complete cds
AT5G50170	1.313156317	3.06E-14	Arabidopsis thaliana C2 and GRAM domain-containing protein mRNA, complete cds
AT5G59310	3.194765794	3.16E-14	Arabidopsis thaliana non-specific lipid-transfer protein 4

			mRNA, complete cds
AT1G61800	2.874737232	3.51E-14	Arabidopsis thaliana glucose-6-phosphate/phosphate translocator 2 mRNA, complete cds
AT1G66600	2.874737232	3.51E-14	Arabidopsis thaliana putative WRKY transcription factor 63 mRNA, complete cds
AT5G52410	1.211772219	3.66E-14	Arabidopsis thaliana uncharacterized protein mRNA, complete cds
AT4G22880	1.02753203	3.71E-14	Arabidopsis thaliana leucoanthocyanidin dioxygenase mRNA, complete cds
AT5G28237	2.156630665	3.80E-14	Arabidopsis thaliana tryptophan synthase beta chain-like protein mRNA, complete cds
AT3G57060	1.136089886	4.08E-14	Arabidopsis thaliana putative condensin complex protein Cap-D2 mRNA, complete cds
AT5G27240	1.284570053	4.20E-14	Arabidopsis thaliana chromosome 5 sequence
AT1G61420	1.575279418	4.46E-14	Arabidopsis thaliana G-type lectin S-receptor-like serine/threonine-protein kinase mRNA, complete cds
AT1G63240	1.057289963	4.83E-14	Arabidopsis thaliana uncharacterized protein mRNA, complete cds
AT5G20740	1.155503999	5.05E-14	Arabidopsis thaliana chromosome 5 sequence
AT3G18730	1.681187926	5.15E-14	Arabidopsis thaliana protein BRUSHY 1 mRNA, complete cds
AT2G31230	1.448811416	5.27E-14	Arabidopsis thaliana chromosome 2, complete sequence
AT1G52890	2.086241337	5.66E-14	Arabidopsis thaliana NAC domain-containing protein 19 mRNA, complete cds
AT5G37940	6.786681055	5.78E-14	Arabidopsis thaliana zinc-binding dehydrogenase family protein mRNA, complete cds
AT1G43160	1.490138278	5.83E-14	Arabidopsis thaliana ethylene-responsive transcription factor RAP2-6 mRNA, complete cds
AT5G47220	1.061663314	6.49E-14	Arabidopsis thaliana chromosome 5 sequence
AT1G65470	1.143272282	6.59E-14	Arabidopsis thaliana chromatin assembly factor 1 subunit FAS1 mRNA, complete cds
AT4G39830	1.327902092	9.87E-14	Arabidopsis thaliana putative L-ascorbate oxidase mRNA, complete cds
AT1G63300	1.262830069	1.02E-13	Arabidopsis thaliana Myosin heavy chain-related protein mRNA, complete cds
AT3G44050	1.294827959	1.13E-13	Arabidopsis thaliana kinesin motor protein-like protein mRNA, complete cds
AT2G46980	1.432691751	1.17E-13	Arabidopsis thaliana ASYNAPTIC 3 mRNA, complete cds
AT1G16420	2.346108464	1.20E-13	Arabidopsis thaliana metacaspase 8 mRNA, complete cds
AT1G75150	1.661150173	1.24E-13	Arabidopsis thaliana uncharacterized protein mRNA, complete cds
AT4G17610	1.086241337	1.24E-13	Arabidopsis thaliana tRNA/rRNA methyltransferase (SpoU) family protein mRNA, complete cds
AT4G16960	1.47326446	1.31E-13	Arabidopsis thaliana TIR-NBS-LRR class disease resistance

			protein mRNA, complete cds
AT2G35340	1.236801013	1.35E-13	Arabidopsis thaliana protein MATERNAL EFFECT EMBRYO ARREST 29 mRNA, complete cds
AT3G20440	1.195539979	1.56E-13	Arabidopsis thaliana putative glycoside hydrolase mRNA, complete cds
AT3G16360	4.229199291	1.81E-13	Arabidopsis thaliana histidine-containing phosphotransfer protein 4 mRNA, complete cds
AT4G21680	1.562269466	2.21E-13	Arabidopsis thaliana nitrate transporter 1.8 mRNA, complete cds
AT5G40500	1.046163897	2.51E-13	Arabidopsis thaliana uncharacterized protein mRNA, complete cds
AT1G06490	1.223744861	2.74E-13	Arabidopsis thaliana callose synthase 7 mRNA, complete cds
AT5G20480	1.778119041	2.91E-13	Arabidopsis thaliana LRR receptor-like serine/threonine-protein kinase EFR mRNA, complete cds
AT1G18870	1.983147844	3.51E-13	Arabidopsis thaliana Isochorismate synthase 2 mRNA, complete cds
AT4G29940	1.133938079	3.56E-13	Arabidopsis thaliana Pathogenesis-related homeodomain protein mRNA, complete cds
AT4G00930	1.296601203	3.59E-13	Arabidopsis thaliana COP1-interacting protein 4.1 mRNA, complete cds
AT1G51780	6.671203838	4.53E-13	Arabidopsis thaliana IAA-amino acid hydrolase ILR1-like 5 mRNA, complete cds
AT2G47190	3.841128839	5.24E-13	Arabidopsis thaliana mRNA for MYB transcription factor, complete cds, clone: RAFL16-42-L19
AT2G31320	1.103450627	5.44E-13	Arabidopsis thaliana poly [ADP-ribose] polymerase 2 mRNA, complete cds
AT3G22910	2.391095918	5.77E-13	Arabidopsis thaliana chromosome 3, complete sequence
AT2G34500	1.049715461	6.23E-13	Arabidopsis thaliana chromosome 2, complete sequence
AT5G64810	1.993131932	6.25E-13	Arabidopsis thaliana putative WRKY transcription factor 51 mRNA, complete cds
AT2G36770	2.691103395	7.11E-13	Arabidopsis thaliana chromosome 2, complete sequence
AT1G33960	2.691103395	7.11E-13	Arabidopsis thaliana protein AIG1 mRNA, complete cds
AT1G06000	1.086241337	7.79E-13	Arabidopsis thaliana chromosome 1 sequence
AT3G02930	1.272654461	8.82E-13	Arabidopsis thaliana uncharacterized protein mRNA, complete cds
AT2G42200	1.47397149	1.09E-12	Arabidopsis thaliana squamosa promoter-binding-like protein 9 mRNA, complete cds
AT5G65280	1.110088079	1.09E-12	Arabidopsis thaliana protein GCR2-like 1 mRNA, complete cds
AT1G52450	2.671203838	1.25E-12	Arabidopsis thaliana ubiquitin carboxyl-terminal hydrolase-related protein mRNA, complete cds
AT4G15620	1.053575148	1.55E-12	Arabidopsis thaliana uncharacterized protein mRNA, complete cds

AT1G64660	1.006624169	1.60E-12	Arabidopsis thaliana methionine gamma-lyase mRNA, complete cds
AT4G02360	2.3563305	1.68E-12	Arabidopsis thaliana chromosome 4 sequence
AT4G02110	1.408169432	1.76E-12	Arabidopsis thaliana transcription coactivator protein mRNA, complete cds
AT1G25370	1.200574012	2.04E-12	Arabidopsis thaliana uncharacterized protein mRNA, complete cds
AT5G15920	1.152129272	2.10E-12	Arabidopsis thaliana structural maintenance of chromosomes 5 mRNA, complete cds
AT5G24380	1.001499137	2.17E-12	Arabidopsis thaliana metal-nicotianamine transporter YSL2 mRNA, complete cds
AT1G03830	1.362758972	2.17E-12	Arabidopsis thaliana guanylate-binding protein mRNA, complete cds
AT4G18490	2.575626178	2.41E-12	Arabidopsis thaliana uncharacterized protein mRNA, complete cds
AT1G80810	1.201718554	2.50E-12	Arabidopsis thaliana Tudor/PWWP/MBT superfamily protein mRNA, complete cds
AT1G26730	1.131029259	2.54E-12	Arabidopsis thaliana phosphate transporter PHO1-7 mRNA, complete cds
AT5G46400	1.052818335	2.86E-12	Arabidopsis thaliana pre-mRNA-processing factor 39 mRNA, complete cds
AT3G44730	1.117811416	3.27E-12	Arabidopsis thaliana kinesin-like protein 1 mRNA, complete cds
AT4G23230	1.466665081	3.51E-12	Arabidopsis thaliana cysteine-rich receptor-like protein kinase 15 mRNA, complete cds
AT3G24090	1.287875198	3.54E-12	Arabidopsis thaliana putative glucosamine-fructose-6-phosphate aminotransferase mRNA, complete cds
AT1G12210	1.747152691	3.71E-12	Arabidopsis thaliana chromosome 1 sequence
AT1G01010	1.153985943	3.87E-12	Arabidopsis thaliana NAC domain-containing protein 1 mRNA, complete cds
AT4G21070	1.303270573	4.43E-12	Arabidopsis thaliana protein BREAST CANCER SUSCEPTIBILITY 1-like protein mRNA, complete cds
AT5G04895	1.303270573	4.43E-12	Arabidopsis thaliana DEA(D/H)-box RNA helicase family protein mRNA, complete cds
AT5G15500	2.153355533	4.47E-12	Arabidopsis thaliana chromosome 5 sequence
AT2G33440	1.47855876	5.04E-12	Arabidopsis thaliana RNA recognition motif-containing protein mRNA, complete cds
AT5G37630	1.105100364	5.15E-12	Arabidopsis thaliana protein EMBRYO DEFECTIVE 2656 mRNA, complete cds
AT5G24110	1.235754878	5.34E-12	Arabidopsis thaliana WRKY DNA-binding protein 30 mRNA, complete cds
AT2G32390	1.246706009	5.58E-12	Arabidopsis thaliana glutamate receptor 3.5 mRNA, complete cds

AT5G50450	1.111776429	5.58E-12	Arabidopsis thaliana MYND-type zinc finger protein mRNA, complete cds
AT3G60120	3.730097527	6.12E-12	Arabidopsis thaliana beta glucosidase 27 mRNA, complete cds
AT2G29170	3.28263855	6.94E-12	Arabidopsis thaliana NAD(P)-binding Rossmann-fold superfamily protein mRNA, complete cds
AT1G71210	1.086241337	7.39E-12	Arabidopsis thaliana chromosome 1 sequence
AT1G76650	1.447428862	7.83E-12	Arabidopsis thaliana chromosome 1 sequence
AT3G57680	2.213353255	8.04E-12	Arabidopsis thaliana peptidase S41 family protein mRNA, complete cds
AT4G14370	1.427278255	8.04E-12	Arabidopsis thaliana TIR-NBS-LRR class disease resistance protein mRNA, complete cds
AT5G48310	1.092500327	8.05E-12	Arabidopsis thaliana uncharacterized protein mRNA, complete cds
AT1G69930	4.545672955	8.77E-12	Arabidopsis thaliana glutathione S-transferase TAU 11 mRNA, complete cds
AT5G14580	1.219266755	8.87E-12	Arabidopsis thaliana polyribonucleotide nucleotidyltransferase mRNA, complete cds
AT1G35140	1.314269293	9.73E-12	Arabidopsis thaliana chromosome 1 sequence
AT5G22110	1.32881164	9.90E-12	Arabidopsis thaliana DNA polymerase epsilon subunit B2 mRNA, complete cds
AT2G31340	1.210420748	1.03E-11	Arabidopsis thaliana protein embryo defective 1381 mRNA, complete cds
AT2G24210	6.47855876	1.05E-11	Arabidopsis thaliana terpene synthase 10 mRNA, complete cds
AT1G67810	1.165808594	1.15E-11	Arabidopsis thaliana chromosome 1 sequence
AT1G31173	2.588741677	1.18E-11	Arabidopsis thaliana chromosome 1 sequence
AT1G20390	1.832484745	1.29E-11	Arabidopsis thaliana chromosome 1 sequence
AT1G10270	1.056370807	1.31E-11	Arabidopsis thaliana chromosome 1 sequence
AT1G76470	4.024840792	1.35E-11	Arabidopsis thaliana NAD(P)-binding Rossmann-fold superfamily protein mRNA, complete cds
AT3G55060	1.545672955	1.37E-11	Arabidopsis thaliana uncharacterized protein mRNA, complete cds
AT3G22160	1.193156541	1.38E-11	Arabidopsis thaliana chromosome 3, complete sequence
AT5G44820	1.256166338	1.52E-11	Arabidopsis thaliana Nucleotide-diphospho-sugar transferase family protein mRNA, complete cds
AT4G25200	6.443793341	1.79E-11	Arabidopsis thaliana small heat shock protein 23.6 mRNA, complete cds
AT1G80110	1.800486854	1.81E-11	Arabidopsis thaliana phloem protein 2-B11 mRNA, complete cds
AT4G14970	1.501278836	1.86E-11	Arabidopsis thaliana uncharacterized protein mRNA, complete cds
AT1G09530	1.050021149	2.21E-11	Arabidopsis thaliana transcription factor PIF3 mRNA, complete cds

AT2G18660	1.94039047	2.26E-11	Arabidopsis thaliana plant natriuretic peptide A mRNA, complete cds
AT1G16850	1.571668164	2.29E-11	Arabidopsis thaliana uncharacterized protein mRNA, complete cds
AT1G71000	3.993131932	2.51E-11	Arabidopsis thaliana chaperone DnaJ-domain containing protein mRNA, complete cds
AT4G32700	1.169134345	2.59E-11	Arabidopsis thaliana MUS308 and mammalian DNA polymerase-like protein mRNA, complete cds
AT1G66370	5.408169432	2.60E-11	Arabidopsis thaliana transcription factor MYB113 mRNA, complete cds
AT5G52230	1.302816432	3.04E-11	Arabidopsis thaliana methyl-CPG-binding domain protein 13 mRNA, complete cds
AT1G23550	1.804470368	3.28E-11	Arabidopsis thaliana probable inactive poly [ADP-ribose] polymerase SRO2 mRNA, complete cds
AT1G67920	1.92496043	3.65E-11	Arabidopsis thaliana chromosome 1 sequence
AT1G33030	2.919131351	3.76E-11	Arabidopsis thaliana O-methyltransferase-like protein mRNA, complete cds
AT1G63020	1.408169432	3.82E-11	Arabidopsis thaliana DNA-directed RNA polymerase IV subunit 1 mRNA, complete cds
AT5G10370	1.336467966	3.83E-11	Arabidopsis thaliana helicase , IBR and zinc finger protein domain-containing protein mRNA, complete cds
AT3G21480	1.145135026	3.89E-11	Arabidopsis thaliana BRCT domain-containing DNA repair protein mRNA, complete cds
AT3G05130	1.456352965	4.38E-11	Arabidopsis thaliana chromosome 3, complete sequence
AT2G19120	1.658492786	4.65E-11	Arabidopsis thaliana P-loop containing nucleoside triphosphate hydrolases superfamily protein mRNA, complete cds
AT5G38910	5.371643556	4.68E-11	Arabidopsis thaliana putative germin-like protein subfamily 1 member 9 mRNA, complete cds
AT5G28320	1.512506092	4.78E-11	Arabidopsis thaliana uncharacterized protein mRNA, complete cds
AT5G39580	1.256166338	4.86E-11	Arabidopsis thaliana peroxidase 62 mRNA, complete cds
AT2G42340	6.371643556	5.24E-11	Arabidopsis thaliana uncharacterized protein mRNA, complete cds
AT5G16930	1.191358043	5.40E-11	Arabidopsis thaliana AAA-type ATPase family protein mRNA, complete cds
AT1G65920	1.311661451	5.49E-11	Arabidopsis thaliana Regulator of chromosome condensation (RCC1) family with FYVE zinc finger domain mRNA, complete cds
AT4G16970	1.34362918	5.66E-11	Arabidopsis thaliana protein kinase superfamily protein mRNA, complete cds
AT5G40010	1.960710455	7.21E-11	Arabidopsis thaliana chromosome 5 sequence
AT5G49015	2.208231861	1.14E-10	Arabidopsis thaliana uncharacterized protein mRNA, complete cds

AT3G18524	1.093296094	1.15E-10	Arabidopsis thaliana DNA mismatch repair protein Msh2 mRNA, complete cds
AT4G24415	1.49743677	1.30E-10	Arabidopsis thaliana AT4g24411 mRNA, complete cds
AT2G14620	1.699218214	1.33E-10	Arabidopsis thaliana xyloglucan endotransglucosylase/hydrolase protein 10 mRNA, complete cds
AT1G34355	1.564288634	1.36E-10	Arabidopsis thaliana parallel Spindle 1 protein mRNA, complete cds
AT4G18280	1.281497628	1.37E-10	Arabidopsis thaliana chromosome 4 sequence
AT5G28030	1.04707074	1.39E-10	Arabidopsis thaliana bifunctional cystathionine gamma-lyase/cysteine synthase mRNA, complete cds
AT5G48610	1.02377725	1.42E-10	Arabidopsis thaliana uncharacterized protein mRNA, complete cds
AT4G15200	6.295694702	1.54E-10	Arabidopsis thaliana formin 3 mRNA, complete cds
AT1G79680	2.646956291	1.60E-10	Arabidopsis thaliana wall-associated receptor kinase-like 10 mRNA, complete cds
AT4G22470	3.893596259	1.60E-10	Arabidopsis thaliana chromosome 4 sequence
AT3G09270	1.331717371	1.78E-10	Arabidopsis thaliana glutathione S-transferase TAU 8 mRNA, complete cds
AT4G11911	4.371643556	1.90E-10	Arabidopsis thaliana uncharacterized protein mRNA, complete cds
AT3G47460	1.028653845	1.92E-10	Arabidopsis thaliana structural maintenance of chromosomes protein 2-2 mRNA, complete cds
AT2G40435	2.408169432	1.95E-10	Arabidopsis thaliana uncharacterized protein mRNA, complete cds
AT2G23910	1.272337629	1.97E-10	Arabidopsis thaliana Rossmann-fold NAD(P)-binding domain-containing protein mRNA, complete cds
AT5G27610	1.321869585	2.60E-10	Arabidopsis thaliana protein ALWAYS EARLY 1 mRNA, complete cds
AT1G49870	1.160666178	2.62E-10	Arabidopsis thaliana uncharacterized protein mRNA, complete cds
AT1G25175	6.256166338	2.66E-10	Arabidopsis thaliana At1g25170/F4F7_2 mRNA, complete cds
AT1G69920	5.256166338	2.76E-10	Arabidopsis thaliana glutathione S-transferase TAU 12 mRNA, complete cds
AT4G34060	1.802448371	3.07E-10	Arabidopsis thaliana DEMETER-like protein 3 mRNA, complete cds
AT2G47780	2.323280534	3.40E-10	Arabidopsis thaliana Rubber elongation factor protein mRNA, complete cds
AT5G18290	1.630561853	3.66E-10	Arabidopsis thaliana putative aquaporin SIP1-2 mRNA, complete cds
AT5G18370	1.172656089	4.22E-10	Arabidopsis thaliana TIR-NBS-LRR class disease resistance protein mRNA, complete cds
AT4G11170	1.643458847	4.29E-10	Arabidopsis thaliana putative disease resistance protein

			mRNA, complete cds
AT1G13750	1.014902555	4.38E-10	Arabidopsis thaliana Purple acid phosphatases superfamily protein mRNA, complete cds
AT2G45760	2.363081542	5.74E-10	Arabidopsis thaliana chromosome 2, complete sequence
AT3G07520	1.118302546	6.21E-10	Arabidopsis thaliana glutamate receptor 1.4 mRNA, complete cds
AT1G14640	1.050792501	6.44E-10	Arabidopsis thaliana SWAP (Suppressor-of-White-APricot)/surp domain-containing protein mRNA, complete cds
AT2G32190	2.012240755	7.30E-10	Arabidopsis thaliana uncharacterized protein mRNA, complete cds
AT2G29090	2.012240755	7.30E-10	Arabidopsis thaliana abscisic acid 8'-hydroxylase 2 mRNA, complete cds
AT3G13950	2.012240755	7.30E-10	Arabidopsis thaliana uncharacterized protein mRNA, complete cds
AT5G39120	1.642634685	7.79E-10	Arabidopsis thaliana germin-like protein subfamily 1 member 15 mRNA, complete cds
AT2G40740	2.671203838	7.82E-10	Arabidopsis thaliana WRKY transcription factor 55 mRNA, complete cds
AT1G26420	1.55572662	8.04E-10	Arabidopsis thaliana chromosome 1 sequence
AT3G46370	1.012924659	8.09E-10	Arabidopsis thaliana leucine-rich repeat protein kinase-like protein mRNA, complete cds
AT1G11950	1.187041978	8.28E-10	Arabidopsis thaliana transcription factor jumonji (jmjC) domain-containing protein mRNA, complete cds
AT2G45460	1.198716066	8.79E-10	Arabidopsis thaliana SMAD/FHA domain-containing protein mRNA, complete cds
AT5G44050	1.13686741	8.89E-10	Arabidopsis thaliana MATE efflux family protein mRNA, complete cds
AT1G69610	1.078628153	9.29E-10	Arabidopsis thaliana uncharacterized protein mRNA, complete cds
AT5G38120	1.078628153	9.29E-10	Arabidopsis thaliana 4-coumarate--CoA ligase-like 8 mRNA, complete cds
AT4G28460	3.786681055	1.03E-09	Arabidopsis thaliana chromosome 4 sequence
AT1G12805	2.758666679	1.22E-09	Arabidopsis thaliana chromosome 1 sequence
AT5G36910	4.256166338	1.22E-09	Arabidopsis thaliana thionin 2.2 mRNA, complete cds
AT3G55970	1.284787016	1.41E-09	Arabidopsis thaliana jasmonate-regulated protein JRG21 mRNA, complete cds
AT3G56970	1.035867814	1.48E-09	Arabidopsis thaliana transcription factor ORG2 mRNA, complete cds
AT1G77620	1.010833934	1.50E-09	Arabidopsis thaliana P-loop containing nucleoside triphosphate hydrolases superfamily protein mRNA, complete cds
AT1G47890	3.443793341	1.53E-09	Arabidopsis thaliana chromosome 1 sequence
AT1G33430	5.130635456	1.66E-09	Arabidopsis thaliana putative

			beta-1,3-galactosyltransferase 8 mRNA, complete cds
AT1G60930	1.216121619	1.71E-09	Arabidopsis thaliana RECQ helicase L4B mRNA, complete cds
AT4G14020	1.180568719	1.76E-09	Arabidopsis thaliana chromosome 4 sequence
AT1G10060	1.192440741	1.87E-09	Arabidopsis thaliana branched-chain-amino-acid aminotransferase 1 mRNA, complete cds
AT4G33980	1.003779177	2.03E-09	Arabidopsis thaliana uncharacterized protein mRNA, complete cds
AT3G11020	2.43416464	2.76E-09	Arabidopsis thaliana dehydration-responsive element-binding protein 2B mRNA, complete cds
AT5G08020	1.00862044	2.77E-09	Arabidopsis thaliana RPA70-kDa subunit B mRNA, complete cds
AT5G40000	3.408169432	2.81E-09	Arabidopsis thaliana chromosome 5 sequence
AT1G62510	1.195363059	2.82E-09	Arabidopsis thaliana chromosome 1 sequence
AT1G77320	1.086241337	2.84E-09	Arabidopsis thaliana meiosis defective 1 mRNA, complete cds
AT2G22860	1.264028456	2.93E-09	Arabidopsis thaliana phytosulfokine-beta mRNA, complete cds
AT5G19100	1.483576834	2.98E-09	Arabidopsis thaliana chromosome 5 sequence
AT1G13608	5.086241337	3.02E-09	Arabidopsis thaliana putative defensin-like protein 288 mRNA, complete cds
AT3G59700	1.280413885	3.03E-09	Arabidopsis thaliana chromosome 3, complete sequence
AT5G64630	1.172541183	3.06E-09	Arabidopsis thaliana chromatin assembly factor 1 subunit FAS2 mRNA, complete cds
AT5G06720	1.297745442	3.12E-09	Arabidopsis thaliana peroxidase 53 mRNA, complete cds
AT5G45000	1.427278255	3.19E-09	Arabidopsis thaliana TIR-NBS-LRR class disease resistance protein mRNA, complete cds
AT5G44620	1.335600806	3.24E-09	Arabidopsis thaliana cytochrome P450, family 706, subfamily A, polypeptide 3 mRNA, complete cds
AT3G12915	1.020486891	3.39E-09	Arabidopsis thaliana ribosomal protein S5/Elongation factor G/III/V family protein mRNA, complete cds
AT3G53680	1.250986099	3.44E-09	Arabidopsis thaliana Acyl-CoA N-acyltransferase with RING/FYVE/PHD-type zinc finger domain-containing protein mRNA, complete cds
AT5G28330	1.988060943	3.49E-09	Arabidopsis thaliana chromosome 5 sequence
AT1G63720	1.062262474	4.02E-09	Arabidopsis thaliana uncharacterized protein mRNA, complete cds
AT4G16820	4.173704178	4.21E-09	Arabidopsis thaliana chromosome 4 sequence
AT1G10040	1.039947685	4.57E-09	Arabidopsis thaliana alpha/beta-hydrolases superfamily protein mRNA, complete cds
AT5G23980	1.545672955	4.78E-09	Arabidopsis thaliana ferric reduction oxidase 4 mRNA, complete cds
AT1G20060	1.38963348	4.80E-09	Arabidopsis thaliana ATP binding microtubule motor family protein mRNA, complete cds

AT1G80820	1.086241337	5.27E-09	Arabidopsis thaliana cinnamoyl-CoA reductase mRNA, complete cds
AT3G22142	1.655084172	5.46E-09	Arabidopsis thaliana chromosome 3, complete sequence
AT5G22750	1.213620643	5.49E-09	Arabidopsis thaliana mRNA for hypothetical protein, complete cds, clone: RAFL16-05-A16
AT2G18190	5.040437647	5.52E-09	Arabidopsis thaliana P-loop containing nucleoside triphosphate hydrolases superfamily protein mRNA, complete cds
AT5G39890	1.448811416	5.58E-09	Arabidopsis thaliana mRNA for hypothetical protein, complete cds, clone: RAFL14-60-L23
AT4G07960	1.671203838	6.38E-09	Arabidopsis thaliana Cellulose-synthase-like C12 mRNA, complete cds
AT3G15536	3.671203838	6.56E-09	Arabidopsis thaliana chromosome 3, complete sequence
AT2G15780	5.993131932	7.38E-09	Arabidopsis thaliana Cupredoxin superfamily protein mRNA, complete cds
AT1G77310	1.581006029	7.98E-09	Arabidopsis thaliana uncharacterized protein mRNA, complete cds
AT5G22380	2.18577701	7.99E-09	Arabidopsis thaliana NAC domain-containing protein mRNA, complete cds
AT5G16980	2.243782614	8.21E-09	Arabidopsis thaliana zinc-binding dehydrogenase family protein mRNA, complete cds
AT1G51080	1.038412812	8.47E-09	Arabidopsis thaliana chromosome 1 sequence
AT1G65690	1.178687586	8.51E-09	Arabidopsis thaliana late embryogenesis abundant (LEA) hydroxyproline-rich glycoprotein mRNA, complete cds
AT5G46830	2.927543591	8.82E-09	Arabidopsis thaliana chromosome 5 sequence
AT3G20280	1.114075545	1.02E-08	Arabidopsis thaliana PHD finger protein mRNA, complete cds
AT3G55290	1.124209187	1.11E-08	Arabidopsis thaliana Rossmann-fold NAD(P)-binding domain-containing protein mRNA, complete cds
AT2G02990	5.944222332	1.30E-08	Arabidopsis thaliana ribonuclease 1 mRNA, complete cds
AT1G17665	1.095343544	1.31E-08	Arabidopsis thaliana uncharacterized protein mRNA, complete cds
AT2G41380	1.622294237	1.31E-08	Arabidopsis thaliana S-adenosyl-L-methionine-dependent methyltransferase-like protein mRNA, complete cds
AT3G13090	1.537937307	1.57E-08	Arabidopsis thaliana multidrug resistance-associated protein 8 mRNA, complete cds
AT5G15070	1.095459867	1.61E-08	Arabidopsis thaliana phosphoglycerate mutase-like protein mRNA, complete cds
AT5G52910	1.125235468	1.67E-08	Arabidopsis thaliana protein TIMELESS mRNA, complete cds
AT5G03210	3.295694702	1.75E-08	Arabidopsis thaliana chromosome 5 sequence
AT5G57510	3.295694702	1.75E-08	Arabidopsis thaliana chromosome 5 sequence
AT1G76780	1.727787366	1.82E-08	Arabidopsis thaliana chromosome 1 sequence
AT2G30660	4.944222332	1.86E-08	Arabidopsis thaliana probable 3-hydroxyisobutyryl-CoA

			hydrolase 3 mRNA, complete cds
AT2G33710	2.501278836	2.27E-08	Arabidopsis thaliana ethylene-responsive transcription factor ERF112 mRNA, complete cds
AT2G14290	5.893596259	2.29E-08	Arabidopsis thaliana chromosome 2, complete sequence
AT1G22275	2.408169432	2.37E-08	Arabidopsis thaliana synaptonemal complex protein 2 mRNA, complete cds
AT4G04223	2.858830841	2.85E-08	Arabidopsis thaliana ARM repeat superfamily protein mRNA, complete cds
AT1G35530	1.709171688	2.85E-08	Arabidopsis thaliana helicase FANCM mRNA, complete cds
AT3G61920	1.496117131	3.02E-08	Arabidopsis thaliana chromosome 3, complete sequence
AT4G04510	4.893596259	3.42E-08	Arabidopsis thaliana cysteine-rich receptor-like protein kinase 38 mRNA, complete cds
AT4G22860	1.116403307	3.53E-08	Arabidopsis thaliana cell cycle regulated microtubule associated protein mRNA, complete cds
AT3G18100	1.019127141	3.61E-08	Arabidopsis thaliana myb domain protein 4r1 mRNA, complete cds
AT4G36120	1.095956492	3.68E-08	Arabidopsis thaliana uncharacterized protein mRNA, complete cds
AT1G01680	2.111776429	3.75E-08	Arabidopsis thaliana U-box domain-containing protein 54 mRNA, complete cds
AT5G08600	1.671203838	3.84E-08	Arabidopsis thaliana U3 ribonucleoprotein (Utp) family protein mRNA, complete cds
AT5G52160	5.841128839	4.04E-08	Arabidopsis thaliana bifunctional inhibitor/lipid-transfer protein/seed storage 2S albumin-like protein mRNA, complete cds
AT4G14368	3.545672955	4.18E-08	Arabidopsis thaliana regulator of chromosome condensation repeat-containing protein mRNA, complete cds
AT5G47455	1.003779177	4.43E-08	Arabidopsis thaliana uncharacterized protein mRNA, complete cds
AT3G10310	1.076718563	4.67E-08	Arabidopsis thaliana myosin and kinesin motor and CH domain-containing protein mRNA, complete cds
AT2G43510	1.3604163	4.93E-08	Arabidopsis thaliana trypsin inhibitor protein 1 mRNA, complete cds
AT5G64510	1.002825329	5.44E-08	Arabidopsis thaliana protein TUNICAMYCIN INDUCED 1 mRNA, complete cds
AT1G76690	1.140689121	6.22E-08	Arabidopsis thaliana 12-oxophytodienoate reductase 2 mRNA, complete cds
AT2G29110	2.086241337	6.24E-08	Arabidopsis thaliana glutamate receptor 2.8 mRNA, complete cds
AT4G19750	2.545672955	6.46E-08	Arabidopsis thaliana Glycosyl hydrolase family protein with chitinase insertion domain mRNA, complete cds
AT5G22530	2.439878291	6.84E-08	Arabidopsis thaliana chromosome 5 sequence

AT5G17460	1.29983681	7.00E-08	Arabidopsis thaliana uncharacterized protein mRNA, complete cds
AT4G12480	1.893596259	7.16E-08	Arabidopsis thaliana chromosome 4 sequence
AT5G64870	1.346477109	7.25E-08	Arabidopsis thaliana SPFH/Band 7/PHB domain-containing membrane-associated protein family mRNA, complete cds
AT4G13880	1.554390173	7.33E-08	Arabidopsis thaliana receptor like protein 48 mRNA, complete cds
AT5G11210	3.501278836	7.75E-08	Arabidopsis thaliana glutamate receptor 2.5 mRNA, complete cds
AT1G64890	1.023310081	9.11E-08	Arabidopsis thaliana probable folate-biopterin transporter 7 mRNA, complete cds
AT5G49110	1.524362449	9.38E-08	Arabidopsis thaliana uncharacterized protein mRNA, complete cds
AT4G19760	3.944222332	9.50E-08	Arabidopsis thaliana Glycosyl hydrolase family protein with chitinase insertion domain mRNA, complete cds
AT5G63130	1.415946782	1.06E-07	Arabidopsis thaliana octicosapeptide/Phox/Bem1p domain-containing protein mRNA, complete cds
AT5G35760	2.113722073	1.10E-07	Arabidopsis thaliana Beta-galactosidase related protein mRNA, complete cds
AT4G23496	2.512506092	1.13E-07	Arabidopsis thaliana protein SPIRAL1-like5 mRNA, complete cds
AT1G74350	1.870512646	1.16E-07	Arabidopsis thaliana chromosome 1 sequence
AT2G37430	1.760013105	1.26E-07	Arabidopsis thaliana chromosome 2, complete sequence
AT4G40065	1.760013105	1.26E-07	Arabidopsis thaliana chromosome 4 sequence
AT5G17860	1.760013105	1.26E-07	Arabidopsis thaliana chromosome 5 sequence
AT5G52740	5.730097527	1.28E-07	Arabidopsis thaliana copper transport family protein mRNA, complete cds
AT2G24350	1.424110976	1.28E-07	Arabidopsis thaliana RNA recognition motif-containing protein mRNA, complete cds
AT1G11210	1.392902675	1.30E-07	Arabidopsis thaliana uncharacterized protein mRNA, complete cds
AT5G11470	1.468111972	1.50E-07	Arabidopsis thaliana protein ANTI-SILENCING 1 mRNA, complete cds
AT2G17770	1.986705663	1.58E-07	Arabidopsis thaliana bZIP transcription factor FD-like protein mRNA, complete cds
AT1G29560	1.632209706	1.70E-07	Arabidopsis thaliana zinc finger C-x8-C-x5-C-x3-H type family protein mRNA, complete cds
AT5G61890	3.893596259	1.77E-07	Arabidopsis thaliana ethylene-responsive transcription factor ERF114 mRNA, complete cds
AT2G37770	1.323280534	1.90E-07	Arabidopsis thaliana aldo-keto reductase family 4 member C9 mRNA, complete cds
AT5G22520	3.130635456	1.97E-07	Arabidopsis thaliana chromosome 5 sequence
AT4G21820	1.132783923	2.00E-07	Arabidopsis thaliana binding / calmodulin binding protein

			mRNA, complete cds
AT5G07700	2.211772219	2.02E-07	Arabidopsis thaliana myb domain protein 76 mRNA, complete cds
AT3G50760	1.003779177	2.08E-07	Arabidopsis thaliana chromosome 3, complete sequence
AT1G24145	1.18933483	2.14E-07	Arabidopsis thaliana uncharacterized protein mRNA, complete cds
AT5G09470	4.730097527	2.16E-07	Arabidopsis thaliana dicarboxylate carrier 3 mRNA, complete cds
AT1G02065	1.205980581	2.26E-07	Arabidopsis thaliana squamosa promoter binding protein-like 8 mRNA, complete cds
AT3G25180	5.671203838	2.28E-07	Arabidopsis thaliana cytochrome P450, family 82, subfamily G, polypeptide 1 mRNA, complete cds
AT4G18470	1.764313242	2.30E-07	Arabidopsis thaliana negative regulator of systemic acquired resistance (SNI1) mRNA, complete cds
AT4G30097	1.416389939	2.31E-07	Arabidopsis thaliana chromosome 4 sequence
AT5G01550	1.919131351	2.34E-07	Arabidopsis thaliana chromosome 5 sequence
AT4G31870	1.501278836	2.59E-07	Arabidopsis thaliana glutathione peroxidase 7 mRNA, complete cds
AT1G20570	1.792510134	2.63E-07	Arabidopsis thaliana Spc97 / Spc98 family of spindle pole body (SBP) component mRNA, complete cds
AT3G10185	3.408169432	2.66E-07	Arabidopsis thaliana Gibberellin-regulated family protein mRNA, complete cds
AT3G66652	1.176843886	3.03E-07	Arabidopsis thaliana fip1 motif-containing protein mRNA, complete cds
AT2G36780	2.058226961	3.05E-07	Arabidopsis thaliana chromosome 2, complete sequence
AT2G43000	1.716291727	3.15E-07	Arabidopsis thaliana NAC domain-containing protein 42 mRNA, complete cds
AT4G30660	2.564288634	3.22E-07	Arabidopsis thaliana putative low temperature and salt responsive protein mRNA, complete cds
AT1G54970	3.841128839	3.31E-07	Arabidopsis thaliana proline-rich protein 1 mRNA, complete cds
AT4G04370	1.026535091	3.48E-07	Arabidopsis thaliana chromosome 4 sequence
AT2G25625	1.741593165	3.64E-07	Arabidopsis thaliana uncharacterized protein mRNA, complete cds
AT4G13290	1.054532477	3.80E-07	Arabidopsis thaliana cytochrome P450 71A19 mRNA, complete cds
AT4G02390	1.017293983	3.87E-07	Arabidopsis thaliana poly(ADP-ribose) polymerase mRNA, complete cds
AT5G48010	1.483131489	3.91E-07	Arabidopsis thaliana thalianol synthase 1 mRNA, complete cds
AT5G67180	1.149251134	4.00E-07	Arabidopsis thaliana target of early activation tagged 3 mRNA, complete cds
AT1G68390	1.375747954	4.19E-07	Arabidopsis thaliana core-2/I-branching beta-1,6-N-acetylglucosaminyltransferase family protein

			mRNA, complete cds
AT1G14120	1.044421161	4.25E-07	Arabidopsis thaliana 2-oxoglutarate (2OG) and Fe(II)-dependent oxygenase-like protein mRNA, complete cds
AT5G59520	1.025699795	4.29E-07	Arabidopsis thaliana zinc transporter 2 mRNA, complete cds
AT3G23250	1.097829311	4.42E-07	Arabidopsis thaliana myb domain protein 15 mRNA, complete cds
AT4G25480	2.851776083	4.46E-07	Arabidopsis thaliana chromosome 4 sequence
AT4G14150	1.215524354	5.14E-07	Arabidopsis thaliana phragmoplast-associated kinesin-related protein 1 mRNA, complete cds
AT4G22960	2.150371674	5.74E-07	Arabidopsis thaliana uncharacterized protein mRNA, complete cds
AT5G45520	2.408169432	5.94E-07	Arabidopsis thaliana chromosome 5 sequence
AT4G26255	1.303472053	6.02E-07	Arabidopsis thaliana unknown mRNA sequence
AT5G44990	3.786681055	6.20E-07	Arabidopsis thaliana Glutathione S-transferase family protein mRNA, complete cds
AT3G44970	1.745204419	6.65E-07	Arabidopsis thaliana cytochrome P450 family protein mRNA, complete cds
AT1G67000	1.907271196	6.87E-07	Arabidopsis thaliana probable receptor-like protein kinase mRNA, complete cds
AT5G10800	1.259889424	6.92E-07	Arabidopsis thaliana RNA recognition motif (RRM)-containing protein mRNA, complete cds
AT2G34940	1.110903391	7.29E-07	Arabidopsis thaliana vacuolar-sorting receptor 5 mRNA, complete cds
AT1G09050	1.435825775	7.36E-07	Arabidopsis thaliana uncharacterized protein mRNA, complete cds
AT3G49340	5.545672955	7.38E-07	Arabidopsis thaliana putative cysteine proteinase mRNA, complete cds
AT1G16330	1.06352126	7.78E-07	Arabidopsis thaliana cyclin B3-1 mRNA, complete cds
AT4G16563	1.098416051	8.26E-07	Arabidopsis thaliana chromosome 4 sequence
AT5G44390	1.223744861	9.52E-07	Arabidopsis thaliana FAD-binding Berberine family protein mRNA, complete cds
AT3G04690	1.139680596	1.05E-06	Arabidopsis thaliana receptor-like protein kinase ANXUR1 mRNA, complete cds
AT1G52620	1.139680596	1.05E-06	Arabidopsis thaliana chromosome 1 sequence
AT1G04650	1.567368027	1.14E-06	Arabidopsis thaliana uncharacterized protein mRNA, complete cds
AT1G24575	1.189863968	1.29E-06	Arabidopsis thaliana chromosome 1 sequence
AT5G24640	5.47855876	1.33E-06	Arabidopsis thaliana chromosome 5 sequence
AT1G65480	1.3563305	1.35E-06	Arabidopsis thaliana protein FLOWERING LOCUS T mRNA, complete cds
AT5G23810	1.156630665	1.39E-06	Arabidopsis thaliana putative amino acid permease 7 mRNA, complete cds

AT5G67080	1.671203838	1.42E-06	Arabidopsis thaliana chromosome 5 sequence
AT3G05415	2.024840792	1.50E-06	Arabidopsis thaliana chromosome 3, complete sequence
AT1G18560	1.141736449	1.59E-06	Arabidopsis thaliana BED zinc finger and hAT dimerization domain-containing protein mRNA, complete cds
AT5G61290	1.073963007	1.59E-06	Arabidopsis thaliana flavin-containing monooxygenase FMO GS-OX-like 8 mRNA, complete cds
AT2G31830	1.604089642	1.60E-06	Arabidopsis thaliana Type II inositol-1,4,5-trisphosphate 5-phosphatase 14 mRNA, complete cds
AT3G23240	2.588741677	1.62E-06	Arabidopsis thaliana chromosome 3, complete sequence
AT5G64060	2.588741677	1.62E-06	Arabidopsis thaliana NAC domain containing protein 103 mRNA, complete cds
AT3G44690	1.398831567	1.64E-06	Arabidopsis thaliana ecotype Bla-1 DM2A, DM2B, DM2C, DM2D, DM2E, DM2F, DM2G, and DM2H genes, complete cds
AT3G08860	1.696294819	1.66E-06	Arabidopsis thaliana PYRIMIDINE 4 mRNA, complete cds
AT1G55040	1.112713548	1.68E-06	Arabidopsis thaliana Zn-finger in Ran binding domain-containing protein mRNA, complete cds
AT3G62590	1.15803202	1.71E-06	Arabidopsis thaliana alpha/beta-Hydrolases superfamily protein mRNA, complete cds
AT3G04640	2.23824443	1.76E-06	Arabidopsis thaliana chromosome 3, complete sequence
AT3G49370	1.000084693	1.83E-06	Arabidopsis thaliana CDPK-related kinase 6 mRNA, complete cds
AT5G61390	1.019127141	1.83E-06	Arabidopsis thaliana polynucleotidyl transferase, ribonuclease H-like superfamily protein mRNA, complete cds
AT3G30340	1.624661252	1.89E-06	Arabidopsis thaliana nodulin MtN21 /EamA-like transporter family protein mRNA, complete cds
AT2G15830	1.196665327	2.39E-06	Arabidopsis thaliana chromosome 2, complete sequence
AT5G28085	5.408169432	2.42E-06	Arabidopsis thaliana chromosome 5 sequence
AT5G24540	5.408169432	2.42E-06	Arabidopsis thaliana beta glucosidase 31 mRNA, complete cds
AT1G14520	1.007631502	2.50E-06	Arabidopsis thaliana inositol oxygenase 1 mRNA, complete cds
AT1G66570	4.47855876	2.60E-06	Arabidopsis thaliana putative sucrose transport protein SUC7 mRNA, complete cds
AT1G02190	1.671203838	2.60E-06	Arabidopsis thaliana protein CER1-like 1 mRNA, complete cds
AT1G04600	2.053074473	2.68E-06	Arabidopsis thaliana myosin XI A mRNA, complete cds
AT1G10417	2.121865247	2.86E-06	Arabidopsis thaliana uncharacterized protein mRNA, complete cds
AT5G02190	1.823206931	2.90E-06	Arabidopsis thaliana chromosome 5 sequence
AT1G09040	1.428633534	2.92E-06	Arabidopsis thaliana uncharacterized protein mRNA, complete cds
AT4G24010	1.60081451	2.93E-06	Arabidopsis thaliana cellulose synthase-like protein G1

			mRNA, complete cds
AT5G50940	2.201718554	2.98E-06	Arabidopsis thaliana RNA-binding KH domain-containing protein mRNA, complete cds
AT2G25820	2.295694702	3.04E-06	Arabidopsis thaliana chromosome 2, complete sequence
AT2G12490	1.086241337	3.26E-06	Arabidopsis thaliana chromosome 2, complete sequence
AT5G66960	1.486779266	3.39E-06	Arabidopsis thaliana Prolyl oligopeptidase family protein mRNA, complete cds
AT2G07698	1.622294237	3.46E-06	Arabidopsis thaliana ATPase, F1 complex, alpha subunit protein mRNA, complete cds
AT3G10815	1.72669895	3.52E-06	Arabidopsis thaliana RING/U-box domain-containing protein mRNA, complete cds
AT3G44450	1.100047136	3.56E-06	Arabidopsis thaliana chromosome 3, complete sequence
AT2G33240	1.557547056	3.81E-06	Arabidopsis thaliana myosin XI D mRNA, complete cds
AT3G02840	1.223744861	3.82E-06	Arabidopsis thaliana chromosome 3, complete sequence
AT4G05631	1.130635456	4.18E-06	Arabidopsis thaliana uncharacterized protein mRNA, complete cds
AT5G04150	1.100316522	4.38E-06	Arabidopsis thaliana transcription factor bHLH101 mRNA, complete cds
AT1G27710	5.33416885	4.41E-06	Arabidopsis thaliana chromosome 1 sequence
AT1G74430	1.794060585	4.66E-06	Arabidopsis thaliana putative transcription factor MYB95 mRNA, complete cds
AT4G19080	2.086241337	4.79E-06	Arabidopsis thaliana chromosome 4 sequence
AT1G73810	1.251827403	4.91E-06	Arabidopsis thaliana core-2/I-branching beta-1,6-N-acetylglucosaminyltransferase-like protein mRNA, complete cds
AT5G48410	2.501278836	5.01E-06	Arabidopsis thaliana glutamate receptor 1.3 mRNA, complete cds
AT4G13420	2.164243849	5.05E-06	Arabidopsis thaliana potassium transporter 5 mRNA, complete cds
AT5G24205	2.164243849	5.05E-06	Arabidopsis thaliana chromosome 5 sequence
AT5G39100	3.145135026	5.69E-06	Arabidopsis thaliana germin-like protein 6 mRNA, complete cds
AT5G02430	1.100888113	6.67E-06	Arabidopsis thaliana transducin/WD40 domain-containing protein mRNA, complete cds
AT1G12480	1.551904909	6.97E-06	Arabidopsis thaliana guard cell S-type anion channel SLAC1 mRNA, complete cds
AT4G37290	2.841128839	7.12E-06	Arabidopsis thaliana chromosome 4 sequence
AT1G69120	1.493416718	7.44E-06	Arabidopsis thaliana Floral homeotic protein APETALA 1 mRNA, complete cds
AT5G51920	1.64423679	7.47E-06	Arabidopsis thaliana chromosome 5 sequence
AT3G02040	1.0587606	7.74E-06	Arabidopsis thaliana glycerophosphodiester phosphodiesterase 1 mRNA, complete cds
AT5G28390	1.133547052	7.84E-06	Arabidopsis thaliana RNA recognition motif-containing

			protein mRNA, complete cds
AT2G29165	5.256166338	8.08E-06	Arabidopsis thaliana chromosome 2, complete sequence
AT5G22545	5.256166338	8.08E-06	Arabidopsis thaliana chromosome 5 sequence
AT1G12030	2.622294237	8.16E-06	Arabidopsis thaliana uncharacterized protein mRNA, complete cds
AT4G11370	1.571668164	8.30E-06	Arabidopsis thaliana chromosome 4 sequence
AT5G48400	2.215524354	8.87E-06	Arabidopsis thaliana glutamate receptor 1.2 mRNA, complete cds
AT1G48330	1.117268232	8.93E-06	Arabidopsis thaliana chromosome 1 sequence
AT1G43675	1.295694702	9.38E-06	Arabidopsis thaliana chromosome 1 sequence
AT1G48500	1.071741767	1.05E-05	Arabidopsis thaliana protein TIFY 6A mRNA, complete cds
AT1G24330	1.173704178	1.11E-05	Arabidopsis thaliana U-box domain-containing protein 6 mRNA, complete cds
AT1G15310	1.469569976	1.13E-05	Arabidopsis thaliana signal recognition particle protein SRP54A mRNA, complete cds
AT5G26690	1.301970028	1.15E-05	Arabidopsis thaliana heavy-metal-associated domain-containing protein mRNA, complete cds
AT1G19630	1.616756054	1.17E-05	Arabidopsis thaliana cytochrome P450, family 722, subfamily A, polypeptide 1 mRNA, complete cds
AT5G66130	1.336784798	1.17E-05	Arabidopsis thaliana cell cycle checkpoint protein RAD17 mRNA, complete cds
AT2G35480	1.375747954	1.18E-05	Arabidopsis thaliana uncharacterized protein mRNA, complete cds
AT3G56870	1.135710013	1.19E-05	Arabidopsis thaliana uncharacterized protein mRNA, complete cds
AT4G35640	1.770739511	1.37E-05	Arabidopsis thaliana serine acetyltransferase 3;2 mRNA, complete cds
AT2G26010	3.47855876	1.42E-05	Arabidopsis thaliana plant defensin 1.3 mRNA, complete cds
AT2G38340	3.47855876	1.42E-05	Arabidopsis thaliana chromosome 2, complete sequence
AT4G15370	3.47855876	1.42E-05	Arabidopsis thaliana baruol synthase 1 mRNA, complete cds
AT2G17850	3.47855876	1.42E-05	Arabidopsis thaliana rhodanese-like domain-containing protein 17 mRNA, complete cds
AT1G05950	1.057094991	1.45E-05	Arabidopsis thaliana uncharacterized protein mRNA, complete cds
AT4G29990	1.13686741	1.47E-05	Arabidopsis thaliana leucine-rich repeat transmembrane protein kinase mRNA, complete cds
AT2G44070	5.173704178	1.49E-05	Arabidopsis thaliana NagB/RpiA/CoA transferase-like superfamily protein mRNA, complete cds
AT2G44798	5.173704178	1.49E-05	Arabidopsis thaliana mRNA for hypothetical protein, complete cds, clone: RAFL16-84-G21
AT4G27670	5.173704178	1.49E-05	Arabidopsis thaliana heat shock protein 21 mRNA, complete cds

AT1G71390	5.173704178	1.49E-05	Arabidopsis thaliana chromosome 1 sequence
AT5G06510	1.566234278	1.52E-05	Arabidopsis thaliana nuclear transcription factor Y subunit A-10 mRNA, complete cds
AT5G40100	1.071134444	1.60E-05	Arabidopsis thaliana TIR-NBS-LRR class disease resistance protein mRNA, complete cds
AT4G24110	1.44532243	1.70E-05	Arabidopsis thaliana chromosome 4 sequence
AT2G20800	4.256166338	1.73E-05	Arabidopsis thaliana NAD(P)H dehydrogenase B4 mRNA, complete cds
AT3G49540	4.256166338	1.73E-05	Arabidopsis thaliana uncharacterized protein mRNA, complete cds
AT5G35932	4.256166338	1.73E-05	Arabidopsis thaliana chromosome 5 sequence
AT5G44540	4.256166338	1.73E-05	Arabidopsis thaliana chromosome 5 sequence
AT3G28945	1.353721648	1.74E-05	Arabidopsis thaliana chromosome 3, complete sequence
AT1G11100	1.086241337	1.75E-05	Arabidopsis thaliana SNF2 , helicase and zinc-finger domain-containing protein mRNA, complete cds
AT1G51820	1.588741677	1.81E-05	Arabidopsis thaliana putative LRR receptor-like serine/threonine protein kinase mRNA, complete cds
AT2G30766	1.138080268	1.81E-05	Arabidopsis thaliana chromosome 2, complete sequence
AT5G10100	1.138080268	1.81E-05	Arabidopsis thaliana probable trehalose-phosphate phosphatase I mRNA, complete cds
AT5G60150	1.029657808	1.82E-05	Arabidopsis thaliana uncharacterized protein mRNA, complete cds
AT4G29610	1.702912697	1.88E-05	Arabidopsis thaliana chromosome 4 sequence
AT2G33760	3.024840792	1.92E-05	Arabidopsis thaliana chromosome 2, complete sequence
AT1G44130	3.024840792	1.92E-05	Arabidopsis thaliana aspartyl protease family protein mRNA, complete cds
AT5G17370	1.070811296	1.97E-05	Arabidopsis thaliana transducin/WD40 domain-containing protein mRNA, complete cds
AT5G60250	1.912211937	1.99E-05	Arabidopsis thaliana C3H4 type zinc finger protein mRNA, complete cds
AT2G30640	1.459699732	2.06E-05	Arabidopsis thaliana chromosome 2, complete sequence
AT2G39240	1.61348834	2.14E-05	Arabidopsis thaliana RNA polymerase I specific transcription initiation factor RRN3 protein mRNA, complete cds
AT4G23200	1.973766608	2.20E-05	Arabidopsis thaliana putative cysteine-rich receptor-like protein kinase 12 mRNA, complete cds
AT1G66590	1.028525839	2.24E-05	Arabidopsis thaliana cytochrome c oxidase 19-1 mRNA, complete cds
AT5G61940	1.538753542	2.34E-05	Arabidopsis thaliana ubiquitin carboxyl-terminal hydrolase-related protein mRNA, complete cds
AT3G48770	2.045599352	2.39E-05	Arabidopsis thaliana ATP/DNA binding protein mRNA, complete cds
AT3G23370	1.475283628	2.48E-05	Arabidopsis thaliana RNA recognition motif-containing protein mRNA, complete cds

AT4G23160	2.130635456	2.55E-05	Arabidopsis thaliana cysteine-rich receptor-like protein kinase 8 mRNA, complete cds
AT1G57560	1.121006755	2.55E-05	Arabidopsis thaliana myb domain protein 50 mRNA, complete cds
AT1G75880	1.373122485	2.59E-05	Arabidopsis thaliana GDSL esterase/lipase EXL1 mRNA, complete cds
AT2G46455	3.408169432	2.65E-05	Arabidopsis thaliana OxaA/YidC-like membrane insertion protein mRNA, complete cds
AT2G12440	5.086241337	2.75E-05	Arabidopsis thaliana chromosome 2, complete sequence
AT1G70130	5.086241337	2.75E-05	Arabidopsis thaliana putative L-type lectin-domain containing receptor kinase V.2 mRNA, complete cds
AT5G10040	5.086241337	2.75E-05	Arabidopsis thaliana chromosome 5 sequence
AT3G25490	5.086241337	2.75E-05	Arabidopsis thaliana putative wall-associated receptor kinase-like 16 mRNA, complete cds
AT4G25000	1.560172525	2.80E-05	Arabidopsis thaliana alpha-amylase 1 mRNA, complete cds
AT4G15730	1.16219019	2.97E-05	Arabidopsis thaliana CW-type zinc-finger protein mRNA, complete cds
AT4G23700	1.266813582	3.00E-05	Arabidopsis thaliana cation/H(+) antiporter 17 mRNA, complete cds
AT2G31180	1.43416464	3.11E-05	Arabidopsis thaliana myb domain protein 14 mRNA, complete cds
AT1G70800	1.383921885	3.16E-05	Arabidopsis thaliana protein ENHANCED BENDING 1 mRNA, complete cds
AT2G45940	4.173704178	3.26E-05	Arabidopsis thaliana uncharacterized protein mRNA, complete cds
AT4G13650	1.211772219	3.36E-05	Arabidopsis thaliana pentatricopeptide repeat-containing protein mRNA, complete cds
AT1G71520	2.960710455	3.52E-05	Arabidopsis thaliana chromosome 1 sequence
AT3G09450	1.934238243	3.62E-05	Arabidopsis thaliana uncharacterized protein mRNA, complete cds
AT2G47560	1.069753214	3.71E-05	Arabidopsis thaliana chromosome 2, complete sequence
AT4G37030	1.069753214	3.71E-05	Arabidopsis thaliana uncharacterized protein mRNA, complete cds
AT2G39725	1.448811416	3.76E-05	Arabidopsis thaliana LYR family of Fe/S cluster biogenesis protein mRNA, complete cds
AT1G06160	1.122767213	3.89E-05	Arabidopsis thaliana chromosome 1 sequence
AT1G68870	1.024840792	4.21E-05	Arabidopsis thaliana chromosome 1 sequence
AT5G19097	1.786681055	4.62E-05	Arabidopsis thaliana chromosome 5 sequence
AT4G12490	1.786681055	4.62E-05	Arabidopsis thaliana chromosome 4 sequence
AT1G52560	1.63878236	4.66E-05	Arabidopsis thaliana heat shock protein 26.5 mRNA, complete cds
AT2G22880	1.408169432	4.67E-05	Arabidopsis thaliana chromosome 2, complete sequence

AT1G04700	1.359259831	4.70E-05	Arabidopsis thaliana PB1 domain-containing protein tyrosine kinase mRNA, complete cds
AT4G28700	3.33416885	4.94E-05	Arabidopsis thaliana chromosome 4 sequence
AT4G25950	3.33416885	4.94E-05	Arabidopsis thaliana V-type proton ATPase subunit G3 mRNA, complete cds
AT2G16367	3.33416885	4.94E-05	Arabidopsis thaliana chromosome 2, complete sequence
AT3G51400	3.33416885	4.94E-05	Arabidopsis thaliana chromosome 3, complete sequence
AT4G29980	4.993131932	5.10E-05	Arabidopsis thaliana chromosome 4 sequence
AT3G62455	1.836263084	5.27E-05	Arabidopsis thaliana chromosome 3, complete sequence
AT1G05490	1.836263084	5.27E-05	Arabidopsis thaliana chromatin remodeling 31 mRNA, complete cds
AT3G52740	1.037331736	5.76E-05	Arabidopsis thaliana chromosome 3, complete sequence
ATMG00660	1.893596259	5.94E-05	Arabidopsis thaliana ecotype Landsberg erecta mitochondrion, complete genome
AT1G21525	4.086241337	6.16E-05	Arabidopsis thaliana chromosome 1 sequence
AT4G14050	1.086241337	6.20E-05	Arabidopsis thaliana chromosome 4 sequence
AT1G66830	1.707729714	6.39E-05	Arabidopsis thaliana probable inactive leucine-rich repeat receptor-like protein kinase mRNA, complete cds
AT3G61400	2.893596259	6.43E-05	Arabidopsis thaliana 1-aminocyclopropane-1-carboxylate oxidase-like 8 mRNA, complete cds
AT1G57750	2.893596259	6.43E-05	Arabidopsis thaliana chromosome 1 sequence
AT1G26330	1.105100364	6.76E-05	Arabidopsis thaliana DNA binding protein mRNA, complete cds
AT2G30424	1.436738584	6.88E-05	Arabidopsis thaliana protein trichomeless 2 mRNA, complete cds
AT4G14850	1.036200654	7.11E-05	Arabidopsis thaliana pentatricopeptide repeat-containing protein LOI1 mRNA, complete cds
AT3G60540	2.040437647	7.20E-05	Arabidopsis thaliana chromosome 3, complete sequence
AT5G39220	1.74920635	7.41E-05	Arabidopsis thaliana hydrolase, alpha/beta fold family protein mRNA, complete cds
AT4G36950	2.609803293	7.42E-05	Arabidopsis thaliana chromosome 4 sequence
AT1G11925	2.609803293	7.42E-05	Arabidopsis thaliana chromosome 1 sequence
AT2G17060	1.228260342	7.74E-05	Arabidopsis thaliana TIR-NBS-LRR class disease resistance protein mRNA, complete cds
AT5G24155	1.148525615	7.92E-05	Arabidopsis thaliana FAD/NAD(P)-binding oxidoreductase family protein mRNA, complete cds
AT1G69710	2.256166338	7.95E-05	Arabidopsis thaliana regulator of chromosome condensation and FYVE zinc finger domain-containing protein mRNA, complete cds
AT2G32030	1.105606662	8.36E-05	Arabidopsis thaliana chromosome 2, complete sequence
AT4G17100	1.173704178	8.50E-05	Arabidopsis thaliana uncharacterized protein mRNA, complete cds
AT3G04420	1.79673472	8.52E-05	Arabidopsis thaliana NAC domain containing protein 48

			mRNA, complete cds
AT4G23493	1.201718554	9.04E-05	Arabidopsis thaliana uncharacterized protein mRNA, complete cds
AT1G52130	3.256166338	9.23E-05	Arabidopsis thaliana mannose-binding lectin-like protein mRNA, complete cds
AT2G27880	3.256166338	9.23E-05	Arabidopsis thaliana argonaute 5 mRNA, complete cds
AT4G14080	4.893596259	9.53E-05	Arabidopsis thaliana putative glucan endo-1,3-beta-glucosidase A6 mRNA, complete cds
AT3G24900	4.893596259	9.53E-05	Arabidopsis thaliana chromosome 3, complete sequence
AT1G36610	4.893596259	9.53E-05	Arabidopsis thaliana chromosome 1 sequence
AT2G30830	4.893596259	9.53E-05	Arabidopsis thaliana 2-oxoglutarate dependent oxygenase-like protein mRNA, complete cds
AT4G09820	1.851776083	9.69E-05	Arabidopsis thaliana transcription factor TT8 mRNA, complete cds
AT3G13784	1.308633758	0.000102575	Arabidopsis thaliana beta-fructofuranosidase, insoluble isoenzyme CWINV5 mRNA, complete cds
AT5G15800	1.354730173	0.000104059	Arabidopsis thaliana MADS box transcription factor SEPALLATA 1 mRNA, complete cds
AT5G47980	1.205540265	0.000111597	Arabidopsis thaliana chromosome 5 sequence
AT2G22905	3.993131932	0.000116912	Arabidopsis thaliana chromosome 2, complete sequence
AT1G08860	3.993131932	0.000116912	Arabidopsis thaliana protein BONZAI 3 mRNA, complete cds
AT4G23240	1.710732202	0.000118226	Arabidopsis thaliana cysteine-rich receptor-like protein kinase 16 mRNA, complete cds
AT3G18250	1.993131932	0.000120117	Arabidopsis thaliana chromosome 3, complete sequence
AT4G21300	1.423276324	0.000125878	Arabidopsis thaliana chromosome 4 sequence
AT2G38823	1.366349256	0.000127144	Arabidopsis thaliana uncharacterized protein mRNA, complete cds
AT4G08210	2.086241337	0.000129818	Arabidopsis thaliana chromosome 4 sequence
AT5G44630	2.545672955	0.000132184	Arabidopsis thaliana sesquiterpene synthase mRNA, complete cds
AT5G24655	2.201718554	0.000136396	Arabidopsis thaliana chromosome 5 sequence
ATCG00180	1.209623752	0.000137713	Arabidopsis lyrata subsp. lyrata RNA polymerase beta subunit-1, mRNA
AT3G28740	2.349275743	0.000137925	Arabidopsis thaliana cytochrome P450 CYP81D11 mRNA, complete cds
AT3G28600	2.349275743	0.000137925	Arabidopsis thaliana chromosome 3, complete sequence

AT4G17660	2.349275743	0.000137925	Arabidopsis thaliana protein kinase family protein mRNA, complete cds
AT1G26208	2.349275743	0.000137925	Arabidopsis thaliana clone 231852 mRNA sequence
AT1G18100	2.349275743	0.000137925	Arabidopsis thaliana protein MOTHER of FT and TF 1 mRNA, complete cds
AT3G19330	1.086241337	0.000144902	Arabidopsis thaliana uncharacterized protein mRNA, complete cds
AT3G04980	1.182456652	0.000160154	Arabidopsis thaliana chromosome 3, complete sequence
AT4G11393	3.173704178	0.000171948	Arabidopsis thaliana Defensin-like (DEFL) family protein mRNA, complete cds
AT1G07500	3.173704178	0.000171948	Arabidopsis thaliana uncharacterized protein mRNA, complete cds
AT5G44565	1.249740069	0.000178495	Arabidopsis thaliana uncharacterized protein mRNA, complete cds
AT3G11580	1.249740069	0.000178495	Arabidopsis thaliana AP2/B3-like transcriptional factor family protein mRNA, complete cds
AT2G04515	4.786681055	0.000179044	Arabidopsis thaliana chromosome 2, complete sequence
AT2G17140	1.086241337	0.000179317	Arabidopsis thaliana pentatricopeptide repeat-containing protein mRNA, complete cds
AT1G02460	1.047767189	0.000183672	Arabidopsis thaliana pectin lyase-like superfamily protein mRNA, complete cds
AT1G27720	1.564288634	0.000209727	Arabidopsis thaliana TBP-associated factor 4B mRNA, complete cds
AT2G39510	1.564288634	0.000209727	Arabidopsis thaliana nodulin MtN21-like transporter family protein mRNA, complete cds
AT4G01895	1.218691633	0.00020979	Arabidopsis thaliana chromosome 4 sequence
AT3G07274	1.132045026	0.000212324	Arabidopsis thaliana chromosome 3, complete sequence
AT5G37490	2.74920635	0.000212851	Arabidopsis thaliana chromosome 5 sequence
AT3G47720	2.74920635	0.000212851	Arabidopsis thaliana probable inactive poly [ADP-ribose] polymerase SRO4 mRNA, complete cds
AT2G47460	2.033773917	0.000218336	Arabidopsis thaliana transcription factor MYB12 mRNA, complete cds
AT1G21230	2.033773917	0.000218336	Arabidopsis thaliana wall-associated receptor kinase 5 mRNA, complete cds
AT1G07390	1.086241337	0.000221979	Arabidopsis thaliana receptor like protein 1 mRNA, complete cds
AT5G27220	3.893596259	0.000222284	Arabidopsis thaliana Frigida-like protein mRNA, complete cds
AT5G44570	3.893596259	0.00022228	Arabidopsis thaliana uncharacterized protein mRNA,

		4	complete cds
AT1G69140	3.893596259	0.00022228 4	Arabidopsis thaliana chromosome 1 sequence
AT1G12845	1.046712973	0.00022732 1	Arabidopsis thaliana chromosome 1 sequence
AT1G02670	1.29923506	0.00022751 4	Arabidopsis thaliana P-loop containing nucleoside triphosphate hydrolases superfamily protein mRNA, complete cds
AT5G41550	1.349275743	0.00023146 8	Arabidopsis thaliana TIR-NBS-LRR class disease resistance protein mRNA, complete cds
AT5G54060	1.065482777	0.00025053 8	Arabidopsis thaliana chromosome 5 sequence
AT4G10940	1.764313242	0.00025396 4	Arabidopsis thaliana RING/U-box protein mRNA, complete cds
AT1G58430	1.764313242	0.00025396 4	Arabidopsis thaliana anther-specific proline-rich protein RXF26 mRNA, complete cds
AT3G14395	1.764313242	0.00025396 4	Arabidopsis thaliana uncharacterized protein mRNA, complete cds
AT1G13330	1.501278836	0.00026993 8	Arabidopsis thaliana homologous-pairing protein 2-like protein mRNA, complete cds
AT5G01180	1.42504325	0.00028060 1	Arabidopsis thaliana peptide transporter PTR5 mRNA, complete cds
AT4G06746	1.42504325	0.00028060 1	Arabidopsis thaliana chromosome 4 sequence
AT4G15165	1.630561853	0.00029604 1	Arabidopsis thaliana N-terminal nucleophile aminohydrolases (Ntn hydrolases) superfamily protein mRNA, complete cds
AT5G46100	1.10932495	0.00030001 8	Arabidopsis thaliana chromosome 5 sequence
AT2G33350	3.086241337	0.00032002 3	Arabidopsis thaliana CCT motif-containing protein mRNA, complete cds
AT2G23270	3.086241337	0.00032002 3	Arabidopsis thaliana chromosome 2, complete sequence
AT5G57720	3.086241337	0.00032002 3	Arabidopsis thaliana AP2/B3-like transcriptional factor family protein mRNA, complete cds
AT1G51620	1.135150937	0.00032542 3	Arabidopsis thaliana protein kinase superfamily protein mRNA, complete cds
AT5G61950	1.135150937	0.00032542 3	Arabidopsis thaliana ubiquitin carboxyl-terminal hydrolase-related protein mRNA, complete cds
AT5G14960	1.270665908	0.00033391 1	Arabidopsis thaliana DP-E2F-like 2 mRNA, complete cds
AT4G05370	4.671203838	0.00033830 7	Arabidopsis thaliana chromosome 4 sequence
AT4G38780	1.443793341	0.00034083	Arabidopsis thaliana Pre-mRNA-processing-splicing factor

		1	mRNA, complete cds
AT5G11930	1.375747954	0.00034636 8	Arabidopsis thaliana chromosome 5 sequence
AT4G14120	1.044421161	0.00034855 6	Arabidopsis thaliana uncharacterized protein mRNA, complete cds
AT4G17785	1.979326133	0.00036518 7	Arabidopsis thaliana transcription factor MYB39 mRNA, complete cds
AT4G36830	1.197272649	0.00037393 9	Arabidopsis thaliana chromosome 4 sequence
AT4G15236	2.671203838	0.00038541 5	Arabidopsis thaliana ABC transporter G family member 43 mRNA, complete cds
AT1G66960	1.46475296	0.00041318 5	Arabidopsis thaliana Terpenoid cyclases family protein mRNA, complete cds
AT5G52290	3.786681055	0.00042357 4	Arabidopsis thaliana protein SHORTAGE IN CHIASMATA 1 mRNA, complete cds
AT3G44830	3.786681055	0.00042357 4	Arabidopsis thaliana putative phospholipid:diacylglycerol acyltransferase 2 mRNA, complete cds
AT1G57850	3.786681055	0.00042357 4	Arabidopsis thaliana Toll-Interleukin-Resistance domain-containing protein mRNA, complete cds
AT5G44005	1.043172615	0.00043184 5	Arabidopsis thaliana chromosome 5 sequence
AT1G33720	1.02304751	0.00048281 4	Arabidopsis thaliana cytochrome P450, family 76, subfamily C, polypeptide 6 mRNA, complete cds
AT1G61460	1.02304751	0.00048281 4	Arabidopsis thaliana G-type lectin S-receptor-like serine/threonine-protein kinase mRNA, complete cds
AT3G59710	1.241519562	0.00048753	Arabidopsis thaliana Rossmann-fold NAD(P)-binding domain-containing protein mRNA, complete cds
AT1G22380	1.48833978	0.00049972 1	Arabidopsis thaliana UDP-glucosyl transferase 85A3 mRNA, complete cds
AT1G23935	1.34258109	0.00051730 6	Arabidopsis thaliana uncharacterized protein mRNA, complete cds
AT1G19940	1.170305602	0.00053722 3	Arabidopsis thaliana glycosyl hydrolase 9B5 mRNA, complete cds
AT1G34460	1.841128839	0.00053970 9	Arabidopsis thaliana cyclin-B1-5 mRNA, complete cds
AT1G64572	1.626809718	0.00055139 6	Arabidopsis thaliana chromosome 1 sequence
AT5G05300	1.626809718	0.00055139 6	Arabidopsis thaliana chromosome 5 sequence
AT2G36026	2.993131932	0.00059451 2	Arabidopsis thaliana chromosome 2, complete sequence
ATMG0056 0	2.993131932	0.00059451 2	Arabidopsis thaliana ecotype Landsberg erecta mitochondrion, complete genome
ATCG00760	2.993131932	0.00059451	Olimarabidopsis pumila chloroplast DNA, complete

		2	sequence
AT5G09950	1.515084636	0.00060264 4	Arabidopsis thaliana chromosome 5 sequence
AT5G27895	1.515084636	0.00060264 4	Arabidopsis thaliana chromosome 5 sequence
AT3G06630	1.515084636	0.00060264 4	Arabidopsis thaliana protein kinase family protein mRNA, complete cds
AT1G09350	1.922742605	0.00060720 6	Arabidopsis thaliana galactinol synthase 3 mRNA, complete cds
AT3G54530	4.545672955	0.00064326	Arabidopsis thaliana uncharacterized protein mRNA, complete cds
AT5G07230	4.545672955	0.00064326	Arabidopsis thaliana Tapetum-specific protein A9 mRNA, complete cds
AT1G55230	4.545672955	0.00064326	Arabidopsis thaliana chromosome 1 sequence
AT4G10250	4.545672955	0.00064326	Arabidopsis thaliana chromosome 4 sequence
AT1G59950	4.545672955	0.00064326	Arabidopsis thaliana putative aldo/keto reductase mRNA, complete cds
AT3G52130	4.545672955	0.00064326	Arabidopsis thaliana chromosome 3, complete sequence
AT1G47405	4.545672955	0.00064326	Arabidopsis thaliana chromosome 1 sequence
AT5G19880	4.545672955	0.00064326	Arabidopsis thaliana peroxidase mRNA, complete cds
AT2G35570	4.545672955	0.00064326	Arabidopsis thaliana chromosome 2, complete sequence
AT4G11350	1.086241337	0.00064894 7	Arabidopsis thaliana uncharacterized protein mRNA, complete cds
AT2G29950	1.671203838	0.00065032	Arabidopsis thaliana chromosome 2, complete sequence
AT3G50770	1.040437647	0.00066368 6	Arabidopsis thaliana chromosome 3, complete sequence
AT2G38720	1.211772219	0.00070802 7	Arabidopsis thaliana microtubule-associated protein 65-5 mRNA, complete cds
AT1G53100	2.33416885	0.00072842 6	Arabidopsis thaliana core-2/I-branching beta-1,6-N-acetylglucosaminyltransferase-like protein mRNA, complete cds
AT5G01900	2.33416885	0.00072842 6	Arabidopsis thaliana putative WRKY transcription factor 62 mRNA, complete cds
AT1G01700	1.019127141	0.00074178 4	Arabidopsis thaliana Rop guanine nucleotide exchange factor 2 mRNA, complete cds
AT4G00870	1.256166338	0.00074355 4	Arabidopsis thaliana transcription factor bHLH14 mRNA, complete cds
AT1G50400	1.723671257	0.00076056 6	Arabidopsis thaliana eukaryotic porin family protein mRNA, complete cds
AT3G43250	3.671203838	0.00080887	Arabidopsis thaliana chromosome 3, complete sequence
AT1G26976	3.671203838	0.00080887	Arabidopsis thaliana uncharacterized protein mRNA, complete cds
AT1G43765	3.671203838	0.00080887	Arabidopsis thaliana clone 103892 mRNA sequence

AT2G19530	1.038935622	0.000823309	Arabidopsis thaliana uncharacterized protein mRNA, complete cds
AT3G02330	1.581006029	0.000866415	Arabidopsis thaliana pentatricopeptide repeat-containing protein mRNA, complete cds
AT2G32770	1.21748587	0.000876337	Arabidopsis thaliana purple acid phosphatase 13 mRNA, complete cds
AT4G25420	1.786681055	0.000879851	Arabidopsis thaliana gibberellin 20 oxidase 1 mRNA, complete cds
AT3G09480	1.786681055	0.000879851	Arabidopsis thaliana chromosome 3, complete sequence
AT5G66700	1.786681055	0.000879851	Arabidopsis thaliana homeobox-leucine zipper protein ATHB-53 mRNA, complete cds
AT1G22490	1.016978674	0.000920032	Arabidopsis thaliana transcription factor bHLH94 mRNA, complete cds
AT4G35520	1.32070659	0.00094539	Arabidopsis thaliana DNA mismatch repair protein MLH3 mRNA, complete cds
AT5G57540	1.863848915	0.00100323	Arabidopsis thaliana xyloglucan endotransglucosylase/hydrolase 13 mRNA, complete cds
AT3G01319	2.893596259	0.001101872	Arabidopsis thaliana chromosome 3, complete sequence
AT5G06400	2.893596259	0.001101872	Arabidopsis thaliana chromosome 5 sequence
AT1G72660	2.893596259	0.001101872	Arabidopsis thaliana developmentally regulated G-protein 2 mRNA, complete cds
AT1G54160	1.014688076	0.001141634	Arabidopsis thaliana nuclear transcription factor Y subunit A-5 mRNA, complete cds
AT4G33985	1.014688076	0.001141634	Arabidopsis thaliana uncharacterized protein mRNA, complete cds
AT1G74870	1.408169432	0.001162325	Arabidopsis thaliana RING-finger domain-containing protein mRNA, complete cds
AT3G57970	2.086241337	0.001219673	Arabidopsis thaliana Emsy N Terminus and plant Tudor-like domain-containing protein mRNA, complete cds
AT4G25980	2.086241337	0.001219673	Arabidopsis thaliana peroxidase 43 mRNA, complete cds
AT1G35112	2.086241337	0.001219673	Arabidopsis thaliana chromosome 1 sequence
AT5G66890	4.408169432	0.001231292	Arabidopsis thaliana putative disease resistance protein mRNA, complete cds
AT2G43580	4.408169432	0.001231292	Arabidopsis thaliana chitinase family protein mRNA, complete cds
AT4G37140	4.408169432	0.001231292	Arabidopsis thaliana putative inactive methylesterase 20 mRNA, complete cds
AT1G74420	1.18577701	0.00126827	Arabidopsis thaliana fucosyltransferase 3 mRNA,

		4	complete cds
AT5G40990	2.256166338	0.001273444	Arabidopsis thaliana GDSL lipase 1 mRNA, complete cds
AT5G24600	2.256166338	0.001273444	Arabidopsis thaliana uncharacterized protein mRNA, complete cds
AT4G21920	2.256166338	0.001273444	Arabidopsis thaliana chromosome 4 sequence
AT5G20710	1.730097527	0.001424716	Arabidopsis thaliana beta-galactosidase 7 mRNA, complete cds
AT4G30700	1.150371674	0.001468509	Arabidopsis thaliana chromosome 4 sequence
AT4G13390	1.150371674	0.001468509	Arabidopsis thaliana chromosome 4 sequence
AT2G26020	3.545672955	0.0015477	Arabidopsis thaliana putative defensin-like protein mRNA, complete cds
AT5G60470	3.545672955	0.0015477	Arabidopsis thaliana C2H2 and C2HC zinc finger-containing protein mRNA, complete cds
AT5G21120	3.545672955	0.0015477	Arabidopsis thaliana chromosome 5 sequence
AT3G09640	3.545672955	0.0015477	Arabidopsis thaliana L-ascorbate peroxidase 2 mRNA, complete cds
AT3G03480	1.190577997	0.001573627	Arabidopsis thaliana acetyl CoA:(Z)-3-hexen-1-ol acetyltransferase mRNA, complete cds
AT5G44578	1.190577997	0.001573627	Arabidopsis thaliana uncharacterized protein mRNA, complete cds
AT1G15040	1.802448371	0.001646274	Arabidopsis thaliana chromosome 1 sequence
AT1G56250	1.117268232	0.001685639	Arabidopsis thaliana F-box protein PP2-B14 mRNA, complete cds
AT5G61070	1.455475146	0.001726561	Arabidopsis thaliana histone deacetylase 18 mRNA, complete cds
AT3G11000	1.295694702	0.001728997	Arabidopsis thaliana DCD (Development and Cell Death) domain protein mRNA, complete cds
AT1G32950	1.153355533	0.001824691	Arabidopsis thaliana subtilase family protein mRNA, complete cds
AT1G44120	1.893596259	0.001869638	Arabidopsis thaliana armadillo/beta-catenin-like repeat and C2 calcium/lipid-binding domain-containing protein mRNA, complete cds
AT5G22540	1.893596259	0.001869638	Arabidopsis thaliana chromosome 5 sequence
AT5G54190	1.893596259	0.001869638	Arabidopsis thaliana protochlorophyllide reductase A mRNA, complete cds
AT5G62040	1.893596259	0.001869638	Arabidopsis thaliana protein BROTHER of FT and TFL 1 mRNA, complete cds
AT5G54470	1.086241337	0.00191960	Arabidopsis thaliana B-box type zinc finger-containing

		2	protein mRNA, complete cds
AT3G42800	1.616756054	0.001935466	Arabidopsis thaliana uncharacterized protein mRNA, complete cds
AT5G59580	1.616756054	0.001935466	Arabidopsis thaliana UDP-glucosyl transferase 76E1 mRNA, complete cds
AT3G54340	1.616756054	0.001935466	Arabidopsis thaliana Floral homeotic protein APETALA 3 mRNA, complete cds
AT5G36740	2.786681055	0.00203635	Arabidopsis thaliana Acyl-CoA N-acyltransferase domain-containing protein mRNA, complete cds
ATMG00730	2.786681055	0.00203635	Arabidopsis thaliana mitochondrial genome
AT1G67370	1.246706009	0.002061184	Arabidopsis thaliana asynaptic 1 mRNA, complete cds
AT5G38130	2.012240755	0.002071598	Arabidopsis thaliana chromosome 5 sequence
AT1G71420	1.484790713	0.00209627	Arabidopsis thaliana chromosome 1 sequence
AT1G32970	1.484790713	0.00209627	Arabidopsis thaliana Subtilisin-like serine endopeptidase family protein mRNA, complete cds
AT5G22555	1.118662815	0.002096505	Arabidopsis thaliana uncharacterized protein mRNA, complete cds
AT3G47790	1.057094991	0.002170261	Arabidopsis thaliana ABC transporter A family member 8 mRNA, complete cds
AT2G34610	2.173704178	0.002212651	Arabidopsis thaliana chromosome 2, complete sequence
AT5G47130	2.408169432	0.002230279	Arabidopsis thaliana Bax inhibitor-1 family protein mRNA, complete cds
AT1G22260	2.408169432	0.002230279	Arabidopsis thaliana synaptonemal complex protein ZYP1a mRNA, complete cds
AT3G05550	2.408169432	0.002230279	Arabidopsis thaliana Hypoxia-responsive family protein mRNA, complete cds
AT5G54570	2.408169432	0.002230279	Arabidopsis thaliana beta glucosidase 41 mRNA, complete cds
AT1G21320	2.408169432	0.002230279	Arabidopsis thaliana nucleic acid/nucleotide binding protein mRNA, complete cds
AT3G19230	1.671203838	0.002290419	Arabidopsis thaliana leucine-rich repeat-containing protein mRNA, complete cds
AT4G36700	4.256166338	0.002373731	Arabidopsis thaliana cupin family protein mRNA, complete cds
AT3G10100	4.256166338	0.002373731	Arabidopsis thaliana At4g20730 mRNA, complete cds
AT1G75790	4.256166338	0.002373731	Arabidopsis thaliana SKU5 similar 18 protein mRNA, complete cds
AT3G55820	1.086241337	0.002388852	Arabidopsis thaliana chromosome 3, complete sequence

AT4G13992	1.519200744	0.002536066	Arabidopsis thaliana chromosome 4 sequence
AT1G51670	1.256166338	0.002553761	Arabidopsis thaliana uncharacterized protein mRNA, complete cds
AT2G34850	1.323280534	0.002633189	Arabidopsis thaliana putative UDP-arabinose 4-epimerase 2 mRNA, complete cds
AT1G48580	1.408169432	0.002638107	Arabidopsis thaliana uncharacterized protein mRNA, complete cds
AT4G30430	1.738318033	0.002681735	Arabidopsis thaliana tetraspanin9 mRNA, complete cds
AT1G76610	1.738318033	0.002681735	Arabidopsis thaliana chromosome 1 sequence
AT3G03660	3.408169432	0.002966598	Arabidopsis thaliana WUSCHEL related homeobox 11 mRNA, complete cds
AT5G62080	3.408169432	0.002966598	Arabidopsis thaliana protease inhibitor/seed storage/lipid transfer protein (LTP) family protein mRNA, complete cds
ATCG00770	1.560172525	0.003053712	Arabidopsis thaliana chloroplast DNA, complete genome, ecotype: Columbia
AT1G51400	1.560172525	0.003053712	Arabidopsis thaliana chromosome 1 sequence
AT1G23450	1.823206931	0.003093637	Arabidopsis thaliana pentatricopeptide repeat-containing protein mRNA, complete cds
AT5G47850	1.823206931	0.003093637	Arabidopsis thaliana chromosome 5 sequence
AT1G14470	1.266813582	0.003164417	Arabidopsis thaliana chromosome 1 sequence
ATCG00170	1.266813582	0.003164417	Arabidopsis thaliana chloroplast DNA, complete genome, ecotype: Columbia
AT4G31070	1.43416464	0.003225366	Arabidopsis thaliana chromosome 4 sequence
AT3G02310	1.43416464	0.003225366	Arabidopsis thaliana developmental protein SEPALLATA 2 mRNA, complete cds
AT3G49142	1.339997929	0.00324708	Arabidopsis thaliana putative pentatricopeptide repeat-containing protein mRNA, complete cds
AT5G43980	1.121865247	0.003249647	Arabidopsis thaliana plasmodesmata-located protein 1 mRNA, complete cds
AT3G50040	1.054532477	0.003365013	Arabidopsis thaliana uncharacterized protein mRNA, complete cds
AT4G25540	1.054532477	0.003365013	Arabidopsis thaliana DNA mismatch repair protein MSH3 mRNA, complete cds
AT2G37580	1.000511463	0.003385591	Arabidopsis thaliana chromosome 2, complete sequence
AT4G38410	1.164243849	0.00351294	Arabidopsis thaliana putative dehydrin mRNA, complete

		7	cds
AT5G64990	1.609803293	0.00365382 9	Arabidopsis thaliana RAB GTPase homolog H1A mRNA, complete cds
AT5G66607	1.215524354	0.00374592 1	Arabidopsis thaliana chromosome 5 sequence
AT5G65600	1.215524354	0.00374592 1	Arabidopsis thaliana chromosome 5 sequence
AT5G50790	1.215524354	0.00374592 1	Arabidopsis thaliana bidirectional sugar transporter SWEET10 mRNA, complete cds
AT3G24230	2.671203838	0.00374999 8	Arabidopsis thaliana putative pectate lyase 9 mRNA, complete cds
AT1G09930	2.671203838	0.00374999 8	Arabidopsis thaliana oligopeptide transporter 2 mRNA, complete cds
AT3G09550	1.024840792	0.00377887 7	Arabidopsis thaliana ankyrin repeat-containing protein mRNA, complete cds
AT2G33847	2.086241337	0.00381834 9	Arabidopsis thaliana uncharacterized protein mRNA, complete cds
AT4G26200	1.46475296	0.00393320 4	Arabidopsis thaliana 1-aminocyclopropane-1-carboxylate synthase 7 mRNA, complete cds
AT4G08115	1.46475296	0.00393320 4	Arabidopsis thaliana chromosome 4 sequence
AT4G03292	2.308633758	0.00396151 7	Arabidopsis thaliana chromosome 4 sequence
AT3G04050	2.308633758	0.00396151 7	Arabidopsis thaliana pyruvate kinase mRNA, complete cds
AT4G21323	1.359259831	0.00400085 6	Arabidopsis thaliana Subtilase family protein mRNA, complete cds
AT5G52800	1.123716042	0.00405035 4	Arabidopsis thaliana DNA primase mRNA, complete cds
AT3G14225	1.123716042	0.00405035 4	Arabidopsis thaliana GDSL esterase/lipase 4 mRNA, complete cds
AT1G36622	1.671203838	0.00433403 2	Arabidopsis thaliana uncharacterized protein mRNA, complete cds
AT3G47830	1.168703497	0.00437597	Arabidopsis thaliana DNA glycosylase superfamily protein mRNA, complete cds
AT2G36660	1.168703497	0.00437597	Arabidopsis thaliana poly(A) binding protein 7 mRNA, complete cds
AT2G07711	1.168703497	0.00437597	Arabidopsis thaliana chromosome 2, complete sequence
AT1G16705	4.086241337	0.00461134 7	Arabidopsis thaliana p300/CBP acetyltransferase-related protein mRNA, complete cds
AT2G02340	4.086241337	0.00461134 7	Arabidopsis thaliana phloem protein 2-B8 mRNA, complete cds
AT2G07682	4.086241337	0.00461134 7	Arabidopsis thaliana chromosome 2, complete sequence

AT5G53230	4.086241337	0.004611347	Arabidopsis thaliana chromosome 5 sequence
AT1G26410	4.086241337	0.004611347	Arabidopsis thaliana chromosome 1 sequence
AT4G05540	4.086241337	0.004611347	Arabidopsis thaliana P-loop containing nucleoside triphosphate hydrolases superfamily protein mRNA, complete cds
AT2G07773	4.086241337	0.004611347	Arabidopsis thaliana chromosome 2, complete sequence
AT1G36600	4.086241337	0.004611347	Arabidopsis thaliana chromosome 1 sequence
AT1G30170	4.086241337	0.004611347	Arabidopsis thaliana uncharacterized protein mRNA, complete cds
AT1G36620	4.086241337	0.004611347	Arabidopsis thaliana chromosome 1 sequence
AT5G45150	1.086241337	0.004624517	Arabidopsis thaliana RNAse THREE-like protein 3 mRNA, complete cds
AT1G77390	1.022110999	0.004710555	Arabidopsis thaliana cyclin-A1-2 mRNA, complete cds
AT4G11480	1.501278836	0.004779731	Arabidopsis thaliana putative cysteine-rich receptor-like protein kinase 32 mRNA, complete cds
AT5G24180	1.501278836	0.004779731	Arabidopsis thaliana lipase class 3-related protein mRNA, complete cds
AT3G24929	1.501278836	0.004779731	Arabidopsis thaliana uncharacterized protein mRNA, complete cds
AT4G15350	1.292692214	0.004858998	Arabidopsis thaliana cytochrome P450, family 705, subfamily A, polypeptide 2 mRNA, complete cds
AT3G27730	1.38169722	0.004924007	Arabidopsis thaliana DNA helicase mRNA, complete cds
AT5G55340	1.125769701	0.005052555	Arabidopsis thaliana chromosome 5 sequence
AT2G03130	3.256166338	0.005694551	Arabidopsis thaliana chromosome 2, complete sequence
AT4G21490	3.256166338	0.005694551	Arabidopsis thaliana NAD(P)H dehydrogenase B3 mRNA, complete cds
AT2G22760	3.256166338	0.005694551	Arabidopsis thaliana transcription factor bHLH19 mRNA, complete cds
AT3G30120	3.256166338	0.005694551	Arabidopsis thaliana chromosome 3, complete sequence
AT1G32510	3.256166338	0.005694551	Arabidopsis thaliana NAC domain containing protein 11 mRNA, complete cds
AT2G18720	3.256166338	0.005694551	Arabidopsis thaliana translation elongation factor EF1A/initiation factor IF2gamma family protein mRNA, complete cds

AT4G19560	1.086241337	0.005773462	Arabidopsis thaliana Cyclin-T1-2 mRNA, complete cds
AT4G16910	1.545672955	0.005780773	Arabidopsis thaliana chromosome 4 sequence
AT1G74400	1.545672955	0.005780773	Arabidopsis thaliana chromosome 1 sequence
AT2G30760	1.233082725	0.005795521	Arabidopsis thaliana uncharacterized protein mRNA, complete cds
AT2G17210	1.233082725	0.005795521	Arabidopsis thaliana pentatricopeptide repeat-containing protein mRNA, complete cds
AT5G52720	1.851776083	0.005843179	Arabidopsis thaliana copper transport family protein mRNA, complete cds
AT4G04710	1.851776083	0.005843179	Arabidopsis thaliana calcium-dependent protein kinase 22 mRNA, complete cds
AT2G33100	1.851776083	0.005843179	Arabidopsis thaliana cellulose synthase-like protein D1 mRNA, complete cds
AT1G16360	1.851776083	0.005843179	Arabidopsis thaliana ligand-effect modulator 3 family protein / CDC50 family protein mRNA, complete cds
AT2G42990	1.308633758	0.006019884	Arabidopsis thaliana GDSL esterase/lipase mRNA, complete cds
AT5G65350	1.408169432	0.006050479	Arabidopsis thaliana chromosome 5 sequence
AT1G11460	1.408169432	0.006050479	Arabidopsis thaliana nodulin MtN21-like transporter family protein mRNA, complete cds
AT3G18610	1.128061513	0.006308451	Arabidopsis thaliana protein NUCLEOLIN LIKE 2 mRNA, complete cds
AT1G02230	1.128061513	0.006308451	Arabidopsis thaliana NAC domain-containing protein 4 mRNA, complete cds
AT5G63390	1.993131932	0.006539016	Arabidopsis thaliana O-fucosyltransferase family protein mRNA, complete cds
AT2G04495	1.179350741	0.006805655	Arabidopsis thaliana chromosome 2, complete sequence
AT2G44590	2.545672955	0.006875568	Arabidopsis thaliana dynamin-related protein 1d mRNA, complete cds
AT2G04033	2.545672955	0.006875568	Arabidopsis thaliana clone asmbl_4915 defensin-like mRNA sequence
AT2G17490	2.545672955	0.006875568	Arabidopsis thaliana chromosome 2, complete sequence
AT3G24340	1.60081451	0.006944725	Arabidopsis thaliana chromatin remodeling 40 mRNA, complete cds
AT5G01570	1.60081451	0.006944725	Arabidopsis thaliana uncharacterized protein mRNA, complete cds
AT1G58390	2.201718554	0.006989613	Arabidopsis thaliana CC-NBS-LRR class disease resistance protein mRNA, complete cds

AT5G61850	2.201718554	0.006989613	Arabidopsis thaliana protein LEAFY mRNA, complete cds
AT2G01510	1.086241337	0.007215008	Arabidopsis thaliana chromosome 2, complete sequence
AT1G61255	1.439878291	0.007417857	Arabidopsis thaliana chromosome 1 sequence
AT4G25850	1.327249436	0.007455372	Arabidopsis thaliana OSBP(oxysterol binding protein)-related protein 4B mRNA, complete cds
ATMG00160	1.130635456	0.007884329	Raphanus sativus cytochrome c oxidase subunit II (cox2) mRNA, mitochondrial mRNA encoding mitochondrial protein, complete cds
AT3G18670	1.047767189	0.008171182	Arabidopsis thaliana ankyrin repeat-containing protein mRNA, complete cds
AT4G16270	1.671203838	0.008262529	Arabidopsis thaliana peroxidase 40 mRNA, complete cds
AT2G35075	1.671203838	0.008262529	Arabidopsis thaliana uncharacterized protein mRNA, complete cds
AT3G05790	1.671203838	0.008262529	Arabidopsis thaliana lon protease 4 mRNA, complete cds
AT4G38850	1.18577701	0.008497898	Arabidopsis thaliana chromosome 4 sequence
AT5G05365	1.18577701	0.008497898	Arabidopsis thaliana Heavy metal transport/detoxification superfamily protein mRNA, complete cds
AT5G50335	1.256166338	0.008982763	Arabidopsis thaliana chromosome 5 sequence
AT2G40350	3.893596259	0.009032786	Arabidopsis thaliana chromosome 2, complete sequence
AT3G26614	3.893596259	0.009032786	Arabidopsis thaliana chromosome 3, complete sequence
AT5G39330	3.893596259	0.009032786	Arabidopsis thaliana uncharacterized protein mRNA, complete cds
AT3G29190	3.893596259	0.009032786	Arabidopsis thaliana terpenoid synthase 15 mRNA, complete cds
AT5G52700	3.893596259	0.009032786	Arabidopsis thaliana copper transport family protein mRNA, complete cds
AT4G12500	3.893596259	0.009032786	Arabidopsis thaliana chromosome 4 sequence
AT1G71200	3.893596259	0.009032786	Arabidopsis thaliana basic helix-loop-helix (bHLH) DNA-binding superfamily protein mRNA, complete cds
AT1G43785	3.893596259	0.009032786	Arabidopsis thaliana chromosome 1 sequence
AT2G36750	3.893596259	0.009032786	Arabidopsis thaliana chromosome 2, complete sequence

AT3G53040	3.893596259	0.009032786	Arabidopsis thaliana putative late embryogenesis abundant protein mRNA, complete cds
AT1G62420	3.893596259	0.009032786	Arabidopsis thaliana uncharacterized protein mRNA, complete cds
AT4G07830	3.893596259	0.009032786	Arabidopsis thaliana chromosome 4 sequence
AT3G47875	1.47855876	0.009064878	Arabidopsis thaliana chromosome 3, complete sequence
AT1G61440	1.47855876	0.009064878	Arabidopsis thaliana G-type lectin S-receptor-like serine/threonine-protein kinase mRNA, complete cds
AT2G15800	1.47855876	0.009064878	Arabidopsis thaliana chromosome 2, complete sequence
AT3G44350	1.47855876	0.009064878	Arabidopsis thaliana putative NAC domain-containing protein 61 mRNA, complete cds
AT5G27230	1.47855876	0.009064878	Arabidopsis thaliana Frigida-like protein mRNA, complete cds
AT3G27660	1.012240755	0.009173386	Arabidopsis thaliana oleosin 4 mRNA, complete cds
AT3G28890	1.012240755	0.009173386	Arabidopsis thaliana receptor like protein 43 mRNA, complete cds
AT3G44765	1.349275743	0.009227144	Arabidopsis thaliana Full-length cDNA Complete sequence from clone GSLTPGH55ZA10 of Hormone Treated Callus of strain col-0 of Arabidopsis thaliana (thale cress)
AT2G25470	1.349275743	0.009227144	Arabidopsis thaliana receptor like protein 21 mRNA, complete cds
AT5G03060	1.764313242	0.009688367	Arabidopsis thaliana uncharacterized protein mRNA, complete cds
AT2G23550	1.045599352	0.010226406	Arabidopsis thaliana methyl esterase 6 mRNA, complete cds
AT4G23610	1.193156541	0.010620648	Arabidopsis thaliana chromosome 4 sequence
AT2G45350	1.193156541	0.010620648	Arabidopsis thaliana chlororespiratory reduction 4 protein mRNA, complete cds
AT2G14230	3.086241337	0.010942071	Arabidopsis thaliana chromosome 2, complete sequence
AT5G67411	3.086241337	0.010942071	Arabidopsis thaliana clone asmbl_17543 unknown mRNA sequence
AT4G15100	3.086241337	0.010942071	Arabidopsis thaliana serine carboxypeptidase-like 30 mRNA, complete cds
AT5G25045	3.086241337	0.010942071	Arabidopsis thaliana chromosome 5 sequence
AT1G07060	3.086241337	0.010942071	Arabidopsis thaliana protein DSB formation mRNA, complete cds

AT3G58480	3.086241337	0.010942071	Arabidopsis thaliana calmodulin-binding protein-like protein mRNA, complete cds
AT3G26820	3.086241337	0.010942071	Arabidopsis thaliana Esterase/lipase/thioesterase family protein mRNA, complete cds
AT3G48300	3.086241337	0.010942071	Arabidopsis thaliana cytochrome P450 71A23 mRNA, complete cds
AT3G03341	3.086241337	0.010942071	Arabidopsis thaliana chromosome 3, complete sequence
AT3G48840	3.086241337	0.010942071	Arabidopsis thaliana RNA-binding (RRM/RBD/RNP motifs) family protein mRNA, complete cds
AT5G38750	3.086241337	0.010942071	Arabidopsis thaliana asparaginyl-tRNA synthetase-like protein mRNA, complete cds
AT1G20180	3.086241337	0.010942071	Arabidopsis thaliana uncharacterized protein mRNA, complete cds
AT5G51950	1.526813928	0.011025508	Arabidopsis thaliana Glucose-methanol-choline (GMC) oxidoreductase family protein mRNA, complete cds
AT1G42980	1.526813928	0.011025508	Arabidopsis thaliana formin-like protein 12 mRNA, complete cds
AT2G44578	1.526813928	0.011025508	Arabidopsis thaliana chromosome 2, complete sequence
AT5G01760	1.526813928	0.011025508	Arabidopsis thaliana ENTH/VHS/GAT family protein mRNA, complete cds
AT3G50940	1.893596259	0.011102268	Arabidopsis thaliana cytochrome BC1 synthesis-like protein mRNA, complete cds
AT1G67270	1.893596259	0.011102268	Arabidopsis thaliana zinc-finger domain of monoamine-oxidase A repressor R1 protein mRNA, complete cds
AT5G50570	1.270665908	0.011189696	Arabidopsis thaliana squamosa promoter-binding-like protein 13 mRNA, complete cds
AT1G03540	1.270665908	0.011189696	Arabidopsis thaliana chromosome 1 sequence
AT4G20420	1.270665908	0.011189696	Arabidopsis thaliana chromosome 4 sequence
AT5G67245	1.086241337	0.011306009	Arabidopsis thaliana chromosome 5 sequence
AT5G25990	1.086241337	0.011306009	Arabidopsis thaliana uncharacterized protein mRNA, complete cds
ATCG00740	1.086241337	0.011306009	Arabidopsis thaliana chloroplast DNA, complete genome, ecotype: Columbia
AT1G31290	1.086241337	0.011306009	Arabidopsis thaliana argonaute 3 mRNA, complete cds
ATMG00090	1.086241337	0.011306009	Arabidopsis thaliana ecotype Landsberg erecta mitochondrion, complete genome
AT5G01380	1.375747954	0.01140771	Arabidopsis thaliana trihelix transcription factor GT-3a

			mRNA, complete cds
AT3G28153	2.086241337	0.012237159	Arabidopsis thaliana chromosome 3, complete sequence
AT5G39130	2.086241337	0.012237159	Arabidopsis thaliana germin-like protein subfamily 1 member 16 mRNA, complete cds
AT5G25390	2.408169432	0.012538405	Arabidopsis thaliana ethylene-responsive transcription factor SHINE 3 mRNA, complete cds
AT1G64310	2.408169432	0.012538405	Arabidopsis thaliana chromosome 1 sequence
AT5G67310	2.408169432	0.012538405	Arabidopsis thaliana cytochrome P450, family 81, subfamily G, polypeptide 1 mRNA, complete cds
AT1G33640	2.408169432	0.012538405	Arabidopsis thaliana uncharacterized protein mRNA, complete cds
AT1G78340	1.043172615	0.01281447	Arabidopsis thaliana glutathione S-transferase TAU 22 mRNA, complete cds
AT3G01580	1.043172615	0.01281447	Arabidopsis thaliana pentatricopeptide repeat-containing protein mRNA, complete cds
AT2G12460	1.043172615	0.01281447	Arabidopsis thaliana chromosome 2, complete sequence
AT2G31751	1.201718554	0.01328668	Arabidopsis thaliana clone asmbl_6700 unknown mRNA sequence
AT5G24206	1.201718554	0.01328668	Arabidopsis thaliana chromosome 5 sequence
AT5G46590	1.201718554	0.01328668	Arabidopsis thaliana NAC domain containing protein 96 mRNA, complete cds
AT4G26330	1.588741677	0.013316025	Arabidopsis thaliana protein UNFERTILIZED EMBRYO SAC 17 mRNA, complete cds
AT4G18960	1.588741677	0.013316025	Arabidopsis thaliana MADS domain transcription factor AGAMOUS mRNA, complete cds
AT4G39363	1.588741677	0.013316025	Arabidopsis thaliana chromosome 4 sequence
AT3G13840	1.287875198	0.013942379	Arabidopsis thaliana chromosome 3, complete sequence
AT1G29290	1.287875198	0.013942379	Arabidopsis thaliana chromosome 1 sequence
AT3G50030	1.408169432	0.014079368	Arabidopsis thaliana uncharacterized protein mRNA, complete cds
AT4G35590	1.408169432	0.014079368	Arabidopsis thaliana RWP-RK domain-containing protein mRNA, complete cds
AT3G15930	1.086241337	0.01418046	Arabidopsis thaliana pentatricopeptide repeat-containing protein mRNA, complete cds
AT3G45660	1.086241337	0.01418046	Arabidopsis thaliana probable nitrate excretion transporter 2 mRNA, complete cds
AT4G08100	1.086241337	0.01418046	Arabidopsis thaliana chromosome 4 sequence
AT1G08730	1.140689121	0.01549996	Arabidopsis thaliana myosin class XI mRNA, complete cds
AT1G49900	1.671203838	0.01590761	Arabidopsis thaliana C2H2 type zinc finger transcription

		3	factor-like protein mRNA, complete cds
AT5G55650	1.671203838	0.015907613	Arabidopsis thaliana chromosome 5 sequence
AT1G30660	1.671203838	0.015907613	Arabidopsis thaliana nucleic acid binding protein mRNA, complete cds
AT4G17380	1.211772219	0.016639336	Arabidopsis thaliana DNA mismatch repair protein MSH4 mRNA, complete cds
AT2G17740	1.448811416	0.017329341	Arabidopsis thaliana chromosome 2, complete sequence
AT1G52920	1.308633758	0.01737341	Arabidopsis thaliana G-protein coupled receptor 2 mRNA, complete cds
AT4G20970	1.308633758	0.01737341	Arabidopsis thaliana basic helix-loop-helix domain-containing protein mRNA, complete cds
AT3G21520	1.308633758	0.01737341	Arabidopsis thaliana chromosome 3, complete sequence
AT4G04450	1.086241337	0.017812453	Arabidopsis thaliana putative WRKY transcription factor 42 mRNA, complete cds
AT2G31210	3.671203838	0.017854353	Arabidopsis thaliana transcription factor bHLH91 mRNA, complete cds
AT4G25510	3.671203838	0.017854353	Arabidopsis thaliana uncharacterized protein mRNA, complete cds
AT5G45830	3.671203838	0.017854353	Arabidopsis thaliana protein DELAY OF GERMINATION 1 mRNA, complete cds
AT5G28310	3.671203838	0.017854353	Arabidopsis thaliana NAD(P)-binding Rossmann-fold superfamily protein mRNA, complete cds
AT2G29100	3.671203838	0.017854353	Arabidopsis thaliana glutamate receptor 2.9 mRNA, complete cds
AT1G31570	3.671203838	0.017854353	Arabidopsis thaliana chromosome 1 sequence
AT2G22821	3.671203838	0.017854353	Arabidopsis thaliana chromosome 2, complete sequence
AT4G08109	3.671203838	0.017854353	Arabidopsis thaliana chromosome 4 sequence
AT5G55420	3.671203838	0.017854353	Arabidopsis thaliana chromosome 5 sequence
AT3G60966	1.786681055	0.018667854	Arabidopsis thaliana chromosome 3, complete sequence
ATMG01200	1.786681055	0.018667854	Arabidopsis thaliana ecotype Landsberg erecta mitochondrion, complete genome
AT4G14180	1.786681055	0.018667854	Arabidopsis thaliana protein PRD1 mRNA, complete cds
AT4G14700	1.145135026	0.0194711	Arabidopsis thaliana chromosome 4 sequence
AT4G25410	1.037331736	0.020208327	Arabidopsis thaliana transcription factor bHLH126 mRNA, complete cds
AT1G48870	1.037331736	0.02020832	Arabidopsis thaliana WD40 domain-containing protein

		7	mRNA, complete cds
AT3G13640	1.223744861	0.02086100 6	Arabidopsis thaliana ABC transporter E family member 1 mRNA, complete cds
AT4G03540	2.893596259	0.02103340 8	Arabidopsis thaliana uncharacterized protein mRNA, complete cds
AT5G05490	2.893596259	0.02103340 8	Arabidopsis thaliana RAD21-like protein SYN1 mRNA, complete cds
AT2G16210	2.893596259	0.02103340 8	Arabidopsis thaliana B3 domain-containing protein mRNA, complete cds
AT5G54165	2.893596259	0.02103340 8	Arabidopsis thaliana chromosome 5 sequence
AT4G20920	2.893596259	0.02103340 8	Arabidopsis thaliana double-stranded RNA-binding domain (DsRBD)-containing protein mRNA, complete cds
AT5G53895	2.893596259	0.02103340 8	Arabidopsis thaliana uncharacterized protein mRNA, complete cds
AT3G13130	2.893596259	0.02103340 8	Arabidopsis thaliana chromosome 3, complete sequence
ATMG0066 5	2.893596259	0.02103340 8	.
AT5G13940	2.893596259	0.02103340 8	Arabidopsis thaliana aminopeptidase mRNA, complete cds
AT2G45135	2.893596259	0.02103340 8	Arabidopsis thaliana RING/U-box family protein mRNA, complete cds
AT3G44805	2.893596259	0.02103340 8	Arabidopsis thaliana TRAF-like family protein mRNA, complete cds
AT5G19151	1.960710455	0.02123247 2	Arabidopsis thaliana uncharacterized protein mRNA, complete cds
AT2G30670	1.960710455	0.02123247 2	Arabidopsis thaliana NAD(P)-binding Rossmann-fold superfamily protein mRNA, complete cds
AT1G71280	1.960710455	0.02123247 2	Arabidopsis thaliana DEAD-box ATP-dependent RNA helicase 55 mRNA, complete cds
ATCG00360	1.501278836	0.02123626 1	Arabidopsis lyrata subsp. lyrata predicted protein, mRNA
AT2G27220	1.501278836	0.02123626 1	Arabidopsis thaliana BEL1-like homeodomain 5 mRNA, complete cds
AT2G34240	1.501278836	0.02123626 1	Arabidopsis thaliana uncharacterized protein mRNA, complete cds
AT3G58150	1.501278836	0.02123626 1	Arabidopsis thaliana Optic atrophy 3 protein (OPA3) mRNA, complete cds
AT2G26380	1.33416885	0.02164342 3	Arabidopsis thaliana chromosome 2, complete sequence
AT5G02570	1.33416885	0.02164342 3	Arabidopsis thaliana chromosome 5 sequence
ATMG0136	1.086241337	0.02241262	Arabidopsis thaliana ecotype Landsberg erecta

0		9	mitochondrion, complete genome
AT5G01730	1.086241337	0.022412629	Arabidopsis thaliana protein SCAR4 mRNA, complete cds
AT1G02530	2.256166338	0.022713134	Arabidopsis thaliana P-glycoprotein 12 mRNA, complete cds
ATMG00630	2.256166338	0.022713134	Arabidopsis thaliana ecotype Landsberg erecta mitochondrion, complete genome
AT2G27630	2.256166338	0.022713134	Arabidopsis thaliana ubiquitin carboxyl-terminal hydrolase-related protein mRNA, complete cds
AT1G47930	2.256166338	0.022713134	Arabidopsis thaliana chromosome 1 sequence
AT5G44575	2.256166338	0.022713134	Arabidopsis thaliana uncharacterized protein mRNA, complete cds
AT1G24577	2.256166338	0.022713134	Arabidopsis thaliana chromosome 1 sequence
AT3G62510	1.150371674	0.024500461	Arabidopsis thaliana protein disulfide isomerase-like protein mRNA, complete cds
AT4G16892	1.033773917	0.025441438	Arabidopsis thaliana clone asmbl_12323 unknown mRNA sequence
AT4G20160	1.571668164	0.025836838	Arabidopsis thaliana uncharacterized protein mRNA, complete cds
AT2G29480	1.571668164	0.025836838	Arabidopsis thaliana glutathione S-transferase tau 2 mRNA, complete cds
AT2G21450	1.571668164	0.025836838	Arabidopsis thaliana chromatin remodeling 34 mRNA, complete cds
AT1G76640	1.571668164	0.025836838	Arabidopsis thaliana chromosome 1 sequence
ATMG01220	1.23824443	0.026183821	Arabidopsis thaliana ecotype Landsberg erecta mitochondrion, complete genome
AT3G16610	1.23824443	0.026183821	Arabidopsis thaliana chromosome 3, complete sequence
AT1G08630	1.23824443	0.026183821	Arabidopsis thaliana threonine aldolase mRNA, complete cds
AT3G60470	1.366349256	0.026941705	Arabidopsis thaliana uncharacterized protein mRNA, complete cds
AT3G53150	1.366349256	0.026941705	Arabidopsis thaliana chromosome 3, complete sequence
AT5G13230	1.366349256	0.026941705	Arabidopsis thaliana chromosome 5 sequence
AT3G21755	1.366349256	0.026941705	Arabidopsis thaliana clone 151422 mRNA sequence
AT3G01840	1.086241337	0.028255201	Arabidopsis thaliana LysM-containing receptor-like kinase mRNA, complete cds
AT2G02240	1.156630665	0.03088699	Arabidopsis thaliana F-box domain protein MEE66 mRNA,

		8	complete cds
AT1G48660	1.671203838	0.03104548 9	Arabidopsis thaliana auxin-responsive GH3 family protein mRNA, complete cds
AT2G07787	1.671203838	0.03104548 9	Arabidopsis thaliana uncharacterized protein mRNA, complete cds
AT5G60180	1.671203838	0.03104548 9	Arabidopsis thaliana chromosome 5 sequence
AT5G64395	1.671203838	0.03104548 9	Arabidopsis thaliana chromosome 5 sequence
AT1G05894	1.671203838	0.03104548 9	Arabidopsis thaliana chromosome 1 sequence
AT4G19970	1.029657808	0.03209357	Arabidopsis thaliana uncharacterized protein mRNA, complete cds
AT5G67050	1.256166338	0.03290275 7	Arabidopsis thaliana alpha/beta-Hydrolases superfamily protein mRNA, complete cds
AT2G30432	1.256166338	0.03290275 7	Arabidopsis thaliana protein trichomeless 1 mRNA, complete cds
AT4G37430	1.408169432	0.03347886 5	Arabidopsis thaliana cytochrome P450 81F1 mRNA, complete cds
AT3G10590	1.408169432	0.03347886 5	Arabidopsis thaliana SANT DNA-binding domain-containing protein mRNA, complete cds
AT3G13900	1.408169432	0.03347886 5	Arabidopsis thaliana putative phospholipid-transporting ATPase 7 mRNA, complete cds
AT3G17110	3.408169432	0.03564623 5	Arabidopsis thaliana chromosome 3, complete sequence
AT5G14565	3.408169432	0.03564623 5	Arabidopsis thaliana mRNA for hypothetical protein, complete cds, clone: RAFL21-89-F22
AT4G09430	3.408169432	0.03564623 5	Arabidopsis thaliana TIR-NBS-LRR class disease resistance protein mRNA, complete cds
AT2G21910	3.408169432	0.03564623 5	Arabidopsis thaliana chromosome 2, complete sequence
AT1G32910	3.408169432	0.03564623 5	Arabidopsis thaliana HXXXD-type acyl-transferase-like protein mRNA, complete cds
AT5G26700	3.408169432	0.03564623 5	Arabidopsis thaliana putative germin-like protein subfamily 2 member 5 mRNA, complete cds
AT2G15070	3.408169432	0.03564623 5	Arabidopsis thaliana chromosome 2, complete sequence
AT2G42247	3.408169432	0.03564623 5	Arabidopsis thaliana Full-length cDNA Complete sequence from clone GSLTFB53ZC11 of Flowers and buds of strain col-0 of Arabidopsis thaliana (thale cress)
AT1G21202	3.408169432	0.03564623 5	Arabidopsis thaliana chromosome 1 sequence
AT5G07520	3.408169432	0.03564623 5	Arabidopsis thaliana glycine-rich protein 18 mRNA, complete cds

AT1G66870	3.408169432	0.035646235	Arabidopsis thaliana carbohydrate-binding X8 domain-containing protein mRNA, complete cds
AT1G33830	3.408169432	0.035646235	Arabidopsis thaliana P-loop containing nucleoside triphosphate hydrolases superfamily protein mRNA, complete cds
AT5G41755	3.408169432	0.035646235	Arabidopsis thaliana chromosome 5 sequence
AT2G07697	3.408169432	0.035646235	Arabidopsis thaliana chromosome 2, complete sequence
AT3G56790	3.408169432	0.035646235	Arabidopsis thaliana chromosome 3, complete sequence
AT2G35070	3.408169432	0.035646235	Arabidopsis thaliana uncharacterized protein mRNA, complete cds
AT2G14245	3.408169432	0.035646235	Arabidopsis thaliana chromosome 2, complete sequence
AT5G64540	3.408169432	0.035646235	Arabidopsis thaliana uncharacterized protein mRNA, complete cds
AT3G56920	3.408169432	0.035646235	Arabidopsis thaliana putative S-acyltransferase mRNA, complete cds
AT5G14980	3.408169432	0.035646235	Arabidopsis thaliana chromosome 5 sequence
AT1G66550	3.408169432	0.035646235	Arabidopsis thaliana putative WRKY transcription factor 67 mRNA, complete cds
AT1G61110	3.408169432	0.035646235	Arabidopsis thaliana NAC domain containing protein 25 mRNA, complete cds
AT1G60720	3.408169432	0.035646235	Arabidopsis thaliana chromosome 1 sequence
AT1G64405	1.086241337	0.035699692	Arabidopsis thaliana chromosome 1 sequence
AT2G21460	1.823206931	0.03645484	Arabidopsis thaliana chromosome 2, complete sequence
AT2G30380	1.823206931	0.03645484	Arabidopsis thaliana uncharacterized protein mRNA, complete cds
AT4G36740	1.823206931	0.03645484	Arabidopsis thaliana homeobox protein 40 mRNA, complete cds
AT4G10580	1.823206931	0.03645484	Arabidopsis thaliana chromosome 4 sequence
AT1G52430	1.823206931	0.03645484	Arabidopsis thaliana ubiquitin carboxyl-terminal hydrolase-related protein mRNA, complete cds
AT1G77405	1.164243849	0.039021806	Arabidopsis thaliana chromosome 1 sequence
AT1G64260	1.164243849	0.039021806	Arabidopsis thaliana chromosome 1 sequence
AT5G04000	2.671203838	0.04041122	Arabidopsis thaliana uncharacterized protein mRNA, complete cds
AT1G61290	2.671203838	0.04041122	Arabidopsis thaliana syntaxin-124 mRNA, complete cds

AT1G66170	2.671203838	0.04041122	Arabidopsis thaliana PHD finger protein MALE MEIOCYTE DEATH 1 mRNA, complete cds
AT1G18350	2.671203838	0.04041122	Arabidopsis thaliana chromosome 1 sequence
AT3G26235	2.671203838	0.04041122	Arabidopsis thaliana uncharacterized protein mRNA, complete cds
AT1G72000	2.671203838	0.04041122	Arabidopsis thaliana putative invertase mRNA, complete cds
AT5G43570	2.671203838	0.04041122	Arabidopsis thaliana PR-6 proteinase inhibitor family protein mRNA, complete cds
AT1G45240	2.671203838	0.04041122	Theobroma cacao Uncharacterized protein (TCM_007238) mRNA, complete cds
ATCG00160	2.671203838	0.04041122	Arabidopsis thaliana chloroplast DNA, complete genome, ecotype: Columbia
AT1G03710	2.671203838	0.04041122	Arabidopsis thaliana cystatin/monellin-related protein mRNA, complete cds
AT1G63960	2.671203838	0.04041122	Arabidopsis thaliana chromosome 1 sequence
ATMG00060	2.671203838	0.04041122	Arabidopsis thaliana NADH dehydrogenase subunit 5 mRNA, complete cds; mitochondrial
AT3G13433	2.671203838	0.04041122	Arabidopsis thaliana uncharacterized protein mRNA, complete cds
AT4G32205	2.671203838	0.04041122	Arabidopsis thaliana clone asmbl_13272 unknown mRNA sequence
AT4G10265	2.671203838	0.04041122	Arabidopsis thaliana chromosome 4 sequence
AT4G04480	2.671203838	0.04041122	Arabidopsis thaliana chromosome 4 sequence
AT4G05380	2.671203838	0.04041122	Arabidopsis thaliana AAA-type ATPase family protein mRNA, complete cds
AT2G07734	2.671203838	0.04041122	Arabidopsis thaliana chromosome 2, complete sequence
AT5G64790	2.671203838	0.04041122	Arabidopsis thaliana O-Glycosyl hydrolases family 17 protein mRNA, complete cds
AT4G25631	2.671203838	0.04041122	Arabidopsis thaliana chromosome 4 sequence
AT2G41230	2.671203838	0.04041122	Arabidopsis thaliana chromosome 2, complete sequence
AT3G53250	2.671203838	0.04041122	Arabidopsis thaliana chromosome 3, complete sequence
AT1G51840	2.671203838	0.04041122	Arabidopsis thaliana protein kinase-related protein mRNA, complete cds
AT1G28890	2.671203838	0.04041122	Theobroma cacao Uncharacterized protein (TCM_007238) mRNA, complete cds
AT1G66610	2.671203838	0.04041122	Arabidopsis thaliana E3 ubiquitin-protein ligase SINA-like 1 mRNA, complete cds
AT2G35200	2.086241337	0.040804401	Arabidopsis thaliana chromosome 2, complete sequence
AT2G28085	2.086241337	0.040804401	Arabidopsis thaliana chromosome 2, complete sequence
AT3G60970	2.086241337	0.040804401	Arabidopsis thaliana putative ABC transporter C-15 mRNA, complete cds

AT4G18540	2.086241337	0.040804401	Arabidopsis thaliana uncharacterized protein mRNA, complete cds
AT2G07706	2.086241337	0.040804401	Arabidopsis thaliana uncharacterized protein mRNA, complete cds
AT4G28790	2.086241337	0.040804401	Arabidopsis thaliana transcription factor bHLH23 mRNA, complete cds
AT2G44383	2.086241337	0.040804401	Arabidopsis thaliana chromosome 2, complete sequence
AT5G34825	2.086241337	0.040804401	Arabidopsis thaliana chromosome 5 sequence
AT1G04445	2.086241337	0.040804401	Arabidopsis thaliana chromosome 1 sequence
AT2G21040	2.086241337	0.040804401	Arabidopsis thaliana C2 domain-containing protein mRNA, complete cds
AT1G64110	2.086241337	0.040804401	Arabidopsis thaliana transcription factor DUO1 mRNA, complete cds
AT2G02930	1.278886415	0.041390614	Arabidopsis thaliana glutathione S-transferase F3 mRNA, complete cds
AT1G58460	1.278886415	0.041390614	Arabidopsis thaliana uncharacterized protein mRNA, complete cds
AT4G21970	1.278886415	0.041390614	Arabidopsis thaliana uncharacterized protein mRNA, complete cds
AT5G41280	1.278886415	0.041390614	Arabidopsis thaliana cysteine-rich repeat secretory protein 57 mRNA, complete cds
AT1G32570	1.46475296	0.041457965	Arabidopsis thaliana uncharacterized protein mRNA, complete cds
AT5G08030	1.46475296	0.041457965	Arabidopsis thaliana glycerophosphoryl diester phosphodiesterase mRNA, complete cds
AT4G33905	1.46475296	0.041457965	Arabidopsis thaliana Peroxisomal membrane protein Mpv17/PMP22 mRNA, complete cds
AT1G10540	1.086241337	0.045221594	Arabidopsis thaliana nucleobase-ascorbate transporter 8 mRNA, complete cds
AT5G49620	1.086241337	0.045221594	Arabidopsis thaliana myb domain protein 78 mRNA, complete cds
AT3G04440	1.086241337	0.045221594	Arabidopsis thaliana plasma-membrane choline transporter family protein mRNA, complete cds
AT5G61920	1.173704178	0.049420292	Arabidopsis thaliana protein FLX-like 4 mRNA, complete cds
AT3G25880	1.545672955	0.050987043	Arabidopsis thaliana NAD(P)-binding Rossmann-fold superfamily protein mRNA, complete cds
AT1G36640	1.545672955	0.050987043	Arabidopsis thaliana uncharacterized protein mRNA, complete cds
AT1G69880	1.545672955	0.050987043	Arabidopsis thaliana thioredoxin H8 mRNA, complete cds

AT1G62835	1.545672955	0.050987043	Arabidopsis thaliana chromosome 1 sequence
AT2G07667	1.545672955	0.050987043	Arabidopsis thaliana uncharacterized protein mRNA, complete cds
AT1G28920	1.545672955	0.050987043	Theobroma cacao Uncharacterized protein (TCM_007238) mRNA, complete cds
AT5G52220	1.019127141	0.05144176	Arabidopsis thaliana chromosome transmission fidelity protein 8 domain-containing protein mRNA, complete cds
AT1G16120	1.019127141	0.05144176	Arabidopsis thaliana wall-associated receptor kinase-like 1 mRNA, complete cds
AT2G07708	1.019127141	0.05144176	Arabidopsis thaliana chromosome 2, complete sequence
AT5G48050	1.308633758	0.052112043	Arabidopsis thaliana chromosome 5 sequence
AT4G37410	1.308633758	0.052112043	Arabidopsis thaliana cytochrome P450, family 81, subfamily F, polypeptide 4 mRNA, complete cds
AT5G04610	1.086241337	0.057456598	Arabidopsis thaliana chromosome 5 sequence
AT5G18633	1.671203838	0.061821433	Arabidopsis thaliana chromosome 5 sequence
AT1G05560	1.671203838	0.061821433	Arabidopsis thaliana chromosome 1 sequence
AT2G24870	1.671203838	0.061821433	Arabidopsis thaliana chromosome 2, complete sequence
AT1G10385	1.671203838	0.061821433	Arabidopsis thaliana Vps51/Vps67 family (components of vesicular transport) protein mRNA, complete cds
AT1G03800	1.671203838	0.061821433	Arabidopsis thaliana chromosome 1 sequence
AT5G48175	1.671203838	0.061821433	Arabidopsis thaliana uncharacterized protein mRNA, complete cds
AT4G31351	1.671203838	0.061821433	Arabidopsis thaliana chromosome 4 sequence
AT5G42230	1.671203838	0.061821433	Arabidopsis thaliana serine carboxypeptidase-like 41 mRNA, complete cds
AT3G14950	1.671203838	0.061821433	Arabidopsis thaliana TPR repeat-containing thioredoxin TTL2 mRNA, complete cds
AT5G66150	1.671203838	0.061821433	Arabidopsis thaliana Glycosyl hydrolase family protein mRNA, complete cds
AT4G27810	1.671203838	0.061821433	Arabidopsis thaliana uncharacterized protein mRNA, complete cds
ATMG00640	1.671203838	0.061821433	A.thaliana mitochondrial DNA for genes nad4L and orf25
AT2G07798	1.671203838	0.061821433	Arabidopsis thaliana uncharacterized protein mRNA, complete cds

AT3G25905	1.18577701	0.062767433	Arabidopsis thaliana chromosome 3, complete sequence
AT1G29000	1.18577701	0.062767433	Arabidopsis thaliana heavy-metal-associated domain-containing protein mRNA, complete cds
AT3G58770	1.012240755	0.065419178	Arabidopsis thaliana uncharacterized protein mRNA, complete cds
AT4G37780	1.012240755	0.065419178	Arabidopsis thaliana myb domain protein 87 mRNA, complete cds
AT1G09950	1.012240755	0.065419178	Arabidopsis thaliana chromosome 1 sequence
AT2G27610	1.012240755	0.065419178	Arabidopsis thaliana chromosome 2, complete sequence
AT4G36770	1.012240755	0.065419178	Arabidopsis thaliana chromosome 4 sequence
AT1G66690	1.012240755	0.065419178	Arabidopsis thaliana S-adenosyl-L-methionine-dependent methyltransferases superfamily protein mRNA, complete cds
AT5G56300	1.349275743	0.065626345	Arabidopsis thaliana gibberellic acid methyltransferase 2 mRNA, complete cds
AT3G46150	1.893596259	0.072546774	Arabidopsis thaliana uncharacterized protein mRNA, complete cds
AT2G33000	1.893596259	0.072546774	Arabidopsis thaliana ubiquitin-associated (UBA)/TS-N domain-containing protein-like protein mRNA, complete cds
AT5G24820	1.893596259	0.072546774	Arabidopsis thaliana aspartyl protease family protein mRNA, complete cds
AT1G28980	1.893596259	0.072546774	Theobroma cacao Uncharacterized protein (TCM_007238) mRNA, complete cds
AT2G07683	1.893596259	0.072546774	Arabidopsis thaliana chromosome 2, complete sequence
AT3G14710	1.893596259	0.072546774	Arabidopsis thaliana RNI-like superfamily protein mRNA, complete cds
AT1G35240	1.893596259	0.072546774	Arabidopsis thaliana auxin response factor 20 mRNA, complete cds
AT1G12070	1.893596259	0.072546774	Arabidopsis thaliana Rho GDP-dissociation inhibitor mRNA, complete cds
AT1G70640	1.893596259	0.072546774	Arabidopsis thaliana octicosapeptide/Phox/Bem1p (PB1) domain-containing protein mRNA, complete cds
AT1G22000	1.893596259	0.072546774	Arabidopsis thaliana putative F-box/FBD/LRR-repeat protein mRNA, complete cds
AT2G07737	1.893596259	0.072546774	Arabidopsis thaliana ecotype Landsberg erecta mitochondrion, complete genome
AT1G02770	1.893596259	0.072546774	Arabidopsis thaliana uncharacterized protein mRNA, complete cds

AT3G59740	1.893596259	0.072546774	Arabidopsis thaliana chromosome 3, complete sequence
AT1G27740	1.893596259	0.072546774	Arabidopsis thaliana transcription factor RSL4 mRNA, complete cds
AT4G19800	1.893596259	0.072546774	Arabidopsis thaliana Glycosyl hydrolase family protein with chitinase insertion domain mRNA, complete cds
AT2G22340	1.893596259	0.072546774	Arabidopsis thaliana chromosome 2, complete sequence
AT2G07722	1.893596259	0.072546774	Arabidopsis thaliana chromosome 2, complete sequence
AT5G47170	2.408169432	0.077500732	Arabidopsis thaliana chromosome 5 sequence
AT3G05770	2.408169432	0.077500732	Arabidopsis thaliana uncharacterized protein mRNA, complete cds
AT1G57650	2.408169432	0.077500732	Arabidopsis thaliana ATP binding protein mRNA, complete cds
AT2G47050	2.408169432	0.077500732	Arabidopsis thaliana chromosome 2, complete sequence
AT1G73220	2.408169432	0.077500732	Arabidopsis thaliana organic cation/carnitine transporter1 mRNA, complete cds
ATCG01070	2.408169432	0.077500732	Arabidopsis thaliana chloroplast DNA, complete genome, ecotype: Columbia
AT1G13370	2.408169432	0.077500732	Arabidopsis thaliana histone H3 mRNA, complete cds
AT5G23155	2.408169432	0.077500732	Arabidopsis thaliana chromosome 5 sequence
AT5G28510	2.408169432	0.077500732	Arabidopsis thaliana beta glucosidase 24 mRNA, complete cds
AT2G07783	2.408169432	0.077500732	Arabidopsis thaliana chromosome 2, complete sequence
AT5G39180	2.408169432	0.077500732	Arabidopsis thaliana germin-like protein subfamily 1 member 19 mRNA, complete cds
AT1G32850	2.408169432	0.077500732	Arabidopsis thaliana putative ubiquitin carboxyl-terminal hydrolase 11 mRNA, complete cds
AT1G31900	2.408169432	0.077500732	Arabidopsis thaliana chromosome 1 sequence
AT2G07687	2.408169432	0.077500732	Arabidopsis thaliana ecotype Landsberg erecta mitochondrion, complete genome
AT2G07728	2.408169432	0.077500732	Arabidopsis thaliana uncharacterized protein mRNA, complete cds
AT5G43120	1.201718554	0.079982136	Arabidopsis thaliana chromosome 5 sequence
AT5G01335	1.201718554	0.079982136	Arabidopsis thaliana chromosome 5 sequence

AT4G23515	1.201718554	0.079982136	Arabidopsis thaliana Toll-Interleukin-1 receptor domain-containing protein mRNA, complete cds
AT4G26055	1.201718554	0.079982136	Arabidopsis thaliana uncharacterized protein mRNA, complete cds
AT1G23540	1.201718554	0.079982136	Arabidopsis thaliana proline-rich receptor-like protein kinase PERK12 mRNA, complete cds

Down regulated genes in Col-0(+Cd)/Col-0(-Cd)

Gene	Log2FoldChange	pval	NT:Description
AT5G14740	-1.236660711	0	Arabidopsis thaliana carbonic anhydrase 2 mRNA, complete cds
AT4G35090	-1.412117018	0	Arabidopsis thaliana catalase 2 mRNA, complete cds
AT1G64720	-1.188857432	0	Arabidopsis thaliana membrane related protein CP5 mRNA, complete cds
AT4G26850	-1.409488376	0	Arabidopsis thaliana GDP-L-galactose phosphorylase 1 mRNA, complete cds
AT1G69530	-2.066639969	0	Arabidopsis thaliana expansin A1 mRNA, complete cds
AT1G74470	-1.042684895	0	Arabidopsis thaliana geranylgeranyl diphosphate reductase mRNA, complete cds
AT4G12550	-2.050183805	0	Arabidopsis thaliana chromosome 4 sequence
AT2G37170	-1.430952536	0	Arabidopsis thaliana aquaporin PIP2-2 mRNA, complete cds
AT3G16240	-1.139177402	0	Arabidopsis thaliana aquaporin TIP2-1 mRNA, complete cds
AT1G01620	-1.124089187	0	Arabidopsis thaliana aquaporin PIP1-3 mRNA, complete cds
AT5G37260	-1.72580741	0	Arabidopsis thaliana MYB family transcription factor Circadian 1 mRNA, complete cds
AT3G24420	-2.555087925	0	Arabidopsis thaliana hydrolase, alpha/beta fold family protein mRNA, complete cds
AT2G28630	-3.488667499	0	Arabidopsis thaliana chromosome 2, complete sequence
AT1G62480	-1.389014907	0	Arabidopsis thaliana vacuolar calcium-binding protein-like protein mRNA, complete cds
AT3G61430	-1.233380418	0	Arabidopsis thaliana aquaporin PIP1-1 mRNA, complete cds
AT5G18670	-1.810103788	0	Arabidopsis thaliana putative beta-amylase BMY3 mRNA, complete cds
AT3G01190	-1.672263628	0	Arabidopsis thaliana peroxidase 27 mRNA, complete cds

AT3G15450	-1.660560748	0	Arabidopsis thaliana aluminum induced protein with YGL and LRDR motif mRNA, complete cds
AT5G47450	-2.636620272	0	Arabidopsis thaliana aquaporin TIP2-3 mRNA, complete cds
AT2G06850	-1.376611299	0	Arabidopsis thaliana endoxyloglucan transferase A1 mRNA, complete cds
AT4G19690	-4.182537052	0	Arabidopsis thaliana Fe(2+) transport protein 1 mRNA, complete cds
AT1G68520	-1.509326264	0	Arabidopsis thaliana zinc finger protein CONSTANS-LIKE 6 mRNA, complete cds
AT4G23400	-1.330020757	0	Arabidopsis thaliana putative aquaporin PIP1-5 mRNA, complete cds
AT4G12520	-2.677895264	0	Arabidopsis thaliana chromosome 4 sequence
AT3G48360	-1.485075623	0	Arabidopsis thaliana TAC1-mediated telomerase activation pathway protein BT2 mRNA, complete cds
AT1G61740	-1.216429141	0	Arabidopsis thaliana Sulfite exporter TauE/SafE family protein mRNA, complete cds
AT4G15390	-1.599406734	0	Arabidopsis thaliana chromosome 4 sequence
AT5G63160	-1.614198381	1.31E-296	Arabidopsis thaliana BTB and TAZ domain protein 1 mRNA, complete cds
AT4G17340	-1.131129516	1.64E-295	Arabidopsis thaliana tonoplast intrinsic protein 2;2 mRNA, complete cds
AT1G01580	-4.057423474	1.64E-280	Arabidopsis thaliana ferric reduction oxidase 2 mRNA, complete cds
AT1G55960	-1.646961638	6.92E-280	Arabidopsis thaliana putative polyketide cyclase/dehydrase and lipid transport-like protein mRNA, complete cds
AT1G64230	-1.022799815	3.22E-278	Arabidopsis thaliana ubiquitin-conjugating enzyme E2 28 mRNA, complete cds
AT5G46890	-3.45041323	7.93E-265	Arabidopsis thaliana chromosome 5 sequence
AT2G29980	-1.067146871	8.08E-261	Arabidopsis thaliana omega-3 fatty acid desaturase mRNA, complete cds
AT5G40850	-1.194954178	3.83E-256	Arabidopsis thaliana urophorphyrin methylase 1 mRNA, complete cds
AT3G52720	-1.625464701	1.09E-249	Arabidopsis thaliana alpha carbonic anhydrase 1 mRNA, complete cds
AT5G16110	-1.123762929	1.38E-243	Arabidopsis thaliana uncharacterized protein mRNA, complete cds
AT1G12080	-1.7725826	6.72E-242	Arabidopsis thaliana vacuolar calcium-binding protein-like protein mRNA, complete cds
AT5G02840	-1.321713367	3.39E-241	Arabidopsis thaliana protein LHY/CCA1-like 1 mRNA, complete cds
AT3G16770	-1.608528722	7.66E-236	Arabidopsis thaliana ethylene-responsive

			transcription factor RAP2-3 mRNA, complete cds
AT5G17820	-1.957222312	2.77E-231	Arabidopsis thaliana peroxidase 57 mRNA, complete cds
AT5G44020	-1.120104948	4.69E-231	Arabidopsis thaliana HAD superfamily, subfamily IIIB acid phosphatase mRNA, complete cds
AT1G15380	-1.330705752	2.13E-230	Arabidopsis thaliana Lactoylglutathione lyase / glyoxalase I family protein mRNA, complete cds
AT1G58290	-1.082255189	9.42E-229	Arabidopsis thaliana glutamyl-tRNA reductase 1 mRNA, complete cds
AT1G08570	-1.491734734	6.91E-224	Arabidopsis thaliana atypical CYS HIS rich thioredoxin 4 mRNA, complete cds
AT5G21020	-1.307656083	2.13E-222	Arabidopsis thaliana chromosome 5 sequence
AT5G17300	-1.4643848	2.98E-222	Arabidopsis thaliana myb family transcription factor RVE1 mRNA, complete cds
AT5G64100	-1.472224642	1.07E-215	Arabidopsis thaliana peroxidase 69 mRNA, complete cds
AT5G46900	-3.411258323	1.71E-210	Arabidopsis thaliana chromosome 5 sequence
AT5G60660	-2.147039401	6.62E-209	Arabidopsis thaliana putative aquaporin PIP2-4 mRNA, complete cds
AT1G12520	-1.290976628	7.13E-207	Arabidopsis thaliana copper chaperone for SOD1 mRNA, complete cds
AT1G68440	-1.467075537	1.90E-197	Arabidopsis thaliana chromosome 1 sequence
AT2G46830	-1.015772485	4.44E-195	Arabidopsis thaliana protein CCA1 mRNA, complete cds
AT2G14910	-1.016613959	3.29E-192	Arabidopsis thaliana uncharacterized protein mRNA, complete cds
AT4G34881	-1.291529155	1.73E-191	Arabidopsis thaliana chromosome 4 sequence
AT1G52190	-1.519385826	7.69E-187	Arabidopsis thaliana nitrate transporter 1.11 mRNA, complete cds
AT1G01120	-1.25206307	8.42E-178	Arabidopsis thaliana chromosome 1 sequence
AT5G24150	-1.188531905	9.30E-178	Arabidopsis thaliana Squalene monooxygenase 5 mRNA, complete cds
AT5G17170	-1.280980371	2.14E-177	Arabidopsis thaliana protein ENHANCER OF SOS3-1 mRNA, complete cds
AT1G80920	-1.180883929	1.22E-164	Arabidopsis thaliana chaperone protein dnaJ 8 mRNA, complete cds
AT3G17510	-1.156277612	8.17E-164	Arabidopsis thaliana CBL-interacting serine/threonine-protein kinase 1 mRNA, complete cds
AT2G39570	-1.304134108	6.92E-155	Arabidopsis thaliana ACT domain-containing protein mRNA, complete cds
AT2G22330	-1.23161033	7.07E-153	Arabidopsis thaliana tryptophan N-monooxygenase 2 mRNA, complete cds
AT4G37610	-1.992327716	7.28E-145	Arabidopsis thaliana BTB and TAZ domain protein

			5 mRNA, complete cds
AT1G64500	-1.424981491	4.44E-141	Arabidopsis thaliana chromosome 1 sequence
AT4G26010	-2.390661418	9.35E-139	Arabidopsis thaliana peroxidase 44 mRNA, complete cds
AT4G21850	-1.135053561	3.02E-137	Arabidopsis thaliana methionine sulfoxide reductase B9 mRNA, complete cds
AT5G57660	-1.103639526	4.86E-137	Arabidopsis thaliana zinc finger protein CONSTANS-LIKE 5 mRNA, complete cds
AT1G30250	-1.90644328	3.80E-133	Arabidopsis thaliana chromosome 1 sequence
AT4G18205	-1.69008669	6.80E-132	Arabidopsis thaliana nucleotide-sugar transporter family protein mRNA, complete cds
AT1G64380	-2.967232076	2.62E-129	Arabidopsis thaliana chromosome 1 sequence
AT5G67400	-2.00449326	1.50E-125	Arabidopsis thaliana peroxidase 73 mRNA, complete cds
AT3G52060	-1.026580613	5.01E-124	Arabidopsis thaliana chromosome 3, complete sequence
AT5G41900	-2.867004894	3.47E-119	Arabidopsis thaliana hydrolase, alpha/beta fold family protein mRNA, complete cds
AT2G26690	-1.045670339	2.42E-117	Arabidopsis thaliana nitrate transporter 1.4 mRNA, complete cds
AT5G49360	-1.214884179	4.56E-117	Arabidopsis thaliana bifunctional {beta}-D-xylosidase/{alpha}-L-arabinofuranosidase mRNA, complete cds
AT4G00970	-1.650724257	1.09E-116	Arabidopsis thaliana cysteine-rich receptor-like protein kinase 41 mRNA, complete cds
AT3G13790	-1.141833231	2.71E-115	Arabidopsis thaliana beta-fructofuranosidase, insoluble isoenzyme CWINV1 mRNA, complete cds
AT1G64860	-1.03851692	3.72E-112	Arabidopsis thaliana RNA polymerase sigma subunit 1 mRNA, complete cds
AT4G30170	-1.141240652	1.67E-111	Arabidopsis thaliana peroxidase 45 mRNA, complete cds
AT1G32900	-1.040222664	1.17E-109	Arabidopsis thaliana granule-bound starch synthase 1 mRNA, complete cds
AT3G21670	-1.211272859	4.64E-108	Arabidopsis thaliana nitrate transporter 1.3 mRNA, complete cds
AT3G12900	-4.895611316	1.23E-107	Arabidopsis thaliana oxidoreductase, 2OG-Fe(II) oxygenase family protein mRNA, complete cds
AT5G58770	-1.421415438	2.10E-104	Arabidopsis thaliana dehydrodolichyl diphosphate synthase 2 mRNA, complete cds
AT5G53160	-1.043351571	9.29E-103	Arabidopsis thaliana regulatory component of ABA receptor 3 mRNA, complete cds
AT3G09600	-1.245602227	9.34E-103	Arabidopsis thaliana protein REVEILLE 8 mRNA, complete cds

AT4G33120	-2.580714536	5.16E-102	Arabidopsis thaliana S-adenosyl-L-methionine-dependent methyltransferases superfamily protein mRNA, complete cds
AT5G44610	-1.712750112	5.70E-101	Arabidopsis thaliana microtubule-associated protein 18 mRNA, complete cds
AT4G22570	-1.106671543	9.07E-100	Arabidopsis thaliana adenine phosphoribosyl transferase 3 mRNA, complete cds
AT2G21650	-3.137839435	5.79E-98	Arabidopsis thaliana MYB transcription factor RSM1 mRNA, complete cds
AT4G16780	-1.856316771	5.41E-97	Arabidopsis thaliana homeobox protein 2 mRNA, complete cds
AT4G15780	-1.170847305	2.80E-96	Arabidopsis thaliana vesicle-associated membrane protein 724 mRNA, complete cds
AT2G15020	-1.366413872	5.69E-96	Arabidopsis thaliana chromosome 2, complete sequence
AT2G40000	-1.042250946	6.17E-94	Arabidopsis thaliana chromosome 2, complete sequence
AT5G21940	-1.177709357	2.04E-90	Arabidopsis thaliana uncharacterized protein mRNA, complete cds
AT5G38940	-1.025770067	3.89E-90	Arabidopsis thaliana germin-like protein subfamily 1 member 11 mRNA, complete cds
AT5G45340	-1.628162103	2.40E-89	Arabidopsis thaliana abscisic acid 8'-hydroxylase 3 mRNA, complete cds
AT4G17730	-1.363253158	3.62E-89	Arabidopsis thaliana syntaxin-23 mRNA, complete cds
AT1G01190	-1.862604575	6.24E-89	Arabidopsis thaliana cytochrome P450, family 78, subfamily A, polypeptide 8 mRNA, complete cds
AT3G25930	-1.798506568	9.63E-89	Arabidopsis thaliana Adenine nucleotide alpha hydrolases-like superfamily protein mRNA, complete cds
AT3G06070	-1.496429352	4.50E-87	Arabidopsis thaliana uncharacterized protein mRNA, complete cds
AT3G44300	-2.077330169	1.70E-86	Arabidopsis thaliana nitrilase 2 mRNA, complete cds
AT5G43360	-5.425511317	3.95E-85	Arabidopsis thaliana putative inorganic phosphate transporter 1-3 mRNA, complete cds
AT3G29410	-1.460635387	6.57E-85	Arabidopsis thaliana terpenoid synthase 25 mRNA, complete cds
AT1G65490	-2.098010446	6.73E-84	Arabidopsis thaliana uncharacterized protein mRNA, complete cds
AT3G28270	-1.208107518	9.35E-83	Arabidopsis thaliana chromosome 3, complete sequence
AT5G26130	-2.588358376	1.66E-82	Arabidopsis thaliana chromosome 5 sequence

AT5G03555	-1.182247499	8.08E-82	Arabidopsis thaliana chromosome 5 sequence
AT4G08300	-1.583000171	6.13E-81	Arabidopsis thaliana nodulin MtN21 /EamA-like transporter family protein mRNA, complete cds
AT1G74670	-1.174410418	6.96E-81	Arabidopsis thaliana gibberellin-regulated protein 6 mRNA, complete cds
AT4G30470	-1.684016786	1.47E-80	Arabidopsis thaliana cinnamoyl-CoA reductase like protein mRNA, complete cds
AT1G70880	-2.467435475	7.31E-80	Arabidopsis thaliana SRPBCC domain-containing protein mRNA, complete cds
AT1G04040	-1.1019496	1.09E-79	Arabidopsis thaliana HAD superfamily, subfamily IIIB acid phosphatase mRNA, complete cds
AT3G60300	-1.021212166	1.46E-78	Arabidopsis thaliana RWD domain-containing protein mRNA, complete cds
AT4G31910	-1.57087095	1.93E-73	Arabidopsis thaliana BR-related acyltransferase 1 mRNA, complete cds
AT3G23030	-1.032052395	5.14E-73	Arabidopsis thaliana auxin-responsive protein IAA2 mRNA, complete cds
AT2G25680	-1.984965614	6.70E-73	Arabidopsis thaliana chromosome 2, complete sequence
AT3G15630	-1.603517354	1.84E-72	Arabidopsis thaliana chromosome 3, complete sequence
AT2G24550	-1.130717198	1.15E-71	Arabidopsis thaliana uncharacterized protein mRNA, complete cds
AT2G24600	-1.35156747	4.02E-71	Arabidopsis thaliana Ankyrin repeat family protein mRNA, complete cds
AT2G31380	-1.066316533	4.30E-71	Arabidopsis thaliana B-box zinc finger protein STH mRNA, complete cds
AT3G25190	-2.682321102	1.09E-70	Arabidopsis thaliana vacuolar iron transporter homolog 2.1 mRNA, complete cds
AT3G26510	-1.134908988	3.15E-70	Arabidopsis thaliana PB1_UP2 domain-containing protein mRNA, complete cds
AT5G53250	-2.216157593	8.12E-70	Arabidopsis thaliana mRNA for predicted GPI-anchored protein, complete cds, clone: RAFL16-23-F12
AT3G25585	-1.009238972	1.64E-68	Arabidopsis thaliana aminoalcoholphosphotransferase mRNA, complete cds
AT1G63570	-3.266547722	6.36E-68	Arabidopsis thaliana chromosome 1 sequence
AT4G02330	-1.537863419	9.96E-68	Arabidopsis thaliana Probable pectinesterase/pectinesterase inhibitor 41 mRNA, complete cds
AT1G78860	-1.264198296	1.83E-67	Arabidopsis thaliana chromosome 1 sequence
AT5G56870	-1.349705152	2.46E-66	Arabidopsis thaliana beta-galactosidase 4 mRNA, complete cds

AT2G32800	-1.358470445	4.23E-66	Arabidopsis thaliana chromosome 2, complete sequence
AT5G62470	-1.430373868	8.10E-66	Arabidopsis thaliana Myb transcription factor mRNA, complete cds
AT1G80440	-1.653677575	1.05E-65	Arabidopsis thaliana chromosome 1 sequence
AT1G16400	-1.225546728	1.53E-65	Arabidopsis thaliana Hexahomomethionine N-hydroxylase mRNA, complete cds
AT3G56290	-1.149848743	1.69E-65	Arabidopsis thaliana mRNA for hypothetical protein, complete cds, clone: RAFL14-54-O14
AT4G14630	-1.15894849	5.57E-65	Arabidopsis thaliana germin-like protein 9 mRNA, complete cds
AT5G28770	-1.110819795	1.11E-63	Arabidopsis thaliana basic leucine zipper 63 mRNA, complete cds
AT5G12940	-1.965710743	1.19E-63	Arabidopsis thaliana chromosome 5 sequence
AT2G34620	-1.076502983	8.93E-61	Arabidopsis thaliana mitochondrial transcription termination factor family protein mRNA, complete cds
AT4G33420	-1.206719566	1.09E-60	Arabidopsis thaliana probable peroxidase mRNA, complete cds
AT1G05260	-1.051843255	4.92E-60	Arabidopsis thaliana peroxidase 3 mRNA, complete cds
AT5G19120	-1.253485826	8.74E-59	Arabidopsis thaliana chromosome 5 sequence
AT1G21100	-1.30679906	6.50E-57	Arabidopsis thaliana indole glucosinolate o-methyltransferase 1 mRNA, complete cds
AT5G19190	-1.044954646	1.64E-56	Arabidopsis thaliana uncharacterized protein mRNA, complete cds
AT5G04730	-1.601814657	4.15E-56	Arabidopsis thaliana Ankyrin-repeat containing protein mRNA, complete cds
AT5G52900	-1.821246524	5.26E-56	Arabidopsis thaliana membrane-associated kinase regulator family protein mRNA, complete cds
AT4G14440	-1.31423604	6.82E-55	Arabidopsis thaliana chromosome 4 sequence
AT3G48720	-1.143915218	1.24E-54	Arabidopsis thaliana HXXXD-type acyl-transferase family protein mRNA, complete cds
AT4G12510	-2.905145533	3.48E-54	Arabidopsis thaliana chromosome 4 sequence
AT4G07820	-2.103394991	8.50E-54	Arabidopsis thaliana chromosome 4 sequence
AT5G49100	-1.237812015	1.64E-53	Arabidopsis thaliana chromosome 5 sequence
AT4G02270	-1.799047004	5.54E-53	Arabidopsis thaliana protein root hair specific 13 mRNA, complete cds
AT3G32030	-1.990484454	1.51E-52	Arabidopsis thaliana terpenoid synthase 30 mRNA, complete cds
AT4G01630	-1.913758663	1.80E-52	Arabidopsis thaliana expansin A17 mRNA, complete cds
AT2G32540	-1.202189846	2.40E-52	Arabidopsis thaliana cellulose synthase-like protein B4 mRNA, complete cds

AT5G61600	-1.324390983	5.84E-51	Arabidopsis thaliana chromosome 5 sequence
AT1G22890	-1.460803211	6.25E-51	Arabidopsis thaliana uncharacterized protein mRNA, complete cds
AT3G57040	-1.102291204	2.40E-50	Arabidopsis thaliana two-component response regulator ARR9 mRNA, complete cds
AT5G65980	-1.742713122	4.24E-50	Arabidopsis thaliana auxin efflux carrier family protein mRNA, complete cds
AT5G48412	-1.120610234	8.57E-50	Arabidopsis thaliana chromosome 5 sequence
AT5G56860	-1.038870583	9.52E-50	Arabidopsis thaliana GATA transcription factor 21 mRNA, complete cds
AT3G47420	-1.086823372	3.31E-49	Arabidopsis thaliana putative glycerol-3-phosphate transporter 1 mRNA, complete cds
AT5G57625	-2.034613058	2.12E-48	Arabidopsis thaliana CAP (Cysteine-rich secretory proteins, Antigen 5, and Pathogenesis-related 1 protein) superfamily protein mRNA, complete cds
AT1G07180	-1.077663846	5.46E-48	Arabidopsis thaliana internal alternative NAD(P)H-ubiquinone oxidoreductase A1 mRNA, complete cds
AT1G15350	-1.420200796	9.15E-48	Arabidopsis thaliana uncharacterized protein mRNA, complete cds
AT1G31350	-1.313591291	2.46E-47	Arabidopsis thaliana chromosome 1 sequence
AT3G59370	-2.130284077	6.46E-47	Arabidopsis thaliana vacuolar calcium-binding protein-like protein mRNA, complete cds
AT1G49130	-2.028125688	1.19E-46	Arabidopsis thaliana zinc finger protein CONSTANS-LIKE 8 mRNA, complete cds
AT5G12050	-1.576330196	1.63E-46	Arabidopsis thaliana chromosome 5 sequence
AT5G15180	-2.119662962	2.65E-46	Arabidopsis thaliana peroxidase 56 mRNA, complete cds
AT4G38932	-1.205031774	5.76E-46	Arabidopsis thaliana chromosome 4 sequence
AT2G36100	-1.444975592	6.94E-46	Arabidopsis thaliana casparian strip membrane protein 1 mRNA, complete cds
AT3G15310	-2.235686758	1.00E-45	Arabidopsis thaliana chromosome 3, complete sequence
AT4G27030	-1.173424074	1.11E-45	Arabidopsis thaliana chromosome 4 sequence
AT1G25230	-1.072425487	1.45E-45	Arabidopsis thaliana Calcineurin-like metallo-phosphoesterase superfamily protein mRNA, complete cds
AT1G30720	-1.784393373	1.64E-45	Arabidopsis thaliana chromosome 1 sequence
AT2G22122	-1.34480848	2.25E-45	Arabidopsis thaliana chromosome 2, complete sequence
AT5G37770	-1.159039376	2.26E-45	Arabidopsis thaliana chromosome 5 sequence
AT4G15290	-4.049895351	4.78E-45	Arabidopsis thaliana cellulose synthase-like protein B5 mRNA, complete cds

AT3G27350	-1.200379889	9.96E-45	Arabidopsis thaliana uncharacterized protein mRNA, complete cds
AT3G54366	-1.201432355	4.14E-44	Arabidopsis thaliana chromosome 3, complete sequence
AT1G33610	-1.510517591	5.20E-44	Arabidopsis thaliana leucine-rich repeat (LRR) family protein mRNA, complete cds
AT1G29025	-1.173290221	5.44E-44	Arabidopsis thaliana Calcium-binding EF-hand family protein mRNA, complete cds
AT1G49200	-1.759986477	1.17E-43	Arabidopsis thaliana chromosome 1 sequence
AT3G51910	-1.628831651	1.29E-43	Arabidopsis thaliana heat stress transcription factor A-7a mRNA, complete cds
AT4G19030	-1.731309352	2.23E-43	Arabidopsis thaliana aquaporin NIP1-1 mRNA, complete cds
AT5G22920	-1.150349401	2.59E-43	Arabidopsis thaliana ring finger and CHY zinc finger domain-containing protein 1 mRNA, complete cds
AT5G43520	-1.322294597	2.68E-43	Arabidopsis thaliana chromosome 5 sequence
AT1G21140	-3.038771243	3.03E-43	Arabidopsis thaliana chromosome 1 sequence
AT5G47540	-1.184432545	5.08E-43	Arabidopsis thaliana putative MO25-like protein mRNA, complete cds
AT1G65310	-2.203714368	7.04E-43	Arabidopsis thaliana probable xyloglucan endotransglucosylase/hydrolase protein 17 mRNA, complete cds
AT5G66052	-1.305588771	1.33E-42	Arabidopsis thaliana uncharacterized protein mRNA, complete cds
AT3G55500	-1.856171172	1.68E-42	Arabidopsis thaliana expansin A16 mRNA, complete cds
AT1G51470	-1.470842131	1.93E-42	Arabidopsis thaliana myrosinase 5 mRNA, complete cds
AT5G57685	-1.200570452	4.99E-42	Arabidopsis thaliana chromosome 5 sequence
AT4G15430	-1.142577354	5.16E-42	Arabidopsis thaliana ERD (early-responsive to dehydration stress) family protein mRNA, complete cds
AT5G11070	-1.205404191	6.06E-42	Arabidopsis thaliana chromosome 5 sequence
AT3G47430	-1.068508671	1.30E-41	Arabidopsis thaliana peroxisomal membrane protein 11B mRNA, complete cds
AT1G05650	-3.065761757	1.92E-41	Arabidopsis thaliana pectin lyase-like protein mRNA, complete cds
AT1G30870	-1.848467203	2.35E-41	Arabidopsis thaliana peroxidase 7 mRNA, complete cds
AT2G33250	-1.117772555	4.76E-41	Arabidopsis thaliana uncharacterized protein mRNA, complete cds
AT1G47600	-1.355560388	5.38E-41	Arabidopsis thaliana myrosinase 4 mRNA, complete cds

AT5G15830	-1.781815793	6.83E-41	Arabidopsis thaliana chromosome 5 sequence
AT1G06830	-1.491265359	7.50E-41	Arabidopsis thaliana chromosome 1 sequence
AT1G52070	-1.469721292	8.29E-41	Arabidopsis thaliana jacalin-like lectin domain-containing protein mRNA, complete cds
AT5G22390	-1.134514448	1.02E-40	Arabidopsis thaliana chromosome 5 sequence
AT5G48540	-1.077257395	1.78E-40	Arabidopsis thaliana chromosome 5 sequence
AT2G45220	-1.083299202	2.96E-40	Arabidopsis thaliana putative pectinesterase/pectinesterase inhibitor 17 mRNA, complete cds
AT4G37070	-1.381151568	5.03E-40	Arabidopsis thaliana patatin-related phospholipase A mRNA, complete cds
AT2G21320	-1.327352746	1.82E-39	Arabidopsis thaliana B-BOX domain protein 18 mRNA, complete cds
AT5G07080	-1.257866993	2.60E-39	Arabidopsis thaliana HXXXD-type acyl-transferase family protein mRNA, complete cds
AT1G09250	-1.201609389	2.86E-39	Arabidopsis thaliana chromosome 1 sequence
AT1G72645	-1.102922612	3.09E-39	Arabidopsis thaliana chromosome 1 sequence
AT2G33790	-1.093034687	8.10E-39	Arabidopsis thaliana arabinogalactan protein 30 mRNA, complete cds
AT4G30670	-1.783698123	9.14E-39	Arabidopsis thaliana chromosome 4 sequence
AT3G15770	-1.549946487	1.24E-38	Arabidopsis thaliana uncharacterized protein mRNA, complete cds
AT3G54200	-1.186303288	6.75E-38	Arabidopsis thaliana chromosome 3, complete sequence
AT4G26400	-1.130764538	4.94E-37	Arabidopsis thaliana RING/U-box domain-containing protein mRNA, complete cds
AT2G02450	-1.381325214	1.54E-36	Arabidopsis thaliana protein LONG VEGETATIVE PHASE 1 mRNA, complete cds
AT1G53830	-1.634604592	4.19E-36	Arabidopsis thaliana pectin methylesterase 2 mRNA, complete cds
AT5G42825	-1.282804045	5.11E-36	Arabidopsis thaliana chromosome 5 sequence
AT5G51190	-2.211439212	7.03E-36	Arabidopsis thaliana chromosome 5 sequence
AT1G75180	-1.028173513	7.39E-36	Arabidopsis thaliana Erythronate-4-phosphate dehydrogenase family protein mRNA, complete cds
AT2G44300	-1.834324196	2.10E-35	Arabidopsis thaliana bifunctional inhibitor/lipid-transfer protein/seed storage 2S albumin superfamily protein mRNA, complete cds
AT4G22212	-1.237871005	2.50E-35	Arabidopsis thaliana defensin-like protein 98 mRNA, complete cds
AT1G68190	-1.035555667	3.42E-35	Arabidopsis thaliana putative zinc finger protein mRNA, complete cds
AT2G24710	-1.799754034	4.55E-35	Arabidopsis thaliana glutamate receptor 2.3 mRNA, complete cds

AT5G27920	-1.382434126	9.02E-35	Arabidopsis thaliana F-box protein mRNA, complete cds
AT5G24410	-1.556436662	9.31E-35	Arabidopsis thaliana 6-phosphogluconolactonase 4 mRNA, complete cds
AT3G11550	-2.215294969	2.50E-34	Arabidopsis thaliana uncharacterized protein mRNA, complete cds
AT5G19970	-1.834031471	1.13E-33	Arabidopsis thaliana chromosome 5 sequence
AT4G31380	-6.02228312	2.37E-33	Arabidopsis thaliana chromosome 4 sequence
AT4G38060	-1.128124944	3.60E-33	Arabidopsis thaliana chromosome 4 sequence
AT4G32280	-2.434330577	5.08E-33	Arabidopsis thaliana auxin-responsive protein IAA29 mRNA, complete cds
AT1G69252	-1.01038421	7.76E-33	Arabidopsis thaliana chromosome 1 sequence
AT2G27402	-1.281329424	9.81E-33	Arabidopsis thaliana chromosome 2, complete sequence
AT4G14130	-1.112993364	1.15E-32	Arabidopsis thaliana probable xyloglucan endotransglucosylase/hydrolase protein 15 mRNA, complete cds
AT4G40090	-1.518349971	1.55E-32	Arabidopsis thaliana chromosome 4 sequence
AT5G61340	-1.094594439	3.24E-32	Arabidopsis thaliana chromosome 5 sequence
AT5G35777	-2.449811563	4.52E-32	Arabidopsis thaliana chromosome 5 sequence
AT4G30290	-1.512110541	1.37E-31	Arabidopsis thaliana xyloglucan endotransglucosylase/hydrolase 19 mRNA, complete cds
AT4G25820	-1.421362281	2.09E-31	Arabidopsis thaliana xyloglucan endotransglucosylase/hydrolase protein 14 mRNA, complete cds
AT3G49790	-1.454904932	2.71E-31	Arabidopsis thaliana chromosome 3, complete sequence
AT3G02850	-1.105212907	4.40E-31	Arabidopsis thaliana potassium channel SKOR mRNA, complete cds
AT2G40300	-1.145756772	4.40E-31	Arabidopsis thaliana ferritin 4 mRNA, complete cds
AT3G29250	-1.183847827	7.43E-31	Arabidopsis thaliana short-chain dehydrogenase reductase 4 mRNA, complete cds
AT2G40670	-1.409083951	9.01E-31	Arabidopsis thaliana two-component response regulator ARR16 mRNA, complete cds
AT4G22666	-1.590351639	1.11E-30	Arabidopsis thaliana Bifunctional inhibitor/lipid-transfer protein/seed storage 2S albumin superfamily protein mRNA, complete cds
AT1G52050	-1.599325105	1.28E-30	Arabidopsis thaliana jacalin-like lectin domain-containing protein mRNA, complete cds
AT5G54370	-1.053616327	1.30E-30	Arabidopsis thaliana late embryogenesis abundant protein-like protein mRNA, complete cds

AT1G26800	-1.561698597	1.67E-30	Arabidopsis thaliana chromosome 1 sequence
AT3G16440	-1.596369728	3.05E-30	Arabidopsis thaliana myrosinase-binding protein-like protein-300B mRNA, complete cds
AT2G39430	-1.833339981	3.33E-30	Arabidopsis thaliana chromosome 2, complete sequence
AT4G25250	-2.017046472	4.72E-30	Arabidopsis thaliana chromosome 4 sequence
AT2G28160	-1.164957933	9.53E-30	Arabidopsis thaliana FER-LIKE IRON DEFICIENCY-INDUCED transcription factor mRNA, complete cds
AT4G29740	-1.362598871	9.72E-30	Arabidopsis thaliana cytokinin dehydrogenase 4 mRNA, complete cds
AT4G11210	-2.24791306	1.03E-29	Arabidopsis thaliana chromosome 4 sequence
AT1G72820	-1.102615481	2.81E-29	Arabidopsis thaliana chromosome 1 sequence
AT1G49010	-1.143240509	3.87E-29	Arabidopsis thaliana myb/SANT-like DNA-binding domain-containing protein mRNA, complete cds
AT4G17280	-1.813594501	4.56E-29	Arabidopsis thaliana putative auxin-responsive protein mRNA, complete cds
AT1G52060	-1.129187137	5.97E-29	Arabidopsis thaliana jacalin-like lectin domain-containing protein mRNA, complete cds
AT5G04770	-1.697550113	6.04E-29	Arabidopsis thaliana cationic amino acid transporter 6 mRNA, complete cds
AT4G08380	-1.328796162	1.66E-28	Arabidopsis thaliana chromosome 4 sequence
AT5G56550	-1.450512071	1.98E-28	Arabidopsis thaliana protein OXIDATIVE STRESS 3 mRNA, complete cds
AT4G21250	-1.341396199	3.31E-28	Arabidopsis thaliana Sulfite exporter TauE/SafE family protein mRNA, complete cds
AT4G12390	-1.181429479	3.31E-28	Arabidopsis thaliana chromosome 4 sequence
AT1G13420	-1.806149689	4.12E-28	Arabidopsis thaliana chromosome 1 sequence
AT3G12320	-1.019111663	4.17E-28	Arabidopsis thaliana uncharacterized protein mRNA, complete cds
AT2G45430	-1.264833104	5.26E-28	Arabidopsis thaliana chromosome 2, complete sequence
AT4G08290	-1.039481585	6.10E-28	Arabidopsis thaliana nodulin MtN21 /EamA-like transporter family protein mRNA, complete cds
AT3G48115	-1.147785785	6.50E-28	Arabidopsis thaliana chromosome 3, complete sequence
AT3G02700	-1.004732323	7.40E-28	Arabidopsis thaliana NC domain-containing protein-related mRNA, complete cds
AT5G42180	-1.472990889	1.60E-27	Arabidopsis thaliana peroxidase mRNA, complete cds
AT1G53680	-1.924646979	1.77E-27	Arabidopsis thaliana glutathione S-transferase TAU 28 mRNA, complete cds
AT3G08040	-2.546026879	3.93E-27	Arabidopsis thaliana protein FERRIC REDUCTASE DEFECTIVE 3 mRNA, complete cds

AT4G16690	-1.19465949	1.05E-26	Arabidopsis thaliana methyl esterase 16 mRNA, complete cds
AT5G56320	-1.45776449	1.15E-26	Arabidopsis thaliana expansin A14 mRNA, complete cds
AT4G30140	-1.173554695	1.16E-26	Arabidopsis thaliana GDSL esterase/lipase mRNA, complete cds
AT5G05860	-1.082495772	1.29E-26	Arabidopsis thaliana cytokinin-N-glucosyltransferase 2 mRNA, complete cds
AT4G20240	-1.692731784	3.13E-26	Arabidopsis thaliana cytochrome P450, family 71, subfamily A, polypeptide 27 mRNA, complete cds
AT5G14330	-1.156974782	3.82E-26	Arabidopsis thaliana uncharacterized protein mRNA, complete cds
AT5G03570	-2.074223335	4.59E-26	Arabidopsis thaliana nickel transport protein FPN2 mRNA, complete cds
AT2G44220	-5.641679118	4.89E-26	Arabidopsis thaliana uncharacterized protein mRNA, complete cds
AT2G18980	-1.360897006	8.64E-26	Arabidopsis thaliana peroxidase 16 mRNA, complete cds
AT1G07570	-1.129057302	1.82E-25	Arabidopsis thaliana protein kinase APK1A mRNA, complete cds
AT4G23870	-1.60846657	2.07E-25	Arabidopsis thaliana chromosome 4 sequence
AT3G58810	-1.644368762	3.08E-25	Arabidopsis thaliana chromosome 3, complete sequence
AT5G41080	-1.061315852	1.38E-24	Arabidopsis thaliana glycerophosphoryl diester phosphodiesterase family protein mRNA, complete cds
AT1G33800	-1.084343182	3.30E-24	Arabidopsis thaliana glucuronoxylan 4-O-methyltransferase mRNA, complete cds
AT1G73870	-1.088410496	7.58E-24	Arabidopsis thaliana zinc finger protein CONSTANS-LIKE 7 mRNA, complete cds
AT1G63560	-2.656983248	9.31E-24	Arabidopsis thaliana chromosome 1 sequence
AT1G17190	-1.485545775	1.13E-23	Arabidopsis thaliana glutathione S-transferase tau 26 mRNA, complete cds
AT1G05660	-1.967197922	1.23E-23	Arabidopsis thaliana pectin lyase-like protein mRNA, complete cds
AT3G21150	-1.67929341	1.54E-23	Arabidopsis thaliana chromosome 3, complete sequence
AT1G32520	-1.242459987	1.56E-23	Arabidopsis thaliana uncharacterized protein mRNA, complete cds
AT5G38930	-1.27330105	2.23E-23	Arabidopsis thaliana germin-like protein subfamily 1 member 10 mRNA, complete cds
AT2G32300	-1.68363888	3.92E-23	Arabidopsis thaliana uclacyanin 1 mRNA, complete cds

AT2G28200	-1.512796349	5.00E-23	Arabidopsis thaliana chromosome 2, complete sequence
AT1G78460	-1.139178777	5.98E-23	Arabidopsis thaliana SOUL heme-binding protein mRNA, complete cds
AT1G53940	-1.485046227	7.47E-23	Arabidopsis thaliana GDSL-motif lipase 2 mRNA, complete cds
AT1G73300	-1.913758663	7.58E-23	Arabidopsis thaliana serine carboxypeptidase-like 2 mRNA, complete cds
AT5G57220	-1.212282154	9.44E-23	Arabidopsis thaliana cytochrome P450, family 81, subfamily F, polypeptide 2 mRNA, complete cds
AT1G21050	-1.107238393	1.01E-22	Arabidopsis thaliana chromosome 1 sequence
AT5G63190	-1.306076086	1.37E-22	Arabidopsis thaliana MA3 domain-containing protein mRNA, complete cds
AT3G50560	-1.682639437	1.50E-22	Arabidopsis thaliana Rossmann-fold NAD(P)-binding domain-containing protein mRNA, complete cds
AT4G13620	-1.322294597	1.75E-22	Arabidopsis thaliana chromosome 4 sequence
AT4G26050	-1.341039758	3.93E-22	Arabidopsis thaliana plant intracellular ras group-related LRR 8 mRNA, complete cds
AT5G40730	-1.809569813	6.38E-22	Arabidopsis thaliana chromosome 5 sequence
AT2G43890	-2.614198381	8.14E-22	Arabidopsis thaliana putative polygalacturonase /pectinase mRNA, complete cds
AT3G18773	-1.26823733	8.22E-22	Arabidopsis thaliana chromosome 3, complete sequence
AT2G23620	-1.626786203	1.45E-21	Arabidopsis thaliana methyl esterase 1 mRNA, complete cds
AT2G30210	-1.121429579	1.86E-21	Arabidopsis thaliana laccase 3 mRNA, complete cds
AT1G35612	-1.007941102	2.50E-21	Arabidopsis thaliana chromosome 1 sequence
AT3G61060	-1.071760497	3.80E-21	Arabidopsis thaliana phloem protein 2-A13 mRNA, complete cds
AT2G27370	-1.999334395	3.82E-21	Arabidopsis thaliana uncharacterized protein mRNA, complete cds
AT5G01600	-1.433765722	4.62E-21	Arabidopsis thaliana ferretin 1 mRNA, complete cds
AT3G43960	-1.536057315	4.95E-21	Arabidopsis thaliana putative cysteine proteinase mRNA, complete cds
AT2G28110	-1.217308229	5.33E-21	Arabidopsis thaliana probable glucuronoxylan glucuronosyltransferase IRX7 mRNA, complete cds
AT3G23800	-1.274053198	6.11E-21	Arabidopsis thaliana selenium-binding protein 3 mRNA, complete cds
AT5G25130	-1.044864416	7.39E-21	Arabidopsis thaliana cytochrome P450 71B12 mRNA, complete cds
AT5G18030	-2.075646346	7.95E-21	Arabidopsis thaliana chromosome 5 sequence

AT3G48100	-1.062100514	8.05E-21	Arabidopsis thaliana two-component response regulator ARR5 mRNA, complete cds
AT1G63180	-1.578574472	8.66E-21	Arabidopsis thaliana UDP-glucose 4-epimerase mRNA, complete cds
AT3G47710	-3.123212029	2.25E-20	Arabidopsis thaliana atypical non-DNA binding bHLH protein BNQ3 mRNA, complete cds
AT5G04960	-1.504054608	3.12E-20	Arabidopsis thaliana putative pectinesterase/pectinesterase inhibitor 46 mRNA, complete cds
AT1G30730	-1.354985686	4.15E-20	Arabidopsis thaliana chromosome 1 sequence
AT2G25980	-1.037903804	8.43E-20	Arabidopsis thaliana myrosinase-binding protein-like protein mRNA, complete cds
AT2G38090	-1.137341918	1.24E-19	Arabidopsis thaliana Duplicated homeodomain-like superfamily protein mRNA, complete cds
AT1G06090	-1.221689607	1.47E-19	Arabidopsis thaliana delta-9 desaturase-like 1 protein mRNA, complete cds
AT3G23510	-1.106403741	2.31E-19	Arabidopsis thaliana cyclopropane-fatty-acyl-phospholipid synthase mRNA, complete cds
AT1G06120	-3.250042051	2.66E-19	Arabidopsis thaliana delta-9 desaturase-like 3 protein mRNA, complete cds
AT5G49640	-1.113982265	2.95E-19	Arabidopsis thaliana chromosome 5 sequence
AT2G01300	-2.839758082	3.48E-19	Arabidopsis thaliana chromosome 2, complete sequence
AT3G50060	-1.11148903	4.60E-19	Arabidopsis thaliana chromosome 3, complete sequence
AT3G18450	-1.383243946	4.62E-19	Arabidopsis thaliana PLAC8 family protein mRNA, complete cds
AT5G66580	-1.625413169	5.36E-19	Arabidopsis thaliana chromosome 5 sequence
AT4G12000	-1.411877904	9.77E-19	Arabidopsis thaliana SNARE associated Golgi family protein mRNA, complete cds
AT1G68110	-1.947705995	1.33E-18	Arabidopsis thaliana chromosome 1 sequence
AT4G24972	-1.14641942	1.34E-18	Arabidopsis thaliana protein TAPETUM DETERMINANT 1 mRNA, complete cds
AT3G02620	-1.406602962	1.41E-18	Arabidopsis thaliana acyl-[acyl-carrier-protein] desaturase mRNA, complete cds
AT4G31320	-1.710595713	1.44E-18	Arabidopsis thaliana chromosome 4 sequence
AT4G33790	-1.816796933	1.98E-18	Arabidopsis thaliana fatty acyl-CoA reductase CER4 mRNA, complete cds
AT3G59880	-1.859015699	2.09E-18	Arabidopsis thaliana chromosome 3, complete sequence
AT4G01250	-1.126062267	2.73E-18	Arabidopsis thaliana WRKY transcription factor 22 mRNA, complete cds

AT1G12560	-1.536195869	3.41E-18	Arabidopsis thaliana alpha-expansin family protein mRNA, complete cds
AT3G19850	-1.009643098	3.51E-18	Arabidopsis thaliana phototropic-responsive NPH3 family protein mRNA, complete cds
AT3G20340	-3.176793069	4.36E-18	Arabidopsis thaliana chromosome 3, complete sequence
AT4G26320	-1.602556975	6.87E-18	Arabidopsis thaliana chromosome 4 sequence
AT3G24020	-1.323778088	7.25E-18	Arabidopsis thaliana chromosome 3, complete sequence
AT5G33355	-1.136151085	8.10E-18	Arabidopsis thaliana defensin-like protein mRNA, complete cds
AT2G31085	-2.378881394	1.00E-17	Arabidopsis thaliana chromosome 2, complete sequence
AT1G66230	-1.30421814	1.02E-17	Arabidopsis thaliana myb domain protein 20 mRNA, complete cds
AT3G54770	-1.425870598	1.57E-17	Arabidopsis thaliana putative RNA binding protein mRNA, complete cds
AT5G65207	-2.010620202	1.81E-17	Arabidopsis thaliana chromosome 5 sequence
AT1G71692	-1.111466821	2.21E-17	Arabidopsis thaliana agamous-like MADS-box protein AGL12 mRNA, complete cds
AT5G17350	-1.683832569	4.66E-17	Arabidopsis thaliana chromosome 5 sequence
AT3G52450	-1.099625208	5.54E-17	Arabidopsis thaliana chromosome 3, complete sequence
AT3G55646	-1.433536995	7.97E-17	Arabidopsis thaliana uncharacterized protein mRNA, complete cds
AT3G21550	-1.077878043	8.09E-17	Arabidopsis thaliana chromosome 3, complete sequence
AT5G19040	-1.210069224	1.02E-16	Arabidopsis thaliana chromosome 5 sequence
AT3G29110	-1.341751818	1.07E-16	Arabidopsis thaliana putative terpenoid synthase 16 mRNA, complete cds
AT5G17290	-1.031672866	1.21E-16	Arabidopsis thaliana autophagy protein 5 mRNA, complete cds
AT4G01720	-1.042342869	1.37E-16	Arabidopsis thaliana putative WRKY transcription factor 47 mRNA, complete cds
AT1G53170	-1.032298278	1.40E-16	Arabidopsis thaliana chromosome 1 sequence
AT5G06200	-1.614198381	1.87E-16	Arabidopsis thaliana uncharacterized protein mRNA, complete cds
AT5G42590	-1.485665011	1.92E-16	Arabidopsis thaliana cytochrome P450 71A16 mRNA, complete cds
AT1G74890	-1.526333395	2.04E-16	Arabidopsis thaliana two-component response regulator ARR15 mRNA, complete cds
AT1G49320	-1.050495	2.39E-16	Arabidopsis thaliana BURP domain protein USPL1 mRNA, complete cds
AT2G25735	-1.415236178	2.42E-16	Arabidopsis thaliana chromosome 2, complete

			sequence
AT5G52570	-1.029855728	2.48E-16	Arabidopsis thaliana beta-carotene hydroxylase 2 mRNA, complete cds
AT3G14680	-1.001665752	2.52E-16	Arabidopsis thaliana cytochrome P450, family 72, subfamily A, polypeptide 14 mRNA, complete cds
AT3G29670	-1.204863835	2.54E-16	Arabidopsis thaliana chromosome 3, complete sequence
AT1G49000	-1.66378041	2.57E-16	Arabidopsis thaliana chromosome 1 sequence
AT1G66800	-1.59773117	2.60E-16	Arabidopsis thaliana alcohol dehydrogenase-like protein mRNA, complete cds
AT1G50630	-1.011224473	2.90E-16	Arabidopsis thaliana uncharacterized protein mRNA, complete cds
AT3G60220	-1.505574835	4.06E-16	Arabidopsis thaliana chromosome 3, complete sequence
AT2G35710	-1.003488824	4.45E-16	Arabidopsis thaliana putative glucuronosyltransferase PGSIP8 mRNA, complete cds
AT1G76800	-2.072957258	5.87E-16	Arabidopsis thaliana chromosome 1 sequence
AT3G05890	-1.282540571	6.00E-16	Arabidopsis thaliana mRNA for low temperature and salt responsive protein LTI6B, complete cds, clone: RAFL16-06-D03
AT3G27950	-1.396700227	7.27E-16	Arabidopsis thaliana GDSL esterase/lipase mRNA, complete cds
AT5G02090	-1.704530701	7.71E-16	Arabidopsis thaliana chromosome 5 sequence
AT2G25090	-2.930060476	9.37E-16	Arabidopsis thaliana SNF1-related kinase mRNA, complete cds
AT2G27000	-1.206041296	9.38E-16	Arabidopsis thaliana cytochrome P450, family 705, subfamily A, polypeptide 8 mRNA, complete cds
AT2G42060	-1.083683665	1.04E-15	Arabidopsis thaliana chromosome 2, complete sequence
AT5G24100	-1.51300539	1.15E-15	Arabidopsis thaliana Leucine-rich repeat protein kinase family protein mRNA, complete cds
AT1G30750	-1.379422236	1.35E-15	Arabidopsis thaliana chromosome 1 sequence
AT1G29440	-1.384209283	1.51E-15	Arabidopsis thaliana chromosome 1 sequence
AT1G52565	-1.434015475	1.51E-15	Arabidopsis thaliana uncharacterized protein mRNA, complete cds
AT4G24700	-1.364010855	1.55E-15	Arabidopsis thaliana chromosome 4 sequence
AT3G16390	-1.51344267	2.96E-15	Arabidopsis thaliana Nitrile-specifier protein 3 mRNA, complete cds
AT2G31585	-1.176793069	3.66E-15	Arabidopsis thaliana chromosome 2, complete sequence
AT3G45700	-1.598256837	4.13E-15	Arabidopsis thaliana putative nitrate excretion transporter 5 mRNA, complete cds

AT4G38960	-1.062435931	4.66E-15	Arabidopsis thaliana B-box type zinc finger-containing protein mRNA, complete cds
AT5G05500	-1.37480856	5.56E-15	Arabidopsis thaliana chromosome 5 sequence
AT3G05155	-1.590219518	6.16E-15	Arabidopsis thaliana mRNA for hypothetical protein, complete cds, clone: RAFL14-21-B09
AT3G21330	-3.065761757	6.95E-15	Arabidopsis thaliana transcription factor bHLH87 mRNA, complete cds
AT4G10540	-2.863717981	8.28E-15	Arabidopsis thaliana Subtilase family protein mRNA, complete cds
AT4G01430	-1.280540994	8.90E-15	Arabidopsis thaliana nodulin MtN21-like transporter family protein mRNA, complete cds
AT5G15150	-1.184156457	1.12E-14	Arabidopsis thaliana mRNA for homeobox protein, complete cds, clone: RAFL16-61-C06
AT1G61590	-1.21926121	1.20E-14	Arabidopsis thaliana putative receptor-like cytoplasmic kinase mRNA, complete cds
AT1G29430	-1.196158394	1.20E-14	Arabidopsis thaliana chromosome 1 sequence
AT4G22810	-1.050393355	1.31E-14	Arabidopsis thaliana chromosome 4 sequence
AT3G24460	-1.149713535	1.58E-14	Arabidopsis thaliana Serinc-domain containing serine and sphingolipid biosynthesis protein mRNA, complete cds
AT5G39860	-1.316305218	1.65E-14	Arabidopsis thaliana bHLH transcription factor PRE1 mRNA, complete cds
AT5G38820	-3.657919759	1.69E-14	Arabidopsis thaliana putative amino acid transporter mRNA, complete cds
AT3G47210	-1.040075113	1.77E-14	Arabidopsis thaliana uncharacterized protein mRNA, complete cds
AT5G01330	-1.636224688	2.09E-14	Arabidopsis thaliana pyruvate decarboxylase-3 mRNA, complete cds
AT1G33817	-3.029235881	2.10E-14	Arabidopsis thaliana chromosome 1 sequence
AT3G18200	-1.530961501	2.35E-14	Arabidopsis thaliana nodulin MtN21-like transporter UMAMIT4 mRNA, complete cds
AT1G14260	-1.539593445	2.56E-14	Arabidopsis thaliana RING-variant domain-containing protein mRNA, complete cds
AT2G34180	-1.358170765	2.93E-14	Arabidopsis thaliana chromosome 2, complete sequence
AT2G18328	-1.031410252	3.17E-14	Arabidopsis thaliana chromosome 2, complete sequence
AT1G34315	-1.215942823	3.42E-14	Arabidopsis thaliana uncharacterized protein mRNA, complete cds
AT5G53200	-1.650724257	3.57E-14	Arabidopsis thaliana transcription factor TRY mRNA, complete cds
AT3G06145	-1.276328743	4.08E-14	Arabidopsis thaliana chromosome 3, complete sequence
AT3G56620	-1.2476594	4.31E-14	Arabidopsis thaliana nodulin MtN21-like

			transporter family protein mRNA, complete cds
AT3G11370	-1.730894606	4.90E-14	Arabidopsis thaliana chromosome 3, complete sequence
AT1G22250	-1.44012651	5.13E-14	Arabidopsis thaliana uncharacterized protein mRNA, complete cds
AT2G38940	-1.36121764	5.27E-14	Arabidopsis thaliana inorganic phosphate transporter 1-4 mRNA, complete cds
AT3G26610	-1.169191346	6.07E-14	Arabidopsis thaliana putative polygalacturonase / pectinase mRNA, complete cds
AT5G47550	-1.559750597	7.80E-14	Arabidopsis thaliana chromosome 5 sequence
AT4G13580	-1.736115749	7.81E-14	Arabidopsis thaliana chromosome 4 sequence
AT3G20940	-1.148862136	1.21E-13	Arabidopsis thaliana cytochrome P450, family 705, subfamily A, polypeptide 30 mRNA, complete cds
AT4G25220	-1.467356993	1.22E-13	Arabidopsis thaliana putative glycerol-3-phosphate transporter 2 mRNA, complete cds
AT2G18800	-1.64758455	1.38E-13	Arabidopsis thaliana probable xyloglucan endotransglucosylase/hydrolase protein 21 mRNA, complete cds
AT4G08040	-2.482601499	1.88E-13	Arabidopsis thaliana 1-aminocyclopropane-1-carboxylate synthase 11 mRNA, complete cds
AT2G25810	-1.061988594	1.93E-13	Arabidopsis thaliana aquaporin TIP4-1 mRNA, complete cds
AT3G14260	-1.466837103	1.97E-13	Arabidopsis thaliana LURP-one-related 11 protein mRNA, complete cds
AT1G62320	-1.858049231	2.32E-13	Arabidopsis thaliana ERD (early-responsive to dehydration stress) family protein mRNA, complete cds
AT5G15600	-1.44427338	2.36E-13	Arabidopsis thaliana protein SPIRAL1-like4 mRNA, complete cds
AT3G15620	-1.451415449	2.61E-13	Arabidopsis thaliana (6-4)DNA photolyase mRNA, complete cds
AT2G24980	-1.365885481	2.72E-13	Arabidopsis thaliana chromosome 2, complete sequence
AT5G51720	-1.155285251	3.74E-13	Arabidopsis thaliana CDGSH iron-sulfur domain-containing protein NEET mRNA, complete cds
AT5G47950	-1.482042423	3.84E-13	Arabidopsis thaliana chromosome 5 sequence
AT1G29450	-1.394563933	4.72E-13	Arabidopsis thaliana chromosome 1 sequence
AT3G21710	-1.04151421	5.47E-13	Arabidopsis thaliana uncharacterized protein mRNA, complete cds
AT1G65985	-1.043351571	7.27E-13	Arabidopsis thaliana uncharacterized protein

			mRNA, complete cds
AT4G31875	-1.305183605	8.96E-13	Arabidopsis thaliana chromosome 4 sequence
AT2G40110	-1.052278527	9.54E-13	Arabidopsis thaliana Yippee family putative zinc-binding protein mRNA, complete cds
AT5G01490	-1.481443172	9.94E-13	Arabidopsis thaliana vacuolar cation/proton exchanger 4 mRNA, complete cds
AT5G16480	-1.140034519	1.11E-12	Arabidopsis thaliana atypical dual-specificity phosphatase 5 mRNA, complete cds
AT5G01050	-1.546976333	1.85E-12	Arabidopsis thaliana laccase-9 mRNA, complete cds
AT4G15380	-1.185718299	2.13E-12	Arabidopsis thaliana cytochrome P450, family 705, subfamily A, polypeptide 4 mRNA, complete cds
AT1G04180	-1.299649817	2.29E-12	Arabidopsis thaliana flavin-containing monooxygenase YUCCA9 mRNA, complete cds
AT4G12050	-1.215878277	2.43E-12	Arabidopsis thaliana chromosome 4 sequence
AT3G15760	-1.167999951	3.12E-12	Arabidopsis thaliana uncharacterized protein mRNA, complete cds
AT1G48745	-1.193866582	3.17E-12	Arabidopsis thaliana chromosome 1 sequence
AT3G06390	-1.23915895	3.32E-12	Arabidopsis thaliana uncharacterized protein mRNA, complete cds
AT3G45060	-1.170451542	3.56E-12	Arabidopsis thaliana high affinity nitrate transporter 2.6 mRNA, complete cds
AT3G61270	-1.225466916	3.59E-12	Arabidopsis thaliana lipopolysaccharide-modifying domain-containing protein mRNA, complete cds
AT3G49860	-1.367124281	3.63E-12	Arabidopsis thaliana ADP-ribosylation factor-like A1B mRNA, complete cds
AT2G39040	-1.246334002	4.29E-12	Arabidopsis thaliana peroxidase 24 mRNA, complete cds
AT3G29035	-1.446253744	4.53E-12	Arabidopsis thaliana NAC domain-containing protein 3 mRNA, complete cds
AT3G48940	-2.323149599	4.64E-12	Arabidopsis thaliana Remorin-like protein mRNA, complete cds
AT5G02440	-1.175499752	4.66E-12	Arabidopsis thaliana uncharacterized protein mRNA, complete cds
AT1G31950	-1.110650712	5.28E-12	Arabidopsis thaliana terpenoid synthase 29 mRNA, complete cds
AT4G15160	-1.508307213	5.48E-12	Arabidopsis thaliana bifunctional inhibitor/lipid-transfer protein/seed storage 2S albumin-like protein mRNA, complete cds
AT5G54980	-1.028361785	5.50E-12	Arabidopsis thaliana uncharacterized protein mRNA, complete cds
AT3G50900	-1.281734126	7.32E-12	Arabidopsis thaliana chromosome 3, complete sequence

AT4G22640	-1.550068044	7.72E-12	Arabidopsis thaliana chromosome 4 sequence
AT3G21870	-1.061402217	8.07E-12	Arabidopsis thaliana cyclin p2;1 mRNA, complete cds
AT2G26710	-1.176793069	9.62E-12	Arabidopsis thaliana cytochrome P450 734A1 mRNA, complete cds
AT3G62680	-1.089608499	1.00E-11	Arabidopsis thaliana proline-rich protein 3 mRNA, complete cds
AT2G26215	-1.368953289	1.05E-11	Arabidopsis thaliana chromosome 2, complete sequence
AT3G52520	-1.342601962	1.07E-11	Arabidopsis thaliana chromosome 3, complete sequence
AT5G45120	-5.373190282	1.17E-11	Arabidopsis thaliana chromosome 5 sequence
AT4G01140	-1.886791616	1.33E-11	Arabidopsis thaliana chromosome 4 sequence
AT2G01818	-1.20092134	1.54E-11	Arabidopsis thaliana PLATZ transcription factor family protein mRNA, complete cds
AT1G01520	-2.024182653	1.95E-11	Arabidopsis thaliana transcription factor ASG4 mRNA, complete cds
AT1G65390	-1.121200743	2.50E-11	Arabidopsis thaliana protein PHLOEM protein 2-LIKE A5 mRNA, complete cds
AT2G29130	-1.101037231	2.67E-11	Arabidopsis thaliana laccase 2 mRNA, complete cds
AT3G06020	-1.343266645	3.08E-11	Arabidopsis thaliana chromosome 3, complete sequence
AT3G20130	-1.220813482	3.37E-11	Arabidopsis thaliana cytochrome P450, family 705, subfamily A, polypeptide 22 mRNA, complete cds
AT3G60070	-1.010927322	3.58E-11	Arabidopsis thaliana major facilitator protein mRNA, complete cds
AT4G01525	-1.67929341	3.75E-11	Arabidopsis thaliana chromosome 4 sequence
AT2G20520	-1.775052392	4.69E-11	Arabidopsis thaliana chromosome 2, complete sequence
AT3G49160	-1.441005666	4.91E-11	Arabidopsis thaliana pyruvate kinase-like protein mRNA, complete cds
AT2G45550	-1.406798674	5.22E-11	Arabidopsis thaliana cytochrome P450 76C4 mRNA, complete cds
AT3G56000	-1.172492932	5.67E-11	Arabidopsis thaliana cellulose synthase like A14 mRNA, complete cds
AT3G49845	-1.202434952	5.74E-11	Arabidopsis thaliana uncharacterized protein mRNA, complete cds
AT1G26790	-1.2822977	6.90E-11	Arabidopsis thaliana Dof-type zinc finger DNA-binding family protein mRNA, complete cds
AT1G11175	-1.038206034	7.22E-11	Arabidopsis thaliana chromosome 1 sequence
AT3G49190	-1.857175135	7.96E-11	Arabidopsis thaliana O-acyltransferase (WSD1-like) family protein mRNA, complete cds

AT3G03500	-1.458079179	9.68E-11	Arabidopsis thaliana TatD related DNase mRNA, complete cds
AT5G02020	-1.387689852	1.09E-10	Arabidopsis thaliana uncharacterized protein mRNA, complete cds
AT3G43890	-1.770023186	1.15E-10	Arabidopsis thaliana chromosome 3, complete sequence
AT1G07690	-2.939293755	1.19E-10	Arabidopsis thaliana uncharacterized protein mRNA, complete cds
AT5G38100	-1.642456641	1.30E-10	Arabidopsis thaliana putative S-adenosylmethionine-dependent methyltransferase mRNA, complete cds
AT1G08500	-1.208501929	1.35E-10	Arabidopsis thaliana early nodulin-like protein 18 mRNA, complete cds
AT5G62420	-3.498721164	1.72E-10	Arabidopsis thaliana aldo/keto reductase family protein mRNA, complete cds
AT2G19210	-1.533486582	1.85E-10	Arabidopsis thaliana putative leucine-rich repeat receptor-like protein kinase mRNA, complete cds
AT4G34790	-2.2962283	1.96E-10	Arabidopsis thaliana chromosome 4 sequence
AT2G15370	-1.546026879	2.01E-10	Arabidopsis thaliana probable fucosyltransferase 5 mRNA, complete cds
AT3G05150	-1.437320619	2.06E-10	Arabidopsis thaliana sugar transporter ERD6-like 8 mRNA, complete cds
AT1G52820	-1.487750046	2.08E-10	Arabidopsis thaliana putative 2-oxoglutarate-dependent dioxygenase mRNA, complete cds
AT5G55090	-1.66378041	2.22E-10	Arabidopsis thaliana chromosome 5 sequence
AT4G15740	-1.510125927	2.50E-10	Arabidopsis thaliana chromosome 4 sequence
AT1G55152	-1.001985846	4.12E-10	Arabidopsis thaliana chromosome 1 sequence
AT1G14185	-1.548474199	5.23E-10	Arabidopsis thaliana glucose-methanol-choline oxidoreductase-like protein mRNA, complete cds
AT1G10550	-1.65522565	5.39E-10	Arabidopsis thaliana probable xyloglucan endotransglucosylase/hydrolase protein 33 mRNA, complete cds
AT3G53840	-2.454327045	5.73E-10	Arabidopsis thaliana wall-associated receptor kinase-like 15 mRNA, complete cds
AT2G03200	-1.454327045	6.56E-10	Arabidopsis thaliana chromosome 2, complete sequence
AT2G46940	-1.591830568	6.62E-10	Arabidopsis thaliana uncharacterized protein mRNA, complete cds
AT1G15580	-2.373190282	6.75E-10	Arabidopsis thaliana auxin-responsive protein IAA5 mRNA, complete cds
AT5G52790	-1.16875575	6.94E-10	Arabidopsis thaliana CBS domain-containing protein with a domain of unknown function (DUF21) mRNA, complete cds

AT5G49270	-1.475637551	7.97E-10	Arabidopsis thaliana COBRA-like protein 9 mRNA, complete cds
AT3G51710	-1.294182407	8.37E-10	Arabidopsis thaliana D-mannose binding lectin protein with Apple-like carbohydrate-binding domain mRNA, complete cds
AT5G18010	-2.721113585	8.40E-10	Arabidopsis thaliana chromosome 5 sequence
AT2G31081	-2.235686758	8.66E-10	Arabidopsis thaliana chromosome 2, complete sequence
AT3G21340	-1.660002071	8.67E-10	Arabidopsis thaliana receptor-like protein kinase mRNA, complete cds
AT5G56100	-1.360274394	9.42E-10	Arabidopsis thaliana chromosome 5 sequence
AT1G56630	-2.431606968	9.58E-10	Arabidopsis thaliana alpha/beta-Hydrolases superfamily protein mRNA, complete cds
AT4G37235	-3.40561176	9.63E-10	Arabidopsis thaliana CASP-like protein mRNA, complete cds
AT1G47603	-1.510999494	1.05E-09	Arabidopsis thaliana purine permease 19 mRNA, complete cds
AT2G20550	-1.04304168	1.11E-09	Arabidopsis thaliana HSP40/DnaJ peptide-binding protein mRNA, complete cds
AT3G25790	-1.149646927	1.15E-09	Arabidopsis thaliana myb-like transcription factor family protein mRNA, complete cds
AT3G62950	-1.218092698	1.19E-09	Arabidopsis thaliana chromosome 3, complete sequence
AT3G06460	-1.421905567	1.26E-09	Arabidopsis thaliana chromosome 3, complete sequence
AT1G48930	-1.152545523	1.32E-09	Arabidopsis thaliana glycosyl hydrolase 9C1 mRNA, complete cds
AT5G42500	-1.349857778	1.35E-09	Arabidopsis thaliana chromosome 5 sequence
AT5G14020	-1.581183324	1.60E-09	Arabidopsis thaliana Endosomal targeting BRO1-like domain-containing protein mRNA, complete cds
AT2G22630	-1.511321201	1.70E-09	Arabidopsis thaliana agamous-like MADS-box protein AGL17 mRNA, complete cds
AT2G03720	-1.804529593	1.81E-09	Arabidopsis thaliana MORPHOGENESIS OF ROOT HAIR 6 mRNA, complete cds
AT5G49870	-1.420111329	2.03E-09	Arabidopsis thaliana Mannose-binding lectin superfamily protein mRNA, complete cds
AT3G61160	-1.039935769	2.29E-09	Arabidopsis thaliana shaggy-related protein kinase beta mRNA, complete cds
AT3G51360	-1.498721164	2.51E-09	Arabidopsis thaliana aspartyl protease family protein mRNA, complete cds
AT1G45015	-1.362219164	2.73E-09	Arabidopsis thaliana MD-2-related lipid recognition domain-containing protein mRNA, complete cds

AT1G74500	-1.138933525	2.86E-09	Arabidopsis thaliana transcription factor bHLH135 mRNA, complete cds
AT3G63470	-1.282146069	3.44E-09	Arabidopsis thaliana serine carboxypeptidase-like 40 mRNA, complete cds
AT1G62280	-1.055777668	4.00E-09	Arabidopsis thaliana S-type anion channel SLAH1 mRNA, complete cds
AT1G29418	-1.199160882	4.19E-09	Arabidopsis thaliana chromosome 1 sequence
AT5G42510	-1.266275078	4.29E-09	Arabidopsis thaliana chromosome 5 sequence
AT2G14100	-1.1195682	4.69E-09	Arabidopsis thaliana cytochrome P450, family 705, subfamily A, polypeptide 13 mRNA, complete cds
AT4G01350	-1.1195682	4.69E-09	Arabidopsis thaliana chromosome 4 sequence
AT1G02340	-1.406357146	4.75E-09	Arabidopsis thaliana transcription factor HFR1 mRNA, complete cds
AT3G60490	-1.300781786	4.98E-09	Arabidopsis thaliana chromosome 3, complete sequence
AT1G61750	-1.416259004	5.29E-09	Arabidopsis thaliana Receptor-like protein kinase-related protein mRNA, complete cds
AT2G38600	-1.108774646	5.58E-09	Arabidopsis thaliana HAD superfamily, subfamily IIIB acid phosphatase mRNA, complete cds
AT1G65970	-1.256150861	6.05E-09	Arabidopsis thaliana thioredoxin-dependent peroxidase 2 mRNA, complete cds
AT2G27420	-1.102792488	8.75E-09	Arabidopsis thaliana cysteine proteinase-like protein mRNA, complete cds
AT1G34510	-1.472489622	8.79E-09	Arabidopsis thaliana peroxidase 8 mRNA, complete cds
AT3G46400	-1.336449735	9.06E-09	Arabidopsis thaliana Leucine-rich repeat protein kinase family protein mRNA, complete cds
AT1G73280	-1.065761757	9.40E-09	Arabidopsis thaliana serine carboxypeptidase-like 3 mRNA, complete cds
AT1G11735	-3.271310668	9.52E-09	Arabidopsis thaliana chromosome 1 sequence
AT5G19790	-2.128771554	9.93E-09	Arabidopsis thaliana chromosome 5 sequence
AT5G65925	-1.136151085	1.08E-08	Arabidopsis thaliana chromosome 5 sequence
AT2G24400	-1.976042941	1.13E-08	Arabidopsis thaliana chromosome 2, complete sequence
AT2G32610	-1.976042941	1.13E-08	Arabidopsis thaliana cellulose synthase-like protein B1 mRNA, complete cds
AT4G14830	-1.61036652	1.17E-08	Arabidopsis thaliana chromosome 4 sequence
AT5G01610	-1.046513872	1.29E-08	Arabidopsis thaliana uncharacterized protein mRNA, complete cds
AT5G54690	-1.158177391	1.35E-08	Arabidopsis thaliana probable galacturonosyltransferase 12 mRNA, complete cds
AT1G30560	-2.235686758	1.37E-08	Arabidopsis thaliana chromosome 1 sequence
AT4G38390	-1.268601381	1.42E-08	Arabidopsis thaliana protein root hair specific 17

			mRNA, complete cds
AT3G26818	-1.696167228	1.49E-08	Arabidopsis thaliana chromosome 3, complete sequence
AT5G18340	-1.721113585	1.57E-08	Arabidopsis thaliana U-box domain-containing protein 48 mRNA, complete cds
AT1G33813	-3.235686758	1.68E-08	Arabidopsis thaliana chromosome 1 sequence
AT5G56080	-1.875284515	1.81E-08	Arabidopsis thaliana chromosome 5 sequence
AT4G25790	-1.313366122	1.85E-08	Arabidopsis thaliana allergen V5/Tpx-1-related family protein mRNA, complete cds
AT1G53700	-1.634604592	2.02E-08	Arabidopsis thaliana chromosome 1 sequence
AT4G14548	-1.703835594	2.40E-08	Arabidopsis thaliana chromosome 4 sequence
AT4G22520	-1.703835594	2.40E-08	Arabidopsis thaliana chromosome 4 sequence
AT2G32620	-2.029235881	2.71E-08	Arabidopsis thaliana cellulose synthase-like protein B2 mRNA, complete cds
AT3G23630	-1.820649259	2.78E-08	Arabidopsis thaliana chromosome 3, complete sequence
AT3G61410	-1.934820279	2.84E-08	Arabidopsis thaliana uncharacterized protein mRNA, complete cds
AT4G12360	-1.893580781	2.85E-08	Arabidopsis thaliana Bifunctional inhibitor/lipid-transfer protein/seed storage 2S albumin superfamily protein mRNA, complete cds
AT5G10946	-1.126752387	3.07E-08	Arabidopsis thaliana uncharacterized protein mRNA, complete cds
AT3G48410	-1.002196628	3.13E-08	Arabidopsis thaliana alpha/beta-hydrolase domain-containing protein mRNA, complete cds
AT4G22630	-4.820649259	3.19E-08	Arabidopsis thaliana bifunctional inhibitor/lipid-transfer protein/seed storage 2S albumin superfamily protein mRNA, complete cds
AT3G62210	-1.162930416	3.22E-08	Arabidopsis thaliana putative endonuclease or glycosyl hydrolase mRNA, complete cds
AT1G52240	-1.185060685	3.49E-08	Arabidopsis thaliana RHO guanyl-nucleotide exchange factor 11 mRNA, complete cds
AT5G46040	-1.739729263	4.03E-08	Arabidopsis thaliana peptide transporter PTR3-B mRNA, complete cds
AT1G27140	-1.769368754	4.19E-08	Arabidopsis thaliana glutathione S-transferase tau 14 mRNA, complete cds
AT1G15830	-1.769368754	4.19E-08	Arabidopsis thaliana chromosome 1 sequence
AT2G14210	-1.801283934	4.32E-08	Arabidopsis thaliana protein agamous-like 44 mRNA, complete cds
AT5G18060	-1.801283934	4.32E-08	Arabidopsis thaliana chromosome 5 sequence
AT4G29450	-1.913758663	4.49E-08	Arabidopsis thaliana leucine-rich repeat protein kinase-like protein mRNA, complete cds
AT3G52480	-1.03774738	4.78E-08	Arabidopsis thaliana chromosome 3, complete sequence

AT4G22460	-1.514151204	4.93E-08	Arabidopsis thaliana chromosome 4 sequence
AT2G47540	-1.360014893	6.03E-08	Arabidopsis thaliana pollen Ole e 1 allergen and extensin family protein mRNA, complete cds
AT2G13910	-1.781655127	6.70E-08	Arabidopsis thaliana chromosome 2, complete sequence
AT3G50640	-1.369823431	6.76E-08	Arabidopsis thaliana uncharacterized protein mRNA, complete cds
AT5G35480	-1.585135916	6.88E-08	Arabidopsis thaliana chromosome 5 sequence
AT4G18510	-1.428331836	7.21E-08	Arabidopsis thaliana chromosome 4 sequence
AT1G73290	-2.913758663	7.80E-08	Arabidopsis thaliana serine carboxypeptidase-like 5 mRNA, complete cds
AT1G23160	-2.292270286	8.04E-08	Arabidopsis thaliana auxin-responsive GH3 family protein mRNA, complete cds
AT5G43370	-1.241563324	8.19E-08	Arabidopsis thaliana phosphate transporter Pht1;2 mRNA, complete cds
AT1G65870	-1.34671807	8.68E-08	Arabidopsis thaliana chromosome 1 sequence
AT3G01760	-2.136151085	9.72E-08	Arabidopsis thaliana Lysine histidine transporter-like 4 mRNA, complete cds
AT4G28720	-1.730894606	9.95E-08	Arabidopsis thaliana chromosome 4 sequence
AT5G18080	-1.253987575	1.06E-07	Arabidopsis thaliana chromosome 5 sequence
AT2G47485	-1.483124309	1.08E-07	Arabidopsis thaliana chromosome 2, complete sequence
AT2G03540	-1.961064378	1.10E-07	Arabidopsis thaliana chromosome 2, complete sequence
AT1G60190	-1.324390983	1.10E-07	Arabidopsis thaliana chromosome 1 sequence
AT4G11780	-1.498721164	1.18E-07	Arabidopsis thaliana uncharacterized protein mRNA, complete cds
AT2G45210	-1.076488163	1.26E-07	Arabidopsis thaliana chromosome 2, complete sequence
AT1G56680	-1.12580914	1.33E-07	Arabidopsis thaliana Chitinase family protein mRNA, complete cds
AT5G06640	-1.241812861	1.49E-07	Arabidopsis thaliana chromosome 5 sequence
AT3G04860	-1.190598869	1.51E-07	Arabidopsis thaliana uncharacterized protein mRNA, complete cds
AT4G00680	-1.217912056	1.61E-07	Arabidopsis thaliana actin depolymerizing factor 8 mRNA, complete cds
AT4G02970	-1.081215409	1.68E-07	Arabidopsis thaliana signal recognition particle mRNA, complete cds
AT4G22790	-1.15079786	1.68E-07	Arabidopsis thaliana chromosome 4 sequence
AT1G44160	-1.281909775	1.76E-07	Arabidopsis thaliana HSP40/DnaJ peptide-binding protein mRNA, complete cds
AT2G35000	-1.004524127	1.79E-07	Arabidopsis thaliana chromosome 2, complete sequence

AT5G44417	-2.235686758	2.19E-07	Arabidopsis thaliana chromosome 5 sequence
AT4G28850	-1.384388488	2.21E-07	Arabidopsis thaliana xyloglucan endotransglucosylase/hydrolase 26 mRNA, complete cds
AT1G62980	-1.043638946	2.35E-07	Arabidopsis thaliana expansin A18 mRNA, complete cds
AT4G15680	-1.211439212	2.57E-07	Arabidopsis thaliana chromosome 4 sequence
AT1G35180	-1.03240316	2.74E-07	Arabidopsis thaliana TRAM, LAG1 and CLN8 (TLC) lipid-sensing domain containing protein mRNA, complete cds
AT1G29510	-1.276328743	2.82E-07	Arabidopsis thaliana chromosome 1 sequence
AT1G74930	-1.482042423	2.83E-07	Arabidopsis thaliana chromosome 1 sequence
AT4G31940	-5.557614853	3.01E-07	Arabidopsis thaliana cytochrome P450, family 82, subfamily C, polypeptide 4 mRNA, complete cds
AT2G37700	-1.284127113	3.20E-07	Arabidopsis thaliana protein CER1-like 2 mRNA, complete cds
AT2G29740	-1.369438147	3.20E-07	Arabidopsis thaliana chromosome 2, complete sequence
AT5G44690	-4.614198381	3.26E-07	Arabidopsis thaliana uncharacterized protein mRNA, complete cds
AT2G24240	-1.094330909	3.42E-07	Arabidopsis thaliana chromosome 2, complete sequence
AT3G47965	-1.078503426	3.50E-07	Arabidopsis thaliana uncharacterized protein mRNA, complete cds
AT1G52550	-1.13239895	3.67E-07	Arabidopsis thaliana uncharacterized protein mRNA, complete cds
AT1G15825	-2.801283934	4.09E-07	Arabidopsis thaliana chromosome 1 sequence
AT4G24180	-1.766201475	4.11E-07	Arabidopsis thaliana thaumatin-like protein 1 mRNA, complete cds
AT4G02090	-1.766201475	4.11E-07	Arabidopsis thaliana chromosome 4 sequence
AT5G06630	-1.204659862	4.11E-07	Arabidopsis thaliana chromosome 5 sequence
AT4G30320	-1.939293755	4.35E-07	Arabidopsis thaliana chromosome 4 sequence
AT5G43040	-1.235686758	4.35E-07	Arabidopsis thaliana chromosome 5 sequence
AT1G61840	-1.889511117	4.36E-07	Arabidopsis thaliana chromosome 1 sequence
AT4G30420	-1.576723676	4.38E-07	Arabidopsis thaliana nodulin MtN21-like transporter family protein mRNA, complete cds
AT4G09990	-1.242381411	4.96E-07	Arabidopsis thaliana uncharacterized protein mRNA, complete cds
AT3G49330	-1.365454633	5.18E-07	Arabidopsis thaliana chromosome 3, complete sequence
AT2G23630	-1.216321433	5.36E-07	Arabidopsis thaliana protein SKU5 similar 16 mRNA, complete cds
AT1G01390	-1.027578563	5.69E-07	Arabidopsis thaliana chromosome 1 sequence

AT1G19320	-3.282992473	5.79E-07	Arabidopsis thaliana pathogenesis-related thaumatin-like protein mRNA, complete cds
AT4G37700	-2.176793069	5.88E-07	Arabidopsis thaliana chromosome 4 sequence
AT1G48220	-3.721113585	6.03E-07	Arabidopsis thaliana protein kinase family prtein mRNA, complete cds
AT2G43880	-1.072957258	6.33E-07	Arabidopsis thaliana putative polygalacturonase /pectinase mRNA, complete cds
AT2G26211	-2.029235881	6.62E-07	Arabidopsis thaliana chromosome 2, complete sequence
AT2G16230	-1.864849063	6.87E-07	Arabidopsis thaliana O-glycosyl hydrolases family 17 protein mRNA, complete cds
AT5G42630	-1.174286213	6.97E-07	Arabidopsis thaliana probable transcription factor KAN4 mRNA, complete cds
AT1G17147	-1.580021266	7.11E-07	Arabidopsis thaliana chromosome 1 sequence
AT1G13600	-1.349857778	7.46E-07	Arabidopsis thaliana chromosome 1 sequence
AT5G59260	-1.030942203	7.61E-07	Arabidopsis thaliana chromosome 5 sequence
AT3G05727	-1.604074164	7.67E-07	Arabidopsis thaliana defensin-like protein 204 mRNA, complete cds
AT5G40310	-2.340023418	7.79E-07	Arabidopsis thaliana exonuclease family protein mRNA, complete cds
AT5G22870	-1.629965697	8.22E-07	Arabidopsis thaliana chromosome 5 sequence
AT5G37478	-1.657919759	8.76E-07	Arabidopsis thaliana TPX2 (targeting protein for Xklp2) family protein mRNA, complete cds
AT2G19970	-1.006204912	8.86E-07	Arabidopsis thaliana chromosome 2, complete sequence
AT3G07070	-1.373190282	9.39E-07	Arabidopsis thaliana serine/threonine-protein kinase mRNA, complete cds
AT3G06990	-1.161686177	9.67E-07	Arabidopsis thaliana cysteine/histidine-rich C1 domain-containing protein mRNA, complete cds
AT1G79220	-1.538249528	9.81E-07	Arabidopsis thaliana chromosome 1 sequence
AT1G55280	-1.049110516	1.01E-06	Arabidopsis thaliana Lipase/lipooxygenase, PLAT/LH2 family protein mRNA, complete cds
AT5G42610	-2.001221504	1.06E-06	Arabidopsis thaliana uncharacterized protein mRNA, complete cds
AT3G23930	-1.334090462	1.07E-06	Arabidopsis thaliana uncharacterized protein mRNA, complete cds
AT1G29230	-1.839758082	1.08E-06	Arabidopsis thaliana chromosome 1 sequence
AT4G20820	-1.195794031	1.20E-06	Arabidopsis thaliana chromosome 4 sequence
AT5G11180	-2.306076086	1.30E-06	Arabidopsis thaliana glutamate receptor 2.6 mRNA, complete cds
AT3G24110	-1.308037602	1.36E-06	Arabidopsis thaliana putative calcium-binding protein CML22 mRNA, complete cds
AT2G23410	-2.20326528	1.45E-06	Arabidopsis thaliana cis-prenyltransferase mRNA, complete cds

AT5G51870	-2.20326528	1.45E-06	Arabidopsis thaliana MADS-box transcription factor AGL71 mRNA, complete cds
AT5G52260	-1.318148918	1.54E-06	Arabidopsis thaliana myb domain protein 19 mRNA, complete cds
AT4G11020	-2.913758663	1.55E-06	Arabidopsis thaliana uncharacterized protein mRNA, complete cds
AT2G03260	-1.055777668	1.55E-06	Arabidopsis thaliana phosphate transporter PHO1-like 2 mRNA, complete cds
AT1G04540	-1.025651543	1.58E-06	Arabidopsis thaliana chromosome 1 sequence
AT4G36060	-1.131938833	1.61E-06	Arabidopsis thaliana transcription factor bHLH11 mRNA, complete cds
AT2G34000	-1.771739658	1.64E-06	Arabidopsis thaliana chromosome 2, complete sequence
AT3G58850	-1.282992473	1.71E-06	Arabidopsis thaliana chromosome 3, complete sequence
AT2G45890	-1.092728804	1.72E-06	Arabidopsis thaliana mRNA for hypothetical protein, complete cds, clone: RAFL16-63-K09
AT3G61930	-3.614198381	1.96E-06	Arabidopsis thaliana chromosome 3, complete sequence
AT1G04610	-1.614198381	2.02E-06	Arabidopsis thaliana flavin-containing monooxygenase YUCCA3 mRNA, complete cds
AT5G43540	-1.258894149	2.14E-06	Arabidopsis thaliana chromosome 5 sequence
AT1G64910	-1.643111073	2.16E-06	Arabidopsis thaliana chromosome 1 sequence
AT5G38970	-1.425657702	2.37E-06	Arabidopsis thaliana brassinosteroid-6-oxidase 1 mRNA, complete cds
AT5G28010	-2.170098416	2.37E-06	Arabidopsis thaliana polyketide cyclase/dehydrase and lipid transport superfamily protein mRNA, complete cds
AT5G26300	-1.145084209	2.44E-06	Arabidopsis thaliana TRAF-like family protein mRNA, complete cds
AT2G04800	-1.00256793	2.46E-06	Arabidopsis thaliana chromosome 2, complete sequence
AT5G44585	-1.442137636	2.63E-06	Arabidopsis thaliana uncharacterized protein mRNA, complete cds
AT2G20080	-2.008915896	2.65E-06	Arabidopsis thaliana uncharacterized protein mRNA, complete cds
AT3G09925	-1.149826021	2.81E-06	Arabidopsis thaliana pollen Ole e 1 allergen and extensin family protein mRNA, complete cds
AT2G17036	-1.591830568	3.05E-06	Arabidopsis thaliana F-box protein mRNA, complete cds
AT1G30757	-1.591830568	3.05E-06	Arabidopsis thaliana chromosome 1 sequence
AT5G58360	-1.478543282	3.21E-06	Arabidopsis thaliana chromosome 5 sequence
AT1G23760	-1.478543282	3.21E-06	Arabidopsis thaliana putative polygalacturonase non-catalytic subunit JP630 mRNA, complete cds

AT1G51550	-1.019800301	3.30E-06	Arabidopsis thaliana F-box/kelch-repeat protein mRNA, complete cds
AT5G14750	-1.406798674	3.45E-06	Arabidopsis thaliana transcription factor WER mRNA, complete cds
AT1G66440	-1.422772311	3.85E-06	Arabidopsis thaliana chromosome 1 sequence
AT4G37850	-1.721113585	3.91E-06	Arabidopsis thaliana transcription factor bHLH25 mRNA, complete cds
AT1G18290	-1.913758663	4.30E-06	Arabidopsis thaliana chromosome 1 sequence
AT2G15128	-1.122345285	5.34E-06	Arabidopsis thaliana chromosome 2, complete sequence
AT1G26200	-1.403143504	5.62E-06	Arabidopsis thaliana TRAM, LAG1 and CLN8 (TLC) lipid-sensing domain containing protein mRNA, complete cds
AT2G27740	-1.027216713	5.91E-06	Arabidopsis thaliana uncharacterized protein mRNA, complete cds
AT5G35870	-2.591830568	6.21E-06	Arabidopsis thaliana chromosome 5 sequence
AT5G47635	-2.101385666	6.33E-06	Arabidopsis thaliana pollen Ole e 1 allergen and extensin family protein mRNA, complete cds
AT3G01175	-1.828028789	6.73E-06	Arabidopsis thaliana uncharacterized protein mRNA, complete cds
AT5G39520	-1.946180141	6.79E-06	Arabidopsis thaliana uncharacterized protein mRNA, complete cds
AT2G43050	-1.546026879	6.87E-06	Arabidopsis thaliana putative pectinesterase/ pectinerase inhibitor 16 mRNA, complete cds
AT1G66400	-1.087090266	7.56E-06	Arabidopsis thaliana chromosome 1 sequence
AT4G31470	-1.601814657	8.06E-06	Arabidopsis thaliana chromosome 4 sequence
AT2G28860	-1.322564209	8.31E-06	Arabidopsis thaliana chromosome 2, complete sequence
AT2G16970	-1.112538527	8.46E-06	Arabidopsis thaliana tetracycline transporter-like protein 1 mRNA, complete cds
AT5G50560	-1.476694858	8.52E-06	Arabidopsis thaliana chromosome 5 sequence
AT1G19840	-1.235686758	8.93E-06	Arabidopsis thaliana chromosome 1 sequence
AT3G50220	-1.016852156	9.21E-06	Arabidopsis thaliana chromosome 3, complete sequence
AT3G59480	-1.750259931	1.02E-05	Arabidopsis thaliana probable fructokinase-4 mRNA, complete cds
AT1G46768	-1.416259004	1.02E-05	Arabidopsis thaliana chromosome 1 sequence
AT1G63600	-1.984147991	1.07E-05	Arabidopsis thaliana chromosome 1 sequence
AT3G21890	-1.913758663	1.08E-05	Arabidopsis thaliana chromosome 3, complete sequence
AT1G71740	-1.913758663	1.08E-05	Arabidopsis thaliana chromosome 1 sequence
AT5G04120	-4.235686758	1.13E-05	Arabidopsis thaliana phosphoglycerate mutase-like protein mRNA, complete cds

AT4G11490	-4.235686758	1.13E-05	Arabidopsis thaliana putative cysteine-rich receptor-like protein kinase 33 mRNA, complete cds
AT1G70110	-3.437320619	1.14E-05	Arabidopsis thaliana putative L-type lectin-domain containing receptor kinase V.1 mRNA, complete cds
AT3G23200	-1.102203753	1.34E-05	Arabidopsis thaliana uncharacterized protein mRNA, complete cds
AT1G27045	-5.083683665	1.58E-05	Arabidopsis thaliana homeobox-leucine zipper protein ATHB-54 mRNA, complete cds
AT5G40630	-2.029235881	1.66E-05	Arabidopsis thaliana AT5G40630 mRNA, complete cds, clone: RAFL25-37-N20
AT5G19410	-1.284596359	1.70E-05	Arabidopsis thaliana chromosome 5 sequence
AT4G04293	-1.949382573	1.71E-05	Arabidopsis thaliana chromosome 4 sequence
AT5G22355	-2.972652352	1.80E-05	Arabidopsis thaliana chromosome 5 sequence
AT5G16080	-1.244964572	1.87E-05	Arabidopsis thaliana chromosome 5 sequence
AT1G06660	-1.170098416	1.88E-05	Arabidopsis thaliana protein JASON mRNA, complete cds
AT3G18400	-1.170098416	1.88E-05	Arabidopsis thaliana NAC domain containing protein 58 mRNA, complete cds
AT1G44608	-1.357365315	1.93E-05	Arabidopsis thaliana uncharacterized protein mRNA, complete cds
AT3G23450	-4.161686177	2.05E-05	Arabidopsis thaliana chromosome 3, complete sequence
AT1G69240	-1.452178578	2.05E-05	Arabidopsis thaliana methyl esterase 15 mRNA, complete cds
AT1G24580	-1.452178578	2.05E-05	Arabidopsis thaliana chromosome 1 sequence
AT4G12330	-1.498721164	2.50E-05	Arabidopsis thaliana cytochrome P450, family 706, subfamily A, polypeptide 7 mRNA, complete cds
AT1G27670	-1.498721164	2.50E-05	Arabidopsis thaliana chromosome 1 sequence
AT3G12540	-2.083683665	2.58E-05	Arabidopsis thaliana uncharacterized protein mRNA, complete cds
AT1G10460	-1.153224598	2.61E-05	Arabidopsis thaliana chromosome 1 sequence
AT1G11740	-1.991761175	2.68E-05	Arabidopsis thaliana ankyrin repeat family protein mRNA, complete cds
AT1G09610	-1.913758663	2.72E-05	Arabidopsis thaliana chromosome 1 sequence
AT4G22214	-5.001221504	2.83E-05	Arabidopsis thaliana defensin-like protein 99 mRNA, complete cds
AT4G39000	-5.001221504	2.83E-05	Arabidopsis thaliana glycosyl hydrolase 9B17 mRNA, complete cds
AT1G03935	-1.191292639	2.84E-05	Arabidopsis thaliana chromosome 1 sequence
AT5G66815	-2.449811563	3.08E-05	Arabidopsis thaliana chromosome 5 sequence
AT1G23965	-2.449811563	3.08E-05	Arabidopsis thaliana chromosome 1 sequence

AT3G27970	-1.130570052	3.14E-05	Arabidopsis thaliana exonuclease-like protein mRNA, complete cds
AT3G60700	-2.913758663	3.17E-05	Arabidopsis thaliana uncharacterized protein mRNA, complete cds
AT3G56730	-1.449811563	3.35E-05	Arabidopsis thaliana putative endonuclease or glycosyl hydrolase mRNA, complete cds
AT3G21090	-1.166145825	3.45E-05	Arabidopsis thaliana ABC transporter G family member 15 mRNA, complete cds
AT5G59070	-1.136151085	3.62E-05	Arabidopsis thaliana glycosyl transferase family protein mRNA, complete cds
AT3G21500	-3.306076086	3.69E-05	Arabidopsis thaliana 1-deoxy-D-xylulose 5-phosphate synthase 1 mRNA, complete cds
AT4G14380	-1.473186072	3.71E-05	Arabidopsis thaliana chromosome 4 sequence
AT2G41970	-1.207489866	3.72E-05	Arabidopsis thaliana putative protein kinase mRNA, complete cds
AT1G68510	-4.083683665	3.73E-05	Arabidopsis thaliana LOB domain-containing protein 42 mRNA, complete cds
AT5G19560	-1.705172041	3.94E-05	Arabidopsis thaliana ROP uanine nucleotide exchange factor 10 mRNA, complete cds
AT3G20160	-2.15079786	3.95E-05	Arabidopsis thaliana chromosome 3, complete sequence
AT1G68238	-1.142027651	4.17E-05	Arabidopsis thaliana chromosome 1 sequence
AT1G62370	-1.142027651	4.17E-05	Arabidopsis thaliana chromosome 1 sequence
AT4G01890	-1.811879049	4.25E-05	Arabidopsis thaliana glycoside hydrolase family 28 protein / polygalacturonase (pectinase) family protein mRNA, complete cds
AT2G17330	-1.953287027	4.30E-05	Arabidopsis thaliana chromosome 2, complete sequence
AT5G39160	-1.953287027	4.30E-05	Arabidopsis thaliana germin-like protein subfamily 1 member 18 mRNA, complete cds
AT1G33320	-1.953287027	4.30E-05	Arabidopsis thaliana Pyridoxal phosphate (PLP)-dependent transferases superfamily protein mRNA, complete cds
AT3G20935	-1.40408429	4.43E-05	Arabidopsis thaliana cytochrome P450, family 705, subfamily A, polypeptide 28 mRNA, complete cds
AT2G04795	-1.021818409	4.67E-05	Arabidopsis thaliana chromosome 2, complete sequence
AT4G15690	-1.021818409	4.67E-05	Arabidopsis thaliana chromosome 4 sequence
AT1G58150	-1.003026001	4.67E-05	Arabidopsis thaliana uncharacterized protein mRNA, complete cds
AT1G05530	-1.557614853	4.88E-05	Arabidopsis thaliana chromosome 1 sequence
AT1G29500	-1.118873093	5.00E-05	Arabidopsis thaliana chromosome 1 sequence
AT2G19660	-1.344393018	5.12E-05	Arabidopsis thaliana chromosome 2, complete

			sequence
AT5G19870	-1.188381043	5.22E-05	Arabidopsis thaliana chromosome 5 sequence
AT1G49390	-1.188381043	5.22E-05	Arabidopsis thaliana 2-oxoglutarate (2OG) and Fe(II)-dependent oxygenase-like protein mRNA, complete cds
AT2G25160	-1.591830568	5.29E-05	Arabidopsis thaliana cytochrome P450, family 82, subfamily F, polypeptide 1 mRNA, complete cds
AT3G07970	-1.447190863	5.49E-05	Arabidopsis thaliana polygalacturonase QRT2 mRNA, complete cds
AT3G44510	-1.447190863	5.49E-05	Arabidopsis thaliana alpha/beta-hydrolases family protein mRNA, complete cds
AT4G37240	-1.124325649	5.77E-05	Arabidopsis thaliana chromosome 4 sequence
AT3G50800	-1.246334002	6.35E-05	Arabidopsis thaliana chromosome 3, complete sequence
AT1G35330	-1.721113585	6.39E-05	Arabidopsis thaliana RING-H2 finger protein ATL34 mRNA, complete cds
AT2G20835	-2.106403741	6.47E-05	Arabidopsis thaliana chromosome 2, complete sequence
AT3G52970	-3.235686758	6.63E-05	Arabidopsis thaliana cytochrome P450, family 76, subfamily G, polypeptide 1 mRNA, complete cds
AT1G02630	-1.839758082	6.83E-05	Arabidopsis thaliana equilibrative nucleotide transporter 8 mRNA, complete cds
AT1G14220	-1.39918549	7.24E-05	Arabidopsis thaliana ribonuclease T2 mRNA, complete cds
AT5G51520	-1.320934045	7.39E-05	Arabidopsis thaliana chromosome 5 sequence
AT1G74453	-1.420718652	8.08E-05	Arabidopsis thaliana chromosome 1 sequence
AT3G18010	-1.054621199	8.34E-05	Arabidopsis thaliana WUSCHEL-related homeobox 1 mRNA, complete cds
AT3G16150	-1.03194009	8.43E-05	Arabidopsis thaliana probable isoaspartyl peptidase/L-asparaginase 2 mRNA, complete cds
AT5G65800	-2.34671807	8.80E-05	Arabidopsis thaliana mRNA for 1-aminocyclopropane-1-carboxylate synthase ACS5, complete cds, clone: RAFL16-37-F05
AT5G39865	-1.44427338	8.99E-05	Arabidopsis thaliana chromosome 5 sequence
AT3G62760	-1.013294337	9.82E-05	Arabidopsis thaliana glutathione S-transferase F13 mRNA, complete cds
AT2G28270	-1.739729263	0.000103798	Arabidopsis thaliana chromosome 2, complete sequence
AT3G59850	-1.297087303	0.000106331	Arabidopsis thaliana pectin lyase-like superfamily protein mRNA, complete cds
AT3G47347	-1.801283934	0.000107532	Arabidopsis thaliana chromosome 3, complete sequence
AT2G44010	-1.088256394	0.000109424	Arabidopsis thaliana chromosome 2, complete sequence

AT4G03330	-1.03774738	0.000113249	Arabidopsis thaliana syntaxin-123 mRNA, complete cds
AT3G22470	-1.015638277	0.000113896	Arabidopsis thaliana chromosome 3, complete sequence
AT1G66470	-1.393751604	0.000118589	Arabidopsis thaliana protein ROOT HAIR DEFECTIVE6 mRNA, complete cds
AT4G31877	-3.161686177	0.000118926	Arabidopsis thaliana mRNA for unknown protein, complete cds, clone: RAFL17-06-E12
AT5G11440	-3.913758663	0.000124369	Arabidopsis thaliana CTC-interacting domain 5 mRNA, complete cds
AT3G49820	-3.913758663	0.000124369	Arabidopsis thaliana chromosome 3, complete sequence
AT1G73120	-3.913758663	0.000124369	Arabidopsis thaliana uncharacterized protein mRNA, complete cds
AT5G53380	-2.498721164	0.00012589	Arabidopsis thaliana O-acyltransferase (WSD1-like) family protein mRNA, complete cds
AT1G63450	-1.093082363	0.000126523	Arabidopsis thaliana chromosome 1 sequence
AT5G28960	-1.018095323	0.000132158	Arabidopsis thaliana uncharacterized protein mRNA, complete cds
AT1G28040	-1.416259004	0.000132513	Arabidopsis thaliana RING-H2 finger protein ATL20 mRNA, complete cds
AT5G09978	-1.2595335	0.000133739	Arabidopsis thaliana elicitor peptide 7 mRNA, complete cds
AT1G13635	-1.164302125	0.000134779	Arabidopsis thaliana putative 3-methyladenine glycosylase I mRNA, complete cds
AT5G63090	-1.213318945	0.000145271	Arabidopsis thaliana protein LATERAL ORGAN BOUNDARIES mRNA, complete cds
AT5G43240	-2.292270286	0.000147917	Arabidopsis thaliana uncharacterized protein mRNA, complete cds
AT1G55390	-1.468347515	0.000163533	Arabidopsis thaliana chromosome 1 sequence
AT1G76430	-1.76175557	0.0001685	Arabidopsis thaliana putative inorganic phosphate transporter 1-9 mRNA, complete cds
AT2G45580	-2.721113585	0.000171084	Arabidopsis thaliana cytochrome P450 76C3 mRNA, complete cds
AT1G02575	-2.013294337	0.000171793	Arabidopsis thaliana uncharacterized protein mRNA, complete cds
AT2G03600	-2.013294337	0.000171793	Arabidopsis thaliana ureide permease 3 mRNA, complete cds
AT5G02420	-1.287217059	0.000173197	Arabidopsis thaliana chromosome 5 sequence
AT3G15250	-1.831296503	0.000173421	Arabidopsis thaliana chromosome 3, complete sequence
AT5G36925	-1.074223335	0.000173545	Arabidopsis thaliana uncharacterized protein mRNA, complete cds
AT4G37950	-1.913758663	0.000174874	Arabidopsis thaliana Rhamnogalacturonate lyase

			family protein mRNA, complete cds
AT4G21590	-1.04761441	0.000176465	Arabidopsis thaliana endonuclease 3 mRNA, complete cds
AT5G06900	-1.498721164	0.000180376	Arabidopsis thaliana cytochrome P450, family 93, subfamily D, polypeptide 1 mRNA, complete cds
AT3G07900	-1.302800954	0.000196612	Arabidopsis thaliana O-fucosyltransferase-like protein mRNA, complete cds
AT4G23590	-1.302800954	0.000196612	Arabidopsis thaliana probable aminotransferase TAT4 mRNA, complete cds
AT5G59490	-1.190598869	0.00020463	Arabidopsis thaliana haloacid dehalogenase-like hydrolase (HAD) superfamily protein mRNA, complete cds
AT5G16410	-1.003395876	0.000206963	Arabidopsis thaliana HXXXD-type acyl-transferase-like protein mRNA, complete cds
AT2G06002	-3.083683665	0.00021307	Arabidopsis thaliana chromosome 2, complete sequence
AT1G59218	-1.57087095	0.000215394	Arabidopsis thaliana putative disease resistance protein RDL6/RF9 mRNA, complete cds
AT4G05130	-1.115392524	0.000225741	Arabidopsis thaliana equilibrative nucleoside transporter 4 mRNA, complete cds
AT1G47590	-1.614198381	0.000232628	Arabidopsis thaliana chromosome 1 sequence
AT4G15417	-1.200639811	0.000234863	Arabidopsis thaliana protein RNAse II-like 1 mRNA, complete cds
AT1G05135	-1.338256492	0.000251825	Arabidopsis thaliana chromosome 1 sequence
AT4G09130	-2.083683665	0.000268085	Arabidopsis thaliana chromosome 4 sequence
AT3G44326	-1.466299686	0.000268975	Arabidopsis thaliana chromosome 3, complete sequence
AT1G75720	-1.466299686	0.000268975	Arabidopsis thaliana uncharacterized protein mRNA, complete cds
AT3G45330	-1.211439212	0.000269336	Arabidopsis thaliana chromosome 3, complete sequence
AT5G28610	-1.358543506	0.000283954	Arabidopsis thaliana chromosome 5 sequence
AT1G11915	-1.358543506	0.000283954	Arabidopsis thaliana uncharacterized protein mRNA, complete cds
AT5G39000	-1.128771554	0.000301085	Arabidopsis thaliana chromosome 5 sequence
AT2G34330	-4.614198381	0.000303997	Arabidopsis thaliana chromosome 2, complete sequence
AT4G34410	-1.380884674	0.000319238	Arabidopsis thaliana chromosome 4 sequence
AT1G49210	-1.380884674	0.000319238	Arabidopsis thaliana chromosome 1 sequence
AT2G39370	-1.292270286	0.000321098	Arabidopsis thaliana chromosome 2, complete sequence
AT3G56230	-1.035749188	0.000322934	Arabidopsis thaliana BTB/POZ domain-containing protein mRNA, complete cds
AT5G56990	-1.010620202	0.000324437	Arabidopsis thaliana uncharacterized protein

			mRNA, complete cds
AT3G21680	-1.53524704	0.000325549	Arabidopsis thaliana chromosome 3, complete sequence
AT3G12820	-1.176793069	0.000330296	Arabidopsis thaliana myb domain protein 10 mRNA, complete cds
AT3G03850	-1.136151085	0.000347546	Arabidopsis thaliana chromosome 3, complete sequence
AT4G33880	-1.136151085	0.000347546	Arabidopsis thaliana mRNA for putative bHLH transcription factor, complete cds, clone: RAFL14-58-P18
AT4G01925	-1.40561176	0.000357675	Arabidopsis thaliana cysteine/histidine-rich C1 domain-containing protein mRNA, complete cds
AT1G59725	-2.373190282	0.000366125	Arabidopsis thaliana putative DNAJ heat shock protein mRNA, complete cds
AT5G46130	-2.373190282	0.000366125	Arabidopsis thaliana uncharacterized protein mRNA, complete cds
AT2G36110	-1.013294337	0.000376962	Arabidopsis thaliana chromosome 2, complete sequence
AT5G24910	-1.186777158	0.000379779	Arabidopsis thaliana cytochrome P450, family 714, subfamily A, polypeptide 1 mRNA, complete cds
AT1G17300	-1.186777158	0.000379779	Arabidopsis thaliana uncharacterized protein mRNA, complete cds
AT4G22080	-1.433132822	0.000399128	Arabidopsis thaliana root hair specific 14 mRNA, complete cds
AT1G76220	-2.176793069	0.000412437	Arabidopsis thaliana chromosome 1 sequence
AT4G23070	-2.176793069	0.000412437	Arabidopsis thaliana RHOMBOID-like protein 7 mRNA, complete cds
AT1G14750	-1.328796162	0.000412759	Arabidopsis thaliana cyclin-SDS mRNA, complete cds
AT4G27654	-3.721113585	0.000417636	Arabidopsis thaliana chromosome 4 sequence
AT5G20860	-2.029235881	0.000438469	Arabidopsis thaliana putative pectinesterase/pectinesterase inhibitor 54 mRNA, complete cds
AT1G52660	-1.820649259	0.000442961	Arabidopsis thaliana probable disease resistance protein mRNA, complete cds
AT5G24313	-1.463955746	0.000443272	Arabidopsis thaliana chromosome 5 sequence
AT4G01200	-1.498721164	0.000489519	Arabidopsis thaliana chromosome 4 sequence
AT3G03280	-1.280540994	0.000524975	Arabidopsis thaliana chromosome 3, complete sequence
AT5G25750	-1.373190282	0.000525086	Arabidopsis thaliana uncharacterized protein mRNA, complete cds
AT1G24485	-1.161686177	0.000533147	Arabidopsis thaliana uncharacterized protein mRNA, complete cds

AT1G03506	-4.498721164	0.000557045	Arabidopsis thaliana chromosome 1 sequence
AT3G10600	-1.221880959	0.000574365	Arabidopsis thaliana cationic amino acid transporter 7 mRNA, complete cds
AT5G13150	-1.083683665	0.000575329	Arabidopsis thaliana chromosome 5 sequence
AT1G02610	-1.051262187	0.000586378	Arabidopsis thaliana RING/FYVE/PHD zinc finger-containing protein mRNA, complete cds
AT5G26270	-1.39918549	0.00058925	Arabidopsis thaliana uncharacterized protein mRNA, complete cds
AT5G42785	-1.39918549	0.00058925	Arabidopsis thaliana uncharacterized protein mRNA, complete cds
AT5G52270	-2.306076086	0.000620345	Arabidopsis thaliana SNARE-like superfamily protein mRNA, complete cds
AT1G52830	-1.636224688	0.00062888	Arabidopsis thaliana indole-3-acetic acid 6 mRNA, complete cds
AT5G38540	-1.089608499	0.000666694	Arabidopsis thaliana Mannose-binding lectin superfamily protein mRNA, complete cds
AT2G01275	-1.318148918	0.000677557	Arabidopsis thaliana RING/FYVE/PHD zinc finger-containing protein mRNA, complete cds
AT5G38770	-2.913758663	0.000680257	Arabidopsis thaliana chromosome 5 sequence
AT3G21805	-1.972652352	0.000713354	Arabidopsis thaliana chromosome 3, complete sequence
AT3G03830	-1.861291243	0.000716266	Arabidopsis thaliana chromosome 3, complete sequence
AT1G32928	-1.25079365	0.000752479	Arabidopsis thaliana chromosome 1 sequence
AT5G15890	-1.340023418	0.000766894	Arabidopsis thaliana protein trichome birefringence-like 21 mRNA, complete cds
AT5G59845	-1.267395618	0.000859331	Arabidopsis thaliana gibberellin-regulated protein mRNA, complete cds
AT1G51870	-1.145084209	0.000860561	Arabidopsis thaliana putative serine/threonine protein kinase mRNA, complete cds
AT5G43890	-1.541789886	0.00088761	Arabidopsis thaliana chromosome 5 sequence
AT1G10400	-1.033057592	0.000929428	Arabidopsis thaliana UDP-glycosyltransferase 90A2 mRNA, complete cds
AT4G26580	-1.003956472	0.000932416	Arabidopsis thaliana RING/U-box domain-containing protein mRNA, complete cds
AT5G46370	-1.154766763	0.000993466	Arabidopsis thaliana two-pore potassium channel 2 mRNA, complete cds
AT1G63590	-4.373190282	0.001026381	Arabidopsis thaliana chromosome 1 sequence
AT1G33920	-4.373190282	0.001026381	Arabidopsis thaliana phloem protein 2-A4 mRNA, complete cds
AT2G34440	-2.235686758	0.001045955	Arabidopsis thaliana chromosome 2, complete sequence
AT3G22820	-2.235686758	0.001045955	Arabidopsis thaliana allergen-related protein mRNA, complete cds

AT1G55410	-2.235686758	0.001045955	Arabidopsis thaliana chromosome 1 sequence
AT5G67620	-1.220420001	0.001073121	Arabidopsis thaliana mRNA for hypothetical protein, complete cds, clone: RAFL14-69-L22
AT5G04330	-1.037141079	0.001080635	Arabidopsis thaliana cytochrome P450 84A4 mRNA, complete cds
AT1G01130	-1.037141079	0.001080635	Arabidopsis thaliana uncharacterized protein mRNA, complete cds
AT3G08490	-1.422772311	0.001088951	Arabidopsis thaliana uncharacterized protein mRNA, complete cds
AT1G26360	-1.721113585	0.001097665	Arabidopsis thaliana methyl esterase 13 mRNA, complete cds
AT4G21340	-1.306076086	0.001114092	Arabidopsis thaliana transcription factor bHLH103 mRNA, complete cds
AT1G33910	-2.051262187	0.001127179	Arabidopsis thaliana P-loop containing nucleoside triphosphate hydrolases superfamily protein mRNA, complete cds
AT1G48670	-2.051262187	0.001127179	Arabidopsis thaliana auxin-responsive GH3 family protein mRNA, complete cds
AT4G02780	-2.051262187	0.001127179	Arabidopsis thaliana Ent-copalyl diphosphate synthase mRNA, complete cds
AT2G15130	-2.051262187	0.001127179	Arabidopsis thaliana chromosome 2, complete sequence
AT3G15700	-2.051262187	0.001127179	Arabidopsis thaliana P-loop containing nucleoside triphosphate hydrolases superfamily protein mRNA, complete cds
AT1G73580	-1.806843459	0.001140071	Arabidopsis thaliana C2 domain-containing protein mRNA, complete cds
AT5G11290	-1.806843459	0.001140071	Arabidopsis thaliana chromosome 5 sequence
AT5G59340	-1.806843459	0.001140071	Arabidopsis thaliana WUSCHEL-related homeobox 2 mRNA, complete cds
AT4G00070	-1.913758663	0.001153988	Arabidopsis thaliana RING/U-box superfamily protein mRNA, complete cds
AT1G74830	-1.913758663	0.001153988	Arabidopsis thaliana uncharacterized protein mRNA, complete cds
AT2G35890	-2.820649259	0.001211275	Arabidopsis thaliana calcium-dependent protein kinase 25 mRNA, complete cds
AT2G02690	-2.820649259	0.001211275	Arabidopsis thaliana Cysteine/Histidine-rich C1 domain family protein mRNA, complete cds
AT1G66650	-2.820649259	0.001211275	Arabidopsis thaliana E3 ubiquitin-protein ligase SINA-like 4 mRNA, complete cds
AT4G28620	-2.820649259	0.001211275	Arabidopsis thaliana ABC transporter B family member 24 mRNA, complete cds
AT5G50175	-2.820649259	0.001211275	Arabidopsis thaliana chromosome 5 sequence
AT1G05990	-2.820649259	0.001211275	Arabidopsis thaliana chromosome 1 sequence

AT4G15248	-1.458079179	0.001212406	Arabidopsis thaliana chromosome 4 sequence
AT1G64920	-1.235686758	0.001230354	Arabidopsis thaliana chromosome 1 sequence
AT5G45440	-1.04151421	0.001256545	Arabidopsis thaliana chromosome 5 sequence
AT1G20470	-1.04151421	0.001256545	Arabidopsis thaliana chromosome 1 sequence
AT4G25470	-1.498721164	0.001341054	Arabidopsis thaliana chromosome 4 sequence
AT5G19800	-1.498721164	0.001341054	Arabidopsis thaliana chromosome 5 sequence
AT5G45210	-1.126752387	0.001388933	Arabidopsis thaliana TIR-NBS-LRR class disease resistance protein mRNA, complete cds
AT1G75620	-1.252560577	0.001408495	Arabidopsis thaliana chromosome 1 sequence
AT3G55720	-1.252560577	0.001408495	Arabidopsis thaliana uncharacterized protein mRNA, complete cds
AT2G43470	-3.498721164	0.001411143	Arabidopsis thaliana uncharacterized protein mRNA, complete cds
AT5G18050	-1.354331255	0.001429252	Arabidopsis thaliana chromosome 5 sequence
AT3G47740	-1.668646165	0.0017123	Arabidopsis thaliana ABC transporter A family member 3 mRNA, complete cds
AT3G16900	-2.161686177	0.001754107	Arabidopsis thaliana uncharacterized protein mRNA, complete cds
AT3G20850	-1.750259931	0.001803439	Arabidopsis thaliana chromosome 3, complete sequence
AT3G28857	-1.292270286	0.001835334	Arabidopsis thaliana protein PACLOBUTRAZOL RESISTANCE 5 mRNA, complete cds
AT1G58037	-1.984147991	0.001847473	Arabidopsis thaliana chromosome 1 sequence
AT3G27490	-1.852358119	0.001855416	Arabidopsis thaliana chromosome 3, complete sequence
AT5G51500	-1.852358119	0.001855416	Arabidopsis thaliana putative pectinesterase/pectinesterase inhibitor 60 mRNA, complete cds
AT5G32621	-4.235686758	0.001902229	Arabidopsis thaliana chromosome 5 sequence
AT5G45105	-4.235686758	0.001902229	Arabidopsis thaliana zinc transporter 8 precursor mRNA, complete cds
AT3G53235	-1.056716617	0.001976294	Arabidopsis thaliana uncharacterized protein mRNA, complete cds
AT2G44910	-1.020673867	0.001995489	Arabidopsis thaliana homeobox-leucine zipper protein ATHB-4 mRNA, complete cds
AT1G73160	-1.315857107	0.002087404	Arabidopsis thaliana chromosome 1 sequence
AT5G58784	-1.498721164	0.002229695	Arabidopsis thaliana dehydrodolichyl diphosphate synthase 5 mRNA, complete cds
AT3G07490	-1.498721164	0.002229695	Arabidopsis thaliana chromosome 3, complete sequence
AT3G24750	-1.062622049	0.002298522	Arabidopsis thaliana uncharacterized protein mRNA, complete cds
AT3G03820	-1.062622049	0.002298522	Arabidopsis thaliana chromosome 3, complete

			sequence
AT5G14130	-1.235686758	0.002311984	Arabidopsis thaliana peroxidase 55 mRNA, complete cds
AT5G14180	-1.235686758	0.002311984	Arabidopsis thaliana Myzus persicae-induced lipase 1 mRNA, complete cds
AT2G14960	-1.235686758	0.002311984	Arabidopsis thaliana putative indole-3-acetic acid-amido synthetase GH3.1 mRNA, complete cds
AT5G23370	-1.342601962	0.002366645	Arabidopsis thaliana chromosome 5 sequence
AT1G76420	-1.342601962	0.002366645	Arabidopsis thaliana protein CUP-SHAPED COTYLEDON 3 mRNA, complete cds
AT4G34800	-1.342601962	0.002366645	Arabidopsis thaliana chromosome 4 sequence
AT4G09110	-1.342601962	0.002366645	Arabidopsis thaliana chromosome 4 sequence
AT4G20362	-1.551188584	0.00244668	Arabidopsis thaliana Full-length cDNA Complete sequence from clone GSLTPGH40ZC03 of Hormone Treated Callus of strain col-0 of Arabidopsis thaliana (thale cress)
AT4G10150	-1.551188584	0.00244668	Arabidopsis thaliana RING-H2 finger protein ATL7 mRNA, complete cds
AT3G21660	-3.373190282	0.002598794	Arabidopsis thaliana UBX domain-containing protein mRNA, complete cds
AT1G19371	-3.373190282	0.002598794	Arabidopsis thaliana chromosome 1 sequence
AT1G55580	-1.115392524	0.002599621	Arabidopsis thaliana chromosome 1 sequence
AT3G60090	-1.254795581	0.00264947	Arabidopsis thaliana chromosome 3, complete sequence
AT4G34970	-1.254795581	0.00264947	Arabidopsis thaliana actin depolymerizing factor 9 mRNA, complete cds
AT2G35612	-2.328796162	0.002655515	Arabidopsis thaliana chromosome 2, complete sequence
AT4G11390	-2.328796162	0.002655515	Arabidopsis thaliana cysteine/histidine-rich C1 domain-containing protein mRNA, complete cds
AT2G34350	-1.373190282	0.002672904	Arabidopsis thaliana nodulin-like domain-containing protein mRNA, complete cds
AT5G01060	-1.691366242	0.002833982	Arabidopsis thaliana Protein kinase protein with tetratricopeptide repeat domain mRNA, complete cds
AT3G46240	-1.183847827	0.002863446	Arabidopsis thaliana uncharacterized protein mRNA, complete cds
AT2G37780	-1.183847827	0.002863446	Arabidopsis thaliana chromosome 2, complete sequence
AT1G13500	-2.083683665	0.002924295	Arabidopsis thaliana uncharacterized protein mRNA, complete cds
AT3G19320	-2.083683665	0.002924295	Arabidopsis thaliana leucine-rich repeat-containing protein mRNA, complete cds

AT3G60280	-2.083683665	0.002924295	Arabidopsis thaliana uclacyanin 3 mRNA, complete cds
AT4G34810	-1.788227781	0.002963608	Arabidopsis thaliana chromosome 4 sequence
AT3G45080	-1.788227781	0.002963608	Arabidopsis thaliana chromosome 3, complete sequence
AT3G55515	-1.788227781	0.002963608	Arabidopsis thaliana chromosome 3, complete sequence
AT4G28840	-1.913758663	0.003008732	Arabidopsis thaliana TCP interactor containing EAR motif protein1 mRNA, complete cds
AT4G14305	-1.076030092	0.003109206	Arabidopsis thaliana Mpv17/PMP22 domain-containing protein mRNA, complete cds
AT4G02180	-1.199160882	0.003301236	Arabidopsis thaliana DC1 domain-containing protein mRNA, complete cds
AT1G51260	-1.449811563	0.00335655	Arabidopsis thaliana 1-acyl-sn-glycerol-3-phosphate acyltransferase 3 mRNA, complete cds
AT3G52530	-1.300781786	0.003456365	Arabidopsis thaliana chromosome 3, complete sequence
AT3G11150	-1.300781786	0.003456365	Arabidopsis thaliana 2-oxoglutarate (2OG) and Fe(II)-dependent oxygenase superfamily protein mRNA, complete cds
AT1G21340	-4.083683665	0.003547399	Arabidopsis thaliana chromosome 1 sequence
AT3G51560	-4.083683665	0.003547399	Arabidopsis thaliana TIR-NBS-LRR class disease resistance protein mRNA, complete cds
AT5G17590	-4.083683665	0.003547399	Arabidopsis thaliana chromosome 5 sequence
AT1G48070	-4.083683665	0.003547399	Arabidopsis thaliana TRX domain-containing protein mRNA, complete cds
AT1G34500	-4.083683665	0.003547399	Arabidopsis thaliana chromosome 1 sequence
AT2G45650	-4.083683665	0.003547399	Arabidopsis thaliana agamous-like MADS-box protein AGL6 mRNA, complete cds
AT1G65570	-1.083683665	0.003615988	Arabidopsis thaliana polygalacturonase family protein mRNA, complete cds
AT2G42820	-1.083683665	0.003615988	Arabidopsis thaliana HVA22-like protein F mRNA, complete cds
AT1G35250	-1.001221504	0.003693192	Arabidopsis thaliana thioesterase family protein mRNA, complete cds
AT5G35525	-1.498721164	0.003721138	Arabidopsis thaliana PLAC8 family protein mRNA, complete cds
AT2G34910	-1.216321433	0.00380119	Arabidopsis thaliana chromosome 2, complete sequence
AT1G69990	-2.614198381	0.003804419	Arabidopsis thaliana chromosome 1 sequence
AT3G54450	-2.614198381	0.003804419	Arabidopsis thaliana probable peptide/nitrate transporter mRNA, complete cds
AT5G45240	-1.148223917	0.004046734	Arabidopsis thaliana TIR-NBS-LRR class disease

			resistance protein mRNA, complete cds
AT2G37820	-1.557614853	0.004083885	Arabidopsis thaliana chromosome 2, complete sequence
AT3G53010	-1.004906551	0.004311841	Arabidopsis thaliana uncharacterized protein mRNA, complete cds
AT4G22110	-1.004906551	0.004311841	Arabidopsis thaliana GroES-like zinc-binding dehydrogenase family protein mRNA, complete cds
AT2G26390	-1.004906551	0.004311841	Arabidopsis thaliana serpin-Z3 mRNA, complete cds
AT1G04778	-1.629965697	0.004421968	Arabidopsis thaliana uncharacterized protein mRNA, complete cds
AT4G05170	-1.36121764	0.004451977	Arabidopsis thaliana transcription factor bHLH114 mRNA, complete cds
AT1G79250	-2.235686758	0.004529575	Arabidopsis thaliana AGC kinase 1.7 mRNA, complete cds
AT1G34520	-2.235686758	0.004529575	Arabidopsis thaliana MBOAT (membrane bound O-acyl transferase) family protein mRNA, complete cds
AT3G45638	-2.235686758	0.004529575	Arabidopsis thaliana clone asmbl_10177 unknown mRNA sequence
AT2G17070	-2.235686758	0.004529575	Arabidopsis thaliana chromosome 2, complete sequence
AT5G39630	-2.235686758	0.004529575	Arabidopsis thaliana vesicle transport v-SNARE family protein mRNA, complete cds
AT1G19968	-2.235686758	0.004529575	Arabidopsis thaliana chromosome 1 sequence
AT3G13403	-2.235686758	0.004529575	Arabidopsis thaliana defensin-like protein 302 mRNA, complete cds
AT3G01513	-1.161686177	0.004684636	Arabidopsis thaliana chromosome 3, complete sequence
AT1G10000	-1.721113585	0.004700164	Arabidopsis thaliana chromosome 1 sequence
AT3G14185	-1.721113585	0.004700164	Arabidopsis thaliana Full-length cDNA Complete sequence from clone GSLTSIL56ZC04 of Silique of strain col-0 of Arabidopsis thaliana (thale cress)
AT1G09380	-3.235686758	0.004790244	Arabidopsis thaliana nodulin MtN21-like transporter family protein mRNA, complete cds
AT1G51960	-3.235686758	0.004790244	Arabidopsis thaliana IQ-domain 27 protein mRNA, complete cds
AT2G23400	-3.235686758	0.004790244	Arabidopsis thaliana undecaprenyl pyrophosphate synthetase family protein mRNA, complete cds
AT2G22750	-3.235686758	0.004790244	Arabidopsis thaliana transcription factor bHLH18 mRNA, complete cds
AT1G07795	-3.235686758	0.004790244	Arabidopsis thaliana chromosome 1 sequence
AT2G06200	-1.101385666	0.004889249	Arabidopsis thaliana growth-regulating factor 6

			mRNA, complete cds
AT1G47578	-1.101385666	0.004889249	Arabidopsis thaliana putative lipoyltransferase-like protein mRNA, complete cds
AT3G13435	-1.101385666	0.004889249	Arabidopsis thaliana uncharacterized protein mRNA, complete cds
AT3G15358	-1.051262187	0.005000807	Arabidopsis thaliana uncharacterized protein mRNA, complete cds
AT5G37210	-1.051262187	0.005000807	Arabidopsis thaliana cysteine/histidine-rich C1 domain-containing protein mRNA, complete cds
AT1G73410	-1.257713064	0.005014462	Arabidopsis thaliana myb domain protein 54 mRNA, complete cds
AT1G75700	-1.008915896	0.005035677	Arabidopsis thaliana HVA22-like protein G mRNA, complete cds
AT1G61480	-1.176793069	0.005418603	Arabidopsis thaliana G-type lectin S-receptor-like serine/threonine-protein kinase mRNA, complete cds
AT5G46845	-1.44427338	0.005618674	Arabidopsis thaliana chromosome 5 sequence
AT3G46800	-1.111698041	0.005683629	Arabidopsis thaliana chromosome 3, complete sequence
AT4G25707	-1.111698041	0.005683629	Arabidopsis thaliana chromosome 4 sequence
AT5G47530	-1.282992473	0.005739937	Arabidopsis thaliana putative auxin-responsive protein mRNA, complete cds
AT5G23903	-1.282992473	0.005739937	Arabidopsis thaliana uncharacterized protein mRNA, complete cds
AT3G03650	-1.058148573	0.005831344	Arabidopsis thaliana Exostosin family protein mRNA, complete cds
AT1G52827	-1.013294337	0.005882897	Arabidopsis thaliana cadmium tolerance 1 mRNA, complete cds
AT1G31835	-1.013294337	0.005882897	Arabidopsis thaliana chromosome 1 sequence
AT4G03480	-1.498721164	0.006238449	Arabidopsis thaliana Ankyrin repeat family protein mRNA, complete cds
AT1G24420	-1.498721164	0.006238449	Arabidopsis thaliana chromosome 1 sequence
AT5G26790	-1.31230804	0.006550197	Arabidopsis thaliana chromosome 5 sequence
AT1G07460	-3.913758663	0.006659328	Arabidopsis thaliana chromosome 1 sequence
AT5G06905	-3.913758663	0.006659328	Arabidopsis thaliana cytochrome P450, family 712, subfamily A, polypeptide 2 mRNA, complete cds
AT2G31930	-2.498721164	0.006701927	Arabidopsis thaliana chromosome 2, complete sequence
AT2G02640	-2.498721164	0.006701927	Arabidopsis thaliana chromosome 2, complete sequence
AT1G35730	-2.498721164	0.006701927	Arabidopsis thaliana protein pumilio 9 mRNA, complete cds
AT1G67460	-1.065761757	0.006800543	Arabidopsis thaliana Minichromosome

			maintenance (MCM2/3/5) family protein mRNA, complete cds
AT2G20700	-1.56583536	0.006847536	Arabidopsis thaliana LORELEI-like glucosylphosphatidylinositol-anchored protein 2 mRNA, complete cds
AT1G32583	-1.56583536	0.006847536	Arabidopsis thaliana uncharacterized protein mRNA, complete cds
AT5G23030	-1.56583536	0.006847536	Arabidopsis thaliana tetraspanin12 mRNA, complete cds
AT3G60580	-1.018095323	0.006874906	Arabidopsis thaliana chromosome 3, complete sequence
AT1G78520	-1.213318945	0.007224612	Arabidopsis thaliana carbohydrate-binding X8 domain-containing protein mRNA, complete cds
AT4G15396	-1.213318945	0.007224612	Arabidopsis thaliana cytochrome P450, family 702, subfamily A, polypeptide 6 mRNA, complete cds
AT2G02620	-1.650724257	0.007397207	Arabidopsis thaliana chromosome 2, complete sequence
AT3G11390	-1.34671807	0.007445295	Arabidopsis thaliana chromosome 3, complete sequence
AT1G69150	-1.34671807	0.007445295	Arabidopsis thaliana chromosome 1 sequence
AT4G12450	-1.136151085	0.007672669	Arabidopsis thaliana chromosome 4 sequence
AT3G25060	-2.136151085	0.007677135	Arabidopsis thaliana chromosome 3, complete sequence
AT2G26695	-2.136151085	0.007677135	Arabidopsis thaliana Ran BP2/NZF zinc finger-like protein mRNA, complete cds
AT3G63360	-1.76175557	0.007809487	Arabidopsis thaliana defensin-like protein mRNA, complete cds
AT2G15350	-1.76175557	0.007809487	Arabidopsis thaliana chromosome 2, complete sequence
AT5G51930	-1.76175557	0.007809487	Arabidopsis thaliana Glucose-methanol-choline (GMC) oxidoreductase family protein mRNA, complete cds
AT3G14510	-1.76175557	0.007809487	Arabidopsis thaliana putative geranylgeranyl pyrophosphate synthase 8 mRNA, complete cds
AT5G38790	-1.76175557	0.007809487	Arabidopsis thaliana chromosome 5 sequence
AT5G59270	-1.074223335	0.007931415	Arabidopsis thaliana concanavalin A-like lectin protein kinase family protein mRNA, complete cds
AT5G18910	-1.913758663	0.007963202	Arabidopsis thaliana protein kinase family protein mRNA, complete cds
AT2G04680	-1.913758663	0.007963202	Arabidopsis thaliana chromosome 2, complete sequence
AT3G47170	-1.913758663	0.007963202	Arabidopsis thaliana HXXXD-type acyl-transferase family protein mRNA, complete cds

AT1G01590	-1.913758663	0.007963202	Arabidopsis thaliana ferric reduction oxidase 1 mRNA, complete cds
AT1G52100	-1.235686758	0.008322202	Arabidopsis thaliana jacalin-like lectin domain-containing protein mRNA, complete cds
AT5G25020	-1.235686758	0.008322202	Arabidopsis thaliana uncharacterized protein mRNA, complete cds
AT4G25400	-1.387689852	0.00841898	Arabidopsis thaliana transcription factor bHLH118 mRNA, complete cds
AT2G44390	-1.387689852	0.00841898	Arabidopsis thaliana cysteine/histidine-rich C1 domain-containing protein mRNA, complete cds
AT4G21902	-3.083683665	0.008834015	Arabidopsis thaliana chromosome 4 sequence
AT1G31260	-3.083683665	0.008834015	Arabidopsis thaliana putative zinc transporter 10 mRNA, complete cds
AT2G18010	-3.083683665	0.008834015	Arabidopsis thaliana chromosome 2, complete sequence
AT4G38495	-3.083683665	0.008834015	Arabidopsis thaliana uncharacterized protein mRNA, complete cds
AT5G58280	-3.083683665	0.008834015	Arabidopsis thaliana AP2/B3-like transcriptional factor family protein mRNA, complete cds
AT5G14470	-3.083683665	0.008834015	Arabidopsis thaliana GHMP kinase family protein mRNA, complete cds
AT1G19610	-3.083683665	0.008834015	Arabidopsis thaliana defensin-like protein 19 mRNA, complete cds
AT4G34850	-3.083683665	0.008834015	Arabidopsis thaliana protein LESS ADHESIVE POLLEN 5 mRNA, complete cds
AT2G35290	-3.083683665	0.008834015	Arabidopsis thaliana chromosome 2, complete sequence
AT5G38096	-3.083683665	0.008834015	Arabidopsis thaliana chromosome 5 sequence
AT5G58910	-1.437320619	0.009454424	Arabidopsis thaliana laccase 16 mRNA, complete cds
AT5G54050	-1.261681967	0.009565127	Arabidopsis thaliana cysteine/histidine-rich C1 domain-containing protein mRNA, complete cds
AT2G18120	-1.167515255	0.010333537	Arabidopsis thaliana SHI-related sequence 4 protein mRNA, complete cds
AT1G05770	-1.094330909	0.010788846	Arabidopsis thaliana mannose-binding lectin-like protein mRNA, complete cds
AT1G09155	-1.292270286	0.010961448	Arabidopsis thaliana phloem protein 2-B15 mRNA, complete cds
AT5G63690	-1.035749188	0.010994443	Arabidopsis thaliana chromosome 5 sequence
AT4G15360	-1.035749188	0.010994443	Arabidopsis thaliana cytochrome P450, family 705, subfamily A, polypeptide 3 mRNA, complete cds
AT3G46410	-1.576723676	0.011545344	Arabidopsis thaliana protein kinase family protein mRNA, complete cds

AT3G47770	-1.576723676	0.011545344	Arabidopsis thaliana ABC transporter A family member 6 mRNA, complete cds
AT2G31083	-1.576723676	0.011545344	Arabidopsis thaliana chromosome 2, complete sequence
AT2G31082	-2.373190282	0.011746088	Arabidopsis thaliana chromosome 2, complete sequence
AT3G29772	-2.373190282	0.011746088	Arabidopsis thaliana chromosome 3, complete sequence
AT1G03445	-2.373190282	0.011746088	Arabidopsis thaliana serine/threonine-protein phosphatase BSU1 mRNA, complete cds
AT1G33280	-1.186777158	0.011975014	Arabidopsis thaliana protein BEARSKIN1 mRNA, complete cds
AT1G01453	-1.67929341	0.012429649	Arabidopsis thaliana chromosome 1 sequence
AT4G37770	-1.67929341	0.012429649	Arabidopsis thaliana 1-aminocyclopropane-1-carboxylate synthase 8 mRNA, complete cds
AT3G17225	-1.328796162	0.012512528	Arabidopsis thaliana chromosome 3, complete sequence
AT4G10780	-3.721113585	0.012590237	Arabidopsis thaliana chromosome 4 sequence
AT3G18460	-3.721113585	0.012590237	Arabidopsis thaliana PLAC8 family protein mRNA, complete cds
AT2G23440	-3.721113585	0.012590237	Arabidopsis thaliana chromosome 2, complete sequence
AT3G50710	-3.721113585	0.012590237	Arabidopsis thaliana putative FBD-associated F-box protein mRNA, complete cds
AT1G50720	-3.721113585	0.012590237	Arabidopsis thaliana chromosome 1 sequence
AT2G47040	-3.721113585	0.012590237	Arabidopsis thaliana pectinesterase 5 mRNA, complete cds
AT3G47342	-1.04304168	0.012865818	Arabidopsis thaliana chromosome 3, complete sequence
AT2G04480	-2.029235881	0.012917738	Arabidopsis thaliana uncharacterized protein mRNA, complete cds
AT1G43590	-2.029235881	0.012917738	Arabidopsis thaliana chromosome 1 sequence
AT1G70450	-2.029235881	0.012917738	Arabidopsis thaliana protein kinase superfamily protein mRNA, complete cds
AT4G37220	-2.029235881	0.012917738	Arabidopsis thaliana cold acclimation protein WCOR413 mRNA, complete cds
AT1G73780	-2.029235881	0.012917738	Arabidopsis thaliana chromosome 1 sequence
AT2G16960	-2.029235881	0.012917738	Arabidopsis thaliana ARM repeat superfamily protein mRNA, complete cds
AT5G16330	-2.029235881	0.012917738	Arabidopsis thaliana NC domain-containing protein-like protein mRNA, complete cds
AT5G03860	-1.820649259	0.012987394	Arabidopsis thaliana malate synthase mRNA, complete cds

AT5G54790	-1.820649259	0.012987394	Arabidopsis thaliana uncharacterized protein mRNA, complete cds
AT4G21260	-1.820649259	0.012987394	Arabidopsis thaliana sulfite exporter TauE/SafE family protein mRNA, complete cds
AT1G29830	-1.209214547	0.01385754	Arabidopsis thaliana Magnesium transporter CorA-like family protein mRNA, complete cds
AT3G11460	-1.209214547	0.01385754	Arabidopsis thaliana chromosome 3, complete sequence
AT1G08310	-1.120209541	0.014668237	Arabidopsis thaliana alpha/beta-Hydrolases superfamily protein mRNA, complete cds
AT3G10470	-1.051262187	0.015060667	Arabidopsis thaliana chromosome 3, complete sequence
AT4G13440	-1.051262187	0.015060667	Arabidopsis thaliana chromosome 4 sequence
AT5G36260	-1.235686758	0.016004986	Arabidopsis thaliana aspartyl protease family protein mRNA, complete cds
AT2G18480	-1.235686758	0.016004986	Arabidopsis thaliana putative polyol transporter 3 mRNA, complete cds
AT1G75166	-1.235686758	0.016004986	Arabidopsis thaliana chromosome 1 sequence
AT3G51680	-1.428331836	0.01601285	Arabidopsis thaliana short-chain dehydrogenase reductase 2a mRNA, complete cds
AT4G31250	-2.913758663	0.016290784	Arabidopsis thaliana putative LRR receptor-like serine/threonine-protein kinase mRNA, complete cds
AT2G27440	-2.913758663	0.016290784	Arabidopsis lyrata subsp. lyrata hypothetical protein, mRNA
AT1G64480	-2.913758663	0.016290784	Arabidopsis thaliana calcineurin B-like protein 8 mRNA, complete cds
AT3G56500	-2.913758663	0.016290784	Arabidopsis thaliana chromosome 3, complete sequence
AT3G20610	-2.913758663	0.016290784	Arabidopsis thaliana chromosome 3, complete sequence
AT1G25240	-2.913758663	0.016290784	Arabidopsis thaliana chromosome 1 sequence
AT5G60230	-1.060600052	0.017635367	Arabidopsis thaliana chromosome 5 sequence
AT5G05900	-1.060600052	0.017635367	Arabidopsis thaliana UDP-glycosyltransferase 76C3 mRNA, complete cds
AT3G17580	-1.001221504	0.017764641	Arabidopsis thaliana uncharacterized protein mRNA, complete cds
AT3G61300	-1.001221504	0.017764641	Arabidopsis thaliana chromosome 3, complete sequence
AT4G39361	-1.001221504	0.017764641	Arabidopsis thaliana chromosome 4 sequence
AT2G37740	-1.498721164	0.017857487	Arabidopsis thaliana chromosome 2, complete sequence
AT1G57570	-1.498721164	0.017857487	Arabidopsis thaliana jacalin-like plant lectin domain-containing protein mRNA, complete cds

AT5G23780	-1.498721164	0.017857487	Arabidopsis thaliana uncharacterized protein mRNA, complete cds
AT1G61130	-1.498721164	0.017857487	Arabidopsis thaliana serine carboxypeptidase-like 32 mRNA, complete cds
AT1G07175	-1.498721164	0.017857487	Arabidopsis thaliana uncharacterized protein mRNA, complete cds
AT2G04500	-1.498721164	0.017857487	Arabidopsis thaliana chromosome 2, complete sequence
AT4G24204	-1.267395618	0.018435942	Arabidopsis thaliana RING-finger domain-containing protein mRNA, complete cds
AT2G45040	-1.267395618	0.018435942	Arabidopsis thaliana chromosome 2, complete sequence
AT4G15258	-1.591830568	0.019602459	Arabidopsis thaliana chromosome 4 sequence
AT1G09860	-1.591830568	0.019602459	Arabidopsis thaliana chromosome 1 sequence
AT1G65484	-1.154766763	0.019909115	Arabidopsis thaliana uncharacterized protein mRNA, complete cds
AT1G52110	-2.235686758	0.020460192	Arabidopsis thaliana jacalin-like lectin domain-containing protein mRNA, complete cds
AT1G23350	-2.235686758	0.020460192	Arabidopsis thaliana chromosome 1 sequence
AT5G46871	-2.235686758	0.020460192	Arabidopsis thaliana defensin-like protein 308 mRNA, complete cds
AT1G17285	-1.07129994	0.020655801	Arabidopsis thaliana uncharacterized protein mRNA, complete cds
AT5G57240	-1.07129994	0.020655801	Arabidopsis thaliana OSBP(oxysterol binding protein)-related protein 4C mRNA, complete cds
AT1G11920	-1.07129994	0.020655801	Arabidopsis thaliana putative pectate lyase mRNA, complete cds
AT2G35950	-1.721113585	0.020990776	Arabidopsis thaliana protein EMBRYO SAC DEVELOPMENT ARREST 12 mRNA, complete cds
AT5G62330	-1.721113585	0.020990776	Arabidopsis thaliana uncharacterized protein mRNA, complete cds
AT5G51470	-1.306076086	0.021156913	Arabidopsis thaliana auxin-responsive GH3 family protein mRNA, complete cds
AT5G56795	-1.306076086	0.021156913	Arabidopsis thaliana metallothionein 1B mRNA, complete cds
AT3G22250	-1.913758663	0.021555675	Arabidopsis thaliana UDP-glycosyltransferase 82A1 mRNA, complete cds
ATCG01100	-1.913758663	0.021555675	Arabidopsis lyrata subsp. lyrata NADH-plastoquinone oxidoreductase subunit 1, mRNA
AT3G58060	-1.913758663	0.021555675	Arabidopsis thaliana putative metal tolerance protein C3 mRNA, complete cds
AT3G48630	-1.913758663	0.021555675	Arabidopsis thaliana uncharacterized protein mRNA, complete cds

AT3G21050	-1.176793069	0.023164884	Arabidopsis thaliana chromosome 3, complete sequence
AT4G24080	-1.176793069	0.023164884	Arabidopsis thaliana aldolase like protein mRNA, complete cds
AT1G57943	-3.498721164	0.023986793	Arabidopsis thaliana purine permease 17 mRNA, complete cds
AT5G01080	-3.498721164	0.023986793	Arabidopsis thaliana chromosome 5 sequence
AT3G09340	-3.498721164	0.023986793	Arabidopsis thaliana transmembrane amino acid transporter-like protein mRNA, complete cds
AT4G15075	-3.498721164	0.023986793	Arabidopsis thaliana FBD-like domain family protein mRNA, complete cds
ATCG01060	-3.498721164	0.023986793	Arabidopsis thaliana chloroplast DNA, complete genome, ecotype: Columbia
AT5G27200	-3.498721164	0.023986793	Arabidopsis thaliana acyl carrier protein 5 mRNA, complete cds
AT3G62320	-3.498721164	0.023986793	Arabidopsis thaliana putative nucleic acid binding protein mRNA, complete cds
AT3G17760	-3.498721164	0.023986793	Arabidopsis thaliana glutamate decarboxylase 5 mRNA, complete cds
AT1G80470	-3.498721164	0.023986793	Arabidopsis thaliana F-box/FBD/LRR-repeat protein mRNA, complete cds
AT5G48980	-3.498721164	0.023986793	Arabidopsis thaliana chromosome 5 sequence
AT4G01230	-3.498721164	0.023986793	Arabidopsis thaliana reticulon-like protein B7 mRNA, complete cds
AT1G74110	-3.498721164	0.023986793	Arabidopsis thaliana cytochrome P450, family 78, subfamily A, polypeptide 10 mRNA, complete cds
AT5G48575	-3.498721164	0.023986793	Arabidopsis thaliana uncharacterized protein mRNA, complete cds
AT1G03700	-1.354331255	0.024149994	Arabidopsis thaliana uncharacterized protein mRNA, complete cds
AT1G10680	-1.354331255	0.024149994	Arabidopsis thaliana ABC transporter B family member 10 mRNA, complete cds
AT2G43310	-1.083683665	0.024198591	Arabidopsis thaliana chromosome 2, complete sequence
AT3G21310	-1.083683665	0.024198591	Arabidopsis thaliana core-2/I-branching beta-1,6-N-acetylglucosaminyltransferase family protein mRNA, complete cds
AT3G49630	-1.013294337	0.024523624	Arabidopsis thaliana 2-oxoglutarate (2OG) and Fe(II)-dependent oxygenase superfamily protein mRNA, complete cds
AT2G46860	-1.013294337	0.024523624	Arabidopsis thaliana pyrophosphorylase 3 mRNA, complete cds
AT1G52530	-1.013294337	0.024523624	Arabidopsis thaliana Hus1 domain-containing protein mRNA, complete cds

AT4G14450	-1.20326528	0.026913837	Arabidopsis thaliana chromosome 4 sequence
AT4G22610	-1.20326528	0.026913837	Arabidopsis thaliana chromosome 4 sequence
AT3G46760	-1.416259004	0.027350294	Arabidopsis thaliana chromosome 3, complete sequence
AT5G04390	-1.416259004	0.027350294	Arabidopsis thaliana chromosome 5 sequence
AT4G22370	-1.416259004	0.027350294	Arabidopsis thaliana uncharacterized protein mRNA, complete cds
AT5G60310	-1.416259004	0.027350294	Arabidopsis thaliana putative L-type lectin-domain containing receptor kinase I.10 mRNA, complete cds
AT1G36675	-1.416259004	0.027350294	Arabidopsis thaliana glycine-rich protein mRNA, complete cds
AT2G46950	-1.416259004	0.027350294	Arabidopsis thaliana cytochrome P450, family 709, subfamily B, polypeptide 2 mRNA, complete cds
AT1G06923	-1.416259004	0.027350294	Arabidopsis thaliana uncharacterized protein mRNA, complete cds
AT5G35110	-1.098183234	0.028352138	Arabidopsis thaliana chromosome 5 sequence
AT5G11570	-1.098183234	0.028352138	Arabidopsis thaliana major facilitator protein mRNA, complete cds
AT1G78750	-1.020673867	0.028845109	Arabidopsis thaliana F-box/FBD/LRR-repeat protein mRNA, complete cds
AT1G03510	-1.020673867	0.028845109	Arabidopsis thaliana chromosome 1 sequence
AT2G01175	-2.721113585	0.030018609	Arabidopsis thaliana chromosome 2, complete sequence
AT4G02190	-2.721113585	0.030018609	Arabidopsis thaliana chromosome 4 sequence
AT5G53700	-2.721113585	0.030018609	Arabidopsis thaliana RNA-binding (RRM/RBD/RNP motifs) family protein mRNA, complete cds
AT4G18980	-2.721113585	0.030018609	Arabidopsis thaliana chromosome 4 sequence
AT1G27610	-2.721113585	0.030018609	Arabidopsis thaliana uncharacterized protein mRNA, complete cds
AT2G46494	-2.721113585	0.030018609	Arabidopsis thaliana RING/U-box family protein mRNA, complete cds
AT3G02590	-2.721113585	0.030018609	Arabidopsis thaliana putative Delta(7)-sterol-C5(6)-desaturase 2 mRNA, complete cds
AT5G39490	-2.721113585	0.030018609	Arabidopsis thaliana chromosome 5 sequence
ATCG01010	-2.721113585	0.030018609	Arabidopsis thaliana chloroplast DNA, complete genome, ecotype: Columbia
AT2G31540	-2.721113585	0.030018609	Arabidopsis thaliana GDSL esterase/lipase mRNA, complete cds
AT1G76135	-2.721113585	0.030018609	Arabidopsis thaliana chromosome 1 sequence
AT4G25190	-1.498721164	0.030603583	Arabidopsis thaliana uncharacterized protein mRNA, complete cds

AT4G26170	-1.498721164	0.030603583	Arabidopsis thaliana uncharacterized protein mRNA, complete cds
AT4G08400	-1.498721164	0.030603583	Arabidopsis thaliana chromosome 4 sequence
AT1G70660	-1.498721164	0.030603583	Arabidopsis thaliana ubiquitin-conjugating enzyme E2 variant 1B mRNA, complete cds
AT1G11570	-1.235686758	0.031201168	Arabidopsis thaliana chromosome 1 sequence
AT4G11655	-1.235686758	0.031201168	Arabidopsis thaliana uncharacterized protein mRNA, complete cds
AT1G05420	-1.235686758	0.031201168	Arabidopsis thaliana chromosome 1 sequence
AT3G05820	-1.235686758	0.031201168	Arabidopsis thaliana alkaline/neutral invertase H mRNA, complete cds
AT3G09330	-1.235686758	0.031201168	Arabidopsis thaliana transmembrane amino acid transporter-like protein mRNA, complete cds
AT3G05327	-1.115392524	0.033217084	Arabidopsis thaliana cyclin family protein mRNA, complete cds
AT2G43220	-1.115392524	0.033217084	Arabidopsis thaliana chromosome 2, complete sequence
AT2G05230	-1.614198381	0.033584017	Arabidopsis thaliana DNAJ heat shock N-terminal domain-containing protein mRNA, complete cds
AT5G57420	-1.614198381	0.033584017	Arabidopsis thaliana auxin-responsive protein IAA33 mRNA, complete cds
AT3G46668	-1.614198381	0.033584017	Arabidopsis thaliana mRNA, clone: RAFL21-22-L12
AT5G49320	-1.029235881	0.033954444	Arabidopsis thaliana uncharacterized protein mRNA, complete cds
AT1G53163	-1.029235881	0.033954444	Arabidopsis thaliana chromosome 1 sequence
AT1G31750	-2.083683665	0.03537351	Arabidopsis thaliana mRNA for hypothetical protein, complete cds, clone: RAFL16-18-G02
AT3G61090	-2.083683665	0.03537351	Arabidopsis thaliana putative endonuclease or glycosyl hydrolase mRNA, complete cds
AT5G07880	-2.083683665	0.03537351	Arabidopsis thaliana SNAP25-like protein SNAP29 mRNA, complete cds
AT1G78360	-2.083683665	0.03537351	Arabidopsis thaliana glutathione S-transferase TAU 21 mRNA, complete cds
AT5G60610	-2.083683665	0.03537351	Arabidopsis thaliana FBD-associated F-box protein mRNA, complete cds
AT3G54310	-2.083683665	0.03537351	Arabidopsis thaliana uncharacterized protein mRNA, complete cds
AT1G72590	-1.788227781	0.03562704	Arabidopsis thaliana 3-oxo-5-alpha-steroid 4-dehydrogenase family protein mRNA, complete cds
AT5G22150	-1.788227781	0.03562704	Arabidopsis thaliana uncharacterized protein mRNA, complete cds
AT5G44973	-1.788227781	0.03562704	Arabidopsis thaliana defensin-like protein 285 mRNA, complete cds

AT3G42658	-1.788227781	0.03562704	Arabidopsis thaliana chromosome 3, complete sequence
AT1G02405	-1.788227781	0.03562704	Arabidopsis thaliana chromosome 1 sequence
AT5G22660	-1.276328743	0.036051581	Arabidopsis thaliana F-box/FBD/LRR-repeat protein mRNA, complete cds
AT3G49510	-1.276328743	0.036051581	Arabidopsis thaliana F-box protein mRNA, complete cds
AT3G59130	-1.136151085	0.038905399	Arabidopsis thaliana Cysteine/Histidine-rich C1 domain family protein mRNA, complete cds
AT4G21780	-1.039289545	0.040000971	Arabidopsis thaliana chromosome 4 sequence
AT5G09670	-1.039289545	0.040000971	Arabidopsis thaliana chromosome 5 sequence
AT2G45800	-1.039289545	0.040000971	Arabidopsis thaliana protein PLIM2A mRNA, complete cds
AT4G12440	-1.039289545	0.040000971	Arabidopsis thaliana adenine phosphoribosyl transferase 4 mRNA, complete cds
AT5G62850	-1.161686177	0.0455364	Arabidopsis thaliana bidirectional sugar transporter SWEET5 mRNA, complete cds
AT5G65500	-1.161686177	0.0455364	Arabidopsis thaliana U-box domain-containing protein kinase family protein mRNA, complete cds
AT2G19140	-3.235686758	0.046084957	Arabidopsis thaliana chromosome 2, complete sequence
AT5G09500	-3.235686758	0.046084957	Arabidopsis thaliana 40S ribosomal protein S15-3 mRNA, complete cds
AT5G37620	-3.235686758	0.046084957	Arabidopsis thaliana chromosome 5 sequence
AT5G22490	-3.235686758	0.046084957	Arabidopsis thaliana O-acyltransferase (WSD1-like) family protein mRNA, complete cds
AT3G48346	-3.235686758	0.046084957	Arabidopsis thaliana chromosome 3, complete sequence
AT1G30740	-3.235686758	0.046084957	Arabidopsis thaliana chromosome 1 sequence
AT4G15970	-3.235686758	0.046084957	Arabidopsis thaliana Nucleotide-diphospho-sugar transferase family protein mRNA, complete cds
AT3G05780	-3.235686758	0.046084957	Arabidopsis thaliana lon protease 3 mRNA, complete cds
AT1G13400	-3.235686758	0.046084957	Arabidopsis thaliana zinc finger protein JAGGED-like/NUBBIN mRNA, complete cds
AT1G61270	-3.235686758	0.046084957	Arabidopsis thaliana transmembrane amino acid transporter family protein mRNA, complete cds
AT5G06520	-3.235686758	0.046084957	Arabidopsis thaliana SWAP/Surp domain-containing protein mRNA, complete cds
AT5G28888	-3.235686758	0.046084957	Arabidopsis thaliana chromosome 5 sequence
AT3G46350	-3.235686758	0.046084957	Arabidopsis thaliana Leucine-rich repeat protein kinase family protein mRNA, complete cds
ATCG00500	-3.235686758	0.046084957	Arabidopsis thaliana ribulose-1,5-bisphosphate carboxylase (RBCL) gene, partial cds; and

			carboxyltransferase beta subunit (ACCD) gene, complete cds; chloroplast
AT4G26930	-3.235686758	0.046084957	Arabidopsis thaliana myb domain protein 97 mRNA, complete cds
AT2G11891	-3.235686758	0.046084957	Arabidopsis thaliana chromosome 2, complete sequence
AT5G51105	-3.235686758	0.046084957	Arabidopsis thaliana chromosome 5 sequence
AT1G62760	-1.051262187	0.047162849	Arabidopsis thaliana chromosome 1 sequence
AT1G01810	-1.39918549	0.047247844	Arabidopsis thaliana chromosome 1 sequence
AT4G25490	-1.39918549	0.047247844	Arabidopsis thaliana chromosome 4 sequence
AT5G17165	-1.39918549	0.047247844	Arabidopsis thaliana uncharacterized protein mRNA, complete cds
ATCG00100	-1.39918549	0.047247844	Liverwort chloroplast Gly-tRNA-tcc
AT5G55770	-1.498721164	0.05312286	Arabidopsis thaliana chromosome 5 sequence
AT3G03290	-1.498721164	0.05312286	Arabidopsis thaliana Adenine nucleotide alpha hydrolases-like protein mRNA, complete cds
AT3G08885	-1.498721164	0.05312286	Arabidopsis thaliana chromosome 3, complete sequence
AT3G20557	-1.498721164	0.05312286	Arabidopsis thaliana uncharacterized protein mRNA, complete cds
AT3G29252	-1.498721164	0.05312286	Arabidopsis thaliana chromosome 3, complete sequence
AT5G21910	-1.498721164	0.05312286	Arabidopsis thaliana uncharacterized protein mRNA, complete cds
AT2G36210	-1.193866582	0.053226171	Arabidopsis thaliana chromosome 2, complete sequence
AT5G41590	-1.193866582	0.053226171	Arabidopsis thaliana uncharacterized protein mRNA, complete cds
AT2G02310	-1.193866582	0.053226171	Arabidopsis thaliana putative F-box protein PP2-B6 mRNA, complete cds
AT5G41685	-1.193866582	0.053226171	Arabidopsis thaliana chromosome 5 sequence
AT2G21010	-1.193866582	0.053226171	Arabidopsis thaliana Calcium-dependent lipid-binding (CaLB domain) family protein mRNA, complete cds
AT2G01008	-2.498721164	0.055214957	Arabidopsis thaliana uncharacterized protein mRNA, complete cds
AT2G04300	-2.498721164	0.055214957	Arabidopsis thaliana putative leucine-rich repeat receptor-like serine/threonine-protein kinase mRNA, complete cds
ATCG01090	-2.498721164	0.055214957	Arabidopsis thaliana chloroplast DNA, complete genome, ecotype: Columbia
AT2G01770	-2.498721164	0.055214957	Arabidopsis thaliana vacuolar iron transporter 1 mRNA, complete cds
AT4G22050	-2.498721164	0.055214957	Arabidopsis thaliana aspartyl protease family

			protein mRNA, complete cds
AT1G17710	-2.498721164	0.055214957	Arabidopsis thaliana phosphoethanolamine/phosphocholine phosphatase mRNA, complete cds
AT2G27120	-2.498721164	0.055214957	Arabidopsis thaliana DNA polymerase epsilon catalytic subunit B mRNA, complete cds
AT4G22210	-2.498721164	0.055214957	Arabidopsis thaliana clone asmbl_12628 defensin-like mRNA sequence
AT1G11482	-2.498721164	0.055214957	Arabidopsis thaliana chromosome 1 sequence
AT4G28170	-2.498721164	0.055214957	Arabidopsis thaliana uncharacterized protein mRNA, complete cds
AT2G28670	-2.498721164	0.055214957	Arabidopsis thaliana protein ENHANCED SUBERIN 1 mRNA, complete cds
AT2G39490	-2.498721164	0.055214957	Arabidopsis thaliana F-box protein mRNA, complete cds
AT1G34490	-2.498721164	0.055214957	Arabidopsis thaliana chromosome 1 sequence
AT2G17055	-2.498721164	0.055214957	Arabidopsis thaliana chromosome 2, complete sequence
AT5G39460	-2.498721164	0.055214957	Arabidopsis thaliana chromosome 5 sequence
AT5G24040	-1.065761757	0.055651714	Arabidopsis thaliana uncharacterized protein mRNA, complete cds
AT3G17600	-1.650724257	0.0582352	Arabidopsis thaliana auxin-responsive protein IAA31 mRNA, complete cds
AT2G38510	-1.650724257	0.0582352	Arabidopsis thaliana chromosome 2, complete sequence
AT1G53080	-1.650724257	0.0582352	Arabidopsis thaliana chromosome 1 sequence
AT3G07260	-1.913758663	0.060600823	Arabidopsis thaliana SMAD/FHA domain-containing protein mRNA, complete cds
AT1G65670	-1.913758663	0.060600823	Arabidopsis thaliana cytochrome P450, family 702, subfamily A, polypeptide 1 mRNA, complete cds
AT4G30730	-1.913758663	0.060600823	Arabidopsis thaliana chromosome 4 sequence
AT3G05860	-1.913758663	0.060600823	Arabidopsis thaliana MADS-box transcription factor family protein mRNA, complete cds
AT2G28056	-1.913758663	0.060600823	Arabidopsis thaliana mRNA for unknown protein, complete cds, clone: RAFL21-01-E23
ATMG00080	-1.913758663	0.060600823	Arabidopsis thaliana ecotype Landsberg erecta mitochondrion, complete genome
AT5G60220	-1.913758663	0.060600823	Arabidopsis thaliana tetraspanin4 mRNA, complete cds
AT2G24545	-1.913758663	0.060600823	Arabidopsis thaliana chromosome 2, complete sequence
AT4G18990	-1.913758663	0.060600823	Arabidopsis thaliana probable xyloglucan endotransglucosylase/hydrolase protein 29

			mRNA, complete cds
AT5G09910	-1.913758663	0.060600823	Arabidopsis thaliana Ras-related small GTP-binding family protein mRNA, complete cds
AT4G10860	-1.913758663	0.060600823	Arabidopsis thaliana chromosome 4 sequence
AT5G61100	-1.913758663	0.060600823	Arabidopsis thaliana uncharacterized protein mRNA, complete cds
AT1G14550	-1.235686758	0.062062429	Arabidopsis thaliana peroxidase 5 mRNA, complete cds
AT5G58630	-1.235686758	0.062062429	Arabidopsis thaliana uncharacterized protein mRNA, complete cds
AT3G59730	-1.235686758	0.062062429	Arabidopsis thaliana chromosome 3, complete sequence
AT1G73910	-1.235686758	0.062062429	Arabidopsis thaliana actin-related protein 4A mRNA, complete cds
AT1G71890	-1.083683665	0.065716654	Arabidopsis thaliana sucrose transport protein SUC5 mRNA, complete cds
AT2G29590	-1.083683665	0.065716654	Arabidopsis thaliana thioesterase-like protein mRNA, complete cds
AT5G55570	-1.083683665	0.065716654	Arabidopsis thaliana uncharacterized protein mRNA, complete cds
AT3G18530	-1.083683665	0.065716654	Arabidopsis thaliana ARM repeat family protein-like protein mRNA, complete cds
AT2G17940	-1.292270286	0.072046141	Arabidopsis thaliana uncharacterized protein mRNA, complete cds
AT4G06536	-1.292270286	0.072046141	Arabidopsis thaliana chromosome 4 sequence
AT1G53690	-1.292270286	0.072046141	Arabidopsis thaliana uncharacterized protein mRNA, complete cds
AT1G02570	-1.292270286	0.072046141	Arabidopsis thaliana uncharacterized protein mRNA, complete cds
AT1G50310	-1.292270286	0.072046141	Arabidopsis thaliana sugar transporter 9 mRNA, complete cds
AT4G02650	-1.106403741	0.077645048	Arabidopsis thaliana putative clathrin assembly protein mRNA, complete cds
AT2G20723	-1.106403741	0.077645048	Arabidopsis thaliana chromosome 2, complete sequence

Up regulated genes in *fc1*(+Cd)/*fc1*(-Cd)

Gene	Log2FoldChange	pval	NT:Description
AT3G45140	2.8869626	0	Arabidopsis thaliana lipoxygenase 2 mRNA, complete cds
AT1G29910	1.668462885	0	Arabidopsis thaliana chromosome 1 sequence
AT1G52400	2.849407707	0	Arabidopsis thaliana beta glucosidase 18 mRNA, complete cds
AT2G34430	2.100690478	0	Arabidopsis thaliana chromosome 2, complete sequence
AT3G25770	4.870837314	0	Arabidopsis thaliana allene oxide cyclase 2 mRNA,

			complete cds
AT5G24770	3.588272609	0	Arabidopsis thaliana acid phosphatase VSP2 mRNA, complete cds
AT5G24780	6.063109837	0	Arabidopsis thaliana acid phosphatase VSP1 mRNA, complete cds
AT3G54890	1.156237406	0	Arabidopsis thaliana chlorophyll a-b binding protein 6 mRNA, complete cds
AT1G19670	3.79796086	0	Arabidopsis thaliana chlorophyllase 1 mRNA, complete cds
AT1G13930	2.699039463	0	Arabidopsis thaliana salt tolerance-related protein mRNA, complete cds
AT3G22231	3.836686217	0	Arabidopsis thaliana protein PATHOGEN AND CIRCADIAN CONTROLLED 1 mRNA, complete cds
AT1G12090	2.307854251	0	Arabidopsis thaliana chromosome 1 sequence
AT4G23600	5.599302723	0	Arabidopsis thaliana cystine lyase CORI3 mRNA, complete cds
AT1G20620	1.162740391	0	Arabidopsis thaliana catalase 3 mRNA, complete cds
AT5G42650	2.897727027	0	Arabidopsis thaliana chromosome 5 sequence
AT2G21660	2.08369519	0	Arabidopsis thaliana glycine-rich RNA-binding protein 7 mRNA, complete cds
AT4G18440	3.309683995	0	Arabidopsis thaliana L-aspartase-like family protein mRNA, complete cds
AT2G24850	7.849961477	0	Arabidopsis thaliana tyrosine aminotransferase 3 mRNA, complete cds
AT5G11670	1.750566453	0	Arabidopsis thaliana NADP-dependent malic enzyme 2 mRNA, complete cds
AT3G26740	2.275402163	0	Arabidopsis thaliana CCR-like protein mRNA, complete cds
AT5G20230	3.444933941	0	Arabidopsis thaliana blue-copper-binding protein mRNA, complete cds
AT2G42540	6.636127815	0	Arabidopsis thaliana cold-regulated protein 15a mRNA, complete cds
AT4G02520	1.831844165	0	Arabidopsis thaliana glutathione S-transferase F2 mRNA, complete cds
AT1G31580	1.259831227	0	Arabidopsis thaliana protein ECS1 mRNA, complete cds
AT4G34710	2.255992878	0	Arabidopsis thaliana chromosome 4 sequence
AT1G15520	5.445748248	0	Arabidopsis thaliana ABC transporter G family member 40 mRNA, complete cds
AT5G15970	4.458483109	0	Arabidopsis thaliana stress-induced protein KIN2 mRNA, complete cds
AT5G50950	3.702636724	0	Arabidopsis thaliana fumarate hydratase 2 mRNA, complete cds
AT4G08870	2.778766758	0	Arabidopsis thaliana arginine amidohydrolase 2 mRNA, complete cds

AT1G19570	1.8719011	0	Arabidopsis thaliana dehydroascorbate reductase mRNA, complete cds
AT3G27690	1.740296853	0	Arabidopsis thaliana photosystem II light harvesting complex protein 2.3 mRNA, complete cds
AT1G02930	2.797828772	0	Arabidopsis thaliana glutathione S-transferase F6 mRNA, complete cds
AT1G05010	1.606081302	0	Arabidopsis thaliana 1-aminocyclopropane-1-carboxylate oxidase mRNA, complete cds
AT1G02920	2.70232684	0	Arabidopsis thaliana glutathione S-transferase F7 mRNA, complete cds
AT4G37370	4.99843841	0	Arabidopsis thaliana cytochrome P450, family 81, subfamily D, polypeptide 8 mRNA, complete cds
AT3G46970	2.666393614	0	Arabidopsis thaliana alpha-glucan phosphorylase 2 mRNA, complete cds
AT5G35735	2.865434778	0	Arabidopsis thaliana putative auxin-responsive protein mRNA, complete cds
AT2G14560	3.325517799	0	Arabidopsis thaliana protein LURP1 mRNA, complete cds
AT2G38470	2.65603014	0	Arabidopsis thaliana putative WRKY transcription factor 33 mRNA, complete cds
AT5G11740	1.719512435	0	Arabidopsis thaliana chromosome 5 sequence
AT2G15970	2.913886901	0	Arabidopsis thaliana cold regulated 413 plasma membrane 1 mRNA, complete cds
AT1G69830	3.706859781	0	Arabidopsis thaliana alpha-amylase-like 3 mRNA, complete cds
AT3G57260	4.507146277	0	Arabidopsis thaliana beta 1,3-glucanase 2 mRNA, complete cds
AT3G22235	3.268799858	0	Arabidopsis thaliana uncharacterized protein mRNA, complete cds
AT3G44860	8.683796594	0	Arabidopsis thaliana farnesoic acid carboxyl-O-methyltransferase mRNA, complete cds
AT4G21990	2.622155077	0	Arabidopsis thaliana 5'-adenylylsulfate reductase 3 mRNA, complete cds
AT4G01870	4.493443512	0	Arabidopsis thaliana chromosome 4 sequence
AT2G25510	1.172162418	0	Arabidopsis thaliana uncharacterized protein mRNA, complete cds
AT2G39330	4.157812484	0	Arabidopsis thaliana jacalin-related lectin 23 mRNA, complete cds
AT4G16260	2.967041881	0	Arabidopsis thaliana putative beta-1,3-endoglucanase mRNA, complete cds
AT1G70700	1.92081489	0	Arabidopsis thaliana putative jasmonate signaling protein JAZ9 mRNA, complete cds
AT1G32640	2.425733152	0	Arabidopsis thaliana chromosome 1 sequence
AT1G14250	4.501982291	0	Arabidopsis thaliana probable apyrase 5 mRNA, complete cds

AT4G24350	2.497744218	0	Arabidopsis thaliana phosphorylase family protein mRNA, complete cds
AT4G04020	1.951118523	0	Arabidopsis thaliana fibrillin mRNA, complete cds
AT5G53450	2.185893953	0	Arabidopsis thaliana OBP3-responsive protein 1 mRNA, complete cds
AT1G76680	2.313157085	0	Arabidopsis thaliana 12-oxophytodienoate reductase 1 mRNA, complete cds
AT4G34950	1.829815488	0	Arabidopsis thaliana major facilitator family protein mRNA, complete cds
AT3G26830	4.536942573	0	Arabidopsis thaliana protein PHYTOALEXIN DEFICIENT 3 mRNA, complete cds
AT2G42530	6.131117617	0	Arabidopsis thaliana cold-regulated protein 15b mRNA, complete cds
AT1G52040	3.694975048	0	Arabidopsis thaliana myrosinase-binding protein 1 mRNA, complete cds
AT5G08790	2.911526856	0	Arabidopsis thaliana protein ATAF2 mRNA, complete cds
AT2G25450	1.490490479	0	Arabidopsis thaliana 1-aminocyclopropane-1-carboxylate oxidase-like protein mRNA, complete cds
AT1G72450	2.434108916	0	Arabidopsis thaliana jasmonate-zim-domain protein 6 mRNA, complete cds
AT5G26340	4.167643493	1.36E-304	Arabidopsis thaliana sugar transport protein 13 mRNA, complete cds
AT4G14400	1.147751141	6.89E-302	Arabidopsis thaliana protein ACCELERATED CELL DEATH 6 mRNA, complete cds
AT4G27520	1.185075778	9.33E-301	Arabidopsis thaliana early nodulin-like protein 2 mRNA, complete cds
AT3G23810	1.236789515	1.97E-293	Arabidopsis thaliana adenosylhomocysteinase 2 mRNA, complete cds
AT4G30650	3.773696934	1.39E-290	Arabidopsis thaliana putative low temperature and salt responsive protein mRNA, complete cds
AT5G51070	1.17925867	7.39E-289	Arabidopsis thaliana chaperone protein ClpD mRNA, complete cds
AT1G80840	4.621404913	1.02E-286	Arabidopsis thaliana putative WRKY transcription factor 40 mRNA, complete cds
AT1G75040	3.474891777	5.05E-282	Arabidopsis thaliana pathogenesis-related protein 5 mRNA, complete cds
AT3G09390	1.642687685	7.06E-277	Arabidopsis thaliana metallothionein 2A mRNA, complete cds
AT1G45201	1.493251821	2.89E-274	Arabidopsis thaliana triacylglycerol lipase-like 1 mRNA, complete cds
AT2G26560	3.91425154	1.44E-271	Arabidopsis thaliana phospholipase A 2A mRNA, complete cds
AT1G52410	2.180922197	2.24E-269	Arabidopsis thaliana TSK-associating protein 1 mRNA, complete cds

AT1G51760	2.182942476	5.45E-269	Arabidopsis thaliana IAA-amino acid hydrolase IAR3 mRNA, complete cds
AT2G06050	2.480711181	1.35E-268	Arabidopsis thaliana AT2G06050 mRNA, complete cds, clone: RAFL22-42-B17
AT2G03760	3.02381734	4.72E-263	Arabidopsis thaliana chromosome 2, complete sequence
AT3G29320	1.611717924	3.67E-261	Arabidopsis thaliana alpha-glucan phosphorylase 1 mRNA, complete cds
AT4G02380	1.34043314	1.17E-259	Arabidopsis thaliana senescence-associated protein SAG21 mRNA, complete cds
AT1G20510	2.283508121	1.90E-258	Arabidopsis thaliana OPC-8:0 CoA ligase1 mRNA, complete cds
AT4G17470	5.719448541	3.16E-252	Arabidopsis thaliana putative palmitoyl-protein thioesterase mRNA, complete cds
AT1G29395	4.69540953	2.39E-250	Arabidopsis thaliana cold regulated 314 inner membrane 1 mRNA, complete cds
AT5G03350	3.176106455	4.25E-248	Arabidopsis thaliana chromosome 5 sequence
AT3G49620	6.872796805	1.69E-244	Arabidopsis thaliana 2-oxoacid-dependent dioxygenase-like protein DIN11 mRNA, complete cds
AT5G06320	1.448216566	6.47E-244	Arabidopsis thaliana chromosome 5 sequence
AT3G45640	1.561127231	8.48E-244	Arabidopsis thaliana mitogen-activated protein kinase 3 mRNA, complete cds
AT1G70820	2.018737702	2.34E-242	Arabidopsis thaliana phosphoglucomutase-like protein mRNA, complete cds
AT3G14620	2.091876224	1.41E-239	Arabidopsis thaliana cytochrome P450, family 72, subfamily A, polypeptide 8 mRNA, complete cds
AT2G34810	3.021761876	2.31E-232	Arabidopsis thaliana chromosome 2, complete sequence
AT3G48990	1.145284168	8.60E-232	Arabidopsis thaliana 4-coumarate--CoA ligase-like 10 mRNA, complete cds
AT5G05600	3.896404643	1.90E-230	Arabidopsis thaliana oxidoreductase, 2OG-Fe(II) oxygenase family protein mRNA, complete cds
AT4G39980	1.585793569	4.44E-229	Arabidopsis thaliana 3-deoxy-D-arabino-heptulosonate 7-phosphate synthase 1 mRNA, complete cds
AT5G06870	2.915249863	2.54E-228	Arabidopsis thaliana polygalacturonase inhibitor 2 mRNA, complete cds
AT3G16470	1.176252562	2.96E-228	Arabidopsis thaliana JA-responsive protein 1 mRNA, complete cds
AT1G19180	2.856164805	2.10E-226	Arabidopsis thaliana chromosome 1 sequence
AT1G05680	7.685405078	2.87E-225	Arabidopsis thaliana Uridine diphosphate glycosyltransferase 74E2 mRNA, complete cds
AT4G24570	3.363417072	6.34E-222	Arabidopsis thaliana chromosome 4 sequence
AT4G26530	1.143043599	1.65E-221	Arabidopsis thaliana fructose-bisphosphate aldolase 5 mRNA, complete cds
AT5G23820	1.55354863	1.92E-221	Arabidopsis thaliana MD-2-related lipid recognition domain-containing protein mRNA, complete cds

AT4G15210	6.543623628	6.29E-220	Arabidopsis thaliana beta-amylase 5 mRNA, complete cds
AT4G14365	1.937024429	2.37E-216	Arabidopsis thaliana putative E3 ubiquitin-protein ligase XBAT34 mRNA, complete cds
AT2G40840	1.653359351	4.54E-214	Arabidopsis thaliana 4-alpha-glucanotransferase DPE2 mRNA, complete cds
AT3G18080	1.145367918	4.78E-214	Arabidopsis thaliana beta-glucosidase 44 mRNA, complete cds
AT3G50930	2.752895024	1.76E-212	Arabidopsis thaliana chromosome 3, complete sequence
AT3G56710	3.836767582	3.45E-210	Arabidopsis thaliana chromosome 3, complete sequence
AT1G01560	4.992891264	1.47E-209	Arabidopsis thaliana mitogen-activated protein kinase 11 mRNA, complete cds
AT5G02940	1.401851976	3.00E-209	Arabidopsis thaliana uncharacterized protein mRNA, complete cds
AT1G43670	1.369142499	1.26E-207	Arabidopsis thaliana fructose-1,6-bisphosphatase mRNA, complete cds
AT2G26900	1.227424644	3.82E-201	Arabidopsis thaliana sodium/pyruvate cotransporter BASS2 mRNA, complete cds
AT3G22370	2.401669919	3.87E-201	Arabidopsis thaliana alternative oxidase 1A mRNA, complete cds
AT1G36370	1.957103501	1.78E-200	Arabidopsis thaliana putative serine hydroxymethyltransferase mRNA, complete cds
AT4G11280	2.599258327	3.37E-199	Arabidopsis thaliana 1-aminocyclopropane-1-carboxylate synthase 6 mRNA, complete cds
AT5G04340	3.868799012	5.84E-198	Arabidopsis thaliana chromosome 5 sequence
AT5G02810	2.609669501	4.55E-197	Arabidopsis thaliana pseudo-response regulator 7 mRNA, complete cds
AT3G56200	3.006261049	2.12E-196	Arabidopsis thaliana putative amino acid transporter mRNA, complete cds
AT1G06460	1.665276052	3.60E-196	Arabidopsis thaliana alpha-crystallin domain 32.1 mRNA, complete cds
AT3G54040	1.650254982	3.08E-194	Arabidopsis thaliana PAR1 protein mRNA, complete cds
AT5G47240	4.281095875	1.22E-193	Arabidopsis thaliana nudix hydrolase 8 mRNA, complete cds
AT1G16410	2.643824868	7.34E-190	Arabidopsis thaliana dihomomethionine N-hydroxylase mRNA, complete cds
AT3G04720	2.162814764	3.80E-186	Arabidopsis thaliana pathogenesis-related 4 mRNA, complete cds
AT2G43010	2.491862971	1.31E-183	Arabidopsis thaliana transcription factor PIF4 mRNA, complete cds
AT3G51450	3.338270609	3.63E-183	Arabidopsis thaliana strictosidine synthase family protein mRNA, complete cds
AT4G16590	5.971525527	1.25E-180	Arabidopsis thaliana cellulose synthase-like A01 mRNA, complete cds
AT4G04840	2.963391588	4.54E-179	Arabidopsis thaliana methionine sulfoxide reductase B6

			mRNA, complete cds
AT5G16970	2.009720943	7.64E-179	Arabidopsis thaliana 2-alkenal reductase mRNA, complete cds
AT1G61890	2.262049939	1.37E-178	Arabidopsis thaliana MATE efflux family protein mRNA, complete cds
AT2G43530	2.834243564	5.68E-175	Arabidopsis thaliana defensin-like protein 194 mRNA, complete cds
AT4G21910	1.75133687	9.83E-174	Arabidopsis thaliana MATE efflux family protein mRNA, complete cds
AT3G25760	3.901286864	1.58E-172	Arabidopsis thaliana allene oxide cyclase 1 mRNA, complete cds
AT5G64120	3.283626476	3.16E-172	Arabidopsis thaliana peroxidase 71 mRNA, complete cds
AT3G28220	2.153471711	1.33E-171	Arabidopsis thaliana TRAF-like family protein mRNA, complete cds
AT5G57560	2.080274533	2.59E-171	Arabidopsis thaliana xyloglucan endotransglucosylase/hydrolase protein 22 mRNA, complete cds
AT5G10760	2.983849459	2.75E-168	Arabidopsis thaliana aspartyl protease family protein mRNA, complete cds
AT1G72520	3.311570834	6.76E-166	Arabidopsis thaliana lipoxygenase 4 mRNA, complete cds
AT2G39800	1.594966152	1.17E-164	Arabidopsis thaliana delta1-pyrroline-5-carboxylate synthase 1 mRNA, complete cds
AT1G10760	1.105537741	4.98E-163	Arabidopsis thaliana alpha-glucan water dikinase 1 mRNA, complete cds
AT2G47180	2.926956196	1.28E-162	Arabidopsis thaliana galactinol synthase 1 mRNA, complete cds
AT5G54960	2.103682377	3.77E-159	Arabidopsis thaliana chromosome 5 sequence
AT4G11290	1.683510655	4.45E-158	Arabidopsis thaliana peroxidase 39 mRNA, complete cds
AT5G26220	5.126103624	1.72E-157	Arabidopsis thaliana ChaC-like family protein mRNA, complete cds
AT1G78680	1.555507578	1.89E-157	Arabidopsis thaliana gamma-glutamyl hydrolase 2 mRNA, complete cds
AT4G12730	1.115232767	2.39E-157	Arabidopsis thaliana chromosome 4 sequence
AT2G29350	4.133435279	1.17E-156	Arabidopsis thaliana senescence-associated protein 13 mRNA, complete cds
AT1G70830	1.148044893	5.02E-154	Arabidopsis thaliana MLP-like protein 28 mRNA, complete cds
AT3G57520	1.046541372	1.37E-153	Arabidopsis thaliana putative galactinol--sucrose galactosyltransferase 2 mRNA, complete cds
AT3G15356	1.330215238	7.62E-153	Arabidopsis thaliana chromosome 3, complete sequence
AT4G15760	2.404997257	3.96E-152	Arabidopsis thaliana monooxygenase 1 mRNA, complete cds
AT4G34150	1.226614555	7.45E-151	Arabidopsis thaliana calcium-dependent lipid-binding domain-containing protein mRNA, complete cds

AT5G02490	1.211150204	1.06E-150	Arabidopsis thaliana heat shock protein 70 mRNA, complete cds
AT3G26290	4.599778311	2.99E-150	Arabidopsis thaliana cytochrome P450 71B26 mRNA, complete cds
AT4G37980	1.484043869	3.68E-149	Arabidopsis thaliana cinnamyl alcohol dehydrogenase 7 mRNA, complete cds
AT1G20450	1.13076871	1.31E-146	Arabidopsis thaliana dehydrin ERD10 mRNA, complete cds
AT1G60730	2.342003462	1.68E-146	Arabidopsis thaliana probable aldo-keto reductase 5 mRNA, complete cds
AT5G43350	1.178893681	1.28E-145	Arabidopsis thaliana inorganic phosphate transporter 1-1 mRNA, complete cds
AT4G01080	3.53562028	1.39E-145	Arabidopsis thaliana protein TRICHOME BIREFRINGENCE-LIKE 26 mRNA, complete cds
AT4G16760	1.150584749	1.95E-144	Arabidopsis thaliana peroxisomal acyl-coenzyme A oxidase 1 mRNA, complete cds
AT1G02205	1.181363451	3.94E-144	Arabidopsis thaliana protein ECERIFERUM 1 mRNA, complete cds
AT3G01420	1.793740077	3.39E-142	Arabidopsis thaliana alpha-dioxygenase mRNA, complete cds
AT1G09070	1.115501954	5.45E-142	Arabidopsis thaliana chromosome 1 sequence
AT1G56300	4.102399364	1.09E-141	Arabidopsis thaliana chaperone DnaJ-domain containing protein mRNA, complete cds
AT3G09350	1.640303198	5.86E-141	Arabidopsis thaliana protein Fes1A mRNA, complete cds
AT5G52310	2.085032852	2.45E-139	Arabidopsis thaliana protein LOW-TEMPERATURE-INDUCED 78 mRNA, complete cds
AT3G05500	1.913715039	9.41E-139	Arabidopsis thaliana Rubber elongation factor protein mRNA, complete cds
AT4G33050	1.548772598	1.89E-138	Arabidopsis thaliana calmodulin-binding protein mRNA, complete cds
AT5G59820	3.729126858	4.09E-138	Arabidopsis thaliana chromosome 5 sequence
AT2G39030	10.88518053	8.76E-138	Arabidopsis thaliana chromosome 2, complete sequence
AT5G59540	1.772794582	4.95E-137	Arabidopsis thaliana oxidoreductase, 2OG-Fe(II) oxygenase family protein mRNA, complete cds
AT3G52400	1.713751944	7.60E-137	Arabidopsis thaliana syntaxin-122 mRNA, complete cds
AT1G64370	1.047251792	4.78E-136	Arabidopsis thaliana chromosome 1 sequence
AT5G59130	3.237560736	4.99E-136	Arabidopsis thaliana Subtilase family protein mRNA, complete cds
AT2G28900	1.387445534	9.53E-136	Arabidopsis thaliana outer plastid envelope protein 16-1 mRNA, complete cds
AT4G39260	1.313225125	1.52E-135	Arabidopsis thaliana glycine-rich RNA-binding protein 8 mRNA, complete cds
AT1G27730	3.16605279	5.99E-135	Arabidopsis thaliana chromosome 1 sequence
AT5G45820	5.130105555	2.50E-133	Arabidopsis thaliana chromosome 5 sequence

AT2G29460	5.029931662	1.46E-131	Arabidopsis thaliana glutathione S-transferase tau 4 mRNA, complete cds
AT5G53420	2.464325526	1.43E-130	Arabidopsis thaliana CCT motif family protein mRNA, complete cds
AT1G72180	1.727662479	5.69E-130	Arabidopsis thaliana leucine-rich receptor-like protein kinase mRNA, complete cds
AT5G63790	1.926006543	1.13E-129	Arabidopsis thaliana NAC domain-containing protein 102 mRNA, complete cds
AT2G18690	1.878411914	1.18E-129	Arabidopsis thaliana chromosome 2, complete sequence
AT1G76790	1.912383702	6.62E-127	Arabidopsis thaliana indole glucosinolate o-methyltransferase 5 mRNA, complete cds
AT3G06500	1.73549604	7.52E-127	Arabidopsis thaliana protein alkaline/neutral invertase C mRNA, complete cds
AT2G43550	2.447852122	1.16E-126	Arabidopsis thaliana defensin-like protein 197 mRNA, complete cds
AT3G25780	2.073201202	4.49E-126	Arabidopsis thaliana allene oxide cyclase 3 mRNA, complete cds
AT5G25250	2.303003693	1.43E-125	Arabidopsis thaliana Flotillin-like protein 1 mRNA, complete cds
AT3G28210	4.746116492	5.36E-125	Arabidopsis thaliana zinc finger (AN1-like) family protein mRNA, complete cds
AT1G20693	1.271557098	1.10E-123	Arabidopsis thaliana high mobility group B2 protein mRNA, complete cds
AT1G73260	3.015253853	2.94E-123	Arabidopsis thaliana chromosome 1 sequence
AT1G78000	1.242790709	4.22E-123	Arabidopsis thaliana sulfate transporter 1;2 mRNA, complete cds
AT4G05020	2.17225727	6.60E-123	Arabidopsis thaliana NAD(P)H dehydrogenase B2 mRNA, complete cds
AT3G56400	1.704954958	1.31E-122	Arabidopsis thaliana WRKY transcription factor 70 mRNA, complete cds
AT1G71030	4.385983398	1.58E-122	Arabidopsis thaliana putative myb family transcription factor mRNA, complete cds
AT3G28930	1.862699033	1.29E-121	Arabidopsis thaliana avrRpt2-induced protein AIG2 mRNA, complete cds
AT1G21250	1.067936734	2.04E-121	Arabidopsis thaliana wall-associated receptor kinase 1 mRNA, complete cds
AT1G17170	4.413580297	1.75E-120	Arabidopsis thaliana glutathione S-transferase TAU 24 mRNA, complete cds
AT2G47000	1.385055508	1.19E-119	Arabidopsis thaliana auxin efflux transmembrane transporter MDR4 mRNA, complete cds
AT3G16530	1.092414897	2.72E-119	Arabidopsis thaliana chromosome 3, complete sequence
AT4G30270	1.71397772	3.20E-119	Arabidopsis thaliana xyloglucan endotransglucosylase/hydrolase protein 24 mRNA, complete cds

AT1G68600	2.204918693	3.48E-119	Arabidopsis thaliana Aluminum activated malate transporter family protein mRNA, complete cds
AT1G52000	1.576160465	1.57E-117	Arabidopsis thaliana mannose-binding lectin superfamily protein mRNA, complete cds
AT4G23170	2.245287749	1.51E-116	Arabidopsis thaliana chromosome 4 sequence
AT5G03630	1.070232318	8.19E-116	Arabidopsis thaliana monodehydroascorbate reductase (NADH) mRNA, complete cds
AT1G73480	2.303508659	1.06E-115	Arabidopsis thaliana alpha/beta-Hydrolases superfamily protein mRNA, complete cds
AT2G23810	1.318121872	6.25E-115	Arabidopsis thaliana tetraspanin8 mRNA, complete cds
AT2G40080	3.708894028	1.00E-114	Arabidopsis thaliana chromosome 2, complete sequence
AT3G07390	1.280512662	2.45E-114	Arabidopsis thaliana chromosome 3, complete sequence
AT1G75280	1.167844638	1.51E-112	Arabidopsis thaliana putative Isoflavone reductase mRNA, complete cds
AT1G44350	3.701911096	2.01E-111	Arabidopsis thaliana IAA-amino acid hydrolase ILR1-like 6 mRNA, complete cds
AT4G18950	1.511988686	2.89E-111	Arabidopsis thaliana Integrin-linked protein kinase family protein mRNA, complete cds
AT1G24147	3.087762175	2.05E-110	Arabidopsis thaliana uncharacterized protein mRNA, complete cds
AT5G67480	1.978017239	4.79E-110	Arabidopsis thaliana BTB and TAZ domain protein 4 mRNA, complete cds
AT4G27560	2.08108211	5.51E-110	Arabidopsis thaliana chromosome 4 sequence
AT2G22500	1.610435704	5.88E-110	Arabidopsis thaliana chromosome 2, complete sequence
AT1G01470	1.479760029	9.81E-110	Arabidopsis thaliana putative desiccation-related protein LEA14 mRNA, complete cds
AT3G49110	1.745257307	1.08E-108	Arabidopsis thaliana peroxidase 33 mRNA, complete cds
AT3G62550	2.556458367	4.31E-108	Arabidopsis thaliana drought responsive ATP-binding motif containing protein mRNA, complete cds
AT5G51830	2.305677119	6.69E-108	Arabidopsis thaliana pfkB-like carbohydrate kinase family protein mRNA, complete cds
AT5G48180	1.178682561	7.18E-108	Arabidopsis thaliana nitrile specifier protein 5 mRNA, complete cds
AT5G13200	2.523065026	2.29E-107	Arabidopsis thaliana GRAM domain family protein mRNA, complete cds
AT1G52030	2.344810286	3.82E-107	Arabidopsis thaliana myrosinase-binding protein 2 mRNA, complete cds
AT3G61820	1.389753241	5.68E-106	Arabidopsis thaliana chromosome 3, complete sequence
AT5G51440	1.962699624	8.25E-106	Arabidopsis thaliana mRNA for mitochondrial heat shock 22 kd protein-like, complete cds, clone: RAFL21-16-A12
AT5G07010	3.8165897	9.88E-106	Arabidopsis thaliana chromosome 5 sequence
AT3G02570	1.330625956	1.37E-105	Arabidopsis thaliana mannose-6-phosphate isomerase mRNA, complete cds

AT1G17380	3.513455735	2.53E-105	Arabidopsis thaliana protein TIFY 11A mRNA, complete cds
AT2G26440	2.652117967	2.68E-103	Arabidopsis thaliana Probable pectinesterase/pectinesterase inhibitor 12 mRNA, complete cds
AT5G37600	1.015500758	3.70E-103	Arabidopsis thaliana glutamine synthetase 1;1 mRNA, complete cds
AT5G61160	5.926155738	5.06E-103	Arabidopsis thaliana chromosome 5 sequence
AT4G12320	1.204793762	1.07E-102	Arabidopsis thaliana cytochrome P450, family 706, subfamily A, polypeptide 6 mRNA, complete cds
AT2G43820	1.826191148	2.80E-102	Arabidopsis thaliana UDP-glucosyltransferase 74F2 mRNA, complete cds
AT5G03380	1.03063048	2.93E-102	Arabidopsis thaliana heavy metal transport/detoxification domain-containing protein mRNA, complete cds
AT3G20810	2.201054594	3.47E-102	Arabidopsis thaliana jumonji-C domain-containing protein 30 mRNA, complete cds
AT5G01100	2.477139821	5.86E-102	Arabidopsis thaliana O-fucosyltransferase family protein mRNA, complete cds
AT3G22200	1.344948151	2.28E-101	Arabidopsis thaliana gamma-aminobutyrate transaminase POP2 mRNA, complete cds
AT1G19960	3.701774522	5.51E-101	Arabidopsis thaliana chromosome 1 sequence
AT5G07440	1.235610867	1.18E-100	Arabidopsis thaliana glutamate dehydrogenase 2 mRNA, complete cds
AT1G42990	1.593880724	1.26E-100	Arabidopsis thaliana bZIP transcription factor 60 mRNA, complete cds
AT4G09020	2.018914378	1.36E-100	Arabidopsis thaliana isoamylase 3 mRNA, complete cds
AT2G31360	1.105684083	6.39E-100	Arabidopsis thaliana 16:0delta9 desaturase 2 mRNA, complete cds
AT4G23210	4.390324945	1.11E-99	Arabidopsis thaliana cysteine-rich receptor-like protein kinase 13 mRNA, complete cds
AT4G34135	1.753444727	1.39E-99	Arabidopsis thaliana UDP-glucosyltransferase 73B2 mRNA, complete cds
AT5G43440	3.404971721	1.96E-99	Arabidopsis thaliana 1-aminocyclopropane-1-carboxylate oxidase-like protein 9 mRNA, complete cds
AT1G61120	7.820777497	6.26E-99	Arabidopsis thaliana terpene synthase 04 mRNA, complete cds
AT4G34120	1.132256792	9.93E-98	Arabidopsis thaliana cystathionine beta-synthase domain-containing protein mRNA, complete cds
AT4G36500	1.363638149	2.02E-97	Arabidopsis thaliana chromosome 4 sequence
AT5G17380	1.405672909	4.38E-97	Arabidopsis thaliana 2-hydroxyacyl-CoA lyase mRNA, complete cds
AT1G49570	1.472666393	8.07E-97	Arabidopsis thaliana peroxidase mRNA, complete cds
AT1G74950	1.596775822	3.58E-96	Arabidopsis thaliana protein TIFY 10B mRNA, complete

			cds
AT4G08620	1.700818145	5.31E-96	Arabidopsis thaliana sulfate transporter 1.1 mRNA, complete cds
AT3G30775	1.027689144	1.84E-95	Arabidopsis thaliana proline dehydrogenase 1 mRNA, complete cds
AT5G17000	1.909563264	5.39E-94	Arabidopsis thaliana zinc-binding dehydrogenase family protein mRNA, complete cds
AT3G47960	1.145571014	5.80E-94	Arabidopsis thaliana glucosinolate transporter 1 mRNA, complete cds
AT1G17420	2.119123218	9.68E-94	Arabidopsis thaliana lipoxygenase 3 mRNA, complete cds
AT5G42380	4.253948087	1.24E-93	Arabidopsis thaliana chromosome 5 sequence
AT1G31690	1.799834568	4.24E-93	Arabidopsis thaliana copper amine oxidase family protein mRNA, complete cds
AT3G09940	1.352858283	6.06E-93	Arabidopsis thaliana monodehydroascorbate reductase (NADH) mRNA, complete cds
AT3G19010	1.422283055	1.42E-92	Arabidopsis thaliana 2-oxoglutarate (2OG) and Fe(II)-dependent oxygenase superfamily protein mRNA, complete cds
AT5G47220	3.779418467	2.98E-91	Arabidopsis thaliana chromosome 5 sequence
AT5G24420	1.952669143	6.07E-91	Arabidopsis thaliana 6-phosphogluconolactonase 5 mRNA, complete cds
AT1G13280	1.36858596	8.09E-91	Arabidopsis thaliana allene oxide cyclase 4 mRNA, complete cds
AT3G25250	4.33283945	8.15E-91	Arabidopsis thaliana AGC (cAMP-dependent, cGMP-dependent and protein kinase C) kinase family protein mRNA, complete cds
AT2G43570	2.619726425	9.27E-91	Arabidopsis thaliana putative chitinase mRNA, complete cds
AT2G29670	1.486527475	1.09E-90	Arabidopsis thaliana tetratricopeptide repeat-containing protein mRNA, complete cds
AT5G54500	1.023255479	2.03E-90	Arabidopsis thaliana flavodoxin-like quinone reductase 1 mRNA, complete cds
AT2G36800	1.782642368	1.37E-89	Arabidopsis thaliana chromosome 2, complete sequence
AT5G13740	1.310845926	3.85E-89	Arabidopsis thaliana zinc induced facilitator 1 proetin mRNA, complete cds
AT5G43450	2.173453751	3.57E-88	Arabidopsis thaliana 1-aminocyclopropane-1-carboxylate oxidase-like protein mRNA, complete cds
AT4G15440	1.901941296	1.73E-87	Arabidopsis thaliana hydroperoxide lyase 1 mRNA, complete cds
AT1G70890	1.891192354	6.19E-87	Arabidopsis thaliana MLP-like protein 43 mRNA, complete cds
AT3G13080	1.028431393	7.43E-87	Arabidopsis thaliana ABC transporter C family member 3 mRNA, complete cds
AT1G54020	3.961348255	7.58E-87	Arabidopsis thaliana GDSL esterase/lipase mRNA,

			complete cds
AT2G38750	2.350326877	3.93E-86	Arabidopsis thaliana annexin D4 mRNA, complete cds
AT3G52180	1.535755385	8.46E-86	Arabidopsis thaliana phosphoglucan phosphatase DSP4 mRNA, complete cds
AT1G66090	2.192316682	2.25E-85	Arabidopsis thaliana TIR-NBS class of disease resistance protein mRNA, complete cds
AT3G55980	1.242834508	2.89E-85	Arabidopsis thaliana salt-inducible zinc finger 1 mRNA, complete cds
AT4G11600	1.26335435	3.92E-85	Arabidopsis thaliana glutathione peroxidase 6 mRNA, complete cds
AT1G07050	3.230942321	1.40E-84	Arabidopsis thaliana CCT motif family protein mRNA, complete cds
AT2G15480	2.435879253	3.54E-84	Arabidopsis thaliana UDP-glucosyl transferase 73B5 mRNA, complete cds
AT5G47120	1.00344168	5.84E-84	Arabidopsis thaliana BAX inhibitor 1 mRNA, complete cds
AT2G20340	1.925799263	6.81E-84	Arabidopsis thaliana tyrosine decarboxylase 1 mRNA, complete cds
AT5G22430	3.43143648	1.65E-83	Arabidopsis thaliana pollen Ole e 1 allergen and extensin family protein mRNA, complete cds
AT5G48880	2.088181502	1.73E-83	Arabidopsis thaliana 3-keto-acyl-CoA thiolase 2 mRNA, complete cds
AT4G33720	1.260807846	3.62E-83	Arabidopsis thaliana chromosome 4 sequence
AT1G64980	1.491082724	4.07E-83	Arabidopsis thaliana putative nucleotide-diphospho-sugar transferase mRNA, complete cds
AT1G22400	1.708754384	2.20E-82	Arabidopsis thaliana UDP-glycosyltransferase 85A1 mRNA, complete cds
AT4G35630	1.214327115	4.00E-82	Arabidopsis thaliana chromosome 4 sequence
AT3G04290	1.112473754	2.68E-81	Arabidopsis thaliana Li-tolerant lipase 1 mRNA, complete cds
AT4G16146	3.320512161	5.95E-81	Arabidopsis thaliana cAMP-regulated phosphoprotein 19-related protein mRNA, complete cds
AT5G24300	1.082143964	7.94E-81	Arabidopsis thaliana starch synthase 1 mRNA, complete cds
AT1G72910	1.806833832	7.97E-81	Arabidopsis thaliana Toll-Interleukin-Resistance domain-containing protein mRNA, complete cds
AT3G60420	1.604337701	9.59E-81	Arabidopsis thaliana phosphoglycerate mutase family protein mRNA, complete cds
AT3G11820	1.181874164	1.74E-80	Arabidopsis thaliana syntaxin-121 mRNA, complete cds
AT1G32350	7.46312748	5.84E-80	Arabidopsis thaliana alternative oxidase 1D mRNA, complete cds
AT1G13650	1.311439781	8.39E-80	Arabidopsis thaliana uncharacterized protein mRNA, complete cds
AT1G22770	2.328140114	8.65E-79	Arabidopsis thaliana protein GIGANTEA mRNA, complete

			cds
AT4G39030	2.094228518	2.15E-78	Arabidopsis thaliana enhanced disease susceptibility 5 mRNA, complete cds
AT1G77510	1.175102019	1.96E-77	Arabidopsis thaliana protein disulfide isomerase-like 1-2 mRNA, complete cds
AT4G36220	1.010263584	1.22E-76	Arabidopsis thaliana ferulic acid 5-hydroxylase 1 mRNA, complete cds
AT1G16030	2.33925076	1.41E-76	Arabidopsis thaliana chromosome 1 sequence
AT1G32940	2.199217102	3.64E-75	Arabidopsis thaliana Subtilase 3.5 mRNA, complete cds
AT4G15490	2.017351505	6.67E-75	Arabidopsis thaliana chromosome 4 sequence
AT1G31550	2.230316016	1.17E-74	Arabidopsis thaliana GDSL esterase/lipase mRNA, complete cds
AT4G29780	2.354308046	5.72E-74	Arabidopsis thaliana chromosome 4 sequence
AT1G76600	2.47598103	1.33E-73	Arabidopsis thaliana chromosome 1 sequence
AT1G06620	1.967113514	2.42E-73	Arabidopsis thaliana 2-oxoglutarate-dependent dioxygenase-like protein mRNA, complete cds
AT5G15870	1.43145283	2.89E-73	Arabidopsis thaliana chromosome 5 sequence
AT5G59550	1.712820918	4.52E-73	Arabidopsis thaliana chromosome 5 sequence
AT4G32340	2.244661235	4.53E-73	Arabidopsis thaliana tetratricopeptide repeat domain-containing protein-like protein mRNA, complete cds
AT4G21380	3.362023836	1.22E-72	Arabidopsis thaliana receptor kinase 3 mRNA, complete cds
AT1G32920	1.98160122	2.72E-72	Arabidopsis thaliana chromosome 1 sequence
AT1G23710	2.090014627	2.87E-72	Arabidopsis thaliana chromosome 1 sequence
AT2G20870	3.159712017	3.58E-71	Arabidopsis thaliana chromosome 2, complete sequence
AT1G28600	1.165694051	3.75E-71	Arabidopsis thaliana GDSL esterase/lipase mRNA, complete cds
AT1G78490	1.309655197	5.43E-71	Arabidopsis thaliana cytochrome P450, family 708, subfamily A, polypeptide 3 mRNA, complete cds
AT2G02010	3.152480448	1.11E-70	Arabidopsis thaliana glutamate decarboxylase 4 mRNA, complete cds
AT3G23550	2.792423389	4.16E-70	Arabidopsis thaliana MATE efflux family protein LAL5 mRNA, complete cds
AT5G52750	2.080780575	5.16E-70	Arabidopsis thaliana heavy metal transport/detoxification domain-containing protein mRNA, complete cds
AT4G26910	1.057749606	6.10E-70	Arabidopsis thaliana Dihydrolipoamide succinyltransferase mRNA, complete cds
AT3G51660	2.429127216	8.43E-70	Arabidopsis thaliana Tautomerase/MIF superfamily protein mRNA, complete cds
AT1G54040	2.042509495	8.57E-70	Arabidopsis thaliana epithiospecifier protein mRNA, complete cds

AT3G12145	1.106006981	8.80E-70	Arabidopsis thaliana leucine-rich repeat protein FLOR1 mRNA, complete cds
AT5G52320	1.986308852	1.99E-69	Arabidopsis thaliana chromosome 5 sequence
AT4G16860	1.087616146	2.61E-69	Arabidopsis thaliana protein RECOGNITION OF PERONOSPORA PARASITICA 4 mRNA, complete cds
AT5G42900	3.17542706	4.79E-69	Arabidopsis thaliana cold regulated protein 27 mRNA, complete cds
AT1G74640	1.473648056	1.58E-68	Arabidopsis thaliana alpha/beta-Hydrolases superfamily protein mRNA, complete cds
AT3G22600	1.250681999	1.80E-68	Arabidopsis thaliana protease inhibitor/seed storage/lipid transfer protein (LTP) family protein mRNA, complete cds
AT3G04210	1.041750606	4.16E-68	Arabidopsis thaliana TIR-NBS class disease resistance protein mRNA, complete cds
AT4G17500	2.471923164	5.99E-68	Arabidopsis thaliana chromosome 4 sequence
AT2G04080	2.319241847	8.05E-68	Arabidopsis thaliana MATE efflux family protein mRNA, complete cds
AT1G23080	1.044126322	8.37E-68	Arabidopsis thaliana auxin efflux carrier component 7 mRNA, complete cds
AT5G05410	2.700361144	1.33E-67	Arabidopsis thaliana dehydration-responsive element-binding protein 2A mRNA, complete cds
AT3G17860	1.578628476	7.09E-67	Arabidopsis thaliana jasmonate-zim-domain protein 3 mRNA, complete cds
AT1G33970	2.017931783	9.68E-67	Arabidopsis thaliana AIG1 domain-containing protein mRNA, complete cds
AT5G05250	1.903454701	1.37E-66	Arabidopsis thaliana chromosome 5 sequence
AT4G26130	1.127853103	1.75E-65	Arabidopsis thaliana chromosome 4 sequence
AT5G19110	2.3289244	2.18E-65	Arabidopsis thaliana Eukaryotic aspartyl protease family protein mRNA, complete cds
AT1G69870	1.242592289	2.70E-65	Arabidopsis thaliana nitrate transporter 1.7 mRNA, complete cds
AT1G53540	4.519607962	8.04E-64	Arabidopsis thaliana chromosome 1 sequence
AT5G13220	3.309889371	9.04E-64	Arabidopsis thaliana protein TIFY 9 mRNA, complete cds
AT1G15125	2.028028908	1.13E-63	Arabidopsis thaliana S-adenosyl-L-methionine-dependent methyltransferase mRNA, complete cds
AT2G23010	3.137175128	1.72E-63	Arabidopsis thaliana serine carboxypeptidase-like 9 mRNA, complete cds
AT2G40000	1.019514905	2.45E-63	Arabidopsis thaliana chromosome 2, complete sequence
AT1G19020	1.694835987	3.08E-63	Arabidopsis thaliana chromosome 1 sequence
AT1G55920	1.241269136	5.27E-63	Arabidopsis thaliana chromosome 1 sequence
AT1G72900	1.785809647	8.26E-63	Arabidopsis thaliana Toll-Interleukin-Resistance domain-containing protein mRNA, complete cds
AT4G23010	1.364739535	1.94E-62	Arabidopsis thaliana UDP-galactose transporter 2 mRNA, complete cds

AT2G30770	2.876509291	3.23E-62	Arabidopsis thaliana cytochrome P450, family 71, subfamily A, polypeptide 13 mRNA, complete cds
AT2G46400	2.587862178	1.16E-61	Arabidopsis thaliana putative WRKY transcription factor 46 mRNA, complete cds
AT5G12030	2.666892507	1.86E-61	Arabidopsis thaliana chromosome 5 sequence
AT1G51090	4.356996769	8.03E-61	Arabidopsis thaliana uncharacterized protein mRNA, complete cds
AT5G25350	1.102066126	1.18E-60	Arabidopsis thaliana EIN3-binding F-box protein 2 mRNA, complete cds
AT4G18880	1.702057273	1.32E-60	Arabidopsis thaliana heat stress transcription factor A-4a mRNA, complete cds
AT3G05950	1.064128519	2.27E-60	Arabidopsis thaliana mRNA for germin-like protein, complete cds, clone: RAFL16-44-D15
AT4G19120	1.169778368	4.93E-60	Arabidopsis thaliana putative methyltransferase PMT21 mRNA, complete cds
AT1G03740	1.208878074	6.56E-60	Arabidopsis thaliana protein kinase mRNA, complete cds
AT2G46790	3.004349911	6.92E-60	Arabidopsis thaliana two-component response regulator-like APRR9 mRNA, complete cds
AT3G01830	4.95828102	9.78E-60	Arabidopsis thaliana chromosome 3, complete sequence
AT1G58270	1.173226823	2.35E-59	Arabidopsis thaliana protein ZW9 mRNA, complete cds
AT4G36010	2.619143636	2.70E-59	Arabidopsis thaliana pathogenesis-related thaumatin family protein mRNA, complete cds
AT1G23020	1.325712343	2.78E-59	Arabidopsis thaliana ferric reduction oxidase 3 mRNA, complete cds
AT2G41240	5.122128834	4.63E-59	Arabidopsis thaliana transcription factor bHLH100 mRNA, complete cds
AT4G24160	1.463432493	9.82E-59	Arabidopsis thaliana lysophosphatidic acid acyltransferase mRNA, complete cds
AT5G64770	1.213512657	1.48E-58	Arabidopsis thaliana root meristem growth factor 9 mRNA, complete cds
AT3G55970	5.894150683	2.03E-58	Arabidopsis thaliana jasmonate-regulated protein JRG21 mRNA, complete cds
AT4G39730	1.158087055	3.20E-58	Arabidopsis thaliana Lipase/lipooxygenase, PLAT/LH2 family protein mRNA, complete cds
AT2G40140	1.083109202	3.46E-58	Arabidopsis thaliana zinc finger CCCH domain-containing protein 29 mRNA, complete cds
AT4G21390	2.272447635	3.58E-58	Arabidopsis thaliana G-type lectin S-receptor-like serine/threonine-protein kinase B120 mRNA, complete cds
AT1G78600	1.980581745	8.55E-58	Arabidopsis thaliana light-regulated zinc finger protein 1 mRNA, complete cds
AT5G37940	8.057436487	8.76E-58	Arabidopsis thaliana zinc-binding dehydrogenase family protein mRNA, complete cds
AT5G64230	3.024046758	6.40E-57	Arabidopsis thaliana uncharacterized protein mRNA,

			complete cds
AT4G23140	2.142240881	8.08E-57	Arabidopsis thaliana cysteine-rich receptor-like protein kinase 6 mRNA, complete cds
AT1G64200	1.192385567	6.01E-55	Arabidopsis thaliana V-type proton ATPase subunit E3 mRNA, complete cds
AT3G17120	1.427242804	1.42E-54	Arabidopsis thaliana uncharacterized protein mRNA, complete cds
AT1G62430	1.2119345	1.77E-54	Arabidopsis thaliana phosphatidate cytidylyltransferase mRNA, complete cds
AT1G14880	1.854064405	2.73E-54	Arabidopsis thaliana cadmium resistance protein 1 mRNA, complete cds
AT1G05675	2.125228073	3.90E-54	Arabidopsis thaliana UDP-Glycosyltransferase superfamily protein mRNA, complete cds
AT3G09830	1.338556336	1.37E-53	Arabidopsis thaliana protein kinase family protein mRNA, complete cds
AT1G68050	4.80817746	1.61E-53	Arabidopsis thaliana flavin-binding, kelch repeat, f box 1 mRNA, complete cds
AT1G13609	4.016764082	3.03E-53	Arabidopsis thaliana defensin-like protein 287 mRNA, complete cds
AT5G22630	1.314937311	4.25E-53	Arabidopsis thaliana chromosome 5 sequence
AT3G50910	1.042033184	4.33E-53	Arabidopsis thaliana uncharacterized protein mRNA, complete cds
AT1G54010	1.247200003	4.39E-53	Arabidopsis thaliana GDSL esterase/lipase mRNA, complete cds
AT4G39670	3.053289958	6.90E-53	Arabidopsis thaliana chromosome 4 sequence
AT2G38360	1.510735991	4.44E-52	Arabidopsis thaliana chromosome 2, complete sequence
AT2G30140	1.742310274	5.76E-52	Arabidopsis thaliana UDP-glycosyltransferase 87A2 mRNA, complete cds
AT1G17745	1.03589421	5.34E-51	Arabidopsis thaliana D-3-phosphoglycerate dehydrogenase mRNA, complete cds
AT3G05030	1.432817027	5.46E-51	Arabidopsis thaliana K+/H+ exchanger mRNA, complete cds
AT3G15210	1.663343439	5.71E-51	Arabidopsis thaliana chromosome 3, complete sequence
AT4G15920	1.065601316	7.68E-51	Arabidopsis thaliana bidirectional sugar transporter SWEET17 mRNA, complete cds
AT1G45145	1.465897375	1.75E-50	Arabidopsis thaliana thioredoxin H5 mRNA, complete cds
AT5G52810	1.472432068	2.88E-50	Arabidopsis thaliana chromosome 5 sequence
AT4G29700	1.438628809	4.76E-50	Arabidopsis thaliana chromosome 4 sequence
AT2G40750	1.851628978	5.61E-50	Arabidopsis thaliana WRKY DNA-binding protein 54 mRNA, complete cds
AT5G27420	1.785316502	6.00E-50	Arabidopsis thaliana chromosome 5 sequence
AT1G62510	3.109713766	7.79E-50	Arabidopsis thaliana chromosome 1 sequence
AT3G25610	1.439641263	8.99E-50	Arabidopsis thaliana ATPase E1-E2 type family protein /

			haloacid dehalogenase-like hydrolase family protein mRNA, complete cds
AT1G18150	1.115168821	9.12E-50	Arabidopsis thaliana mitogen-activated protein kinase 8 mRNA, complete cds
AT1G13340	2.675727164	1.31E-49	Arabidopsis thaliana Regulator of Vps4 activity in the MVB pathway protein mRNA, complete cds
AT2G15490	5.647712788	1.58E-49	Arabidopsis thaliana mRNA for putative glucosyltransferase, complete cds, clone: RAFL14-26-J02
AT2G04050	3.718251781	1.69E-49	Arabidopsis thaliana MATE efflux family protein mRNA, complete cds
AT5G18470	1.612717366	2.22E-49	Arabidopsis thaliana chromosome 5 sequence
AT5G41750	1.560249946	2.75E-49	Arabidopsis thaliana TIR-NBS-LRR class disease resistance protein mRNA, complete cds
AT2G31865	1.771895986	5.24E-49	Arabidopsis thaliana poly(ADP-ribose) glycohydrolase 2 mRNA, complete cds
AT5G13750	1.521255815	2.08E-48	Arabidopsis thaliana zinc induced facilitator-like 1 protein mRNA, complete cds
AT5G39050	1.342841839	4.45E-48	Arabidopsis thaliana chromosome 5 sequence
AT2G01180	1.476026039	4.56E-48	Arabidopsis thaliana chromosome 2, complete sequence
AT4G14230	1.440853851	4.58E-48	Arabidopsis thaliana CBS domain-containing protein with a domain of unknown function (DUF21) mRNA, complete cds
AT1G73325	5.289983971	4.88E-48	Arabidopsis thaliana chromosome 1 sequence
AT2G47890	1.88518053	5.42E-48	Arabidopsis thaliana zinc finger protein CONSTANS-LIKE 13 mRNA, complete cds
AT2G44080	2.154190043	7.77E-48	Arabidopsis thaliana ARGOS-like protein mRNA, complete cds
AT5G36220	1.972092107	8.64E-48	Arabidopsis thaliana cytochrome P450 81D1 mRNA, complete cds
AT1G76520	1.542074021	1.82E-47	Arabidopsis thaliana auxin efflux carrier family protein mRNA, complete cds
AT5G60100	2.709113332	2.07E-47	Arabidopsis thaliana pseudo-response regulator 3 mRNA, complete cds
AT1G28190	1.991737967	2.33E-47	Arabidopsis thaliana chromosome 1 sequence
AT4G16880	2.621450103	7.86E-47	Arabidopsis thaliana leucine-rich repeat (LRR) family protein mRNA, complete cds
AT5G27760	1.366636942	8.41E-47	Arabidopsis thaliana hypoxia-responsive family protein mRNA, complete cds
AT4G26080	1.064387667	1.23E-46	Arabidopsis thaliana protein phosphatase 2C 56 mRNA, complete cds
AT1G54100	1.008036028	1.44E-46	Arabidopsis thaliana aldehyde dehydrogenase 7B4 mRNA, complete cds
AT1G18300	2.08138887	1.57E-46	Arabidopsis thaliana nudix hydrolase 4 mRNA, complete cds

AT5G27060	5.238321852	2.47E-46	Arabidopsis thaliana chromosome 5 sequence
AT2G04400	1.01768603	3.52E-46	Arabidopsis thaliana indole-3-glycerol phosphate synthase mRNA, complete cds
AT2G05940	1.180885846	1.28E-45	Arabidopsis thaliana RPM1-induced protein kinase mRNA, complete cds
AT1G07000	1.19284131	1.91E-45	Arabidopsis thaliana exocyst subunit exo70 family protein B2 mRNA, complete cds
AT3G14050	1.120140425	2.72E-45	Arabidopsis thaliana RelA-SpoT like protein RSH2 mRNA, complete cds
AT4G03400	1.555384191	3.48E-45	Arabidopsis thaliana auxin-responsive GH3 family protein mRNA, complete cds
AT1G13990	1.607044158	3.85E-45	Arabidopsis thaliana uncharacterized protein mRNA, complete cds
AT4G33920	1.17804384	6.37E-45	Arabidopsis thaliana putative protein phosphatase 2C 63 mRNA, complete cds
AT1G22070	1.395927651	1.02E-44	Arabidopsis thaliana transcription factor TGA3 mRNA, complete cds
AT5G64860	1.219574222	2.28E-44	Arabidopsis thaliana 4-alpha-glucanotransferase DPE1 mRNA, complete cds
AT2G30250	1.153205238	1.04E-43	Arabidopsis thaliana WRKY transcription factor 25 mRNA, complete cds
AT3G48650	2.072148985	1.15E-43	Arabidopsis thaliana chromosome 3, complete sequence
AT5G48850	3.33603479	6.68E-43	Arabidopsis thaliana protein SULPHUR DEFICIENCY-INDUCED 1 mRNA, complete cds
AT1G76590	2.231627199	7.50E-43	Arabidopsis thaliana PLATZ transcription factor family protein mRNA, complete cds
AT5G52760	2.650282147	1.05E-42	Arabidopsis thaliana copper transport family protein mRNA, complete cds
AT2G40610	1.098345842	1.21E-42	Arabidopsis thaliana expansin A8 mRNA, complete cds
AT5G38120	2.887824604	1.50E-42	Arabidopsis thaliana 4-coumarate--CoA ligase-like 8 mRNA, complete cds
AT2G18193	2.601472609	2.59E-42	Arabidopsis thaliana P-loop containing nucleoside triphosphate hydrolases superfamily protein mRNA, complete cds
AT2G43620	2.068658944	1.28E-41	Arabidopsis thaliana chitinase family protein mRNA, complete cds
AT2G44500	1.206770603	2.02E-41	Arabidopsis thaliana O-fucosyltransferase family protein mRNA, complete cds
AT4G27280	1.52197993	2.09E-41	Arabidopsis thaliana chromosome 4 sequence
AT5G22250	2.641779832	2.11E-41	Arabidopsis thaliana chromosome 5 sequence
AT2G15390	1.65101541	4.26E-41	Arabidopsis thaliana probable fucosyltransferase 4 mRNA, complete cds
AT4G11890	2.153624687	4.46E-41	Arabidopsis thaliana receptor-like cytosolic kinase ARCK1 mRNA, complete cds

AT4G33625	1.18351833	6.10E-41	Arabidopsis thaliana uncharacterized protein mRNA, complete cds
AT1G75270	1.369566385	8.03E-41	Arabidopsis thaliana glutathione S-transferase DHAR2 mRNA, complete cds
AT5G44420	4.80817746	1.39E-40	Arabidopsis thaliana ethylene- and jasmonate-responsive plant defensin mRNA, complete cds
AT3G44190	1.035994705	1.59E-40	Arabidopsis thaliana FAD/NAD(P)-binding oxidoreductase family protein mRNA, complete cds
AT3G46080	6.350326877	2.79E-40	Arabidopsis thaliana chromosome 3, complete sequence
AT5G13320	2.34057191	3.18E-40	Arabidopsis thaliana 4-substituted benzoates-glutamate ligase GH3.12 mRNA, complete cds
AT4G38080	1.031522679	3.26E-40	Arabidopsis thaliana chromosome 4 sequence
AT4G27830	1.144782626	3.39E-40	Arabidopsis thaliana beta glucosidase 10 mRNA, complete cds
AT3G48850	5.34160966	3.45E-40	Arabidopsis thaliana phosphate transporter 3;2 mRNA, complete cds
AT2G34500	2.199151254	4.04E-40	Arabidopsis thaliana chromosome 2, complete sequence
AT5G38510	1.11411515	5.32E-40	Arabidopsis thaliana rhomboid-related intramembrane serine protease-like protein mRNA, complete cds
AT5G13490	1.232492773	6.27E-40	Arabidopsis thaliana ADP/ATP carrier protein 2 mRNA, complete cds
AT2G29310	1.48911677	7.15E-40	Arabidopsis thaliana tropinone reductase-like protein mRNA, complete cds
AT3G10260	1.117826559	1.01E-39	Arabidopsis thaliana reticulon-like protein B8 mRNA, complete cds
AT5G18400	1.053628897	1.19E-39	Arabidopsis thaliana anamorsin homolog mRNA, complete cds
AT2G31230	2.870105209	1.90E-39	Arabidopsis thaliana chromosome 2, complete sequence
AT1G14580	1.55464724	1.96E-39	Arabidopsis thaliana C2H2-like zinc finger protein mRNA, complete cds
AT2G46440	1.178765007	2.40E-39	Arabidopsis thaliana cyclic nucleotide-gated channel 11 mRNA, complete cds
AT2G30750	1.955973971	3.24E-39	Arabidopsis thaliana cytochrome P450 71A12 mRNA, complete cds
AT2G33380	1.18956254	3.65E-39	Arabidopsis thaliana caleosin 3 mRNA, complete cds
AT2G26530	1.565997743	5.06E-39	Arabidopsis thaliana chromosome 2, complete sequence
AT2G32150	1.071392214	8.38E-39	Arabidopsis thaliana haloacid dehalogenase-like hydrolase domain-containing protein mRNA, complete cds
AT1G15430	1.708970456	1.29E-38	Arabidopsis thaliana chromosome 1 sequence
AT1G07160	3.874140722	3.16E-38	Arabidopsis thaliana putative protein phosphatase 2C 2 mRNA, complete cds
AT5G41740	1.155369062	3.30E-38	Arabidopsis thaliana TIR-NBS-LRR class disease resistance protein mRNA, complete cds

AT3G11340	1.288445331	5.61E-38	Arabidopsis thaliana UDP-dependent glycosyltransferase 76B1 mRNA, complete cds
AT2G30600	1.005219952	7.22E-38	Arabidopsis thaliana BTB/POZ domain-containing protein mRNA, complete cds
AT4G20860	1.098792399	8.01E-38	Arabidopsis thaliana chromosome 4 sequence
AT4G34131	2.161383738	8.30E-38	Arabidopsis thaliana chromosome 4 sequence
AT3G12500	1.827730257	1.06E-37	Arabidopsis thaliana basic chitinase B mRNA, complete cds
AT3G49570	4.507782527	1.24E-37	Arabidopsis thaliana chromosome 3, complete sequence
AT5G24660	2.307304358	1.87E-37	Arabidopsis thaliana chromosome 5 sequence
AT2G28720	2.16394901	2.12E-37	Arabidopsis thaliana chromosome 2, complete sequence
AT1G56060	5.232675288	2.87E-37	Arabidopsis thaliana uncharacterized protein mRNA, complete cds
AT1G73500	1.763134209	3.44E-37	Arabidopsis thaliana chromosome 1 sequence
AT1G28480	4.489860619	3.99E-37	Arabidopsis thaliana chromosome 1 sequence
AT5G23990	3.514446257	4.41E-37	Arabidopsis thaliana ferric reduction oxidase 5 mRNA, complete cds
AT1G28230	2.722521375	5.34E-37	Arabidopsis thaliana purine permease 1 mRNA, complete cds
AT4G26670	1.16216558	6.59E-37	Arabidopsis thaliana mitochondrial import inner membrane translocase subunit TIM22-2 mRNA, complete cds
AT1G71140	2.666700888	8.15E-37	Arabidopsis thaliana MATE efflux family protein mRNA, complete cds
AT3G05640	1.640981219	1.04E-36	Arabidopsis thaliana putative protein phosphatase 2C mRNA, complete cds
AT2G29500	2.560249946	2.87E-36	Arabidopsis thaliana chromosome 2, complete sequence
AT2G30550	1.057053349	4.21E-36	Arabidopsis thaliana phospholipase A1-Igamma2 mRNA, complete cds
AT2G35980	3.799715881	6.08E-36	Arabidopsis thaliana chromosome 2, complete sequence
AT4G04490	2.242391516	7.03E-36	Arabidopsis thaliana cysteine-rich receptor-like protein kinase 36 mRNA, complete cds
AT2G39420	1.137558875	7.39E-36	Arabidopsis thaliana alpha/beta-Hydrolases superfamily protein mRNA, complete cds
AT1G47395	2.934645461	9.41E-36	Arabidopsis thaliana chromosome 1 sequence
AT3G44260	1.820168379	1.16E-35	Arabidopsis thaliana chromosome 3, complete sequence
AT4G17490	1.810123104	1.70E-35	Arabidopsis thaliana chromosome 4 sequence
AT4G22530	2.21814397	2.00E-35	Arabidopsis thaliana S-adenosyl-L-methionine-dependent methyltransferase domain-containing protein mRNA, complete cds
AT1G66700	4.1182454	2.17E-35	Arabidopsis thaliana SABATH family methyltransferase PXMT1 mRNA, complete cds
AT1G70210	1.05560661	3.86E-35	Arabidopsis thaliana cyclin-D1-1 mRNA, complete cds

AT5G10695	2.530624777	3.97E-35	Arabidopsis thaliana chromosome 5 sequence
AT1G14360	1.015088454	5.52E-35	Arabidopsis thaliana UDP-galactose transporter 3 mRNA, complete cds
AT3G55240	3.765364376	6.28E-35	Arabidopsis thaliana uncharacterized protein mRNA, complete cds
AT3G44300	1.535375278	7.33E-35	Arabidopsis thaliana nitrilase 2 mRNA, complete cds
AT3G46230	2.718795886	7.55E-35	Arabidopsis thaliana chromosome 3, complete sequence
AT3G28340	2.231048617	7.87E-35	Arabidopsis thaliana chromosome 3, complete sequence
AT3G10300	1.141734276	7.95E-35	Arabidopsis thaliana putative calcium-binding protein CML49 mRNA, complete cds
AT1G24070	1.489860619	8.44E-35	Arabidopsis thaliana cellulose synthase-like A10 mRNA, complete cds
AT1G30755	1.26142055	1.03E-34	Arabidopsis thaliana uncharacterized protein mRNA, complete cds
AT5G61560	1.456049127	1.28E-34	Arabidopsis thaliana U-box domain-containing protein 51 mRNA, complete cds
AT1G78210	1.128601484	1.28E-34	Arabidopsis thaliana alpha/beta-Hydrolases superfamily protein mRNA, complete cds
AT4G30260	1.013985959	1.96E-34	Arabidopsis thaliana Yip1 integral membrane domain-containing protein mRNA, complete cds
AT4G12290	1.006959666	2.76E-34	Arabidopsis thaliana copper amine oxidase family protein mRNA, complete cds
AT3G10020	1.017999122	3.41E-34	Arabidopsis thaliana chromosome 3, complete sequence
AT1G55260	1.030406344	1.09E-33	Arabidopsis thaliana Bifunctional inhibitor/lipid-transfer protein/seed storage 2S albumin superfamily protein mRNA, complete cds
AT1G54820	1.109252541	1.14E-33	Arabidopsis thaliana putative serine/threonine protein kinase mRNA, complete cds
AT1G24150	1.18958928	1.65E-33	Arabidopsis thaliana formin-like protein 4 mRNA, complete cds
AT2G04070	3.623575882	1.94E-33	Arabidopsis thaliana MATE efflux family protein mRNA, complete cds
AT4G23150	3.920145892	2.28E-33	Arabidopsis thaliana cysteine-rich receptor-like protein kinase 7 mRNA, complete cds
AT4G30280	1.219456809	2.61E-33	Arabidopsis thaliana mRNA for hypothetical protein, complete cds, clone: RAFL24-01-A19
AT3G05880	1.070787606	3.29E-33	Arabidopsis thaliana Hydrophobic protein RCI2A mRNA, complete cds
AT5G12020	2.357336533	5.17E-33	Arabidopsis thaliana chromosome 5 sequence
AT1G51800	1.099734817	6.30E-33	Arabidopsis thaliana putative leucine-rich repeat protein kinase mRNA, complete cds
AT2G02230	1.458861893	6.54E-33	Arabidopsis thaliana F-box protein PP2-B1 mRNA, complete cds
AT2G43590	1.394177271	6.54E-33	Arabidopsis thaliana chitinase family protein mRNA,

			complete cds
AT1G01500	2.30297481	6.71E-33	Arabidopsis thaliana Erythronate-4-phosphate dehydrogenase-like protein mRNA, complete cds
AT3G10940	1.062750287	7.97E-33	Arabidopsis thaliana phosphoglucan phosphatase LSF2 mRNA, complete cds
AT5G59320	1.876639858	8.50E-33	Arabidopsis thaliana non-specific lipid-transfer protein 3 mRNA, complete cds
AT1G04770	1.652613965	9.81E-33	Arabidopsis thaliana tetratricopeptide repeat-containing protein mRNA, complete cds
AT4G02370	1.195388056	1.27E-32	Arabidopsis thaliana chromosome 4 sequence
AT1G67810	2.201355525	1.60E-32	Arabidopsis thaliana chromosome 1 sequence
AT1G64160	4.740822192	1.78E-32	Arabidopsis thaliana chromosome 1 sequence
AT3G45040	1.099898887	2.25E-32	Arabidopsis thaliana putative dolichol kinase mRNA, complete cds
AT4G09760	1.223515052	2.36E-32	Arabidopsis thaliana choline synthase mRNA, complete cds
AT3G09010	1.754073216	3.16E-32	Arabidopsis thaliana protein kinase mRNA, complete cds
AT5G02780	3.074823119	3.18E-32	Arabidopsis thaliana glutathione transferase lambda 1 mRNA, complete cds
AT2G30020	1.211301638	5.68E-32	Arabidopsis thaliana putative protein phosphatase 2C 25 mRNA, complete cds
AT1G27930	1.236333298	9.02E-32	Arabidopsis thaliana chromosome 1 sequence
AT5G61270	1.5294439	1.37E-31	Arabidopsis thaliana transcription factor PIF7 mRNA, complete cds
AT1G52890	3.399313728	1.61E-31	Arabidopsis thaliana NAC domain-containing protein 19 mRNA, complete cds
AT5G64810	4.104570463	2.63E-31	Arabidopsis thaliana putative WRKY transcription factor 51 mRNA, complete cds
AT1G27330	1.061751536	4.98E-31	Arabidopsis thaliana ribosome associated membrane protein RAMP4 mRNA, complete cds
AT1G02850	2.148021981	5.04E-31	Arabidopsis thaliana beta glucosidase 11 mRNA, complete cds
AT5G65140	1.322953102	5.05E-31	Arabidopsis thaliana probable trehalose-phosphate phosphatase J mRNA, complete cds
AT5G66070	1.627919773	8.13E-31	Arabidopsis thaliana RING/U-box superfamily protein mRNA, complete cds
AT2G04040	3.944913797	8.25E-31	Arabidopsis thaliana MATE efflux family protein DTX1 mRNA, complete cds
AT3G49580	4.434719064	9.21E-31	Arabidopsis thaliana protein RESPONSE TO LOW SULFUR 1 mRNA, complete cds
AT2G36220	1.552945146	1.40E-30	Arabidopsis thaliana chromosome 2, complete sequence
AT5G33290	1.437994196	1.53E-30	Arabidopsis thaliana xylogalacturonan beta-1,3-xylosyltransferase mRNA, complete cds
AT1G54050	1.08480093	1.79E-30	Arabidopsis thaliana CIII heat shock protein 17.4 mRNA,

			complete cds
AT2G45560	1.120380594	2.02E-30	Arabidopsis thaliana cytochrome P450 76C1 mRNA, complete cds
AT3G43270	1.339122492	2.11E-30	Arabidopsis thaliana probable pectinesterase/pectinesterase inhibitor 32 mRNA, complete cds
AT4G01360	4.642044038	3.27E-30	Arabidopsis thaliana BYPASS1-related protein mRNA, complete cds
AT1G67970	1.911375682	7.63E-30	Arabidopsis thaliana heat stress transcription factor A-8 mRNA, complete cds
AT5G52050	2.036926512	9.63E-30	Arabidopsis thaliana chromosome 5 sequence
AT2G14247	4.619143636	1.04E-29	Arabidopsis thaliana chromosome 2, complete sequence
AT1G70420	2.181738323	1.27E-29	Arabidopsis thaliana chromosome 1 sequence
AT4G21840	3.325784693	1.60E-29	Arabidopsis thaliana methionine sulfoxide reductase B8 mRNA, complete cds
AT1G80110	2.849756564	2.06E-29	Arabidopsis thaliana phloem protein 2-B11 mRNA, complete cds
AT3G63380	1.873407832	4.29E-29	Arabidopsis thaliana chromosome 3, complete sequence
AT3G22160	2.026715685	6.34E-29	Arabidopsis thaliana chromosome 3, complete sequence
AT5G60900	1.321825008	6.54E-29	Arabidopsis thaliana receptor-like protein kinase 1 mRNA, complete cds
AT3G26840	1.318771684	9.33E-29	Arabidopsis thaliana phytyl ester synthesis and diacylglycerol acyltransferase activity protein mRNA, complete cds
AT1G43160	2.824953173	1.06E-28	Arabidopsis thaliana ethylene-responsive transcription factor RAP2-6 mRNA, complete cds
AT4G37470	1.071046429	1.21E-28	Arabidopsis thaliana probable esterase KAI2 mRNA, complete cds
AT3G55880	1.430369664	1.47E-28	Arabidopsis thaliana protein SULPHATE UTILIZATION EFFICIENCY 4 mRNA, complete cds
AT2G27310	1.465785262	1.50E-28	Arabidopsis thaliana chromosome 2, complete sequence
AT2G23840	1.620192487	1.64E-28	Arabidopsis thaliana HNH endonuclease mRNA, complete cds
AT3G50280	2.774374752	2.36E-28	Arabidopsis thaliana chromosome 3, complete sequence
AT5G11610	3.090764663	2.81E-28	Arabidopsis thaliana Exostosin family protein mRNA, complete cds
AT5G62360	2.221598494	3.14E-28	Arabidopsis thaliana chromosome 5 sequence
AT4G29510	1.001685136	3.63E-28	Arabidopsis thaliana arginine methyltransferase 11 mRNA, complete cds
AT5G54100	1.33283945	5.61E-28	Arabidopsis thaliana SPFH/Band 7/PHB domain-containing membrane-associated protein mRNA, complete cds
AT5G18130	1.244696494	7.66E-28	Arabidopsis thaliana chromosome 5 sequence
AT1G75900	1.389183121	7.85E-28	Arabidopsis thaliana GDSL esterase/lipase EXL3 mRNA,

			complete cds
AT1G18360	1.089982926	9.07E-28	Arabidopsis thaliana alpha/beta-Hydrolases superfamily protein mRNA, complete cds
AT1G14200	1.206011142	1.14E-27	Arabidopsis thaliana chromosome 1 sequence
AT5G28237	4.80817746	1.28E-27	Arabidopsis thaliana tryptophan synthase beta chain-like protein mRNA, complete cds
AT3G53800	1.17212918	1.79E-27	Arabidopsis thaliana hsp70-interacting protein FES1B-like protein mRNA, complete cds
AT1G32960	4.285142708	1.85E-27	Arabidopsis thaliana Subtilase family protein SBT3.3 mRNA, complete cds
AT1G23850	1.572849983	1.86E-27	Arabidopsis thaliana chromosome 1 sequence
AT2G06925	1.437467928	1.89E-27	Arabidopsis thaliana phospholipase A2-alpha mRNA, complete cds
AT1G76955	4.511340346	1.97E-27	Arabidopsis thaliana uncharacterized protein mRNA, complete cds
AT5G54860	1.144568531	2.20E-27	Arabidopsis thaliana probable folate-biopterin transporter 4 mRNA, complete cds
AT1G72120	1.920425511	3.72E-27	Arabidopsis thaliana putative peptide/nitrate transporter mRNA, complete cds
AT1G55110	1.132433842	4.89E-27	Arabidopsis thaliana indeterminate(ID)-domain 7 protein mRNA, complete cds
AT1G26380	1.8088263	5.40E-27	Arabidopsis thaliana chromosome 1 sequence
AT4G11650	1.209244619	8.89E-27	Arabidopsis thaliana osmotin-like protein OSM34 mRNA, complete cds
AT1G21670	1.273883037	1.10E-26	Arabidopsis thaliana chromosome 1 sequence
AT1G69690	1.042944396	1.15E-26	Arabidopsis thaliana chromosome 1 sequence
AT1G16850	2.675727164	1.29E-26	Arabidopsis thaliana uncharacterized protein mRNA, complete cds
AT2G32210	4.235814996	1.93E-26	Arabidopsis thaliana uncharacterized protein mRNA, complete cds
AT5G57630	1.204469033	2.02E-26	Arabidopsis thaliana CBL-interacting serine/threonine-protein kinase 21 mRNA, complete cds
AT2G44940	1.308843543	2.13E-26	Arabidopsis thaliana chromosome 2, complete sequence
AT1G21400	1.626052005	2.70E-26	Arabidopsis thaliana thiamin diphosphate-binding fold protein mRNA, complete cds
AT4G12900	1.957439444	4.36E-26	Arabidopsis thaliana Gamma interferon responsive lysosomal thiol (GILT) reductase family protein mRNA, complete cds
AT1G73470	1.101519857	6.94E-26	Arabidopsis thaliana uncharacterized protein mRNA, complete cds
AT1G09080	2.936813298	1.04E-25	Arabidopsis thaliana protein BIP3 mRNA, complete cds
AT4G21680	2.312322433	1.13E-25	Arabidopsis thaliana nitrate transporter 1.8 mRNA, complete cds
AT3G47340	1.272831382	1.45E-25	Arabidopsis thaliana asparagine synthetase

			[glutamine-hydrolyzing] mRNA, complete cds
AT4G38580	1.312868805	1.97E-25	Arabidopsis thaliana farnesylated protein 6 mRNA, complete cds
AT1G10585	4.184740811	2.02E-25	Arabidopsis thaliana basic helix-loop-helix domain-containing protein mRNA, complete cds
AT4G08555	5.633498928	2.24E-25	Arabidopsis thaliana chromosome 4 sequence
AT1G76070	1.969055492	2.95E-25	Arabidopsis thaliana chromosome 1 sequence
AT1G21240	2.737127709	2.96E-25	Arabidopsis thaliana wall-associated receptor kinase 3 mRNA, complete cds
AT3G16720	1.167932524	3.44E-25	Arabidopsis thaliana chromosome 3, complete sequence
AT1G57750	5.619143636	3.88E-25	Arabidopsis thaliana chromosome 1 sequence
AT4G27310	1.277177787	4.31E-25	Arabidopsis thaliana B-box domain protein 28 mRNA, complete cds
AT1G24148	1.307733413	4.62E-25	Arabidopsis thaliana chromosome 1 sequence
AT1G76650	2.141645832	1.19E-24	Arabidopsis thaliana chromosome 1 sequence
AT5G25930	1.065985756	1.67E-24	Arabidopsis thaliana protein kinase family protein with leucine-rich repeat domain mRNA, complete cds
AT1G52070	1.503836638	1.76E-24	Arabidopsis thaliana jacalin-like lectin domain-containing protein mRNA, complete cds
AT2G32680	1.417597875	1.78E-24	Arabidopsis thaliana chromosome 2, complete sequence
AT2G46170	1.024869475	2.20E-24	Arabidopsis thaliana reticulon-like protein B5 mRNA, complete cds
AT5G61810	1.273255642	2.21E-24	Arabidopsis thaliana ATP-Mg/Pi transporter mRNA, complete cds
AT5G46330	1.465001631	2.37E-24	Arabidopsis thaliana LRR receptor-like serine/threonine-protein kinase FLS2 mRNA, complete cds
AT1G18970	1.475610133	3.45E-24	Arabidopsis thaliana chromosome 1 sequence
AT4G23215	3.782642368	3.54E-24	Arabidopsis thaliana chromosome 4 sequence
AT3G57240	4.619143636	4.28E-24	Arabidopsis thaliana beta-1,3-glucanase 3 mRNA, complete cds
AT5G66400	3.101819852	4.40E-24	Arabidopsis thaliana dehydrin Rab18 mRNA, complete cds
AT5G62480	1.837783922	5.23E-24	Arabidopsis thaliana glutathione S-transferase tau 9 mRNA, complete cds
AT4G25810	1.506810688	5.51E-24	Arabidopsis thaliana probable xyloglucan endotransglucosylase/hydrolase protein 23 mRNA, complete cds
AT5G13370	1.229044038	5.58E-24	Arabidopsis thaliana auxin-responsive GH3 family protein mRNA, complete cds
AT4G26270	1.155055065	6.17E-24	Arabidopsis thaliana 6-phosphofructokinase 3 mRNA, complete cds
AT3G23150	1.386311883	6.65E-24	Arabidopsis thaliana ethylene receptor 2 mRNA, complete cds

AT2G16700	1.07252217	7.30E-24	Arabidopsis thaliana actin depolymerizing factor 5 mRNA, complete cds
AT5G13080	2.856642949	8.70E-24	Arabidopsis thaliana putative WRKY transcription factor 75 mRNA, complete cds
AT5G59310	3.15661721	9.50E-24	Arabidopsis thaliana non-specific lipid-transfer protein 4 mRNA, complete cds
AT2G29420	1.234263635	1.03E-23	Arabidopsis thaliana glutathione S-transferase tau 7 mRNA, complete cds
AT3G55760	1.199847704	1.21E-23	Arabidopsis thaliana uncharacterized protein mRNA, complete cds
AT5G27520	1.195520144	1.45E-23	Arabidopsis thaliana peroxisomal adenine nucleotide carrier 2 mRNA, complete cds
AT1G47400	2.846301005	1.51E-23	Arabidopsis thaliana chromosome 1 sequence
AT5G14700	1.369108459	1.52E-23	Arabidopsis thaliana Rossmann-fold NAD(P)-binding domain-containing protein mRNA, complete cds
AT1G65890	2.524180692	1.54E-23	Arabidopsis thaliana acyl activating enzyme 12 mRNA, complete cds
AT1G19300	1.021416616	1.71E-23	Arabidopsis thaliana chromosome 1 sequence
AT4G04330	1.092644396	1.77E-23	Arabidopsis thaliana Chaperonin-like RbcX protein mRNA, complete cds
AT1G56650	2.152825631	2.00E-23	Arabidopsis thaliana transcription factor MYB75 mRNA, complete cds
AT5G58670	1.18696143	2.05E-23	Arabidopsis thaliana phosphoinositide phospholipase C 1 mRNA, complete cds
AT2G20670	1.158574469	3.01E-23	Arabidopsis thaliana uncharacterized protein mRNA, complete cds
AT4G35180	3.373836822	4.91E-23	Arabidopsis thaliana LYS/HIS transporter 7 mRNA, complete cds
AT1G24625	1.063440077	5.52E-23	Arabidopsis thaliana zinc finger protein 7 mRNA, complete cds
AT1G28370	3.473538313	5.56E-23	Arabidopsis thaliana chromosome 1 sequence
AT3G51430	1.108078201	5.64E-23	Arabidopsis thaliana strictosidine synthase-like 5 mRNA, complete cds
AT3G25010	1.436940304	6.46E-23	Arabidopsis thaliana chromosome 3, complete sequence
AT1G70810	1.239743927	7.99E-23	Arabidopsis thaliana Calcium-dependent lipid-binding (CaLB domain) family protein mRNA, complete cds
AT5G67080	4.913886901	9.53E-23	Arabidopsis thaliana chromosome 5 sequence
AT5G39790	1.42476559	1.20E-22	Arabidopsis thaliana putative starch binding scaffold protein mRNA, complete cds
AT3G24982	1.470052137	1.23E-22	Arabidopsis thaliana receptor like protein 40 mRNA, complete cds
AT1G08890	1.570554955	1.27E-22	Arabidopsis thaliana sugar transporter ERD6-like 1 mRNA, complete cds
AT1G57630	3.034181135	1.29E-22	Arabidopsis thaliana chromosome 1 sequence

AT5G57550	6.467140542	1.62E-22	Arabidopsis thaliana probable xyloglucan endotransglucosylase/hydrolase protein 25 mRNA, complete cds
AT1G65790	2.239990672	1.79E-22	Arabidopsis thaliana receptor-like serine/threonine-protein kinase SD1-7 mRNA, complete cds
AT3G23120	1.592311156	1.96E-22	Arabidopsis thaliana chromosome 3, complete sequence
AT1G14370	1.150061847	2.41E-22	Arabidopsis thaliana protein kinase 2A mRNA, complete cds
AT1G18710	2.352008013	3.34E-22	Arabidopsis thaliana myb domain protein 47 mRNA, complete cds
AT3G01510	1.132584839	3.34E-22	Arabidopsis thaliana phosphoglucan phosphatase LSF1 mRNA, complete cds
AT3G46090	3.235814996	4.18E-22	Arabidopsis thaliana chromosome 3, complete sequence
AT1G30620	1.179645944	4.32E-22	Arabidopsis thaliana UDP-arabinose 4-epimerase 1 mRNA, complete cds
AT5G01790	2.098669861	5.89E-22	Arabidopsis thaliana chromosome 5 sequence
AT2G41870	1.132331156	8.68E-22	Arabidopsis thaliana remorin-like protein mRNA, complete cds
AT1G14130	1.62335999	9.09E-22	Arabidopsis thaliana 2-oxoglutarate (2OG) and Fe(II)-dependent oxygenase-like protein mRNA, complete cds
AT1G11210	2.591276842	9.29E-22	Arabidopsis thaliana uncharacterized protein mRNA, complete cds
AT1G71100	1.207403207	1.66E-21	Arabidopsis thaliana chromosome 1 sequence
AT4G40065	3.394826337	1.79E-21	Arabidopsis thaliana chromosome 4 sequence
AT2G27830	1.20376463	2.31E-21	Arabidopsis thaliana chromosome 2, complete sequence
AT2G40435	3.960180553	2.44E-21	Arabidopsis thaliana uncharacterized protein mRNA, complete cds
AT5G44430	7.467140542	2.56E-21	Arabidopsis thaliana defensin-like protein mRNA, complete cds
AT2G38240	4.8165897	3.03E-21	Arabidopsis thaliana 2-oxoglutarate (2OG) and Fe(II)-dependent oxygenase-like protein mRNA, complete cds
AT1G74450	1.233749122	3.28E-21	Arabidopsis thaliana chromosome 1 sequence
AT1G16090	1.472787105	3.37E-21	Arabidopsis thaliana wall associated kinase-like 7 mRNA, complete cds
AT1G52050	1.953034639	3.73E-21	Arabidopsis thaliana jacalin-like lectin domain-containing protein mRNA, complete cds
AT4G21215	1.189267627	3.92E-21	Arabidopsis thaliana uncharacterized protein mRNA, complete cds
AT2G17500	1.236779687	4.45E-21	Arabidopsis thaliana auxin efflux carrier family protein mRNA, complete cds
AT1G01010	1.554774816	5.49E-21	Arabidopsis thaliana NAC domain-containing protein 1

			mRNA, complete cds
AT1G75940	7.434719064	6.28E-21	Arabidopsis thaliana beta-glucosidase 20 mRNA, complete cds
AT5G24470	1.518277676	6.83E-21	Arabidopsis thaliana pseudo-response regulator 5 mRNA, complete cds
AT3G54420	1.290974601	7.22E-21	Arabidopsis thaliana chitinase class IV mRNA, complete cds
AT1G64890	1.859263649	7.42E-21	Arabidopsis thaliana probable folate-biopterin transporter 7 mRNA, complete cds
AT1G52060	1.28281932	7.62E-21	Arabidopsis thaliana jacalin-like lectin domain-containing protein mRNA, complete cds
AT2G16060	1.571521059	7.65E-21	Arabidopsis thaliana non-symbiotic hemoglobin 1 mRNA, complete cds
AT3G61280	1.549278829	8.19E-21	Arabidopsis thaliana lipopolysaccharide-modifying domain-containing protein mRNA, complete cds
AT4G35770	2.171684659	9.22E-21	Arabidopsis thaliana senescence-associated protein DIN1 mRNA, complete cds
AT5G41120	1.470982608	9.36E-21	Arabidopsis thaliana Esterase/lipase/thioesterase family protein mRNA, complete cds
AT4G18270	1.424971088	1.05E-20	Arabidopsis thaliana phospho-N-acetylmuramoyl-pentapeptide-transferase-like protein mRNA, complete cds
AT4G13310	1.634250528	1.29E-20	Arabidopsis thaliana cytochrome P450 71A20 mRNA, complete cds
AT5G38900	2.870105209	1.51E-20	Arabidopsis thaliana Thioredoxin superfamily protein mRNA, complete cds
AT5G22270	1.975287446	1.60E-20	Arabidopsis thaliana uncharacterized protein mRNA, complete cds
AT4G10310	1.00408471	1.99E-20	Arabidopsis thaliana sodium transporter HKT1 mRNA, complete cds
AT3G50760	1.793084115	2.24E-20	Arabidopsis thaliana chromosome 3, complete sequence
AT3G56970	3.575200288	2.27E-20	Arabidopsis thaliana transcription factor ORG2 mRNA, complete cds
AT2G37770	2.071211866	2.65E-20	Arabidopsis thaliana aldo-keto reductase family 4 member C9 mRNA, complete cds
AT2G47130	1.537968499	3.03E-20	Arabidopsis thaliana short-chain dehydrogenase reductase 3 mRNA, complete cds
AT5G40500	1.356594088	3.11E-20	Arabidopsis thaliana uncharacterized protein mRNA, complete cds
AT2G22860	2.269018649	3.14E-20	Arabidopsis thaliana phytosulfokine-beta mRNA, complete cds
AT1G25370	1.664744114	3.15E-20	Arabidopsis thaliana uncharacterized protein mRNA, complete cds
AT4G39830	1.688724504	4.11E-20	Arabidopsis thaliana putative L-ascorbate oxidase mRNA,

			complete cds
AT1G80130	1.688724504	4.11E-20	Arabidopsis thaliana tetratricopeptide repeat domain-containing protein mRNA, complete cds
AT5G44820	2.044000196	4.28E-20	Arabidopsis thaliana Nucleotide-diphospho-sugar transferase family protein mRNA, complete cds
AT2G34490	1.064350614	4.58E-20	Arabidopsis thaliana chromosome 2, complete sequence
AT4G25200	3.882178041	4.62E-20	Arabidopsis thaliana small heat shock protein 23.6 mRNA, complete cds
AT1G65690	1.847279833	7.69E-20	Arabidopsis thaliana late embryogenesis abundant (LEA) hydroxyproline-rich glycoprotein mRNA, complete cds
AT1G51470	1.590752035	8.19E-20	Arabidopsis thaliana myrosinase 5 mRNA, complete cds
AT1G13470	3.02973523	9.90E-20	Arabidopsis thaliana uncharacterized protein mRNA, complete cds
AT1G02400	1.521028637	9.94E-20	Arabidopsis thaliana gibberellin 2-oxidase 6 mRNA, complete cds
AT2G39650	1.804557144	1.01E-19	Arabidopsis thaliana uncharacterized protein mRNA, complete cds
AT3G01600	2.888575813	1.04E-19	Arabidopsis thaliana NAC domain containing protein 44 mRNA, complete cds
AT4G13410	1.815979552	1.07E-19	Arabidopsis thaliana putative mannan synthase 15 mRNA, complete cds
AT2G39050	1.27552047	1.24E-19	Arabidopsis thaliana Euonymus lectin S3 mRNA, complete cds
AT5G47230	1.531680794	1.26E-19	Arabidopsis thaliana chromosome 5 sequence
AT5G54370	1.148935533	1.41E-19	Arabidopsis thaliana late embryogenesis abundant protein-like protein mRNA, complete cds
AT3G29575	1.393691796	1.64E-19	Arabidopsis thaliana Ninja-family protein AFP3 mRNA, complete cds
AT1G75960	2.000822538	1.75E-19	Arabidopsis thaliana chromosome 1 sequence
AT3G23250	2.000822538	1.75E-19	Arabidopsis thaliana myb domain protein 15 mRNA, complete cds
AT1G61610	2.434719064	3.46E-19	Arabidopsis thaliana putative G-type lectin S-receptor-like serine/threonine-protein kinase mRNA, complete cds
AT2G02990	7.279068194	3.75E-19	Arabidopsis thaliana ribonuclease 1 mRNA, complete cds
AT4G09500	2.038613381	4.57E-19	Arabidopsis thaliana chromosome 4 sequence
AT5G54190	3.8165897	4.85E-19	Arabidopsis thaliana protochlorophyllide reductase A mRNA, complete cds
AT1G02220	1.285810777	4.96E-19	Arabidopsis thaliana NAC domain-containing protein 3 mRNA, complete cds
AT1G19530	1.519111252	7.71E-19	Arabidopsis thaliana uncharacterized protein mRNA, complete cds
AT5G14730	1.860423489	8.82E-19	Arabidopsis thaliana chromosome 5 sequence
AT3G25180	7.242073986	9.41E-19	Arabidopsis thaliana cytochrome P450, family 82,

			subfamily G, polypeptide 1 mRNA, complete cds
AT2G40340	4.638252458	1.01E-18	Arabidopsis thaliana dehydration-responsive element-binding protein 2C mRNA, complete cds
AT4G27410	1.345778506	1.22E-18	Arabidopsis thaliana NAC transcription factor RD26 mRNA, complete cds
AT1G35230	1.651992515	1.42E-18	Arabidopsis thaliana chromosome 1 sequence
AT1G72330	1.081639427	3.71E-18	Arabidopsis thaliana alanine aminotransferase 2 mRNA, complete cds
AT2G36770	3.57732346	4.73E-18	Arabidopsis thaliana chromosome 2, complete sequence
AT2G42340	6.104570463	4.95E-18	Arabidopsis thaliana uncharacterized protein mRNA, complete cds
AT1G24140	1.815247033	4.98E-18	Arabidopsis thaliana chromosome 1 sequence
AT5G57050	1.236485193	6.08E-18	Arabidopsis thaliana protein phosphatase 2C 77 mRNA, complete cds
AT1G06160	2.730174948	6.26E-18	Arabidopsis thaliana chromosome 1 sequence
AT2G21640	2.023221923	7.85E-18	Arabidopsis thaliana uncharacterized oxidative stress response protein mRNA, complete cds
AT3G21510	1.225514075	1.03E-17	Arabidopsis thaliana histidine-containing phosphotransmitter 1 mRNA, complete cds
AT2G44578	5.083811903	1.34E-17	Arabidopsis thaliana chromosome 2, complete sequence
AT2G29170	6.062750287	1.42E-17	Arabidopsis thaliana NAD(P)-binding Rossmann-fold superfamily protein mRNA, complete cds
AT2G21130	1.258869229	1.70E-17	Arabidopsis thaliana chromosome 2, complete sequence
AT3G54150	2.223214959	1.70E-17	Arabidopsis thaliana S-adenosyl-L-methionine-dependent methyltransferase-like protein mRNA, complete cds
AT2G32140	1.966009632	1.95E-17	Arabidopsis thaliana transmembrane receptor protein mRNA, complete cds
AT5G05340	2.826144006	1.99E-17	Arabidopsis thaliana peroxidase 52 mRNA, complete cds
AT3G26280	1.243122838	2.14E-17	Arabidopsis thaliana cytochrome P450 71B4 mRNA, complete cds
AT5G37450	1.193192476	2.17E-17	Arabidopsis thaliana probable LRR receptor-like serine/threonine-protein kinase mRNA, complete cds
AT1G66600	3.055014638	2.49E-17	Arabidopsis thaliana putative WRKY transcription factor 63 mRNA, complete cds
AT5G59520	1.873407832	2.61E-17	Arabidopsis thaliana zinc transporter 2 mRNA, complete cds
AT1G61340	2.242767757	2.65E-17	Arabidopsis thaliana F-box stress induced 1 mRNA, complete cds
AT1G10140	1.084298068	2.99E-17	Arabidopsis thaliana chromosome 1 sequence
AT1G76960	2.370588727	3.19E-17	Arabidopsis thaliana uncharacterized protein mRNA, complete cds
AT4G02360	2.486249365	3.74E-17	Arabidopsis thaliana chromosome 4 sequence
AT1G74360	1.251181063	3.93E-17	Arabidopsis thaliana putative LRR receptor-like

			serine/threonine-protein kinase mRNA, complete cds
AT1G58430	6.019681565	4.11E-17	Arabidopsis thaliana anther-specific proline-rich protein RXF26 mRNA, complete cds
AT2G29990	1.357158081	4.12E-17	Arabidopsis thaliana alternative NAD(P)H dehydrogenase 2 mRNA, complete cds
AT5G22570	3.367604869	4.32E-17	Arabidopsis thaliana putative WRKY transcription factor 38 mRNA, complete cds
AT4G36850	1.699313984	4.35E-17	Arabidopsis thaliana PQ-loop repeat family protein / transmembrane family protein mRNA, complete cds
AT3G18500	1.096805265	5.00E-17	Arabidopsis thaliana DNAse I-like superfamily protein mRNA, complete cds
AT1G20190	1.145212447	6.09E-17	Arabidopsis thaliana expansin-A11 mRNA, complete cds
AT1G76470	3.231627199	6.57E-17	Arabidopsis thaliana NAD(P)-binding Rossmann-fold superfamily protein mRNA, complete cds
AT2G36690	1.870950786	6.72E-17	Arabidopsis thaliana 2-oxoglutarate (2OG) and Fe(II)-dependent oxygenase superfamily protein mRNA, complete cds
AT1G10060	1.604644066	7.14E-17	Arabidopsis thaliana branched-chain-amino-acid aminotransferase 1 mRNA, complete cds
AT1G61800	5.019681565	7.46E-17	Arabidopsis thaliana glucose-6-phosphate/phosphate translocator 2 mRNA, complete cds
AT4G33070	1.818047704	8.99E-17	Arabidopsis thaliana pyruvate decarboxylase 1 mRNA, complete cds
AT1G02450	3.862812716	1.01E-16	Arabidopsis thaliana chromosome 1 sequence
AT3G05660	2.782642368	1.03E-16	Arabidopsis thaliana receptor like protein 33 mRNA, complete cds
AT1G64660	1.245376609	1.06E-16	Arabidopsis thaliana methionine gamma-lyase mRNA, complete cds
AT3G23240	4.997655259	1.33E-16	Arabidopsis thaliana chromosome 3, complete sequence
AT3G46620	1.000566854	1.69E-16	Arabidopsis thaliana chromosome 3, complete sequence
AT4G35480	1.380382142	1.82E-16	Arabidopsis thaliana chromosome 4 sequence
AT3G19430	1.975287446	2.12E-16	Arabidopsis thaliana late embryogenesis abundant protein-like protein mRNA, complete cds
AT5G23850	1.168595375	2.14E-16	Arabidopsis thaliana uncharacterized protein mRNA, complete cds
AT3G28580	2.087180326	2.16E-16	Arabidopsis thaliana chromosome 3, complete sequence
AT3G19580	1.155554391	2.55E-16	Arabidopsis thaliana chromosome 3, complete sequence
AT5G57010	1.752895024	2.75E-16	Arabidopsis thaliana calmodulin-binding family protein mRNA, complete cds
AT1G69890	1.067853564	3.67E-16	Arabidopsis thaliana uncharacterized protein mRNA, complete cds
AT1G06090	1.633006289	4.51E-16	Arabidopsis thaliana delta-9 desaturase-like 1 protein mRNA, complete cds
AT4G37180	1.021830032	4.57E-16	Arabidopsis thaliana myb family transcription factor

			mRNA, complete cds
AT1G79310	1.935759082	5.23E-16	Arabidopsis thaliana metacaspase 7 mRNA, complete cds
AT5G67390	1.394512742	5.24E-16	Arabidopsis thaliana uncharacterized protein mRNA, complete cds
AT4G38560	2.117306451	5.48E-16	Arabidopsis thaliana phospholipase like protein (PEARLI 4) mRNA, complete cds
AT2G47560	2.090764663	5.58E-16	Arabidopsis thaliana chromosome 2, complete sequence
AT2G23680	1.140625178	5.72E-16	Arabidopsis thaliana Cold acclimation protein WCOR413 family mRNA, complete cds
AT2G28850	1.08580816	6.29E-16	Arabidopsis thaliana chromosome 2, complete sequence
AT2G16720	1.221927413	6.76E-16	Arabidopsis thaliana myb domain protein 7 mRNA, complete cds
AT4G16740	6.952567369	6.85E-16	Arabidopsis thaliana tricyclene synthase mRNA, complete cds
AT3G09020	1.423340002	7.06E-16	Arabidopsis thaliana chromosome 3, complete sequence
AT5G10300	1.286074983	7.16E-16	Arabidopsis thaliana methyl esterase 5 mRNA, complete cds
AT5G63130	3.279068194	7.73E-16	Arabidopsis thaliana octicosapeptide/Phox/Bem1p domain-containing protein mRNA, complete cds
AT5G64750	1.925705417	8.28E-16	Arabidopsis thaliana ethylene-responsive transcription factor ABR1 mRNA, complete cds
AT1G06080	1.416797156	1.03E-15	Arabidopsis thaliana delta-9 acyl-lipid desaturase 1 mRNA, complete cds
AT4G12735	6.929483756	1.11E-15	Arabidopsis thaliana chromosome 4 sequence
AT1G52770	1.778055098	1.35E-15	Arabidopsis thaliana phototropic-responsive NPH3 family protein mRNA, complete cds
AT2G34260	1.23192408	1.46E-15	Arabidopsis thaliana protein WDR55 mRNA, complete cds
AT5G16600	1.062223083	1.52E-15	Arabidopsis thaliana myb domain protein 43 mRNA, complete cds
AT2G32120	1.288619517	1.59E-15	Arabidopsis thaliana heat-shock protein 70T-2 mRNA, complete cds
AT3G02840	2.531680794	1.61E-15	Arabidopsis thaliana chromosome 3, complete sequence
AT3G09540	1.090029532	1.66E-15	Arabidopsis thaliana pectin lyase-like superfamily protein mRNA, complete cds
AT4G23130	1.69775347	2.18E-15	Arabidopsis thaliana cysteine-rich receptor-like protein kinase 5 mRNA, complete cds
AT5G60530	1.002918565	2.73E-15	Arabidopsis thaliana late embryogenesis abundant protein-like protein mRNA, complete cds
AT2G41730	3.377385889	2.85E-15	Arabidopsis thaliana chromosome 2, complete sequence
AT3G13950	6.882178041	2.91E-15	Arabidopsis thaliana uncharacterized protein mRNA, complete cds
AT1G20130	5.833268441	3.02E-15	Arabidopsis thaliana anther-specific proline-rich protein APG mRNA, complete cds

AT3G11840	1.515855827	3.20E-15	Arabidopsis thaliana E3 ubiquitin-protein ligase PUB24 mRNA, complete cds
AT1G23550	2.127290539	3.65E-15	Arabidopsis thaliana probable inactive poly [ADP-ribose] polymerase SRO2 mRNA, complete cds
AT2G41380	1.997655259	3.74E-15	Arabidopsis thaliana S-adenosyl-L-methionine-dependent methyltransferase-like protein mRNA, complete cds
AT1G28610	1.012124214	4.81E-15	Arabidopsis thaliana GDSL esterase/lipase mRNA, complete cds
AT1G66370	6.833268441	7.71E-15	Arabidopsis thaliana transcription factor MYB113 mRNA, complete cds
AT2G38760	1.408940623	7.93E-15	Arabidopsis thaliana annexin D3 mRNA, complete cds
AT5G18290	1.874358537	8.11E-15	Arabidopsis thaliana putative aquaporin SIP1-2 mRNA, complete cds
AT4G01540	1.480281642	8.12E-15	Arabidopsis thaliana NAC with transmembrane motif1 mRNA, complete cds
AT5G61290	1.892825286	8.46E-15	Arabidopsis thaliana flavin-containing monooxygenase FMO GS-OX-like 8 mRNA, complete cds
AT5G03210	5.782642368	8.95E-15	Arabidopsis thaliana chromosome 5 sequence
AT1G72416	1.031700755	8.97E-15	Arabidopsis thaliana chaperone DnaJ-domain containing protein mRNA, complete cds
AT1G21520	1.641169942	9.80E-15	Arabidopsis thaliana uncharacterized protein mRNA, complete cds
AT3G28540	1.132828723	1.02E-14	Arabidopsis thaliana chromosome 3, complete sequence
AT1G10040	1.459220026	1.55E-14	Arabidopsis thaliana alpha/beta-hydrolases superfamily protein mRNA, complete cds
AT3G47780	1.326759816	1.77E-14	Arabidopsis thaliana ABC transporter A family member 7 mRNA, complete cds
AT3G09405	1.560249946	1.79E-14	Arabidopsis thaliana pectinacetylesterase family protein mRNA, complete cds
AT2G42200	1.284884739	1.84E-14	Arabidopsis thaliana squamosa promoter-binding-like protein 9 mRNA, complete cds
AT5G20740	1.05429532	1.92E-14	Arabidopsis thaliana chromosome 5 sequence
AT2G44840	2.85570583	2.05E-14	Arabidopsis thaliana chromosome 2, complete sequence
AT1G10070	2.457680213	2.23E-14	Arabidopsis thaliana branched-chain-amino-acid aminotransferase 2 mRNA, complete cds
AT5G24110	1.623575882	2.25E-14	Arabidopsis thaliana WRKY DNA-binding protein 30 mRNA, complete cds
AT5G17860	3.165112005	2.42E-14	Arabidopsis thaliana chromosome 5 sequence
AT4G25480	4.782642368	2.42E-14	Arabidopsis thaliana chromosome 4 sequence
AT1G35140	1.699653003	2.58E-14	Arabidopsis thaliana chromosome 1 sequence
AT3G56980	2.765364376	2.65E-14	Arabidopsis thaliana transcription factor ORG3 mRNA, complete cds
AT2G38250	2.356716552	2.80E-14	Arabidopsis thaliana trihelix transcription factor GT-3b mRNA, complete cds

AT5G67180	1.560249946	2.99E-14	Arabidopsis thaliana target of early activation tagged 3 mRNA, complete cds
AT5G59510	2.684238664	3.31E-14	Arabidopsis thaliana chromosome 5 sequence
AT1G73810	2.837783922	3.57E-14	Arabidopsis thaliana core-2/I-branching beta-1,6-N-acetylglucosaminyltransferase-like protein mRNA, complete cds
AT5G64870	2.09502669	4.03E-14	Arabidopsis thaliana SPFH/Band 7/PHB domain-containing membrane-associated protein family mRNA, complete cds
AT5G16980	3.145212447	4.28E-14	Arabidopsis thaliana zinc-binding dehydrogenase family protein mRNA, complete cds
AT2G20750	1.170303428	4.93E-14	Arabidopsis thaliana expansin B1 mRNA, complete cds
AT1G21550	2.483082086	5.56E-14	Arabidopsis thaliana chromosome 1 sequence
AT1G62540	1.041089504	5.59E-14	Arabidopsis thaliana flavin-containing monooxygenase FMO GS-OX2 mRNA, complete cds
AT3G16565	1.180871579	5.70E-14	Arabidopsis thaliana threonyl and alanyl tRNA synthetase second additional domain-containing protein mRNA, complete cds
AT4G17615	1.108718433	5.84E-14	Arabidopsis thaliana calcineurin B-like protein 1 mRNA, complete cds
AT5G50800	2.025913519	6.57E-14	Arabidopsis thaliana bidirectional sugar transporter SWEET13 mRNA, complete cds
AT4G30660	4.223214959	7.14E-14	Arabidopsis thaliana putative low temperature and salt responsive protein mRNA, complete cds
AT2G17280	1.350986797	8.02E-14	Arabidopsis thaliana phosphoglycerate mutase-like protein mRNA, complete cds
AT5G13205	1.488696685	8.13E-14	Arabidopsis thaliana chromosome 5 sequence
AT2G34600	6.7032079	9.03E-14	Arabidopsis thaliana jasmonate-zim-domain protein 7 mRNA, complete cds
AT5G55050	1.310890478	1.15E-13	Arabidopsis thaliana GDSL esterase/lipase mRNA, complete cds
AT4G18360	1.363984553	1.23E-13	Arabidopsis thaliana glycolate oxidase mRNA, complete cds
AT1G47600	1.284040152	1.29E-13	Arabidopsis thaliana myrosinase 4 mRNA, complete cds
AT2G21020	1.233355599	1.32E-13	Arabidopsis thaliana chromosome 2, complete sequence
AT2G23910	1.518185887	1.32E-13	Arabidopsis thaliana Rossmann-fold NAD(P)-binding domain-containing protein mRNA, complete cds
AT1G79680	4.7032079	1.40E-13	Arabidopsis thaliana wall-associated receptor kinase-like 10 mRNA, complete cds
AT1G58225	2.632399732	1.68E-13	Arabidopsis thaliana uncharacterized protein mRNA, complete cds
ATCG00020	1.06839685	1.93E-13	Arabidopsis thaliana chloroplast DNA, complete genome, ecotype: Columbia
AT5G59720	1.588264323	1.95E-13	Arabidopsis thaliana chromosome 5 sequence

AT1G16420	2.560249946	2.00E-13	Arabidopsis thaliana metacaspase 8 mRNA, complete cds
AT1G26730	1.240877556	2.09E-13	Arabidopsis thaliana phosphate transporter PHO1-7 mRNA, complete cds
AT5G42250	1.167105053	2.10E-13	Arabidopsis thaliana alcohol dehydrogenase-like 7 mRNA, complete cds
AT2G29470	5.619143636	2.41E-13	Arabidopsis thaliana glutathione S-transferase tau 3 mRNA, complete cds
AT5G16380	1.349411752	2.55E-13	Arabidopsis thaliana uncharacterized protein mRNA, complete cds
AT5G25260	1.675727164	2.57E-13	Arabidopsis thaliana SPFH/Band 7/PHB domain-containing membrane-associated protein mRNA, complete cds
AT1G72920	1.43673541	2.60E-13	Arabidopsis thaliana Toll-Interleukin-Resistance domain-containing protein mRNA, complete cds
AT3G14440	1.988111486	2.70E-13	Arabidopsis thaliana chromosome 3, complete sequence
AT5G39850	1.046170932	3.38E-13	Arabidopsis thaliana 40S ribosomal protein S9-2 mRNA, complete cds
AT5G17460	1.598982341	3.63E-13	Arabidopsis thaliana uncharacterized protein mRNA, complete cds
AT5G01830	1.064663253	3.64E-13	Arabidopsis thaliana chromosome 5 sequence
AT1G13480	1.665602947	3.92E-13	Arabidopsis thaliana uncharacterized protein mRNA, complete cds
AT3G20300	1.239628049	4.01E-13	Arabidopsis thaliana uncharacterized protein mRNA, complete cds
AT4G18280	1.390324945	4.70E-13	Arabidopsis thaliana chromosome 4 sequence
AT1G05575	3.190300337	5.13E-13	Arabidopsis thaliana chromosome 1 sequence
AT5G22300	1.643305686	5.43E-13	Arabidopsis thaliana bifunctional nitrilase/nitrile hydratase NIT4 mRNA, complete cds
AT2G03310	1.073319529	6.05E-13	Arabidopsis thaliana uncharacterized protein mRNA, complete cds
AT2G32100	1.151137281	6.54E-13	Arabidopsis thaliana chromosome 2, complete sequence
AT1G11185	2.182882865	6.58E-13	Arabidopsis thaliana chromosome 1 sequence
AT4G13800	2.145212447	6.82E-13	Arabidopsis thaliana uncharacterized protein mRNA, complete cds
AT2G29090	2.045676774	7.13E-13	Arabidopsis thaliana abscisic acid 8'-hydroxylase 2 mRNA, complete cds
AT3G16360	4.1182454	7.63E-13	Arabidopsis thaliana histidine-containing phosphotransfer protein 4 mRNA, complete cds
AT2G29490	1.694551038	7.84E-13	Arabidopsis thaliana glutathione S-transferase tau 1 mRNA, complete cds
AT4G13300	1.412351251	8.11E-13	Arabidopsis thaliana (Z)-gamma-bisabolene synthase 2 mRNA, complete cds
AT1G07260	1.297215541	8.62E-13	Arabidopsis thaliana chromosome 1 sequence
AT1G49450	1.474858455	9.40E-13	Arabidopsis thaliana chromosome 1 sequence

AT5G67210	1.043624576	1.04E-12	Arabidopsis thaliana chromosome 5 sequence
AT1G17180	2.167932524	1.08E-12	Arabidopsis thaliana glutathione S-transferase TAU 25 mRNA, complete cds
AT3G47480	1.683824632	1.20E-12	Arabidopsis thaliana chromosome 3, complete sequence
AT3G26170	1.07716706	1.37E-12	Arabidopsis thaliana cytochrome P450 71B19 mRNA, complete cds
AT2G47520	4.58999729	1.46E-12	Arabidopsis thaliana ethylene-responsive transcription factor ERF071 mRNA, complete cds
AT1G65480	2.235154573	1.61E-12	Arabidopsis thaliana protein FLOWERING LOCUS T mRNA, complete cds
AT1G74430	2.892825286	1.76E-12	Arabidopsis thaliana putative transcription factor MYB95 mRNA, complete cds
AT1G48370	1.086094232	1.93E-12	Arabidopsis thaliana metal-nicotianamine transporter YSL8 mRNA, complete cds
AT5G23980	1.776741766	1.93E-12	Arabidopsis thaliana ferric reduction oxidase 4 mRNA, complete cds
AT3G46930	1.134687267	2.02E-12	Arabidopsis thaliana protein kinase family protein mRNA, complete cds
AT5G44050	1.382104438	2.18E-12	Arabidopsis thaliana MATE efflux family protein mRNA, complete cds
AT4G10500	1.289798069	2.36E-12	Arabidopsis thaliana oxidoreductase, 2OG-Fe(II) oxygenase family protein mRNA, complete cds
AT5G39120	1.479329951	2.99E-12	Arabidopsis thaliana germin-like protein subfamily 1 member 15 mRNA, complete cds
AT3G50260	1.425948855	3.38E-12	Arabidopsis thaliana chromosome 3, complete sequence
AT2G44460	2.599778311	3.50E-12	Arabidopsis thaliana beta glucosidase 28 mRNA, complete cds
AT2G41835	1.191016137	3.66E-12	Arabidopsis thaliana zinc finger (C2H2 type, AN1-like) family protein mRNA, complete cds
AT2G34090	1.113993717	3.81E-12	Arabidopsis thaliana maternal effect embryo arrest 18 protein mRNA, complete cds
AT3G25882	3.098669861	5.06E-12	Arabidopsis thaliana chromosome 3, complete sequence
AT1G51820	2.33283945	5.92E-12	Arabidopsis thaliana putative LRR receptor-like serine/threonine protein kinase mRNA, complete cds
AT1G63840	1.042926163	6.13E-12	Arabidopsis thaliana chromosome 1 sequence
AT5G61660	1.211824049	6.70E-12	Arabidopsis thaliana chromosome 5 sequence
AT5G07475	1.156734317	7.25E-12	Arabidopsis thaliana cupredoxin superfamily protein mRNA, complete cds
AT5G48657	1.012762151	7.27E-12	Arabidopsis thaliana defense protein-like protein mRNA, complete cds
AT4G23230	1.604305127	8.02E-12	Arabidopsis thaliana cysteine-rich receptor-like protein kinase 15 mRNA, complete cds
AT5G22380	4.005034789	8.19E-12	Arabidopsis thaliana NAC domain-containing protein mRNA, complete cds

AT4G36950	6.434719064	8.46E-12	Arabidopsis thaliana chromosome 4 sequence
AT1G67990	6.434719064	8.46E-12	Arabidopsis thaliana tapetum-specific methyltransferase 1 mRNA, complete cds
AT1G65500	1.795466408	8.84E-12	Arabidopsis thaliana uncharacterized protein mRNA, complete cds
AT1G31173	2.21475338	1.13E-11	Arabidopsis thaliana chromosome 1 sequence
AT2G42800	1.09691689	1.15E-11	Arabidopsis thaliana chromosome 2, complete sequence
AT2G22880	2.719448541	1.19E-11	Arabidopsis thaliana chromosome 2, complete sequence
AT2G32220	1.7458056	1.19E-11	Arabidopsis thaliana chromosome 2, complete sequence
AT5G06730	1.655669512	1.19E-11	Arabidopsis thaliana peroxidase 54 mRNA, complete cds
AT1G16370	1.782642368	1.38E-11	Arabidopsis thaliana chromosome 1 sequence
AT1G77960	3.647712788	1.41E-11	Arabidopsis thaliana uncharacterized protein mRNA, complete cds
AT1G11925	6.4015522	1.41E-11	Arabidopsis thaliana chromosome 1 sequence
AT5G22460	1.560249946	1.46E-11	Arabidopsis thaliana esterase/lipase/thioesterase family protein mRNA, complete cds
AT5G04150	4.467140542	1.54E-11	Arabidopsis thaliana transcription factor bHLH101 mRNA, complete cds
AT2G27690	2.197679867	1.86E-11	Arabidopsis thaliana chromosome 2, complete sequence
AT1G79410	1.157227823	1.90E-11	Arabidopsis thaliana chromosome 1 sequence
AT4G19230	1.314089359	2.11E-11	Arabidopsis thaliana abscisic acid 8'-hydroxylase 1 mRNA, complete cds
AT4G32800	1.047437232	2.28E-11	Arabidopsis thaliana chromosome 4 sequence
AT2G21900	3.381279805	2.28E-11	Arabidopsis thaliana putative WRKY transcription factor 59 mRNA, complete cds
AT1G62280	1.509266018	2.30E-11	Arabidopsis thaliana S-type anion channel SLAH1 mRNA, complete cds
AT5G39110	1.738248248	3.07E-11	Arabidopsis thaliana germin-like protein subfamily 1 member 14 mRNA, complete cds
AT4G29610	3.171684659	3.45E-11	Arabidopsis thaliana chromosome 4 sequence
AT5G05590	1.297215541	4.32E-11	Arabidopsis thaliana phosphoribosylanthranilate isomerase 2 mRNA, complete cds
AT4G23280	3.000822538	4.91E-11	Arabidopsis thaliana putative cysteine-rich receptor-like protein kinase 20 mRNA, complete cds
AT2G23150	1.16896857	4.95E-11	Arabidopsis thaliana metal transporter Nramp3 mRNA, complete cds
AT3G28007	2.424594847	5.74E-11	Arabidopsis thaliana bidirectional sugar transporter SWEET4 mRNA, complete cds
AT5G55450	1.00408471	5.76E-11	Arabidopsis thaliana bifunctional inhibitor/lipid-transfer protein/seed storage 2S albumin-like protein mRNA, complete cds
AT1G66760	1.128057726	5.91E-11	Arabidopsis thaliana MATE efflux family protein mRNA, complete cds

AT5G08240	1.043851732	5.95E-11	Arabidopsis thaliana uncharacterized protein mRNA, complete cds
AT1G59590	1.238321852	6.05E-11	Arabidopsis thaliana chromosome 1 sequence
AT5G55110	5.297215541	6.60E-11	Arabidopsis thaliana chromosome 5 sequence
AT1G69930	6.297215541	6.70E-11	Arabidopsis thaliana glutathione S-transferase TAU 11 mRNA, complete cds
AT5G43370	1.71225304	7.31E-11	Arabidopsis thaliana phosphate transporter Pht1;2 mRNA, complete cds
AT1G76980	1.102044588	8.02E-11	Arabidopsis thaliana uncharacterized protein mRNA, complete cds
AT2G24720	1.094123675	9.33E-11	Arabidopsis thaliana glutamate receptor 2.2 mRNA, complete cds
AT5G43580	1.344521255	9.70E-11	Arabidopsis thaliana unusual seine protease inhibitor mRNA, complete cds
AT3G51590	6.260689665	1.13E-10	Arabidopsis thaliana non-specific lipid-transfer protein 12 mRNA, complete cds
AT3G29000	1.859810228	1.17E-10	Arabidopsis thaliana chromosome 3, complete sequence
AT2G37580	2.223214959	1.24E-10	Arabidopsis thaliana chromosome 2, complete sequence
AT2G36780	3.297215541	1.31E-10	Arabidopsis thaliana chromosome 2, complete sequence
AT5G44350	2.045676774	1.37E-10	Arabidopsis thaliana chromosome 5 sequence
AT4G03450	1.421803176	1.61E-10	Arabidopsis thaliana ankyrin repeat-containing protein mRNA, complete cds
AT4G22980	1.139674264	1.83E-10	Arabidopsis thaliana chromosome 4 sequence
AT5G52160	6.223214959	1.91E-10	Arabidopsis thaliana bifunctional inhibitor/lipid-transfer protein/seed storage 2S albumin-like protein mRNA, complete cds
AT4G30460	1.325784693	2.01E-10	Arabidopsis thaliana chromosome 4 sequence
AT2G47780	2.154611145	2.12E-10	Arabidopsis thaliana Rubber elongation factor protein mRNA, complete cds
AT1G24260	2.515855827	2.13E-10	Arabidopsis thaliana MADs box transcription factor SEPALLATA3 mRNA, complete cds
AT5G52390	1.992360959	2.19E-10	Arabidopsis thaliana PAR1 protein mRNA, complete cds
AT3G09270	1.205900374	2.37E-10	Arabidopsis thaliana glutathione S-transferase TAU 8 mRNA, complete cds
AT3G28500	1.598724094	2.70E-10	Arabidopsis thaliana chromosome 3, complete sequence
AT3G61920	1.363852734	2.86E-10	Arabidopsis thaliana chromosome 3, complete sequence
AT5G60650	1.854993212	3.05E-10	Arabidopsis thaliana chromosome 5 sequence
AT4G24415	1.503219001	3.05E-10	Arabidopsis thaliana AT4g24411 mRNA, complete cds
AT3G60140	1.226419763	3.15E-10	Arabidopsis thaliana beta-glucosidase 30 mRNA, complete cds
AT3G22275	6.184740811	3.24E-10	Arabidopsis thaliana uncharacterized protein mRNA, complete cds
AT4G14080	6.184740811	3.24E-10	Arabidopsis thaliana putative glucan

			endo-1,3-beta-glucosidase A6 mRNA, complete cds
AT5G53750	1.261810205	3.40E-10	Arabidopsis thaliana CBS domain-containing protein mRNA, complete cds
AT4G04540	2.782642368	3.50E-10	Arabidopsis thaliana putative cysteine-rich receptor-like protein kinase 39 mRNA, complete cds
AT1G02820	1.572849983	3.66E-10	Arabidopsis thaliana late embryogenesis abundant 3-like protein mRNA, complete cds
AT1G80820	1.105486169	4.04E-10	Arabidopsis thaliana cinnamoyl-CoA reductase mRNA, complete cds
AT2G43510	1.790255552	4.25E-10	Arabidopsis thaliana trypsin inhibitor protein 1 mRNA, complete cds
AT4G16515	1.599778311	4.54E-10	Arabidopsis thaliana chromosome 4 sequence
AT5G35490	1.560249946	5.50E-10	Arabidopsis thaliana chromosome 5 sequence
AT1G62940	6.145212447	5.50E-10	Arabidopsis thaliana acyl-CoA synthetase 5 mRNA, complete cds
AT4G28395	6.145212447	5.50E-10	Arabidopsis thaliana lipid transfer protein-related mRNA, complete cds
AT3G26960	1.000822538	5.61E-10	Arabidopsis thaliana pollen Ole e 1 allergen and extensin family protein mRNA, complete cds
AT4G06746	2.470052137	6.14E-10	Arabidopsis thaliana chromosome 4 sequence
AT3G46110	1.089544625	6.44E-10	Arabidopsis thaliana uncharacterized protein mRNA, complete cds
AT1G09090	1.089544625	6.44E-10	Arabidopsis thaliana Respiratory burst oxidase-B mRNA, complete cds
AT3G22910	1.679302618	7.41E-10	Arabidopsis thaliana chromosome 3, complete sequence
AT4G23680	1.06364332	8.46E-10	Arabidopsis thaliana polyketide cyclase/dehydrase and lipid transport superfamily protein mRNA, complete cds
AT2G46495	2.012762151	9.30E-10	Arabidopsis thaliana putative RING-H2 finger protein mRNA, complete cds
AT3G28890	3.74787695	9.50E-10	Arabidopsis thaliana receptor like protein 43 mRNA, complete cds
AT3G47510	1.008607519	9.57E-10	Arabidopsis thaliana uncharacterized protein mRNA, complete cds
AT1G48330	1.4015522	1.10E-09	Arabidopsis thaliana chromosome 1 sequence
AT1G63720	1.351663325	1.33E-09	Arabidopsis thaliana uncharacterized protein mRNA, complete cds
AT2G46870	1.095917151	1.46E-09	Arabidopsis thaliana chromosome 2, complete sequence
AT1G68875	6.062750287	1.60E-09	Arabidopsis thaliana chromosome 1 sequence
AT4G18530	1.09727797	1.72E-09	Arabidopsis thaliana uncharacterized protein mRNA, complete cds
AT5G15960	4.184740811	1.79E-09	Arabidopsis thaliana cold and ABA inducible protein kin1 mRNA, complete cds
AT1G71000	2.975287446	1.88E-09	Arabidopsis thaliana chaperone DnaJ-domain containing protein mRNA, complete cds

AT3G17790	1.017515681	1.92E-09	Arabidopsis thaliana purple acid phosphatase 17 mRNA, complete cds
AT1G68620	1.588264323	1.94E-09	Arabidopsis thaliana chromosome 1 sequence
AT4G33985	2.212326643	2.25E-09	Arabidopsis thaliana uncharacterized protein mRNA, complete cds
AT1G52560	1.975287446	2.40E-09	Arabidopsis thaliana heat shock protein 26.5 mRNA, complete cds
AT2G32030	1.728194584	2.47E-09	Arabidopsis thaliana chromosome 2, complete sequence
AT1G67030	1.302452189	2.54E-09	Arabidopsis thaliana chromosome 1 sequence
AT2G24210	6.019681565	2.74E-09	Arabidopsis thaliana terpene synthase 10 mRNA, complete cds
AT3G18690	1.026426295	2.77E-09	Arabidopsis thaliana chromosome 3, complete sequence
AT1G77450	1.061796439	3.09E-09	Arabidopsis thaliana NAC domain containing protein 32 mRNA, complete cds
AT1G32690	2.321062283	3.27E-09	Arabidopsis thaliana chromosome 1 sequence
AT1G51830	2.944913797	3.32E-09	Arabidopsis thaliana putative leucine-rich repeat protein kinase mRNA, complete cds
AT4G24340	1.367604869	3.39E-09	Arabidopsis thaliana phosphorylase family protein mRNA, complete cds
AT5G51580	1.886750771	3.59E-09	Arabidopsis thaliana chromosome 5 sequence
AT5G46080	1.213692185	3.68E-09	Arabidopsis thaliana chromosome 5 sequence
AT5G50335	2.560249946	3.82E-09	Arabidopsis thaliana chromosome 5 sequence
AT4G19750	2.560249946	3.82E-09	Arabidopsis thaliana Glycosyl hydrolase family protein with chitinase insertion domain mRNA, complete cds
AT3G61560	1.154090599	3.92E-09	Arabidopsis thaliana reticulon-like protein B6 mRNA, complete cds
AT4G23200	2.460714273	4.43E-09	Arabidopsis thaliana putative cysteine-rich receptor-like protein kinase 12 mRNA, complete cds
AT1G53625	2.460714273	4.43E-09	Arabidopsis thaliana chromosome 1 sequence
AT5G44540	5.975287446	4.70E-09	Arabidopsis thaliana chromosome 5 sequence
AT4G38940	1.22076348	5.03E-09	Arabidopsis thaliana chromosome 4 sequence
AT3G50770	1.837783922	5.40E-09	Arabidopsis thaliana chromosome 3, complete sequence
AT5G39610	1.696133375	5.86E-09	Arabidopsis thaliana NAC-domain transcription factor mRNA, complete cds
AT2G05380	1.606053636	6.09E-09	Arabidopsis thaliana glycine-rich protein 3 short isoform mRNA, complete cds
AT2G18350	1.546036087	6.60E-09	Arabidopsis thaliana chromosome 2, complete sequence
AT1G76690	1.246589468	6.63E-09	Arabidopsis thaliana 12-oxophytodienoate reductase 2 mRNA, complete cds
AT1G33430	2.434719064	7.51E-09	Arabidopsis thaliana putative beta-1,3-galactosyltransferase 8 mRNA, complete cds
AT5G26230	1.078380939	8.46E-09	Arabidopsis thaliana chromosome 5 sequence
AT3G56330	1.138423282	8.84E-09	Arabidopsis thaliana N2,N2-dimethylguanosine tRNA

			methyltransferase mRNA, complete cds
AT1G24145	1.468826919	8.98E-09	Arabidopsis thaliana uncharacterized protein mRNA, complete cds
AT1G67000	2.272967994	9.10E-09	Arabidopsis thaliana probable receptor-like protein kinase mRNA, complete cds
ATMG01080	2.145212447	1.00E-08	Arabidopsis thaliana ecotype Landsberg erecta mitochondrion, complete genome
AT4G11370	2.505802162	1.11E-08	Arabidopsis thaliana chromosome 4 sequence
AT3G06460	1.773653585	1.24E-08	Arabidopsis thaliana chromosome 3, complete sequence
AT4G24380	1.560249946	1.25E-08	Arabidopsis thaliana uncharacterized protein mRNA, complete cds
AT3G45730	1.040875787	1.27E-08	Arabidopsis thaliana chromosome 3, complete sequence
AT2G42990	2.408246853	1.27E-08	Arabidopsis thaliana GDSL esterase/lipase mRNA, complete cds
AT3G21950	1.050575573	1.30E-08	Arabidopsis thaliana methyltransferase mRNA, complete cds
AT3G42180	1.801258046	1.33E-08	Arabidopsis thaliana Exostosin family protein mRNA, complete cds
AT5G62080	5.882178041	1.40E-08	Arabidopsis thaliana protease inhibitor/seed storage/lipid transfer protein (LTP) family protein mRNA, complete cds
AT5G49015	1.862812716	1.49E-08	Arabidopsis thaliana uncharacterized protein mRNA, complete cds
AT4G24010	1.862812716	1.49E-08	Arabidopsis thaliana cellulose synthase-like protein G1 mRNA, complete cds
AT2G32190	2.181738323	1.59E-08	Arabidopsis thaliana uncharacterized protein mRNA, complete cds
AT1G74930	1.975287446	1.65E-08	Arabidopsis thaliana chromosome 1 sequence
AT3G04010	1.215338534	1.86E-08	Arabidopsis thaliana O-glycosyl hydrolases family 17 protein mRNA, complete cds
AT2G37430	1.782642368	2.08E-08	Arabidopsis thaliana chromosome 2, complete sequence
AT3G06490	4.882178041	2.14E-08	Arabidopsis thaliana putative transcription factor MYB108 mRNA, complete cds
AT1G75030	1.66716515	2.32E-08	Arabidopsis thaliana thaumatin-like protein 3 mRNA, complete cds
AT1G52830	3.010911355	2.35E-08	Arabidopsis thaliana indole-3-acetic acid 6 mRNA, complete cds
AT5G43620	1.877107052	2.46E-08	Arabidopsis thaliana chromosome 5 sequence
AT4G01550	1.007348655	2.55E-08	Arabidopsis thaliana NAC transcription factor mRNA, complete cds
AT4G17660	3.223214959	2.90E-08	Arabidopsis thaliana protein kinase family protein mRNA, complete cds
AT1G12480	1.737127709	3.00E-08	Arabidopsis thaliana guard cell S-type anion channel SLAC1 mRNA, complete cds

AT1G07430	2.8165897	3.15E-08	Arabidopsis thaliana protein phosphatase 2C 3 mRNA, complete cds
AT1G15790	1.089243635	3.15E-08	Arabidopsis thaliana uncharacterized protein mRNA, complete cds
AT1G61470	2.449218634	3.22E-08	Arabidopsis thaliana chromosome 1 sequence
AT4G22470	2.449218634	3.22E-08	Arabidopsis thaliana chromosome 4 sequence
AT4G15280	2.353799069	3.60E-08	Arabidopsis thaliana chromosome 4 sequence
AT4G11911	2.975287446	4.16E-08	Arabidopsis thaliana uncharacterized protein mRNA, complete cds
AT4G10250	5.782642368	4.19E-08	Arabidopsis thaliana chromosome 4 sequence
AT2G15760	2.132828723	4.30E-08	Arabidopsis thaliana chromosome 2, complete sequence
AT1G07135	1.594197278	4.41E-08	Arabidopsis thaliana chromosome 1 sequence
AT1G17665	1.08148685	5.09E-08	Arabidopsis thaliana uncharacterized protein mRNA, complete cds
AT2G22200	1.351154348	5.11E-08	Arabidopsis thaliana chromosome 2, complete sequence
AT5G17490	1.359202428	5.90E-08	Arabidopsis thaliana chromosome 5 sequence
AT3G27270	1.445917271	6.31E-08	Arabidopsis thaliana chromosome 3, complete sequence
AT3G09490	1.000524737	6.65E-08	Arabidopsis thaliana chromosome 3, complete sequence
AT3G22410	1.000524737	6.65E-08	Arabidopsis thaliana Sec14p-like phosphatidylinositol transfer family protein mRNA, complete cds
AT4G15270	1.952567369	6.95E-08	Arabidopsis thaliana glucosyltransferase-related protein mRNA, complete cds
AT5G38130	4.782642368	6.96E-08	Arabidopsis thaliana chromosome 5 sequence
AT5G02170	2.050575573	7.12E-08	Arabidopsis thaliana transmembrane amino acid transporter family protein mRNA, complete cds
AT1G03070	1.699653003	7.18E-08	Arabidopsis thaliana protein lifeguard 4 mRNA, complete cds
AT1G27710	5.730174948	7.30E-08	Arabidopsis thaliana chromosome 1 sequence
AT5G44260	1.595439375	7.43E-08	Arabidopsis thaliana chromosome 5 sequence
AT1G78410	1.725309193	7.81E-08	Arabidopsis thaliana chromosome 1 sequence
AT1G56600	1.050249503	7.84E-08	Arabidopsis thaliana galactinol synthase 2 mRNA, complete cds
AT5G47980	2.498849402	8.12E-08	Arabidopsis thaliana chromosome 5 sequence
AT5G02190	1.782642368	9.06E-08	Arabidopsis thaliana chromosome 5 sequence
AT1G33030	2.74787695	9.61E-08	Arabidopsis thaliana O-methyltransferase-like protein mRNA, complete cds
AT5G45000	1.442413456	1.06E-07	Arabidopsis thaliana TIR-NBS-LRR class disease resistance protein mRNA, complete cds
AT1G48500	1.442413456	1.06E-07	Arabidopsis thaliana protein TIFY 6A mRNA, complete cds
AT5G10625	1.221725341	1.11E-07	Arabidopsis thaliana chromosome 5 sequence
AT5G60250	2.025913519	1.15E-07	Arabidopsis thaliana C3H4 type zinc finger protein mRNA, complete cds
AT3G04640	2.594197278	1.18E-07	Arabidopsis thaliana chromosome 3, complete sequence

AT3G57460	1.132012949	1.19E-07	Arabidopsis thaliana catalytic/ metal ion binding / metalloendopeptidase/ zinc ion binding protein mRNA, complete cds
AT5G12340	1.331980959	1.24E-07	Arabidopsis thaliana chromosome 5 sequence
AT1G72260	5.675727164	1.28E-07	Arabidopsis thaliana thionin 2.1 mRNA, complete cds
AT5G01900	5.675727164	1.28E-07	Arabidopsis thaliana putative WRKY transcription factor 62 mRNA, complete cds
AT5G22545	5.675727164	1.28E-07	Arabidopsis thaliana chromosome 5 sequence
AT4G16563	1.053289958	1.29E-07	Arabidopsis thaliana chromosome 4 sequence
AT4G04500	2.901286864	1.29E-07	Arabidopsis thaliana cysteine-rich receptor-like protein kinase 37 mRNA, complete cds
AT4G17100	1.732717142	1.31E-07	Arabidopsis thaliana uncharacterized protein mRNA, complete cds
AT5G28913	1.065172949	1.32E-07	Arabidopsis thaliana chromosome 5 sequence
AT3G27880	1.021980681	1.65E-07	Arabidopsis thaliana chromosome 3, complete sequence
AT4G38380	1.235814996	1.78E-07	Arabidopsis thaliana MATE efflux family protein 3 mRNA, complete cds
AT2G43000	2.1182454	1.85E-07	Arabidopsis thaliana NAC domain-containing protein 42 mRNA, complete cds
AT4G28140	1.71225304	2.03E-07	Arabidopsis thaliana chromosome 4 sequence
AT1G14120	1.197679867	2.20E-07	Arabidopsis thaliana 2-oxoglutarate (2OG) and Fe(II)-dependent oxygenase-like protein mRNA, complete cds
AT3G11980	4.675727164	2.28E-07	Arabidopsis thaliana fatty acyl-CoA reductase 2 mRNA, complete cds
AT2G24220	1.083811903	2.61E-07	Arabidopsis thaliana purine permease 5 mRNA, complete cds
AT3G01960	2.238321852	2.82E-07	Arabidopsis thaliana uncharacterized protein mRNA, complete cds
AT3G55940	1.251618674	2.84E-07	Arabidopsis thaliana phosphoinositide phospholipase C 7 mRNA, complete cds
AT2G24040	1.492345882	3.00E-07	Arabidopsis thaliana Low temperature and salt responsive protein mRNA, complete cds
AT5G13330	1.492345882	3.00E-07	Arabidopsis thaliana ethylene-responsive transcription factor ERF113 mRNA, complete cds
AT3G25597	2.090764663	3.03E-07	Arabidopsis thaliana chromosome 3, complete sequence
AT1G80120	1.206067653	3.03E-07	Arabidopsis thaliana uncharacterized protein mRNA, complete cds
AT4G23310	2.02973523	3.04E-07	Arabidopsis thaliana putative cysteine-rich receptor-like protein kinase 23 mRNA, complete cds
AT3G08860	1.69149448	3.14E-07	Arabidopsis thaliana PYRIMIDINE 4 mRNA, complete cds
AT3G04220	1.210503907	3.55E-07	Arabidopsis thaliana TIR-NBS-LRR class disease resistance protein mRNA, complete cds
AT2G16367	5.560249946	3.93E-07	Arabidopsis thaliana chromosome 2, complete sequence

AT2G26020	5.560249946	3.93E-07	Arabidopsis thaliana putative defensin-like protein mRNA, complete cds
AT5G16960	2.823284352	4.01E-07	Arabidopsis thaliana zinc-binding dehydrogenase family protein mRNA, complete cds
AT4G37290	4.619143636	4.14E-07	Arabidopsis thaliana chromosome 4 sequence
AT1G12570	1.004238819	4.18E-07	Arabidopsis thaliana glucose-methanol-choline (GMC) oxidoreductase-like protein mRNA, complete cds
AT1G73270	1.074823119	4.21E-07	Arabidopsis thaliana serine carboxypeptidase-like 6 mRNA, complete cds
AT5G47330	1.369566385	4.27E-07	Arabidopsis thaliana palmitoyl protein thioesterase family protein mRNA, complete cds
AT3G61190	1.670432864	4.83E-07	Arabidopsis thaliana BON association protein 1 mRNA, complete cds
AT4G01895	2.062750287	4.94E-07	Arabidopsis thaliana chromosome 4 sequence
AT2G38790	1.275946924	5.25E-07	Arabidopsis thaliana chromosome 2, complete sequence
AT4G03070	1.249462409	5.50E-07	Arabidopsis thaliana oxidoreductase AOP1 mRNA, complete cds
AT1G67980	2.489860619	5.97E-07	Arabidopsis thaliana caffeoyl-CoA 3-O-methyltransferase mRNA, complete cds
AT3G60120	3.297215541	6.44E-07	Arabidopsis thaliana beta glucosidase 27 mRNA, complete cds
AT3G16440	1.039417783	6.46E-07	Arabidopsis thaliana myrosinase-binding protein-like protein-300B mRNA, complete cds
AT2G13810	2.367604869	6.75E-07	Arabidopsis thaliana AGD2-like defense response protein 1 mRNA, complete cds
AT5G43380	1.065485255	6.79E-07	Arabidopsis thaliana type one serine/threonine protein phosphatase 6 mRNA, complete cds
AT5G07550	5.498849402	6.93E-07	Arabidopsis thaliana glycine-rich protein 19 mRNA, complete cds
AT5G16920	5.498849402	6.93E-07	Arabidopsis thaliana chromosome 5 sequence
AT1G06350	1.289798069	7.12E-07	Arabidopsis thaliana delta-9 desaturase-like 4 protein mRNA, complete cds
AT2G34200	1.289798069	7.12E-07	Arabidopsis thaliana RING/FYVE/PHD zinc finger superfamily protein mRNA, complete cds
AT1G30135	4.560249946	7.53E-07	Arabidopsis thaliana protein TIFY 5A mRNA, complete cds
AT4G34410	4.560249946	7.53E-07	Arabidopsis thaliana chromosome 4 sequence
AT1G22570	1.041376636	9.02E-07	Arabidopsis thaliana putative peptide/nitrate transporter mRNA, complete cds
AT5G57123	1.581008507	9.17E-07	Arabidopsis thaliana chromosome 5 sequence
AT4G07960	1.384678382	9.52E-07	Arabidopsis thaliana Cellulose-synthase-like C12 mRNA, complete cds
AT5G38910	1.603318668	1.02E-06	Arabidopsis thaliana putative germin-like protein subfamily 1 member 9 mRNA, complete cds
AT5G24070	1.603318668	1.02E-06	Arabidopsis thaliana probable peroxidase 61 mRNA,

			complete cds
AT4G36830	1.521255815	1.10E-06	Arabidopsis thaliana chromosome 4 sequence
AT3G50120	2.33283945	1.14E-06	Arabidopsis thaliana uncharacterized protein mRNA, complete cds
AT5G24220	3.24830594	1.16E-06	Arabidopsis thaliana lipase class 3-related protein mRNA, complete cds
AT3G57510	3.24830594	1.16E-06	Arabidopsis thaliana polygalacturonase ADPG1 mRNA, complete cds
AT1G29060	1.030569881	1.22E-06	Arabidopsis thaliana Bet1-like protein mRNA, complete cds
AT1G69920	2.231627199	1.23E-06	Arabidopsis thaliana glutathione S-transferase TAU 12 mRNA, complete cds
AT5G55420	5.434719064	1.23E-06	Arabidopsis thaliana chromosome 5 sequence
AT5G26690	1.895852978	1.24E-06	Arabidopsis thaliana heavy-metal-associated domain-containing protein mRNA, complete cds
AT5G52930	3.675727164	1.31E-06	Arabidopsis thaliana chromosome 5 sequence
AT1G25425	1.259740835	1.46E-06	Arabidopsis thaliana chromosome 1 sequence
AT5G52720	2.560249946	1.50E-06	Arabidopsis thaliana copper transport family protein mRNA, complete cds
AT1G24575	1.105347986	1.61E-06	Arabidopsis thaliana chromosome 1 sequence
AT1G12845	1.434719064	1.65E-06	Arabidopsis thaliana chromosome 1 sequence
AT2G15830	1.390324945	1.85E-06	Arabidopsis thaliana chromosome 2, complete sequence
AT1G67920	1.390324945	1.85E-06	Arabidopsis thaliana chromosome 1 sequence
AT4G23160	3.197679867	2.09E-06	Arabidopsis thaliana cysteine-rich receptor-like protein kinase 8 mRNA, complete cds
AT4G12480	1.539188331	2.09E-06	Arabidopsis thaliana chromosome 4 sequence
AT5G45810	1.975287446	2.11E-06	Arabidopsis thaliana chromosome 5 sequence
AT1G69720	1.65809727	2.12E-06	Arabidopsis thaliana heme oxygenase 3 mRNA, complete cds
AT2G05510	1.402708669	2.12E-06	Arabidopsis thaliana glycine-rich protein mRNA, complete cds
AT5G20480	1.076570782	2.18E-06	Arabidopsis thaliana LRR receptor-like serine/threonine-protein kinase EFR mRNA, complete cds
AT5G09470	4.434719064	2.51E-06	Arabidopsis thaliana dicarboxylate carrier 3 mRNA, complete cds
AT3G04060	1.078380939	2.57E-06	Arabidopsis thaliana NAC domain containing protein 46 mRNA, complete cds
AT3G10815	2.377385889	2.95E-06	Arabidopsis thaliana RING/U-box domain-containing protein mRNA, complete cds
AT4G08230	1.080257005	3.04E-06	Arabidopsis thaliana glycine-rich protein mRNA, complete cds
AT5G61930	1.09727797	3.08E-06	Arabidopsis thaliana accumulation of photosystem one 3 mRNA, complete cds

AT4G18425	1.235154573	3.44E-06	Arabidopsis thaliana chromosome 4 sequence
AT5G01180	2.653359351	3.74E-06	Arabidopsis thaliana peptide transporter PTR5 mRNA, complete cds
AT5G37970	5.297215541	3.89E-06	Arabidopsis thaliana probable S-adenosylmethionine-dependent methyltransferase mRNA, complete cds
AT3G43250	5.297215541	3.89E-06	Arabidopsis thaliana chromosome 3, complete sequence
AT5G07530	5.297215541	3.89E-06	Arabidopsis thaliana glycine rich protein 17 mRNA, complete cds
AT1G58420	1.730174948	4.27E-06	Arabidopsis thaliana chromosome 1 sequence
AT1G18870	1.102044588	4.30E-06	Arabidopsis thaliana Isochorismate synthase 2 mRNA, complete cds
AT1G23840	1.163732535	4.31E-06	Arabidopsis thaliana chromosome 1 sequence
AT5G45960	1.121138312	4.33E-06	Arabidopsis thaliana GDSL esterase/lipase mRNA, complete cds
AT5G59580	2.477787786	4.44E-06	Arabidopsis thaliana UDP-glucosyl transferase 76E1 mRNA, complete cds
AT3G54070	2.337857525	4.98E-06	Arabidopsis thaliana ankyrin repeat-containing protein mRNA, complete cds
AT4G40010	1.192518162	5.00E-06	Arabidopsis thaliana SNF1-related protein kinase 2.7 mRNA, complete cds
AT5G37950	2.045676774	5.65E-06	Arabidopsis thaliana UDP-glycosyltransferase-like protein mRNA, complete cds
AT1G77380	1.071046429	5.82E-06	Arabidopsis thaliana amino acid permease 3 mRNA, complete cds
AT1G01680	1.456414135	6.17E-06	Arabidopsis thaliana U-box domain-containing protein 54 mRNA, complete cds
AT1G57560	1.262864036	6.44E-06	Arabidopsis thaliana myb domain protein 50 mRNA, complete cds
AT1G01250	2.607555661	6.50E-06	Arabidopsis thaliana chromosome 1 sequence
AT2G32200	2.607555661	6.50E-06	Arabidopsis thaliana uncharacterized protein mRNA, complete cds
AT3G44350	3.090764663	6.73E-06	Arabidopsis thaliana putative NAC domain-containing protein 61 mRNA, complete cds
AT5G15800	1.203093363	6.90E-06	Arabidopsis thaliana MADS box transcription factor SEPALLATA 1 mRNA, complete cds
AT5G62040	5.223214959	6.97E-06	Arabidopsis thaliana protein BROTHER of FT and TFL 1 mRNA, complete cds
AT3G52130	5.223214959	6.97E-06	Arabidopsis thaliana chromosome 3, complete sequence
AT2G43580	5.223214959	6.97E-06	Arabidopsis thaliana chitinase family protein mRNA, complete cds
AT3G05920	1.109936972	7.07E-06	Arabidopsis thaliana heavy-metal-associated domain-containing protein mRNA, complete cds
AT2G44340	1.418894097	7.98E-06	Arabidopsis thaliana chromosome 2, complete sequence

AT5G50760	1.829436579	8.27E-06	Arabidopsis thaliana chromosome 5 sequence
AT1G13130	2.297215541	8.40E-06	Arabidopsis thaliana Cellulase (glycosyl hydrolase family 5) protein mRNA, complete cds
AT4G01670	2.010911355	9.20E-06	Arabidopsis thaliana uncharacterized protein mRNA, complete cds
AT3G48520	2.010911355	9.20E-06	Arabidopsis thaliana chromosome 3, complete sequence
AT5G19100	1.095581679	9.76E-06	Arabidopsis thaliana chromosome 5 sequence
AT4G24110	1.675727164	1.03E-05	Arabidopsis thaliana chromosome 4 sequence
AT3G11580	1.675727164	1.03E-05	Arabidopsis thaliana AP2/B3-like transcriptional factor family protein mRNA, complete cds
AT5G15240	1.252821421	1.07E-05	Arabidopsis thaliana transmembrane amino acid transporter family protein mRNA, complete cds
AT1G33900	1.22104386	1.11E-05	Arabidopsis thaliana P-loop containing nucleoside triphosphate hydrolases superfamily protein mRNA, complete cds
AT4G30430	2.560249946	1.13E-05	Arabidopsis thaliana tetraspanin9 mRNA, complete cds
AT2G35480	1.192098835	1.14E-05	Arabidopsis thaliana uncharacterized protein mRNA, complete cds
AT3G20660	1.141297397	1.17E-05	Arabidopsis thaliana organic cation/carnitine transporter4 mRNA, complete cds
AT3G59900	1.141297397	1.17E-05	Arabidopsis thaliana chromosome 3, complete sequence
AT4G23496	3.034181135	1.21E-05	Arabidopsis thaliana protein SPIRAL1-like5 mRNA, complete cds
AT5G07230	5.145212447	1.25E-05	Arabidopsis thaliana Tapetum-specific protein A9 mRNA, complete cds
AT3G21500	5.145212447	1.25E-05	Arabidopsis thaliana 1-deoxy-D-xylulose 5-phosphate synthase 1 mRNA, complete cds
AT2G03740	5.145212447	1.25E-05	Arabidopsis thaliana late embryogenesis abundant domain-containing protein mRNA, complete cds
AT1G71390	5.145212447	1.25E-05	Arabidopsis thaliana chromosome 1 sequence
AT1G75910	5.145212447	1.25E-05	Arabidopsis thaliana extracellular lipase 4 mRNA, complete cds
AT2G26010	5.145212447	1.25E-05	Arabidopsis thaliana plant defensin 1.3 mRNA, complete cds
AT2G14290	5.145212447	1.25E-05	Arabidopsis thaliana chromosome 2, complete sequence
AT3G21780	2.390324945	1.29E-05	Arabidopsis thaliana chromosome 3, complete sequence
AT5G53820	2.390324945	1.29E-05	Arabidopsis thaliana Late embryogenesis abundant protein (LEA) family protein mRNA, complete cds
AT1G05020	1.489860619	1.36E-05	Arabidopsis thaliana chromosome 1 sequence
AT5G61890	2.255395365	1.41E-05	Arabidopsis thaliana ethylene-responsive transcription factor ERF114 mRNA, complete cds
AT1G74870	2.145212447	1.48E-05	Arabidopsis thaliana RING-finger domain-containing protein mRNA, complete cds

AT4G17785	2.053289958	1.51E-05	Arabidopsis thaliana transcription factor MYB39 mRNA, complete cds
AT5G40000	2.730174948	1.60E-05	Arabidopsis thaliana chromosome 5 sequence
AT3G57540	1.085711435	1.88E-05	Arabidopsis thaliana Remorin family protein mRNA, complete cds
AT5G36910	2.511340346	1.94E-05	Arabidopsis thaliana thionin 2.2 mRNA, complete cds
AT1G21540	1.249909826	2.10E-05	Arabidopsis thaliana probable acyl-activating enzyme 9 mRNA, complete cds
AT2G40740	2.344521255	2.20E-05	Arabidopsis thaliana WRKY transcription factor 55 mRNA, complete cds
AT3G10110	1.109588537	2.25E-05	Arabidopsis thaliana mitochondrial import inner membrane translocase subunit TIM22-1 mRNA, complete cds
AT5G59260	1.132828723	2.26E-05	Arabidopsis thaliana chromosome 5 sequence
AT5G57510	1.93876157	2.41E-05	Arabidopsis thaliana chromosome 5 sequence
AT4G18170	1.258221409	2.46E-05	Arabidopsis thaliana WRKY DNA-binding protein 28 mRNA, complete cds
AT3G52820	1.223214959	2.55E-05	Arabidopsis thaliana purple acid phosphatase 22 mRNA, complete cds
AT3G56030	1.112790969	2.66E-05	Arabidopsis thaliana chromosome 3, complete sequence
AT1G66160	1.003301822	2.71E-05	Arabidopsis thaliana chromosome 1 sequence
AT2G30432	2.675727164	2.81E-05	Arabidopsis thaliana protein trichomeless 1 mRNA, complete cds
AT3G15270	1.197679867	3.07E-05	Arabidopsis thaliana squamosa promoter-binding-like protein 5 mRNA, complete cds
AT4G19720	1.167932524	3.13E-05	Arabidopsis thaliana Glycosyl hydrolase family protein with chitinase insertion domain mRNA, complete cds
AT3G02240	1.167932524	3.13E-05	Arabidopsis thaliana root meristem growth factor 7 mRNA, complete cds
AT2G32020	1.734279346	3.22E-05	Arabidopsis thaliana chromosome 2, complete sequence
AT2G26380	2.460714273	3.34E-05	Arabidopsis thaliana chromosome 2, complete sequence
AT4G37050	2.460714273	3.34E-05	Arabidopsis thaliana PATATIN-like protein 4 mRNA, complete cds
AT3G15800	1.204106136	3.61E-05	Arabidopsis thaliana glycosyl hydrolase superfamily protein mRNA, complete cds
AT5G05880	1.096302847	3.67E-05	Arabidopsis thaliana UDP-glycosyltransferase 76C4 mRNA, complete cds
AT2G07671	1.837783922	3.70E-05	Arabidopsis thaliana ecotype Col-0 mitochondrion, complete genome
AT3G51400	2.913886901	3.85E-05	Arabidopsis thaliana chromosome 3, complete sequence
AT5G07700	2.913886901	3.85E-05	Arabidopsis thaliana myb domain protein 76 mRNA, complete cds
AT5G24180	1.901286864	3.88E-05	Arabidopsis thaliana lipase class 3-related protein mRNA, complete cds

AT3G10185	2.167932524	3.94E-05	Arabidopsis thaliana Gibberellin-regulated family protein mRNA, complete cds
AT4G23030	2.062750287	4.03E-05	Arabidopsis thaliana chromosome 4 sequence
AT5G24540	4.975287446	4.11E-05	Arabidopsis thaliana beta glucosidase 31 mRNA, complete cds
AT5G55150	4.975287446	4.11E-05	Arabidopsis thaliana uncharacterized protein mRNA, complete cds
AT3G15536	4.975287446	4.11E-05	Arabidopsis thaliana chromosome 3, complete sequence
AT1G59950	4.975287446	4.11E-05	Arabidopsis thaliana putative aldo/keto reductase mRNA, complete cds
AT2G19070	4.975287446	4.11E-05	Arabidopsis thaliana spermidine hydroxycinnamoyl transferase mRNA, complete cds
AT4G29110	1.099276163	4.34E-05	Arabidopsis thaliana chromosome 4 sequence
AT5G01015	1.099276163	4.34E-05	Arabidopsis thaliana uncharacterized protein mRNA, complete cds
AT2G45570	1.505802162	4.46E-05	Arabidopsis thaliana cytochrome P450 76C2 mRNA, complete cds
AT1G03020	1.415860037	4.47E-05	Arabidopsis thaliana chromosome 1 sequence
AT4G05100	1.65978562	4.59E-05	Arabidopsis thaliana myb domain protein 74 mRNA, complete cds
AT1G26420	1.058703454	4.96E-05	Arabidopsis thaliana chromosome 1 sequence
AT2G30760	4.062750287	5.27E-05	Arabidopsis thaliana uncharacterized protein mRNA, complete cds
AT3G29035	1.040875787	5.72E-05	Arabidopsis thaliana NAC domain-containing protein 3 mRNA, complete cds
AT1G21910	1.040875787	5.72E-05	Arabidopsis thaliana chromosome 1 sequence
AT5G50790	1.862812716	6.22E-05	Arabidopsis thaliana bidirectional sugar transporter SWEET10 mRNA, complete cds
AT5G05300	2.24830594	6.27E-05	Arabidopsis thaliana chromosome 5 sequence
AT3G60540	1.934645461	6.47E-05	Arabidopsis thaliana chromosome 3, complete sequence
AT5G22530	1.934645461	6.47E-05	Arabidopsis thaliana chromosome 5 sequence
AT1G54970	2.122128834	6.55E-05	Arabidopsis thaliana proline-rich protein 1 mRNA, complete cds
AT4G16000	1.390324945	6.59E-05	Arabidopsis thaliana chromosome 4 sequence
AT2G29165	4.882178041	7.49E-05	Arabidopsis thaliana chromosome 2, complete sequence
AT4G13395	4.882178041	7.49E-05	Arabidopsis thaliana chromosome 4 sequence
AT1G33760	4.882178041	7.49E-05	Arabidopsis thaliana chromosome 1 sequence
AT2G44070	4.882178041	7.49E-05	Arabidopsis thaliana NagB/RpiA/CoA transferase-like superfamily protein mRNA, complete cds
AT4G15975	1.408246853	7.59E-05	Arabidopsis thaliana chromosome 4 sequence
AT2G18660	1.140346692	8.53E-05	Arabidopsis thaliana plant natriuretic peptide A mRNA, complete cds
AT4G25950	3.223214959	8.77E-05	Arabidopsis thaliana V-type proton ATPase subunit G3

			mRNA, complete cds
AT2G34315	2.353799069	9.80E-05	Arabidopsis thaliana avirulence induced family protein mRNA, complete cds
AT3G48790	2.353799069	9.80E-05	Arabidopsis thaliana Pyridoxal phosphate (PLP)-dependent transferases superfamily protein mRNA, complete cds
AT1G51380	1.090764663	9.95E-05	Arabidopsis thaliana DEAD-box ATP-dependent RNA helicase 34 mRNA, complete cds
AT1G72660	2.074823119	0.000108265	Arabidopsis thaliana developmentally regulated G-protein 2 mRNA, complete cds
AT5G08030	2.782642368	0.0001218	Arabidopsis thaliana glycerophosphoryl diester phosphodiesterase mRNA, complete cds
AT5G47990	1.009234778	0.000125641	Arabidopsis thaliana cytochrome P450 705A5 mRNA, complete cds
AT3G44830	4.782642368	0.000137319	Arabidopsis thaliana putative phospholipid:diacylglycerol acyltransferase 2 mRNA, complete cds
AT5G15290	4.782642368	0.000137319	Arabidopsis thaliana casparian strip membrane protein 5 mRNA, complete cds
AT1G61110	4.782642368	0.000137319	Arabidopsis thaliana NAC domain containing protein 25 mRNA, complete cds
AT1G20150	4.782642368	0.000137319	Arabidopsis thaliana subtilisin-like serine endopeptidase family protein mRNA, complete cds
AT5G13380	4.782642368	0.000137319	Arabidopsis thaliana auxin-responsive GH3 family protein mRNA, complete cds
AT3G57157	1.190300337	0.000138629	Arabidopsis thaliana chromosome 3, complete sequence
AT1G52855	1.725309193	0.000145718	Arabidopsis thaliana chromosome 1 sequence
AT3G27884	1.527828469	0.000147292	Arabidopsis thaliana clone 102688 mRNA sequence
AT4G28460	2.498849402	0.000148719	Arabidopsis thaliana chromosome 4 sequence
AT4G17680	1.010052864	0.000149119	Arabidopsis thaliana SBP (S-ribonuclease binding protein) family protein mRNA, complete cds
AT1G18265	1.284615504	0.000151424	Arabidopsis thaliana chromosome 1 sequence
AT5G24655	1.782642368	0.000157333	Arabidopsis thaliana chromosome 5 sequence
AT5G54165	3.145212447	0.000159299	Arabidopsis thaliana chromosome 5 sequence
AT4G33467	3.145212447	0.000159299	Arabidopsis thaliana uncharacterized protein mRNA, complete cds
AT1G07150	1.197679867	0.000163279	Arabidopsis thaliana mitogen-activated protein kinase kinase kinase 13 mRNA, complete cds

AT4G33960	1.100818328	0.000165216	Arabidopsis thaliana chromosome 4 sequence
AT3G27250	1.560249946	0.000166016	Arabidopsis thaliana chromosome 3, complete sequence
AT4G21970	1.849756564	0.000167375	Arabidopsis thaliana uncharacterized protein mRNA, complete cds
AT5G48400	1.929483756	0.000174766	Arabidopsis thaliana glutamate receptor 1.2 mRNA, complete cds
AT3G28740	1.929483756	0.000174766	Arabidopsis thaliana cytochrome P450 CYP81D11 mRNA, complete cds
AT2G31980	1.929483756	0.000174766	Arabidopsis thaliana cysteine proteinase inhibitor 2 mRNA, complete cds
AT2G35550	2.145212447	0.000176006	Arabidopsis thaliana basic pentacysteine 7 mRNA, complete cds
AT5G54470	1.297215541	0.000176858	Arabidopsis thaliana B-box type zinc finger-containing protein mRNA, complete cds
AT1G29290	2.025913519	0.000178165	Arabidopsis thaliana chromosome 1 sequence
AT3G09640	3.882178041	0.000180611	Arabidopsis thaliana L-ascorbate peroxidase 2 mRNA, complete cds
AT1G34180	1.24830594	0.000186059	Arabidopsis thaliana NAC domain containing protein 16 mRNA, complete cds
AT1G01480	1.467140542	0.000195333	Arabidopsis thaliana 1-aminocyclopropane-1-carboxylate synthase 2 mRNA, complete cds
AT5G60730	1.104570463	0.000195659	Arabidopsis thaliana Anion-transporting ATPase mRNA, complete cds
AT3G55840	1.134486041	0.000196708	Arabidopsis thaliana chromosome 3, complete sequence
AT4G23700	1.638252458	0.000206792	Arabidopsis thaliana cation/H(+) antiporter 17 mRNA, complete cds
AT3G10930	1.494661605	0.000222572	Arabidopsis thaliana chromosome 3, complete sequence
AT2G30640	1.080640446	0.000228657	Arabidopsis thaliana chromosome 2, complete sequence
AT1G49960	1.080640446	0.000228657	Arabidopsis thaliana nucleobase-ascorbate transporter 4 mRNA, complete cds
AT5G24200	1.740822192	0.00024841	Arabidopsis thaliana uncharacterized protein mRNA, complete cds
AT1G51780	4.675727164	0.000252922	Arabidopsis thaliana IAA-amino acid hydrolase ILR1-like 5 mRNA, complete cds
AT1G69500	4.675727164	0.000252922	Arabidopsis thaliana cytochrome P450, family 704, subfamily B, polypeptide 1 mRNA, complete cds
AT1G57850	4.675727164	0.000252922	Arabidopsis thaliana Toll-Interleukin-Resistance domain-containing protein mRNA, complete cds

AT5G43935	4.675727164	0.000252922	Arabidopsis thaliana flavonol synthase 6 mRNA, complete cds
AT5G48210	4.675727164	0.000252922	Arabidopsis thaliana chromosome 5 sequence
AT4G15200	4.675727164	0.000252922	Arabidopsis thaliana formin 3 mRNA, complete cds
AT5G24640	4.675727164	0.000252922	Arabidopsis thaliana chromosome 5 sequence
AT2G29110	1.805362444	0.000267098	Arabidopsis thaliana glutamate receptor 2.8 mRNA, complete cds
AT1G61480	1.805362444	0.000267098	Arabidopsis thaliana G-type lectin S-receptor-like serine/threonine-protein kinase mRNA, complete cds
AT3G30340	1.083811903	0.00027112	Arabidopsis thaliana nodulin MtN21 /EamA-like transporter family protein mRNA, complete cds
AT1G73965	2.238321852	0.000282232	Arabidopsis thaliana chromosome 1 sequence
AT1G21326	2.238321852	0.000282232	Arabidopsis thaliana chromosome 1 sequence
AT1G78440	2.238321852	0.000282232	Arabidopsis thaliana gibberellin 2-beta-dioxygenase 1 mRNA, complete cds
AT5G25910	2.238321852	0.000282232	Arabidopsis thaliana receptor like protein 52 mRNA, complete cds
AT1G09950	1.882178041	0.000282271	Arabidopsis thaliana chromosome 1 sequence
AT2G37720	3.062750287	0.000289038	Arabidopsis thaliana trichome birefringence-like 15 protein mRNA, complete cds
ATCG00080	1.975287446	0.000291727	Cardamine resedifolia plastid, complete genome
AT1G66860	1.975287446	0.000291727	Arabidopsis thaliana class I glutamine amidotransferase-like domain-containing protein mRNA, complete cds
AT5G10570	2.090764663	0.000292745	Arabidopsis thaliana transcription factor bHLH61 mRNA, complete cds
AT3G02480	2.090764663	0.000292745	Arabidopsis thaliana Late embryogenesis abundant protein (LEA) family protein mRNA, complete cds
AT1G47890	2.090764663	0.000292745	Arabidopsis thaliana chromosome 1 sequence
AT2G20562	1.283409741	0.00029908	Arabidopsis thaliana uncharacterized protein mRNA, complete cds
AT5G25390	3.782642368	0.000335412	Arabidopsis thaliana ethylene-responsive transcription factor SHINE 3 mRNA, complete cds
AT2G20800	3.782642368	0.000335412	Arabidopsis thaliana NAD(P)H dehydrogenase B4 mRNA, complete cds
AT2G07723	1.297215541	0.00034962	Arabidopsis thaliana ecotype Col-0 mitochondrion,

		2	complete genome
AT4G26960	1.062750287	0.000374166	Arabidopsis thaliana uncharacterized protein mRNA, complete cds
AT4G27400	1.379677701	0.000375957	Arabidopsis thaliana late embryogenesis abundant protein-like protein mRNA, complete cds
AT2G46860	1.379677701	0.000375957	Arabidopsis thaliana pyrophosphorylase 3 mRNA, complete cds
AT2G29480	2.638252458	0.000380765	Arabidopsis thaliana glutathione S-transferase tau 2 mRNA, complete cds
AT4G24000	1.489860619	0.000381858	Arabidopsis thaliana cellulose synthase-like protein G2 mRNA, complete cds
AT3G02310	1.489860619	0.000381858	Arabidopsis thaliana developmental protein SEPALLATA 2 mRNA, complete cds
AT1G08630	1.69775347	0.00039012	Arabidopsis thaliana threonine aldolase mRNA, complete cds
AT3G23230	1.522775241	0.000433613	Arabidopsis thaliana chromosome 3, complete sequence
AT2G29710	1.4015522	0.000434423	Arabidopsis thaliana chromosome 2, complete sequence
AT1G62981	1.206612992	0.000446607	Arabidopsis thaliana uncharacterized protein mRNA, complete cds
AT1G56250	1.206612992	0.000446607	Arabidopsis thaliana F-box protein PP2-B14 mRNA, complete cds
AT2G39500	1.206612992	0.000446607	Arabidopsis thaliana uncharacterized protein mRNA, complete cds
AT2G33710	1.833268441	0.000453451	Arabidopsis thaliana ethylene-responsive transcription factor ERF112 mRNA, complete cds
AT3G54510	1.833268441	0.000453451	Arabidopsis thaliana Early-responsive to dehydration stress protein (ERD4) mRNA, complete cds
AT5G20240	1.16432127	0.000454431	Arabidopsis thaliana Floral homeotic protein PISTILLATA mRNA, complete cds
AT1G71160	4.560249946	0.000468406	Arabidopsis thaliana chromosome 1 sequence
AT1G56360	4.560249946	0.000468406	Arabidopsis thaliana purple acid phosphatase 6 mRNA, complete cds
AT4G23340	1.3289244	0.000475786	Arabidopsis thaliana chromosome 4 sequence
AT1G10530	1.3289244	0.000475786	Arabidopsis thaliana uncharacterized protein mRNA, complete cds
AT5G11210	2.034181135	0.000484393	Arabidopsis thaliana glutamate receptor 2.5 mRNA, complete cds
AT2G30942	1.425948855	0.00050048	Arabidopsis thaliana uncharacterized protein mRNA, complete cds
AT5G41730	2.975287446	0.00052347	Arabidopsis thaliana protein kinase mRNA, complete cds

		8	
AT1G76610	2.975287446	0.00052347 8	Arabidopsis thaliana chromosome 1 sequence
AT5G44005	1.216295545	0.00052613 4	Arabidopsis thaliana chromosome 5 sequence
AT4G11350	1.098669861	0.00053647 6	Arabidopsis thaliana uncharacterized protein mRNA, complete cds
AT5G51950	1.603318668	0.00054853 5	Arabidopsis thaliana Glucose-methanol-choline (GMC) oxidoreductase family protein mRNA, complete cds
AT1G54095	1.453334743	0.00057453 1	Arabidopsis thaliana uncharacterized protein mRNA, complete cds
ATCG00280	1.018356168	0.00059172 8	Arabidopsis thaliana chloroplast DNA, complete genome, ecotype: Columbia
AT3G55700	1.018356168	0.00059172 8	Arabidopsis thaliana UDP-glycosyltransferase 76F1 mRNA, complete cds
AT3G32047	1.653359351	0.00060923 7	Arabidopsis thaliana cytochrome P450 superfamily protein mRNA, complete cds
AT5G41663	3.675727164	0.00062401 2	Arabidopsis thaliana chromosome 5 sequence
AT1G35310	3.675727164	0.00062401 2	Arabidopsis thaliana MLP-like protein 168 mRNA, complete cds
AT3G10320	1.071502761	0.00062650 7	Arabidopsis thaliana Glycosyltransferase family 61 protein mRNA, complete cds
AT1G66440	1.367604869	0.00064278 8	Arabidopsis thaliana chromosome 1 sequence
AT2G25440	1.71225304	0.00066907 6	Arabidopsis thaliana receptor like protein 20 mRNA, complete cds
AT5G54490	1.71225304	0.00066907 6	Arabidopsis thaliana chromosome 5 sequence
AT1G14100	2.560249946	0.00066961 7	Arabidopsis thaliana fucosyltransferase 8 mRNA, complete cds
AT1G05100	1.782642368	0.00072428	Arabidopsis thaliana chromosome 1 sequence
AT1G56150	1.074823119	0.00074423 1	Arabidopsis thaliana chromosome 1 sequence
AT1G09480	1.519607962	0.00074677	Arabidopsis thaliana alcohol dehydrogenase-like protein mRNA, complete cds
AT3G13437	1.145212447	0.00075749 3	Arabidopsis thaliana uncharacterized protein mRNA, complete cds
AT1G51620	1.145212447	0.00075749 3	Arabidopsis thaliana protein kinase superfamily protein mRNA, complete cds
AT2G07719	2.297215541	0.00075884 6	Arabidopsis thaliana ecotype Landsberg erecta mitochondrion, complete genome
AT1G65240	2.297215541	0.00075884 6	Arabidopsis thaliana aspartyl protease family protein mRNA, complete cds

AT2G43390	1.975287446	0.000797047	Arabidopsis thaliana chromosome 2, complete sequence
AT2G41850	2.112790969	0.000797408	Arabidopsis thaliana polygalacturonase ADPG2 mRNA, complete cds
AT1G49370	2.112790969	0.000797408	Arabidopsis thaliana chromosome 1 sequence
AT4G01975	2.112790969	0.000797408	Arabidopsis thaliana chromosome 4 sequence
AT1G52270	2.112790969	0.000797408	Arabidopsis thaliana chromosome 1 sequence
AT3G51642	2.112790969	0.000797408	Arabidopsis thaliana chromosome 3, complete sequence
AT3G49190	1.560249946	0.000843763	Arabidopsis thaliana O-acyltransferase (WSD1-like) family protein mRNA, complete cds
AT4G38410	1.250921888	0.000856981	Arabidopsis thaliana putative dehydrin mRNA, complete cds
AT1G75790	4.434719064	0.000872509	Arabidopsis thaliana SKU5 similar 18 protein mRNA, complete cds
AT5G59590	4.434719064	0.000872509	Arabidopsis thaliana UDP-glucosyl transferase 76E2 mRNA, complete cds
AT5G05220	4.434719064	0.000872509	Arabidopsis thaliana chromosome 5 sequence
AT4G29980	4.434719064	0.000872509	Arabidopsis thaliana chromosome 4 sequence
AT4G22460	1.112790969	0.000895988	Arabidopsis thaliana chromosome 4 sequence
AT1G61830	1.607555661	0.000945808	Arabidopsis thaliana chromosome 1 sequence
AT1G71520	2.882178041	0.00094595	Arabidopsis thaliana chromosome 1 sequence
AT5G26630	2.882178041	0.00094595	Arabidopsis thaliana chromosome 5 sequence
AT2G47190	1.444772729	0.000988894	Arabidopsis thaliana mRNA for MYB transcription factor, complete cds, clone: RAFL16-42-L19
AT1G30845	1.444772729	0.000988894	Arabidopsis thaliana uncharacterized protein mRNA, complete cds
AT1G50270	1.022593161	0.000996376	Arabidopsis thaliana chromosome 1 sequence
AT3G48640	1.663343439	0.001049604	Arabidopsis thaliana chromosome 3, complete sequence
AT1G66830	1.663343439	0.001049604	Arabidopsis thaliana probable inactive leucine-rich repeat receptor-like protein kinase mRNA, complete cds
AT1G33960	1.159712017	0.001062105	Arabidopsis thaliana protein AIG1 mRNA, complete cds
AT4G34380	1.353799069	0.001101084	Arabidopsis thaliana chromosome 4 sequence

AT2G31180	1.219213029	0.001231878	Arabidopsis thaliana myb domain protein 14 mRNA, complete cds
AT3G28600	1.811788713	0.001238444	Arabidopsis thaliana chromosome 3, complete sequence
AT3G15500	1.086318758	0.001248983	Arabidopsis thaliana ATAF-like NAC-domain transcription factor mRNA, complete cds
AT3G17890	1.167932524	0.001257374	Arabidopsis thaliana uncharacterized protein mRNA, complete cds
AT1G56320	1.124150832	0.001261744	Arabidopsis thaliana uncharacterized protein mRNA, complete cds
AT3G05400	1.515855827	0.001290004	Arabidopsis thaliana sugar transporter ERD6-like 12 mRNA, complete cds
AT2G15780	2.223214959	0.001293636	Arabidopsis thaliana Cupredoxin superfamily protein mRNA, complete cds
AT3G56275	1.913886901	0.001303649	Arabidopsis thaliana chromosome 3, complete sequence
AT1G65570	2.045676774	0.001329129	Arabidopsis thaliana polygalacturonase family protein mRNA, complete cds
AT2G43870	2.045676774	0.001329129	Arabidopsis thaliana putative polygalacturonase /pectinase mRNA, complete cds
AT3G54340	1.297215541	0.001383932	Arabidopsis thaliana Floral homeotic protein APETALA 3 mRNA, complete cds
AT1G44010	1.560249946	0.00145918	Arabidopsis thaliana uncharacterized protein mRNA, complete cds
AT5G39680	1.560249946	0.00145918	Arabidopsis thaliana chromosome 5 sequence
AT4G14815	4.297215541	0.001635238	Arabidopsis thaliana bifunctional inhibitor/lipid-transfer protein/seed storage 2S albumin-like protein mRNA, complete cds
AT1G47980	4.297215541	0.001635238	Arabidopsis thaliana uncharacterized protein mRNA, complete cds
AT2G30830	4.297215541	0.001635238	Arabidopsis thaliana 2-oxoglutarate dependent oxygenase-like protein mRNA, complete cds
AT5G61650	4.297215541	0.001635238	Arabidopsis thaliana CYCLIN P4;2 mRNA, complete cds
AT5G43340	4.297215541	0.001635238	Arabidopsis thaliana chromosome 5 sequence
AT1G66550	4.297215541	0.001635238	Arabidopsis thaliana putative WRKY transcription factor 67 mRNA, complete cds
AT5G52400	4.297215541	0.001635238	Arabidopsis thaliana cytochrome P450, family 715, subfamily A, polypeptide 1 mRNA, complete cds
AT3G60710	4.297215541	0.001635238	Arabidopsis thaliana putative FBD-associated F-box protein mRNA, complete cds
AT1G66380	4.297215541	0.001635238	Arabidopsis thaliana transcription factor MYB114 mRNA, complete cds

AT5G03204	4.297215541	0.001635238	Arabidopsis thaliana chromosome 5 sequence
AT5G45830	4.297215541	0.001635238	Arabidopsis thaliana protein DELAY OF GERMINATION 1 mRNA, complete cds
AT1G55230	4.297215541	0.001635238	Arabidopsis thaliana chromosome 1 sequence
AT5G09980	1.612717366	0.001636092	Arabidopsis thaliana elicitor peptide 4 mRNA, complete cds
AT1G03580	2.782642368	0.001704722	Arabidopsis thaliana chromosome 1 sequence
AT3G45280	2.782642368	0.001704722	Arabidopsis thaliana syntaxin-72 mRNA, complete cds
AT5G67310	2.782642368	0.001704722	Arabidopsis thaliana cytochrome P450, family 81, subfamily G, polypeptide 1 mRNA, complete cds
AT3G17110	2.782642368	0.001704722	Arabidopsis thaliana chromosome 3, complete sequence
AT1G15870	1.095581679	0.001765555	Arabidopsis thaliana mitochondrial glycoprotein family protein mRNA, complete cds
AT1G06980	1.675727164	0.001813669	Arabidopsis thaliana chromosome 1 sequence
AT2G25820	1.675727164	0.001813669	Arabidopsis thaliana chromosome 2, complete sequence
AT1G34050	1.675727164	0.001813669	Arabidopsis thaliana ankyrin repeats-containing protein mRNA, complete cds
AT3G47830	1.337857525	0.001890192	Arabidopsis thaliana DNA glycosylase superfamily protein mRNA, complete cds
AT4G12490	1.752895024	0.001980399	Arabidopsis thaliana chromosome 4 sequence
AT5G53980	1.752895024	0.001980399	Arabidopsis thaliana chromosome 5 sequence
AT2G47950	1.752895024	0.001980399	Arabidopsis thaliana chromosome 2, complete sequence
AT5G39100	1.752895024	0.001980399	Arabidopsis thaliana germin-like protein 6 mRNA, complete cds
AT2G16910	1.752895024	0.001980399	Arabidopsis thaliana transcription factor ABORTED MICROSPORES mRNA, complete cds
AT4G03540	2.390324945	0.00204377	Arabidopsis thaliana uncharacterized protein mRNA, complete cds
AT1G08440	2.390324945	0.00204377	Arabidopsis thaliana aluminum-activated malate transporter 2 mRNA, complete cds
AT3G02430	2.390324945	0.00204377	Arabidopsis thaliana chromosome 3, complete sequence
AT1G30370	1.062750287	0.002062878	Arabidopsis thaliana chromosome 1 sequence
AT4G27290	1.100818328	0.00209976	Arabidopsis thaliana G-type lectin S-receptor-like

		3	serine/threonine-protein kinase mRNA, complete cds
AT4G23610	1.849756564	0.002118477	Arabidopsis thaliana chromosome 4 sequence
AT1G28920	1.849756564	0.002118477	Theobroma cacao Uncharacterized protein (TCM_007238) mRNA, complete cds
AT1G26310	3.434719064	0.002170165	Arabidopsis thaliana transcription factor CAULIFLOWER mRNA, complete cds
AT5G15430	2.145212447	0.002192593	Arabidopsis thaliana chromosome 5 sequence
AT2G31310	2.145212447	0.002192593	Arabidopsis thaliana LOB domain-containing protein 14 mRNA, complete cds
AT1G49900	2.145212447	0.002192593	Arabidopsis thaliana C2H2 type zinc finger transcription factor-like protein mRNA, complete cds
AT1G70640	2.145212447	0.002192593	Arabidopsis thaliana octicosapeptide/Phox/Bem1p (PB1) domain-containing protein mRNA, complete cds
AT1G53100	1.975287446	0.002201552	Arabidopsis thaliana core-2/I-branching beta-1,6-N-acetylglucosaminyltransferase-like protein mRNA, complete cds
AT3G04530	1.209752699	0.002462532	Arabidopsis thaliana phosphoenolpyruvate carboxylase kinase 2 mRNA, complete cds
AT5G55340	1.209752699	0.002462532	Arabidopsis thaliana chromosome 5 sequence
AT4G16820	1.560249946	0.002533122	Arabidopsis thaliana chromosome 4 sequence
AT2G07677	1.560249946	0.002533122	Arabidopsis thaliana ecotype Col-0 mitochondrion, complete genome
AT1G63580	1.560249946	0.002533122	Arabidopsis thaliana receptor-like protein kinase-related family protein mRNA, complete cds
AT2G07698	1.297215541	0.002776674	Arabidopsis thaliana ATPase, F1 complex, alpha subunit protein mRNA, complete cds
AT5G65600	1.297215541	0.002776674	Arabidopsis thaliana chromosome 5 sequence
AT2G01275	1.297215541	0.002776674	Arabidopsis thaliana RING/FYVE/PHD zinc finger-containing protein mRNA, complete cds
AT4G15236	1.619143636	0.002841064	Arabidopsis thaliana ABC transporter G family member 43 mRNA, complete cds
AT2G19050	1.619143636	0.002841064	Arabidopsis thaliana GDSL esterase/lipase mRNA, complete cds
AT5G48410	1.619143636	0.002841064	Arabidopsis thaliana glutamate receptor 1.3 mRNA, complete cds
AT2G17470	1.619143636	0.002841064	Arabidopsis thaliana aluminum-activated malate transporter 6 mRNA, complete cds
AT2G30424	1.034181135	0.002850691	Arabidopsis thaliana protein trichomeless 2 mRNA, complete cds

AT1G17020	1.034181135	0.002850691	Arabidopsis thaliana Fe(II)/ascorbate oxidase family protein SRG1 mRNA, complete cds
AT1G05065	1.223214959	0.002909627	Arabidopsis thaliana chromosome 1 sequence
AT1G07550	1.112790969	0.002971594	Arabidopsis thaliana putative LRR receptor-like serine/threonine-protein kinase mRNA, complete cds
AT1G43765	2.675727164	0.003061992	Arabidopsis thaliana clone 103892 mRNA sequence
AT1G19750	2.675727164	0.003061992	Arabidopsis thaliana UV-B response protein-like protein CSAat1b mRNA, complete cds
AT1G12030	2.675727164	0.003061992	Arabidopsis thaliana uncharacterized protein mRNA, complete cds
AT4G05170	2.675727164	0.003061992	Arabidopsis thaliana transcription factor bHLH114 mRNA, complete cds
AT5G62320	4.145212447	0.003084793	Arabidopsis thaliana myb domain protein 99 mRNA, complete cds
AT1G02580	4.145212447	0.003084793	Arabidopsis thaliana histone-lysine N-methyltransferase MEDEA mRNA, complete cds
ATMG01120	4.145212447	0.003084793	Arabidopsis thaliana ecotype Columbia NADH dehydrogenase subunit 1 mRNA, partial cds; mitochondrial
AT1G44224	4.145212447	0.003084793	Arabidopsis thaliana chromosome 1 sequence
AT1G01280	4.145212447	0.003084793	Arabidopsis thaliana cytochrome P450, family 703, subfamily A, polypeptide 2 mRNA, complete cds
ATMG01020	4.145212447	0.003084793	Arabidopsis thaliana ecotype Landsberg erecta mitochondrion, complete genome
AT2G22760	4.145212447	0.003084793	Arabidopsis thaliana transcription factor bHLH19 mRNA, complete cds
AT5G02330	4.145212447	0.003084793	Arabidopsis thaliana chromosome 5 sequence
ATMG01190	4.145212447	0.003084793	Arabidopsis thaliana ecotype Landsberg erecta mitochondrion, complete genome
AT4G19970	1.69149448	0.003144787	Arabidopsis thaliana uncharacterized protein mRNA, complete cds
AT2G18000	1.003856598	0.003280627	Arabidopsis thaliana TBP-associated factor 14 mRNA, complete cds
AT1G24735	1.460714273	0.003403933	Arabidopsis thaliana S-adenosyl-L-methionine-dependent methyltransferases superfamily protein mRNA, complete cds
AT3G44700	1.782642368	0.00341858	Arabidopsis thaliana uncharacterized protein mRNA, complete cds
AT4G04510	1.782642368	0.00341858	Arabidopsis thaliana cysteine-rich receptor-like protein kinase 38 mRNA, complete cds

AT1G52690	1.782642368	0.00341858	Arabidopsis thaliana Late embryogenesis abundant protein (LEA) family protein mRNA, complete cds
AT3G14510	1.782642368	0.00341858	Arabidopsis thaliana putative geranylgeranyl pyrophosphate synthase 8 mRNA, complete cds
AT5G59845	1.238321852	0.003435262	Arabidopsis thaliana gibberellin-regulated protein mRNA, complete cds
AT2G39518	1.119677355	0.003535982	Arabidopsis thaliana uncharacterized protein mRNA, complete cds
AT3G45960	1.119677355	0.003535982	Arabidopsis thaliana expansin-like A3 mRNA, complete cds
AT5G22240	2.297215541	0.003542768	Arabidopsis thaliana chromosome 5 sequence
AT3G44840	2.062750287	0.003692486	Arabidopsis thaliana S-adenosyl-L-methionine-dependent methyltransferases superfamily protein mRNA, complete cds
AT1G69320	2.062750287	0.003692486	Arabidopsis thaliana chromosome 1 sequence
AT3G21520	2.062750287	0.003692486	Arabidopsis thaliana chromosome 3, complete sequence
AT2G42430	1.505802162	0.003893966	Arabidopsis thaliana LOB domain-containing protein 16 mRNA, complete cds
AT1G27140	1.255395365	0.00405188	Arabidopsis thaliana glutathione S-transferase tau 14 mRNA, complete cds
AT3G59710	1.255395365	0.00405188	Arabidopsis thaliana Rossmann-fold NAD(P)-binding domain-containing protein mRNA, complete cds
AT4G09300	3.297215541	0.004054869	Arabidopsis thaliana LisH and RanBPM domain-containing protein mRNA, complete cds
AT5G35870	3.297215541	0.004054869	Arabidopsis thaliana chromosome 5 sequence
AT1G64195	3.297215541	0.004054869	Arabidopsis thaliana defensin-like protein 35 mRNA, complete cds
AT1G51840	3.297215541	0.004054869	Arabidopsis thaliana protein kinase-related protein mRNA, complete cds
AT2G35570	3.297215541	0.004054869	Arabidopsis thaliana chromosome 2, complete sequence
ATMG01220	3.297215541	0.004054869	Arabidopsis thaliana ecotype Landsberg erecta mitochondrion, complete genome
AT2G27220	3.297215541	0.004054869	Arabidopsis thaliana BEL1-like homeodomain 5 mRNA, complete cds
AT5G40210	1.079624106	0.004162156	Arabidopsis thaliana nodulin MtN21 /EamA-like transporter family protein mRNA, complete cds
AT3G11020	1.079624106	0.004162156	Arabidopsis thaliana dehydration-responsive element-binding protein 2B mRNA, complete cds
AT4G17915	1.184740811	0.00417896	Arabidopsis thaliana chromosome 4 sequence

		6	
AT5G50570	1.560249946	0.004417823	Arabidopsis thaliana squamosa promoter-binding-like protein 13 mRNA, complete cds
AT5G22520	1.560249946	0.004417823	Arabidopsis thaliana chromosome 5 sequence
AT1G18830	1.560249946	0.004417823	Arabidopsis thaliana transport protein SEC31-like protein SEC31B mRNA, complete cds
AT1G11460	1.560249946	0.004417823	Arabidopsis thaliana nodulin MtN21-like transporter family protein mRNA, complete cds
AT5G42785	1.373836822	0.004430261	Arabidopsis thaliana uncharacterized protein mRNA, complete cds
AT2G04090	1.006314341	0.004681161	Arabidopsis thaliana MATE efflux family protein mRNA, complete cds
AT2G14095	1.274847728	0.004773159	Arabidopsis thaliana uncharacterized protein mRNA, complete cds
AT1G32950	1.274847728	0.004773159	Arabidopsis thaliana subtilase family protein mRNA, complete cds
AT1G22260	1.197679867	0.004954845	Arabidopsis thaliana synaptonemal complex protein ZYP1a mRNA, complete cds
AT4G27652	1.197679867	0.004954845	Arabidopsis thaliana chromosome 4 sequence
AT1G02310	1.627364142	0.00495642	Arabidopsis thaliana mannan endo-1,4-beta-mannosidase 1 mRNA, complete cds
AT2G45930	1.084911937	0.004964978	Arabidopsis thaliana uncharacterized protein mRNA, complete cds
AT1G70860	1.135752118	0.005008749	Arabidopsis thaliana SRPBCC ligand-binding domain-containing protein mRNA, complete cds
AT4G20970	1.408246853	0.005142966	Arabidopsis thaliana basic helix-loop-helix domain-containing protein mRNA, complete cds
AT1G05450	1.71225304	0.00547489	Arabidopsis thaliana protease inhibitor/seed storage/LTP family protein mRNA, complete cds
AT3G27620	1.71225304	0.00547489	Arabidopsis thaliana alternative oxidase 1C mRNA, complete cds
AT4G03600	1.71225304	0.00547489	Arabidopsis thaliana chromosome 4 sequence
AT1G67148	1.007708923	0.005596521	Arabidopsis thaliana uncharacterized protein mRNA, complete cds
AT1G21230	1.297215541	0.005613679	Arabidopsis thaliana wall-associated receptor kinase 5 mRNA, complete cds
AT4G11310	3.975287446	0.005860071	Arabidopsis thaliana putative cysteine proteinase mRNA, complete cds
AT2G40350	3.975287446	0.005860071	Arabidopsis thaliana chromosome 2, complete sequence
AT1G66570	3.975287446	0.005860071	Arabidopsis thaliana putative sucrose transport protein SUC7 mRNA, complete cds

AT4G27670	3.975287446	0.005860071	Arabidopsis thaliana heat shock protein 21 mRNA, complete cds
AT5G20045	3.975287446	0.005860071	Arabidopsis thaliana chromosome 5 sequence
AT1G66850	3.975287446	0.005860071	Arabidopsis thaliana chromosome 1 sequence
AT3G49540	3.975287446	0.005860071	Arabidopsis thaliana uncharacterized protein mRNA, complete cds
AT2G21610	3.975287446	0.005860071	Arabidopsis thaliana pectinesterase 11 mRNA, complete cds
AT3G53600	3.975287446	0.005860071	Arabidopsis thaliana chromosome 3, complete sequence
AT4G34850	1.823284352	0.005913496	Arabidopsis thaliana protein LESS ADHESIVE POLLEN 5 mRNA, complete cds
AT5G51790	1.145212447	0.005962087	Arabidopsis thaliana transcription factor bHLH120 mRNA, complete cds
AT1G76640	2.197679867	0.006103369	Arabidopsis thaliana chromosome 1 sequence
AT1G25422	1.975287446	0.006174253	Arabidopsis thaliana chromosome 1 sequence
AT1G36622	1.975287446	0.006174253	Arabidopsis thaliana uncharacterized protein mRNA, complete cds
AT5G41280	1.975287446	0.006174253	Arabidopsis thaliana cysteine-rich repeat secretory protein 57 mRNA, complete cds
AT5G11290	1.323210749	0.00658819	Arabidopsis thaliana chromosome 5 sequence
AT5G43890	1.498849402	0.006815875	Arabidopsis thaliana chromosome 5 sequence
AT5G22890	1.229044038	0.006953578	Arabidopsis thaliana chromosome 5 sequence
AT1G32928	1.09727797	0.007073095	Arabidopsis thaliana chromosome 1 sequence
AT4G19920	1.09727797	0.007073095	Arabidopsis thaliana Toll-Interleukin-Resistance domain-containing protein mRNA, complete cds
AT5G42930	1.09727797	0.007073095	Arabidopsis thaliana lipase class 3-like protein mRNA, complete cds
AT2G07693	3.145212447	0.00758265	Arabidopsis thaliana ecotype Col-0 mitochondrion, complete genome
AT2G14230	3.145212447	0.00758265	Arabidopsis thaliana chromosome 2, complete sequence
AT3G11480	3.145212447	0.00758265	Arabidopsis thaliana SABATH methyltransferase BSMT1 mRNA, complete cds
AT1G18835	3.145212447	0.00758265	Arabidopsis thaliana chromosome 1 sequence
AT1G51400	1.353799069	0.007710217	Arabidopsis thaliana chromosome 1 sequence
AT3G53150	1.560249946	0.00774884	Arabidopsis thaliana chromosome 3, complete sequence

		2	
AT1G66960	1.24830594	0.00822675 5	Arabidopsis thaliana Terpenoid cyclases family protein mRNA, complete cds
AT1G74000	1.053289958	0.00828660 9	Arabidopsis thaliana strictosidine synthase 3 mRNA, complete cds
AT1G13330	1.104570463	0.00844707 7	Arabidopsis thaliana homologous-pairing protein 2-like protein mRNA, complete cds
AT4G11655	1.104570463	0.00844707 7	Arabidopsis thaliana uncharacterized protein mRNA, complete cds
AT1G77640	1.167932524	0.00844756 2	Arabidopsis thaliana chromosome 1 sequence
AT1G10790	1.638252458	0.00869626 9	Arabidopsis thaliana uncharacterized protein mRNA, complete cds
AT3G58780	1.638252458	0.00869626 9	Arabidopsis thaliana agamous-like MADS-box protein AGL1 mRNA, complete cds
AT4G19760	1.390324945	0.00898951 9	Arabidopsis thaliana Glycosyl hydrolase family protein with chitinase insertion domain mRNA, complete cds
AT4G25850	1.390324945	0.00898951 9	Arabidopsis thaliana OSBP(oxysterol binding protein)-related protein 4B mRNA, complete cds
AT3G05550	1.740822192	0.00957633 7	Arabidopsis thaliana Hypoxia-responsive family protein mRNA, complete cds
AT1G13590	1.012762151	0.00960073 9	Arabidopsis thaliana phytosulfokine 1 precursor mRNA, complete cds
AT1G73290	1.270743329	0.00972024 6	Arabidopsis thaliana serine carboxypeptidase-like 5 mRNA, complete cds
AT1G77200	1.270743329	0.00972024 6	Arabidopsis thaliana chromosome 1 sequence
AT2G45760	1.270743329	0.00972024 6	Arabidopsis thaliana chromosome 2, complete sequence
AT4G37140	2.434719064	0.00975262 2	Arabidopsis thaliana putative inactive methylesterase 20 mRNA, complete cds
AT5G37490	2.434719064	0.00975262 2	Arabidopsis thaliana chromosome 5 sequence
AT3G13220	2.434719064	0.00975262 2	Arabidopsis thaliana ABC transporter G family member 26 mRNA, complete cds
AT1G62262	2.434719064	0.00975262 2	Arabidopsis thaliana SLAC1 homologue 4 mRNA, complete cds
AT1G67265	2.434719064	0.00975262 2	Arabidopsis thaliana chromosome 1 sequence
AT1G15900	2.434719064	0.00975262 2	Arabidopsis thaliana chromosome 1 sequence
AT5G11410	1.057749606	0.00991921 3	Arabidopsis thaliana protein kinase family protein mRNA, complete cds
AT2G28500	1.057749606	0.00991921	Arabidopsis thaliana LOB domain-containing protein 11

		3	mRNA, complete cds
AT3G21020	1.181738323	0.010053432	Arabidopsis thaliana chromosome 3, complete sequence
AT2G21350	1.112790969	0.010091812	Arabidopsis thaliana RNA-binding CRS1 / YhbY (CRM) domain protein mRNA, complete cds
AT2G36270	1.882178041	0.010242325	Arabidopsis thaliana protein abscisic acid-insensitive 5 mRNA, complete cds
AT3G55515	1.882178041	0.010242325	Arabidopsis thaliana chromosome 3, complete sequence
AT5G66830	1.882178041	0.010242325	Arabidopsis thaliana chromosome 5 sequence
AT2G42955	1.882178041	0.010242325	Arabidopsis thaliana uncharacterized protein mRNA, complete cds
AT2G36750	1.882178041	0.010242325	Arabidopsis thaliana chromosome 2, complete sequence
AT1G50400	1.434719064	0.010427558	Arabidopsis thaliana eukaryotic porin family protein mRNA, complete cds
AT5G49340	1.434719064	0.010427558	Arabidopsis thaliana protein TRICHOME BIREFRINGENCE-LIKE mRNA, complete cds
AT1G69430	1.434719064	0.010427558	Arabidopsis thaliana chromosome 1 sequence
ATMG00560	2.090764663	0.010440636	Arabidopsis thaliana ecotype Landsberg erecta mitochondrion, complete genome
ATMG00730	2.090764663	0.010440636	Arabidopsis thaliana mitochondrial genome
AT2G38340	2.090764663	0.010440636	Arabidopsis thaliana chromosome 2, complete sequence
AT2G46990	3.782642368	0.011216133	Arabidopsis thaliana auxin-responsive protein IAA20 mRNA, complete cds
AT4G05370	3.782642368	0.011216133	Arabidopsis thaliana chromosome 4 sequence
AT1G27040	3.782642368	0.011216133	Arabidopsis thaliana probable peptide/nitrate transporter mRNA, complete cds
AT1G74130	3.782642368	0.011216133	Arabidopsis thaliana Rhomboid-related intramembrane serine protease family protein mRNA, complete cds
AT5G53230	3.782642368	0.011216133	Arabidopsis thaliana chromosome 5 sequence
AT1G47405	3.782642368	0.011216133	Arabidopsis thaliana chromosome 1 sequence
AT2G02340	3.782642368	0.011216133	Arabidopsis thaliana phloem protein 2-B8 mRNA, complete cds
AT2G18190	3.782642368	0.011216133	Arabidopsis thaliana P-loop containing nucleoside triphosphate hydrolases superfamily protein mRNA, complete cds

AT5G53190	3.782642368	0.011216133	Arabidopsis thaliana bidirectional sugar transporter SWEET3 mRNA, complete cds
AT4G29250	3.782642368	0.011216133	Arabidopsis thaliana HXXXD-type acyl-transferase-like protein mRNA, complete cds
AT1G57650	3.782642368	0.011216133	Arabidopsis thaliana ATP binding protein mRNA, complete cds
AT1G35515	1.297215541	0.011464069	Arabidopsis thaliana R2R3-type MYB transcription factor mRNA, complete cds
AT5G64190	1.489860619	0.01200951	Arabidopsis thaliana uncharacterized protein mRNA, complete cds
AT4G22960	1.489860619	0.01200951	Arabidopsis thaliana uncharacterized protein mRNA, complete cds
AT1G14960	1.122128834	0.012061231	Arabidopsis thaliana polyyketide cyclase/dehydrase and lipid transport superfamily protein mRNA, complete cds
AT4G21340	1.3289244	0.013486715	Arabidopsis thaliana transcription factor bHLH103 mRNA, complete cds
AT1G05560	1.560249946	0.013690091	Arabidopsis thaliana chromosome 1 sequence
AT5G38096	1.560249946	0.013690091	Arabidopsis thaliana chromosome 5 sequence
AT2G02240	1.560249946	0.013690091	Arabidopsis thaliana F-box domain protein MEE66 mRNA, complete cds
AT4G21920	1.560249946	0.013690091	Arabidopsis thaliana chromosome 4 sequence
AT2G46770	2.975287446	0.014184315	Arabidopsis thaliana NAC transcription factor NST1 mRNA, complete cds
AT1G35255	2.975287446	0.014184315	Arabidopsis thaliana chromosome 1 sequence
AT4G15100	2.975287446	0.014184315	Arabidopsis thaliana serine carboxypeptidase-like 30 mRNA, complete cds
AT4G26790	2.975287446	0.014184315	Arabidopsis thaliana GDSL esterase/lipase mRNA, complete cds
AT2G42560	2.975287446	0.014184315	Arabidopsis thaliana late embryogenesis abundant domain-containing protein mRNA, complete cds
ATMG00180	2.975287446	0.014184315	Arabidopsis thaliana ecotype Columbia cytochrome c biogenesis orf452 mRNA, partial cds; mitochondrial
AT1G13608	2.975287446	0.014184315	Arabidopsis thaliana putative defensin-like protein 288 mRNA, complete cds
AT1G64560	2.975287446	0.014184315	Arabidopsis thaliana chromosome 1 sequence
AT1G30660	2.975287446	0.014184315	Arabidopsis thaliana nucleic acid binding protein mRNA, complete cds
AT2G37000	2.975287446	0.014184315	Arabidopsis thaliana chromosome 2, complete sequence

AT1G75590	1.132828723	0.014419876	Arabidopsis thaliana chromosome 1 sequence
AT1G09180	1.132828723	0.014419876	Arabidopsis thaliana secretion-associated RAS 1 protein mRNA, complete cds
AT5G41650	1.132828723	0.014419876	Arabidopsis thaliana lactoylglutathione lyase family protein / glyoxalase I family protein mRNA, complete cds
AT3G04330	1.653359351	0.015368143	Arabidopsis thaliana chromosome 3, complete sequence
AT5G27440	1.653359351	0.015368143	Arabidopsis thaliana chromosome 5 sequence
AT1G01453	1.653359351	0.015368143	Arabidopsis thaliana chromosome 1 sequence
AT3G04181	1.653359351	0.015368143	Arabidopsis thaliana uncharacterized protein mRNA, complete cds
AT5G11140	1.653359351	0.015368143	Arabidopsis thaliana chromosome 5 sequence
AT1G37150	1.653359351	0.015368143	Arabidopsis thaliana holocarboxylase synthetase 2 mRNA, complete cds
AT5G63450	1.367604869	0.015809942	Arabidopsis thaliana chromosome 5 sequence
AT3G52160	1.367604869	0.015809942	Arabidopsis thaliana 3-ketoacyl-CoA synthase 15 mRNA, complete cds
AT4G10270	1.367604869	0.015809942	Arabidopsis thaliana chromosome 4 sequence
AT3G59450	1.782642368	0.016840329	Arabidopsis thaliana putative calcium-binding protein CML46 mRNA, complete cds
AT2G07787	1.782642368	0.016840329	Arabidopsis thaliana uncharacterized protein mRNA, complete cds
AT4G04760	1.782642368	0.016840329	Arabidopsis thaliana sugar transporter ERD6-like 15 mRNA, complete cds
AT1G33640	1.782642368	0.016840329	Arabidopsis thaliana uncharacterized protein mRNA, complete cds
AT1G09350	1.145212447	0.017244749	Arabidopsis thaliana galactinol synthase 3 mRNA, complete cds
AT2G07808	1.145212447	0.017244749	Arabidopsis thaliana chromosome 2, complete sequence
AT5G41710	2.297215541	0.017260999	Arabidopsis thaliana chromosome 5 sequence
AT4G12500	2.297215541	0.017260999	Arabidopsis thaliana chromosome 4 sequence
AT3G16210	2.297215541	0.017260999	Arabidopsis thaliana chromosome 3, complete sequence
AT5G42445	2.297215541	0.017260999	Arabidopsis thaliana chromosome 5 sequence

AT3G13210	1.975287446	0.017715702	Arabidopsis thaliana putative crooked neck protein / cell cycle protein mRNA, complete cds
AT4G16230	1.975287446	0.017715702	Arabidopsis thaliana GDSL-like lipase mRNA, complete cds
AT1G68320	1.975287446	0.017715702	Arabidopsis thaliana R2R3-MYB transcription family mRNA, complete cds
AT2G28680	1.975287446	0.017715702	Arabidopsis thaliana RmlC-like cupins superfamily protein mRNA, complete cds
AT2G13680	1.415860037	0.018438801	Arabidopsis thaliana callose synthase 5 mRNA, complete cds
AT1G73580	1.415860037	0.018438801	Arabidopsis thaliana C2 domain-containing protein mRNA, complete cds
AT5G57540	1.415860037	0.018438801	Arabidopsis thaliana xyloglucan endotransglucosylase/hydrolase 13 mRNA, complete cds
AT2G04495	1.264794063	0.020048555	Arabidopsis thaliana chromosome 2, complete sequence
AT5G39150	1.08220265	0.020510528	Arabidopsis thaliana germin-like protein subfamily 1 member 17 mRNA, complete cds
AT2G13960	1.08220265	0.020510528	Arabidopsis thaliana homeodomain-like superfamily protein mRNA, complete cds
AT3G63050	1.08220265	0.020510528	Arabidopsis thaliana chromosome 3, complete sequence
AT2G44700	1.477787786	0.021342534	Arabidopsis thaliana chromosome 2, complete sequence
AT2G45940	1.477787786	0.021342534	Arabidopsis thaliana uncharacterized protein mRNA, complete cds
AT5G24820	3.560249946	0.021643442	Arabidopsis thaliana aspartyl protease family protein mRNA, complete cds
AT5G64790	3.560249946	0.021643442	Arabidopsis thaliana O-Glycosyl hydrolases family 17 protein mRNA, complete cds
AT4G29340	3.560249946	0.021643442	Arabidopsis thaliana profilin 4 mRNA, complete cds
AT1G61070	3.560249946	0.021643442	Arabidopsis thaliana defensin-like protein mRNA, complete cds
AT4G04409	3.560249946	0.021643442	Arabidopsis thaliana chromosome 4 sequence
AT3G11773	3.560249946	0.021643442	Arabidopsis thaliana electron carrier/ protein disulfide oxidoreductase mRNA, complete cds
AT2G07749	3.560249946	0.021643442	Arabidopsis thaliana chromosome 2, complete sequence
AT1G18120	3.560249946	0.021643442	Arabidopsis thaliana chromosome 1 sequence
AT1G80470	3.560249946	0.021643442	Arabidopsis thaliana F-box/FBD/LRR-repeat protein mRNA, complete cds

AT5G09910	3.560249946	0.021643442	Arabidopsis thaliana Ras-related small GTP-binding family protein mRNA, complete cds
AT5G07600	3.560249946	0.021643442	Arabidopsis thaliana Oleosin family protein mRNA, complete cds
AT2G07683	3.560249946	0.021643442	Arabidopsis thaliana chromosome 2, complete sequence
AT3G28060	3.560249946	0.021643442	Arabidopsis thaliana nodulin MtN21-like transporter family protein mRNA, complete cds
AT5G05965	3.560249946	0.021643442	Arabidopsis thaliana uncharacterized protein mRNA, complete cds
AT2G22750	3.560249946	0.021643442	Arabidopsis thaliana transcription factor bHLH18 mRNA, complete cds
ATMG01410	3.560249946	0.021643442	Arabidopsis thaliana mitochondrial genome
AT2G32130	3.560249946	0.021643442	Arabidopsis thaliana chromosome 2, complete sequence
AT2G31210	3.560249946	0.021643442	Arabidopsis thaliana transcription factor bHLH91 mRNA, complete cds
AT4G28397	3.560249946	0.021643442	Arabidopsis thaliana uncharacterized protein mRNA, complete cds
AT3G25490	3.560249946	0.021643442	Arabidopsis thaliana putative wall-associated receptor kinase-like 16 mRNA, complete cds
AT2G07687	3.560249946	0.021643442	Arabidopsis thaliana ecotype Landsberg erecta mitochondrion, complete genome
AT1G77730	3.560249946	0.021643442	Arabidopsis thaliana Pleckstrin homology (PH) domain-containing protein mRNA, complete cds
AT1G36610	3.560249946	0.021643442	Arabidopsis thaliana chromosome 1 sequence
AT4G35590	1.297215541	0.023739857	Arabidopsis thaliana RWP-RK domain-containing protein mRNA, complete cds
AT3G53232	1.297215541	0.023739857	Arabidopsis thaliana chromosome 3, complete sequence
AT3G48290	1.025913519	0.023998519	Arabidopsis thaliana cytochrome P450 71A24 mRNA, complete cds
AT1G21320	1.560249946	0.024417542	Arabidopsis thaliana nucleic acid/nucleotide binding protein mRNA, complete cds
AT5G18710	1.560249946	0.024417542	Arabidopsis thaliana uncharacterized protein mRNA, complete cds
AT1G15010	1.090764663	0.024644879	Arabidopsis thaliana chromosome 1 sequence
AT4G12580	1.176921307	0.024674774	Arabidopsis thaliana chromosome 4 sequence
AT5G65080	1.176921307	0.024674774	Arabidopsis thaliana protein MADS AFFECTING FLOWERING 5 mRNA, complete cds

AT5G24090	1.176921307	0.024674774	Arabidopsis thaliana chitinase A mRNA, complete cds
AT1G50770	2.782642368	0.026523913	Arabidopsis thaliana Aminotransferase-like, plant mobile domain family protein mRNA, complete cds
AT2G07785	2.782642368	0.026523913	Arabidopsis thaliana NADH dehydrogenase I subunit 1 mRNA, complete cds
AT1G22000	2.782642368	0.026523913	Arabidopsis thaliana putative F-box/FBD/LRR-repeat protein mRNA, complete cds
AT2G32740	2.782642368	0.026523913	Arabidopsis thaliana chromosome 2, complete sequence
ATMG01200	2.782642368	0.026523913	Arabidopsis thaliana ecotype Landsberg erecta mitochondrion, complete genome
AT3G46680	2.782642368	0.026523913	Arabidopsis thaliana UDP-glycosyltransferase 76E6 mRNA, complete cds
AT5G22150	2.782642368	0.026523913	Arabidopsis thaliana uncharacterized protein mRNA, complete cds
AT2G04490	2.782642368	0.026523913	Arabidopsis thaliana chromosome 2, complete sequence
AT5G48175	2.782642368	0.026523913	Arabidopsis thaliana uncharacterized protein mRNA, complete cds
AT2G28755	2.782642368	0.026523913	Arabidopsis thaliana UDP-D-glucuronate carboxy-lyase-related protein mRNA, complete cds
AT5G54095	2.782642368	0.026523913	Arabidopsis thaliana uncharacterized protein mRNA, complete cds
AT3G30120	2.782642368	0.026523913	Arabidopsis thaliana chromosome 3, complete sequence
AT3G63060	1.675727164	0.027413468	Arabidopsis thaliana chromosome 3, complete sequence
AT4G28110	1.675727164	0.027413468	Arabidopsis thaliana myb domain protein 41 mRNA, complete cds
AT3G19550	1.675727164	0.027413468	Arabidopsis thaliana uncharacterized protein mRNA, complete cds
AT1G44130	1.337857525	0.028023683	Arabidopsis thaliana aspartyl protease family protein mRNA, complete cds
AT5G06400	1.02973523	0.02891851	Arabidopsis thaliana chromosome 5 sequence
AT1G79120	1.02973523	0.02891851	Arabidopsis thaliana ubiquitin carboxyl-terminal hydrolase family protein mRNA, complete cds
AT5G42840	1.02973523	0.02891851	Arabidopsis thaliana cysteine/histidine-rich C1 domain-containing protein mRNA, complete cds
AT1G59865	1.197679867	0.029511844	Arabidopsis thaliana uncharacterized protein mRNA, complete cds
AT1G16225	1.197679867	0.029511844	Arabidopsis thaliana putative syntaxin-type t-SNARE protein mRNA, complete cds
AT1G24210	1.100818328	0.02963910	Arabidopsis thaliana paired amphipathic helix

		8	domain-containing protein mRNA, complete cds
AT3G12720	1.100818328	0.02963910 8	Arabidopsis thaliana myb domain protein 67 mRNA, complete cds
AT2G31945	1.100818328	0.02963910 8	Arabidopsis thaliana chromosome 2, complete sequence
AT5G38250	1.849756564	0.02977962 1	Arabidopsis thaliana Protein kinase family protein mRNA, complete cds
ATMG0006 0	1.849756564	0.02977962 1	Arabidopsis thaliana NADH dehydrogenase subunit 5 mRNA, complete cds; mitochondrial
AT5G44574	1.849756564	0.02977962 1	Arabidopsis thaliana uncharacterized protein mRNA, complete cds
AT2G46130	1.849756564	0.02977962 1	Arabidopsis thaliana WRKY DNA-binding protein 43 mRNA, complete cds
AT1G29000	1.849756564	0.02977962 1	Arabidopsis thaliana heavy-metal-associated domain-containing protein mRNA, complete cds
AT1G15360	2.145212447	0.03033082 9	Arabidopsis thaliana ethylene-responsive transcription factor WIN1 mRNA, complete cds
AT1G26720	2.145212447	0.03033082 9	Arabidopsis thaliana chromosome 1 sequence
AT5G42230	2.145212447	0.03033082 9	Arabidopsis thaliana serine carboxypeptidase-like 41 mRNA, complete cds
AT1G21360	2.145212447	0.03033082 9	Arabidopsis thaliana glycolipid transfer protein 2 mRNA, complete cds
AT5G44570	2.145212447	0.03033082 9	Arabidopsis thaliana uncharacterized protein mRNA, complete cds
AT1G70130	2.145212447	0.03033082 9	Arabidopsis thaliana putative L-type lectin-domain containing receptor kinase V.2 mRNA, complete cds
AT2G25470	1.390324945	0.03291995 5	Arabidopsis thaliana receptor like protein 21 mRNA, complete cds
AT3G59730	1.390324945	0.03291995 5	Arabidopsis thaliana chromosome 3, complete sequence
AT5G09970	1.390324945	0.03291995 5	Arabidopsis thaliana cytochrome P450, family 78, subfamily A, polypeptide 7 mRNA, complete cds
ATCG00580	1.034181135	0.03489471 1	Arabidopsis thaliana chloroplast DNA, complete genome, ecotype: Columbia
AT2G32620	1.034181135	0.03489471 1	Arabidopsis thaliana cellulose synthase-like protein B2 mRNA, complete cds
AT5G60770	1.223214959	0.03527857 1	Arabidopsis thaliana nitrate transporter 2.4 mRNA, complete cds
AT2G28105	1.223214959	0.03527857 1	Arabidopsis thaliana uncharacterized protein mRNA, complete cds
AT5G20710	1.223214959	0.03527857 1	Arabidopsis thaliana beta-galactosidase 7 mRNA, complete cds
AT2G01580	1.223214959	0.03527857	Arabidopsis thaliana chromosome 2, complete sequence

		1	
AT5G23950	1.223214959	0.035278571	Arabidopsis thaliana chromosome 5 sequence
AT1G29100	1.460714273	0.038368495	Arabidopsis thaliana heavy-metal-associated domain-containing protein mRNA, complete cds
AT5G02570	1.460714273	0.038368495	Arabidopsis thaliana chromosome 5 sequence
AT2G28420	1.460714273	0.038368495	Arabidopsis thaliana chromosome 2, complete sequence
AT5G13170	1.460714273	0.038368495	Arabidopsis thaliana senescence-associated protein 29 mRNA, complete cds
AT3G09680	1.460714273	0.038368495	Arabidopsis thaliana 40S ribosomal protein S23-1 mRNA, complete cds
AT4G34550	1.460714273	0.038368495	Arabidopsis thaliana chromosome 4 sequence
AT1G02070	1.460714273	0.038368495	Arabidopsis thaliana uncharacterized protein mRNA, complete cds
AT3G48180	1.255395365	0.042124225	Arabidopsis thaliana chromosome 3, complete sequence
AT3G54530	3.297215541	0.042140827	Arabidopsis thaliana uncharacterized protein mRNA, complete cds
AT3G18330	3.297215541	0.042140827	Arabidopsis thaliana chromosome 3, complete sequence
AT3G43715	3.297215541	0.042140827	Arabidopsis thaliana chromosome 3, complete sequence
AT5G27780	3.297215541	0.042140827	Arabidopsis thaliana chromosome 5 sequence
AT4G25670	3.297215541	0.042140827	Arabidopsis thaliana conserved peptide upstream open reading frame 12 mRNA, complete cds
AT5G61100	3.297215541	0.042140827	Arabidopsis thaliana uncharacterized protein mRNA, complete cds
AT1G36600	3.297215541	0.042140827	Arabidopsis thaliana chromosome 1 sequence
AT5G11360	3.297215541	0.042140827	Arabidopsis thaliana Interleukin-1 receptor-associated kinase 4 protein mRNA, complete cds
AT2G35070	3.297215541	0.042140827	Arabidopsis thaliana uncharacterized protein mRNA, complete cds
AT2G21400	3.297215541	0.042140827	Arabidopsis thaliana SHI-related sequence3 mRNA, complete cds
ATMG00010	3.297215541	0.042140827	Arabidopsis thaliana ecotype Landsberg erecta mitochondrion, complete genome
AT1G66990	3.297215541	0.042140827	Arabidopsis thaliana chromosome 1 sequence
AT2G32179	3.297215541	0.04214082	Arabidopsis thaliana uncharacterized protein mRNA,

		7	complete cds
AT5G16023	3.297215541	0.04214082 7	Arabidopsis thaliana chromosome 5 sequence
AT4G37420	3.297215541	0.04214082 7	Arabidopsis thaliana chromosome 4 sequence
AT1G75930	3.297215541	0.04214082 7	Arabidopsis thaliana GDSL esterase/lipase EXL6 mRNA, complete cds
AT4G29570	3.297215541	0.04214082 7	Arabidopsis thaliana chromosome 4 sequence
AT5G21960	3.297215541	0.04214082 7	Arabidopsis thaliana chromosome 5 sequence
AT2G47870	3.297215541	0.04214082 7	Arabidopsis thaliana chromosome 2, complete sequence
AT3G42960	3.297215541	0.04214082 7	Arabidopsis thaliana protein TAPETUM 1 mRNA, complete cds
AT2G07806	3.297215541	0.04214082 7	Arabidopsis thaliana uncharacterized protein mRNA, complete cds
AT5G45890	3.297215541	0.04214082 7	Arabidopsis thaliana cysteine protease SAG12 mRNA, complete cds
AT5G14490	3.297215541	0.04214082 7	Arabidopsis thaliana NAC domain containing protein 85 mRNA, complete cds
AT1G28695	3.297215541	0.04214082 7	Arabidopsis thaliana nucleotide-diphospho-sugar transferase-like protein mRNA, complete cds
AT1G61160	3.297215541	0.04214082 7	Arabidopsis thaliana uncharacterized protein mRNA, complete cds
AT1G34490	3.297215541	0.04214082 7	Arabidopsis thaliana chromosome 1 sequence
AT1G68765	3.297215541	0.04214082 7	Arabidopsis thaliana chromosome 1 sequence
AT1G78330	3.297215541	0.04214082 7	Arabidopsis thaliana chromosome 1 sequence
AT1G77160	3.297215541	0.04214082 7	Arabidopsis thaliana uncharacterized protein mRNA, complete cds
AT1G71920	3.297215541	0.04214082 7	Arabidopsis thaliana histidinol-phosphate aminotransferase 2 mRNA, complete cds
AT1G14455	3.297215541	0.04214082 7	Arabidopsis thaliana chromosome 1 sequence
ATMG0072 0	3.297215541	0.04214082 7	Arabidopsis thaliana mitochondrial genome
AT1G12540	3.297215541	0.04214082 7	Arabidopsis thaliana transcription factor bHLH55 mRNA, complete cds
AT2G11810	1.039417783	0.04217012 5	Arabidopsis thaliana Monogalactosyldiacylglycerol synthase 3 mRNA, complete cds
ATMG0065	1.127290539	0.04298832	Arabidopsis thaliana ecotype Landsberg erecta

0		4	mitochondrion, complete genome
AT2G34610	1.127290539	0.042988324	Arabidopsis thaliana chromosome 2, complete sequence
AT1G27740	1.560249946	0.044124752	Arabidopsis thaliana transcription factor RSL4 mRNA, complete cds
AT5G52270	1.560249946	0.044124752	Arabidopsis thaliana SNARE-like superfamily protein mRNA, complete cds
AT4G20920	1.560249946	0.044124752	Arabidopsis thaliana double-stranded RNA-binding domain (DsRBD)-containing protein mRNA, complete cds
AT1G54400	1.560249946	0.044124752	Arabidopsis thaliana alpha-crystallin domain of heat shock protein-containing protein mRNA, complete cds
AT1G74080	1.560249946	0.044124752	Arabidopsis thaliana myb domain protein 122 mRNA, complete cds
AT5G06760	1.71225304	0.049516832	Arabidopsis thaliana late embryogenesis abundant protein 4-5 mRNA, complete cds
AT5G41570	1.71225304	0.049516832	Arabidopsis thaliana WRKY transcription factor 24 mRNA, complete cds
AT3G29780	1.71225304	0.049516832	Arabidopsis thaliana chromosome 3, complete sequence
AT3G49340	1.71225304	0.049516832	Arabidopsis thaliana putative cysteine proteinase mRNA, complete cds
AT5G60130	1.71225304	0.049516832	Arabidopsis thaliana AP2/B3-like transcriptional factor family protein mRNA, complete cds
AT5G59370	1.71225304	0.049516832	Arabidopsis thaliana actin 4 mRNA, complete cds
AT2G34580	1.71225304	0.049516832	Arabidopsis thaliana uncharacterized protein mRNA, complete cds
AT3G18360	1.71225304	0.049516832	Arabidopsis thaliana chromosome 3, complete sequence
AT1G79250	2.560249946	0.049531582	Arabidopsis thaliana AGC kinase 1.7 mRNA, complete cds
AT4G09100	2.560249946	0.049531582	Arabidopsis thaliana chromosome 4 sequence
AT3G08810	2.560249946	0.049531582	Arabidopsis thaliana chromosome 3, complete sequence
AT1G32880	2.560249946	0.049531582	Arabidopsis thaliana armadillo/beta-catenin-like repeats-containing protein mRNA, complete cds
AT1G50960	2.560249946	0.049531582	Arabidopsis thaliana gibberellin 2-beta-dioxygenase 7 mRNA, complete cds
AT4G31940	2.560249946	0.049531582	Arabidopsis thaliana cytochrome P450, family 82, subfamily C, polypeptide 4 mRNA, complete cds
AT5G04275	2.560249946	0.049531582	Arabidopsis thaliana chromosome 5 sequence
AT3G53250	2.560249946	0.04953158	Arabidopsis thaliana chromosome 3, complete sequence

		2	
AT4G07830	2.560249946	0.049531582	Arabidopsis thaliana chromosome 4 sequence
AT4G31950	2.560249946	0.049531582	Arabidopsis thaliana cytochrome P450, family 82, subfamily C, polypeptide 3 mRNA, complete cds
AT3G29190	2.560249946	0.049531582	Arabidopsis thaliana terpenoid synthase 15 mRNA, complete cds
AT2G07799	2.560249946	0.049531582	Arabidopsis thaliana ecotype Col-0 mitochondrion, complete genome
AT4G10720	1.297215541	0.050188414	Arabidopsis thaliana ankyrin repeat-containing protein mRNA, complete cds
AT2G23270	1.297215541	0.050188414	Arabidopsis thaliana chromosome 2, complete sequence
AT5G53390	1.297215541	0.050188414	Arabidopsis thaliana bifunctional wax ester synthase/diacylglycerol acyltransferase mRNA, complete cds
AT1G69180	1.045676774	0.051049746	Arabidopsis thaliana putative transcription factor CRABS CLAW mRNA, complete cds
AT3G25950	1.045676774	0.051049746	Arabidopsis thaliana chromosome 3, complete sequence
AT3G14630	1.045676774	0.051049746	Arabidopsis thaliana cytochrome P450, family 72, subfamily A, polypeptide 9 mRNA, complete cds
AT3G22560	1.145212447	0.051842013	Arabidopsis thaliana chromosome 3, complete sequence
AT4G30370	1.145212447	0.051842013	Arabidopsis thaliana chromosome 4 sequence
AT5G44990	1.145212447	0.051842013	Arabidopsis thaliana Glutathione S-transferase family protein mRNA, complete cds
AT2G35075	1.145212447	0.051842013	Arabidopsis thaliana uncharacterized protein mRNA, complete cds
AT3G50310	1.975287446	0.052827617	Arabidopsis thaliana chromosome 3, complete sequence
AT5G21120	1.975287446	0.052827617	Arabidopsis thaliana chromosome 5 sequence
AT5G26700	1.975287446	0.052827617	Arabidopsis thaliana putative germin-like protein subfamily 2 member 5 mRNA, complete cds
AT3G26816	1.975287446	0.052827617	Arabidopsis thaliana chromosome 3, complete sequence
AT1G07500	1.975287446	0.052827617	Arabidopsis thaliana uncharacterized protein mRNA, complete cds
AT1G47620	1.975287446	0.052827617	Arabidopsis thaliana chromosome 1 sequence
AT1G04445	1.975287446	0.052827617	Arabidopsis thaliana chromosome 1 sequence

AT3G45638	1.975287446	0.052827617	Arabidopsis thaliana clone asmbl_10177 unknown mRNA sequence
AT5G57567	1.975287446	0.052827617	Arabidopsis thaliana uncharacterized protein mRNA, complete cds
AT4G10860	1.975287446	0.052827617	Arabidopsis thaliana chromosome 4 sequence
AT1G55790	1.353799069	0.059553123	Arabidopsis thaliana uncharacterized protein mRNA, complete cds
AT5G24080	1.353799069	0.059553123	Arabidopsis thaliana G-type lectin S-receptor-like serine/threonine protein kinase mRNA, complete cds
AT2G33350	1.353799069	0.059553123	Arabidopsis thaliana CCT motif-containing protein mRNA, complete cds
AT3G42850	1.353799069	0.059553123	Arabidopsis thaliana Mevalonate/galactokinase family protein mRNA, complete cds
AT2G07708	1.053289958	0.061918833	Arabidopsis thaliana chromosome 2, complete sequence
AT1G29179	1.053289958	0.061918833	Arabidopsis thaliana chromosome 1 sequence
AT2G29950	1.053289958	0.061918833	Arabidopsis thaliana chromosome 2, complete sequence
AT3G47770	1.167932524	0.062566131	Arabidopsis thaliana ABC transporter A family member 6 mRNA, complete cds
AT1G63245	1.167932524	0.062566131	Arabidopsis thaliana chromosome 1 sequence
AT3G61450	1.167932524	0.062566131	Arabidopsis thaliana syntaxin-73 mRNA, complete cds
AT5G09805	1.167932524	0.062566131	Arabidopsis thaliana chromosome 5 sequence
AT4G36230	1.434719064	0.070121414	Arabidopsis thaliana chromosome 4 sequence
AT5G41780	1.434719064	0.070121414	Arabidopsis thaliana myosin heavy chain-like protein mRNA, complete cds
AT1G15330	1.434719064	0.070121414	Arabidopsis thaliana Cystathionine beta-synthase (CBS) protein mRNA, complete cds

Down regulated genes in *fc1*(+Cd)/*fc1*(-Cd)

Gene	Log2FoldChange	pval	NT:Description
AT4G35090	-1.717045526	0	Arabidopsis thaliana catalase 2 mRNA, complete cds
AT1G23310	-1.149130932	0	Arabidopsis thaliana glutamate:glyoxylate aminotransferase mRNA, complete cds
AT1G64720	-1.559057802	0	Arabidopsis thaliana membrane related protein CP5 mRNA, complete cds

AT5G04140	-1.225687599	0	Arabidopsis thaliana ferredoxin-dependent glutamate synthase 1 mRNA, complete cds
AT1G37130	-1.282322937	0	Arabidopsis thaliana nitrate reductase [NADH] 2 mRNA, complete cds
AT1G01060	-1.995091976	0	Arabidopsis thaliana protein LHY mRNA, complete cds
AT5G14740	-1.132051847	0	Arabidopsis thaliana carbonic anhydrase 2 mRNA, complete cds
AT3G14420	-1.057084935	0	Arabidopsis thaliana peroxisomal (S)-2-hydroxy-acid oxidase GLO1 mRNA, complete cds
AT4G26850	-1.544578599	0	Arabidopsis thaliana GDP-L-galactose phosphorylase 1 mRNA, complete cds
AT4G28080	-1.18653999	0	Arabidopsis thaliana tetratricopeptide repeat domain protein mRNA, complete cds
AT1G68520	-2.13210142	0	Arabidopsis thaliana zinc finger protein CONSTANS-LIKE 6 mRNA, complete cds
AT5G37260	-1.897121127	0	Arabidopsis thaliana MYB family transcription factor Circadian 1 mRNA, complete cds
AT2G46830	-1.706348725	0	Arabidopsis thaliana protein CCA1 mRNA, complete cds
AT1G01320	-1.064355663	0	Arabidopsis thaliana tetratricopeptide repeat-containing protein mRNA, complete cds
AT3G24420	-2.312682219	0	Arabidopsis thaliana hydrolase, alpha/beta fold family protein mRNA, complete cds
AT4G10120	-2.050038454	0	Arabidopsis thaliana probable sucrose-phosphate synthase 4 mRNA, complete cds
AT4G25100	-1.695163964	0	Arabidopsis thaliana superoxide dismutase [Fe] mRNA, complete cds
AT5G17300	-1.974849278	0	Arabidopsis thaliana myb family transcription factor RVE1 mRNA, complete cds
AT1G28290	-1.501351614	0	Arabidopsis thaliana arabinogalactan protein 31 mRNA, complete cds
AT4G01800	-1.425928747	0	Arabidopsis thaliana protein translocase subunit secA mRNA, complete cds
AT5G18670	-1.740179438	0	Arabidopsis thaliana putative beta-amylase BMY3 mRNA, complete cds

AT2G30520	-1.062589779	1.16E-281	Arabidopsis thaliana Root phototropism protein 2 mRNA, complete cds
AT1G69530	-1.151069102	1.80E-277	Arabidopsis thaliana expansin A1 mRNA, complete cds
AT1G16720	-1.172984308	4.67E-263	Arabidopsis thaliana high chlorophyll fluorescence phenotype 173 protein mRNA, complete cds
AT5G02840	-1.50377298	3.31E-261	Arabidopsis thaliana protein LHY/CCA1-like 1 mRNA, complete cds
AT5G40850	-1.149249111	7.67E-261	Arabidopsis thaliana urophorphyrin methylase 1 mRNA, complete cds
AT1G15290	-1.200436916	5.33E-258	Arabidopsis thaliana tetratricopeptide repeat-containing protein mRNA, complete cds
AT5G17230	-1.001509729	3.44E-253	Arabidopsis thaliana phytoene synthase mRNA, complete cds
AT5G04810	-1.581987117	2.72E-222	Arabidopsis thaliana pentatricopeptide (PPR) repeat-containing protein mRNA, complete cds
AT3G28270	-1.788541699	1.11E-216	Arabidopsis thaliana chromosome 3, complete sequence
AT5G56850	-1.449951366	3.95E-208	Arabidopsis thaliana uncharacterized protein mRNA, complete cds
AT1G55960	-1.471953321	5.10E-208	Arabidopsis thaliana putative polyketide cyclase/dehydrase and lipid transport-like protein mRNA, complete cds
AT5G24150	-1.222403943	4.59E-196	Arabidopsis thaliana Squalene monooxygenase 5 mRNA, complete cds
AT5G13930	-1.36381303	4.46E-193	Arabidopsis thaliana chalcone synthase mRNA, complete cds
AT5G17170	-1.284035194	3.20E-192	Arabidopsis thaliana protein ENHANCER OF SOS3-1 mRNA, complete cds
AT3G17510	-1.25098841	5.28E-181	Arabidopsis thaliana CBL-interacting serine/threonine-protein kinase 1 mRNA, complete cds
AT3G02380	-1.541853562	5.85E-179	Arabidopsis thaliana zinc finger protein CONSTANS-LIKE 2 mRNA, complete cds
AT1G52870	-1.171525126	2.31E-174	Arabidopsis thaliana peroxisomal membrane Mpv17/PMP22 family protein mRNA, complete cds
AT4G38950	-1.60759774	1.90E-164	Arabidopsis thaliana ATP binding microtubule motor family protein mRNA, complete cds

AT3G09600	-1.789273384	2.82E-163	Arabidopsis thaliana protein REVEILLE 8 mRNA, complete cds
AT3G52720	-1.420548747	4.13E-162	Arabidopsis thaliana alpha carbonic anhydrase 1 mRNA, complete cds
AT5G13770	-1.485259269	2.17E-159	Arabidopsis thaliana chromosome 5 sequence
AT3G02830	-1.121470574	1.39E-158	Arabidopsis thaliana zinc finger protein 1 mRNA, complete cds
AT5G01920	-1.400621742	4.47E-157	Arabidopsis thaliana serine/threonine-protein kinase STN8 mRNA, complete cds
AT1G08570	-1.336500619	3.44E-153	Arabidopsis thaliana atypical CYS HIS rich thioredoxin 4 mRNA, complete cds
AT1G64500	-1.578476511	1.81E-151	Arabidopsis thaliana chromosome 1 sequence
AT2G43150	-1.157931903	5.41E-142	Arabidopsis thaliana Proline-rich extensin-like family protein mRNA, complete cds
AT2G40100	-1.652976476	2.55E-139	Arabidopsis thaliana light harvesting complex photosystem II mRNA, complete cds
AT3G54580	-1.709820619	1.41E-131	Arabidopsis thaliana chromosome 3, complete sequence
AT1G07200	-1.419268449	1.29E-127	Arabidopsis thaliana protein SMAX1-like 6 mRNA, complete cds
AT5G03555	-1.623614246	1.80E-125	Arabidopsis thaliana chromosome 5 sequence
AT3G15354	-1.350647632	2.16E-121	Arabidopsis thaliana protein SPA1-related 3 mRNA, complete cds
AT5G06530	-1.259013669	1.12E-113	Arabidopsis thaliana ABC transporter G family member 22 mRNA, complete cds
AT2G28630	-2.082636038	4.56E-112	Arabidopsis thaliana chromosome 2, complete sequence
AT2G28290	-1.024199048	6.69E-111	Arabidopsis thaliana chromatin structure-remodeling complex protein SYD mRNA, complete cds
AT3G41979	-1.725439005	1.52E-110	Erysimum belvederense internal transcribed spacer 1, partial sequence; 5.8S ribosomal RNA gene, complete sequence; and internal transcribed spacer 2, partial sequence
AT5G33355	-2.942743707	5.57E-109	Arabidopsis thaliana defensin-like protein mRNA, complete cds
AT3G54590	-2.009007654	1.49E-108	Arabidopsis thaliana hydroxyproline-rich

			glycoprotein mRNA, complete cds
AT2G34620	-1.382639417	4.51E-107	Arabidopsis thaliana mitochondrial transcription termination factor family protein mRNA, complete cds
AT3G28550	-1.723663884	2.46E-106	Arabidopsis thaliana chromosome 3, complete sequence
AT1G75100	-1.238259485	8.39E-102	Arabidopsis thaliana J-domain protein required for chloroplast accumulation response 1 mRNA, complete cds
AT3G60300	-1.259038005	2.46E-100	Arabidopsis thaliana RWD domain-containing protein mRNA, complete cds
AT1G68440	-1.169586184	4.17E-100	Arabidopsis thaliana chromosome 1 sequence
AT3G59930	-2.553091527	1.04E-97	Arabidopsis thaliana defensin-like protein 206 mRNA, complete cds
AT2G21650	-3.027362133	3.52E-94	Arabidopsis thaliana MYB transcription factor RSM1 mRNA, complete cds
AT1G23090	-1.133803222	4.71E-93	Arabidopsis thaliana sulfate transporter 3.3 mRNA, complete cds
AT3G22120	-1.50831434	7.64E-92	Arabidopsis thaliana chromosome 3, complete sequence
AT4G25290	-1.387570913	1.07E-91	Arabidopsis thaliana DNA photolyase mRNA, complete cds
AT3G06070	-1.767937139	2.44E-91	Arabidopsis thaliana uncharacterized protein mRNA, complete cds
AT5G21222	-1.194434374	3.27E-90	Arabidopsis thaliana pentatricopeptide repeat and serine/threonine kinase domain-containing protein mRNA, complete cds
AT3G56290	-1.360173062	5.52E-90	Arabidopsis thaliana mRNA for hypothetical protein, complete cds, clone: RAFL14-54-O14
AT3G01770	-1.272865366	6.22E-90	Arabidopsis thaliana bromodomain and extraterminal domain protein 10 mRNA, complete cds
AT4G37590	-1.255420244	2.71E-89	Arabidopsis thaliana protein NAKED PINS IN YUC MUTANTS 5 mRNA, complete cds
AT1G13640	-1.279399617	7.25E-88	Arabidopsis thaliana phosphatidylinositol 4-kinase gamma 6 mRNA, complete cds
AT1G49130	-2.528798274	3.85E-87	Arabidopsis thaliana zinc finger protein CONSTANS-LIKE 8 mRNA, complete cds
AT4G22570	-1.071769378	1.18E-82	Arabidopsis thaliana adenine phosphoribosyl transferase 3 mRNA,

			complete cds
AT4G15430	-1.804931346	8.51E-82	Arabidopsis thaliana ERD (early-responsive to dehydration stress) family protein mRNA, complete cds
AT1G17360	-1.110657355	8.97E-81	Arabidopsis thaliana uncharacterized protein mRNA, complete cds
AT4G22490	-1.596735999	3.68E-79	Arabidopsis thaliana chromosome 4 sequence
AT1G23720	-1.038160871	7.09E-79	Arabidopsis thaliana chromosome 1 sequence
AT1G15740	-1.029866357	9.06E-79	Arabidopsis thaliana leucine-rich repeat-containing protein mRNA, complete cds
AT1G30250	-1.681945341	1.24E-78	Arabidopsis thaliana chromosome 1 sequence
AT1G49200	-2.974959519	7.94E-78	Arabidopsis thaliana chromosome 1 sequence
AT2G41250	-1.23867504	7.93E-77	Arabidopsis thaliana haloacid dehalogenase-like hydrolase domain-containing protein mRNA, complete cds
AT1G49010	-1.673955806	4.64E-74	Arabidopsis thaliana myb/SANT-like DNA-binding domain-containing protein mRNA, complete cds
AT1G68190	-1.39383737	1.23E-71	Arabidopsis thaliana putative zinc finger protein mRNA, complete cds
AT5G58770	-1.142282151	3.65E-71	Arabidopsis thaliana dehydrodolichyl diphosphate synthase 2 mRNA, complete cds
AT1G72410	-1.072076881	1.61E-70	Arabidopsis thaliana COP1-interacting protein-related protein mRNA, complete cds
AT5G39080	-1.408350221	4.40E-67	Arabidopsis thaliana chromosome 5 sequence
AT3G02690	-1.192409455	4.23E-66	Arabidopsis thaliana nucleotide/sugar transporter family protein mRNA, complete cds
AT2G18790	-1.022952096	2.50E-65	Arabidopsis thaliana phytochrome B mRNA, complete cds
AT2G15020	-1.214683498	7.70E-65	Arabidopsis thaliana chromosome 2, complete sequence
AT2G31380	-1.038824454	4.25E-63	Arabidopsis thaliana B-box zinc finger protein STH mRNA, complete cds
AT5G52900	-1.891446023	1.22E-62	Arabidopsis thaliana

			membrane-associated kinase regulator family protein mRNA, complete cds
AT2G40300	-1.392495425	2.11E-59	Arabidopsis thaliana ferritin 4 mRNA, complete cds
AT2G04160	-1.193681178	4.24E-58	Arabidopsis thaliana subtilisin-like serine endopeptidase family protein mRNA, complete cds
AT5G47450	-1.089047196	5.76E-57	Arabidopsis thaliana aquaporin TIP2-3 mRNA, complete cds
AT2G33250	-1.376184925	5.96E-57	Arabidopsis thaliana uncharacterized protein mRNA, complete cds
AT1G20390	-2.160978244	3.62E-56	Arabidopsis thaliana chromosome 1 sequence
AT5G51010	-1.31030643	5.26E-56	Arabidopsis thaliana rubredoxin-like protein mRNA, complete cds
AT1G02110	-1.468172374	1.29E-55	Arabidopsis thaliana uncharacterized protein mRNA, complete cds
AT3G22790	-1.239065022	2.45E-55	Arabidopsis thaliana protein NETWORKED 1A mRNA, complete cds
AT3G16670	-1.0721701	7.16E-55	Arabidopsis thaliana pollen Ole e 1 allergen and extensin family protein mRNA, complete cds
AT5G41900	-2.149040689	2.57E-52	Arabidopsis thaliana hydrolase, alpha/beta fold family protein mRNA, complete cds
AT1G64380	-2.069901328	5.90E-52	Arabidopsis thaliana chromosome 1 sequence
AT5G46890	-1.91656375	7.67E-52	Arabidopsis thaliana chromosome 5 sequence
AT2G32540	-1.253872094	1.31E-51	Arabidopsis thaliana cellulose synthase-like protein B4 mRNA, complete cds
AT1G06830	-1.886951383	1.37E-51	Arabidopsis thaliana chromosome 1 sequence
AT5G14760	-1.475641525	2.11E-51	Arabidopsis thaliana L-aspartate oxidase mRNA, complete cds
AT3G14940	-1.039880535	2.50E-51	Arabidopsis thaliana phosphoenolpyruvate carboxylase 3 mRNA, complete cds
AT3G17609	-1.300084887	2.96E-51	Arabidopsis thaliana transcription factor HY5-like protein mRNA, complete cds
AT1G62960	-1.008072289	9.39E-51	Arabidopsis thaliana putative aminotransferase ACS10 mRNA, complete cds

AT2G31560	-1.100277475	1.35E-50	Arabidopsis thaliana uncharacterized protein mRNA, complete cds
AT5G10430	-1.017869719	2.39E-50	Arabidopsis thaliana chromosome 5 sequence
AT5G39660	-1.209842427	4.34E-49	Arabidopsis thaliana cycling DOF factor 2 mRNA, complete cds
AT1G78510	-1.11149632	5.85E-49	Arabidopsis thaliana solanesyl diphosphate synthase 1 mRNA, complete cds
AT1G71340	-1.172407608	1.57E-48	Arabidopsis thaliana glycerophosphodiester phosphodiesterase mRNA, complete cds
AT5G59750	-1.169181975	8.56E-48	Arabidopsis thaliana monofunctional riboflavin biosynthesis protein RIBA 3 mRNA, complete cds
AT5G01600	-1.801905506	1.86E-47	Arabidopsis thaliana ferretin 1 mRNA, complete cds
AT2G30210	-1.435449916	3.77E-46	Arabidopsis thaliana laccase 3 mRNA, complete cds
AT1G76930	-1.131284218	4.55E-46	Arabidopsis thaliana extensin 4 mRNA, complete cds
AT2G16586	-1.565731707	1.30E-45	Arabidopsis thaliana chromosome 2, complete sequence
AT2G02450	-1.630891541	1.48E-45	Arabidopsis thaliana protein LONG VEGETATIVE PHASE 1 mRNA, complete cds
AT5G23050	-1.151374518	2.08E-45	Arabidopsis thaliana acyl-activating enzyme 17 mRNA, complete cds
AT4G22505	-2.382264559	7.20E-45	Arabidopsis thaliana chromosome 4 sequence
AT2G41310	-1.014407546	1.73E-44	Arabidopsis thaliana mRNA for putative two-component response regulator 3 protein, complete cds, clone: RAFL14-76-K16
AT5G62130	-1.202499673	1.70E-43	Arabidopsis thaliana Per1-like family protein mRNA, complete cds
AT5G56860	-1.051812912	3.03E-43	Arabidopsis thaliana GATA transcription factor 21 mRNA, complete cds
AT5G51720	-1.750297921	1.19E-42	Arabidopsis thaliana CDGSH iron-sulfur domain-containing protein NEET mRNA, complete cds
AT5G19730	-1.386014887	1.33E-42	Arabidopsis thaliana probable pectinesterase 53 mRNA, complete cds
AT5G43360	-3.049374608	4.33E-42	Arabidopsis thaliana putative inorganic

			phosphate transporter 1-3 mRNA, complete cds
AT5G54130	-1.088418049	1.54E-41	Arabidopsis thaliana calcium-binding endonuclease/exonuclease/phosphatase family protein mRNA, complete cds
AT3G19050	-1.136822921	3.29E-41	Arabidopsis thaliana phragmoplast orienting kinesin 2 mRNA, complete cds
AT4G18740	-1.390997875	2.32E-40	Arabidopsis thaliana Rho termination factor mRNA, complete cds
AT3G57040	-1.050958132	5.05E-40	Arabidopsis thaliana two-component response regulator ARR9 mRNA, complete cds
AT5G18140	-1.093786455	9.78E-40	Arabidopsis thaliana chaperone DnaJ-domain containing protein mRNA, complete cds
AT4G27030	-1.168518495	2.53E-39	Arabidopsis thaliana chromosome 4 sequence
AT2G40670	-1.671771176	4.24E-39	Arabidopsis thaliana two-component response regulator ARR16 mRNA, complete cds
AT1G30520	-1.023189921	1.56E-38	Arabidopsis thaliana 2-succinylbenzoate--CoA ligase mRNA, complete cds
AT5G44660	-1.237993925	7.49E-38	Arabidopsis thaliana uncharacterized protein mRNA, complete cds
AT5G42760	-1.606507776	2.43E-37	Arabidopsis thaliana leucine carboxyl methyltransferase mRNA, complete cds
AT1G01190	-1.174090178	2.81E-37	Arabidopsis thaliana cytochrome P450, family 78, subfamily A, polypeptide 8 mRNA, complete cds
AT5G58310	-1.44951783	5.78E-35	Arabidopsis thaliana methyl esterase 18 mRNA, complete cds
AT5G64570	-1.137513157	7.10E-35	Arabidopsis thaliana beta-D-xylosidase 4 mRNA, complete cds
AT4G21870	-1.437820026	7.90E-35	Arabidopsis thaliana heat shock protein class V 15.4 mRNA, complete cds
AT2G36885	-1.136148606	1.40E-34	Arabidopsis thaliana uncharacterized protein mRNA, complete cds
AT3G10405	-1.037344111	1.20E-33	Arabidopsis thaliana uncharacterized protein mRNA, complete cds
AT1G21140	-2.590046248	2.07E-33	Arabidopsis thaliana chromosome 1 sequence
AT1G22890	-1.301451875	3.97E-33	Arabidopsis thaliana uncharacterized protein mRNA, complete cds

AT1G72820	-1.14416066	8.29E-33	Arabidopsis thaliana chromosome 1 sequence
AT4G37560	-1.045443996	9.13E-33	Arabidopsis thaliana acetamidase/formamidase family protein mRNA, complete cds
AT3G49160	-1.893068548	1.26E-32	Arabidopsis thaliana pyruvate kinase-like protein mRNA, complete cds
AT5G56870	-1.007097011	1.52E-32	Arabidopsis thaliana beta-galactosidase 4 mRNA, complete cds
AT2G19650	-1.492421276	1.96E-32	Arabidopsis thaliana chromosome 2, complete sequence
AT1G28010	-1.628633213	2.69E-32	Arabidopsis thaliana ABC transporter B family member 14 mRNA, complete cds
AT4G24700	-2.030085726	9.42E-31	Arabidopsis thaliana chromosome 4 sequence
AT2G36255	-5.93160315	2.55E-30	Arabidopsis thaliana putative defensin-like protein 203 mRNA, complete cds
AT4G38060	-1.028703414	4.15E-30	Arabidopsis thaliana chromosome 4 sequence
AT3G50560	-1.958075361	4.99E-30	Arabidopsis thaliana Rossmann-fold NAD(P)-binding domain-containing protein mRNA, complete cds
AT4G30470	-1.052112992	7.85E-30	Arabidopsis thaliana cinnamoyl-CoA reductase like protein mRNA, complete cds
AT5G46900	-1.546372473	8.76E-30	Arabidopsis thaliana chromosome 5 sequence
AT2G28305	-1.110160257	1.65E-29	Arabidopsis thaliana cytokinin riboside 5'-monophosphate phosphoribohydrolase LOG1 mRNA, complete cds
AT2G31150	-1.118913551	1.85E-29	Arabidopsis thaliana ATP binding / ATPase mRNA, complete cds
AT4G24120	-1.334309847	2.27E-29	Arabidopsis thaliana metal-nicotianamine transporter YSL1 mRNA, complete cds
AT2G46250	-1.237833125	5.12E-29	Arabidopsis thaliana myosin heavy chain-like protein mRNA, complete cds
AT5G11260	-1.175846292	1.23E-28	Arabidopsis thaliana transcription factor HY5 mRNA, complete cds
AT1G16260	-1.014310959	3.45E-28	Arabidopsis thaliana wall-associated receptor kinase-like 8 mRNA, complete cds

AT1G72030	-1.069689463	1.38E-27	Arabidopsis thaliana Acyl-CoA N-acyltransferases-like protein mRNA, complete cds
AT1G26790	-2.59355539	1.66E-27	Arabidopsis thaliana Dof-type zinc finger DNA-binding family protein mRNA, complete cds
AT2G32800	-1.018218792	2.36E-27	Arabidopsis thaliana chromosome 2, complete sequence
AT1G01580	-1.461955848	5.36E-27	Arabidopsis thaliana ferric reduction oxidase 2 mRNA, complete cds
AT1G69252	-1.022470603	1.13E-26	Arabidopsis thaliana chromosome 1 sequence
AT5G11070	-1.061424333	2.37E-26	Arabidopsis thaliana chromosome 5 sequence
AT2G24980	-1.53008679	3.90E-26	Arabidopsis thaliana chromosome 2, complete sequence
AT3G15770	-1.197716949	3.22E-25	Arabidopsis thaliana uncharacterized protein mRNA, complete cds
AT1G65490	-1.285672632	4.53E-25	Arabidopsis thaliana uncharacterized protein mRNA, complete cds
AT5G66052	-1.090300896	5.45E-25	Arabidopsis thaliana uncharacterized protein mRNA, complete cds
AT5G05860	-1.100000682	6.22E-25	Arabidopsis thaliana cytokinin-N-glucosyltransferase 2 mRNA, complete cds
AT1G34047	-5.039662896	1.94E-24	Arabidopsis thaliana defensin-like protein 208 mRNA, complete cds
AT5G47610	-1.635576918	2.84E-24	Arabidopsis thaliana chromosome 5 sequence
AT5G25130	-1.094253488	8.04E-24	Arabidopsis thaliana cytochrome P450 71B12 mRNA, complete cds
AT1G12040	-1.683096124	1.27E-23	Arabidopsis thaliana chromosome 1 sequence
AT1G13420	-1.798831147	2.29E-23	Arabidopsis thaliana chromosome 1 sequence
AT3G17360	-1.061890103	8.35E-23	Arabidopsis thaliana phragmoplast orienting kinesin 1 mRNA, complete cds
AT2G22610	-1.265026884	2.39E-22	Arabidopsis thaliana Di-glucose binding protein with Kinesin motor domain mRNA, complete cds
AT1G15410	-1.024712554	2.40E-22	Arabidopsis thaliana aspartate-glutamate racemase-like protein mRNA, complete cds
AT5G06640	-1.953629456	5.13E-22	Arabidopsis thaliana chromosome 5

			sequence
AT2G38980	-1.084375476	9.75E-22	Arabidopsis thaliana chromosome 2, complete sequence
AT2G25050	-1.135743867	1.37E-21	Arabidopsis thaliana actin-binding FH2 protein mRNA, complete cds
AT5G02120	-1.169288127	1.99E-21	Arabidopsis thaliana one helix protein mRNA, complete cds
AT3G21870	-1.570453745	6.29E-21	Arabidopsis thaliana cyclin p2;1 mRNA, complete cds
AT1G15550	-1.160064407	7.66E-21	Arabidopsis thaliana gibberellin 3-beta-dioxygenase 1 mRNA, complete cds
AT1G15830	-2.167670508	2.43E-20	Arabidopsis thaliana chromosome 1 sequence
AT5G23480	-1.98407057	5.15E-20	Arabidopsis thaliana SWIB/MDM2, Plus-3 and GYF domain-containing protein mRNA, complete cds
AT1G70000	-1.179289591	5.62E-20	Arabidopsis thaliana myb-like transcription factor mRNA, complete cds
AT4G19690	-1.267715255	1.02E-19	Arabidopsis thaliana Fe(2+) transport protein 1 mRNA, complete cds
AT3G62680	-1.206116564	2.87E-19	Arabidopsis thaliana proline-rich protein 3 mRNA, complete cds
AT1G23000	-1.28774696	3.33E-19	Arabidopsis thaliana heavy-metal-associated domain-containing protein mRNA, complete cds
AT5G35777	-1.950711973	3.40E-19	Arabidopsis thaliana chromosome 5 sequence
AT5G04290	-1.074343322	4.16E-19	Arabidopsis thaliana kow domain-containing transcription factor 1 mRNA, complete cds
AT3G15310	-1.749399819	6.66E-19	Arabidopsis thaliana chromosome 3, complete sequence
AT1G76800	-2.236763031	1.25E-18	Arabidopsis thaliana chromosome 1 sequence
AT3G22142	-1.565280936	1.47E-18	Arabidopsis thaliana chromosome 3, complete sequence
AT1G79840	-1.108312756	3.46E-18	Arabidopsis thaliana homeobox-leucine zipper protein GLABRA 2 mRNA, complete cds
AT2G42975	-1.128250158	4.02E-18	Arabidopsis thaliana uncharacterized protein mRNA, complete cds
AT2G24700	-1.39208362	5.26E-18	Arabidopsis thaliana transcriptional

			factor B3 family protein mRNA, complete cds
AT3G25590	-1.328328771	8.12E-18	Arabidopsis thaliana chromosome 3, complete sequence
AT4G24972	-1.138094354	1.45E-17	Arabidopsis thaliana protein TAPETUM DETERMINANT 1 mRNA, complete cds
AT5G56840	-1.378349509	1.91E-17	Arabidopsis thaliana myb-like transcription factor family protein mRNA, complete cds
AT1G78865	-1.439750054	4.06E-17	Arabidopsis thaliana chromosome 1 sequence
AT5G24870	-1.187651125	6.01E-17	Arabidopsis thaliana RING/U-box superfamily protein mRNA, complete cds
AT1G66230	-1.38898694	6.22E-17	Arabidopsis thaliana myb domain protein 20 mRNA, complete cds
AT1G35660	-1.028504133	1.35E-16	Arabidopsis thaliana uncharacterized protein mRNA, complete cds
AT3G49790	-1.147770951	1.48E-16	Arabidopsis thaliana chromosome 3, complete sequence
AT5G66580	-1.431032836	3.53E-16	Arabidopsis thaliana chromosome 5 sequence
AT2G44300	-1.314868303	5.52E-16	Arabidopsis thaliana bifunctional inhibitor/lipid-transfer protein/seed storage 2S albumin superfamily protein mRNA, complete cds
AT4G15160	-1.550174043	8.96E-16	Arabidopsis thaliana bifunctional inhibitor/lipid-transfer protein/seed storage 2S albumin-like protein mRNA, complete cds
AT1G63570	-1.396681332	1.19E-15	Arabidopsis thaliana chromosome 1 sequence
AT4G26150	-1.059269776	1.48E-15	Arabidopsis thaliana putative GATA transcription factor 22 mRNA, complete cds
AT2G04790	-1.116882398	2.06E-15	Arabidopsis thaliana uncharacterized protein mRNA, complete cds
AT4G38960	-1.118139418	2.95E-15	Arabidopsis thaliana B-box type zinc finger-containing protein mRNA, complete cds
AT4G37320	-1.080565789	4.48E-15	Arabidopsis thaliana cytochrome P450, family 81, subfamily D, polypeptide 5 mRNA, complete cds
AT3G08040	-1.816907669	7.31E-15	Arabidopsis thaliana protein FERRIC REDUCTASE DEFECTIVE 3 mRNA,

			complete cds
AT2G26710	-1.291499095	1.12E-14	Arabidopsis thaliana cytochrome P450 734A1 mRNA, complete cds
AT3G25190	-1.011773499	2.41E-14	Arabidopsis thaliana vacuolar iron transporter homolog 2.1 mRNA, complete cds
AT1G70430	-1.320168438	3.01E-14	Arabidopsis thaliana protein kinase mRNA, complete cds
AT3G12900	-2.077179974	4.36E-14	Arabidopsis thaliana oxidoreductase, 2OG-Fe(II) oxygenase family protein mRNA, complete cds
AT4G19430	-1.458469251	6.30E-14	Arabidopsis thaliana chromosome 4 sequence
AT5G15840	-1.439750054	6.85E-14	Arabidopsis thaliana chromosome 5 sequence
AT3G10310	-1.020139824	8.12E-14	Arabidopsis thaliana myosin and kinesin motor and CH domain-containing protein mRNA, complete cds
AT1G58807	-1.043948773	9.16E-14	Arabidopsis thaliana putative disease resistance protein RDL5 mRNA, complete cds
AT5G23730	-1.255756846	1.12E-13	Arabidopsis thaliana chromosome 5 sequence
AT5G15830	-1.102117058	2.18E-13	Arabidopsis thaliana chromosome 5 sequence
AT2G01010	-1.640701951	5.32E-13	Arabidopsis thaliana chromosome 2, complete sequence
AT5G53200	-1.619870822	7.17E-13	Arabidopsis thaliana transcription factor TRY mRNA, complete cds
AT3G24460	-1.000786715	7.94E-13	Arabidopsis thaliana Serinc-domain containing serine and sphingolipid biosynthesis protein mRNA, complete cds
AT3G06433	-1.029819445	9.75E-13	Arabidopsis thaliana chromosome 3, complete sequence
AT5G02440	-1.366818531	1.06E-12	Arabidopsis thaliana uncharacterized protein mRNA, complete cds
AT2G40030	-1.063333848	1.37E-12	Arabidopsis thaliana nuclear RNA polymerase D1B mRNA, complete cds
AT2G22420	-1.069450869	1.45E-12	Arabidopsis thaliana peroxidase mRNA, complete cds
AT2G26215	-1.298360634	1.75E-12	Arabidopsis thaliana chromosome 2, complete sequence
AT1G68110	-1.745996526	2.31E-12	Arabidopsis thaliana chromosome 1 sequence

AT2G33240	-1.734122426	3.59E-12	Arabidopsis thaliana myosin XI D mRNA, complete cds
AT1G10610	-1.052836155	3.66E-12	Arabidopsis thaliana transcription factor bHLH90 mRNA, complete cds
AT1G31310	-1.350816325	4.50E-12	Arabidopsis thaliana hydroxyproline-rich glycoprotein-like protein mRNA, complete cds
AT5G14090	-1.146703079	5.40E-12	Arabidopsis thaliana uncharacterized protein mRNA, complete cds
AT2G01020	-1.194637556	6.05E-12	Erysimum belvederense internal transcribed spacer 1, partial sequence; 5.8S ribosomal RNA gene, complete sequence; and internal transcribed spacer 2, partial sequence
AT3G13065	-1.510139381	1.28E-11	Arabidopsis thaliana STRUBBELIG-receptor family 4 mRNA, complete cds
AT4G35420	-1.135743867	1.49E-11	Arabidopsis thaliana dihydroflavonol 4-reductase-like1 mRNA, complete cds
AT5G02670	-1.003017483	1.56E-11	Arabidopsis thaliana uncharacterized protein mRNA, complete cds
AT5G40310	-6.417029977	1.66E-11	Arabidopsis thaliana exonuclease family protein mRNA, complete cds
AT1G29430	-1.042342448	1.68E-11	Arabidopsis thaliana chromosome 1 sequence
AT5G06630	-1.183911149	1.70E-11	Arabidopsis thaliana chromosome 5 sequence
AT5G41761	-2.346640649	2.72E-11	Arabidopsis thaliana chromosome 5 sequence
AT3G18010	-1.731665579	2.88E-11	Arabidopsis thaliana WUSCHEL-related homeobox 1 mRNA, complete cds
AT2G26180	-1.252518472	3.57E-11	Arabidopsis thaliana IQ-domain 6 protein mRNA, complete cds
AT4G15290	-2.272640068	4.77E-11	Arabidopsis thaliana cellulose synthase-like protein B5 mRNA, complete cds
AT2G45900	-1.429696389	5.07E-11	Arabidopsis thaliana phosphatidylinositol N-acetyglucosaminlytransferase subunit P-like protein mRNA, complete cds
AT4G38070	-1.13545962	6.11E-11	Arabidopsis thaliana transcription factor bHLH131 mRNA, complete cds
AT5G55930	-1.101210692	6.28E-11	Arabidopsis thaliana oligopeptide transporter 1 mRNA, complete cds
AT2G43890	-1.609675055	8.91E-11	Arabidopsis thaliana putative

			polygalacturonase /pectinase mRNA, complete cds
AT1G31320	-1.071579159	8.99E-11	Arabidopsis thaliana LOB domain-containing protein 4 mRNA, complete cds
AT3G21330	-2.565280936	9.38E-11	Arabidopsis thaliana transcription factor bHLH87 mRNA, complete cds
AT1G29600	-1.415733172	1.29E-10	Arabidopsis thaliana putative zinc finger CCCH domain-containing protein 10 mRNA, complete cds
AT4G37810	-2.214537113	2.22E-10	Arabidopsis thaliana uncharacterized protein mRNA, complete cds
AT4G31380	-3.516565651	2.73E-10	Arabidopsis thaliana chromosome 4 sequence
AT3G13440	-1.157467763	3.18E-10	Arabidopsis thaliana S-adenosyl-L-methionine-dependent methyltransferase-like protein mRNA, complete cds
AT4G08100	-1.808983863	3.46E-10	Arabidopsis thaliana chromosome 4 sequence
AT4G22485	-1.221109767	4.14E-10	Arabidopsis thaliana chromosome 4 sequence
AT1G01520	-1.771946484	4.95E-10	Arabidopsis thaliana transcription factor ASG4 mRNA, complete cds
AT1G09470	-1.053001599	5.70E-10	Arabidopsis thaliana uncharacterized protein mRNA, complete cds
AT5G47810	-1.038518354	6.09E-10	Arabidopsis thaliana 6-phosphofructokinase 2 mRNA, complete cds
AT4G01430	-1.132241019	7.17E-10	Arabidopsis thaliana nodulin MtN21-like transporter family protein mRNA, complete cds
AT5G18340	-2.393946364	8.89E-10	Arabidopsis thaliana U-box domain-containing protein 48 mRNA, complete cds
AT4G02970	-1.250272255	9.99E-10	Arabidopsis thaliana signal recognition particle mRNA, complete cds
AT4G08115	-1.84914099	1.12E-09	Arabidopsis thaliana chromosome 4 sequence
AT5G49330	-1.017759794	1.52E-09	Arabidopsis thaliana myb domain protein 111 mRNA, complete cds
AT3G21150	-1.080360584	1.99E-09	Arabidopsis thaliana chromosome 3, complete sequence
AT2G28200	-1.031980555	2.07E-09	Arabidopsis thaliana chromosome 2,

			complete sequence
AT5G15600	-1.163987343	2.25E-09	Arabidopsis thaliana protein SPIRAL1-like4 mRNA, complete cds
AT3G04450	-1.23725719	2.87E-09	Arabidopsis thaliana myb family transcription factor mRNA, complete cds
AT1G15825	-1.841000601	3.06E-09	Arabidopsis thaliana chromosome 1 sequence
AT3G18900	-1.064240918	3.12E-09	Arabidopsis thaliana uncharacterized protein mRNA, complete cds
AT5G27890	-1.048299375	4.84E-09	Arabidopsis thaliana uncharacterized protein mRNA, complete cds
AT1G50290	-1.580231277	5.68E-09	Arabidopsis thaliana chromosome 1 sequence
AT5G06790	-1.048559296	5.81E-09	Arabidopsis thaliana chromosome 5 sequence
AT1G59218	-1.658584655	6.23E-09	Arabidopsis thaliana putative disease resistance protein RDL6/RF9 mRNA, complete cds
AT1G49490	-1.609675055	7.42E-09	Arabidopsis thaliana chromosome 1 sequence
AT5G13790	-1.832067476	8.37E-09	Arabidopsis thaliana agamous-like MADS-box protein AGL15 mRNA, complete cds
AT2G34360	-1.405802722	9.47E-09	Arabidopsis thaliana MATE efflux family protein mRNA, complete cds
AT5G25970	-3.310114773	9.82E-09	Arabidopsis thaliana core-2/I-branching beta-1,6-N-acetylglucosaminyltransferase family protein mRNA, complete cds
AT3G15620	-1.247104976	1.17E-08	Arabidopsis thaliana (6-4)DNA photolyase mRNA, complete cds
AT4G12510	-1.127074272	3.50E-08	Arabidopsis thaliana chromosome 4 sequence
AT3G06020	-1.168548327	3.83E-08	Arabidopsis thaliana chromosome 3, complete sequence
AT1G52565	-1.104882903	4.67E-08	Arabidopsis thaliana uncharacterized protein mRNA, complete cds
AT1G15160	-1.224021362	5.73E-08	Arabidopsis thaliana MATE efflux family protein mRNA, complete cds
AT2G46730	-1.397521818	5.75E-08	Arabidopsis thaliana chromosome 2, complete sequence
AT1G74890	-1.052909446	7.60E-08	Arabidopsis thaliana two-component response regulator ARR15 mRNA, complete cds
AT1G16330	-1.052909446	7.60E-08	Arabidopsis thaliana cyclin B3-1 mRNA,

			complete cds
AT2G01940	-1.033814761	8.12E-08	Arabidopsis thaliana protein shoot gravitropism 5 mRNA, complete cds
AT3G45060	-1.063706686	9.64E-08	Arabidopsis thaliana high affinity nitrate transporter 2.6 mRNA, complete cds
AT3G30720	-2.912237825	1.41E-07	Arabidopsis thaliana qua-quine starch mRNA, complete cds
AT2G41150	-1.044077879	1.50E-07	Arabidopsis thaliana uncharacterized protein mRNA, complete cds
AT4G30570	-1.649203419	1.54E-07	Arabidopsis thaliana Glucose-1-phosphate adenylyltransferase family protein mRNA, complete cds
AT1G55200	-1.127398218	1.75E-07	Arabidopsis thaliana protein kinase protein with adenine nucleotide alpha hydrolases-like domain mRNA, complete cds
AT2G39560	-1.23416592	2.36E-07	Arabidopsis thaliana chromosome 2, complete sequence
AT1G52800	-1.673805392	3.04E-07	Arabidopsis thaliana oxidoreductase, 2OG-Fe(II) oxygenase family protein mRNA, complete cds
AT1G49940	-1.698484322	3.42E-07	Arabidopsis thaliana uncharacterized protein mRNA, complete cds
AT3G27970	-1.698484322	3.42E-07	Arabidopsis thaliana exonuclease-like protein mRNA, complete cds
AT5G02020	-1.024712554	4.02E-07	Arabidopsis thaliana uncharacterized protein mRNA, complete cds
AT5G18010	-2.150243436	4.37E-07	Arabidopsis thaliana chromosome 5 sequence
AT3G20200	-1.188211287	4.70E-07	Arabidopsis thaliana Protein kinase protein with adenine nucleotide alpha hydrolases-like domain mRNA, complete cds
AT5G38780	-3.779600056	5.12E-07	Arabidopsis thaliana putative S-adenosylmethionine-dependent methyltransferase mRNA, complete cds
AT3G28770	-1.517752566	5.53E-07	Arabidopsis thaliana uncharacterized protein mRNA, complete cds
AT1G05640	-1.215396116	6.83E-07	Arabidopsis thaliana ankyrin repeats-containing protein mRNA, complete cds
AT1G56680	-1.293899187	8.02E-07	Arabidopsis thaliana Chitinase family protein mRNA, complete cds
AT2G15810	-1.654762945	8.23E-07	Arabidopsis thaliana chromosome 2,

			complete sequence
AT2G17036	-1.654762945	8.23E-07	Arabidopsis thaliana F-box protein mRNA, complete cds
AT5G03790	-2.609675055	9.41E-07	Arabidopsis thaliana homeobox 51 mRNA, complete cds
AT5G46500	-1.11371756	1.01E-06	Arabidopsis thaliana uncharacterized protein mRNA, complete cds
AT4G13572	-1.003650939	1.29E-06	Arabidopsis thaliana uncharacterized protein mRNA, complete cds
AT2G26520	-1.845742413	1.56E-06	Arabidopsis thaliana chromosome 2, complete sequence
AT4G08410	-1.587648749	1.71E-06	Arabidopsis thaliana chromosome 4 sequence
AT1G21810	-1.024712554	1.77E-06	Arabidopsis thaliana uncharacterized protein mRNA, complete cds
AT2G07042	-1.074465589	1.85E-06	Arabidopsis thaliana clone asmbl_5106 unknown mRNA sequence
AT1G04425	-1.153995571	1.88E-06	Arabidopsis thaliana clone 23398 mRNA sequence
AT2G31085	-1.329567136	1.90E-06	Arabidopsis thaliana chromosome 2, complete sequence
AT1G05690	-1.217357632	1.99E-06	Arabidopsis thaliana BTB and TAZ domain protein 3 mRNA, complete cds
AT3G07273	-2.129049214	2.01E-06	Arabidopsis thaliana chromosome 3, complete sequence
AT1G06100	-1.120471537	2.12E-06	Arabidopsis thaliana delta-9 desaturase-like 2 protein mRNA, complete cds
AT1G06310	-2.702784459	2.46E-06	Arabidopsis thaliana putative acyl-CoA oxidase mRNA, complete cds
AT1G03935	-1.861213822	2.70E-06	Arabidopsis thaliana chromosome 1 sequence
AT4G31805	-1.107174714	2.94E-06	Arabidopsis thaliana protein POLAR mRNA, complete cds
AT5G27250	-1.718609427	3.19E-06	Arabidopsis thaliana chromosome 5 sequence
AT2G04795	-1.162216078	3.26E-06	Arabidopsis thaliana chromosome 2, complete sequence
AT5G26320	-1.453555853	3.27E-06	Arabidopsis thaliana TRAF-like family protein mRNA, complete cds
AT4G04223	-1.484144173	4.45E-06	Arabidopsis thaliana ARM repeat superfamily protein mRNA, complete cds
AT1G07690	-1.878861688	4.68E-06	Arabidopsis thaliana uncharacterized protein mRNA, complete cds

AT1G05650	-1.247104976	5.82E-06	Arabidopsis thaliana pectin lyase-like protein mRNA, complete cds
AT2G26211	-1.636147266	6.85E-06	Arabidopsis thaliana chromosome 2, complete sequence
AT4G28720	-1.636147266	6.85E-06	Arabidopsis thaliana chromosome 4 sequence
AT2G45210	-1.024712554	7.83E-06	Arabidopsis thaliana chromosome 2, complete sequence
AT3G45080	-3.083606243	8.68E-06	Arabidopsis thaliana chromosome 3, complete sequence
AT5G49080	-1.137606611	9.25E-06	Arabidopsis thaliana chromosome 5 sequence
AT5G05510	-1.011773499	1.07E-05	Arabidopsis thaliana Mad3/BUB1 homology region 1 mRNA, complete cds
AT5G01190	-1.038518354	1.21E-05	Arabidopsis thaliana laccase 10 mRNA, complete cds
AT2G10931	-1.535674474	1.22E-05	Arabidopsis thaliana chromosome 2, complete sequence
AT1G52820	-1.122009756	1.29E-05	Arabidopsis thaliana putative 2-oxoglutarate-dependent dioxygenase mRNA, complete cds
AT5G35190	-1.357287893	1.33E-05	Arabidopsis thaliana chromosome 5 sequence
AT4G36515	-1.16557509	1.38E-05	Arabidopsis thaliana uncharacterized protein mRNA, complete cds
AT4G13260	-1.279969609	1.41E-05	Arabidopsis thaliana protein YUCCA2 mRNA, complete cds
AT2G20815	-1.086113099	1.43E-05	Arabidopsis thaliana uncharacterized protein mRNA, complete cds
AT5G56100	-1.024712554	1.65E-05	Arabidopsis thaliana chromosome 5 sequence
AT5G34800	-1.194637556	1.69E-05	Arabidopsis thaliana chromosome 5 sequence
AT3G53590	-1.510139381	1.85E-05	Arabidopsis thaliana putative leucine-rich repeat receptor-like serine/threonine-protein kinase mRNA, complete cds
AT5G04120	-5.112175396	1.88E-05	Arabidopsis thaliana phosphoglycerate mutase-like protein mRNA, complete cds
AT4G35210	-5.112175396	1.88E-05	Arabidopsis thaliana chromosome 4 sequence
AT1G66725	-1.172811193	1.99E-05	Arabidopsis thaliana chromosome 1 sequence
AT3G59130	-3.417029977	2.01E-05	Arabidopsis thaliana

			Cysteine/Histidine-rich C1 domain family protein mRNA, complete cds
AT3G09780	-1.03935933	2.12E-05	Arabidopsis thaliana chromosome 3, complete sequence
AT2G12460	-2.024712554	2.55E-05	Arabidopsis thaliana chromosome 2, complete sequence
AT2G15350	-2.668568744	3.53E-05	Arabidopsis thaliana chromosome 2, complete sequence
AT4G38390	-1.137187284	3.94E-05	Arabidopsis thaliana protein root hair specific 17 mRNA, complete cds
AT4G34800	-1.912237825	3.95E-05	Arabidopsis thaliana chromosome 4 sequence
AT2G21010	-4.112175396	4.27E-05	Arabidopsis thaliana Calcium-dependent lipid-binding (CaLB domain) family protein mRNA, complete cds
AT3G03850	-1.39817095	4.54E-05	Arabidopsis thaliana chromosome 3, complete sequence
AT3G48770	-1.346640649	4.78E-05	Arabidopsis thaliana ATP/DNA binding protein mRNA, complete cds
AT5G57785	-1.261751752	5.02E-05	Arabidopsis thaliana chromosome 5 sequence
AT4G31330	-1.413754845	5.35E-05	Arabidopsis thaliana uncharacterized protein mRNA, complete cds
AT4G36050	-1.119039937	5.51E-05	Arabidopsis thaliana endonuclease/exonuclease/phosphatase family protein mRNA, complete cds
AT5G16410	-1.359131593	5.67E-05	Arabidopsis thaliana HXXXD-type acyl-transferase-like protein mRNA, complete cds
AT5G56080	-1.359131593	5.67E-05	Arabidopsis thaliana chromosome 5 sequence
AT3G11402	-1.009282514	5.76E-05	Arabidopsis thaliana cysteine/histidine-rich C1 domain-containing protein mRNA, complete cds
AT2G42980	-1.23282075	6.03E-05	Arabidopsis thaliana chromosome 2, complete sequence
AT2G01300	-1.430704914	6.30E-05	Arabidopsis thaliana chromosome 2, complete sequence
AT4G11460	-1.643622387	6.60E-05	Arabidopsis thaliana putative cysteine-rich receptor-like protein kinase 30 mRNA, complete cds
AT4G18490	-1.121574094	6.64E-05	Arabidopsis thaliana uncharacterized protein mRNA, complete cds

AT1G20400	-3.272640068	6.82E-05	Arabidopsis thaliana uncharacterized protein mRNA, complete cds
AT1G52570	-1.059477972	6.95E-05	Arabidopsis thaliana phospholipase D alpha 2 mRNA, complete cds
AT2G31081	-1.449210383	7.40E-05	Arabidopsis thaliana chromosome 2, complete sequence
AT3G59850	-1.681824841	7.47E-05	Arabidopsis thaliana pectin lyase-like superfamily protein mRNA, complete cds
AT2G36110	-1.387282634	7.95E-05	Arabidopsis thaliana chromosome 2, complete sequence
AT3G12970	-1.150243436	8.28E-05	Arabidopsis thaliana chromosome 3, complete sequence
AT3G04510	-1.247104976	8.65E-05	Arabidopsis thaliana chromosome 3, complete sequence
AT4G11490	-2.832067476	9.20E-05	Arabidopsis thaliana putative cysteine-rich receptor-like protein kinase 33 mRNA, complete cds
AT1G49210	-1.774734301	9.34E-05	Arabidopsis thaliana chromosome 1 sequence
AT1G06120	-1.403224178	9.39E-05	Arabidopsis thaliana delta-9 desaturase-like 3 protein mRNA, complete cds
AT3G24780	-1.127074272	9.65E-05	Arabidopsis thaliana chromosome 3, complete sequence
AT2G46970	-1.127074272	9.65E-05	Arabidopsis thaliana transcription factor PIL1 mRNA, complete cds
AT2G16230	-1.491838565	0.000101256	Arabidopsis thaliana O-glycosyl hydrolases family 17 protein mRNA, complete cds
AT3G63290	-1.008224431	0.000101472	Arabidopsis thaliana chromosome 3, complete sequence
AT3G60280	-2.54827451	0.00010963	Arabidopsis thaliana uclacyanin 3 mRNA, complete cds
ATCG00040	-1.420641231	0.000110747	Arabidopsis thaliana chloroplast DNA, complete genome, ecotype: Columbia
AT2G44630	-1.646200931	0.000116665	Arabidopsis thaliana chromosome 2, complete sequence
AT2G46840	-4.832067476	0.000116989	Arabidopsis thaliana uncharacterized protein mRNA, complete cds
AT5G40860	-4.832067476	0.000116989	Arabidopsis thaliana uncharacterized protein mRNA, complete cds
AT3G09595	-4.832067476	0.000116989	Arabidopsis thaliana chromosome 3, complete sequence
AT4G34770	-1.133237011	0.00014038	Arabidopsis thaliana chromosome 4

			sequence
AT4G02780	-2.761678148	0.000165027	Arabidopsis thaliana Ent-copalyl diphosphate synthase mRNA, complete cds
AT1G45545	-1.166731559	0.000174394	Arabidopsis thaliana uncharacterized protein mRNA, complete cds
AT3G63445	-1.20056239	0.000177775	Arabidopsis thaliana At3g63440 mRNA sequence
AT5G06900	-1.484144173	0.000178935	Arabidopsis thaliana cytochrome P450, family 93, subfamily D, polypeptide 1 mRNA, complete cds
AT3G19184	-1.649203419	0.000206806	Arabidopsis thaliana AP2/B3-like transcriptional factor family protein mRNA, complete cds
AT2G17330	-1.649203419	0.000206806	Arabidopsis thaliana chromosome 2, complete sequence
AT4G31610	-1.510139381	0.000208683	Arabidopsis thaliana B3 domain-containing protein REM1 mRNA, complete cds
AT4G12870	-1.065354539	0.000215876	Arabidopsis thaliana Gamma interferon responsive lysosomal thiol (GILT) reductase family protein mRNA, complete cds
AT5G44973	-3.112175396	0.000230117	Arabidopsis thaliana defensin-like protein 285 mRNA, complete cds
AT5G35914	-1.694563953	0.000234056	Arabidopsis thaliana chromosome 5 sequence
AT3G11750	-1.114910363	0.000237628	Arabidopsis thaliana Dihydroneopterin aldolase mRNA, complete cds
AT5G58360	-1.213746379	0.00025707	Arabidopsis thaliana chromosome 5 sequence
AT3G28455	-1.006096876	0.000261769	Arabidopsis thaliana protein CLAVATA3/ESR-related 25 mRNA, complete cds
AT1G52120	-3.832067476	0.000279979	Arabidopsis thaliana jacalin-like lectin domain-containing protein mRNA, complete cds
AT1G13610	-1.57220035	0.000280209	Arabidopsis thaliana alpha/beta-hydrolase-like protein mRNA, complete cds
AT5G45200	-1.808983863	0.000290398	Arabidopsis thaliana TIR-NBS-LRR class disease resistance protein mRNA, complete cds
AT5G54240	-1.317494303	0.000302614	Arabidopsis thaliana uncharacterized

			protein mRNA, complete cds
AT4G25707	-1.317494303	0.000302614	Arabidopsis thaliana chromosome 4 sequence
AT1G18180	-1.005603731	0.00031665	Arabidopsis thaliana uncharacterized protein mRNA, complete cds
AT5G34825	-2.417029977	0.00033515	Arabidopsis thaliana chromosome 5 sequence
AT2G18480	-1.972245134	0.000338084	Arabidopsis thaliana putative polyol transporter 3 mRNA, complete cds
AT3G59270	-2.226346415	0.00035233	Arabidopsis thaliana FBD domain-containing protein mRNA, complete cds
AT2G43480	-1.502759851	0.000370201	Arabidopsis thaliana probable peroxidase 26 mRNA, complete cds
AT1G05135	-1.417029977	0.000405885	Arabidopsis thaliana chromosome 1 sequence
AT3G05900	-4.609675055	0.000406055	Arabidopsis thaliana neurofilament protein-related protein mRNA, complete cds
AT2G14800	-1.28774696	0.000442551	Arabidopsis thaliana uncharacterized protein mRNA, complete cds
AT3G47710	-1.363514468	0.000510806	Arabidopsis thaliana atypical non-DNA binding bHLH protein BNQ3 mRNA, complete cds
AT2G28040	-1.832067476	0.000512879	Arabidopsis thaliana chromosome 2, complete sequence
AT4G39600	-1.300346997	0.000529402	Arabidopsis thaliana chromosome 4 sequence
AT5G26080	-2.024712554	0.000586598	Arabidopsis thaliana chromosome 5 sequence
AT3G27480	-2.162216078	0.000598823	Arabidopsis thaliana cysteine/histidine-rich C1 domain-containing protein mRNA, complete cds
AT2G45040	-2.162216078	0.000598823	Arabidopsis thaliana chromosome 2, complete sequence
AT3G28153	-1.382264559	0.000606366	Arabidopsis thaliana chromosome 3, complete sequence
AT4G22520	-1.167670508	0.000631032	Arabidopsis thaliana chromosome 4 sequence
AT1G80570	-1.073622155	0.000677166	Arabidopsis thaliana chromosome 1 sequence
AT4G11140	-1.102715066	0.00071111	Arabidopsis thaliana chromosome 4 sequence

AT1G52100	-1.403224178	0.000718354	Arabidopsis thaliana jacalin-like lectin domain-containing protein mRNA, complete cds
AT4G31020	-1.024712554	0.000728212	Arabidopsis thaliana alpha/beta-Hydrolases superfamily protein mRNA, complete cds
AT3G06630	-1.135743867	0.000740055	Arabidopsis thaliana protein kinase family protein mRNA, complete cds
AT3G49950	-1.329567136	0.00075542	Arabidopsis thaliana chromosome 3, complete sequence
AT1G76420	-1.329567136	0.00075542	Arabidopsis thaliana protein CUP-SHAPED COTYLEDON 3 mRNA, complete cds
AT5G60260	-4.484144173	0.000763212	Arabidopsis thaliana uncharacterized protein mRNA, complete cds
AT1G66650	-2.93160315	0.000771498	Arabidopsis thaliana E3 ubiquitin-protein ligase SINA-like 4 mRNA, complete cds
AT3G51325	-2.93160315	0.000771498	Arabidopsis thaliana chromosome 3, complete sequence
AT5G55470	-1.002686248	0.000824086	Arabidopsis thaliana sodium/hydrogen exchanger 4 mRNA, complete cds
AT2G17490	-1.779600056	0.000826067	Arabidopsis thaliana chromosome 2, complete sequence
AT3G46240	-1.10563255	0.000860845	Arabidopsis thaliana uncharacterized protein mRNA, complete cds
AT3G14540	-1.565280936	0.00089015	Arabidopsis thaliana terpenoid synthase 19 mRNA, complete cds
AT3G26040	-1.861213822	0.000906694	Arabidopsis thaliana chromosome 3, complete sequence
AT3G44970	-1.281052308	0.000926805	Arabidopsis thaliana cytochrome P450 family protein mRNA, complete cds
AT5G56810	-1.281052308	0.000926805	Arabidopsis thaliana putative F-box/FBD/LRR-repeat protein mRNA, complete cds
AT3G20850	-1.96331201	0.000973049	Arabidopsis thaliana chromosome 3, complete sequence
AT3G46340	-1.96331201	0.000973049	Arabidopsis thaliana putative receptor-like protein kinase mRNA, complete cds
AT4G21260	-3.609675055	0.000991508	Arabidopsis thaliana sulfite exporter TauE/SafE family protein mRNA, complete cds
AT5G38260	-3.609675055	0.000991508	Arabidopsis thaliana protein kinase family protein mRNA, complete cds

AT4G37780	-2.095101882	0.001012027	Arabidopsis thaliana myb domain protein 87 mRNA, complete cds
AT5G58280	-2.095101882	0.001012027	Arabidopsis thaliana AP2/B3-like transcriptional factor family protein mRNA, complete cds
AT1G68238	-1.024712554	0.001070005	Arabidopsis thaliana chromosome 1 sequence
AT5G09710	-1.365749472	0.001072307	Arabidopsis thaliana putative magnesium transporter MRS2-9 mRNA, complete cds
AT3G21890	-1.236216659	0.001121591	Arabidopsis thaliana chromosome 3, complete sequence
AT1G26390	-1.001253581	0.001211303	Arabidopsis thaliana chromosome 1 sequence
AT4G08040	-1.387282634	0.001274119	Arabidopsis thaliana 1-aminocyclopropane-1-carboxylate synthase 11 mRNA, complete cds
AT1G11340	-1.150243436	0.001309713	Arabidopsis thaliana G-type lectin S-receptor-like serine/threonine-protein kinase mRNA, complete cds
AT5G23770	-1.194637556	0.001340695	Arabidopsis thaliana uncharacterized protein mRNA, complete cds
AT2G22290	-2.832067476	0.001407575	Arabidopsis thaliana RAB GTPase-like protein H1D mRNA, complete cds
AT4G24204	-2.832067476	0.001407575	Arabidopsis thaliana RING-finger domain-containing protein mRNA, complete cds
AT1G68510	-4.346640649	0.001443683	Arabidopsis thaliana LOB domain-containing protein 42 mRNA, complete cds
AT5G13230	-1.802320133	0.001470863	Arabidopsis thaliana chromosome 5 sequence
AT3G11385	-1.802320133	0.001470863	Arabidopsis thaliana chromosome 3, complete sequence
AT5G39520	-1.802320133	0.001470863	Arabidopsis thaliana uncharacterized protein mRNA, complete cds
AT4G29450	-1.024712554	0.001574915	Arabidopsis thaliana leucine-rich repeat protein kinase-like protein mRNA, complete cds
AT1G79480	-1.024712554	0.001574915	Arabidopsis thaliana carbohydrate-binding X8 domain-containing protein mRNA, complete cds
AT1G29770	-1.155957088	0.001584883	Arabidopsis thaliana chromosome 1 sequence

AT5G27889	-1.560765454	0.001594759	Arabidopsis thaliana chromosome 5 sequence
AT1G61840	-1.203049796	0.001618672	Arabidopsis thaliana chromosome 1 sequence
AT1G04090	-2.024712554	0.001699833	Arabidopsis thaliana uncharacterized protein mRNA, complete cds
AT5G26720	-1.083606243	0.001772155	Arabidopsis thaliana chromosome 5 sequence
AT4G26890	-1.609675055	0.001839432	Arabidopsis thaliana chromosome 4 sequence
AT1G54470	-3.484144173	0.001870766	Arabidopsis thaliana resistance to Peronospora parasitica 27 mRNA, complete cds
AT5G53100	-3.484144173	0.001870766	Arabidopsis thaliana Rossmann-fold NAD(P)-binding domain-containing protein mRNA, complete cds
AT5G08150	-3.484144173	0.001870766	Arabidopsis thaliana chromosome 5 sequence
AT1G10550	-1.024712554	0.001912061	Arabidopsis thaliana probable xyloglucan endotransglucosylase/hydrolase protein 33 mRNA, complete cds
AT3G54870	-1.162216078	0.001918258	Arabidopsis thaliana armadillo repeat-containing kinesin-like protein 1 mRNA, complete cds
AT1G02350	-1.272640068	0.001952122	Arabidopsis thaliana protoporphyrinogen oxidase-like protein mRNA, complete cds
AT5G17040	-1.668568744	0.002101274	Arabidopsis thaliana UDP-glycosyltransferase 78D4 mRNA, complete cds
AT3G48940	-1.086113099	0.002150849	Arabidopsis thaliana Remorin-like protein mRNA, complete cds
AT2G35420	-1.086113099	0.002150849	Arabidopsis thaliana chromosome 2, complete sequence
AT1G14220	-1.124248228	0.002249136	Arabidopsis thaliana ribonuclease T2 mRNA, complete cds
AT5G03620	-1.368666955	0.002265445	Arabidopsis thaliana Subtilisin-like serine endopeptidase family protein mRNA, complete cds
AT4G15360	-1.169102464	0.002322204	Arabidopsis thaliana cytochrome P450, family 705, subfamily A, polypeptide 3 mRNA, complete cds
AT1G63550	-1.169102464	0.002322204	Arabidopsis thaliana Receptor-like protein kinase-related family protein mRNA, complete cds

AT3G44765	-1.169102464	0.002322204	Arabidopsis thaliana Full-length cDNA Complete sequence from clone GSLTPGH55ZA10 of Hormone Treated Callus of strain col-0 of Arabidopsis thaliana (thale cress)
AT5G46370	-1.28774696	0.002345281	Arabidopsis thaliana two-pore potassium channel 2 mRNA, complete cds
AT3G03830	-1.510139381	0.002463522	Arabidopsis thaliana chromosome 3, complete sequence
AT5G17810	-2.725152272	0.002560085	Arabidopsis thaliana WUSCHEL-related homeobox 12 mRNA, complete cds
AT5G15940	-2.725152272	0.002560085	Arabidopsis thaliana Rossmann-fold NAD(P)-binding domain-containing protein mRNA, complete cds
AT1G73910	-1.832067476	0.002625248	Arabidopsis thaliana actin-related protein 4A mRNA, complete cds
AT5G42600	-1.393946364	0.002694947	Arabidopsis thaliana marneral synthase mRNA, complete cds
AT1G23060	-1.129049214	0.002728383	Arabidopsis thaliana protein MICROTUBULE DESTABILIZING PROTEIN 40 mRNA, complete cds
AT3G14185	-4.194637556	0.002749466	Arabidopsis thaliana Full-length cDNA Complete sequence from clone GSLTSIL56ZC04 of Silique of strain col-0 of Arabidopsis thaliana (thale cress)
AT5G62330	-4.194637556	0.002749466	Arabidopsis thaliana uncharacterized protein mRNA, complete cds
AT1G67260	-1.304820473	0.002815014	Arabidopsis thaliana transcription factor TCP1 mRNA, complete cds
ATCG00065	-2.346640649	0.002892054	Arabidopsis thaliana ecotype XJalt rpl20-rps12 intergenic spacer, complete sequence; ribosomal protein S12 (rps12) gene, partial cds; and rps12-clpP intergenic spacer, complete sequence; chloroplast
ATCG01050	-2.112175396	0.002950304	Arabidopsis thaliana chloroplast DNA, complete genome, ecotype: Columbia
AT2G17070	-2.112175396	0.002950304	Arabidopsis thaliana chromosome 2, complete sequence
AT4G37608	-1.423261931	0.003196705	Arabidopsis thaliana uncharacterized protein mRNA, complete cds
AT1G31290	-1.423261931	0.003196705	Arabidopsis thaliana argonaute 3 mRNA, complete cds
AT3G26815	-1.247104976	0.003434455	Arabidopsis thaliana chromosome 3,

			complete sequence
AT3G51560	-3.346640649	0.003534833	Arabidopsis thaliana TIR-NBS-LRR class disease resistance protein mRNA, complete cds
AT5G44690	-3.346640649	0.003534833	Arabidopsis thaliana uncharacterized protein mRNA, complete cds
AT1G29110	-3.346640649	0.003534833	Arabidopsis thaliana cysteine proteinases superfamily protein mRNA, complete cds
AT5G46871	-3.346640649	0.003534833	Arabidopsis thaliana defensin-like protein 308 mRNA, complete cds
AT1G61130	-1.676789251	0.003789275	Arabidopsis thaliana serine carboxypeptidase-like 32 mRNA, complete cds
AT5G10880	-1.676789251	0.003789275	Arabidopsis thaliana tRNA synthetase/ligase-related protein mRNA, complete cds
AT4G14690	-1.095101882	0.003856876	Arabidopsis thaliana early light-inducible protein 2 mRNA, complete cds
AT3G06120	-1.095101882	0.003856876	Arabidopsis thaliana transcription factor MUTE mRNA, complete cds
AT2G45800	-1.261751752	0.004141629	Arabidopsis thaliana protein PLIM2A mRNA, complete cds
AT4G24860	-1.761678148	0.004265128	Arabidopsis thaliana P-loop containing nucleoside triphosphate hydrolases superfamily protein mRNA, complete cds
AT5G01335	-1.498643743	0.004442931	Arabidopsis thaliana chromosome 5 sequence
AT4G03610	-1.058659886	0.004448911	Arabidopsis thaliana metallo-beta-lactamase domain-containing protein mRNA, complete cds
AT1G35240	-2.609675055	0.004638582	Arabidopsis thaliana auxin response factor 20 mRNA, complete cds
AT5G11440	-2.609675055	0.004638582	Arabidopsis thaliana CTC-interacting domain 5 mRNA, complete cds
AT5G28262	-1.098713136	0.004690926	Arabidopsis thaliana mRNA for hypothetical protein, complete cds, clone: RAFL16-51-M03
AT2G11150	-1.872709461	0.004696361	Arabidopsis thaliana chromosome 2, complete sequence
AT2G27930	-1.2052848	0.00499465	Arabidopsis thaliana PLATZ transcription factor family protein mRNA, complete cds
AT3G07490	-2.024712554	0.005002079	Arabidopsis thaliana chromosome 3,

			complete sequence
AT5G59340	-2.024712554	0.005002079	Arabidopsis thaliana WUSCHEL-related homeobox 2 mRNA, complete cds
ATCG00650	-2.247104976	0.005049697	Arabidopsis thaliana chloroplast DNA, complete genome, ecotype: Columbia
ATCG00100	-2.247104976	0.005049697	Liverwort chloroplast Gly-tRNA-tcc
AT3G47660	-2.247104976	0.005049697	Arabidopsis thaliana Regulator of chromosome condensation (RCC1) family protein mRNA, complete cds
AT5G35760	-2.247104976	0.005049697	Arabidopsis thaliana Beta-galactosidase related protein mRNA, complete cds
AT2G26940	-4.024712554	0.005274558	Arabidopsis thaliana chromosome 2, complete sequence
AT4G10600	-1.060336464	0.005417568	Arabidopsis thaliana chromosome 4 sequence
AT5G46140	-1.403224178	0.005748722	Arabidopsis thaliana uncharacterized protein mRNA, complete cds
AT1G21530	-1.403224178	0.005748722	Arabidopsis thaliana probable acyl-activating enzyme 10 mRNA, complete cds
AT1G33540	-1.297731049	0.006009727	Arabidopsis thaliana serine carboxypeptidase-like 18 mRNA, complete cds
AT1G73066	-1.06218726	0.006602013	Arabidopsis thaliana chromosome 1 sequence
AT1G26558	-3.194637556	0.006686473	Arabidopsis thaliana Full-length cDNA Complete sequence from clone GSLTLS18ZE08 of Adult vegetative tissue of strain col-0 of Arabidopsis thaliana (thale cress)
AT1G35730	-3.194637556	0.006686473	Arabidopsis thaliana protein pumilio 9 mRNA, complete cds
AT2G44990	-3.194637556	0.006686473	Arabidopsis thaliana carotenoid cleavage dioxygenase 7 mRNA, complete cds
AT2G16005	-3.194637556	0.006686473	Arabidopsis thaliana MD-2-related lipid recognition domain-containing protein mRNA, complete cds
AT1G32172	-3.194637556	0.006686473	Arabidopsis thaliana chromosome 1 sequence
AT1G05577	-3.194637556	0.006686473	Arabidopsis thaliana uncharacterized protein mRNA, complete cds
AT5G45230	-3.194637556	0.006686473	Arabidopsis thaliana TIR-NBS-LRR class disease resistance protein mRNA, complete cds

AT2G37810	-3.194637556	0.006686473	Arabidopsis thaliana chromosome 2, complete sequence
AT3G50570	-1.439750054	0.006823792	Arabidopsis thaliana chromosome 3, complete sequence
AT1G49220	-1.687677567	0.006873189	Arabidopsis thaliana chromosome 1 sequence
AT5G28360	-1.687677567	0.006873189	Arabidopsis thaliana 1-aminocyclopropane-1-carboxylate synthase mRNA, complete cds
AT2G02061	-1.687677567	0.006873189	Arabidopsis thaliana nucleotide-diphospho-sugar transferase domain-containing protein mRNA, complete cds
AT1G22980	-1.231163432	0.007326211	Arabidopsis thaliana uncharacterized protein mRNA, complete cds
AT1G30990	-1.790247301	0.007714422	Arabidopsis thaliana ligand-binding bet-v-1 domain-containing protein mRNA, complete cds
AT1G47760	-1.790247301	0.007714422	Arabidopsis thaliana protein agamous-like 102 mRNA, complete cds
AT5G25640	-1.064240918	0.008051874	Arabidopsis thaliana Rhomboid-related intramembrane serine protease family protein mRNA, complete cds
AT5G23000	-1.064240918	0.008051874	Arabidopsis thaliana transcription factor RAX1 mRNA, complete cds
AT5G19810	-1.484144173	0.008059793	Arabidopsis thaliana chromosome 5 sequence
AT1G02470	-1.484144173	0.008059793	Arabidopsis thaliana SRPBCC ligand-binding domain-containing protein mRNA, complete cds
AT4G27360	-1.484144173	0.008059793	Arabidopsis thaliana Dynein light chain type 1 family protein mRNA, complete cds
AT5G65800	-1.484144173	0.008059793	Arabidopsis thaliana mRNA for 1-aminocyclopropane-1-carboxylate synthase ACS5, complete cds, clone: RAFL16-37-F05
AT5G61610	-2.484144173	0.008365655	Arabidopsis thaliana oleosin mRNA, complete cds
AT2G10735	-2.484144173	0.008365655	Arabidopsis thaliana chromosome 2, complete sequence
AT3G19320	-2.484144173	0.008365655	Arabidopsis thaliana leucine-rich repeat-containing protein mRNA, complete cds

AT5G55020	-2.484144173	0.008365655	Arabidopsis thaliana myb domain protein 120 mRNA, complete cds
AT3G58060	-2.484144173	0.008365655	Arabidopsis thaliana putative metal tolerance protein C3 mRNA, complete cds
AT4G12005	-1.93160315	0.008414685	Arabidopsis thaliana uncharacterized protein mRNA, complete cds
AT5G25240	-1.112175396	0.008472363	Arabidopsis thaliana chromosome 5 sequence
AT2G02300	-2.140189772	0.008756377	Arabidopsis thaliana phloem protein 2-B5 mRNA, complete cds
AT3G44705	-2.140189772	0.008756377	Arabidopsis thaliana chromosome 3, complete sequence
AT1G11735	-1.171553943	0.008769331	Arabidopsis thaliana chromosome 1 sequence
AT3G27860	-1.171553943	0.008769331	Arabidopsis thaliana chromosome 3, complete sequence
AT1G64250	-1.247104976	0.008870194	Arabidopsis thaliana chromosome 1 sequence
AT1G18000	-1.024712554	0.009228113	Arabidopsis thaliana chromosome 1 sequence
AT5G10945	-3.832067476	0.010198461	Arabidopsis thaliana chromosome 5 sequence
AT5G20260	-3.832067476	0.010198461	Arabidopsis thaliana Exostosin family protein mRNA, complete cds
AT1G73510	-3.832067476	0.010198461	Arabidopsis thaliana chromosome 1 sequence
AT5G49370	-1.378349509	0.010390974	Arabidopsis thaliana Pleckstrin homology (PH) domain-containing protein-like protein mRNA, complete cds
AT5G20860	-1.182253831	0.010668423	Arabidopsis thaliana putative pectinesterase/pectinesterase inhibitor 54 mRNA, complete cds
AT3G18485	-1.609675055	0.010978303	Arabidopsis thaliana protein IAA-LEUCINE RESISTANT 2 mRNA, complete cds
AT4G31877	-1.609675055	0.010978303	Arabidopsis thaliana mRNA for unknown protein, complete cds, clone: RAFL17-06-E12
AT1G61440	-1.609675055	0.010978303	Arabidopsis thaliana G-type lectin S-receptor-like serine/threonine-protein kinase mRNA, complete cds
AT4G27250	-1.609675055	0.010978303	Arabidopsis thaliana Rossmann-fold NAD(P)-binding domain-containing protein mRNA, complete cds

AT5G64685	-1.609675055	0.010978303	Arabidopsis thaliana chromosome 5 sequence
AT5G36270	-1.609675055	0.010978303	Arabidopsis thaliana chromosome 5 sequence
AT1G33615	-1.024712554	0.01127537	Arabidopsis thaliana chromosome 1 sequence
AT1G55700	-1.702784459	0.012559008	Arabidopsis thaliana chromosome 1 sequence
AT1G55010	-3.024712554	0.012656076	Arabidopsis thaliana plant defensin 1.5 mRNA, complete cds
AT1G04580	-3.024712554	0.012656076	Arabidopsis thaliana aldehyde oxidase 4 mRNA, complete cds
AT1G59265	-3.024712554	0.012656076	Arabidopsis thaliana chromosome 1 sequence
AT4G02960	-3.024712554	0.012656076	Arabidopsis thaliana copia-like retrotransposon AtRE2 gene for polyprotein, complete cds, ecotype: Isenburg, chromosome: 4
AT1G11990	-1.194637556	0.012983336	Arabidopsis thaliana O-fucosyltransferase family protein mRNA, complete cds
AT1G67035	-1.024712554	0.013791423	Arabidopsis thaliana uncharacterized protein mRNA, complete cds
AT3G55580	-1.024712554	0.013791423	Arabidopsis thaliana regulator of chromosome condensation repeat-containing protein mRNA, complete cds
AT1G60989	-1.832067476	0.014031935	Arabidopsis thaliana protein SCR-like 7 mRNA, complete cds
AT3G12981	-1.832067476	0.014031935	Arabidopsis lyrata subsp. lyrata hypothetical protein, mRNA
AT1G52790	-1.832067476	0.014031935	Arabidopsis thaliana oxidoreductase, 2OG-Fe(II) oxygenase family protein mRNA, complete cds
AT5G51451	-1.072018269	0.014688553	Arabidopsis thaliana root meristem growth factor 5 mRNA, complete cds
AT3G20395	-1.465285146	0.014729832	Arabidopsis thaliana RING-finger domain-containing protein mRNA, complete cds
AT5G42490	-2.346640649	0.015002012	Arabidopsis thaliana ATP binding microtubule motor family protein mRNA, complete cds
AT1G69570	-2.346640649	0.015002012	Arabidopsis thaliana Dof zinc finger protein DOF1.10 mRNA, complete cds
AT5G15725	-2.346640649	0.015002012	Arabidopsis thaliana uncharacterized

			protein mRNA, complete cds
AT3G12470	-2.346640649	0.015002012	Arabidopsis thaliana chromosome 3, complete sequence
AT1G70581	-2.346640649	0.015002012	Arabidopsis thaliana chromosome 1 sequence
AT3G12540	-1.131627758	0.015406113	Arabidopsis thaliana uncharacterized protein mRNA, complete cds
AT3G06640	-1.131627758	0.015406113	Arabidopsis thaliana PAS domain-containing tyrosine kinase-like protein mRNA, complete cds
AT1G69480	-1.314219171	0.015678687	Arabidopsis thaliana EXS (ERD1/XPR1/SYG1) family protein mRNA, complete cds
AT5G46915	-1.209137125	0.015804923	Arabidopsis thaliana putative B3 domain-containing protein mRNA, complete cds
AT1G28690	-1.024712554	0.016888697	Arabidopsis thaliana chromosome 1 sequence
AT2G22460	-1.527212895	0.017366446	Arabidopsis thaliana chromosome 2, complete sequence
AT5G23270	-1.527212895	0.017366446	Arabidopsis thaliana sugar transport protein 11 mRNA, complete cds
AT5G50260	-1.075338627	0.01798654	Arabidopsis thaliana KDEL-tailed cysteine endopeptidase CEP1 mRNA, complete cds
AT2G41905	-1.140189772	0.018837182	Arabidopsis thaliana chromosome 2, complete sequence
AT1G11112	-1.346640649	0.018901318	Arabidopsis thaliana chromosome 1 sequence
AT4G34790	-1.346640649	0.018901318	Arabidopsis thaliana chromosome 4 sequence
AT3G29772	-3.609675055	0.019888233	Arabidopsis thaliana chromosome 3, complete sequence
AT1G74110	-3.609675055	0.019888233	Arabidopsis thaliana cytochrome P450, family 78, subfamily A, polypeptide 10 mRNA, complete cds
AT4G25750	-1.609675055	0.020244491	Arabidopsis thaliana chromosome 4 sequence
AT2G30670	-1.609675055	0.020244491	Arabidopsis thaliana NAD(P)-binding Rossmann-fold superfamily protein mRNA, complete cds
AT5G43170	-1.079160338	0.022053128	Arabidopsis thaliana chromosome 5 sequence
AT2G25160	-1.079160338	0.022053128	Arabidopsis thaliana cytochrome P450,

			family 82, subfamily F, polypeptide 1 mRNA, complete cds
AT1G31720	-1.387282634	0.022719559	Arabidopsis thaliana uncharacterized protein mRNA, complete cds
AT2G29000	-1.387282634	0.022719559	Arabidopsis thaliana leucine-rich repeat protein kinase family protein mRNA, complete cds
AT4G08400	-1.387282634	0.022719559	Arabidopsis thaliana chromosome 4 sequence
AT4G11070	-1.150243436	0.023054584	Arabidopsis thaliana putative WRKY transcription factor 41 mRNA, complete cds
AT5G10890	-1.150243436	0.023054584	Arabidopsis thaliana myosin heavy chain-like protein mRNA, complete cds
AT3G62499	-1.725152272	0.023169248	Arabidopsis thaliana chromosome 3, complete sequence
AT1G26680	-1.725152272	0.023169248	Arabidopsis thaliana transcriptional factor B3 family protein mRNA, complete cds
AT1G49790	-1.725152272	0.023169248	Arabidopsis thaliana F-box associated ubiquitination effector family protein mRNA, complete cds
AT3G23450	-1.725152272	0.023169248	Arabidopsis thaliana chromosome 3, complete sequence
AT1G69880	-1.725152272	0.023169248	Arabidopsis thaliana thioredoxin H8 mRNA, complete cds
ATCG00380	-1.725152272	0.023169248	Arabidopsis thaliana chloroplast DNA, complete genome, ecotype: Columbia
AT5G61920	-1.247104976	0.023426733	Arabidopsis thaliana protein FLX-like 4 mRNA, complete cds
AT5G56795	-1.247104976	0.023426733	Arabidopsis thaliana metallothionein 1B mRNA, complete cds
AT5G56970	-1.247104976	0.023426733	Arabidopsis thaliana cytokinin dehydrogenase 3 mRNA, complete cds
AT2G15060	-2.832067476	0.023954531	Arabidopsis thaliana chromosome 2, complete sequence
AT1G06750	-2.832067476	0.023954531	Arabidopsis thaliana P-loop containing nucleoside triphosphate hydrolases superfamily protein mRNA, complete cds
AT3G48523	-2.832067476	0.023954531	Arabidopsis thaliana chromosome 3, complete sequence
AT5G02000	-2.832067476	0.023954531	Arabidopsis thaliana chromosome 5 sequence
AT4G14403	-2.832067476	0.023954531	Arabidopsis thaliana ecotype Sf-2

			At4g14390 pseudogene, complete sequence; accelerated cell death 6 (ACD6A) gene, complete cds; and ACD6B pseudogene, complete sequence
AT5G28510	-2.832067476	0.023954531	Arabidopsis thaliana beta glucosidase 24 mRNA, complete cds
AT3G47870	-2.832067476	0.023954531	Arabidopsis thaliana LOB domain-containing protein 27 mRNA, complete cds
AT5G04630	-2.832067476	0.023954531	Arabidopsis thaliana chromosome 5 sequence
AT4G31640	-2.832067476	0.023954531	Arabidopsis thaliana B3 domain-containing protein REM5 mRNA, complete cds
ATCG00710	-2.832067476	0.023954531	Arabidopsis thaliana chloroplast DNA, complete genome, ecotype: Columbia
AT4G01200	-1.024712554	0.025429253	Arabidopsis thaliana chromosome 4 sequence
AT2G03020	-1.899181672	0.025676386	Arabidopsis thaliana Heat shock protein HSP20/alpha crystallin family protein mRNA, complete cds
AT2G12450	-1.899181672	0.025676386	Arabidopsis thaliana chromosome 2, complete sequence
AT1G62240	-2.194637556	0.026716088	Arabidopsis thaliana chromosome 1 sequence
AT3G19663	-2.194637556	0.026716088	Arabidopsis thaliana chromosome 3, complete sequence
AT3G28620	-2.194637556	0.026716088	Arabidopsis thaliana chromosome 3, complete sequence
AT5G34820	-2.194637556	0.026716088	Arabidopsis thaliana chromosome 5 sequence
AT5G39820	-2.194637556	0.026716088	Arabidopsis thaliana NAC domain containing protein 94 mRNA, complete cds
AT1G19394	-1.439750054	0.027182506	Arabidopsis thaliana uncharacterized protein mRNA, complete cds
AT3G01319	-1.439750054	0.027182506	Arabidopsis thaliana chromosome 3, complete sequence
AT1G44120	-1.439750054	0.027182506	Arabidopsis thaliana armadillo/beta-catenin-like repeat and C2 calcium/lipid-binding domain-containing protein mRNA, complete cds
AT1G63630	-1.272640068	0.02850925	Arabidopsis thaliana pentatricopeptide

			(PPR) repeat-containing protein mRNA, complete cds
AT5G40320	-1.272640068	0.02850925	Arabidopsis thaliana chromosome 5 sequence
AT5G25045	-1.272640068	0.02850925	Arabidopsis thaliana chromosome 5 sequence
AT5G11180	-1.272640068	0.02850925	Arabidopsis thaliana glutamate receptor 2.6 mRNA, complete cds
AT3G28857	-1.024712554	0.031277435	Arabidopsis thaliana protein PACLOBUTRAZOL RESISTANCE 5 mRNA, complete cds
AT4G35200	-1.510139381	0.032276651	Arabidopsis thaliana chromosome 4 sequence
AT3G53365	-1.510139381	0.032276651	Arabidopsis thaliana chromosome 3, complete sequence
AT1G64310	-1.510139381	0.032276651	Arabidopsis thaliana chromosome 1 sequence
AT3G05820	-1.510139381	0.032276651	Arabidopsis thaliana alkaline/neutral invertase H mRNA, complete cds
AT1G68460	-1.510139381	0.032276651	Arabidopsis thaliana chromosome 1 sequence
AT1G23465	-1.510139381	0.032276651	Arabidopsis thaliana peptidase-S24/S26 domain-containing protein mRNA, complete cds
AT1G72720	-1.510139381	0.032276651	Arabidopsis thaliana chromosome 1 sequence
AT1G27045	-1.510139381	0.032276651	Arabidopsis thaliana homeobox-leucine zipper protein ATHB-54 mRNA, complete cds
AT1G58320	-1.510139381	0.032276651	Arabidopsis thaliana PLAC8 family protein mRNA, complete cds
AT1G15540	-1.510139381	0.032276651	Arabidopsis thaliana 2-oxoglutarate (2OG) and Fe(II)-dependent oxygenase-like protein mRNA, complete cds
AT2G12440	-1.088842892	0.033299563	Arabidopsis thaliana chromosome 2, complete sequence
AT1G13620	-1.176715648	0.034638766	Arabidopsis thaliana root meristem growth factor 2 mRNA, complete cds
AT5G54570	-1.304820473	0.03466052	Arabidopsis thaliana beta glucosidase 41 mRNA, complete cds
AT3G46420	-1.609675055	0.037832219	Arabidopsis thaliana leucine-rich repeat protein kinase-like protein mRNA, complete cds

AT2G46950	-1.609675055	0.037832219	Arabidopsis thaliana cytochrome P450, family 709, subfamily B, polypeptide 2 mRNA, complete cds
AT5G34810	-1.609675055	0.037832219	Arabidopsis thaliana chromosome 5 sequence
AT5G61120	-1.609675055	0.037832219	Arabidopsis thaliana uncharacterized protein mRNA, complete cds
AT3G62510	-1.609675055	0.037832219	Arabidopsis thaliana protein disulfide isomerase-like protein mRNA, complete cds
AT3G46170	-1.609675055	0.037832219	Arabidopsis thaliana chromosome 3, complete sequence
AT5G48090	-1.024712554	0.038542345	Arabidopsis thaliana EDM2-like protein1 mRNA, complete cds
AT1G03550	-1.024712554	0.038542345	Arabidopsis thaliana secretory carrier-associated membrane protein 2 mRNA, complete cds
AT5G65030	-1.024712554	0.038542345	Arabidopsis thaliana chromosome 5 sequence
AT4G31615	-1.024712554	0.038542345	Arabidopsis thaliana transcriptional factor B3 family protein mRNA, complete cds
AT5G66340	-3.346640649	0.039151767	Arabidopsis thaliana uncharacterized protein mRNA, complete cds
AT1G80390	-3.346640649	0.039151767	Arabidopsis thaliana auxin-responsive protein IAA15 mRNA, complete cds
AT1G06540	-3.346640649	0.039151767	Arabidopsis thaliana uncharacterized protein mRNA, complete cds
AT4G39000	-3.346640649	0.039151767	Arabidopsis thaliana glycosyl hydrolase 9B17 mRNA, complete cds
AT5G15100	-3.346640649	0.039151767	Arabidopsis thaliana auxin transporter PIN8 mRNA, complete cds
AT2G35765	-3.346640649	0.039151767	Arabidopsis thaliana uncharacterized protein mRNA, complete cds
AT4G34065	-3.346640649	0.039151767	Arabidopsis thaliana chromosome 4 sequence
AT5G03310	-3.346640649	0.039151767	Arabidopsis thaliana chromosome 5 sequence
AT1G04500	-3.346640649	0.039151767	Arabidopsis thaliana CCT motif-containing protein mRNA, complete cds
AT1G64240	-3.346640649	0.039151767	Arabidopsis thaliana chromosome 1 sequence
AT1G19610	-3.346640649	0.039151767	Arabidopsis thaliana defensin-like protein

			19 mRNA, complete cds
AT5G18220	-3.346640649	0.039151767	Arabidopsis thaliana O-Glycosyl hydrolases family 17 protein mRNA, complete cds
AT5G24355	-3.346640649	0.039151767	Arabidopsis thaliana chromosome 5 sequence
AT3G58420	-3.346640649	0.039151767	Arabidopsis thaliana TRAF-like family protein mRNA, complete cds
AT1G27820	-3.346640649	0.039151767	Arabidopsis thaliana chromosome 1 sequence
AT3G23635	-3.346640649	0.039151767	Arabidopsis thaliana chromosome 3, complete sequence
AT1G28220	-3.346640649	0.039151767	Arabidopsis thaliana purine permease 3 mRNA, complete cds
AT3G09620	-3.346640649	0.039151767	Arabidopsis thaliana DEAD-box ATP-dependent RNA helicase 45 mRNA, complete cds
AT2G30360	-3.346640649	0.039151767	Arabidopsis thaliana chromosome 2, complete sequence
AT2G46494	-3.346640649	0.039151767	Arabidopsis thaliana RING/U-box family protein mRNA, complete cds
AT3G23960	-3.346640649	0.039151767	Arabidopsis thaliana chromosome 3, complete sequence
AT5G16230	-1.346640649	0.042055579	Arabidopsis thaliana acyl-[acyl-carrier-protein] desaturase mRNA, complete cds
AT5G39240	-1.346640649	0.042055579	Arabidopsis thaliana chromosome 5 sequence
AT1G09155	-1.194637556	0.04252335	Arabidopsis thaliana phloem protein 2-B15 mRNA, complete cds
AT3G28750	-1.194637556	0.04252335	Arabidopsis thaliana chromosome 3, complete sequence
AT5G36260	-1.194637556	0.04252335	Arabidopsis thaliana aspartyl protease family protein mRNA, complete cds
AT5G60180	-1.761678148	0.043299481	Arabidopsis thaliana chromosome 5 sequence
AT4G16220	-1.761678148	0.043299481	Arabidopsis thaliana GDSL esterase/lipase mRNA, complete cds
AT1G02940	-1.761678148	0.043299481	Arabidopsis thaliana glutathione S-transferase F5 mRNA, complete cds
AT1G49700	-1.761678148	0.043299481	Arabidopsis thaliana uncharacterized protein mRNA, complete cds
AT1G57780	-2.609675055	0.045295323	Arabidopsis thaliana heavy-metal-associated

			domain-containing protein mRNA, complete cds
AT1G36640	-2.609675055	0.045295323	Arabidopsis thaliana uncharacterized protein mRNA, complete cds
AT5G58830	-2.609675055	0.045295323	Arabidopsis thaliana Subtilisin-like serine endopeptidase family protein mRNA, complete cds
AT1G07985	-2.609675055	0.045295323	Arabidopsis thaliana chromosome 1 sequence
AT1G11490	-2.609675055	0.045295323	Arabidopsis thaliana C2H2 type zinc finger protein mRNA, complete cds
ATCG01040	-2.609675055	0.045295323	Arabidopsis thaliana chloroplast DNA, complete genome, ecotype: Columbia
AT1G22600	-2.609675055	0.045295323	Arabidopsis thaliana Late embryogenesis abundant protein (LEA) family protein mRNA, complete cds
AT3G17760	-2.609675055	0.045295323	Arabidopsis thaliana glutamate decarboxylase 5 mRNA, complete cds
AT5G28715	-2.609675055	0.045295323	Arabidopsis thaliana chromosome 5 sequence
AT3G14517	-2.609675055	0.045295323	Arabidopsis thaliana chromosome 3, complete sequence
AT2G45580	-2.024712554	0.047169216	Arabidopsis thaliana cytochrome P450 76C3 mRNA, complete cds
AT1G48320	-2.024712554	0.047169216	Arabidopsis thaliana 1,4-dihydroxy-2-naphthoyl-CoA thioesterase 1 mRNA, complete cds
ATCG00030	-2.024712554	0.047169216	Cyanidiaceae sp. MX-AZ01 chloroplast, complete genome
AT3G07600	-2.024712554	0.047169216	Arabidopsis thaliana heavy-metal-associated domain-containing protein mRNA, complete cds
AT3G48010	-2.024712554	0.047169216	Arabidopsis thaliana cyclic nucleotide-gated channel 16 mRNA, complete cds
AT4G16160	-2.024712554	0.047169216	Arabidopsis thaliana outer envelope pore protein 16-2 mRNA, complete cds
AT1G23160	-1.024712554	0.047596347	Arabidopsis thaliana auxin-responsive GH3 family protein mRNA, complete cds
AT2G26220	-1.024712554	0.047596347	Arabidopsis thaliana chromosome 2, complete sequence
AT1G02630	-1.102715066	0.050644622	Arabidopsis thaliana equilibrative nucleotide transporter 8 mRNA,

			complete cds
AT1G55410	-1.102715066	0.050644622	Arabidopsis thaliana chromosome 1 sequence
AT1G58889	-1.102715066	0.050644622	Arabidopsis thaliana DNA, retrotransposon:AtRE1, complete sequence, ecotype: Niederzenz
AT1G55980	-1.102715066	0.050644622	Arabidopsis thaliana FAD/NAD(P)-binding oxidoreductase domain-containing protein mRNA, complete cds
AT4G04900	-1.403224178	0.050834675	Arabidopsis thaliana ROP-interactive CRIB motif-containing protein 10 mRNA, complete cds
AT5G52471	-1.403224178	0.050834675	Arabidopsis thaliana chromosome 5 sequence
AT1G78172	-1.403224178	0.050834675	Arabidopsis thaliana uncharacterized protein mRNA, complete cds
AT4G01060	-1.403224178	0.050834675	Arabidopsis thaliana CAPRICE-like MYB3 mRNA, complete cds
AT5G12280	-1.217357632	0.052249746	Arabidopsis thaliana SWAP (Suppressor-of-White-APricot)/surp RNA-binding domain-containing protein mRNA, complete cds
AT1G30280	-1.217357632	0.052249746	Arabidopsis thaliana chaperone DnaJ-domain-containing protein mRNA, complete cds
AT5G07570	-1.217357632	0.052249746	Arabidopsis thaliana glycine/proline-rich protein mRNA, complete cds
ATCG00360	-1.024712554	0.058923168	Arabidopsis lyrata subsp. lyrata predicted protein, mRNA
AT4G01335	-1.484144173	0.060996939	Arabidopsis thaliana uncharacterized protein mRNA, complete cds
AT1G58130	-1.484144173	0.060996939	Arabidopsis thaliana chromosome 1 sequence
AT4G08109	-1.484144173	0.060996939	Arabidopsis thaliana chromosome 4 sequence
AT5G14820	-1.484144173	0.060996939	Arabidopsis thaliana chromosome 5 sequence
AT5G55780	-1.112175396	0.062665394	Arabidopsis thaliana chromosome 5 sequence
AT3G27490	-1.112175396	0.062665394	Arabidopsis thaliana chromosome 3, complete sequence
AT4G37950	-1.112175396	0.062665394	Arabidopsis thaliana Rhamnogalacturonate lyase family protein mRNA, complete cds

AT4G38210	-1.112175396	0.062665394	Arabidopsis thaliana expansin A20 mRNA, complete cds
AT4G17160	-1.112175396	0.062665394	Arabidopsis thaliana RAB GTPase homolog B1A mRNA, complete cds
AT2G20835	-1.112175396	0.062665394	Arabidopsis thaliana chromosome 2, complete sequence
AT1G05061	-1.112175396	0.062665394	Arabidopsis thaliana chromosome 1 sequence
AT2G24030	-1.112175396	0.062665394	Arabidopsis thaliana zinc ion binding / nucleic acid binding protein mRNA, complete cds
AT3G14640	-1.112175396	0.062665394	Arabidopsis thaliana cytochrome P450, family 72, subfamily A, polypeptide 10 mRNA, complete cds
AT3G57740	-1.112175396	0.062665394	Arabidopsis thaliana chromosome 3, complete sequence
AT1G24600	-1.247104976	0.064240102	Arabidopsis thaliana chromosome 1 sequence
AT3G44800	-1.247104976	0.064240102	Arabidopsis thaliana Meprin and TRAF (MATH) homology domain-containing protein mRNA, complete cds
AT3G17600	-1.247104976	0.064240102	Arabidopsis thaliana auxin-responsive protein IAA31 mRNA, complete cds
AT5G54050	-1.247104976	0.064240102	Arabidopsis thaliana cysteine/histidine-rich C1 domain-containing protein mRNA, complete cds
AT4G22630	-1.247104976	0.064240102	Arabidopsis thaliana bifunctional inhibitor/lipid-transfer protein/seed storage 2S albumin superfamily protein mRNA, complete cds
AT1G48820	-1.609675055	0.07210619	Arabidopsis thaliana terpene synthase/cyclase family protein mRNA, complete cds
AT1G51260	-1.609675055	0.07210619	Arabidopsis thaliana 1-acyl-sn-glycerol-3-phosphate acyltransferase 3 mRNA, complete cds
AT2G24870	-1.609675055	0.07210619	Arabidopsis thaliana chromosome 2, complete sequence
AT1G73600	-1.609675055	0.07210619	Arabidopsis thaliana putative phosphoethanolamine N-methyltransferase 3 mRNA, complete cds
AT4G09432	-1.609675055	0.07210619	Arabidopsis thaliana chromosome 4

			sequence
AT1G08080	-1.609675055	0.07210619	Arabidopsis thaliana alpha carbonic anhydrase 7 mRNA, complete cds
AT5G21080	-1.609675055	0.07210619	Arabidopsis thaliana uncharacterized protein mRNA, complete cds
AT1G76190	-1.609675055	0.07210619	Arabidopsis thaliana chromosome 1 sequence
AT2G16580	-1.024712554	0.073158466	Arabidopsis thaliana SAUR-like auxin-responsive protein mRNA, complete cds
ATCG00450	-1.024712554	0.073158466	Spinach chloroplast Val-tRNA
AT4G34510	-1.024712554	0.073158466	Arabidopsis thaliana chromosome 4 sequence
AT1G04150	-1.024712554	0.073158466	Arabidopsis thaliana chromosome 1 sequence

Supplementary Data S9 Up regulated genes in *fc1*/Col-o with Cd treatment.

Gene	Log2FoldChange	pval	NT:Description
AT1G74310	1.043972676	7.41E-97	Arabidopsis thaliana chaperone protein ClpB1 mRNA, complete cds
AT3G41979	2.770002879	1.92E-60	Erysimum belvederense internal transcribed spacer 1, partial sequence; 5.8S ribosomal RNA gene, complete sequence; and internal transcribed spacer 2, partial sequence
AT1G74670	1.004320486	1.52E-55	Arabidopsis thaliana gibberellin-regulated protein 6 mRNA, complete cds
AT2G39030	1.199730003	6.33E-49	Arabidopsis thaliana chromosome 2, complete sequence
AT4G08620	1.059637061	3.60E-47	Arabidopsis thaliana sulfate transporter 1.1 mRNA, complete cds
AT4G24570	1.008883423	2.33E-46	Arabidopsis thaliana chromosome 4 sequence
AT1G16030	1.436705748	4.23E-40	Arabidopsis thaliana chromosome 1 sequence
AT5G37940	2.33688216	9.59E-36	Arabidopsis thaliana zinc-binding dehydrogenase family protein mRNA, complete cds
AT1G54050	1.083006504	3.36E-30	Arabidopsis thaliana CIII heat shock protein 17.4 mRNA, complete cds
AT3G44300	1.333057337	2.52E-28	Arabidopsis thaliana nitrilase 2 mRNA, complete cds
AT1G59860	1.535117262	3.19E-22	Arabidopsis thaliana chromosome 1 sequence
AT1G75940	7.414604455	7.95E-21	Arabidopsis thaliana beta-glucosidase 20 mRNA, complete cds
AT4G33610	1.230590967	1.31E-19	Arabidopsis thaliana chromosome 4 sequence
AT5G12030	1.069468969	2.82E-18	Arabidopsis thaliana chromosome 5 sequence
AT4G07820	1.344215127	8.78E-18	Arabidopsis thaliana chromosome 4 sequence
AT1G18970	1.145951153	9.26E-17	Arabidopsis thaliana chromosome 1 sequence
AT5G26130	1.3721907	2.37E-16	Arabidopsis thaliana chromosome 5 sequence
AT1G20130	5.813153832	3.74E-15	Arabidopsis thaliana anther-specific proline-rich protein APG mRNA, complete cds

AT5G12020	1.291897933	3.77E-15	Arabidopsis thaliana chromosome 5 sequence
AT3G22400	1.196180936	2.44E-14	Arabidopsis thaliana lipoxygenase 5 mRNA, complete cds
AT1G57750	2.206711604	2.44E-13	Arabidopsis thaliana chromosome 1 sequence
AT4G30670	1.085876528	3.09E-12	Arabidopsis thaliana chromosome 4 sequence
AT1G67990	6.414604455	9.80E-12	Arabidopsis thaliana tapetum-specific methyltransferase 1 mRNA, complete cds
AT1G70880	1.078321067	1.10E-10	Arabidopsis thaliana SRPBCC domain-containing protein mRNA, complete cds
AT3G51590	6.240575055	1.29E-10	Arabidopsis thaliana non-specific lipid-transfer protein 12 mRNA, complete cds
AT4G35770	1.277100931	1.61E-10	Arabidopsis thaliana senescence-associated protein DIN1 mRNA, complete cds
AT5G54190	1.989120168	2.07E-10	Arabidopsis thaliana protochlorophyllide reductase A mRNA, complete cds
AT4G17280	1.144515292	4.30E-10	Arabidopsis thaliana putative auxin-responsive protein mRNA, complete cds
AT3G25190	1.218207242	5.03E-10	Arabidopsis thaliana vacuolar iron transporter homolog 2.1 mRNA, complete cds
AT4G28395	6.125097838	6.25E-10	Arabidopsis thaliana lipid transfer protein-related mRNA, complete cds
AT4G32280	1.481718651	8.67E-10	Arabidopsis thaliana auxin-responsive protein IAA29 mRNA, complete cds
AT1G68875	6.042635678	1.81E-09	Arabidopsis thaliana chromosome 1 sequence
AT5G66400	1.316170496	5.46E-09	Arabidopsis thaliana dehydrin Rab18 mRNA, complete cds
AT2G03200	1.353722213	2.04E-08	Arabidopsis thaliana chromosome 2, complete sequence
AT4G28850	1.440599664	6.52E-08	Arabidopsis thaliana xyloglucan endotransglucosylase/hydrolase 26 mRNA, complete cds
AT1G63560	1.698397421	6.65E-08	Arabidopsis thaliana chromosome 1 sequence
AT2G40370	1.048282241	7.54E-08	Arabidopsis thaliana laccase 5 mRNA, complete cds

AT3G28500	1.296209754	7.57E-08	Arabidopsis thaliana chromosome 3, complete sequence
AT2G44578	1.81576978	8.53E-08	Arabidopsis thaliana chromosome 2, complete sequence
AT3G11980	5.655612555	1.41E-07	Arabidopsis thaliana fatty acyl-CoA reductase 2 mRNA, complete cds
AT3G25180	1.636996877	1.43E-07	Arabidopsis thaliana cytochrome P450, family 82, subfamily G, polypeptide 1 mRNA, complete cds
AT3G20340	2.247954586	2.17E-07	Arabidopsis thaliana chromosome 3, complete sequence
AT1G05575	1.807615648	2.20E-07	Arabidopsis thaliana chromosome 1 sequence
AT5G09520	1.210827712	2.41E-07	Arabidopsis thaliana chromosome 5 sequence
AT4G02485	1.558513867	2.71E-07	Arabidopsis thaliana oxidoreductase, 2OG-Fe(II) oxygenase family protein mRNA, complete cds
AT1G02340	1.270375068	2.92E-07	Arabidopsis thaliana transcription factor HFR1 mRNA, complete cds
AT5G07550	5.478734793	7.60E-07	Arabidopsis thaliana glycine-rich protein 19 mRNA, complete cds
AT5G16920	5.478734793	7.60E-07	Arabidopsis thaliana chromosome 5 sequence
AT2G34440	2.909369147	1.95E-06	Arabidopsis thaliana chromosome 2, complete sequence
AT2G02990	1.40097259	2.03E-06	Arabidopsis thaliana ribonuclease 1 mRNA, complete cds
AT3G42658	3.177565258	2.41E-06	Arabidopsis thaliana chromosome 3, complete sequence
AT5G43370	1.121822706	2.46E-06	Arabidopsis thaliana phosphate transporter Pht1;2 mRNA, complete cds
AT5G18030	1.218207242	2.60E-06	Arabidopsis thaliana chromosome 5 sequence
AT4G25780	1.108647317	3.39E-06	Arabidopsis thaliana chromosome 4 sequence
AT5G07530	5.277100931	4.23E-06	Arabidopsis thaliana glycine rich protein 17 mRNA, complete cds
AT5G37970	5.277100931	4.23E-06	Arabidopsis thaliana probable S-adenosylmethionine-dependent methyltransferase mRNA, complete cds
AT5G16960	2.317742916	6.00E-06	Arabidopsis thaliana zinc-binding dehydrogenase family protein mRNA, complete cds

AT3G57510	2.813153832	6.03E-06	Arabidopsis thaliana polygalacturonase ADPG1 mRNA, complete cds
AT3G60490	1.049148985	7.38E-06	Arabidopsis thaliana chromosome 3, complete sequence
AT5G25450	1.068383447	8.76E-06	Arabidopsis thaliana cytochrome bd ubiquinol oxidase mRNA, complete cds
AT5G08250	2.277100931	1.01E-05	Arabidopsis thaliana cytochrome P450 superfamily protein mRNA, complete cds
AT2G25090	1.922005973	1.12E-05	Arabidopsis thaliana SNF1-related kinase mRNA, complete cds
AT1G52830	1.990796746	1.13E-05	Arabidopsis thaliana indole-3-acetic acid 6 mRNA, complete cds
AT5G19410	1.306645207	1.31E-05	Arabidopsis thaliana chromosome 5 sequence
AT2G34000	1.655612555	1.31E-05	Arabidopsis thaliana chromosome 2, complete sequence
AT2G03740	5.125097838	1.35E-05	Arabidopsis thaliana late embryogenesis abundant domain-containing protein mRNA, complete cds
AT1G75910	5.125097838	1.35E-05	Arabidopsis thaliana extracellular lipase 4 mRNA, complete cds
AT1G62940	2.125097838	1.78E-05	Arabidopsis thaliana acyl-CoA synthetase 5 mRNA, complete cds
AT1G63570	1.155110407	2.24E-05	Arabidopsis thaliana chromosome 1 sequence
AT2G15220	1.155110407	2.24E-05	Arabidopsis thaliana chromosome 2, complete sequence
AT4G11655	2.084455853	2.92E-05	Arabidopsis thaliana uncharacterized protein mRNA, complete cds
AT2G43050	1.466134756	2.97E-05	Arabidopsis thaliana putative pectinesterase/ pectinerase inhibitor 16 mRNA, complete cds
AT1G17147	1.385807191	2.99E-05	Arabidopsis thaliana chromosome 1 sequence
AT5G38096	4.125097838	3.12E-05	Arabidopsis thaliana chromosome 5 sequence
AT4G34850	4.125097838	3.12E-05	Arabidopsis thaliana protein LESS ADHESIVE POLLEN 5 mRNA, complete cds
AT4G25200	1.089473928	3.15E-05	Arabidopsis thaliana small heat shock protein 23.6 mRNA, complete cds
AT3G27270	1.031523723	3.59E-05	Arabidopsis thaliana chromosome 3, complete sequence
AT5G39150	2.440599664	3.87E-05	Arabidopsis thaliana germin-like protein subfamily 1 member 17 mRNA, complete

			cds
AT2G19070	4.955172837	4.41E-05	Arabidopsis thaliana spermidine hydroxycinnamoyl transferase mRNA, complete cds
AT2G44220	3.277100931	5.35E-05	Arabidopsis thaliana uncharacterized protein mRNA, complete cds
AT2G46860	1.639671011	5.66E-05	Arabidopsis thaliana pyrophosphorylase 3 mRNA, complete cds
AT1G73290	2.388132244	6.61E-05	Arabidopsis thaliana serine carboxypeptidase-like 5 mRNA, complete cds
AT5G19700	1.19802936	6.63E-05	Arabidopsis thaliana chromosome 5 sequence
AT5G26270	1.571844197	7.86E-05	Arabidopsis thaliana uncharacterized protein mRNA, complete cds
AT4G12360	1.457673177	8.39E-05	Arabidopsis thaliana Bifunctional inhibitor/lipid-transfer protein/seed storage 2S albumin superfamily protein mRNA, complete cds
AT5G60730	1.177565258	9.33E-05	Arabidopsis thaliana Anion-transporting ATPase mRNA, complete cds
AT5G26730	1.647050541	9.52E-05	Arabidopsis thaliana Fasciclin-like arabinogalactan family protein mRNA, complete cds
AT4G18170	1.144997395	9.55E-05	Arabidopsis thaliana WRKY DNA-binding protein 28 mRNA, complete cds
AT3G51360	1.064797328	9.64E-05	Arabidopsis thaliana aspartyl protease family protein mRNA, complete cds
AT1G74930	1.184654683	0.000109055	Arabidopsis thaliana chromosome 1 sequence
AT4G14548	1.277100931	0.00011519	Arabidopsis thaliana chromosome 4 sequence
AT1G75030	1.025562164	0.000129594	Arabidopsis thaliana thaumatin-like protein 3 mRNA, complete cds
AT5G13380	4.762527759	0.000146416	Arabidopsis thaliana auxin-responsive GH3 family protein mRNA, complete cds
AT4G37240	1.073817333	0.000156848	Arabidopsis thaliana chromosome 4 sequence
AT1G77960	1.379670665	0.000161259	Arabidopsis thaliana uncharacterized protein mRNA, complete cds
AT3G21500	3.125097838	0.00017535	Arabidopsis thaliana 1-deoxy-D-xylulose 5-phosphate synthase 1 mRNA, complete cds
AT5G65207	1.052034376	0.0001828	Arabidopsis thaliana chromosome 5

			sequence
AT4G06536	2.277100931	0.000189933	Arabidopsis thaliana chromosome 4 sequence
AT5G58910	1.909369147	0.000204024	Arabidopsis thaliana laccase 16 mRNA, complete cds
AT5G46040	1.277100931	0.000222519	Arabidopsis thaliana peptide transporter PTR3-B mRNA, complete cds
AT4G01925	1.447025933	0.000238725	Arabidopsis thaliana cysteine/histidine-rich C1 domain-containing protein mRNA, complete cds
AT3G58850	1.033175349	0.00024966	Arabidopsis thaliana chromosome 3, complete sequence
AT5G48210	4.655612555	0.000268753	Arabidopsis thaliana chromosome 5 sequence
AT2G30942	1.505369919	0.00030423	Arabidopsis thaliana uncharacterized protein mRNA, complete cds
AT5G47635	1.785247835	0.00031237	Arabidopsis thaliana pollen Ole e 1 allergen and extensin family protein mRNA, complete cds
AT1G33910	2.218207242	0.000319928	Arabidopsis thaliana P-loop containing nucleoside triphosphate hydrolases superfamily protein mRNA, complete cds
AT5G11290	1.955172837	0.000336047	Arabidopsis thaliana chromosome 5 sequence
AT4G14650	1.067065717	0.00041412	Arabidopsis thaliana uncharacterized protein mRNA, complete cds
AT4G22505	1.097191841	0.000415692	Arabidopsis thaliana chromosome 4 sequence
AT5G40460	1.223661672	0.000456813	Arabidopsis thaliana chromosome 5 sequence
AT4G04293	1.677638861	0.000456941	Arabidopsis thaliana chromosome 4 sequence
AT2G21350	1.677638861	0.000456941	Arabidopsis thaliana RNA-binding CRS1 / YhbY (CRM) domain protein mRNA, complete cds
AT1G49960	1.017457115	0.000472767	Arabidopsis thaliana nucleobase-ascorbate transporter 4 mRNA, complete cds
AT1G56360	4.540135337	0.000495979	Arabidopsis thaliana purple acid phosphatase 6 mRNA, complete cds
AT5G43360	1.813153832	0.000523344	Arabidopsis thaliana putative inorganic phosphate transporter 1-3 mRNA, complete cds

AT1G15825	2.156806698	0.000536249	Arabidopsis thaliana chromosome 1 sequence
AT1G35330	1.540135337	0.000578176	Arabidopsis thaliana RING-H2 finger protein ATL34 mRNA, complete cds
AT1G69500	3.655612555	0.000668019	Arabidopsis thaliana cytochrome P450, family 704, subfamily B, polypeptide 1 mRNA, complete cds
AT5G18050	1.433220133	0.000684167	Arabidopsis thaliana chromosome 5 sequence
AT1G50270	1.051388152	0.000791319	Arabidopsis thaliana chromosome 1 sequence
AT2G34315	1.848257633	0.00087631	Arabidopsis thaliana avirulence induced family protein mRNA, complete cds
AT3G04854	1.848257633	0.00087631	Arabidopsis thaliana chromosome 3, complete sequence
AT3G13220	4.414604455	0.000920552	Arabidopsis thaliana ABC transporter G family member 26 mRNA, complete cds
AT4G22214	4.414604455	0.000920552	Arabidopsis thaliana defensin-like protein 99 mRNA, complete cds
AT3G18460	4.414604455	0.000920552	Arabidopsis thaliana PLAC8 family protein mRNA, complete cds
AT2G15400	1.395745428	0.001017478	Arabidopsis thaliana mRNA for DNA-directed RNA polymerase II, third largest subunit, complete cds, clone: RAFL14-92-F19
AT1G70110	2.862063432	0.001026386	Arabidopsis thaliana putative L-type lectin-domain containing receptor kinase V.1 mRNA, complete cds
AT1G80555	3.540135337	0.001239533	Arabidopsis thaliana Isocitrate/isopropylmalate dehydrogenase family protein mRNA, complete cds
AT2G28680	3.540135337	0.001239533	Arabidopsis thaliana RmlC-like cupins superfamily protein mRNA, complete cds
AT1G71160	3.540135337	0.001239533	Arabidopsis thaliana chromosome 1 sequence
AT3G13020	1.260027418	0.001410085	Arabidopsis thaliana hAT transposon superfamily protein mRNA, complete cds
AT4G37235	2.20310035	0.001430564	Arabidopsis thaliana CASP-like protein mRNA, complete cds
AT3G43890	1.004082437	0.001479579	Arabidopsis thaliana chromosome 3, complete sequence
AT5G67620	1.199098419	0.001481841	Arabidopsis thaliana mRNA for hypothetical protein, complete cds,

			clone: RAFL14-69-L22
AT3G52160	2.025562164	0.001481981	Arabidopsis thaliana 3-ketoacyl-CoA synthase 15 mRNA, complete cds
AT1G52690	2.025562164	0.001481981	Arabidopsis thaliana Late embryogenesis abundant protein (LEA) family protein mRNA, complete cds
AT3G14510	2.025562164	0.001481981	Arabidopsis thaliana putative geranylgeranyl pyrophosphate synthase 8 mRNA, complete cds
AT1G56630	1.495741218	0.001495146	Arabidopsis thaliana alpha/beta-Hydrolases superfamily protein mRNA, complete cds
AT4G14080	1.35727128	0.001504325	Arabidopsis thaliana putative glucan endo-1,3-beta-glucosidase A6 mRNA, complete cds
AT5G62080	1.540135337	0.001679227	Arabidopsis thaliana protease inhibitor/seed storage/lipid transfer protein (LTP) family protein mRNA, complete cds
AT2G34330	4.277100931	0.001718915	Arabidopsis thaliana chromosome 2, complete sequence
AT4G14815	4.277100931	0.001718915	Arabidopsis thaliana bifunctional inhibitor/lipid-transfer protein/seed storage 2S albumin-like protein mRNA, complete cds
AT1G47980	4.277100931	0.001718915	Arabidopsis thaliana uncharacterized protein mRNA, complete cds
AT1G23940	1.00579891	0.001750183	Arabidopsis thaliana ARM repeat superfamily protein mRNA, complete cds
AT1G20150	2.762527759	0.001840574	Arabidopsis thaliana subtilisin-like serine endopeptidase family protein mRNA, complete cds
ATCG00430	1.655612555	0.002057188	Arabidopsis thaliana chloroplast DNA, complete genome, ecotype: Columbia
AT1G18830	1.732780415	0.002229755	Arabidopsis thaliana transport protein SEC31-like protein SEC31B mRNA, complete cds
AT1G30135	1.732780415	0.002229755	Arabidopsis thaliana protein TIFY 5A mRNA, complete cds
AT5G05220	3.414604455	0.002303144	Arabidopsis thaliana chromosome 5 sequence
AT4G38495	3.414604455	0.002303144	Arabidopsis thaliana uncharacterized protein mRNA, complete cds
AT5G41780	3.414604455	0.002303144	Arabidopsis thaliana myosin heavy

			chain-like protein mRNA, complete cds
ATCG01010	3.414604455	0.002303144	Arabidopsis thaliana chloroplast DNA, complete genome, ecotype: Columbia
AT5G55420	1.829641954	0.002367275	Arabidopsis thaliana chromosome 5 sequence
AT2G39500	1.009620621	0.002451498	Arabidopsis thaliana uncharacterized protein mRNA, complete cds
AT1G68640	1.080703719	0.002530485	Arabidopsis thaliana transcription factor PERIANTHIA mRNA, complete cds
AT3G44326	1.257735607	0.002769184	Arabidopsis thaliana chromosome 3, complete sequence
AT5G43690	1.370210336	0.002948249	Arabidopsis thaliana chromosome 5 sequence
AT5G47530	1.370210336	0.002948249	Arabidopsis thaliana putative auxin-responsive protein mRNA, complete cds
AT5G53820	1.370210336	0.002948249	Arabidopsis thaliana Late embryogenesis abundant protein (LEA) family protein mRNA, complete cds
AT1G01280	4.125097838	0.003230341	Arabidopsis thaliana cytochrome P450, family 703, subfamily A, polypeptide 2 mRNA, complete cds
AT5G62320	4.125097838	0.003230341	Arabidopsis thaliana myb domain protein 99 mRNA, complete cds
AT2G45650	4.125097838	0.003230341	Arabidopsis thaliana agamous-like MADS-box protein AGL6 mRNA, complete cds
AT1G44224	4.125097838	0.003230341	Arabidopsis thaliana chromosome 1 sequence
AT5G07560	4.125097838	0.003230341	Arabidopsis thaliana glycine-rich protein 20 mRNA, complete cds
AT1G46768	1.014066526	0.003439118	Arabidopsis thaliana chromosome 1 sequence
AT5G50760	1.05033007	0.003507098	Arabidopsis thaliana chromosome 5 sequence
AT5G23780	1.762527759	0.003798283	Arabidopsis thaliana uncharacterized protein mRNA, complete cds
AT2G04680	2.042635678	0.004038828	Arabidopsis thaliana chromosome 2, complete sequence
AT3G46668	2.042635678	0.004038828	Arabidopsis thaliana mRNA, clone: RAFL21-22-L12
AT2G24400	1.153112214	0.004130077	Arabidopsis thaliana chromosome 2, complete sequence
AT3G06220	1.153112214	0.004130077	Arabidopsis thaliana AP2/B3-like

			transcriptional factor family protein mRNA, complete cds
AT5G01110	1.05470851	0.004152495	Arabidopsis thaliana chromosome 5 sequence
AT1G66470	1.099562746	0.004175898	Arabidopsis thaliana protein ROOT HAIR DEFECTIVE6 mRNA, complete cds
AT4G09300	3.277100931	0.004284083	Arabidopsis thaliana LisH and RanBPM domain-containing protein mRNA, complete cds
AT1G13230	3.277100931	0.004284083	Arabidopsis thaliana leucine-rich repeat protein pii-2 mRNA, complete cds
AT5G61650	3.277100931	0.004284083	Arabidopsis thaliana CYCLIN P4;2 mRNA, complete cds
AT5G52400	3.277100931	0.004284083	Arabidopsis thaliana cytochrome P450, family 715, subfamily A, polypeptide 1 mRNA, complete cds
AT5G39490	3.277100931	0.004284083	Arabidopsis thaliana chromosome 5 sequence
AT1G33817	1.485687553	0.004396922	Arabidopsis thaliana chromosome 1 sequence
AT3G48790	1.485687553	0.004396922	Arabidopsis thaliana Pyridoxal phosphate (PLP)-dependent transferases superfamily protein mRNA, complete cds
AT3G59480	1.235280756	0.004681599	Arabidopsis thaliana probable fructokinase-4 mRNA, complete cds
AT1G75700	1.019303174	0.004832902	Arabidopsis thaliana HVA22-like protein G mRNA, complete cds
AT4G17483	1.353722213	0.005043637	Arabidopsis thaliana putative palmitoyl-(protein) hydrolase mRNA, complete cds
AT5G24220	1.353722213	0.005043637	Arabidopsis thaliana lipase class 3-related protein mRNA, complete cds
AT3G51642	1.607249533	0.005516121	Arabidopsis thaliana chromosome 3, complete sequence
AT1G10680	1.607249533	0.005516121	Arabidopsis thaliana ABC transporter B family member 10 mRNA, complete cds
AT1G22260	1.177565258	0.005729405	Arabidopsis thaliana synaptonemal complex protein ZYP1a mRNA, complete cds
AT1G52110	2.540135337	0.005855025	Arabidopsis thaliana jacalin-like lectin domain-containing protein mRNA, complete cds
AT3G21805	1.692138431	0.006048097	Arabidopsis thaliana chromosome 3, complete sequence

AT4G28815	3.955172837	0.006112539	Arabidopsis thaliana transcription factor bHLH127 mRNA, complete cds
AT2G42560	3.955172837	0.006112539	Arabidopsis thaliana late embryogenesis abundant domain-containing protein mRNA, complete cds
AT1G66850	3.955172837	0.006112539	Arabidopsis thaliana chromosome 1 sequence
AT5G49320	1.277100931	0.006397063	Arabidopsis thaliana uncharacterized protein mRNA, complete cds
AT3G06490	1.277100931	0.006397063	Arabidopsis thaliana putative transcription factor MYB108 mRNA, complete cds
AT3G59440	1.803169743	0.006483164	Arabidopsis thaliana chromosome 3, complete sequence
AT5G15290	2.177565258	0.006583848	Arabidopsis thaliana casparian strip membrane protein 5 mRNA, complete cds
AT3G11680	2.177565258	0.006583848	Arabidopsis thaliana Aluminum activated malate transporter family protein mRNA, complete cds
AT2G26695	2.177565258	0.006583848	Arabidopsis thaliana Ran BP2/NZF zinc finger-like protein mRNA, complete cds
AT4G30320	1.192212034	0.006743309	Arabidopsis thaliana chromosome 4 sequence
AT5G37950	1.025562164	0.006804652	Arabidopsis thaliana UDP-glycosyltransferase-like protein mRNA, complete cds
AT2G20080	1.30309614	0.007455465	Arabidopsis thaliana uncharacterized protein mRNA, complete cds
AT4G10250	1.30309614	0.007455465	Arabidopsis thaliana chromosome 4 sequence
AT1G05420	1.478734793	0.007596192	Arabidopsis thaliana chromosome 1 sequence
AT3G18530	1.478734793	0.007596192	Arabidopsis thaliana ARM repeat family protein-like protein mRNA, complete cds
AT1G68030	1.208929429	0.00793089	Arabidopsis thaliana RING/FYVE/PHD zinc finger protein mRNA, complete cds
AT1G55365	3.125097838	0.007974564	Arabidopsis thaliana chromosome 1 sequence
AT5G21130	3.125097838	0.007974564	Arabidopsis thaliana chromosome 5 sequence
AT2G35290	3.125097838	0.007974564	Arabidopsis thaliana chromosome 2, complete sequence
AT5G02330	3.125097838	0.007974564	Arabidopsis thaliana chromosome 5

			sequence
AT2G01175	3.125097838	0.007974564	Arabidopsis thaliana chromosome 2, complete sequence
AT3G21660	3.125097838	0.007974564	Arabidopsis thaliana UBX domain-containing protein mRNA, complete cds
AT1G09380	3.125097838	0.007974564	Arabidopsis thaliana nodulin MtN21-like transporter family protein mRNA, complete cds
AT1G32583	1.540135337	0.008573628	Arabidopsis thaliana uncharacterized protein mRNA, complete cds
AT5G28415	1.540135337	0.008573628	Arabidopsis thaliana chromosome 5 sequence
AT1G10000	1.618137849	0.009551323	Arabidopsis thaliana chromosome 1 sequence
AT3G60170	2.414604455	0.010370186	Arabidopsis thaliana chromosome 3, complete sequence
AT4G21780	1.25062872	0.010933891	Arabidopsis thaliana chromosome 4 sequence
AT5G21910	1.862063432	0.011078695	Arabidopsis thaliana uncharacterized protein mRNA, complete cds
AT2G42860	1.862063432	0.011078695	Arabidopsis thaliana uncharacterized protein mRNA, complete cds
AT2G35950	1.862063432	0.011078695	Arabidopsis thaliana protein EMBRYO SAC DEVELOPMENT ARREST 12 mRNA, complete cds
ATCG01100	2.070650054	0.01120139	Arabidopsis lyrata subsp. lyrata NADH-plastoquinone oxidoreductase subunit 1, mRNA
AT5G52720	1.037634997	0.011415917	Arabidopsis thaliana copper transport family protein mRNA, complete cds
AT3G27510	1.037634997	0.011415917	Arabidopsis thaliana chromosome 3, complete sequence
AT4G29250	3.762527759	0.011651963	Arabidopsis thaliana HXXXD-type acyl-transferase-like protein mRNA, complete cds
AT2G07785	3.762527759	0.011651963	Arabidopsis thaliana NADH dehydrogenase I subunit 1 mRNA, complete cds
AT1G27040	3.762527759	0.011651963	Arabidopsis thaliana probable peptide/nitrate transporter mRNA, complete cds
AT5G60022	3.762527759	0.011651963	Arabidopsis thaliana Full-length cDNA Complete sequence from clone

			GSLTLS5ZA07 of Adult vegetative tissue of strain col-0 of Arabidopsis thaliana (thale cress)
AT2G07719	1.469746009	0.013210035	Arabidopsis thaliana ecotype Landsberg erecta mitochondrion, complete genome
AT4G37050	1.177565258	0.013466294	Arabidopsis thaliana PATATIN-like protein 4 mRNA, complete cds
AT5G24200	1.042635678	0.013581819	Arabidopsis thaliana uncharacterized protein mRNA, complete cds
AT4G39790	1.102014225	0.013684037	Arabidopsis thaliana uncharacterized protein mRNA, complete cds
AT5G67020	1.102014225	0.013684037	Arabidopsis thaliana chromosome 5 sequence
AT5G22890	1.102014225	0.013684037	Arabidopsis thaliana chromosome 5 sequence
AT1G51960	2.955172837	0.014847274	Arabidopsis thaliana IQ-domain 27 protein mRNA, complete cds
AT3G20087	2.955172837	0.014847274	Arabidopsis thaliana chromosome 3, complete sequence
AT3G26816	2.955172837	0.014847274	Arabidopsis thaliana chromosome 3, complete sequence
AT1G64560	2.955172837	0.014847274	Arabidopsis thaliana chromosome 1 sequence
AT1G28830	2.955172837	0.014847274	Theobroma cacao Uncharacterized protein (TCM_007238) mRNA, complete cds
AT2G01008	2.955172837	0.014847274	Arabidopsis thaliana uncharacterized protein mRNA, complete cds
AT4G09130	1.540135337	0.014948938	Arabidopsis thaliana chromosome 4 sequence

Supplementary Data S10 Down regulated genes in *fc1*/Col-o with Cd treatment.

Gene	Log2FoldChange	pval	NT:Description
AT1G35710	-1.221280208	9.43E-186	Arabidopsis thaliana putative leucine-rich repeat receptor-like protein kinase mRNA, complete cds
AT5G26030	-2.052080547	2.02E-111	Arabidopsis thaliana ferrochelatase 1 mRNA, complete cds
AT1G77120	-2.026679817	2.23E-104	Arabidopsis thaliana alcohol dehydrogenase 1 mRNA, complete cds
AT1G66100	-1.026925472	4.66E-71	Arabidopsis thaliana probable thionin-2.4 mRNA, complete cds
AT1G67560	-1.167546322	6.66E-47	Arabidopsis thaliana lipoxygenase 6 mRNA, complete cds
AT3G43190	-1.69194614	5.37E-44	Arabidopsis thaliana sucrose synthase 4 mRNA, complete cds
AT1G13609	-1.337251264	5.29E-38	Arabidopsis thaliana defensin-like protein 287 mRNA, complete cds
AT2G41240	-1.21216437	2.23E-30	Arabidopsis thaliana transcription factor bHLH100 mRNA, complete cds
AT5G28090	-7.614682772	1.14E-22	Arabidopsis thaliana uncharacterized protein mRNA, complete cds
AT4G33070	-1.244558987	5.07E-22	Arabidopsis thaliana pyruvate decarboxylase 1 mRNA, complete cds
AT5G37990	-1.079097066	7.12E-20	Arabidopsis thaliana probable S-adenosylmethionine-dependent methyltransferase mRNA, complete cds
AT5G28464	-7.254280529	1.79E-18	Arabidopsis thaliana chromosome 5 sequence
AT1G56120	-1.098934569	5.41E-16	Arabidopsis thaliana Leucine-rich repeat transmembrane protein kinase mRNA, complete cds
AT2G04070	-1.062421223	2.18E-15	Arabidopsis thaliana MATE efflux family protein mRNA, complete cds
AT1G65790	-1.015340399	9.57E-15	Arabidopsis thaliana receptor-like serine/threonine-protein kinase SD1-7 mRNA, complete cds

AT2G15042	-1.533574349	1.85E-14	Arabidopsis thaliana Leucine-rich repeat (LRR) family protein mRNA, complete cds
AT2G01010	-1.701174297	4.86E-14	Arabidopsis thaliana chromosome 2, complete sequence
AT5G48010	-2.763645411	5.62E-14	Arabidopsis thaliana thalianol synthase 1 mRNA, complete cds
AT5G35760	-4.187785117	3.12E-13	Arabidopsis thaliana Beta-galactosidase related protein mRNA, complete cds
AT5G65080	-2.075854059	3.97E-12	Arabidopsis thaliana protein MADS AFFECTING FLOWERING 5 mRNA, complete cds
AT5G48070	-1.26205788	8.35E-12	Arabidopsis thaliana xyloglucan endotransglucosylase/hydrolase protein 20 mRNA, complete cds
AT3G56970	-1.066966141	7.80E-10	Arabidopsis thaliana transcription factor ORG2 mRNA, complete cds
AT1G49200	-1.249646955	6.74E-09	Arabidopsis thaliana chromosome 1 sequence
AT1G58225	-1.070979451	1.36E-08	Arabidopsis thaliana uncharacterized protein mRNA, complete cds
AT5G55150	-2.044827163	1.43E-08	Arabidopsis thaliana uncharacterized protein mRNA, complete cds
AT3G22740	-1.020848301	1.58E-08	Arabidopsis thaliana homocysteine S-methyltransferase 3 mRNA, complete cds
AT2G25625	-2.022107087	2.34E-08	Arabidopsis thaliana uncharacterized protein mRNA, complete cds
AT2G14610	-1.750083898	3.23E-08	Arabidopsis thaliana chromosome 2, complete sequence
AT1G29715	-1.934644246	5.80E-08	Arabidopsis thaliana mRNA for hypothetical protein, complete cds, clone: RAFL21-67-K19
AT1G26390	-1.533574349	6.06E-08	Arabidopsis thaliana chromosome 1 sequence
AT5G04150	-1.239474594	5.36E-07	Arabidopsis thaliana transcription factor bHLH101 mRNA, complete cds
AT3G44970	-1.781792758	5.54E-07	Arabidopsis thaliana cytochrome

			P450 family protein mRNA, complete cds
AT5G15960	-1.311107229	1.13E-06	Arabidopsis thaliana cold and ABA inducible protein kin1 mRNA, complete cds
AT2G30766	-1.324935083	1.62E-06	Arabidopsis thaliana chromosome 2, complete sequence
AT2G15040	-1.222365349	1.92E-06	Arabidopsis thaliana chromosome 2, complete sequence
AT1G51780	-1.929349946	2.28E-06	Arabidopsis thaliana IAA-amino acid hydrolase ILR1-like 5 mRNA, complete cds
AT2G36255	-3.629789664	2.83E-06	Arabidopsis thaliana putative defensin-like protein 203 mRNA, complete cds
AT4G23310	-1.084355528	2.92E-06	Arabidopsis thaliana putative cysteine-rich receptor-like protein kinase 23 mRNA, complete cds
AT2G17850	-4.437144586	3.23E-06	Arabidopsis thaliana rhodanese-like domain-containing protein 17 mRNA, complete cds
AT2G22410	-1.583247078	3.44E-06	Arabidopsis thaliana chromosome 2, complete sequence
AT1G12805	-1.717252505	6.83E-06	Arabidopsis thaliana chromosome 1 sequence
AT2G34390	-1.058632963	8.18E-06	Arabidopsis thaliana aquaporin NIP2-1 mRNA, complete cds
AT3G47720	-4.292754677	1.12E-05	Arabidopsis thaliana probable inactive poly [ADP-ribose] polymerase SRO4 mRNA, complete cds
AT4G33560	-1.400970974	1.41E-05	Arabidopsis thaliana chromosome 4 sequence
AT3G28510	-1.017860116	1.43E-05	Arabidopsis thaliana chromosome 3, complete sequence
AT2G10931	-1.530253991	1.43E-05	Arabidopsis thaliana chromosome 2, complete sequence
AT2G44798	-5.132290005	1.74E-05	Arabidopsis thaliana mRNA for hypothetical protein, complete cds, clone: RAFL16-84-G21
AT1G33720	-1.303561432	2.61E-05	Arabidopsis thaliana cytochrome P450, family 76, subfamily C, polypeptide 6 mRNA, complete

			cds
AT5G10040	-5.044827163	3.20E-05	Arabidopsis thaliana chromosome 5 sequence
AT5G38780	-3.366755258	3.32E-05	Arabidopsis thaliana putative S-adenosylmethionine-dependent methyltransferase mRNA, complete cds
AT4G14368	-2.089221283	3.66E-05	Arabidopsis thaliana regulator of chromosome condensation repeat-containing protein mRNA, complete cds
AT2G18190	-2.191668552	3.74E-05	Arabidopsis thaliana P-loop containing nucleoside triphosphate hydrolases superfamily protein mRNA, complete cds
AT1G52570	-1.079592582	5.04E-05	Arabidopsis thaliana phospholipase D alpha 2 mRNA, complete cds
AT5G15500	-1.044827163	5.35E-05	Arabidopsis thaliana chromosome 5 sequence
AT1G13608	-2.044827163	6.09E-05	Arabidopsis thaliana putative defensin-like protein 288 mRNA, complete cds
AT2G45760	-1.267219585	6.63E-05	Arabidopsis thaliana chromosome 2, complete sequence
AT1G26790	-1.204025758	7.76E-05	Arabidopsis thaliana Dof-type zinc finger DNA-binding family protein mRNA, complete cds
AT2G12460	-1.919296281	9.41E-05	Arabidopsis thaliana chromosome 2, complete sequence
AT5G46440	-1.244135972	9.59E-05	Arabidopsis thaliana chromosome 5 sequence
AT3G62510	-2.56838912	9.66E-05	Arabidopsis thaliana protein disulfide isomerase-like protein mRNA, complete cds
AT3G59270	-2.366755258	0.000104895	Arabidopsis thaliana FBD domain-containing protein mRNA, complete cds
AT4G18490	-1.064726721	0.000186491	Arabidopsis thaliana uncharacterized protein mRNA, complete cds
AT3G23010	-1.007352458	0.000269221	Arabidopsis thaliana chromosome 3, complete sequence

AT5G15340	-1.992359744	0.000293546	Arabidopsis thaliana chromosome 5 sequence
AT5G22540	-2.437144586	0.000298619	Arabidopsis thaliana chromosome 5 sequence
AT4G15200	-1.553840811	0.000361355	Arabidopsis thaliana formin 3 mRNA, complete cds
AT1G45545	-1.117583506	0.000381409	Arabidopsis thaliana uncharacterized protein mRNA, complete cds
AT1G26976	-4.629789664	0.000382757	Arabidopsis thaliana uncharacterized protein mRNA, complete cds
AT5G15940	-3.044827163	0.000385727	Arabidopsis thaliana Rossmann-fold NAD(P)-binding domain-containing protein mRNA, complete cds
AT3G47790	-1.267219585	0.000434852	Arabidopsis thaliana ABC transporter A family member 8 mRNA, complete cds
AT5G55470	-1.044827163	0.000467679	Arabidopsis thaliana sodium/hydrogen exchanger 4 mRNA, complete cds
AT4G24860	-2.044827163	0.000516529	Arabidopsis thaliana P-loop containing nucleoside triphosphate hydrolases superfamily protein mRNA, complete cds
AT5G28085	-2.366755258	0.000521494	Arabidopsis thaliana chromosome 5 sequence
AT1G31290	-1.585395545	0.00076275	Arabidopsis thaliana argonaute 3 mRNA, complete cds
AT4G22960	-1.246461025	0.000765916	Arabidopsis thaliana uncharacterized protein mRNA, complete cds
AT4G16220	-2.547327504	0.000847866	Arabidopsis thaliana GDSL esterase/lipase mRNA, complete cds
AT3G30720	-2.115216491	0.000903856	Arabidopsis thaliana qua-quine starch mRNA, complete cds
AT3G52115	-1.099274947	0.000972848	Arabidopsis thaliana protein gamma response 1 mRNA, complete cds
AT5G55460	-1.020579617	0.001175653	Arabidopsis thaliana bifunctional inhibitor/lipid-transfer

			protein/seed storage 2S albumin superfamily protein mRNA, complete cds
AT4G09432	-2.459864663	0.001505379	Arabidopsis thaliana chromosome 4 sequence
AT5G57720	-2.459864663	0.001505379	Arabidopsis thaliana AP2/B3-like transcriptional factor family protein mRNA, complete cds
AT3G19310	-2.459864663	0.001505379	Arabidopsis thaliana PLC-like phosphodiesterase-like protein mRNA, complete cds
AT1G55700	-2.044827163	0.001526912	Arabidopsis thaliana chromosome 1 sequence
AT4G11140	-1.044827163	0.001552875	Arabidopsis thaliana chromosome 4 sequence
AT2G17740	-2.214752165	0.001565596	Arabidopsis thaliana chromosome 2, complete sequence
AT1G36640	-3.504258782	0.001759384	Arabidopsis thaliana uncharacterized protein mRNA, complete cds
AT5G07700	-1.106227708	0.001774837	Arabidopsis thaliana myb domain protein 76 mRNA, complete cds
AT4G21300	-1.106227708	0.001774837	Arabidopsis thaliana chromosome 4 sequence
AT2G26520	-1.307861569	0.001999707	Arabidopsis thaliana chromosome 2, complete sequence
AT1G61255	-1.761034197	0.002106725	Arabidopsis thaliana chromosome 1 sequence
AT4G08210	-1.530253991	0.002153204	Arabidopsis thaliana chromosome 4 sequence
AT3G18217	-1.108957501	0.002170713	Arabidopsis thaliana chromosome 3, complete sequence
AT1G61440	-1.852182086	0.002352639	Arabidopsis thaliana G-type lectin S-receptor-like serine/threonine-protein kinase mRNA, complete cds
AT1G44120	-1.852182086	0.002352639	Arabidopsis thaliana armadillo/beta-catenin-like repeat and C2 calcium/lipid-binding domain-containing protein mRNA, complete cds
AT4G37780	-1.970826582	0.002562225	Arabidopsis thaliana myb domain protein 87 mRNA, complete cds

AT3G46190	-4.214752165	0.002621708	Arabidopsis thaliana TRAF-like family protein mRNA, complete cds
AT3G50870	-1.044827163	0.002848524	Arabidopsis thaliana GATA transcription factor 18 mRNA, complete cds
AT5G65090	-1.044827163	0.002848524	Arabidopsis thaliana protein DEFORMED ROOT HAIRS 4 mRNA, complete cds
AT5G41761	-1.267219585	0.002946822	Arabidopsis thaliana chromosome 5 sequence
AT1G02230	-1.267219585	0.002946822	Arabidopsis thaliana NAC domain-containing protein 4 mRNA, complete cds
AT2G47460	-1.477786571	0.003322315	Arabidopsis thaliana transcription factor MYB12 mRNA, complete cds
AT3G01870	-3.366755258	0.003339826	Arabidopsis thaliana chromosome 3, complete sequence
AT2G39510	-1.160304381	0.003417259	Arabidopsis thaliana nodulin MtN21-like transporter family protein mRNA, complete cds
AT1G72070	-1.366755258	0.003521474	Arabidopsis thaliana chaperone DnaJ-domain containing protein mRNA, complete cds
AT3G15536	-1.214752165	0.003534622	Arabidopsis thaliana chromosome 3, complete sequence
AT1G03935	-1.281866361	0.003579765	Arabidopsis thaliana chromosome 1 sequence
AT5G52740	-1.518758352	0.00393687	Arabidopsis thaliana copper transport family protein mRNA, complete cds
ATMG01170	-1.118827745	0.003984183	Arabidopsis thaliana ecotype C24 mitochondrion, complete genome
AT1G18000	-1.118827745	0.003984183	Arabidopsis thaliana chromosome 1 sequence
AT1G22900	-1.166817688	0.004180694	Arabidopsis thaliana chromosome 1 sequence
AT4G37608	-1.392750467	0.004238003	Arabidopsis thaliana uncharacterized protein mRNA, complete cds
AT1G11340	-1.044827163	0.004281962	Arabidopsis thaliana G-type lectin S-receptor-like

			serine/threonine-protein kinase mRNA, complete cds
AT1G74650	-1.122829675	0.004883967	Arabidopsis thaliana myb domain protein 31 mRNA, complete cds
AT5G40310	-4.044827163	0.005049954	Arabidopsis thaliana exonuclease family protein mRNA, complete cds
AT1G06540	-4.044827163	0.005049954	Arabidopsis thaliana uncharacterized protein mRNA, complete cds
AT5G40860	-4.044827163	0.005049954	Arabidopsis thaliana uncharacterized protein mRNA, complete cds
AT4G37810	-1.17411018	0.005116913	Arabidopsis thaliana uncharacterized protein mRNA, complete cds
AT1G34580	-1.629789664	0.005408839	Arabidopsis thaliana sugar transport protein 5 mRNA, complete cds
AT4G15370	-1.629789664	0.005408839	Arabidopsis thaliana baruol synthase 1 mRNA, complete cds
AT5G03790	-1.707792176	0.006233168	Arabidopsis thaliana homeobox 51 mRNA, complete cds
AT1G22600	-3.214752165	0.006347672	Arabidopsis thaliana Late embryogenesis abundant protein (LEA) family protein mRNA, complete cds
AT2G03130	-3.214752165	0.006347672	Arabidopsis thaliana chromosome 2, complete sequence
AT4G00390	-3.214752165	0.006347672	Arabidopsis thaliana chromosome 4 sequence
AT5G17150	-3.214752165	0.006347672	Arabidopsis thaliana Cystatin/monellin superfamily protein mRNA, complete cds
AT2G40200	-1.044827163	0.006456152	Arabidopsis thaliana transcription factor bHLH51 mRNA, complete cds
AT1G68460	-1.81036191	0.007052221	Arabidopsis thaliana chromosome 1 sequence
AT1G06310	-1.81036191	0.007052221	Arabidopsis thaliana putative acyl-CoA oxidase mRNA, complete cds
AT3G06600	-1.366755258	0.007724792	Arabidopsis thaliana uncharacterized protein mRNA,

			complete cds
AT3G13900	-1.951717759	0.007755975	Arabidopsis thaliana putative phospholipid-transporting ATPase 7 mRNA, complete cds
AT5G19880	-2.504258782	0.007851081	Arabidopsis thaliana peroxidase mRNA, complete cds
AT4G39600	-1.044827163	0.007937843	Arabidopsis thaliana chromosome 4 sequence
AT3G27473	-1.044827163	0.007937843	Arabidopsis thaliana chromosome 3, complete sequence
AT5G52290	-2.160304381	0.008140872	Arabidopsis thaliana protein SHORTAGE IN CHIASMATA 1 mRNA, complete cds
AT5G47850	-1.559400336	0.008558166	Arabidopsis thaliana chromosome 5 sequence
AT1G12030	-1.20236844	0.009406171	Arabidopsis thaliana uncharacterized protein mRNA, complete cds
ATMG00665	-3.852182086	0.009805325	.
AT3G09620	-3.852182086	0.009805325	Arabidopsis thaliana DEAD-box ATP-dependent RNA helicase 45 mRNA, complete cds
AT2G24681	-3.852182086	0.009805325	Arabidopsis thaliana AP2/B3-like transcriptional factor family protein mRNA, complete cds
AT5G23270	-1.629789664	0.01001592	Arabidopsis thaliana sugar transport protein 11 mRNA, complete cds
AT4G22230	-1.144362837	0.011107633	Arabidopsis thaliana defensin-like protein 96 mRNA, complete cds
AT1G66570	-1.437144586	0.011209795	Arabidopsis thaliana putative sucrose transport protein SUC7 mRNA, complete cds
AT5G52220	-1.437144586	0.011209795	Arabidopsis thaliana chromosome transmission fidelity protein 8 domain-containing protein mRNA, complete cds
AT3G52072	-1.722899069	0.011549217	Arabidopsis thaliana mRNA for hypothetical protein, complete cds, clone: RAFL21-36-M10
AT3G19230	-1.307861569	0.01162926	Arabidopsis thaliana leucine-rich repeat-containing protein mRNA, complete cds
AT5G08150	-3.044827163	0.012073485	Arabidopsis thaliana chromosome

			5 sequence
AT3G26820	-3.044827163	0.012073485	Arabidopsis thaliana Esterase/lipase/thioesterase family protein mRNA, complete cds
AT4G01533	-3.044827163	0.012073485	Arabidopsis thaliana Full-length cDNA Complete sequence from clone GSLTPGH70ZB05 of Hormone Treated Callus of strain col-0 of Arabidopsis thaliana (thale cress)
AT1G47370	-3.044827163	0.012073485	Arabidopsis thaliana Toll-Interleukin-Resistance domain-containing protein mRNA, complete cds
AT1G54470	-3.044827163	0.012073485	Arabidopsis thaliana resistance to Peronospora parasitica 27 mRNA, complete cds
AT1G36620	-3.044827163	0.012073485	Arabidopsis thaliana chromosome 1 sequence
AT1G49790	-1.852182086	0.013009144	Arabidopsis thaliana F-box associated ubiquitination effector family protein mRNA, complete cds
ATCG01050	-1.852182086	0.013009144	Arabidopsis thaliana chloroplast DNA, complete genome, ecotype: Columbia
AT4G08250	-1.852182086	0.013009144	Arabidopsis thaliana chromosome 4 sequence
AT3G50940	-1.852182086	0.013009144	Arabidopsis thaliana cytochrome BC1 synthesis-like protein mRNA, complete cds
AT1G26870	-1.852182086	0.013009144	Arabidopsis thaliana protein FEZ mRNA, complete cds
AT1G42980	-1.485399755	0.013415718	Arabidopsis thaliana formin-like protein 12 mRNA, complete cds
AT2G26040	-1.004185179	0.013664667	Arabidopsis thaliana chromosome 2, complete sequence
AT1G16515	-2.044827163	0.014084584	Arabidopsis thaliana chromosome 1 sequence
AT5G60180	-2.044827163	0.014084584	Arabidopsis thaliana chromosome 5 sequence
AT4G21200	-2.044827163	0.014084584	Arabidopsis thaliana gibberellin 2-beta-dioxygenase 8 mRNA,

			complete cds
AT1G55980	-1.334333781	0.014151444	Arabidopsis thaliana FAD/NAD(P)-binding oxidoreductase domain-containing protein mRNA, complete cds
AT4G09780	-2.366755258	0.014155906	Arabidopsis thaliana TRAF-like family protein mRNA, complete cds

Supplementary Data S11. Primer and probe sequences used for this study

Primer name	Forward (5'-3')	Reverse (5'-3')
AtFC1	TGCTATTGGTGGTGGCTCT	GTCGGTGATGAAGGATGGT
ACTIN	TCGTTTCGCTTTCCTTAG	CTTCACCATTCCAGTTCC

Supplementary Data 11B. Primer sequences used for mutant identification

Primer name	
LP-1	TTTTGGATTGAGGAACTTTTACAAC
RP-1	AAACTCAATCCACGATTCGTG
LBb1.3	ATTTTGCCGATTTCGGAAC

Supplementary Data 11C. Primer sequences used for quantitative real-time RT-PCR analysis

	Forward (5'-3')	Reverse (5'-3')
FC1-Real	ACCAAAGGCGAGGCTATGTCAATTC	TCTGCTTCTGGTACGGATCTCCA G
AtGSH1-Real	GATGGTTTAGAGCGCAGAGG	TACGCTTTGTCCCCATTCTC
AtGSH2-Real	ACCAACTGCATTCCCAGAAG	GCCATCCAAGCTAACACGAT
AtPCS1-Real	TCCGTACAAGTCAGAGCACCAT	GTGACCAGTCCCAGTCTGCTTA
AtPCS2-Real	CCGCACAAATCAGAGCACCAT	GCCAGTTCCAGTCTGCTTGAG
AtGR1-Real	ACAAGCGGTTATTGGCAAATGC	TTGTCTCACCTCCACTTCGTTG
AtGR2-Real	GCTTCGCCACTAGCTTCGGT	TCCAACGCCTCCAGCAGTATC
AtPDR8-Real	TGGGTCCTCCTTCTTCTGGTAA	GCAGAGGTCTTTCTCGGAACAA
ACTIN2-Real	CTCTCCTTGTACGCCAGTGGTC	TAAGGTCACGTCCAGCAAGGTC
